# Causal associations of brain structure with bone mineral density: a large-scale genetic correlation study

**DOI:** 10.1038/s41413-023-00270-z

**Published:** 2023-07-20

**Authors:** Bin Guo, Chao Wang, Yong Zhu, Zhi Liu, Haitao Long, Zhe Ruan, Zhangyuan Lin, Zhihua Fan, Yusheng Li, Shushan Zhao

**Affiliations:** 1 Department of Orthopedics, Xiangya Hospital, Central South University, Changsha, 410008 Hunan China; 2 Department of Dermatology, Xiangya Hospital, Central South University, Changsha, China; 3 National Clinical Research Center for Geriatric Disorders, Xiangya Hospital, Central South University, Changsha, Hunan 410008 China

**Keywords:** Neurophysiology, Bone quality and biomechanics

## Abstract

In this study, we aimed to investigate the causal associations of brain structure with bone mineral density (BMD). Based on the genome-wide association study (GWAS) summary statistics of 1 325 brain imaging-derived phenotypes (BIDPs) of brain structure from the UK Biobank and GWAS summary datasets of 5 BMD locations, including the total body, femoral neck, lumbar spine, forearm, and heel from the GEFOS Consortium, linkage disequilibrium score regression (LDSC) was conducted to determine the genetic correlations, and Mendelian randomization (MR) was then performed to explore the causal relationship between the BIDPs and BMD. Several sensitivity analyses were performed to verify the strength and stability of the present MR outcomes. To increase confidence in our findings, we also performed confirmatory MR between BIDPs and osteoporosis. LDSC revealed that 1.93% of BIDPs, with a false discovery rate (FDR) < 0.01, were genetically correlated with BMD. Additionally, we observed that 1.31% of BIDPs exhibited a significant causal relationship with BMD (FDR < 0.01) through MR. Both the LDSC and MR results demonstrated that the BIDPs “Volume of normalized brain,” “Volume of gray matter in Left Inferior Frontal Gyrus, pars opercularis,” “Volume of Estimated Total Intra Cranial” and “Volume-ratio of brain segmentation/estimated total intracranial” had strong associations with BMD. Interestingly, our results showed that more left BIDPs were causally associated with BMD, especially within and around the left frontal region. In conclusion, a part of the brain structure causally influences BMD, which may provide important perspectives for the prevention of osteoporosis and offer valuable insights for further research on the brain-bone axis.

## Introduction

Osteoporosis, which can affect individuals of all ethnicities, is becoming more prevalent with the continuing aging of the global population.^1,2^ The most typical manifestation of osteoporosis is a reduction in bone mineral density (BMD), which elevates the risk of fractures in the spine, hip, distal radius, proximal humerus and other locations.^3^ Bone mass changes are caused by multiple factors, and the central nervous system can directly regulate bone mass, thus affecting bone metabolism.^4–6^ Classical neurotransmitters and a variety of neuropeptides from the brain and bone axis mediate the neural connection between brain and bone.^7,8^ With the in-depth study of neurodegenerative diseases such as Parkinson’s disease,^9^ spinal cord injury,^10^ amyotrophic lateral sclerosis,^11^ Alzheimer’s disease,^12^ postpolio syndrome^13^ and multiple sclerosis,^14^ it was found that osteoporosis is more common in patients with each neurodegenerative disease than in the general population. These neurodegenerative diseases are often accompanied by changes in brain structure. A recent GWAS has also shown that genetic loci associated with brain structure also exhibit enrichment in bone mineral density loci.^5^ An increasing number of studies have implied that brain and regional size may affect bone metabolism. Nevertheless, many uncertainties remain to be explored.

Brain imaging holds great potential for early disease prediction,^15^ and one of the best noninvasive tools to study the brain is magnetic resonance imaging (MRI). The UK Biobank links neuroimaging and genetics through an epidemiological study containing 500 000 participants.^16^ To help convert the collected raw imaging data into useful summary information, a fully automated processing flow was developed to generate processed images and brain image-derived phenotypes (BIDPs), which are distinct measures of brain structure and function that are useful for nonimaging experts.^17^

Despite the fact that randomized controlled trials are the gold standard for causal inference, ethical constraints or the high cost of trials limit their use.^18^ It is possible to overcome the limitations of observational research with the help of robust causal inference methods that have emerged over the last few decades. Linkage disequilibrium score regression (LDSC), as one of the most commonly used genetic correlation analysis methods,^19^ can be used to evaluate the genetic correlation between brain structure and BMD. Mendelian randomization (MR) analysis, which is less susceptible to bias from confounding factors and reverse causality,^20^ can be used to investigate the causal relationship between exposure (brain structure) and outcome (BMD).

In this study, LDSC analysis based on genome-wide association study (GWAS) summary statistical data was used to evaluate the genetic associations of brain regional and tissue volume and cortical area and thickness with BMD as affected by heredity. To determine the causal role of brain structure on BMD, MR approaches were also conducted by using large GWAS summary data, including BIDPs^21^ of each brain region measured by MRI and BMD^22^ at different sites examined with dual energy X-ray (total body, lumbar spine, femoral neck, and forearm), and by measuring quantitative heel ultrasound (heel BMD).

## Results

### Linkage disequilibrium score regression (LDSC)

**Fig. 1:**
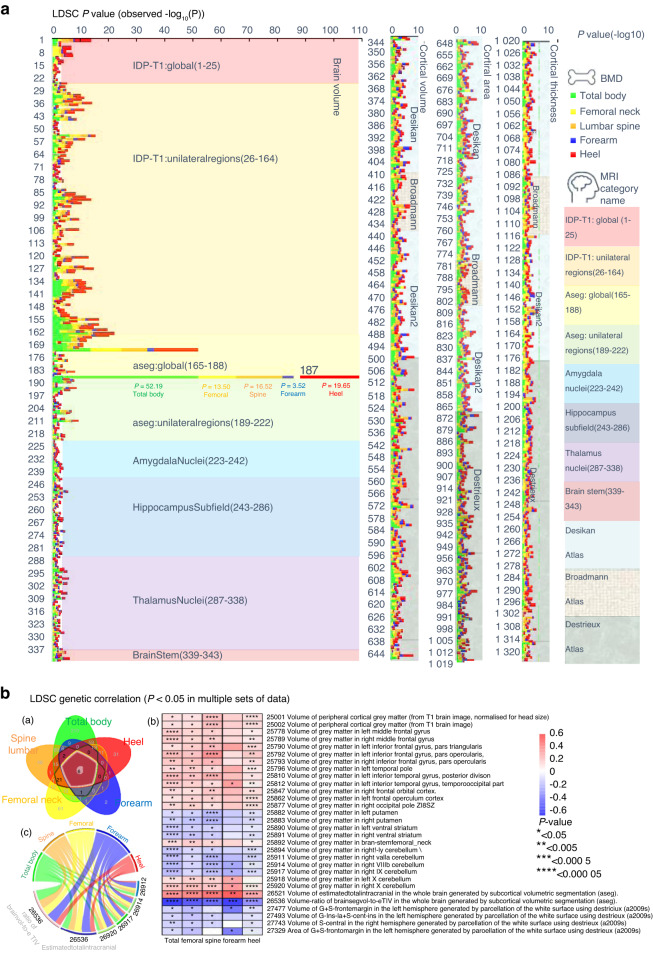
The genetic correlation between brain structure and BMD. **a** All the LDSC results between BIDPs and BMD. (Ordinate: BIDPs in UK Biobank numbering order, abscissa: - log10 *P* values). **b** (a) Venn diagram: all the LDSC results with a *P* < 0.05 in the respective sets. b Heatmap: genetic correlation between BMD and BIDPs with >3 intersecting elements in the Venn diagram. c Chord diagram: genetic correlation between BMD and BIDPs of which intersection = 5 elements in the Venn diagram

Cross-BIDP/BMD LDSC was applied to measure the genetic correlation between brain volume and BMD of different anatomical regions (Fig. 1 and Table S1). We used a *P* value less than 0.05 without adjustment as the threshold to screen the potential brain volume that affects BMD. The LDSC results identified phenotypes with potential genetic correlation as follows: 439 phenotypes with total body BMD, 156 phenotypes with femoral neck BMD, 201 phenotypes with lumbar spine BMD, 32 phenotypes with forearm BMD and 161 phenotypes with heel BMD (Table S1). Six BIDPs showed potential genetic correlation with all five BMD regions in our study (Fig. 1B), including (1) Volume-ratio of BrainSegVol-to-eTIV in the whole brain (UKB ID: 26536); (2) Volume of Estimated Total Intra Cranial in the whole brain (UKB ID: 26521); (3) Volume of gray matter in Right IX Cerebellum (UKB ID: 26917); (4) Volume of gray matter in Right VIIIb Cerebellum (UKB ID: 26914); (5) Volume of gray matter in Right X Cerebellum (UKB ID: 26920) and (6) Volume of gray matter in Left Inferior Temporal Gyrus (UKB ID: 25812).

Under false discovery rate (FDR) correction, the LDSC analyses found phenotypes with significant genetic correlation as follows: 78 phenotypes with total body BMD, 13 phenotypes with femoral neck BMD, 6 phenotypes with lumbar spine BMD, 1 phenotype with forearm BMD, and 30 phenotypes with heel BMD (Table S1). The BIDP of “Volume-ratio of BrainSegVol-to-eTIV” was also found to be genetically correlated with the total body, femoral neck, lumbar spine, forearm, and heel BMD.

### Two-sample Mendelian randomization

#### Overview outlines of the BIDPs with BMD

**Fig. 2:**
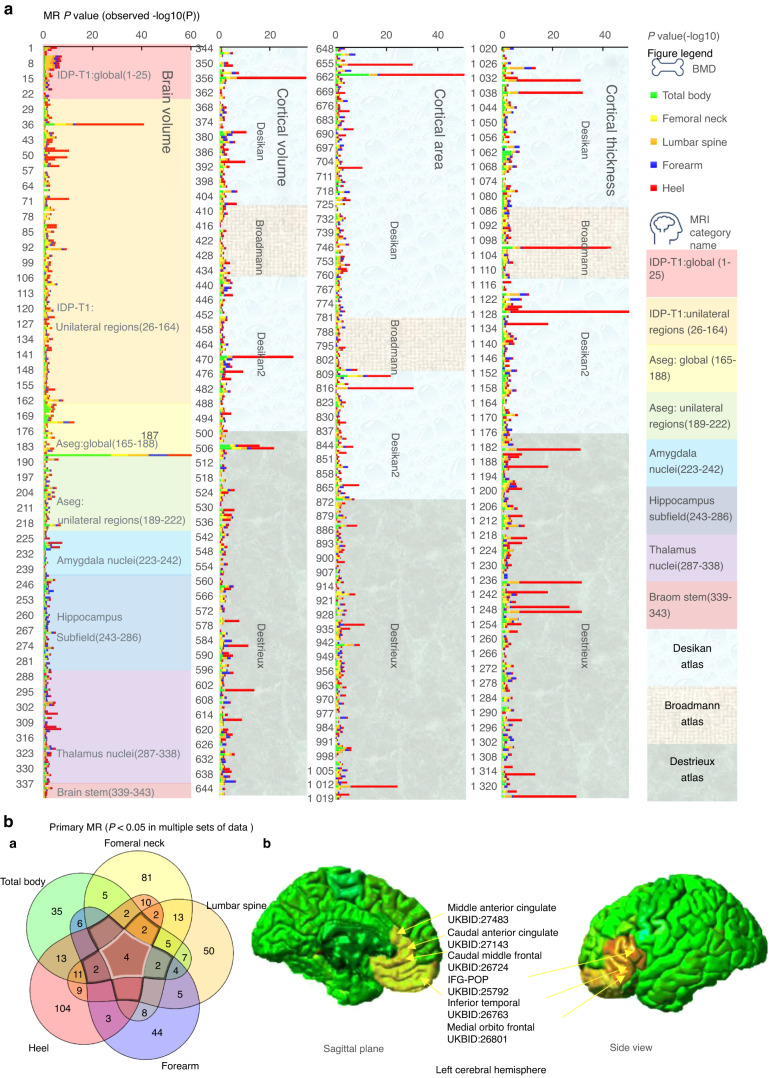
The potential causal relationship between BIDPs and BMD. **a** All MR results between BIDPs and BMD. (Ordinate: BIDPs in UK Biobank numbering order, abscissa: - log10 *P* values). **b** (a) Venn diagram: all the primary MR results among which *P* < 0.05 in respective sets. b Brain regions associated with BMD, labeled yellow, and overlaps labeled orange, intersection >3 elements in the Venn diagram

We used 2 sMR to find evidence for a potential causal relationship between BIDPs and BMD (Fig. 2 and Table S2). A *P* value less than 0.05 without adjustment was considered the threshold to screen the potential brain volume that causally affected BMD. The primary MR results identified 89 phenotypes with potential causal association with total body BMD, 117 phenotypes with potential causal association with femoral neck BMD, 106 phenotypes with potential causal association with lumbar spine BMD, 70 phenotypes with potential causal association with forearm BMD, and 148 phenotypes with potential causal association with heel BMD. Four brain structures were found to have a potential causal association with all five BMD regions in the study (Fig. 2B.a), including (1) Volume-ratio of BrainSegVol-to-eTIV in the whole brain (UKB ID:26536), (2) Volume of Estimated Total Intra Cranial in the whole brain (UKB ID:26521), (3) Volume of G + S-cingul-Mid-Ant in the left hemisphere (UKB ID:27483), and (4) Area of caudal anterior cingulate in the left hemisphere (UKB ID:27143). Figure 2 also identified IDPs associated with BMD in more than three regions. Notably, these brain structures represented by identified IDPs were all located near the left frontal lobe.

Under FDR correction, the MR analyses found significant causal associations as follows: 8 phenotypes with total body BMD, 4 phenotypes with femoral neck BMD, 6 phenotypes with lumbar spine BMD, 1 phenotype with forearm BMD and 66 phenotypes with heel BMD (Table S2). The top 5 correlations of the BIDPs to the BMD among each part are shown in Table 1. Meanwhile, we found that more left BIDPs were associated with BMD (67.9%).

**Table 1:** Mendelian randomization results of the top 5 correlations of the BIDPs to BMD among each part (FDR *P* < 0.01)

No.	UKBID	IDP.short.name	Category name	Outcome	Method	SNP	OR	*p*	FDR
187	26536	aseg_global_volume- ratio_BrainSegVol-to-eTIV	aseg:global	FN BMD	IVW	15	1.50	7.35E-08	1.69E-06
172	26521	aseg_global_volume_ EstimatedTotalIntraCranial	aseg:global	FN BMD	IVW	7	1.42	1.92E-04	2.20E-03
36	25792	IDP_T1_FAST_ROIs_ L_inf_front_gyrus_parsop	IDP T1	FN BMD	WR	1	1.96	2.33E-05	3.05E-03
9	25009	IDP_T1_SIENAX_ brain-normalized_volume	IDP T1:global	FN BMD	IVW	9	1.29	5.85E-04	7.73E-03
187	26536	aseg_global_volume- ratio_BrainSegVol-to-eTIV	aseg:global	Forearm BMD	IVW	15	0.51	1.11E-08	2.55E-07
661	26734	aparc-Desikan_ lh_area_medialorbitofrontal	Desikan Atlas	Heel BMD	WR	1	0.61	1.45E-34	2.73E-32
36	25792	IDP_T1_FAST_ROIs_ L_inf_front_gyrus_parsop	IDP T1	Heel BMD	WR	1	1.55	1.00E-28	1.31E-26
356	26801	aparc-Desikan_lh_ volume_medialorbitofrontal	Desikan Atlas	Heel BMD	WR	1	1.59	3.00E-28	3.51E-26
1 033	26768	aparc-Desikan_lh_ thickness_medialorbitofrontal	Desikan Atlas	Heel BMD	WR	1	0.66	9.89E-26	3.12E-24
1 038	26773	aparc-Desikan_lh_ thickness_parsorbitalis	Desikan Atlas	Heel BMD	WR	1	0.64	7.20E-26	3.12E-24
187	26536	aseg_global_v olume-ratio_BrainSegVol-to-eTIV	aseg:global	LS BMD	IVW	15	0.59	4.52E-09	1.04E-07
1 028	26763	aparc-Desikan_lh_t hickness_inferiortemporal	Desikan Atlas	LS BMD	IVW	4	0.64	1.61E-06	1.01E-04
1 214	27439	aparc-a2009s_lh_ thickness_G-temporal-inf	Destrieux Atlas	LS BMD	IVW	5	0.66	6.02E-05	7.34E-03
93	25849	IDP_T1_FAST_ROIs_R_ parahipp_gyrus_ant	IDP T1	LS BMD	IVW	2	1.81	6.24E-05	8.17E-03
1 237	27462	aparc-a2009s_lh_ thickness_S-oc-temp-lat	Destrieux Atlas	LS BMD	WR	1	0.54	1.74E-04	8.51E-03
187	26536	aseg_global_volume-ratio_BrainSegVol-to-eTIV	aseg:global	TB BMD	IVW	17	0.44	4.00E-28	9.20E-27
661	26734	aparc-Desikan_lh_ area_medialorbitofrontal	Desikan Atlas	TB BMD	WR	1	0.38	6.38E-14	1.20E-11
810	27143	aparc-DKTatlas_lh_ area_caudalanteriorcingulate	Desikan Atlas	TB BMD	WR	1	1.53	1.30E-05	1.22E-03
506	27483	aparc-a2009s_lh_ volume_G + S-cingul-Mid-Ant	Destrieux Atlas	TB BMD	WR	1	1.66	1.30E-05	1.61E-03
505	27482	aparc-a2009s_lh_ volume_G + S-cingul-Ant	Destrieux Atlas	TB BMD	WR	1	1.73	5.25E-05	3.26E-03

*IVW* inverse-variance weighted, *WR* Wald ratio, *FN BMD* femoral neck BMD, *LS BMD* lumbar spine BMD, *TB BMD* total body BMD

#### Phenotypes with shared causal pathways between BIDPs and BMD

**Fig. 3:**
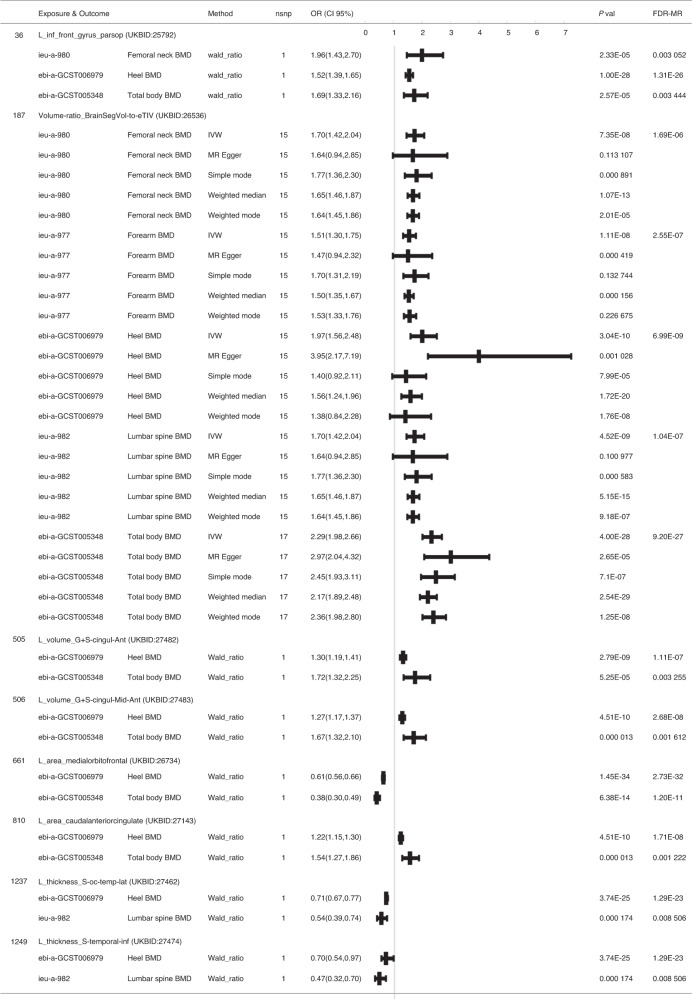
Effects of BIDPs on BMD. The results from MR analyses showing the phenotypes with shared causal relationships between the BIDPs and BMD of different anatomical regions (FDR *P* < 0.01)

In Fig. 3, we identify phenotypes with shared causal relationships between the BIDPs and BMD of different anatomical regions (FDR *P* < 0.01). In the primary analyses, we found that the BIDP “Volume-ratio of BrainSegVol-to-eTIV in the whole brain (UKB ID: 26536)” showed significant associations with all five regional BMDs. In addition, the BIDP regarding the volume of gray matter in the left inferior frontal gyrus pars opercularis (UKB ID: 25792) showed significant associations with total body, femoral neck, and heel BMD.

In addition, a genetically raised region of the medial orbitofrontal in the left hemisphere (UKB ID:26734) showed causal associations with decreased BMD of the total body and heel. The volume of G + S-cingul-Ant in the left hemisphere (UKB ID:27482), the volume of G + S-cingul-Mid-Ant in the left hemisphere (UKB ID:27483), and the area of caudal anterior cingulate in the left hemisphere (UKB ID:27143) were positively associated with the BMD of the total body and heel, and the mean thickness of S-oc-temp-lat in the left hemisphere (UKB ID:27462) and the mean thickness of S-temporal-inf in the left hemisphere (UKB ID:27474) had a significant causal association with the BMD of the heel and lumbar spine.

#### Sensitivity analysis

For each significant analysis in the primary MR, we also examined the results from the MR‒Egger analysis and the mode- and median-based methods to check consistency (Table S3). Except for the BIDPs of “Volume of gray matter (UKB ID: 25005),” “Volume of lateral orbitofrontal in the right hemisphere (UKB ID: 27307),” “Area of S-circular-insula-ant in the left hemisphere (UKB ID: 27375),” and “Mean thickness of isthmus cingulate in the right hemisphere (UKB ID: 26865),” consistent results were obtained using MR sensitivity analysis methods (weighted median, MR‒Egger, and weighted mode showed similar size and direction to IVW).

Furthermore, Table S4 shows the heterogeneous and pleiotropic effects. For the identified BIDPs, Cochran’s *Q*-test indicated that the MR findings of the BIDPs of “Volume of gray matter (UKB ID:25005),” “Volume of normalized brain (UKB ID:25009),” “Volume of Estimated Total Intra Cranial in the whole brain (UKB ID:26521),” “Volume-ratio of BrainSegVol-to-eTIV in the whole brain (UKB ID:26536),” “Volume of lateral orbitofrontal in the right hemisphere (UKB ID:27307),” and “Area of S-orbital-H-Shaped in the left hemisphere (UKB ID:27392)” were heterogeneous (*P* < 0.05). Thus, we performed random effect IVW, and the results were consistent (Table S2). MR-PRESSO was used to identify outliers and provide a corrected estimation. The recalculated MR estimates showed findings consistent with those above, and distortion tests had *P* > 0.05. According to the MR‒Egger intercept test, horizontal pleiotropy was not observed (*P* intercept > 0.05). Level pleiotropy and heterogeneity were visually assessed using funnel and scatter plots (see Additional File 1). Additionally, through MR Steiger, we did not find evidence of reverse causality (Table S4).

#### Validation of the association of BIDPs and osteoporosis

**Fig. 4:**
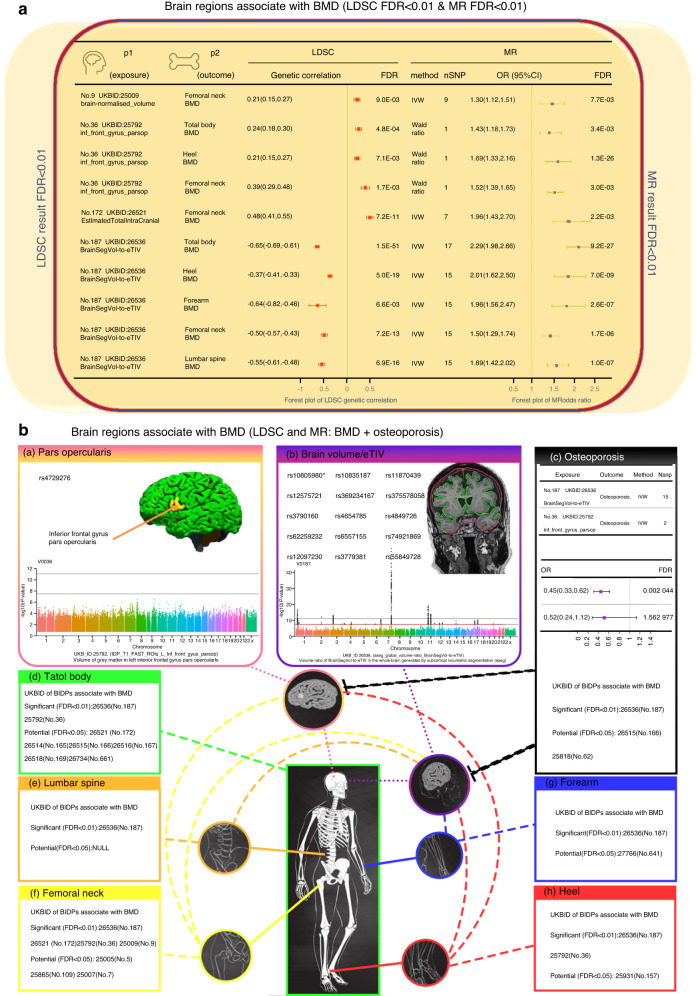
Brain regions associated with BMD and osteoporosis. **a** The forest plot of BIDPs associated with BMD (according to both the results of LDSC and MR FDR < 0.01). **b** The BIDPs that showed significant associations with BMD of different anatomical regions in both the LDSC and MR results (a, b): (a) Pars opercularis: SNP of MR result & Manhattan plot; (b) BrainSegVol-to-eTIV: SNP of MR result & Manhattan plot. (c) MR results and UKB ID of BIDPs for brain structures associated with osteoporosis. (d) UKB ID of BIDPs for brain structure associated with total body BMD. (e) UKB ID of BIDPs for brain structure associated with lumbar spine BMD. (f) UKB ID of BIDPs for brain structure associated with femoral neck BMD. (g) UKB ID of BIDPs for brain structure associated with forearm BMD. (h) UKB ID of BIDPs for brain structure associated with heel BMD

To enhance the credibility of our findings, we performed confirmatory 2 sMR between BIDPs and osteoporosis (Fig. 4 and Table S5). Focusing on the 5.7% potential causal BIDPs, we obtained MR results showing that the BIDP of “Volume-ratio of BrainSegVol-to-eTIV (UKB ID:26536)” was significantly negatively correlated with osteoporosis (OR = 0.45, FDR *P* = 2.28 × 10^−5^), indicating that each 1-SD increment in the volume-ratio of BrainSegVol-to-eTIV was predicted to decrease the risk of osteoporosis by 0.45. Moreover, the potential causal factors of brain volume that causally affect femoral neck, lumbar and total body BMD were further confirmed by the osteoporosis dataset (Table S5). Most of the BIDPs’ potentially causal associations with both BMD (mentioned above) and osteoporosis demonstrated opposite directions, while only the two BIDPs of “area of fusiform in the right hemisphere (UKB ID:26962)” and “area of BA2 in the left hemisphere (UKB ID:27060)” showed consistent directions. These two BIDPs are controversial and difficult to explain and will not be discussed in the remainder of this paper.

## Discussion

With a deeper understanding of the neural regulation of bone remodeling, researchers have identified a number of neural pathways that regulate bone metabolism through the central relay.^23^ Neurogenic control of bone metabolism was also confirmed in animal experiments, and a neural arm involved in bone remodeling was discovered.^24,25^ In this study, we sought to identify the genetic correlations and causal associations of brain structure with BMD. As a first step, LDSC was used identify 46.2% of BIDPs showing suggestive association signals with BMD (*P* < 0.05). Following this, MR was used to find a possible causal link between 5.7% of brain structure-related IDPs and BMD.

To date, the use of MR has succeeded in assessing causal relationships in pioneering studies of BMD. However, studies on the relationship between brain structure and bone are relatively rare. Although previous studies have suggested that the central nervous system is directly involved in bone health as a direct result of actions orchestrated primarily by the hypothalamus,^26,27^ hypothalamic volume was not associated with BMD according to our study. Here, as shown in Fig. 4, this study primarily focuses on the discussion of the BIDPs that showed significant associations in both the LDSC and MR results reported in this work.

Through further study, we found that the BIDP of “Volume of gray matter in left inferior frontal gyrus (IFG), pars opercularis (POP)” showed significant associations with total body, femoral neck, and heel BMD. The posterior part of the IFG of the left hemisphere is traditionally more often regarded as the classic Broca area, which is closely related to some aspects of expressive language processing.^28^ Studies have confirmed that the motion-related part of Broca’s area is mainly located within the POP.^29^ Meanwhile, recent neuroimaging studies have demonstrated that this area is of great significance for grasping, motor imagery, motion sequence learning, observing and preparing actions and imitation, and hierarchical organization of behavior controlling action segment selection.^30–35^ One study found that there was an increase in gray matter volume in Broca’s area, particularly on the left side of the POP, among male orchestrators.^29^ Stefanidou et al. found a link between BMD and verbal and visual memory.^36^ There has been considerable evidence in the past that the volume of the left IFG in neuropsychologically impaired patients is decreased. For example, through voxel-based morphometry, Woodward et al.^37^ found that there was a reduction in gray matter volume of the left IFG among neuropsychologically impaired patients. Studies have also shown that certain psychiatric disorders, such as Alzheimer’s disease, major depression, and bipolar disorder (BD), are associated with low BMD.^38–41^ However, in these previous studies, the possible role of medication or other related confounding factors has been difficult to accurately determine, thus indicating the limitations of those observational studies. Regional homogeneity (ReHo) is related to the pathophysiology of mental disorders as a data-driven method. A study^42^ focused on 40 drug-naive patients with BD and found that the patients with BD had a significant increase in ReHo values in the left IFG. Although this study did not find a link between abnormal ReHo and BMD (*P* > 0.05), the result may in part be explicable by a small sample size. Other studies have shown that patients with BD tend to have relatively low BMD, increasing their risk of fracture.^43,44^ These results indirectly suggest that there may be a potential causality between IFG and BMD. The results of our MR analysis rejected the interference of confounding factors, suggesting that the genetically predicted volume of gray matter in the left IFG and pars opercularis was positively correlated with BMD in the total body, femoral neck, and heel. This may result from the discrepancy in the IFG volume of gray matter leading to changes in regional activities, thus playing a partial role in the neural mechanism of BMD.

The BIDPs of “Volume of normalized brain (UKB ID: 25009)” and “Volume of Estimated Total Intra Cranial in the whole brain (UKB ID: 26521)” showed a significant positive correlation with femoral neck BMD according to LDSC and MR findings. The BIDP of “Volume-ratio of BrainSegVol-to-eTIV in the whole brain” showed a significant positive correlation with BMD in all five regions. Furthermore, FDR correction also showed a significant negative association with osteoporosis. The volume ratio of brain segmentation volume/estimated total intracranial volume (BrainSegVol-to-eTIV), which is the actual brain volume in the total intracranial volume was generated by subcortical volumetric segmentation. In this brain imaging-derived phenotype, 47 696 items of data are available, covering 43 173 participants. After removal of extreme values, the volume ratio of BrainSegVol-to-eTIV ranged from 0.665 102 to 0.892 374, and the median was 0.778 738. This ratio is the actual volume of brain-containing ventricles relative to the entire intracranial volume. During normal development, both intracranial volume (ICV) and total brain volume (TBV) increase rapidly and in tandem during early childhood but diverge during early adolescence. Over time, TBV declines gradually, while ICV remains stable into adulthood.^45,46^ Therefore, the difference between TBV and ICV is an indicator of normal age-related atrophy and later onset of pathological processes.^47^ Osteoporotic vertebral compression fracture (OVCF) is a prevalent complication of osteoporosis. A study^48^ using brain MRI to explore the relationship between brain volume and OVCFs in patients with osteoporosis found that after adjusting for confounding factors, the percentage of brain parenchymal volume (BPV/ICV) in OVCF patients was significantly decreased; this was consistent with our findings. A previous study^49^ on the relationship between BMD and brain atrophy in early AD patients found that higher BMD was associated with larger brain volume. Due to the inherent defect of traditional observational studies, the previous report might be a false-positive. In our study, the BIDP (UKB ID 25009), representing brain-normalized volume, showed a positive correlation with femoral neck BMD. In addition, according to our research results, we should pay more attention to the volume ratio rather than the simple volume of the brain.

Bae et al.^50^ found a linear relationship between BMD and brain parenchymal atrophy in a retrospective study. However, this retrospective study had certain limitations, such as the inability to determine the causal relationship and the inconsistent interval between DXA and brain MRI for all participants. Through LDSC and MR studies, we first confirmed the genetic correlation and causal relationship between brain parenchymal volume, total intracranial volume, and BMD. According to reports, type 1 collagen is an important component of bone matrix protein as well as arachnoid trabeculae and granules.^51^ Research has shown that osteoporosis is closely related to the genetic components of type 1 collagen, such as *COL1A1* and *COL1A2*.^52^ Meanwhile, a previous study found that brain atrophy in Alzheimer’s disease patients had a certain correlation with morphological changes in microvessels,^53^ while vascular smooth muscle is also composed of type 1 collagen to varying degrees. Therefore, we hypothesize that the decrease in BMD caused by brain atrophy (i.e., small volume ratio of BrainSegVol-to-eTIV) may be related to the decrease in the expression of genes such as *COL1A1* and *COL1A2*. In addition, several potential biological mechanisms may underlie the causal association between brain structure and BMD. One possible explanation is the shared genetic factors between brain and bone development, as both are influenced by a complex interplay of genetic and environmental factors. For example, Adams and his colleagues reported a GWAS about the novel genetic loci underlying human intracranial volume. This study found that the genetic loci associated with intracranial volume provided intriguing links between maximal brain size and various processes, including bone mineralization (*CENPW*), growth signaling (*IGF1, HMGA2*), DNA replication (*GMNC*) and rRNA maturation (*PDCD*).^5^ Another possible mechanism is the influence of hormonal factors, particularly estrogen, which has been shown to impact both brain development and bone metabolism.^54^ However, further research is needed to fully understand the underlying biological mechanisms of the association.

Interestingly, our results showed that more left BIDPs were associated with BMD. In addition, as shown in Fig. 2, significant associations of brain structure with BMD were found, especially within and around the left frontal region (area of left caudal anterior cingulate, volume of sulcus and gyrus of left middle anterior cingulate, volume of gray matter in left IFG, POP, volume of left medial orbitofrontal, cortical thickness of left inferior temporal, and area of left caudal middle frontal). Long believed to be mainly regulated by hormones, bone remodeling also responds to local mechanical stimulations. However, a continuing increase in recent evidence suggests that the central nervous system exerts a direct regulatory effect on bone homeostasis through efferent neural connections.^55^ Studies using various animal models and pharmacological approaches have shown that a variety of neurons, including leptin-responsive and neuropeptide Y-ergic neurons, are involved in the bone regulation of central signaling.^24,56,57^ We speculate that changes in the number of particular excitatory/inhibitory neurons caused by brain structure changes may affect the brain-bone regulatory axis, resulting in changes in BMD. Meanwhile, the difference in our study results may be attributed to the lateralization of brain function. Studies of lateralization in species such as fish, birds, and amphibians have shed light on key developmental events of the structure and function of the central nervous system.^58–60^ Lateralization of adult brain functions is well described in the aspects of language, visuospatial cognition, and hand-motor control.^61,62^ Recently, one of the meta-analyses of genome-wide association studies detected that structural lateralization in and around the planum temporale (the part of the brain responsible for language processing) is dimorphic (differing between males and females) in humans and associated with genes involved in steroid hormone biology.^63^ This suggests that the effects of the development of structural asymmetries in the brain associated with language processing may play a role in hormonal secretion. The other study focused on caudate asymmetry and found no significantly associated genetic polymorphisms.^64^ However, due to strict practical and ethical limitations on human research, lateralization remains largely mysterious despite its importance to many aspects of human function, such as cognition. Our findings may help identify subtle molecular variations between homologous left and right regions of the brain that may control fine-tuning of neuronal circuits for specific kinds of information processing. Contrasting the left regions at the genetic level against the right regions of natural control homology may help to understand some properties of cerebral cortical regions.

This study has several strengths. Neuroimaging measures can be considered endophenotypes as quantitative indicators of brain structure or function, indicating genetic responsibility.^65^ To the best of our knowledge, this study is the first to represent the initial assessment of the genetic links and causal connections between brain structure and BMD using LDSC and MR methodologies. The use of MR analysis minimizes the potential for confounding factors and reverse causation, as genetic variants are determined randomly at conception and therefore remain unaltered by environmental factors or illness. Our study is based on recent large-scale GWAS summary statistics data. The large sample sizes of GWAS could minimize the bias arising from the winner’s curse or weak instruments and lead to higher levels of statistical power.^66^ These advantages are beyond the reach of traditional observational studies. At the same time, we used a large sample set of osteoporosis data from the FinnGen Consortium to validate our results, further enhancing the credibility of the study.

Comprehending the correlation between brain structure and BMD is crucial for advancing future research and clinical practice. This relationship implies that interventions aiming to improve brain health may also benefit bone health. Furthermore, this highlights the necessity of a more integrated health care approach where multiple organ systems are treated collectively instead of separately. Investigating the underlying mechanisms connecting brain and bone health could also lead to the development of novel therapeutic targets for osteoporosis and related diseases.

However, there were still some limitations in this study. The ability to apply these findings to other populations is restricted due to the limited range of the study’s participants, who were solely of European descent. Therefore, it is essential to corroborate these findings in other populations. Further studies focused on the underlying mechanisms are required to verify this biological rationale, as the outcomes of both LDSC and MR analyses only suggest potential genetic connections and causal relationships at the genetic level.

In conclusion, our comprehensive large-scale correlational study provides evidence of causal associations of brain regional and tissue volume and cortical area and thickness with BMD. These results were directly or indirectly supported by many previously published studies. We believe that changes in brain structure could affect bone metabolism through certain pathways. However, to improve the prediction and prevention of osteoporosis, the specific mechanism needs to be further studied. We hope that this study can lay a foundation for research on the genetic mechanism of the bone-brain axis.

## Materials and methods

### Study population and genetic data

**Fig. 5:**
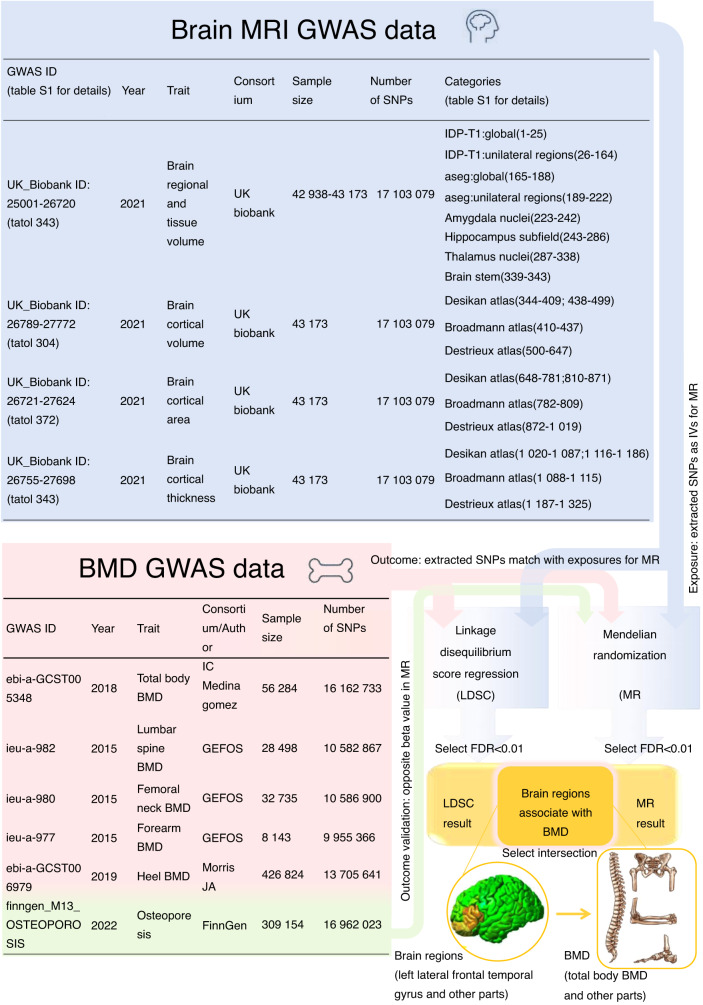
Flow chart of the study

Ethical approval and consent to participate were obtained for the original publications. Figure 5 provides an overview of the study design.

#### Brain structure data estimates from MRI

The brain regional and tissue volume and cortical area and thickness data were obtained from the UK Biobank. Researchers worldwide can access the raw and processed imaging data, BIDPs, and nonimaging measures in the UK Biobank by following a data access application procedure. The original data can be downloaded through the following website: https://open.win.ox.ac.uk/ukbiobank/big40/.

We selected 1 325 IDPs from the multimodal brain imaging dataset of European ancestry in the UK Biobank, which included 647 MRI phenotypes of brain regional and tissue volume, 372 MRI phenotypes of cortical area and 306 MRI phenotypes of cortical thickness.^67^ The UKB IDs and descriptions of the IDPs are shown in Table S6. This open data server contains results from GWAS of thousands of BIDPs, all single nucleotide polymorphisms (SNPs) tested, with a discovery sample of thousands of individuals (Fig. 5).

#### Bone mineral density

Summary statistics of BMD were obtained from the IEU GWAS database of MRC Comprehensive Epidemiology Institute of University of Bristol. The GEFOS Consortium (http://www.gefos.org) measured BMD data, and these data were collected in the IEU open GWAS database. The original data can be downloaded through the following website: https://gwas.mrcieu.ac.uk/.

We selected 5 BMD GWAS datasets of European ancestry, including total body BMD (*n* = 56 284), lumbar spine BMD (*n* = 28 498), femoral neck BMD (*n* = 32 735), forearm BMD (*n* = 8 143), and heel BMD (*n* = 426 824) (Fig. 5).

#### Osteoporosis

Based on a large meta-analysis of GWAS from the FinnGen Consortium, the data on osteoporosis included 309 154 males and females of European ancestry. The original data can be searched and downloaded through the following website: FinnGen https://www.finngen.fi.

### Linkage disequilibrium score regression (LDSC)

The determination of whether distinct traits share common genetic foundations can be achieved by quantifying the contribution of each element through an examination of the relationship between test statistics and LDSC.^68^ As a powerful approach for estimating heritability and genetic correlation from GWAS summary statistics, LDSC can be used to differentiate between real polygenicities and mixed biases (such as population stratification and implicit association).^69^ In this study, the initial stage involved estimating the genetic correlation using the cross-trait LDSC method, which was implemented through LDHub^70^ (https://github.com/bulik/ldsc). FDR correction was utilized with a significance level of *P* = 0.01 for multiple comparison correction.

### Mendelian randomization (MR)

#### Design

The MR approach employs genetic variants as IVs to deduce the potential causality of an exposure on an outcome. The heredity of genetic variation is not influenced by environmental factors or disease processes as it is determined randomly at the time of conception; hence, MR is less prone to bias caused by confounding factors and reverse causality.^20^ MR must meet the following three conditional assumptions: (1) IVs must be directly associated with BIDPs; (2) IVs are not associated with any confounding factor known to obscure the connection between BIDPs and BMD; (3) IVs should only be associated with BMD through BIDPs.^71^

In this study, brain regional and tissue volume and cortical area and thickness were used as exposure factors. SNPs significantly related to brain volume estimated from BIDPs were the IVs, and the outcome variable was BMD. Meanwhile, we used the osteoporosis dataset to verify our results.

#### Selection of genetic IVs

We utilized a genome-wide *P* value threshold of 5 × 10^−8^ to choose SNPs linked with BIDPs as IVs. PhenoScanner V2^72^ was used to evaluate the associations of IVs with potential confounding factors and exclude potential cases of pleiotropy. The confounding factors included alcohol intake frequency, fat mass, basal metabolic rate, body fat percentage, body mass index, height, hip circumference, lean body mass, waist circumference, weight, etc. (Table S7). IV intensity was measured using the F statistic of SNPs. Any IV with an F statistic < 10 was deemed weak and, therefore, removed from consideration. To perform a standard 2 sMR analysis, it was necessary to verify that the IVs were independent of each other (i.e., there is no significant linkage disequilibrium (LD)). The OpenGWAS API stores LD data from five populations (EUR, SAS, EAS, AFR, AMR); we selected European populations for the study. In reference to the entire genome of a thousand people, the aggregation and brain volume had unquestionable significance. To avoid interference from linkage disequilibrium (LD), a threshold value of 0.001 was established for the parameter R2, and a kilobase pair (KB) of 10 000 was applied. To compensate for missing SNPs, those with strong LD (R2 > 0.8) were utilized. However, SNPs that did not have alternative sites were removed from the analysis.

#### Primary and sensitivity MR analyses

We utilized several MR methods to investigate potential causal associations between BIDPs and BMD. We employed the Wald ratio and inverse-variance weighted (IVW) methods to calculate the primary MR estimates. In cases where only a single SNP was accessible, the Wald ratio method was utilized, taking into account that a modification in the outcome could be attributed to a unit change in the exposure.^73^ If multiple genetic variants were linked with the exposure variable under investigation, the IVW method was employed as the primary analysis since it provides the most precise estimation when all IVs are effective.^74^ To carry out sensitivity analyses, we employed several methods, including MR‒Egger regression, simple mode, weighted median, and weighted mode.

We conducted additional statistical tests to evaluate the presence of potential heterogeneity and pleiotropic effects. Cochran’s Q test was mainly used to test the differences between different IVs. If significant heterogeneity was identified (*P* of Cochran’s *Q* < 0.05), we employed the random-effect IVW method; otherwise, we used the fixed-effect IVW method. The pleiotropy test mainly determines whether multiple IVs have horizontal pleiotropy, and it is usually expressed by the intercept term of the MR–Egger method. If the intercept term is very different from 0, horizontal pleiotropy exists.^75^ Additionally, MR-PRESSO was used to evaluate pleiotropy, including tests for global, distortions, and outliers. In the meantime, we conducted the MR Steiger test to assess the possible presence of reverse causality.^76^

### Statistical analysis

To mitigate the risk of a type I error that arises with multiple hypothesis testing, it was crucial to uphold the original level of significance as closely as possible.^77^ We chose FDR correction to adjust probability *P* values so that we could make conservative conclusions.^78^

The following criteria were used to define causal effects: with FDR correction applied, IVW’s MR results met the multiple comparison adjusted *P* value criteria; the remaining MR methods showed similar size and direction to IVW; and there was no evidence of heterogeneity or gene-level pleiotropy after exclusion of potential outliers.

The statistical analyses were performed using R version 4.2.1 and several software packages, including knitr, plyr, two-sampleMR, and MRPRESSO. Statistical significance was determined by bilateral *P* values, and a *P* value less than 0.05 was considered suggestive, whereas highly reliable findings were survival values with an FDR threshold of 0.01 (LDSC and primary MR analysis).

## Supplementary information

Supplementary Table 7. The confounding factors screened by PhenoScanner V2

Supplementary Figure 1-36

Supplementary Table 1. Genetic correlation between BIDPs and BMD

Supplementary Table 2. Primary MR results of BIDPs on BMD (p less than 0.05)

Supplementary Table 3. Results of sensitivity analysis of MR

Supplementary Table 4. Results of the Cochran’s Q, MR-Egger-intercept, MR-PRESSO and MR steiger tests

Supplementary Table 5. Results of confirmatory 2sMR between BIDPs and osteoporosis

Supplementary Table 6. The UKB IDs and descriptions of the 1325 BIDPs

## References

[1] Lane NE (2006). Epidemiology, etiology, and diagnosis of osteoporosis. Am. J. Obstet. Gynecol..

[2] Kelsey JL (1989). Risk factors for osteoporosis and associated fractures. Public Health Rep..

[3] Black DM, Rosen CJ (2016). Clinical practice. Postmenopausal osteoporosis. N. Engl. J. Med..

[4] Maryanovich M, Takeishi S, Frenette PS (2018). Neural regulation of bone and bone marrow. Cold Spring Harb. Perspect Med.

[5] Adams HH (2016). Novel genetic loci underlying human intracranial volume identified through genome-wide association. Nat. Neurosci..

[6] Takano Y (2020). Hypoperfusion in the posterior cingulate cortex is associated with lower bone mass density in elderly women with osteopenia and Alzheimer’s disease. Clin. Exp. Pharmacol. Physiol..

[7] Qin W, Bauman WA, Cardozo CP (2010). Evolving concepts in neurogenic osteoporosis. Curr. Osteoporos. Rep..

[8] Roos PM (2014). Osteoporosis in neurodegeneration. J. Trace Elem. Med. Biol..

[9] Raglione LM, Sorbi S, Nacmias B (2011). Osteoporosis and Parkinson’s disease. Clin. Cases Min. Bone Metab..

[10] Antoniou G, Benetos IS, Vlamis J, Pneumaticos SG (2022). Bone mineral density post a spinal cord injury: a review of the current literature guidelines. Cureus.

[11] Caplliure-Llopis J (2020). Poor bone quality in patients with amyotrophic lateral sclerosis. Front. Neurol..

[12] Kumar S (2021). Alzheimer’s disease and its association with bone health: a case-control study. Cureus.

[13] Mohammad AF, Khan KA, Galvin L, Hardiman O, O’Connell PG (2009). High incidence of osteoporosis and fractures in an aging post-polio population. Eur. Neurol..

[14] Simonsen CS (2016). Bone mineral density in patients with multiple sclerosis, hereditary ataxia or hereditary spastic paraplegia after at least 10 years of disease - a case control study. BMC Neurol..

[15] Douaud G (2013). Brain microstructure reveals early abnormalities more than two years prior to clinical progression from mild cognitive impairment to Alzheimer’s disease. J. Neurosci. Off. J. Soc. Neurosci..

[16] Miller KL (2016). Multimodal population brain imaging in the UK Biobank prospective epidemiological study. Nat. Neurosci..

[17] Alfaro-Almagro F (2018). Image processing and Quality Control for the first 10,000 brain imaging datasets from UK Biobank. NeuroImage.

[18] Ma B (2021). Causal associations of anthropometric measurements with fracture risk and bone mineral density: a mendelian randomization study. J. Bone Miner. Res. Off. J. Am. Soc. Bone Miner. Res..

[19] Bulik-Sullivan BK (2015). LD Score regression distinguishes confounding from polygenicity in genome-wide association studies. Nat. Genet..

[20] Nitsch D (2006). Limits to causal inference based on Mendelian randomization: a comparison with randomized controlled trials. Am. J. Epidemiol..

[21] Raichle ME, Mintun MA (2006). Brain work and brain imaging. Annu. Rev. Neurosci..

[22] Blake GM, Fogelman I (2009). The clinical role of dual energy X-ray absorptiometry. Eur. J. Radio..

[23] Huang S (2019). Neural regulation of bone remodeling: Identifying novel neural molecules and pathways between brain and bone. J. Cell. Physiol..

[24] Takeda S (2002). Leptin regulates bone formation via the sympathetic nervous system. Cell.

[25] Baldock PA (2002). Hypothalamic Y2 receptors regulate bone formation. J. Clin. Investig..

[26] Harada S, Rodan GA (2003). Control of osteoblast function and regulation of bone mass. Nature.

[27] Takeda S (2009). Osteoporosis: a neuroskeletal disease. Int. J. Biochem. cell Biol..

[28] Grodzinsky, Y. & Amunts, K. *Broca’s region*. (Oxford University Press, 2006).

[29] Abdul-Kareem IA, Stancak A, Parkes LM, Sluming V (2011). Increased gray matter volume of left pars opercularis in male orchestral musicians correlate positively with years of musical performance. J. Magn. Reson. Imaging.: JMRI.

[30] Iacoboni M (1999). Cortical mechanisms of human imitation. Science.

[31] Rizzolatti G (1996). Localization of grasp representations in humans by PET: 1. Observation versus execution. Exp. Brain Res..

[32] Binkofski F (1999). A parieto-premotor network for object manipulation: evidence from neuroimaging. Exp. Brain Res..

[33] Krams M, Rushworth MF, Deiber MP, Frackowiak RS, Passingham RE (1998). The preparation, execution and suppression of copied movements in the human brain. Exp. Brain Res..

[34] Koski L (2002). Modulation of motor and premotor activity during imitation of target-directed actions. Cereb. Cortex.

[35] Koechlin E, Jubault T (2006). Broca’s area and the hierarchical organization of human behavior. Neuron.

[36] Stefanidou M (2021). Bone mineral density measurements and association with brain structure and cognitive function: the framingham offspring cohort. Alzheimer Dis. Assoc. Disord..

[37] Woodward ND, Heckers S (2015). Brain structure in neuropsychologically defined subgroups of schizophrenia and psychotic bipolar disorder. Schizophr. Bull..

[38] Misra M, Papakostas GI, Klibanski A (2004). Effects of psychiatric disorders and psychotropic medications on prolactin and bone metabolism. J. Clin. Psychiatry.

[39] Williams LJ (2011). The association between depressive and anxiety symptoms and bone mineral density in the general population: the HUNT Study. J. Affect. Disord..

[40] Zhou R, Deng J, Zhang M, Zhou HD, Wang YJ (2011). Association between bone mineral density and the risk of Alzheimer’s disease. J. Alzheimer’s Dis. JAD.

[41] Jung DU (2011). Bone mineral density and osteoporosis risk in older patients with schizophrenia. J. Clin. Psychopharmacol..

[42] Shan X (2020). Disrupted regional homogeneity in drug-naive patients with bipolar disorder. Front. Psychiatry.

[43] Chandrasekaran V (2017). Association between bipolar spectrum disorder and bone health: a meta-analysis and systematic review protocol. BMJ Open.

[44] Hsu CC (2016). Increased risk of fracture in patients with bipolar disorder: a nationwide cohort study. Soc. Psychiatry Psychiatr. Epidemiol..

[45] Courchesne E (2000). Normal brain development and aging: quantitative analysis at in vivo MR imaging in healthy volunteers. Radiology.

[46] Kamdar MR, Gomez RA, Ascherman JA (2009). Intracranial volumes in a large series of healthy children. Plast. Reconstr. Surg..

[47] Davis PJM, Wright EA (1977). A new method for measuring cranial cavity volume and its application to the assessment of cerebral atrophy at autopsy. Neuropathol. Appl. Neurobiol.

[48] Bae IS, Kim JM, Cheong JH, Han MH, Ryu JI (2019). Association between cerebral atrophy and osteoporotic vertebral compression fractures. PLoS One.

[49] Loskutova N, Honea RA, Vidoni ED, Brooks WM, Burns JM (2009). Bone density and brain atrophy in early Alzheimer’s disease. J. Alzheimer’s Dis. JAD.

[50] Bae IS, Kim JM, Cheong JH, Ryu JI, Han MH (2019). Association between bone mineral density and brain parenchymal atrophy and ventricular enlargement in healthy individuals. Aging.

[51] Saboori P, Sadegh A (2015). Histology and morphology of the brain subarachnoid trabeculae. Anat. Res. Int..

[52] Grant SF (1996). Reduced bone density and osteoporosis associated with a polymorphic Sp1 binding site in the collagen type I alpha 1 gene. Nat. Genet..

[53] Richard E (2010). Morphometric changes in the cortical microvascular network in Alzheimer’s disease. J. Alzheimer’s Dis. JAD.

[54] Miller VM (2019). The Kronos Early Estrogen Prevention Study (KEEPS): what have we learned?. Menopause.

[55] Wong IP, Zengin A, Herzog H, Baldock PA (2008). Central regulation of bone mass. Semin. Cell Dev. Biol..

[56] Hökfelt T (1998). Neuropeptide Y: some viewpoints on a multifaceted peptide in the normal and diseased nervous system. Brain Res. Brain Res. Rev..

[57] Lindefors N, Brené S, Herrera-Marschitz M, Persson H (1990). Regulation of neuropeptide Y gene expression in rat brain. Ann. N. Y. Acad. Sci..

[58] Rogers, L. J. & Andrew, R. *Comparative vertebrate lateralization*. (Cambridge University Press, 2002).

[59] Ocklenburg S, Güntürkün O (2012). Hemispheric asymmetries: the comparative view. Front. Psychol..

[60] Concha ML, Signore IA, Colombo A (2009). Mechanisms of directional asymmetry in the zebrafish epithalamus. Semin. Cell Dev. Biol..

[61] Hervé P-Y, Zago L, Petit L, Mazoyer B, Tzourio-Mazoyer NJ (2013). Revisiting human hemispheric specialization with neuroimaging. Trends Cogn. Sci..

[62] Gotts SJ (2013). Two distinct forms of functional lateralization in the human brain. Proc. Natl. Acad. Sci. USA.

[63] Guadalupe T (2015). Asymmetry within and around the human planum temporale is sexually dimorphic and influenced by genes involved in steroid hormone receptor activity. Cortex.

[64] Guadalupe T (2014). Measurement and genetics of human subcortical and hippocampal asymmetries in large datasets. Hum. Brain Mapp..

[65] Zhu D (2022). Total brain volumetric measures and schizophrenia risk: a two-sample mendelian randomization study. Front. Genet..

[66] van der Sluis S, Posthuma D, Nivard MG, Verhage M, Dolan CV (2013). Power in GWAS: lifting the curse of the clinical cut-off. Mol. Psychiatry.

[67] Elliott LT (2018). Genome-wide association studies of brain imaging phenotypes in UK Biobank. Nature.

[68] Bulik-Sullivan BK (2015). LD Score regression distinguishes confounding from polygenicity in genome-wide association studies. Nat. Genet..

[69] Xu J (2022). Assessing the Association between Important Dietary Habits and Osteoporosis: a genetic correlation and two-sample mendelian randomization study. Nutrients.

[70] Bulik-Sullivan BK (2015). LD Score regression distinguishes confounding from polygenicity in genome-wide association studies. Nat. Genet..

[71] Davies NM, Holmes MV, Davey Smith G (2018). Reading Mendelian randomisation studies: a guide, glossary, and checklist for clinicians. BMJ.

[72] Kamat MA (2019). PhenoScanner V2: an expanded tool for searching human genotype-phenotype associations. Bioinforma. (Oxf., Engl.).

[73] Pagoni P, Dimou NL, Murphy N, Stergiakouli E (2019). Using Mendelian randomisation to assess causality in observational studies. Evid. Based Ment. Health.

[74] Wang C (2022). Causal associations of obesity related anthropometric indicators and body compositions with knee and hip arthritis: a large-scale genetic correlation study. Front. Endocrinol..

[75] Bowden J, Davey Smith G, Burgess S (2015). Mendelian randomization with invalid instruments: effect estimation and bias detection through Egger regression. Int. J. Epidemiol..

[76] Hemani G, Tilling K, Davey Smith G (2017). Orienting the causal relationship between imprecisely measured traits using GWAS summary data. PLoS Genet..

[77] Armstrong RA (2014). When to use the Bonferroni correction. Ophthalmic Physiol. Opt..

[78] Benjamini Y, Hochberg Y (1995). Controlling the false discovery rate: a practical and powerful approach to multiple testing. J. R. Stat. Soc.: Ser. B Methodol.

