# Supplementary material for: Causal associations of brain structure with bone mineral density: a large-scale genetic correlation study
Source: Bone Res. 2023 Jul 20;11:37. doi: 10.1038/s41413-023-00270-z (PMC10359275; doi:10.1038/s41413-023-00270-z)
Supplement: Supplementary file 1 — Supplementary Table 7. The confounding factors screened by PhenoScanner V2 [file 41413_2023_270_MOESM1_ESM.pdf]

Supplemental table 7. The confounding factors screened by PhenoScanner V2

rs_id	hg19_coordinates	hg38_coordinates	a1	a2	trait
rs35518360	chr4:103146890	chr4:102225733	A	T	Alcohol intake frequency
rs1944461	chr11:121917163	chr11:122046455	C	T	Arm fat mass left
rs35518360	chr4:103146890	chr4:102225733	A	T	Arm fat mass left
rs8115257	chr20:25207931	chr20:25227295	A	G	Arm fat mass left
rs1944461	chr11:121917163	chr11:122046455	C	T	Arm fat mass right
rs35518360	chr4:103146890	chr4:102225733	A	T	Arm fat mass right
rs8115257	chr20:25207931	chr20:25227295	A	G	Arm fat mass right
rs35518360	chr4:103146890	chr4:102225733	A	T	Arm fat percentage left
rs8115257	chr20:25207931	chr20:25227295	A	G	Arm fat percentage left
rs35518360	chr4:103146890	chr4:102225733	A	T	Arm fat percentage right
rs8115257	chr20:25207931	chr20:25227295	A	G	Arm fat percentage right
rs12940346	chr17:68075324	chr17:70079183	C	T	Arm fat-free mass left
rs12940346	chr17:68075324	chr17:70079183	C	T	Arm fat-free mass right
rs12940346	chr17:68075324	chr17:70079183	C	T	Arm predicted mass left
rs12940346	chr17:68075324	chr17:70079183	C	T	Arm predicted mass right
rs11049487	chr12:28407090	chr12:28254157	A	T	Basal metabolic rate
rs12940346	chr17:68075324	chr17:70079183	C	T	Basal metabolic rate
rs35518360	chr4:103146890	chr4:102225733	A	T	Body fat percentage
rs8115257	chr20:25207931	chr20:25227295	A	G	Body fat percentage
rs35518360	chr4:103146890	chr4:102225733	A	T	Body mass index
rs8115257	chr20:25207931	chr20:25227295	A	G	Body mass index
rs35518360	chr4:103146890	chr4:102225733	A	T	Comparative body size at age 10
rs35518360	chr4:103146890	chr4:102225733	A	T	Comparative height size at age 10
rs11049487	chr12:28407090	chr12:28254157	A	T	Comparative height size at age 10
rs12940346	chr17:68075324	chr17:70079183	C	T	Comparative height size at age 10
rs35518360	chr4:103146890	chr4:102225733	A	T	Height
rs11049487	chr12:28407090	chr12:28254157	A	T	Height
rs11049487	chr12:28407090	chr12:28254157	A	T	Height
rs12940346	chr17:68075324	chr17:70079183	C	T	Height
rs1944461	chr11:121917163	chr11:122046455	C	T	Hip circumference
rs35518360	chr4:103146890	chr4:102225733	A	T	Hip circumference
rs11049487	chr12:28407090	chr12:28254157	A	T	Hip circumference
rs3779381	chr7:120966790	chr7:121326736	G	A	Lean body mass
rs35518360	chr4:103146890	chr4:102225733	A	T	Leg fat mass left
rs8115257	chr20:25207931	chr20:25227295	A	G	Leg fat mass left
rs35518360	chr4:103146890	chr4:102225733	A	T	Leg fat mass right
rs8115257	chr20:25207931	chr20:25227295	A	G	Leg fat mass right
rs35518360	chr4:103146890	chr4:102225733	A	T	Leg fat percentage left
rs8115257	chr20:25207931	chr20:25227295	A	G	Leg fat percentage left
rs35518360	chr4:103146890	chr4:102225733	A	T	Leg fat percentage right
rs8115257	chr20:25207931	chr20:25227295	A	G	Leg fat percentage right
rs11049487	chr12:28407090	chr12:28254157	A	T	Leg fat-free mass left

rs12940346	chr17:68075324	chr17:70079183	C	T	Leg fat-free mass left
rs12575721	chr11:46823681	chr11:46802131	C	T	Leg fat-free mass right
rs11049487	chr12:28407090	chr12:28254157	A	T	Leg fat-free mass right
rs12940346	chr17:68075324	chr17:70079183	C	T	Leg fat-free mass right
rs11049487	chr12:28407090	chr12:28254157	A	T	Leg predicted mass left
rs12940346	chr17:68075324	chr17:70079183	C	T	Leg predicted mass left
rs3779381	chr7:120966790	chr7:121326736	G	A	Leg predicted mass left
rs12575721	chr11:46823681	chr11:46802131	C	T	Leg predicted mass right
rs11049487	chr12:28407090	chr12:28254157	A	T	Leg predicted mass right
rs12940346	chr17:68075324	chr17:70079183	C	T	Leg predicted mass right
rs1944461	chr11:121917163	chr11:122046455	C	T	Trunk fat mass
rs35518360	chr4:103146890	chr4:102225733	A	T	Trunk fat mass
rs11049487	chr12:28407090	chr12:28254157	A	T	Trunk fat mass
rs8115257	chr20:25207931	chr20:25227295	A	G	Trunk fat mass
rs35518360	chr4:103146890	chr4:102225733	A	T	Trunk fat percentage
rs8115257	chr20:25207931	chr20:25227295	A	G	Trunk fat percentage
rs12940346	chr17:68075324	chr17:70079183	C	T	Trunk fat-free mass
rs12940346	chr17:68075324	chr17:70079183	C	T	Trunk predicted mass
rs35518360	chr4:103146890	chr4:102225733	A	T	Waist circumference
rs8115257	chr20:25207931	chr20:25227295	A	G	Waist circumference
rs11049487	chr12:28407090	chr12:28254157	A	T	Waist circumference adjusted for BMI
rs11049487	chr12:28407090	chr12:28254157	A	T	Waist circumference adjusted for physical activity
rs11049487	chr12:28407090	chr12:28254157	A	T	Waist circumference adjusted for physical activity
rs11049487	chr12:28407090	chr12:28254157	A	T	Waist circumference adjusted for smoking
rs11049487	chr12:28407090	chr12:28254157	A	T	Waist circumference adjusted for smoking
rs11049487	chr12:28407090	chr12:28254157	A	T	Waist circumference in non-smokers
rs11049487	chr12:28407090	chr12:28254157	A	T	Waist circumference in non-smokers
rs11049487	chr12:28407090	chr12:28254157	A	T	Waist circumference in physically active indivdiuals
rs11049487	chr12:28407090	chr12:28254157	A	T	Waist circumference in physically active indivdiuals
rs35518360	chr4:103146890	chr4:102225733	A	T	Weight
rs11049487	chr12:28407090	chr12:28254157	A	T	Weight
rs8115257	chr20:25207931	chr20:25227295	A	G	Weight
rs35518360	chr4:103146890	chr4:102225733	A	T	Whole body fat mass
rs8115257	chr20:25207931	chr20:25227295	A	G	Whole body fat mass
rs12940346	chr17:68075324	chr17:70079183	C	T	Whole body fat-free mass
