# Supplementary material for: Causal associations of brain structure with bone mineral density: a large-scale genetic correlation study
Source: Bone Res. 2023 Jul 20;11:37. doi: 10.1038/s41413-023-00270-z (PMC10359275; doi:10.1038/s41413-023-00270-z)
Supplement: Supplementary file 2 — Supplementary Figure 1-36 [file 41413_2023_270_MOESM2_ESM.pdf]

Supplementary Figure List

Index	UKBID	IDP.short.name	Outcome.name	Plot.type
1	25005	IDP_T1_SIENAX_grey_normalised_volume	Femoral neck	scatter
2	25005	IDP_T1_SIENAX_grey_normalised_volume	Femoral neck	funnel
3	25009	IDP_T1_SIENAX_brain−normalised_volume	Femoral neck	scatter
4	25009	IDP_T1_SIENAX_brain−normalised_volume	Femoral neck	funnel
5	26521	aseg_global_volume_EstimatedTotalIntraCranial	Femoral neck	scatter
6	26521	aseg_global_volume_EstimatedTotalIntraCranial	Femoral neck	funnel
7	26536	aseg_global_volume−ratio_BrainSegVol−to−eTIV	Total body	scatter
8	26536	aseg_global_volume−ratio_BrainSegVol−to−eTIV	Total body	funnel
9	26536	aseg_global_volume−ratio_BrainSegVol−to−eTIV	Heel	scatter
10	26536	aseg_global_volume−ratio_BrainSegVol−to−eTIV	Heel	funnel
11	26536	aseg_global_volume−ratio_BrainSegVol−to−eTIV	Lumbar spine	scatter
12	26536	aseg_global_volume−ratio_BrainSegVol−to−eTIV	Lumbar spine	funnel
13	26536	aseg_global_volume−ratio_BrainSegVol−to−eTIV	Forearm	scatter
14	26536	aseg_global_volume−ratio_BrainSegVol−to−eTIV	Forearm	funnel
15	26536	aseg_global_volume−ratio_BrainSegVol−to−eTIV	Femoral neck	scatter
16	26536	aseg_global_volume−ratio_BrainSegVol−to−eTIV	Femoral neck	funnel
17	26606	AmygNuclei_lh_volume_Cortical−nucleus	Heel	scatter
18	26606	AmygNuclei_lh_volume_Cortical−nucleus	Heel	funnel
19	27307	aparc−DKTatlas_rh_volume_lateralorbitofrontal	Heel	scatter
20	27307	aparc−DKTatlas_rh_volume_lateralorbitofrontal	Heel	funnel
21	27375	aparc−a2009s_lh_area_S−circular−insula−ant	Femoral neck	scatter
22	27375	aparc−a2009s_lh_area_S−circular−insula−ant	Femoral neck	funnel
23	27392	aparc−a2009s_lh_area_S−orbital−H−Shaped	Heel	scatter
24	27392	aparc−a2009s_lh_area_S−orbital−H−Shaped	Heel	funnel
25	26763	aparc−Desikan_lh_thickness_inferiortemporal	Lumbar spine	scatter
26	26763	aparc−Desikan_lh_thickness_inferiortemporal	Lumbar spine	funnel
27	26767	aparc−Desikan_lh_thickness_lingual	Heel	scatter
28	26767	aparc−Desikan_lh_thickness_lingual	Heel	funnel
29	26864	aparc−Desikan_rh_thickness_inferiortemporal	Total body	scatter
30	26864	aparc−Desikan_rh_thickness_inferiortemporal	Total body	funnel
31	26865	aparc−Desikan_rh_thickness_isthmuscingulate	Total body	scatter
32	26865	aparc−Desikan_rh_thickness_isthmuscingulate	Total body	funnel
33	27439	aparc−a2009s_lh_thickness_G−temporal−inf	Lumbar spine	scatter
34	27439	aparc−a2009s_lh_thickness_G−temporal−inf	Lumbar spine	funnel
35	27695	aparc−a2009s_rh_thickness_S−subparietal	Heel	scatter
36	27695	aparc−a2009s_rh_thickness_S−subparietal	Heel	funnel

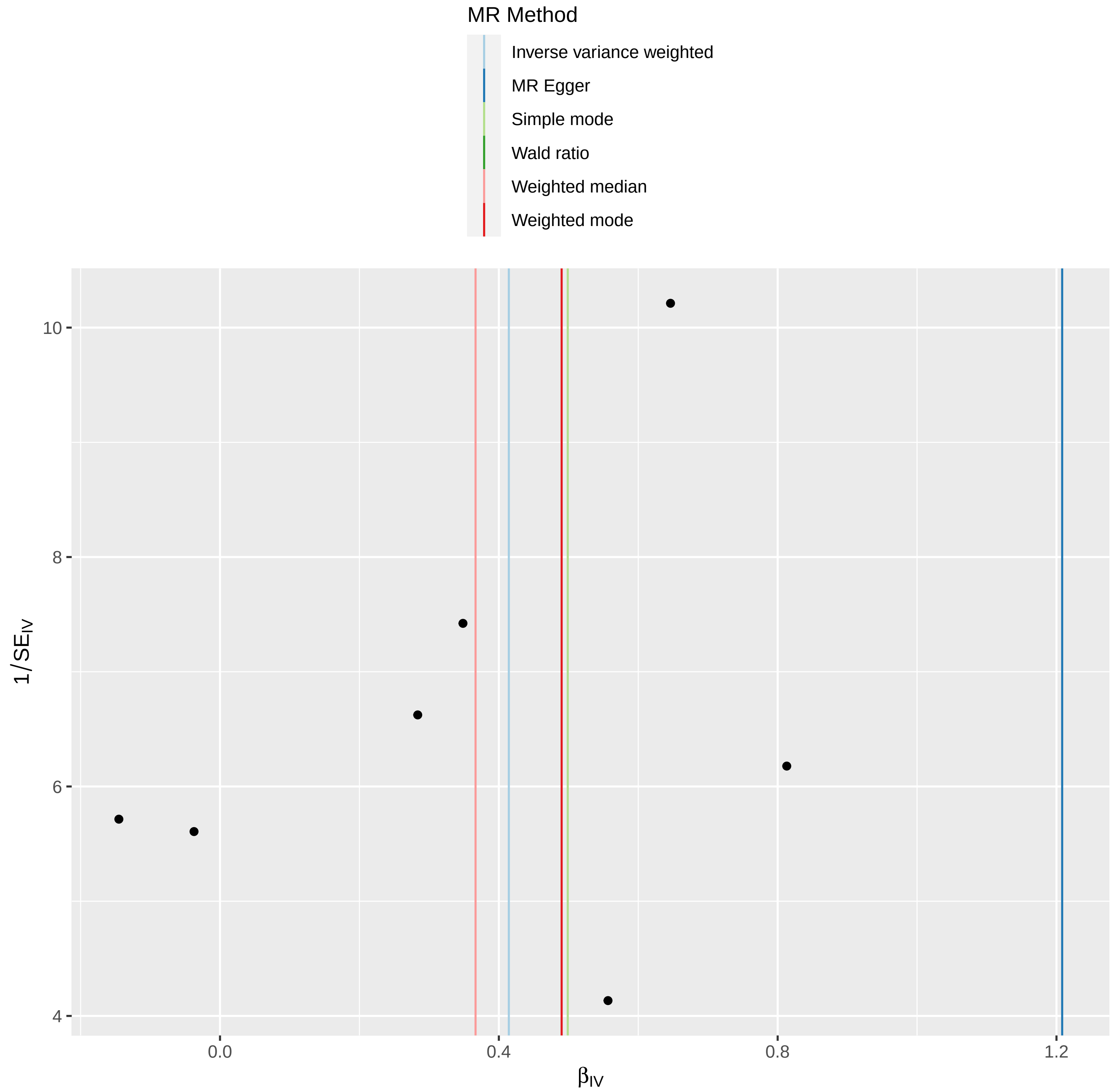
MR Method
Inverse variance weighted
MR Egger
Simple mode
Wald ratio
Weighted median
Weighted mode
10
8
6
4
1/SE$_{IV}$
0.0
0.4
0.8
1.2
β_{IV}

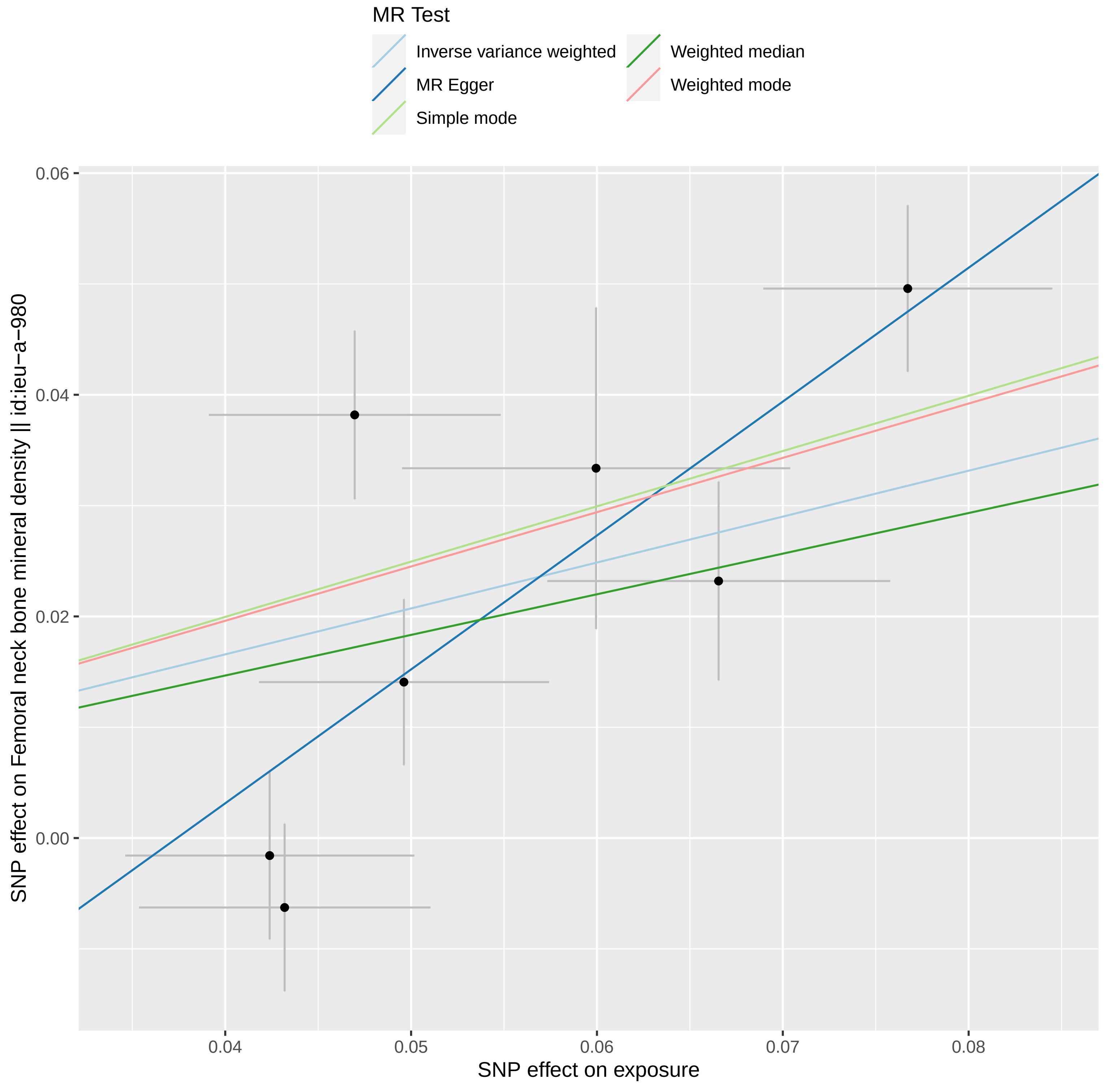

MR Test
Inverse variance weighted
MR Egger
Simple mode
Weighted median
Weighted mode
0.06
0.04
0.02
0.00
SNP effect on Femoral neck bone mineral density || id:ieu−a−980
0.04
0.05
0.06
0.07
0.08
SNP effect on exposure

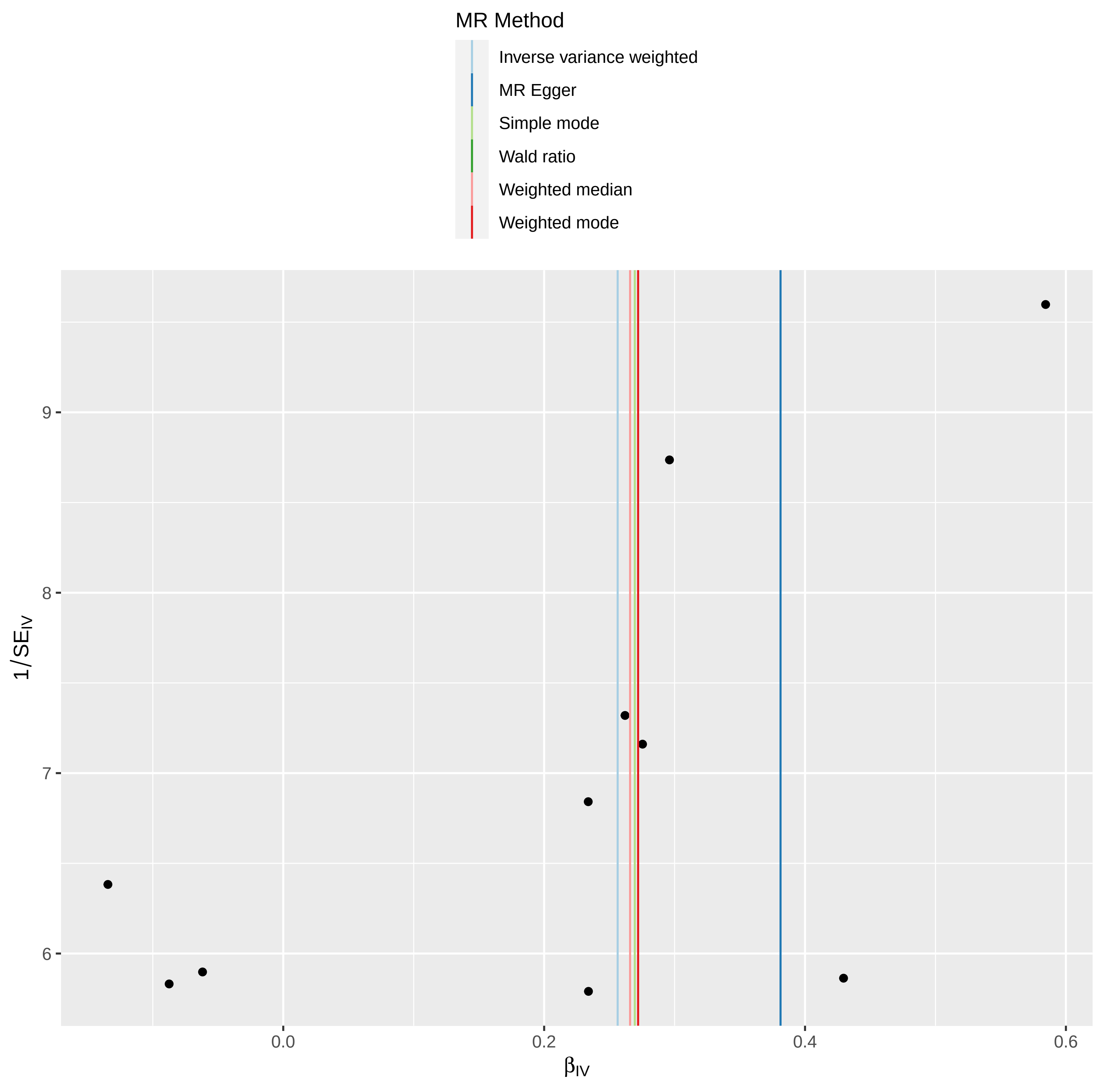

MR Method
Inverse variance weighted
MR Egger
Simple mode
Wald ratio
Weighted median
Weighted mode
9
8
7
6
1/SE_IV
0.0
0.2
0.4
0.6
β_IV

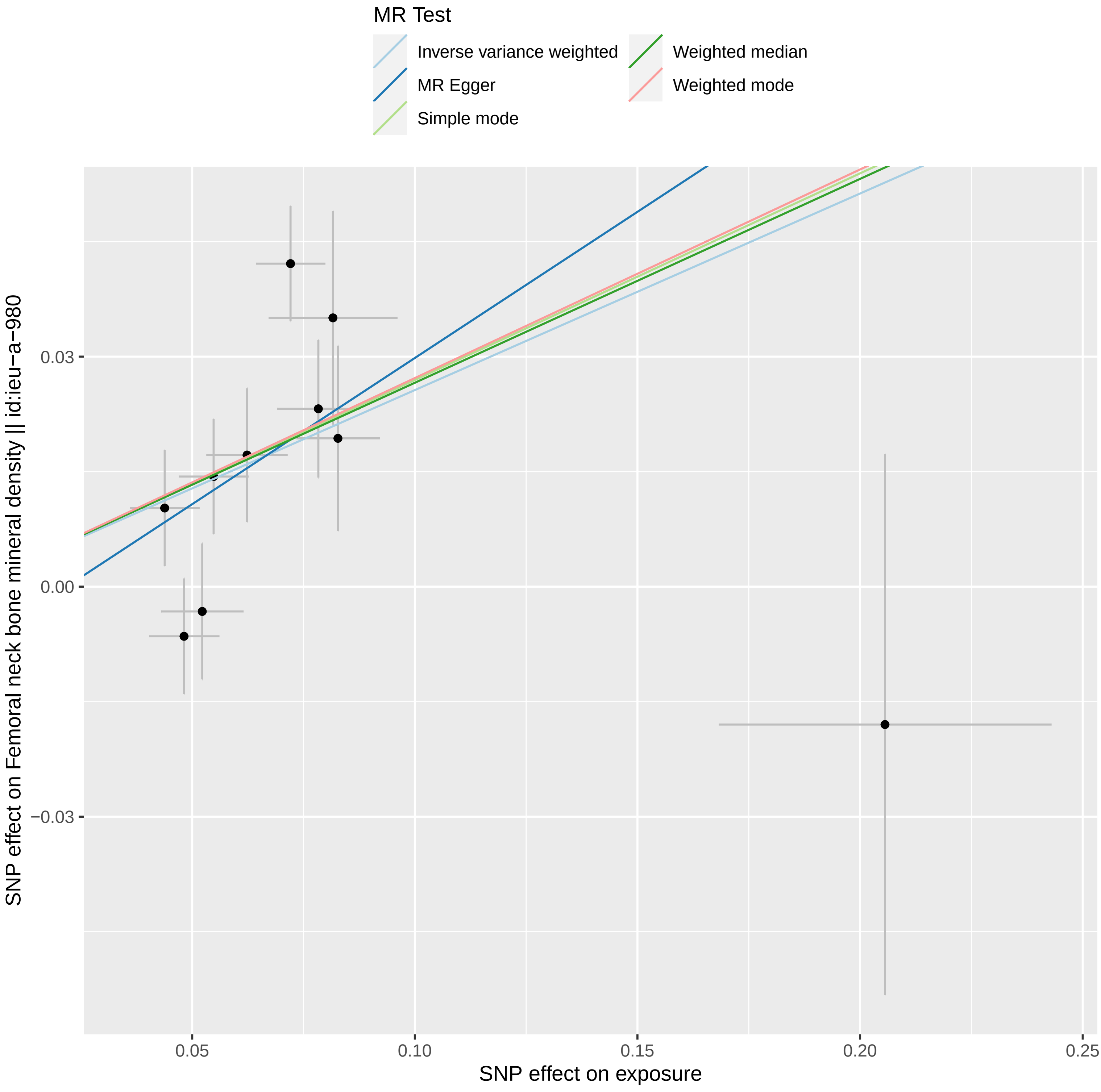
MR Test
Inverse variance weighted
MR Egger
Simple mode
Weighted median
Weighted mode
SNP effect on Femoral neck bone mineral density || id:ieu−a−980
0.03
0.00
−0.03
0.05
0.10
0.15
0.20
0.25
SNP effect on exposure

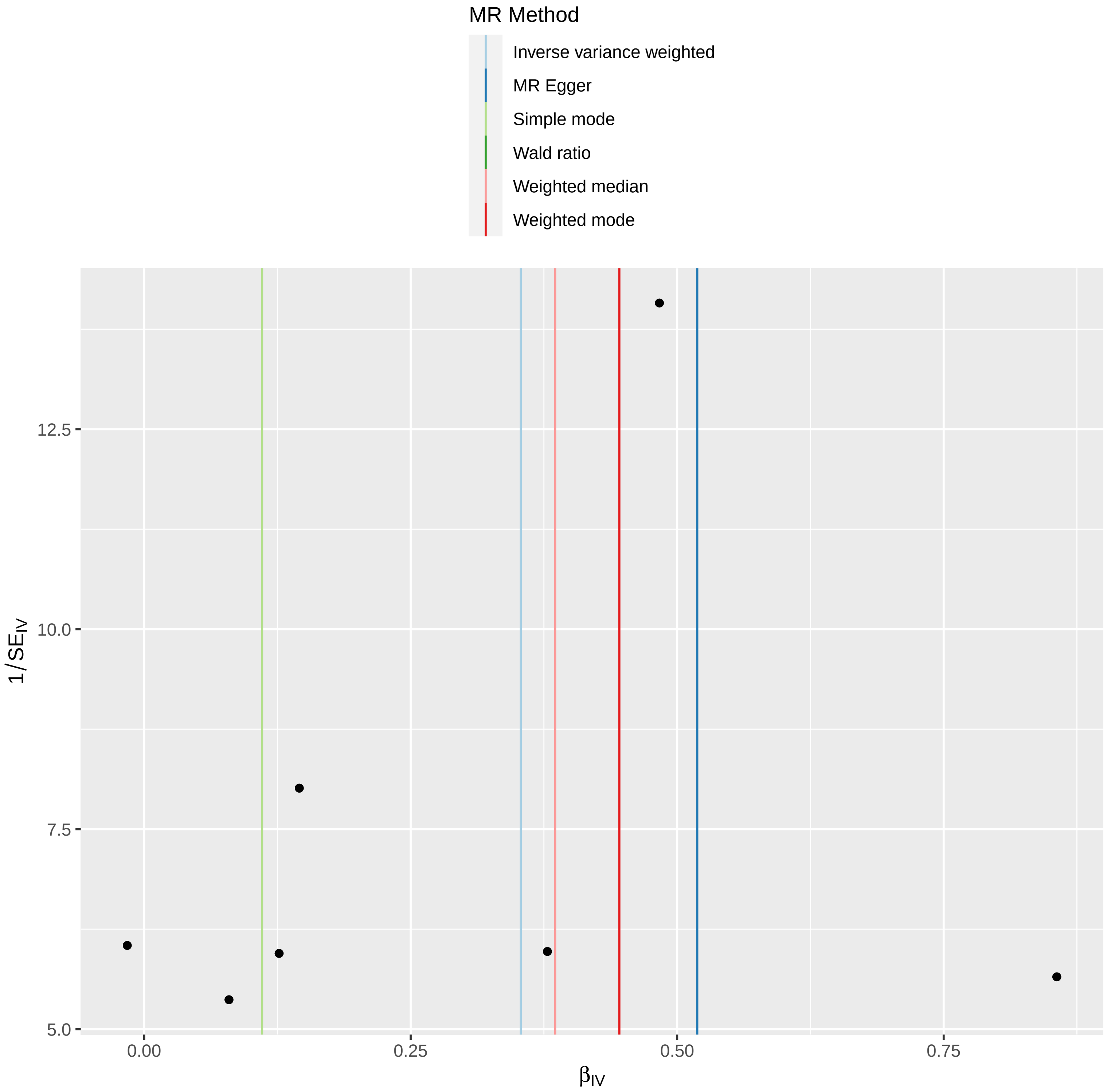

MR Method
Inverse variance weighted
MR Egger
Simple mode
Wald ratio
Weighted median
Weighted mode
12.5
10.0
7.5
5.0
1/SE_IV
0.00
0.25
0.50
0.75
β_IV

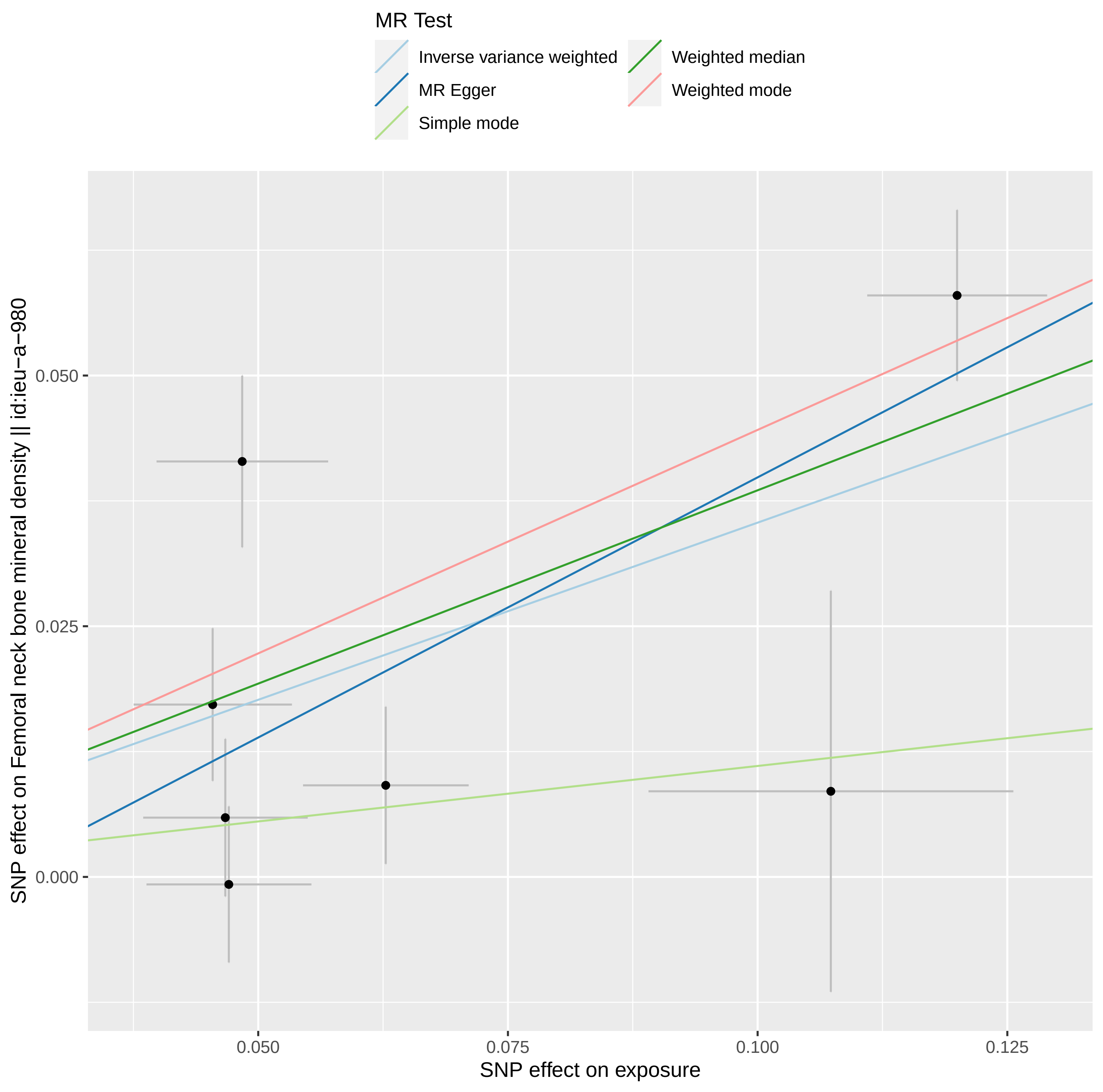
MR Test
Inverse variance weighted
MR Egger
Simple mode
Weighted median
Weighted mode
SNP effect on Femoral neck bone mineral density || id:ieu−a−980
0.050
0.025
0.000
0.050
0.075
0.100
0.125
SNP effect on exposure

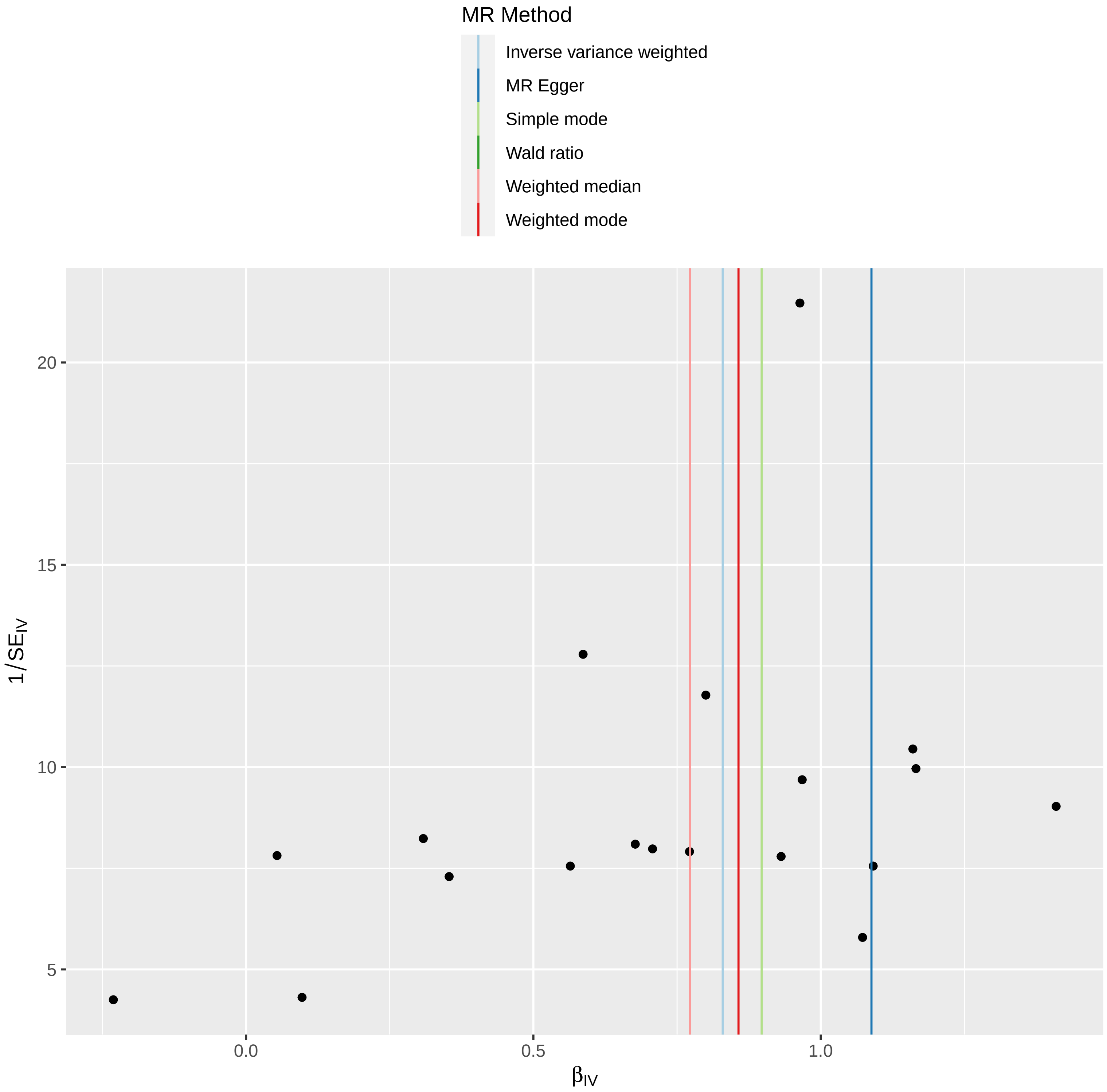

MR Method
Inverse variance weighted
MR Egger
Simple mode
Wald ratio
Weighted median
Weighted mode
20
15
10
5
0.0
0.5
1.0
$1/SE_{IV}$
β_{IV}

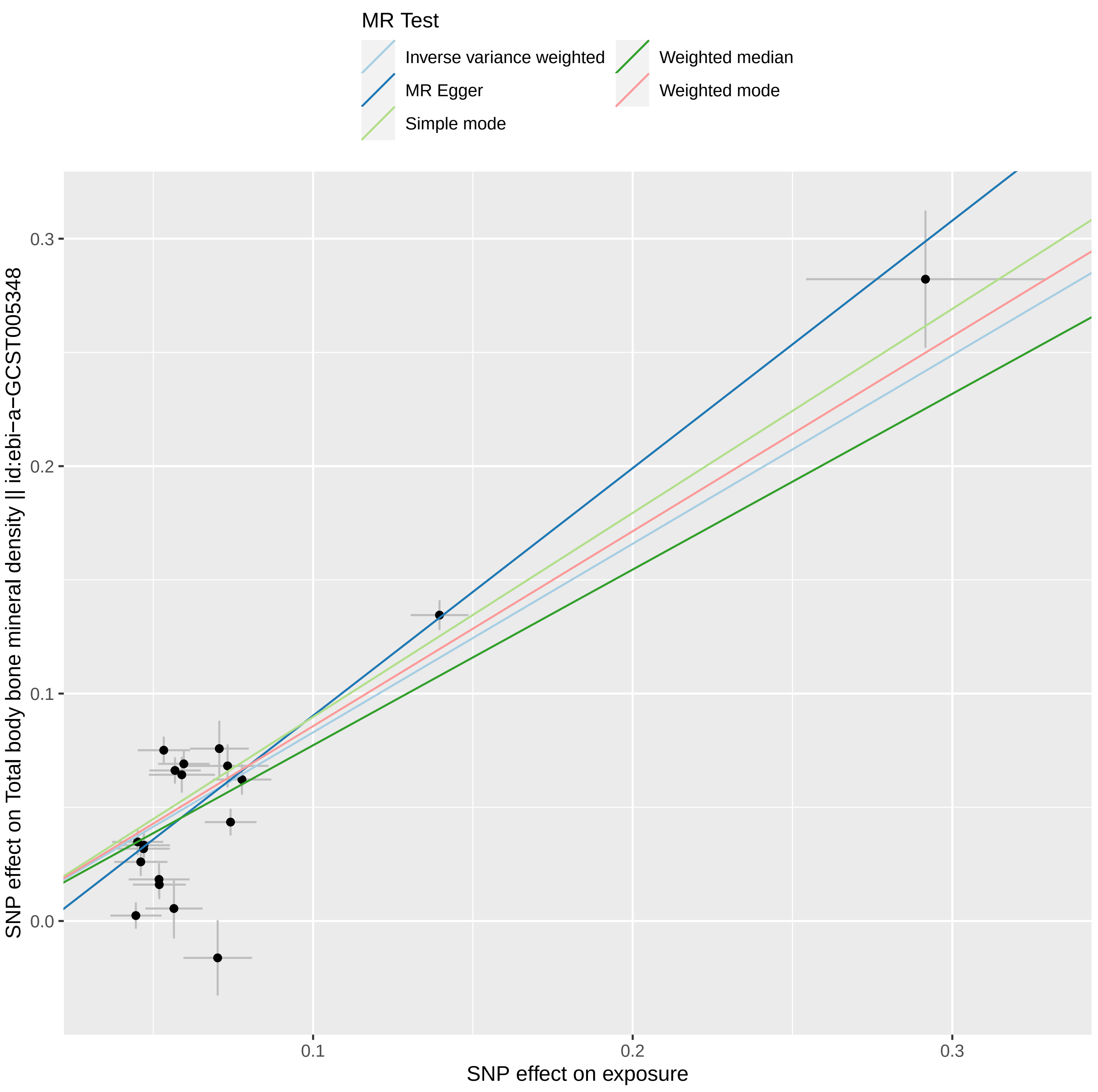
MR Test
Inverse variance weighted
MR Egger
Simple mode
Weighted median
Weighted mode
SNP effect on Total body bone mineral density || id:ebi−a−GCST005348
SNP effect on exposure
0.0
0.1
0.2
0.3
0.1
0.2
0.3

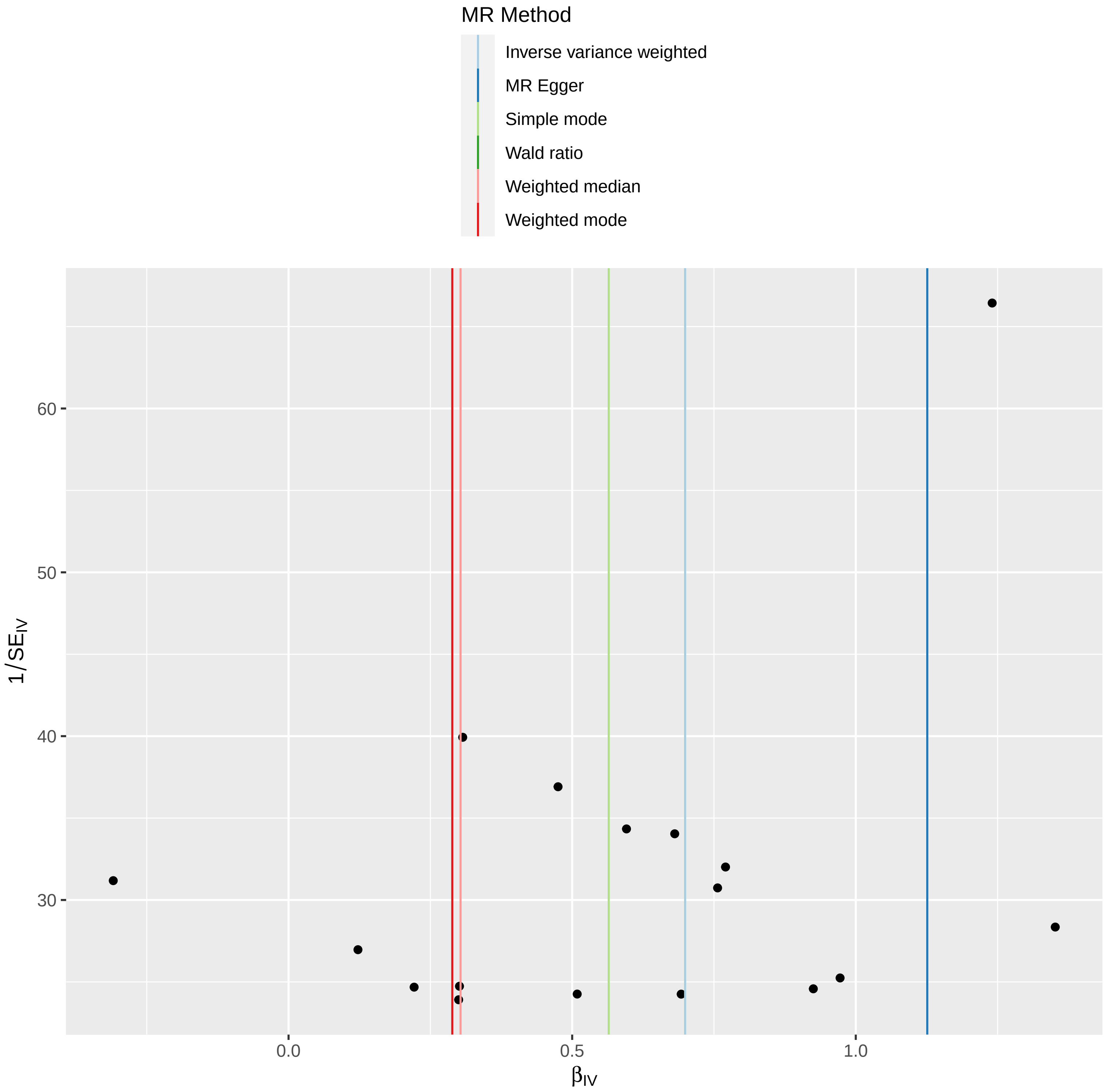
MR Method
Inverse variance weighted
MR Egger
Simple mode
Wald ratio
Weighted median
Weighted mode
60
50
40
30
1/SE_IV
0.0
0.5
1.0
β_IV

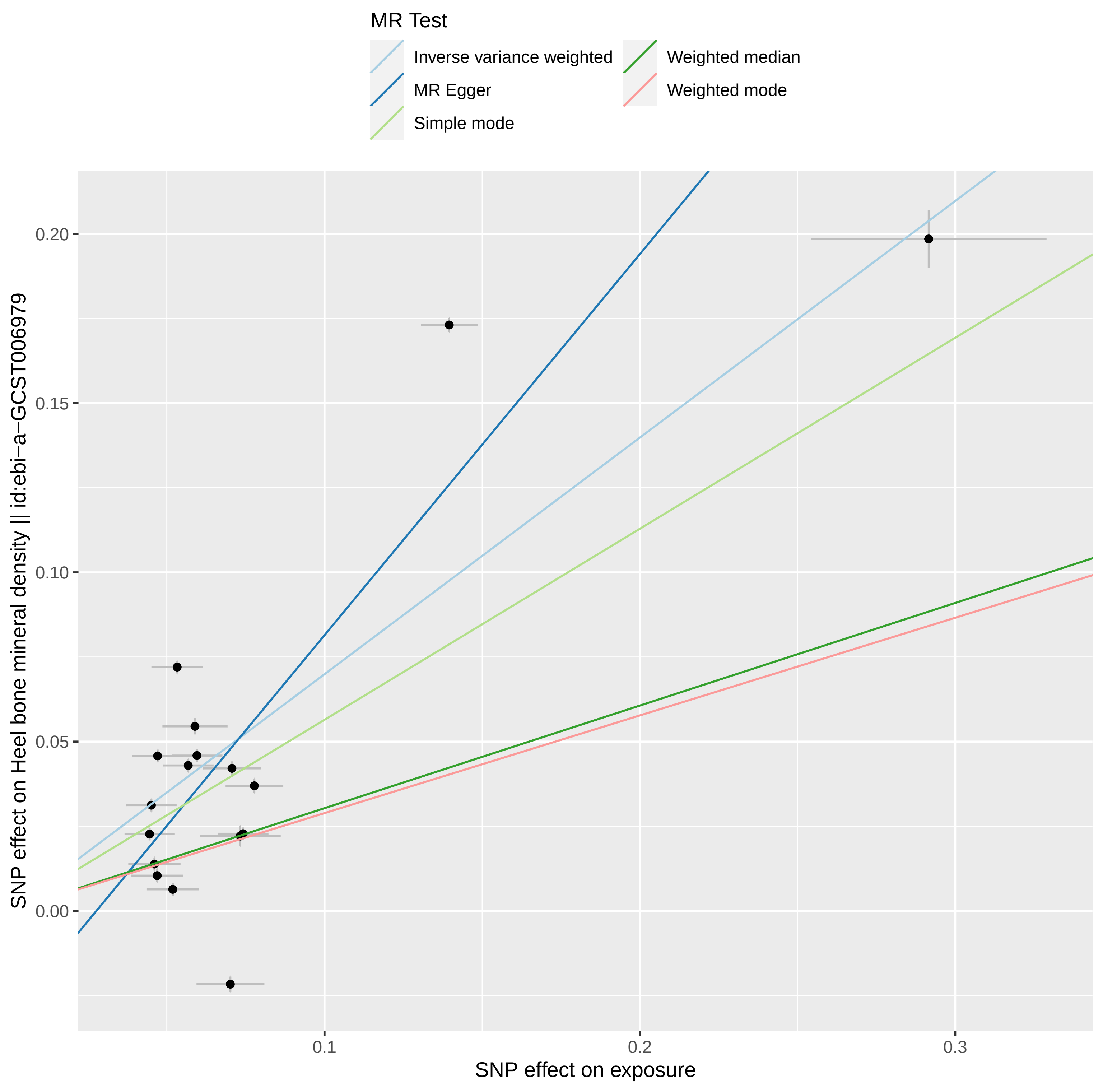
MR Test
Inverse variance weighted
MR Egger
Simple mode
Weighted median
Weighted mode
0.20
0.15
0.10
0.05
0.00
0.1
0.2
0.3
SNP effect on exposure
SNP effect on Heel bone mineral density || id:ebi−a−GCST006979

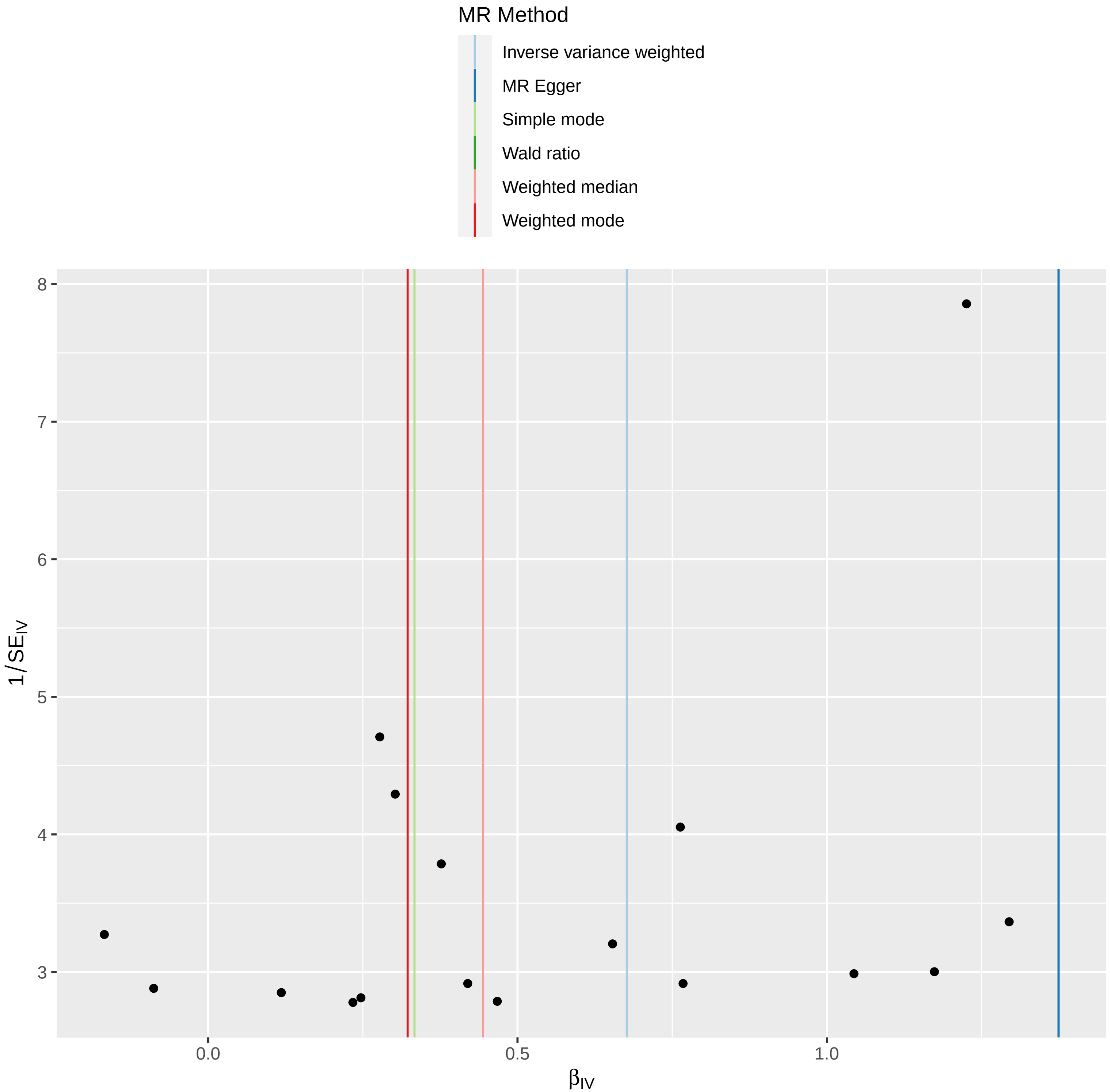
MR Method
Inverse variance weighted
MR Egger
Simple mode
Wald ratio
Weighted median
Weighted mode
8
7
6
5
4
3
1/SE_IV
0.0
0.5
1.0
β_IV

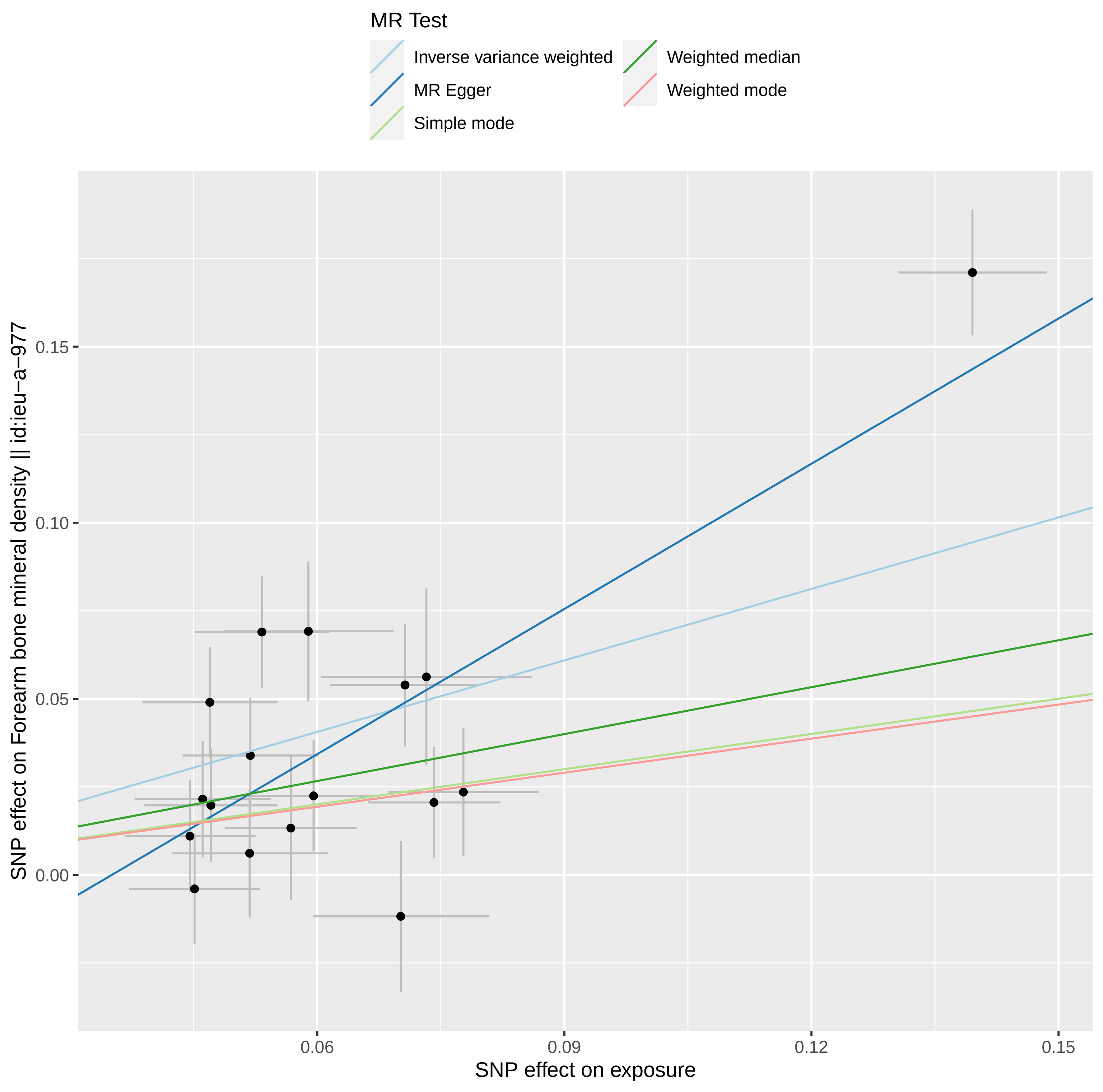
MR Test
Inverse variance weighted
MR Egger
Simple mode
Weighted median
Weighted mode
SNP effect on Forearm bone mineral density || id:ieu-a-977
0.15
0.10
0.05
0.00
0.06
0.09
0.12
0.15
SNP effect on exposure

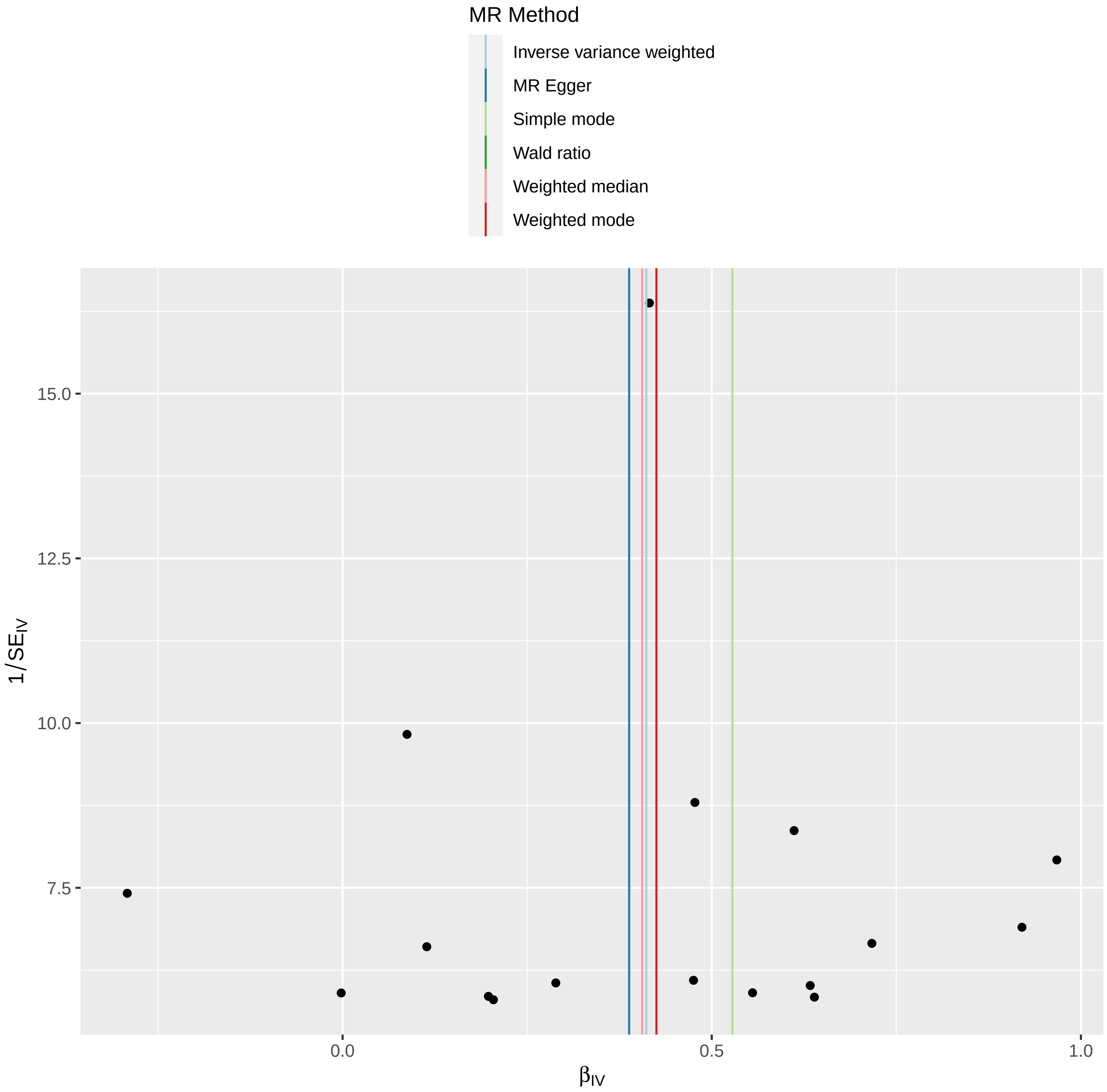

MR Method
Inverse variance weighted
MR Egger
Simple mode
Wald ratio
Weighted median
Weighted mode
15.0
12.5
10.0
7.5
$1/SE_{IV}$
0.0
0.5
1.0
β_{IV}

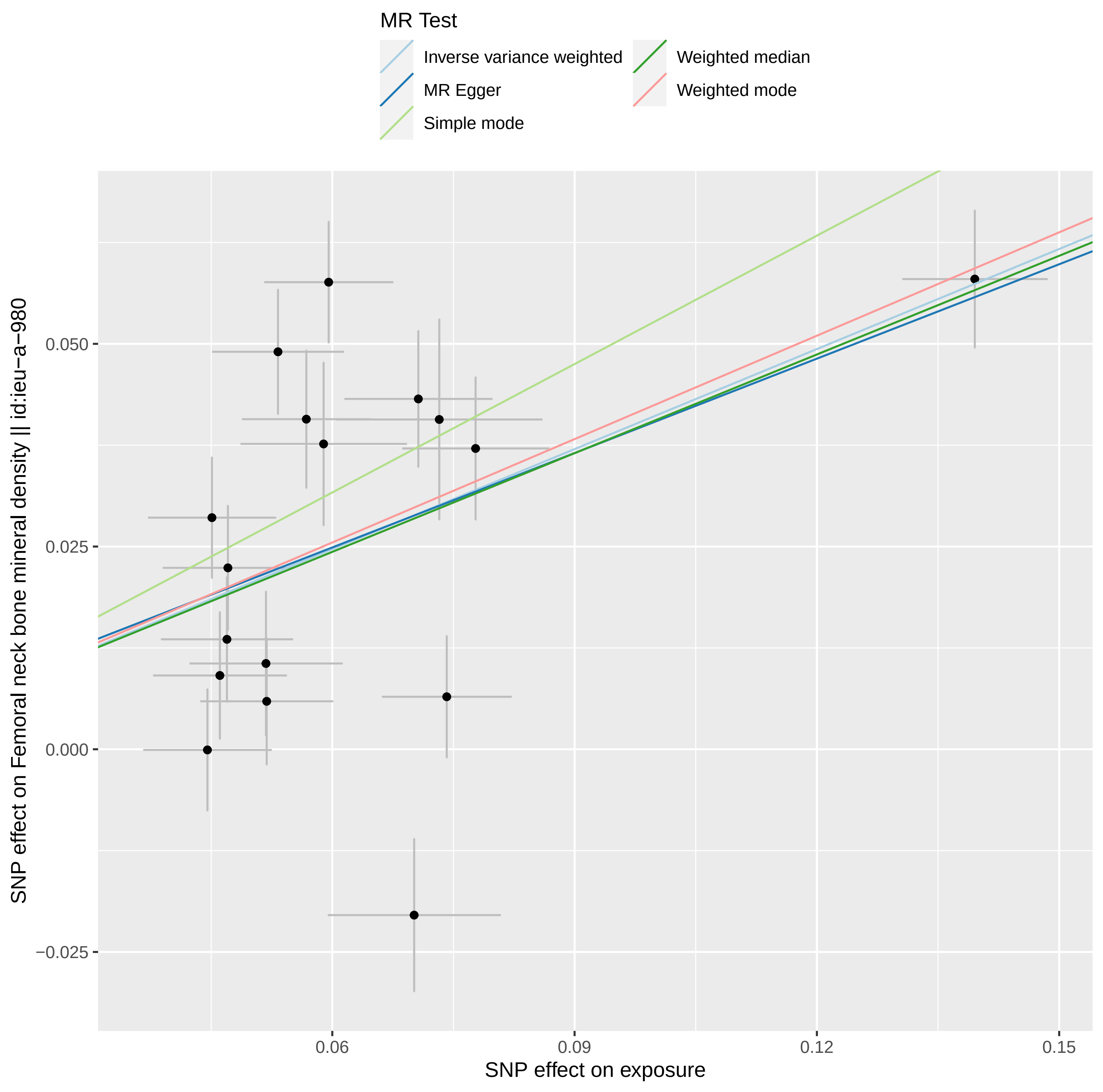

MR Test
Inverse variance weighted
MR Egger
Simple mode
Weighted median
Weighted mode
0.050
0.025
0.000
−0.025
0.06
0.09
0.12
0.15
SNP effect on exposure
SNP effect on Femoral neck bone mineral density || id:ieu−a−980

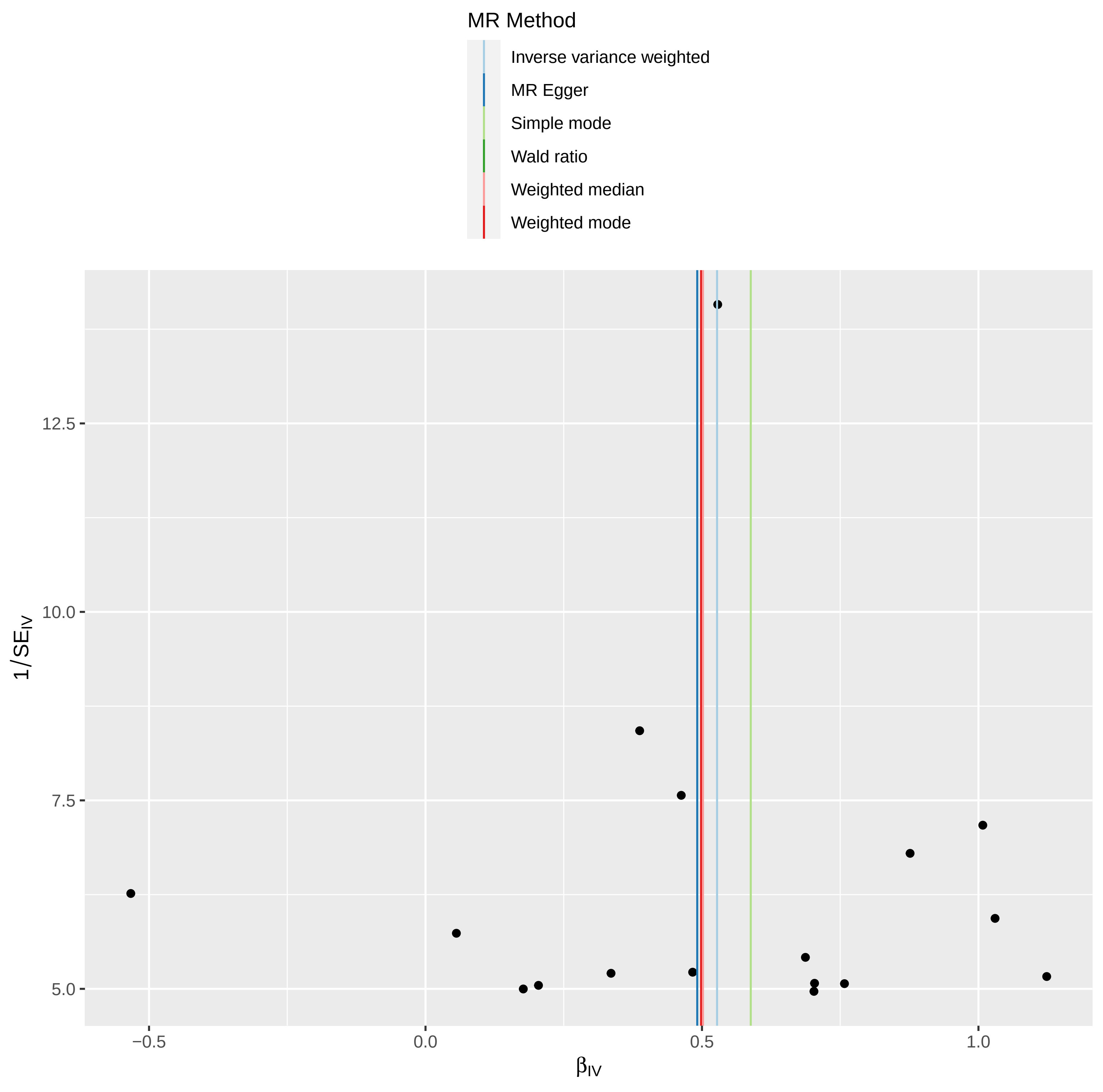
MR Method
Inverse variance weighted
MR Egger
Simple mode
Wald ratio
Weighted median
Weighted mode
12.5
10.0
7.5
5.0
1/SE_IV
−0.5
0.0
0.5
1.0
β_IV

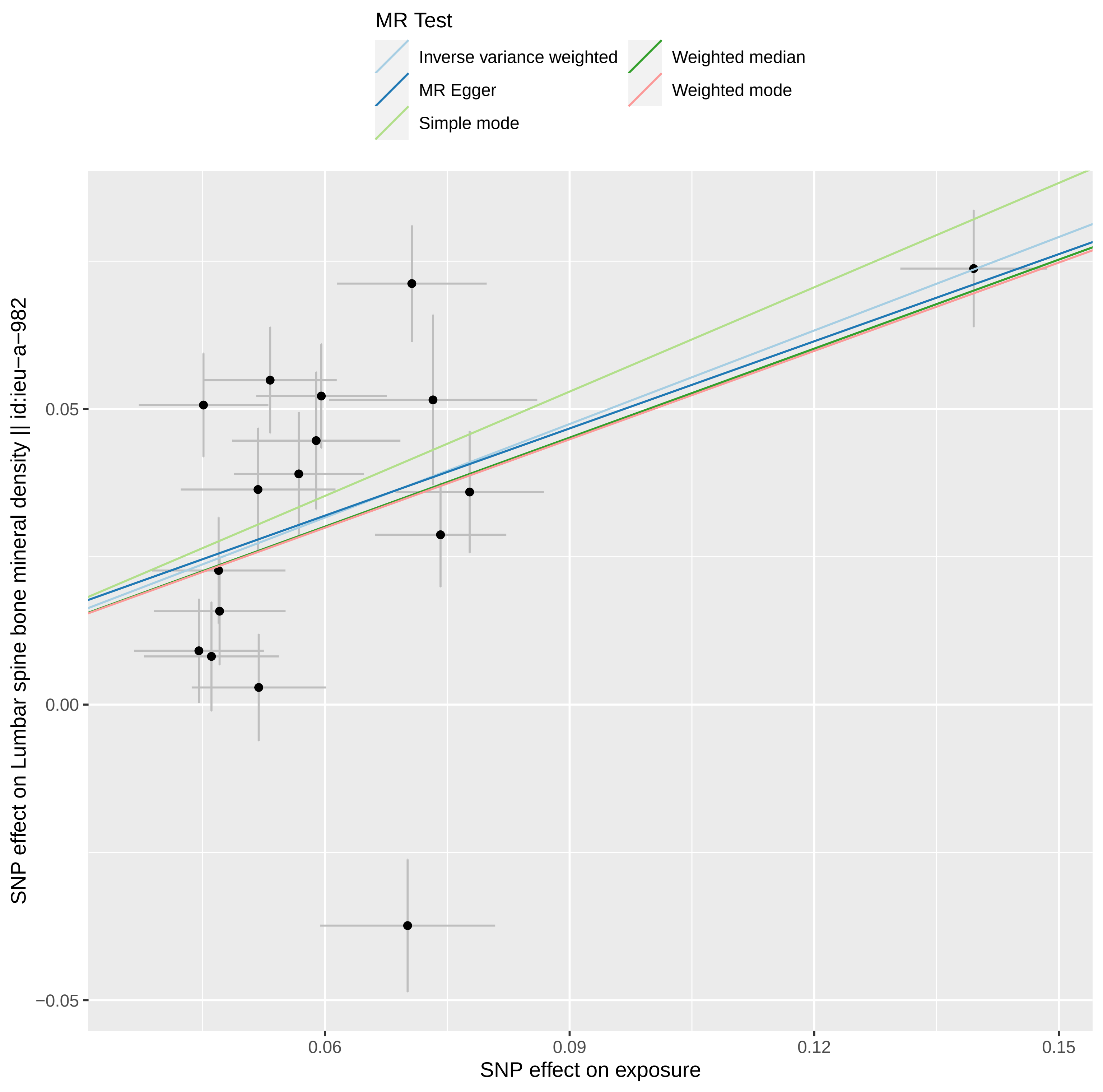
MR Test
Inverse variance weighted
MR Egger
Simple mode
Weighted median
Weighted mode
SNP effect on Lumbar spine bone mineral density || id:ieu-a-982
0.05
0.00
−0.05
0.06
0.09
0.12
0.15
SNP effect on exposure

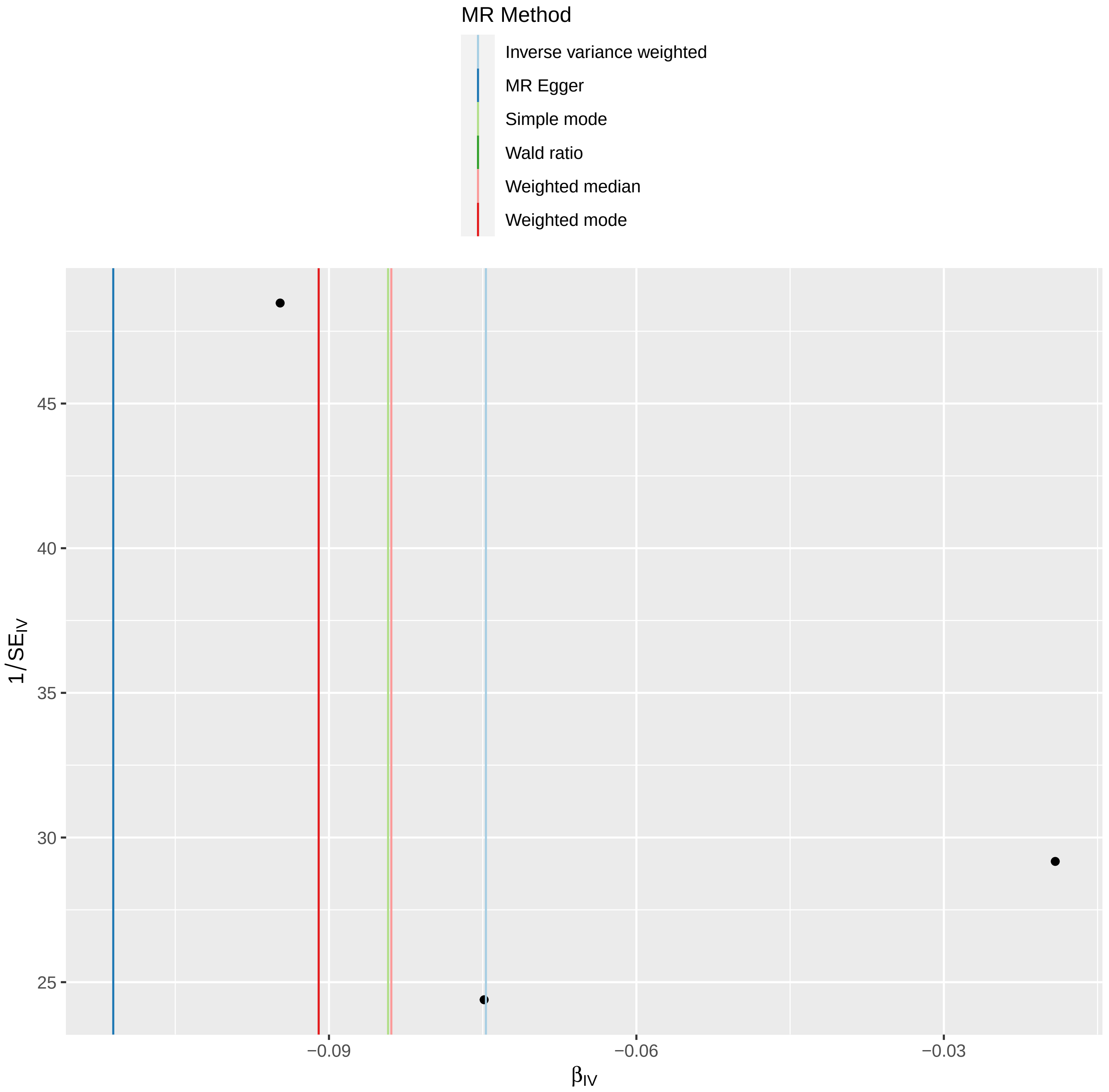
MR Method
Inverse variance weighted
MR Egger
Simple mode
Wald ratio
Weighted median
Weighted mode
45
40
35
30
25
1/SE$_{IV}$
−0.09
−0.06
−0.03
β$_{IV}$

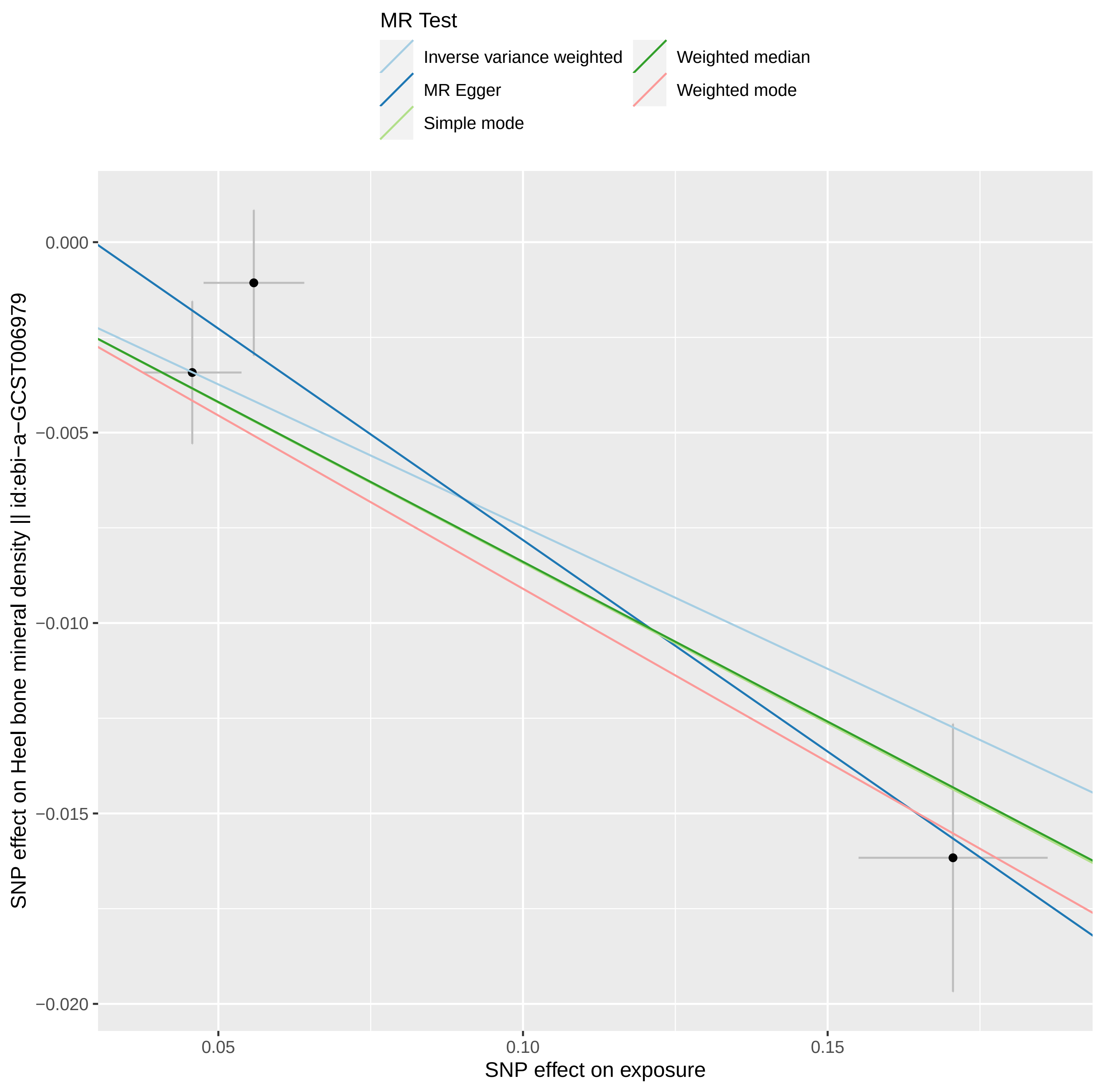

MR Test
Inverse variance weighted
MR Egger
Simple mode
Weighted median
Weighted mode
0.000
−0.005
−0.010
−0.015
−0.020
0.05
0.10
0.15
SNP effect on exposure
SNP effect on Heel bone mineral density || id:ebi−a−GCST006979

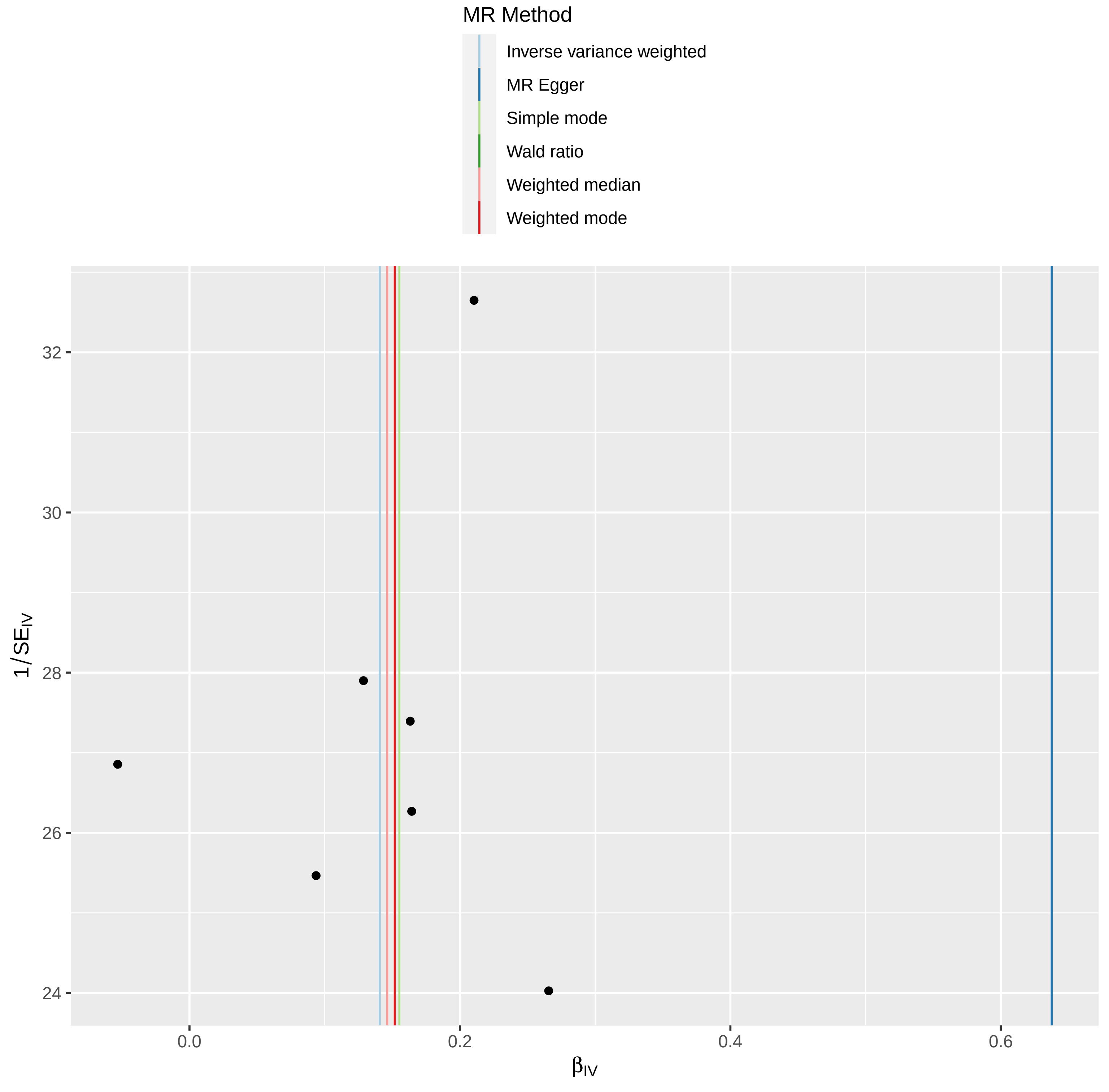
MR Method
Inverse variance weighted
MR Egger
Simple mode
Wald ratio
Weighted median
Weighted mode
1/SE_IV
32
30
28
26
24
0.0
0.2
0.4
0.6
β_IV

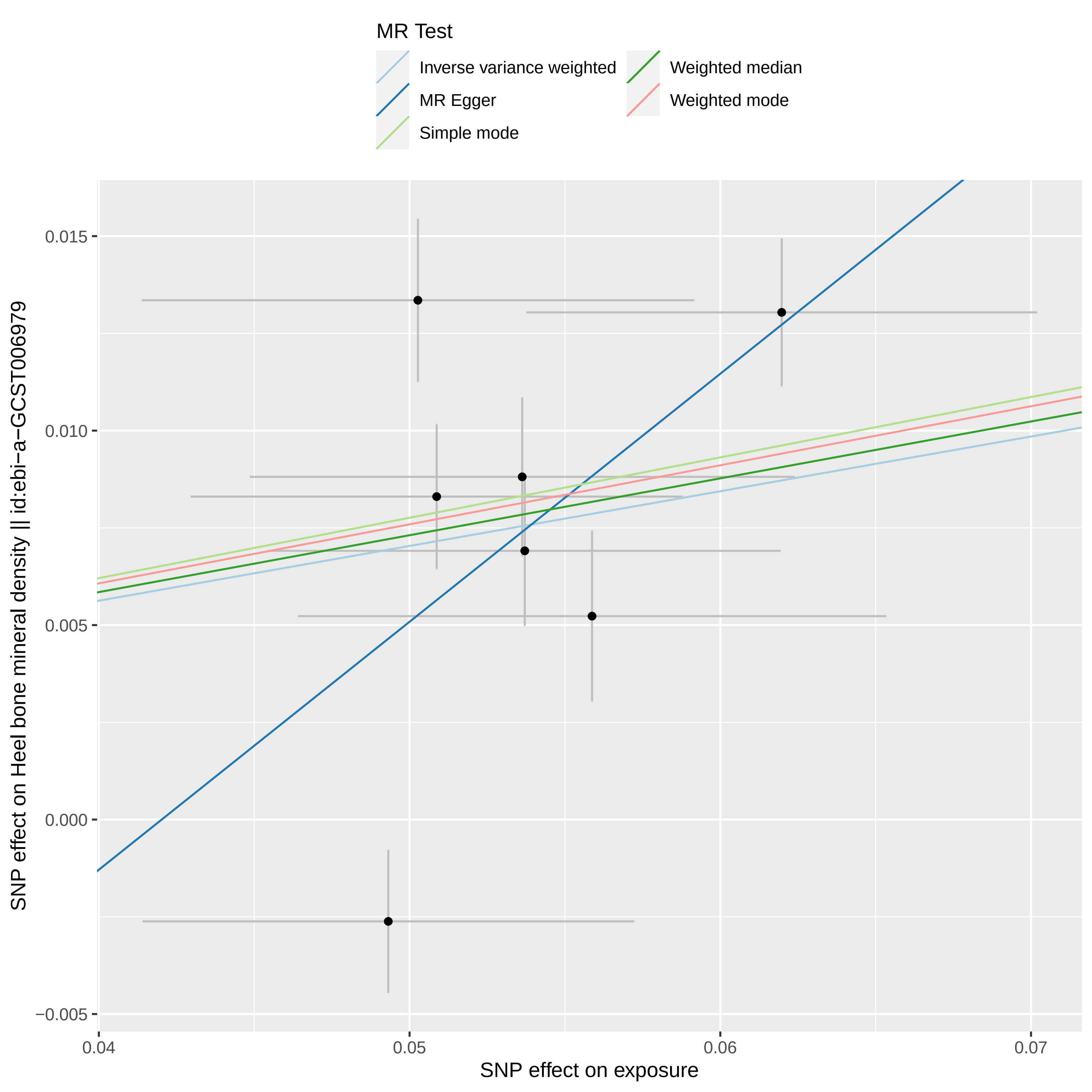

MR Test
Inverse variance weighted
MR Egger
Simple mode
Weighted median
Weighted mode
0.015
0.010
0.005
0.000
−0.005
0.04
0.05
0.06
0.07
SNP effect on exposure
SNP effect on Heel bone mineral density || id:ebi−a−GCST006979

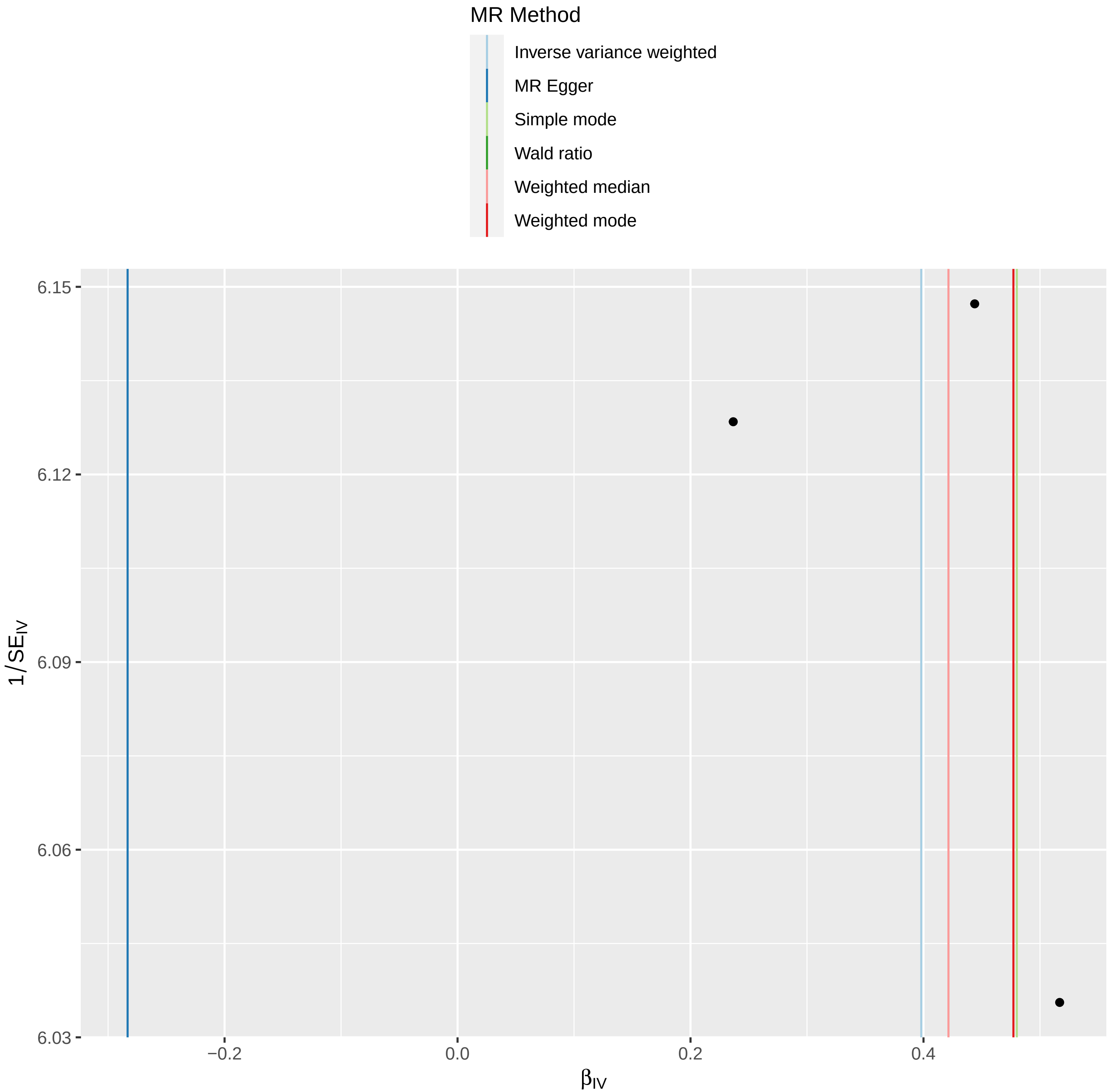

MR Method
Inverse variance weighted
MR Egger
Simple mode
Wald ratio
Weighted median
Weighted mode
6.15
6.12
6.09
6.06
6.03
−0.2
0.0
0.2
0.4
$1/SE_{IV}$
β_{IV}

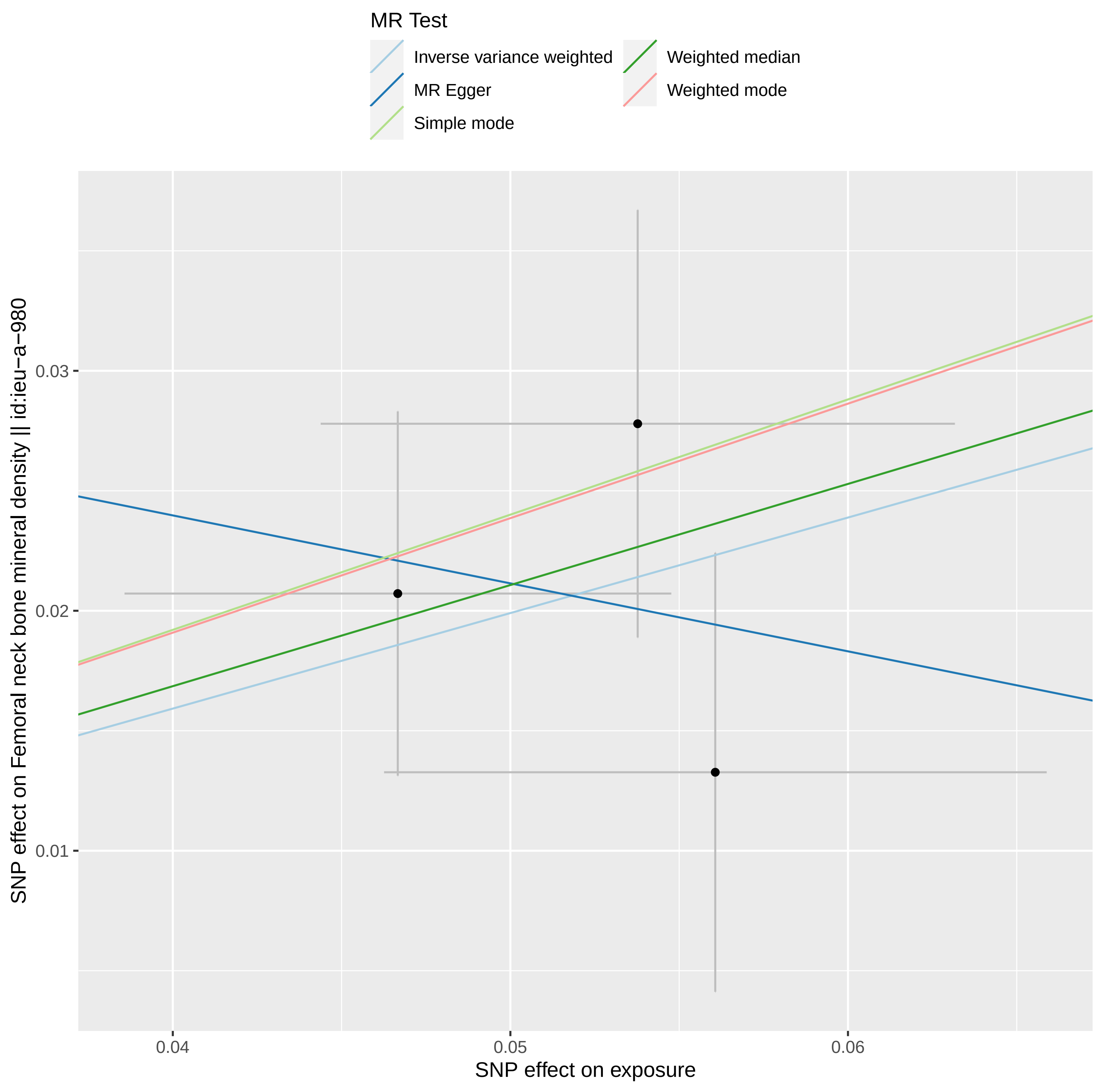

MR Test
Inverse variance weighted
MR Egger
Simple mode
Weighted median
Weighted mode
0.03
0.02
0.01
0.04
0.05
0.06
SNP effect on exposure
SNP effect on Femoral neck bone mineral density || id:ieu−a−980

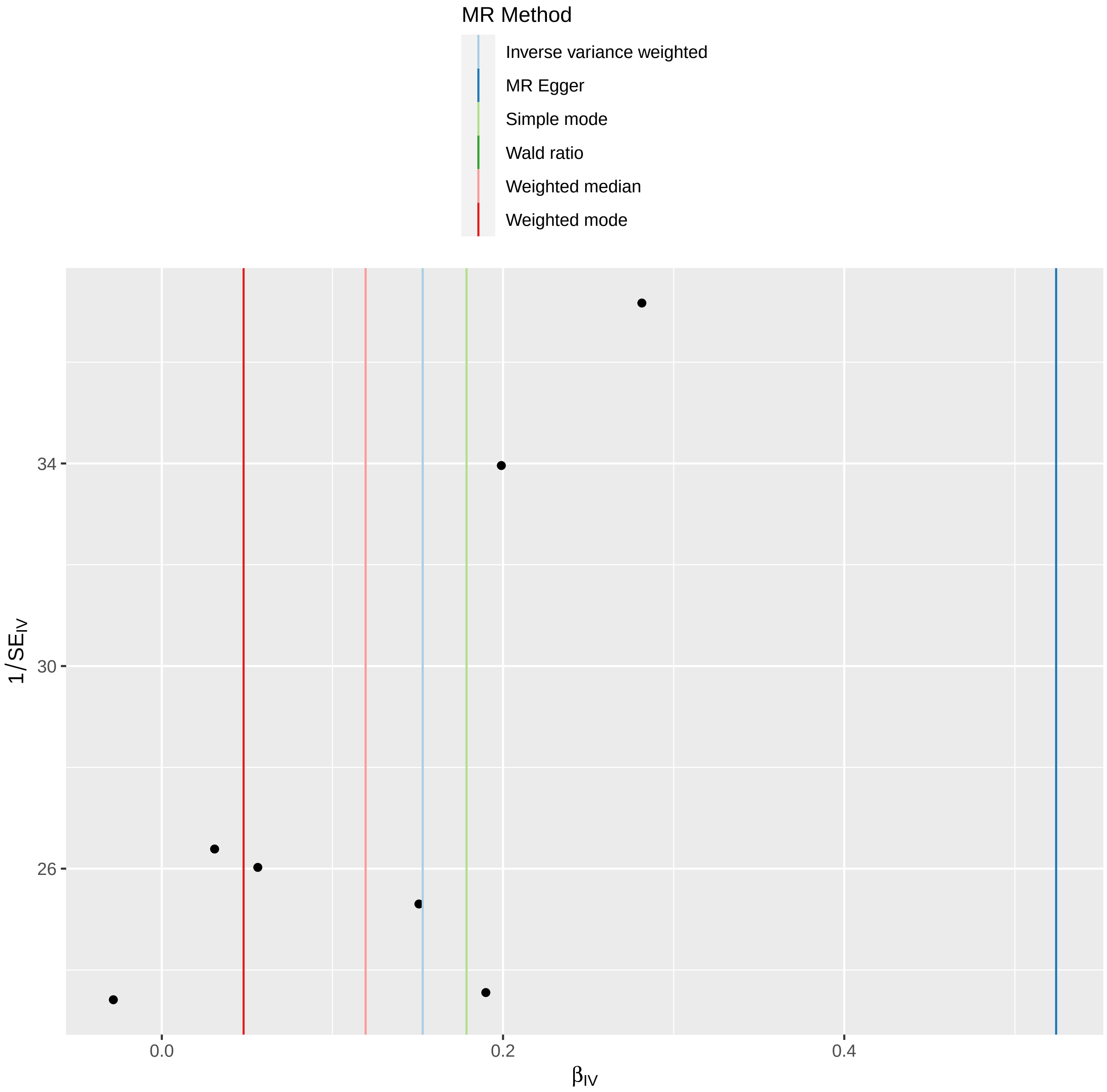
MR Method
Inverse variance weighted
MR Egger
Simple mode
Wald ratio
Weighted median
Weighted mode
34
30
26
1/SE$_{IV}$
0.0
0.2
0.4
β$_{IV}$

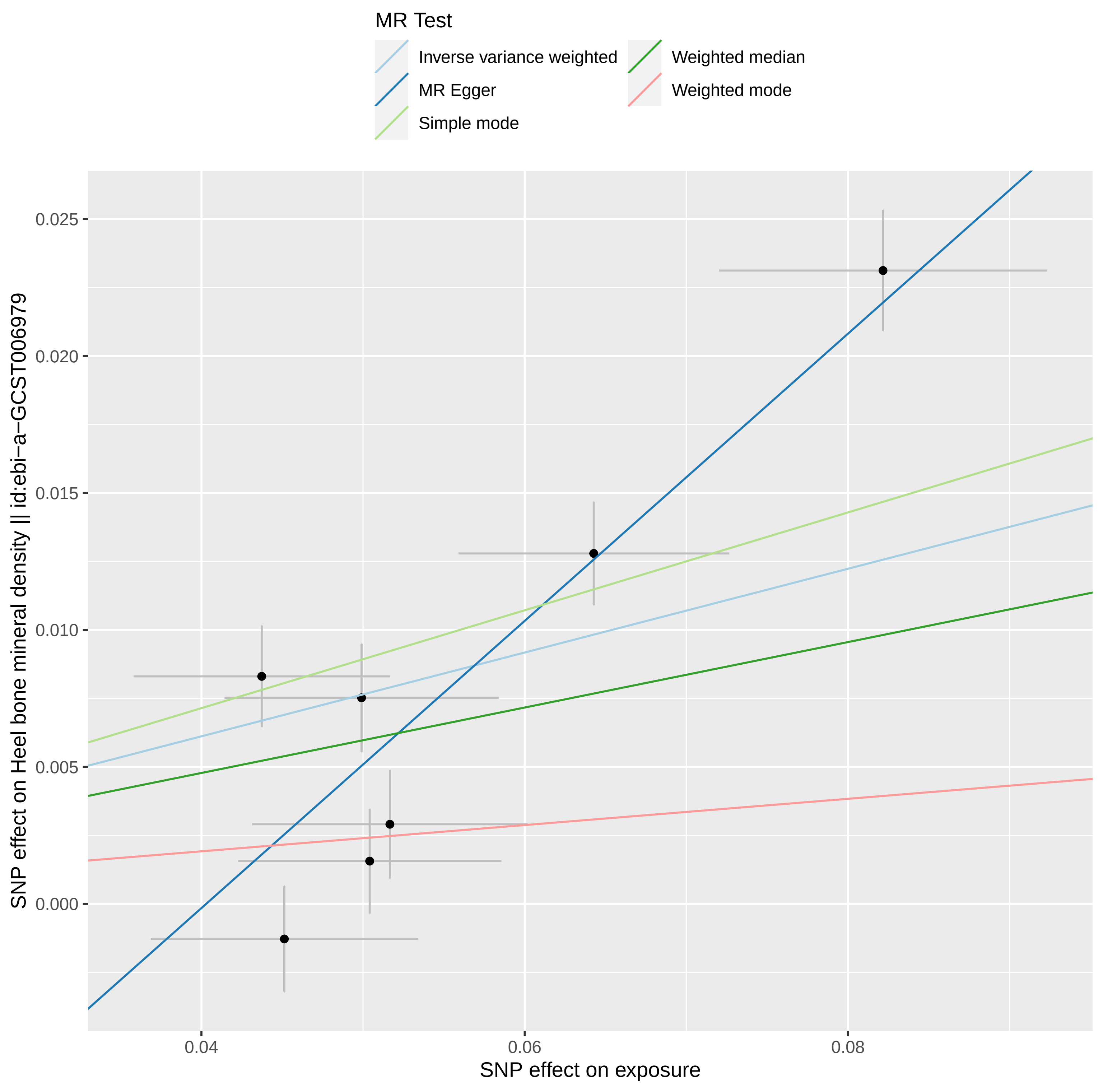

MR Test
Inverse variance weighted
MR Egger
Simple mode
Weighted median
Weighted mode
0.025
0.020
0.015
0.010
0.005
0.000
SNP effect on Heel bone mineral density || id:ebi−a−GCST006979
0.04
0.06
0.08
SNP effect on exposure

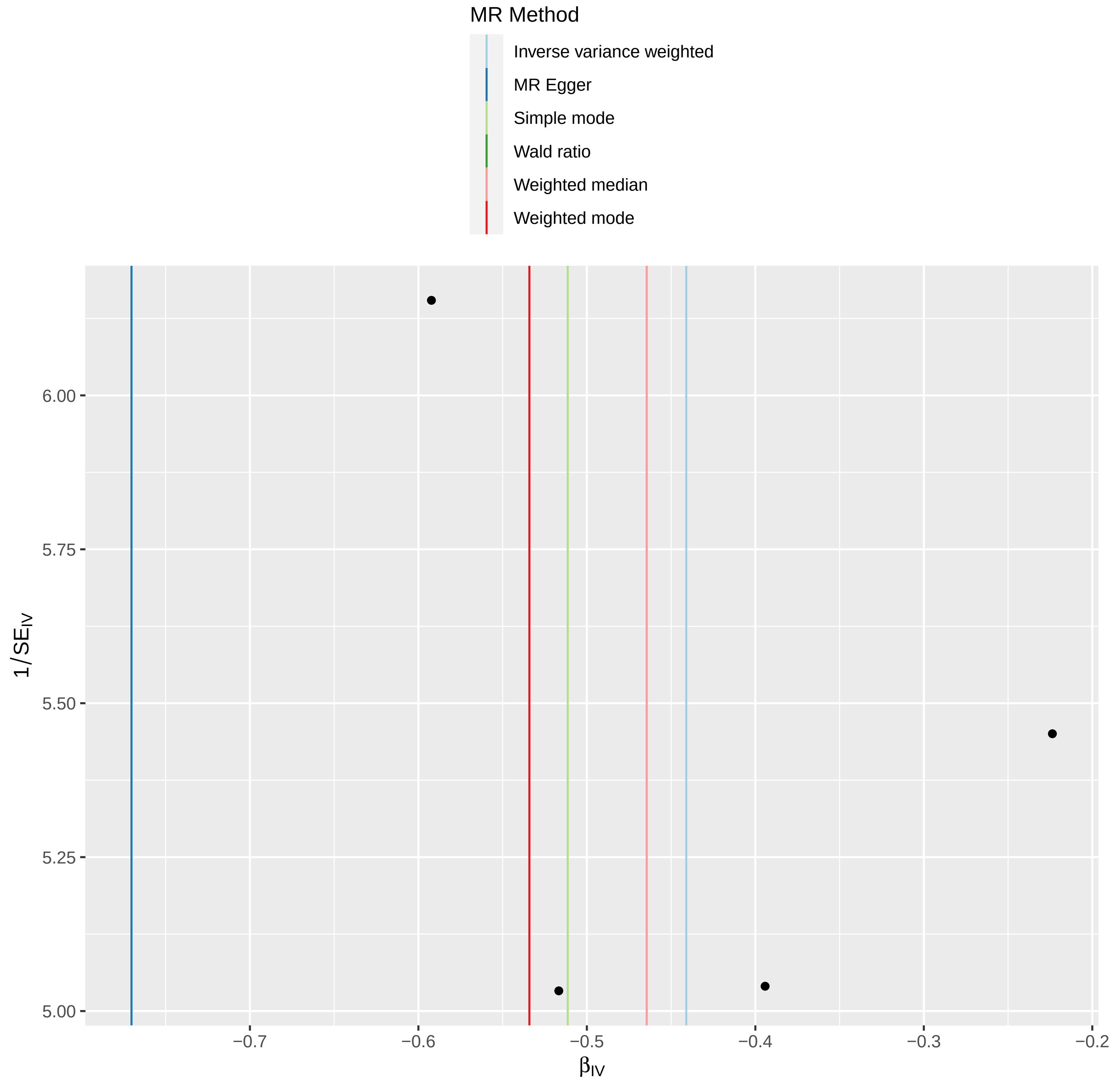

MR Method
Inverse variance weighted
MR Egger
Simple mode
Wald ratio
Weighted median
Weighted mode
6.00
5.75
5.50
5.25
5.00
1/SE_IV
−0.7
−0.6
−0.5
−0.4
−0.3
−0.2
β_IV

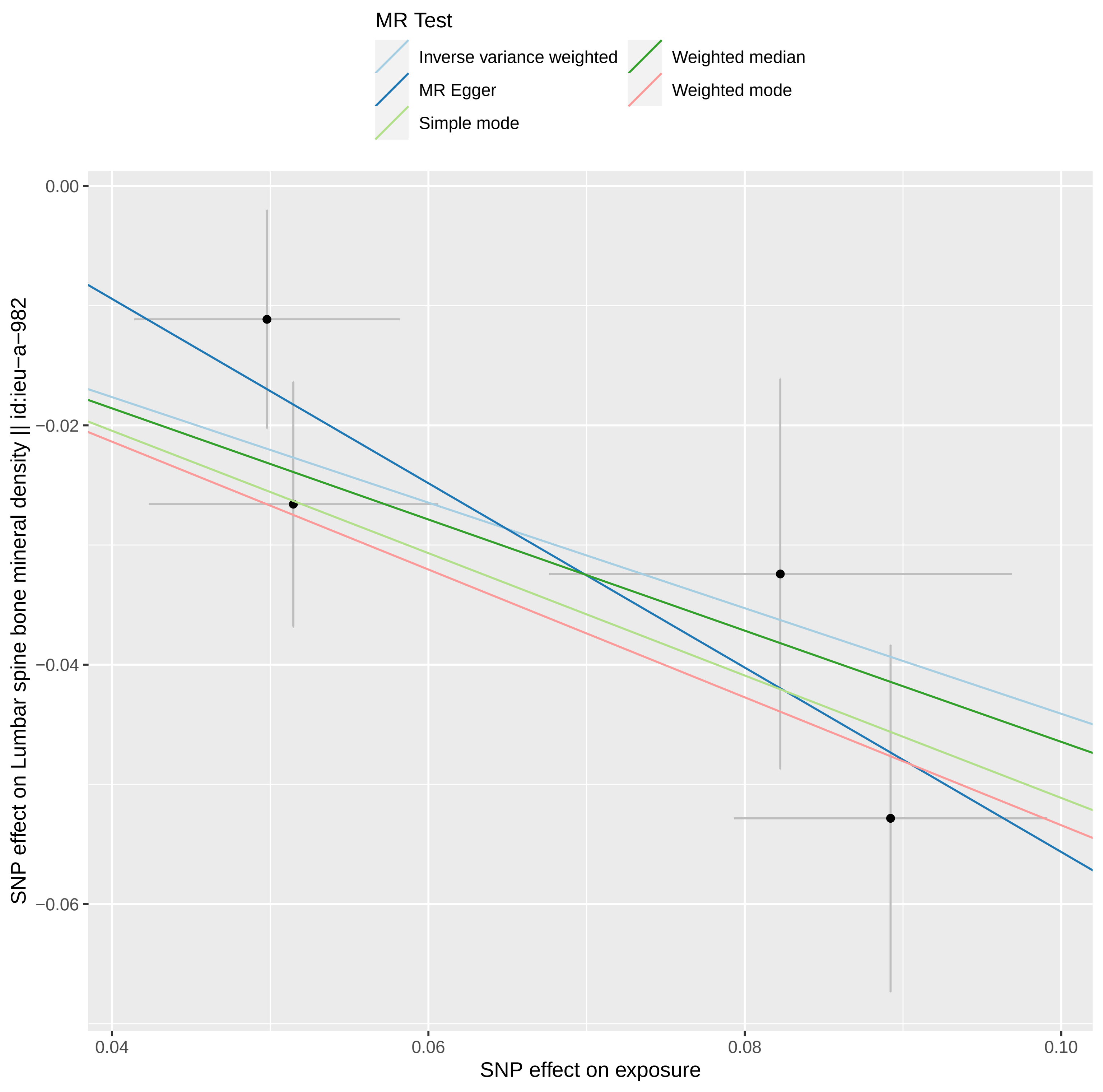
MR Test
Inverse variance weighted
MR Egger
Simple mode
Weighted median
Weighted mode
0.00
−0.02
−0.04
−0.06
0.04
0.06
0.08
0.10
SNP effect on Lumbar spine bone mineral density || id:ieu−a−982
SNP effect on exposure

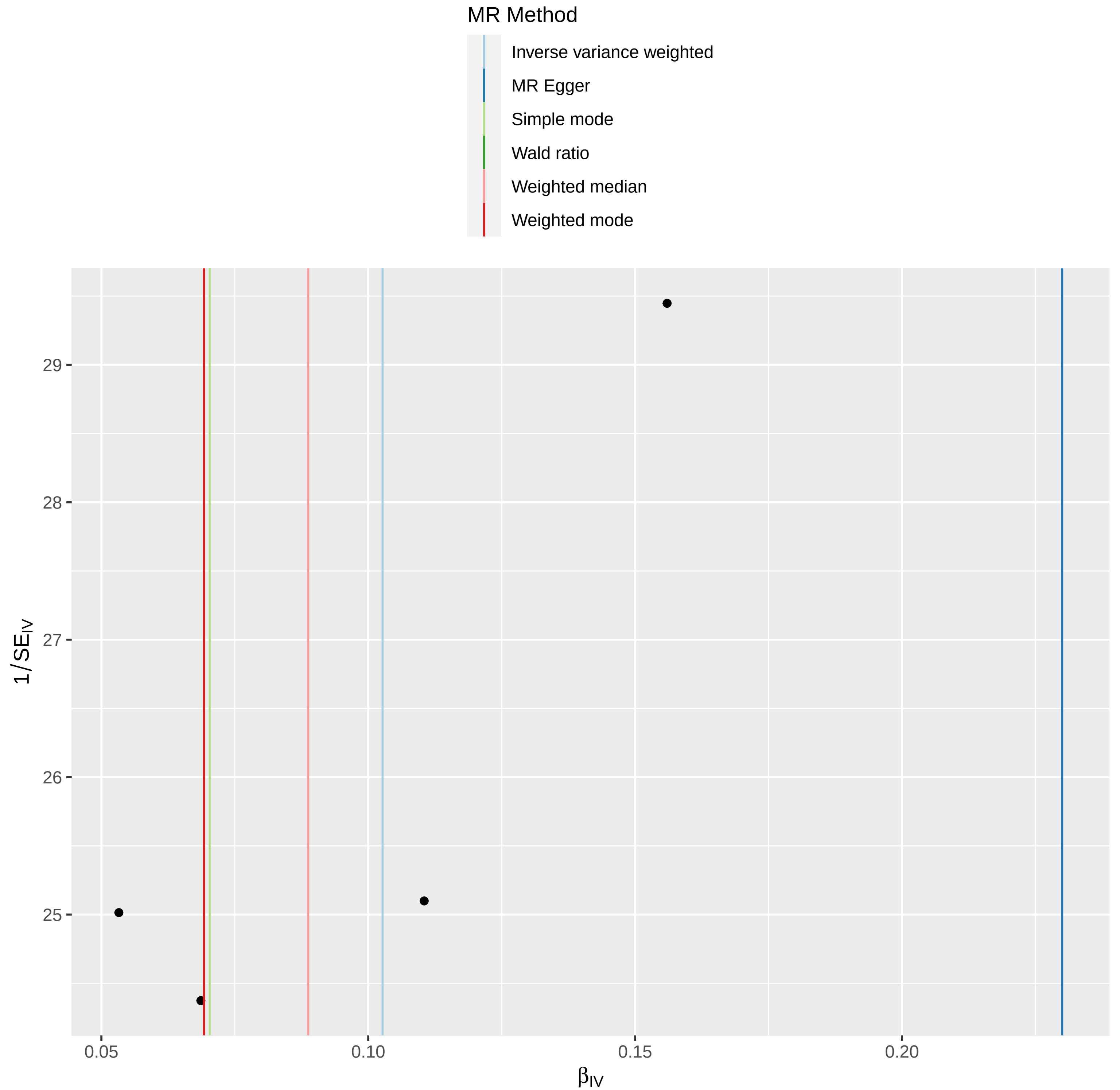

MR Method
Inverse variance weighted
MR Egger
Simple mode
Wald ratio
Weighted median
Weighted mode
29
28
27
26
25
$1/SE_{IV}$
0.05
0.10
0.15
0.20
β_{IV}

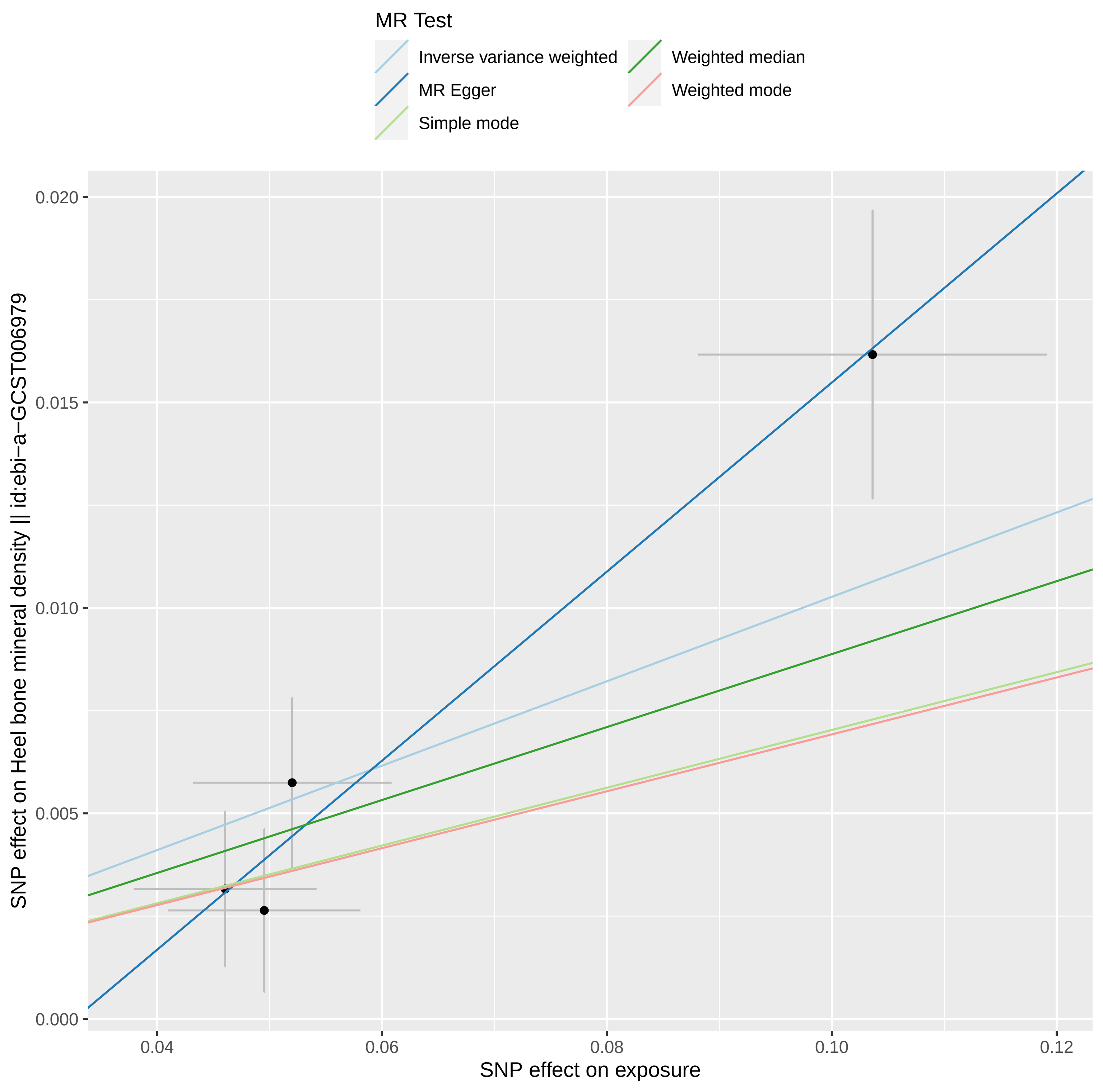

MR Test
Inverse variance weighted
MR Egger
Simple mode
Weighted median
Weighted mode
0.020
0.015
0.010
0.005
0.000
0.04
0.06
0.08
0.10
0.12
SNP effect on exposure
SNP effect on Heel bone mineral density || id:ebi−a−GCST006979

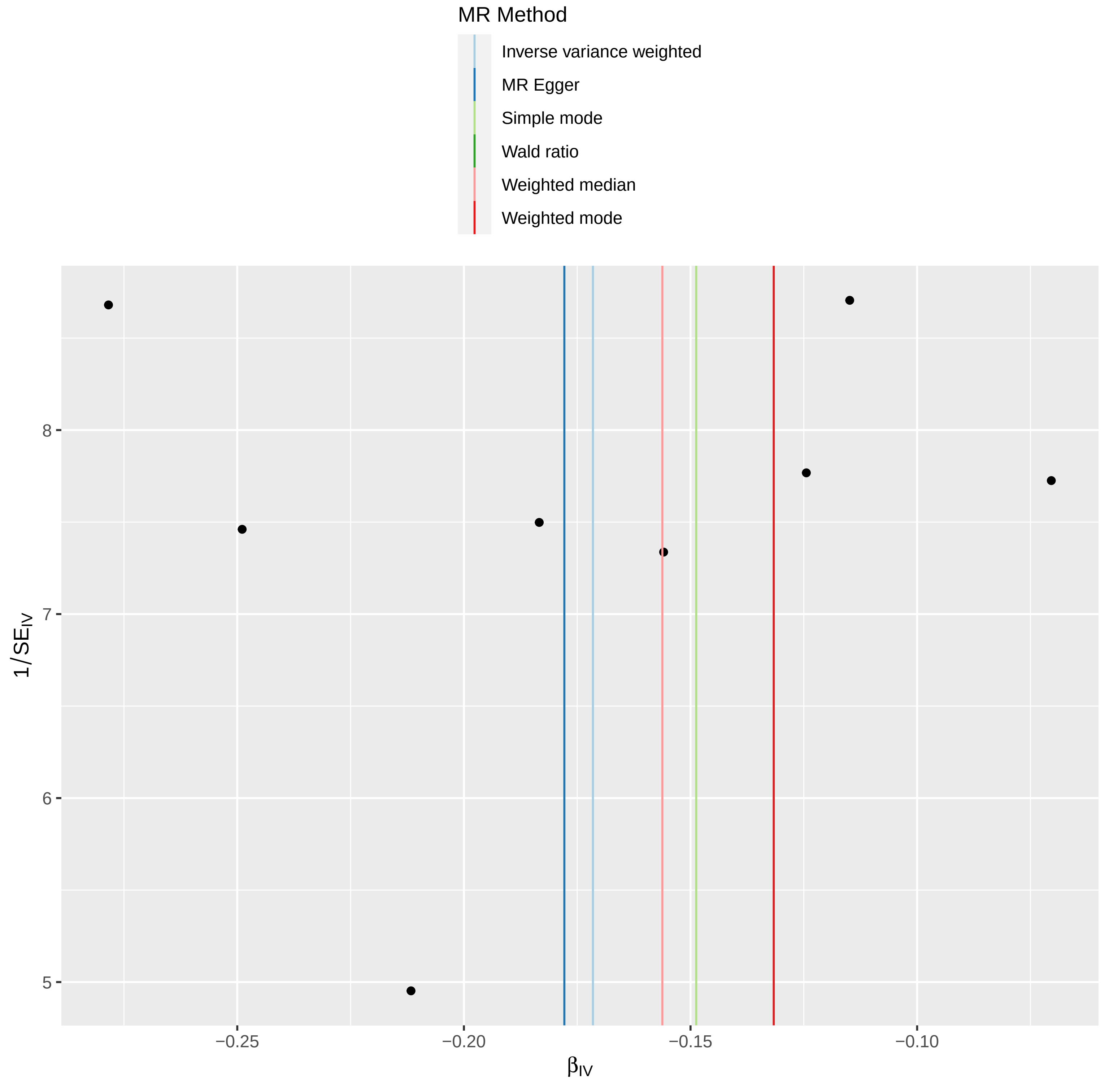

MR Method
Inverse variance weighted
MR Egger
Simple mode
Wald ratio
Weighted median
Weighted mode
8
7
6
5
1/SE_IV
−0.25
−0.20
−0.15
−0.10
β_IV

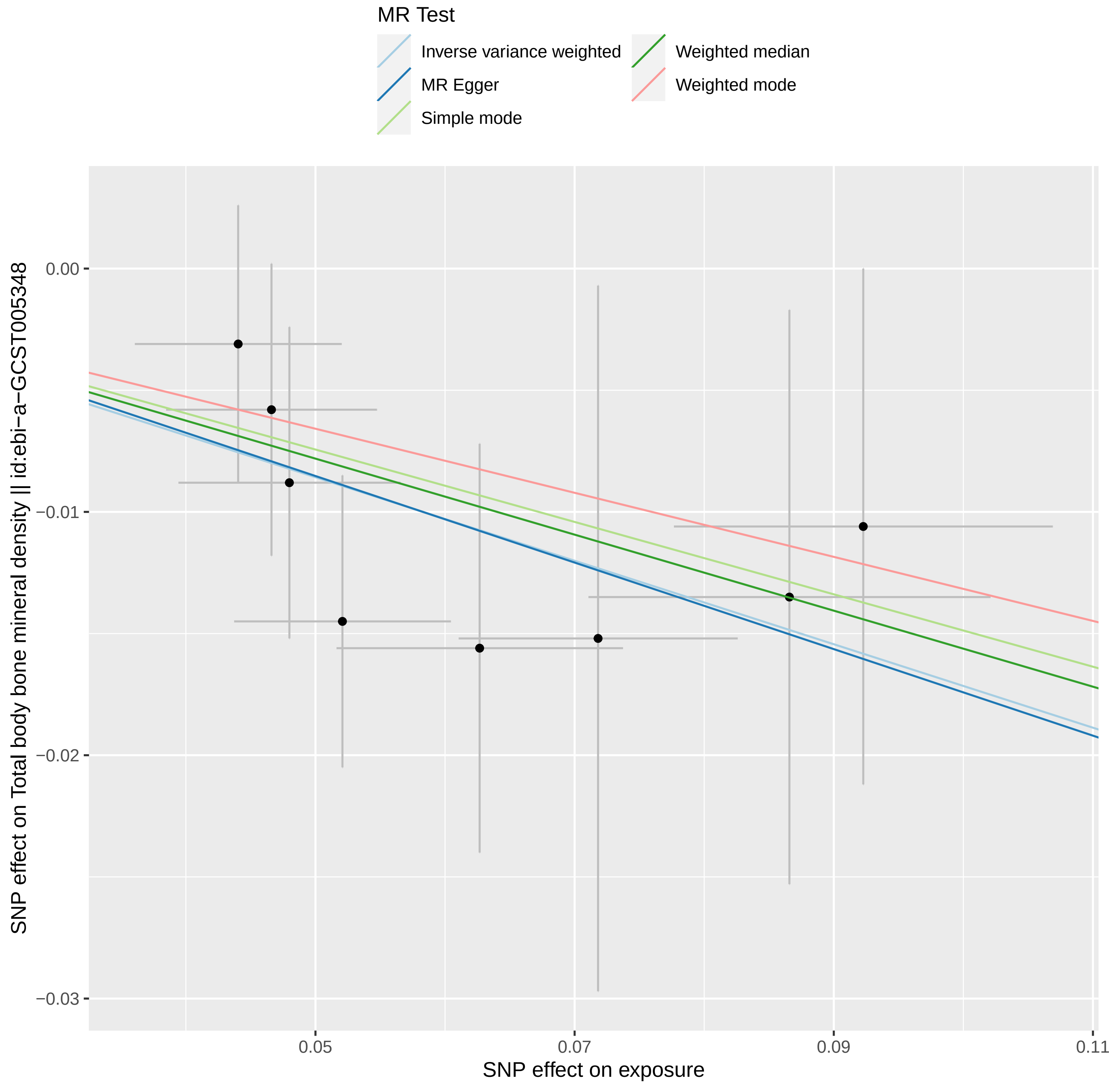
MR Test
Inverse variance weighted
MR Egger
Simple mode
Weighted median
Weighted mode
SNP effect on Total body bone mineral density || id:ebi−a−GCST005348
0.00
−0.01
−0.02
−0.03
0.05
0.07
0.09
0.11
SNP effect on exposure

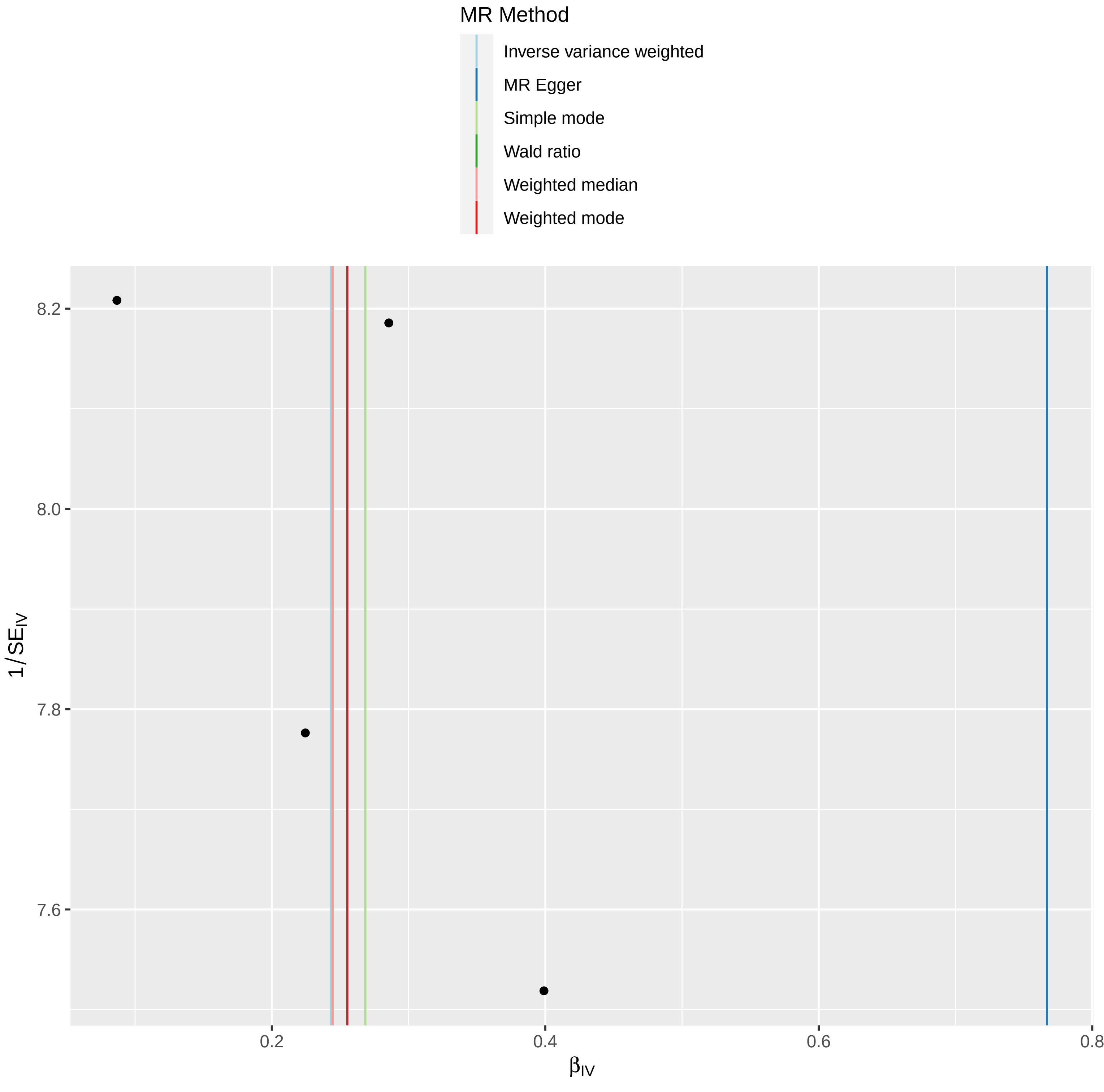
MR Method
Inverse variance weighted
MR Egger
Simple mode
Wald ratio
Weighted median
Weighted mode
8.2
8.0
7.8
7.6
1/SE_IV
0.2
0.4
0.6
0.8
β_IV

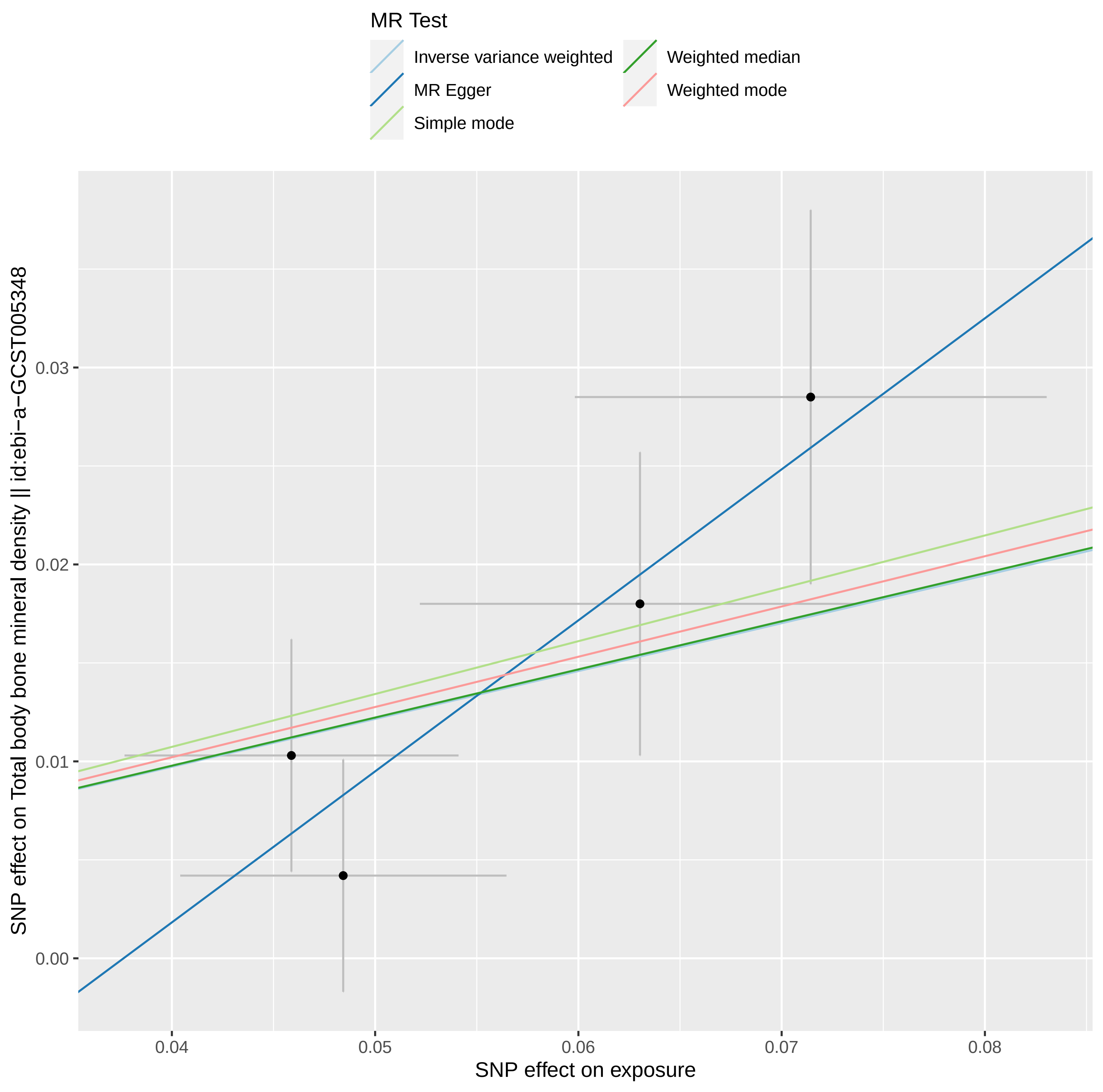
MR Test
Inverse variance weighted
MR Egger
Simple mode
Weighted median
Weighted mode
SNP effect on Total body bone mineral density || id:ebi-a-GCST005348
0.00
0.01
0.02
0.03
0.04
0.05
0.06
0.07
0.08
SNP effect on exposure

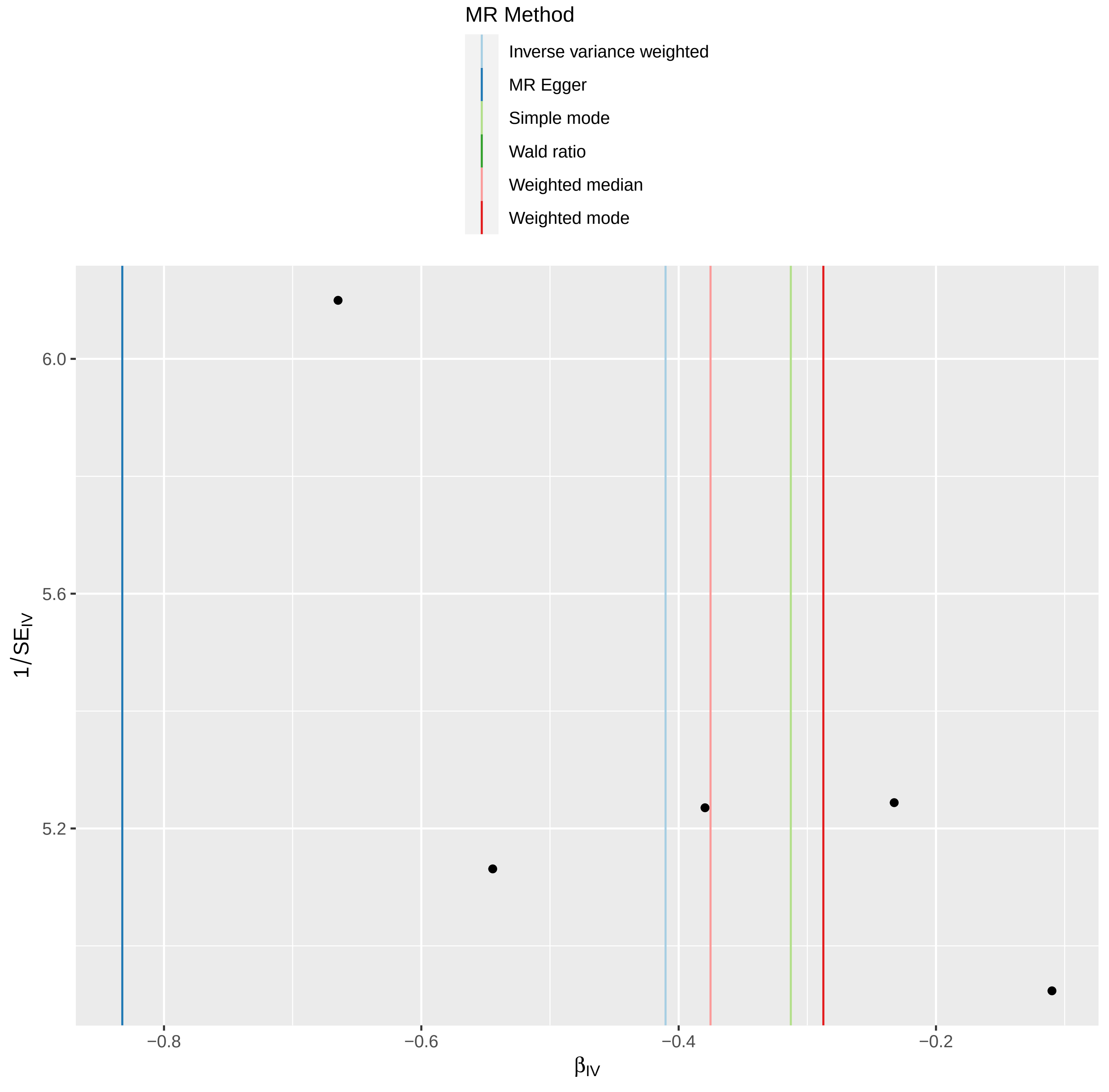
MR Method
Inverse variance weighted
MR Egger
Simple mode
Wald ratio
Weighted median
Weighted mode
6.0
5.6
5.2
1/SE_IV
−0.8
−0.6
−0.4
−0.2
β_IV

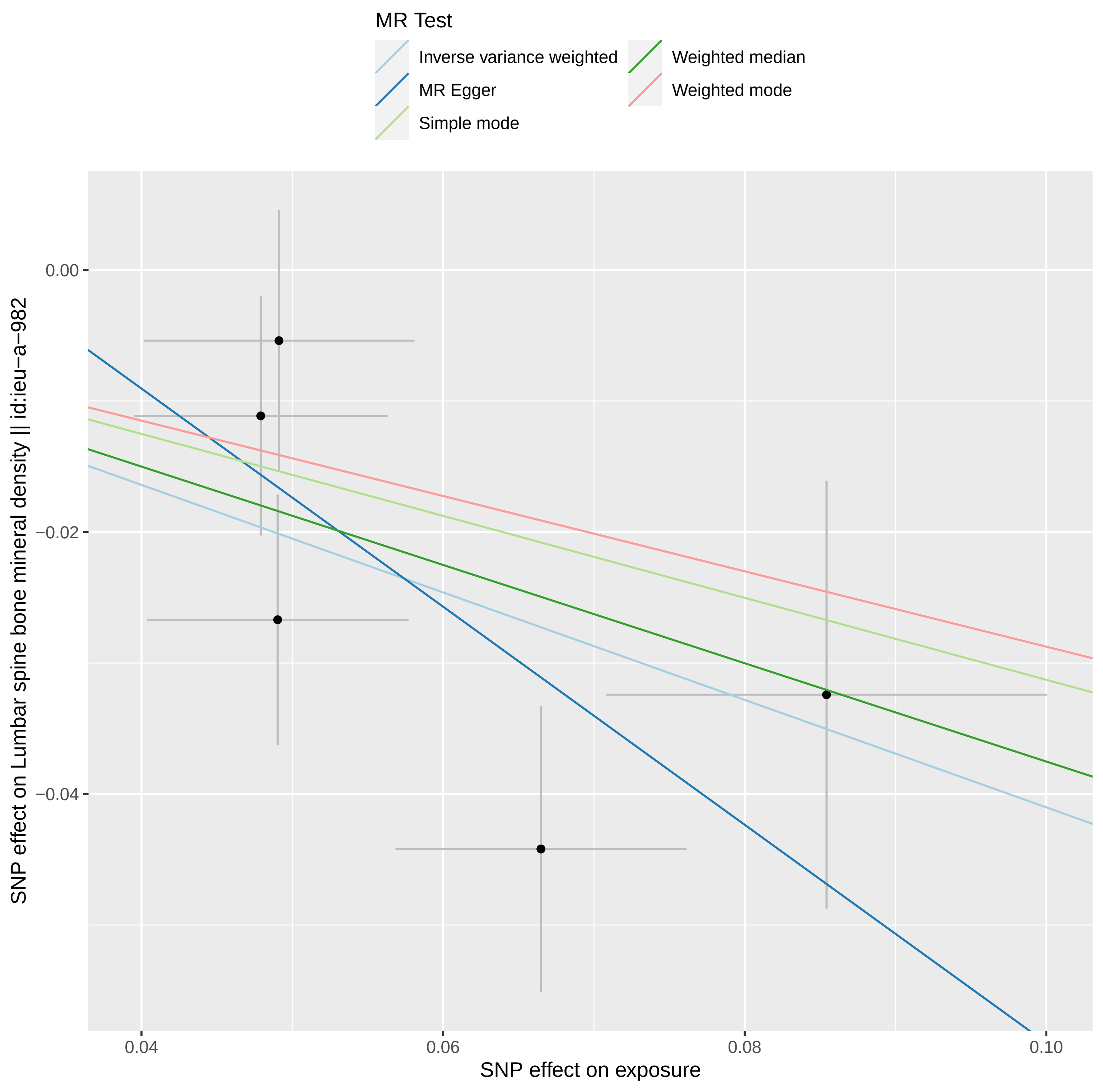
MR Test
Inverse variance weighted
MR Egger
Simple mode
Weighted median
Weighted mode
0.00
−0.02
−0.04
0.04
0.06
0.08
0.10
SNP effect on Lumbar spine bone mineral density || id:ieu−a−982
SNP effect on exposure

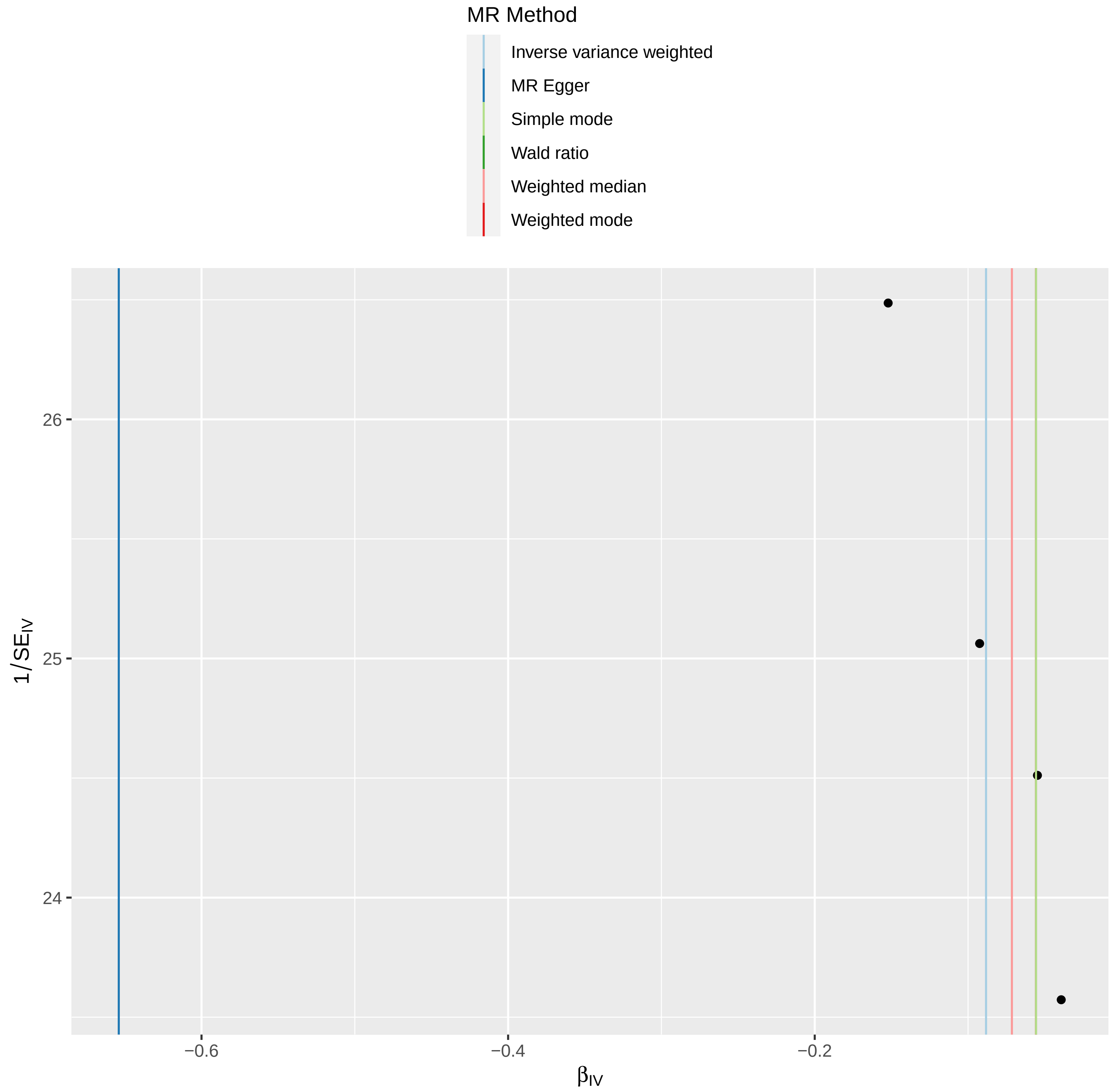

MR Method
Inverse variance weighted
MR Egger
Simple mode
Wald ratio
Weighted median
Weighted mode
26
25
24
1/SE_IV
−0.6
−0.4
−0.2
β_IV

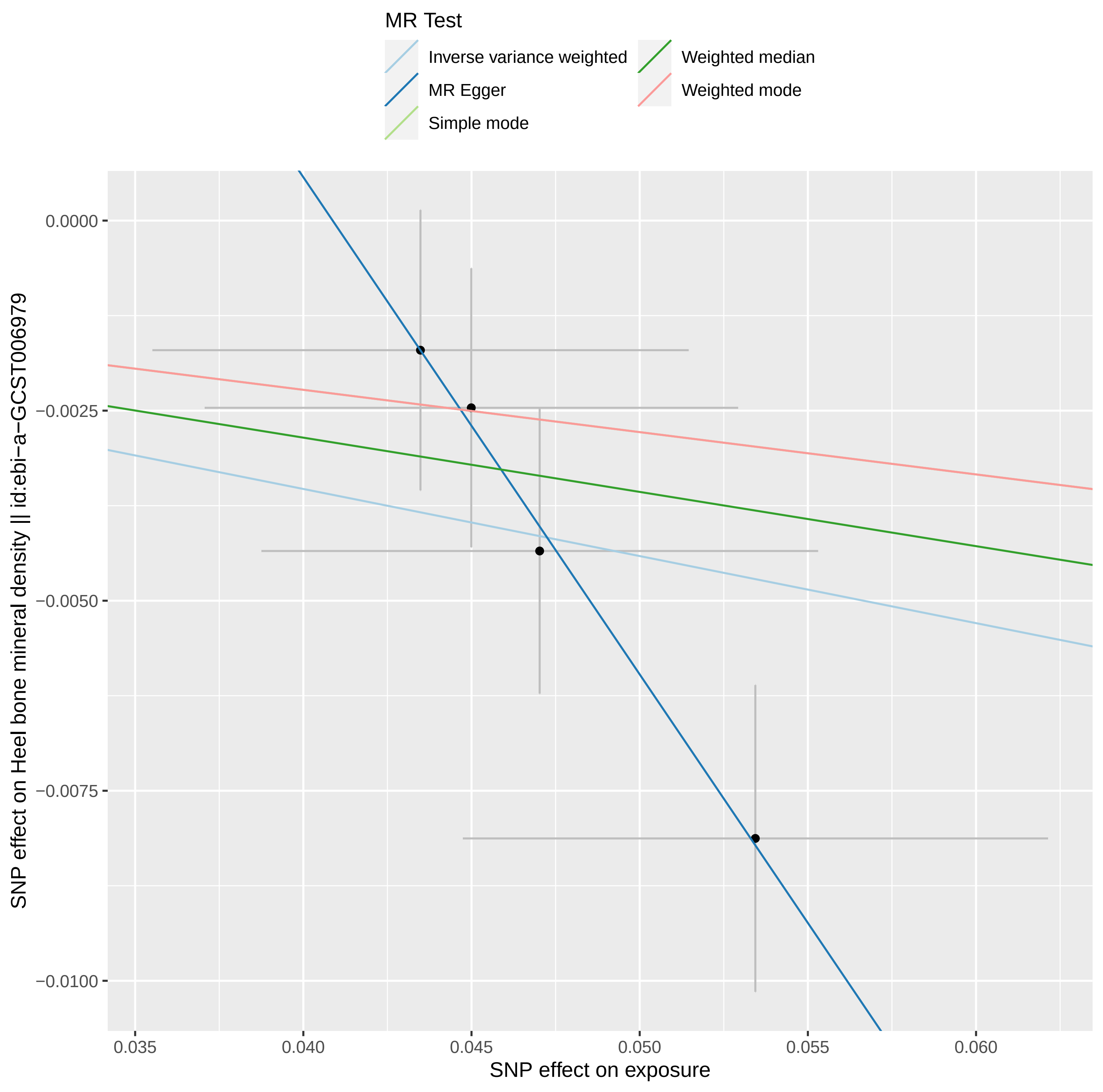
MR Test
Inverse variance weighted
MR Egger
Simple mode
Weighted median
Weighted mode
0.0000
−0.0025
−0.0050
−0.0075
−0.0100
SNP effect on Heel bone mineral density || id:ebi−a−GCST006979
0.035
0.040
0.045
0.050
0.055
0.060
SNP effect on exposure
