# Supplementary material for: Causal associations of brain structure with bone mineral density: a large-scale genetic correlation study
Source: Bone Res. 2023 Jul 20;11:37. doi: 10.1038/s41413-023-00270-z (PMC10359275; doi:10.1038/s41413-023-00270-z)
Supplement: Supplementary file 3 — Supplementary Table 1. Genetic correlation between BIDPs and BMD [file 41413_2023_270_MOESM3_ESM.pdf]

Supplemental table 1. Genetic correlation between BIDPs and BMD

No.	UKBID	IDP.short.name	Category name	Outcome (BMD)	rg	SE	pval	FDR.p
187	26536	aseg_global_volume-ratio_BrainSegVol-to-eTIV	aseg:global	Total body	-0.65	0.04	6.35E-53	1.46E-51
187	26536	aseg_global_volume-ratio_BrainSegVol-to-eTIV	aseg:global	Heel	-0.37	0.04	2.19E-20	5.04E-19
187	26536	aseg_global_volume-ratio_BrainSegVol-to-eTIV	aseg:global	Lumbar spine	-0.55	0.06	3.01E-17	6.93E-16
172	26521	aseg_global_volume_EstimatedTotalIntraCranial	aseg:global	Heel	0.31	0.04	6.61E-16	7.60E-15
172	26521	aseg_global_volume_EstimatedTotalIntraCranial	aseg:global	Total body	0.40	0.05	1.68E-14	1.93E-13
187	26536	aseg_global_volume-ratio_BrainSegVol-to-eTIV	aseg:global	Femoral neck	-0.50	0.07	3.15E-14	7.24E-13
163	25919	IDP_T1_FAST_ROIs_V_cerebellum_X	IDP T1:unilateral regions	Total body	-0.29	0.04	9.68E-14	1.35E-11
172	26521	aseg_global_volume_EstimatedTotalIntraCranial	aseg:global	Femoral neck	0.48	0.07	6.27E-12	7.22E-11
172	26521	aseg_global_volume_EstimatedTotalIntraCranial	aseg:global	Lumbar spine	0.42	0.07	1.01E-09	1.16E-08
161	25917	IDP_T1_FAST_ROIs_R_cerebellum_IX	IDP T1:unilateral regions	Total body	-0.20	0.03	1.84E-09	1.28E-07
165	26514	aseg_global_volume_BrainSeg	aseg:global	Total body	-0.28	0.05	2.15E-09	1.65E-08
159	25915	IDP_T1_FAST_ROIs_L_cerebellum_IX	IDP T1:unilateral regions	Total body	-0.19	0.03	4.78E-08	1.77E-06
160	25916	IDP_T1_FAST_ROIs_V_cerebellum_IX	IDP T1:unilateral regions	Total body	-0.20	0.04	5.10E-08	1.77E-06
167	26516	aseg_global_volume_BrainSegNotVentSurf	aseg:global	Total body	-0.26	0.05	5.45E-08	3.13E-07
158	25914	IDP_T1_FAST_ROIs_R_cerebellum_VIIIb	IDP T1:unilateral regions	Total body	-0.21	0.04	8.58E-08	2.39E-06
166	26515	aseg_global_volume_BrainSegNotVent	aseg:global	Total body	-0.26	0.05	9.56E-08	4.40E-07
170	26519	aseg_global_volume_SupraTentorial	aseg:global	Total body	-0.24	0.04	1.33E-07	5.08E-07
163	25919	IDP_T1_FAST_ROIs_V_cerebellum_X	IDP T1:unilateral regions	Heel	-0.15	0.03	1.58E-07	2.20E-05
137	25893	IDP_T1_FAST_ROIs_L_cerebellum_I-IV	IDP T1:unilateral regions	Total body	-0.19	0.04	2.40E-07	5.56E-06
54	25810	IDP_T1_FAST_ROIs_L_inf_temp_gyrus_post	IDP T1:unilateral regions	Total body	0.25	0.05	4.14E-07	8.12E-06
89	25845	IDP_T1_FAST_ROIs_R_cuneal_cortex	IDP T1:unilateral regions	Heel	0.17	0.03	4.88E-07	3.39E-05
138	25894	IDP_T1_FAST_ROIs_R_cerebellum_I-IV	IDP T1:unilateral regions	Total body	-0.18	0.04	5.75E-07	8.12E-06
155	25911	IDP_T1_FAST_ROIs_R_cerebellum_VIIIa	IDP T1:unilateral regions	Total body	-0.19	0.04	5.75E-07	8.12E-06
164	25920	IDP_T1_FAST_ROIs_R_cerebellum_X	IDP T1:unilateral regions	Total body	0.21	0.04	5.84E-07	8.12E-06
139	25895	IDP_T1_FAST_ROIs_L_cerebellum_V	IDP T1:unilateral regions	Total body	-0.17	0.04	2.26E-06	2.86E-05
171	26520	aseg_global_volume_SupraTentorialNotVent	aseg:global	Total body	-0.22	0.05	2.81E-06	8.40E-06
169	26518	aseg_global_volume_TotalGray	aseg:global	Total body	-0.24	0.05	2.92E-06	8.40E-06
140	25896	IDP_T1_FAST_ROIs_R_cerebellum_V	IDP T1:unilateral regions	Total body	-0.17	0.04	3.99E-06	4.62E-05
1	25001	IDP_T1_SIENAX_peripheral_grey_normalised_volume	IDP T1:global	Heel	0.14	0.03	4.03E-06	8.06E-05
211	26588	aseg_rh_volume_Cerebellum-Cortex	aseg:unilateral regions	Total body	-0.16	0.04	7.19E-06	2.44E-04

154	25910	IDP_T1_FAST_ROIs_V_cerebellum_VIIIa	IDP T1:unilateral regions	Total body	-0.16	0.04	8.08E-06	8.64E-05
156	25912	IDP_T1_FAST_ROIs_L_cerebellum_VIIIb	IDP T1:unilateral regions	Total body	-0.16	0.04	9.94E-06	9.36E-05
88	25844	IDP_T1_FAST_ROIs_L_cuneal_cortex	IDP T1:unilateral regions	Total body	0.19	0.04	1.01E-05	9.36E-05
121	25877	IDP_T1_FAST_ROIs_R_occ_pole	IDP T1:unilateral regions	Heel	0.13	0.03	1.26E-05	5.84E-04
89	25845	IDP_T1_FAST_ROIs_R_cuneal_cortex	IDP T1:unilateral regions	Total body	0.21	0.05	1.94E-05	1.69E-04
160	25916	IDP_T1_FAST_ROIs_V_cerebellum_IX	IDP T1:unilateral regions	Heel	-0.15	0.03	1.98E-05	6.88E-04
852	27247	aparc-DKTatlas_rh_area_medialorbitofrontal	Desikan Atlas	Total body	-0.19	0.05	2.14E-05	4.19E-03
127	25883	IDP_T1_FAST_ROIs_R_putamen	IDP T1:unilateral regions	Femoral neck	-0.24	0.06	2.18E-05	1.74E-03
36	25792	IDP_T1_FAST_ROIs_L_inf_front_gyrus_parsop	IDP T1:unilateral regions	Femoral neck	0.39	0.09	2.90E-05	1.74E-03
164	25920	IDP_T1_FAST_ROIs_R_cerebellum_X	IDP T1:unilateral regions	Lumbar spine	0.25	0.06	2.95E-05	4.10E-03
508	27485	aparc-a2009s_lh_volume_G-cingul-Post-dorsal	Destrieux Atlas	Total body	-0.19	0.04	3.06E-05	3.47E-03
164	25920	IDP_T1_FAST_ROIs_R_cerebellum_X	IDP T1:unilateral regions	Heel	0.12	0.03	3.71E-05	1.03E-03
993	27598	aparc-a2009s_rh_area_S-circular-insula-inf	Destrieux Atlas	Total body	-0.17	0.04	4.10E-05	6.07E-03
190	26553	aseg_lh_volume_CerebralWhiteMatter	aseg:unilateral regions	Total body	-0.16	0.04	4.20E-05	5.12E-04
207	26584	aseg_rh_volume_CerebralWhiteMatter	aseg:unilateral regions	Total body	-0.16	0.04	4.52E-05	5.12E-04
531	27508	aparc-a2009s_lh_volume_G-subcallosal	Destrieux Atlas	Total body	-0.23	0.06	4.69E-05	3.47E-03
155	25911	IDP_T1_FAST_ROIs_R_cerebellum_VIIIa	IDP T1:unilateral regions	Femoral neck	-0.24	0.06	4.78E-05	1.74E-03
412	27089	BA-exvivo_lh_volume_BA3a	Broadmann Atlas	Total body	-0.22	0.06	5.00E-05	1.40E-03
135	25891	IDP_T1_FAST_ROIs_R_ventral_striatum	IDP T1:unilateral regions	Total body	-0.16	0.04	6.18E-05	4.80E-04
36	25792	IDP_T1_FAST_ROIs_L_inf_front_gyrus_parsop	IDP T1:unilateral regions	Total body	0.24	0.06	6.21E-05	4.80E-04
158	25914	IDP_T1_FAST_ROIs_R_cerebellum_VIIIb	IDP T1:unilateral regions	Femoral neck	-0.23	0.06	6.38E-05	1.74E-03
1	25001	IDP_T1_SIENAX_peripheral_grey_normalised_volume	IDP T1:global	Femoral neck	0.23	0.06	6.48E-05	1.30E-03
56	25812	IDP_T1_FAST_ROIs_L_inf_temp_gyrus_tempocc	IDP T1:unilateral regions	Total body	0.18	0.05	7.27E-05	5.10E-04
32	25788	IDP_T1_FAST_ROIs_L_mid_front_gyrus	IDP T1:unilateral regions	Total body	0.19	0.05	7.34E-05	5.10E-04
126	25882	IDP_T1_FAST_ROIs_L_putamen	IDP T1:unilateral regions	Femoral neck	-0.22	0.06	7.45E-05	1.74E-03
88	25844	IDP_T1_FAST_ROIs_L_cuneal_cortex	IDP T1:unilateral regions	Heel	0.14	0.04	7.76E-05	1.80E-03
54	25810	IDP_T1_FAST_ROIs_L_inf_temp_gyrus_post	IDP T1:unilateral regions	Femoral neck	0.29	0.07	8.61E-05	1.74E-03
161	25917	IDP_T1_FAST_ROIs_R_cerebellum_IX	IDP T1:unilateral regions	Femoral neck	-0.20	0.05	8.76E-05	1.74E-03
649	26722	aparc-Desikan_lh_area_bankssts	Desikan Atlas	Total body	-0.18	0.05	9.58E-05	8.40E-03
33	25789	IDP_T1_FAST_ROIs_R_mid_front_gyrus	IDP T1:unilateral regions	Total body	0.18	0.05	9.84E-05	6.04E-04
106	25862	IDP_T1_FAST_ROIs_L_front_operc_cortex	IDP T1:unilateral regions	Heel	0.13	0.03	1.00E-04	1.85E-03

140	25896	IDP_T1_FAST_ROIs_R_cerebellum_V	IDP T1:unilateral regions	Heel	-0.10	0.03	1.00E-04	1.85E-03
342	26719	Brainstem_global_volume_Midbrain	Brain Stem	Total body	-0.14	0.04	1.00E-04	5.00E-04
44	25800	IDP_T1_FAST_ROIs_L_sup_temp_gyrus_post	IDP T1:unilateral regions	Total body	-0.20	0.05	1.00E-04	6.04E-04
134	25890	IDP_T1_FAST_ROIs_L_ventral_striatum	IDP T1:unilateral regions	Total body	-0.16	0.04	1.00E-04	6.04E-04
142	25898	IDP_T1_FAST_ROIs_V_cerebellum_VI	IDP T1:unilateral regions	Total body	-0.14	0.04	1.00E-04	6.04E-04
189	26552	aseg_lh_volume_Cortex	aseg:unilateral regions	Total body	-0.19	0.05	1.00E-04	7.56E-04
193	26556	aseg_lh_volume_Cerebellum-White-Matter	aseg:unilateral regions	Total body	-0.15	0.04	1.00E-04	7.56E-04
572	27549	aparc-a2009s_lh_volume_S-temporal-sup	Destrieux Atlas	Total body	-0.19	0.05	1.00E-04	4.93E-03
294	26671	ThalamNuclei_lh_volume_VLa	Thalamus Nuclei	Total body	-0.12	0.03	1.00E-04	5.20E-03
977	27582	aparc-a2009s_rh_area_G-subcallosal	Destrieux Atlas	Total body	-0.29	0.08	1.00E-04	7.40E-03
344	26789	aparc-Desikan_lh_volume_bankssts	Desikan Atlas	Total body	-0.19	0.05	1.00E-04	9.60E-03
139	25895	IDP_T1_FAST_ROIs_L_cerebellum_V	IDP T1:unilateral regions	Heel	-0.09	0.02	2.00E-04	2.78E-03
34	25790	IDP_T1_FAST_ROIs_L_inf_front_gyrus_parstri	IDP T1:unilateral regions	Heel	0.16	0.04	2.00E-04	2.78E-03
161	25917	IDP_T1_FAST_ROIs_R_cerebellum_IX	IDP T1:unilateral regions	Heel	-0.12	0.03	2.00E-04	2.78E-03
188	26537	aseg_global_volume-ratio_MaskVol-to-eTIV	aseg:global	Total body	-0.26	0.07	2.00E-04	5.11E-04
837	27170	aparc-DKTatlas_lh_area_superiortemporal	Desikan Atlas	Total body	-0.15	0.04	2.00E-04	8.40E-03
715	26855	aparc-Desikan_rh_area_insula	Desikan Atlas	Total body	-0.16	0.04	2.00E-04	8.40E-03
695	26835	aparc-Desikan_rh_area_medialorbitofrontal	Desikan Atlas	Total body	-0.17	0.05	2.00E-04	8.40E-03
465	27232	aparc-DKTatlas_lh_volume_superiortemporal	Desikan Atlas	Total body	-0.17	0.05	2.00E-04	9.60E-03
932	27389	aparc-a2009s_lh_area_S-oc-temp-med+Lingual	Destrieux Atlas	Total body	-0.20	0.05	2.00E-04	9.87E-03
187	26536	aseg_global_volume-ratio_BrainSegVol-to-eTIV	aseg:global	Forearm	-0.64	0.18	3.00E-04	6.60E-03
138	25894	IDP_T1_FAST_ROIs_R_cerebellum_I-IV	IDP T1:unilateral regions	Heel	-0.10	0.03	3.00E-04	3.34E-03
155	25911	IDP_T1_FAST_ROIs_R_cerebellum_VIIIa	IDP T1:unilateral regions	Heel	-0.14	0.04	3.00E-04	3.34E-03
141	25897	IDP_T1_FAST_ROIs_L_cerebellum_VI	IDP T1:unilateral regions	Total body	-0.14	0.04	3.00E-04	1.64E-03
136	25892	IDP_T1_FAST_ROIs_brain_stem	IDP T1:unilateral regions	Total body	0.14	0.04	3.00E-04	1.64E-03
194	26557	aseg_lh_volume_Cerebellum-Cortex	aseg:unilateral regions	Total body	-0.13	0.04	3.00E-04	1.70E-03
301	26678	ThalamNuclei_lh_volume_VA	Thalamus Nuclei	Total body	-0.13	0.03	3.00E-04	6.93E-03
821	27154	aparc-DKTatlas_lh_area_medialorbitofrontal	Desikan Atlas	Total body	-0.18	0.05	3.00E-04	8.40E-03
823	27156	aparc-DKTatlas_lh_area_parahippocampal	Desikan Atlas	Total body	-0.17	0.05	3.00E-04	8.40E-03
868	27263	aparc-DKTatlas_rh_area_superiortemporal	Desikan Atlas	Total body	-0.16	0.04	3.00E-04	8.40E-03
405	26918	aparc-Desikan_rh_volume_superiortemporal	Desikan Atlas	Total body	-0.16	0.04	3.00E-04	9.60E-03
372	26817	aparc-Desikan_lh_volume_superiortemporal	Desikan Atlas	Total body	-0.16	0.04	3.00E-04	9.60E-03
496	27325	aparc-DKTatlas_rh_volume_superiortemporal	Desikan Atlas	Total body	-0.16	0.04	3.00E-04	9.60E-03

605	27730	aparc-a2009s_rh_volume_G-subcallosal	Destrieux Atlas	Total body	-0.20	0.06	3.00E-04	1.11E-02
159	25915	IDP_T1_FAST_ROIs_L_cerebellum_IX	IDP T1:unilateral regions	Heel	-0.12	0.03	4.00E-04	3.83E-03
87	25843	IDP_T1_FAST_ROIs_R_precun_cortex	IDP T1:unilateral regions	Heel	0.14	0.04	4.00E-04	3.83E-03
646	27771	aparc-a2009s_rh_volume_S-temporal-sup	Destrieux Atlas	Heel	-0.11	0.03	4.00E-04	5.92E-02
309	26686	ThalamNuclei_lh_volume_VLp	Thalamus Nuclei	Total body	-0.11	0.03	4.00E-04	6.93E-03
582	27707	aparc-a2009s_rh_volume_G-cingul-Post-dorsal	Destrieux Atlas	Total body	-0.21	0.06	4.00E-04	1.14E-02
944	27401	aparc-a2009s_lh_area_S-temporal-sup	Destrieux Atlas	Total body	-0.15	0.04	4.00E-04	1.48E-02
164	25920	IDP_T1_FAST_ROIs_R_cerebellum_X	IDP T1:unilateral regions	Femoral neck	0.21	0.06	5.00E-04	8.18E-03
100	25856	IDP_T1_FAST_ROIs_L_temp_fusif_cortex_post	IDP T1:unilateral regions	Femoral neck	0.24	0.07	5.00E-04	8.18E-03
40	25796	IDP_T1_FAST_ROIs_L_temporal_pole	IDP T1:unilateral regions	Heel	0.10	0.03	5.00E-04	4.34E-03
157	25913	IDP_T1_FAST_ROIs_V_cerebellum_VIIIb	IDP T1:unilateral regions	Total body	-0.12	0.03	5.00E-04	2.57E-03
515	27492	aparc-a2009s_lh_volume_G-front-sup	Destrieux Atlas	Total body	-0.15	0.04	5.00E-04	1.14E-02
545	27522	aparc-a2009s_lh_volume_S-cingul-Marginalis	Destrieux Atlas	Total body	-0.18	0.05	5.00E-04	1.14E-02
184	26533	aseg_global_volume_CC-Central	aseg:global	Lumbar spine	-0.22	0.06	6.00E-04	4.60E-03
127	25883	IDP_T1_FAST_ROIs_R_putamen	IDP T1:unilateral regions	Lumbar spine	-0.19	0.06	6.00E-04	3.13E-02
136	25892	IDP_T1_FAST_ROIs_brain_stem	IDP T1:unilateral regions	Lumbar spine	0.19	0.05	6.00E-04	3.13E-02
121	25877	IDP_T1_FAST_ROIs_R_occ_pole	IDP T1:unilateral regions	Total body	0.15	0.04	6.00E-04	2.98E-03
426	27131	BA-exvivo_rh_volume_BA3a	Broadmann Atlas	Total body	-0.17	0.05	6.00E-04	8.40E-03
663	26736	aparc-Desikan_lh_area_parahippocampal	Desikan Atlas	Total body	-0.16	0.05	6.00E-04	1.24E-02
731	26938	aparc-pial_lh_area_parahippocampal	Desikan Atlas	Total body	-0.16	0.05	6.00E-04	1.24E-02
143	25899	IDP_T1_FAST_ROIs_R_cerebellum_VI	IDP T1:unilateral regions	Total body	-0.13	0.04	7.00E-04	3.36E-03
771	26978	aparc-pial_rh_area_posteriorcingulate	Desikan Atlas	Total body	-0.16	0.05	7.00E-04	1.25E-02
447	27214	aparc-DKTatlas_lh_volume_lateralorbitofrontal	Desikan Atlas	Total body	-0.16	0.05	7.00E-04	1.44E-02
879	27336	aparc-a2009s_lh_area_G+S-cingul-Mid-Post	Destrieux Atlas	Total body	-0.16	0.05	7.00E-04	1.81E-02
128	25884	IDP_T1_FAST_ROIs_L_pallidum	IDP T1:unilateral regions	Femoral neck	-0.22	0.07	8.00E-04	1.11E-02
903	27360	aparc-a2009s_lh_area_G-subcallosal	Destrieux Atlas	Heel	-0.19	0.06	8.00E-04	9.62E-02
127	25883	IDP_T1_FAST_ROIs_R_putamen	IDP T1:unilateral regions	Total body	-0.14	0.04	8.00E-04	3.71E-03
677	26750	aparc-Desikan_lh_area_superiortemporal	Desikan Atlas	Total body	-0.14	0.04	8.00E-04	1.31E-02
529	27506	aparc-a2009s_lh_volume_G-precuneus	Destrieux Atlas	Total body	-0.14	0.04	8.00E-04	1.48E-02
954	27559	aparc-a2009s_rh_area_G-cingul-Post-dorsal	Destrieux Atlas	Total body	-0.16	0.05	8.00E-04	1.81E-02
9	25009	IDP_T1_SIENAX_brain-normalised_volume	IDP T1:global	Femoral neck	0.21	0.06	9.00E-04	9.00E-03
36	25792	IDP_T1_FAST_ROIs_L_inf_front_gyrus_parsop	IDP T1:unilateral regions	Heel	0.21	0.06	9.00E-04	7.15E-03
135	25891	IDP_T1_FAST_ROIs_R_ventral_striatum	IDP T1:unilateral regions	Heel	-0.09	0.03	9.00E-04	7.15E-03

162	25918	IDP_T1_FAST_ROIs_L_cerebellum_X	IDP T1:unilateral regions	Lumbar spine	0.18	0.05	9.00E-04	3.13E-02
871	27266	aparc-DKTatlas_rh_area_insula	Desikan Atlas	Total body	-0.12	0.04	9.00E-04	1.31E-02
682	26822	aparc-Desikan_rh_area_TotalSurface	Desikan Atlas	Total body	-0.13	0.04	9.00E-04	1.31E-02
450	27217	aparc-DKTatlas_lh_volume_middletemporal	Desikan Atlas	Total body	-0.16	0.05	9.00E-04	1.44E-02
367	26812	aparc-Desikan_lh_volume_precuneus	Desikan Atlas	Total body	-0.13	0.04	9.00E-04	1.44E-02
460	27227	aparc-DKTatlas_lh_volume_precuneus	Desikan Atlas	Total body	-0.13	0.04	9.00E-04	1.44E-02
90	25846	IDP_T1_FAST_ROIs_L_front_orb_cortex	IDP T1:unilateral regions	Heel	0.11	0.03	1.00E-03	7.32E-03
1296	27669	aparc-a2009s_rh_thickness_S-central	Destrieux Atlas	Lumbar spine	-0.24	0.07	1.00E-03	1.48E-01
661	26734	aparc-Desikan_lh_area_medialorbitofrontal	Desikan Atlas	Total body	-0.19	0.06	1.00E-03	1.31E-02
544	27521	aparc-a2009s_lh_volume_S-central	Destrieux Atlas	Total body	-0.16	0.05	1.00E-03	1.59E-02
880	27337	aparc-a2009s_lh_area_G-cingul-Post-dorsal	Destrieux Atlas	Total body	-0.16	0.05	1.00E-03	1.81E-02
962	27567	aparc-a2009s_rh_area_G-Ins-lg+S-cent-ins	Destrieux Atlas	Total body	-0.15	0.05	1.00E-03	1.81E-02
798	27103	BA-exvivo_rh_area_BA3a	Broadmann Atlas	Total body	-0.15	0.04	1.00E-03	2.80E-02
127	25883	IDP_T1_FAST_ROIs_R_putamen	IDP T1:unilateral regions	Heel	-0.12	0.04	1.10E-03	7.65E-03
169	26518	aseg_global_volume_TotalGray	aseg:global	Lumbar spine	-0.23	0.07	1.10E-03	6.33E-03
206	26583	aseg_rh_volume_Cortex	aseg:unilateral regions	Lumbar spine	-0.23	0.07	1.10E-03	3.74E-02
162	25918	IDP_T1_FAST_ROIs_L_cerebellum_X	IDP T1:unilateral regions	Total body	0.13	0.04	1.10E-03	4.85E-03
65	25821	IDP_T1_FAST_ROIs_R_supramarg_gyrus_post	IDP T1:unilateral regions	Total body	0.25	0.08	1.10E-03	4.85E-03
854	27249	aparc-DKTatlas_rh_area_parahippocampal	Desikan Atlas	Total body	-0.14	0.04	1.10E-03	1.31E-02
711	26851	aparc-Desikan_rh_area_superiortemporal	Desikan Atlas	Total body	-0.15	0.05	1.10E-03	1.31E-02
887	27344	aparc-a2009s_lh_area_G-front-sup	Destrieux Atlas	Total body	-0.13	0.04	1.10E-03	1.81E-02
114	25870	IDP_T1_FAST_ROIs_L_heschl_gyrus	IDP T1:unilateral regions	Femoral neck	0.22	0.07	1.20E-03	1.49E-02
648	26721	aparc-Desikan_lh_area_TotalSurface	Desikan Atlas	Total body	-0.13	0.04	1.20E-03	1.31E-02
357	26802	aparc-Desikan_lh_volume_middletemporal	Desikan Atlas	Total body	-0.15	0.05	1.20E-03	1.46E-02
398	26911	aparc-Desikan_rh_volume_posteriorcingulate	Desikan Atlas	Total body	-0.17	0.05	1.20E-03	1.46E-02
562	27539	aparc-a2009s_lh_volume_S-orbital-med-olfact	Destrieux Atlas	Total body	-0.19	0.06	1.20E-03	1.59E-02
537	27514	aparc-a2009s_lh_volume_G-temporal-middle	Destrieux Atlas	Total body	-0.16	0.05	1.20E-03	1.59E-02
594	27719	aparc-a2009s_rh_volume_G-oc-temp-lat-fusifor	Destrieux Atlas	Total body	-0.17	0.05	1.20E-03	1.59E-02
1263	27636	aparc-a2009s_rh_thickness_G-front-inf-Opercular	Destrieux Atlas	Femoral neck	-0.25	0.08	1.30E-03	1.18E-01
1018	27623	aparc-a2009s_rh_area_S-temporal-sup	Destrieux Atlas	Heel	-0.12	0.04	1.30E-03	9.62E-02
339	26716	Brainstem_global_volume_Medulla	Brain Stem	Lumbar spine	0.15	0.05	1.30E-03	6.50E-03
135	25891	IDP_T1_FAST_ROIs_R_ventral_striatum	IDP T1:unilateral regions	Lumbar spine	-0.18	0.06	1.30E-03	3.61E-02
298	26675	ThalamNuclei_lh_volume_VAmc	Thalamus Nuclei	Total body	-0.12	0.04	1.30E-03	1.69E-02

976	27581	aparc-a2009s_rh_area_G-rectus	Destrieux Atlas	Total body	-0.15	0.05	1.30E-03	1.92E-02
250	26627	HippSubfield_lh_volume_presubiculum-body	Hippocampus Subfield	Total body	-0.13	0.04	1.30E-03	5.72E-02
86	25842	IDP_T1_FAST_ROIs_L_precun_cortex	IDP T1:unilateral regions	Femoral neck	0.19	0.06	1.40E-03	1.49E-02
31	25787	IDP_T1_FAST_ROIs_R_sup_front_gyrus	IDP T1:unilateral regions	Femoral neck	0.25	0.08	1.40E-03	1.49E-02
86	25842	IDP_T1_FAST_ROIs_L_precun_cortex	IDP T1:unilateral regions	Heel	0.12	0.04	1.40E-03	9.27E-03
426	27131	BA-exvivo_rh_volume_BA3a	Broadmann Atlas	Lumbar spine	-0.20	0.06	1.40E-03	3.92E-02
452	27219	aparc-DKTatlas_lh_volume_paracentral	Desikan Atlas	Total body	-0.15	0.05	1.40E-03	1.49E-02
534	27511	aparc-a2009s_lh_volume_G-temp-sup-Plan-polar	Destrieux Atlas	Total body	-0.16	0.05	1.40E-03	1.59E-02
91	25847	IDP_T1_FAST_ROIs_R_front_orb_cortex	IDP T1:unilateral regions	Femoral neck	0.20	0.06	1.50E-03	1.49E-02
717	26924	aparc-pial_lh_area_bankssts	Desikan Atlas	Total body	-0.18	0.06	1.50E-03	1.55E-02
1296	27669	aparc-a2009s_rh_thickness_S-central	Destrieux Atlas	Femoral neck	-0.25	0.08	1.60E-03	1.18E-01
91	25847	IDP_T1_FAST_ROIs_R_front_orb_cortex	IDP T1:unilateral regions	Heel	0.14	0.04	1.60E-03	9.67E-03
143	25899	IDP_T1_FAST_ROIs_R_cerebellum_VI	IDP T1:unilateral regions	Heel	-0.09	0.03	1.60E-03	9.67E-03
153	25909	IDP_T1_FAST_ROIs_L_cerebellum_VIIIa	IDP T1:unilateral regions	Heel	-0.12	0.04	1.60E-03	9.67E-03
531	27508	aparc-a2009s_lh_volume_G-subcallosal	Destrieux Atlas	Heel	-0.11	0.04	1.60E-03	1.18E-01
40	25796	IDP_T1_FAST_ROIs_L_temporal_pole	IDP T1:unilateral regions	Total body	0.15	0.05	1.60E-03	6.74E-03
704	26844	aparc-Desikan_rh_area_posteriorcingulate	Desikan Atlas	Total body	-0.15	0.05	1.60E-03	1.57E-02
193	26556	aseg_lh_volume_Cerebellum-White-Matter	aseg:unilateral regions	Heel	-0.08	0.02	1.70E-03	5.78E-02
545	27522	aparc-a2009s_lh_volume_S-cingul-Marginalis	Destrieux Atlas	Lumbar spine	-0.24	0.08	1.70E-03	9.18E-02
582	27707	aparc-a2009s_rh_volume_G-cingul-Post-dorsal	Destrieux Atlas	Lumbar spine	-0.27	0.09	1.70E-03	9.18E-02
45	25801	IDP_T1_FAST_ROIs_R_sup_temp_gyrus_post	IDP T1:unilateral regions	Total body	-0.14	0.05	1.70E-03	6.95E-03
1011	27616	aparc-a2009s_rh_area_S-pericallosal	Destrieux Atlas	Total body	-0.17	0.05	1.70E-03	2.05E-02
903	27360	aparc-a2009s_lh_area_G-subcallosal	Destrieux Atlas	Total body	-0.26	0.08	1.70E-03	2.05E-02
1317	27690	aparc-a2009s_rh_thickness_S-pericallosal	Destrieux Atlas	Total body	0.13	0.04	1.70E-03	2.52E-01
37	25793	IDP_T1_FAST_ROIs_R_inf_front_gyrus_parsop	IDP T1:unilateral regions	Heel	0.14	0.05	1.80E-03	9.78E-03
68	25824	IDP_T1_FAST_ROIs_L_latocc_cortex_sup	IDP T1:unilateral regions	Heel	0.15	0.05	1.80E-03	9.78E-03
5	25005	IDP_T1_SIENAX_grey_normalised_volume	IDP T1:global	Heel	0.09	0.03	1.80E-03	1.80E-02
91	25847	IDP_T1_FAST_ROIs_R_front_orb_cortex	IDP T1:unilateral regions	Total body	0.14	0.04	1.80E-03	7.15E-03
328	26705	ThalamNuclei_rh_volume_PuL	Thalamus Nuclei	Total body	-0.12	0.04	1.80E-03	1.87E-02
523	27500	aparc-a2009s_lh_volume_G-orbital	Destrieux Atlas	Total body	-0.16	0.05	1.80E-03	1.90E-02
920	27377	aparc-a2009s_lh_area_S-circular-insula-sup	Destrieux Atlas	Total body	-0.13	0.04	1.80E-03	2.05E-02
35	25791	IDP_T1_FAST_ROIs_R_inf_front_gyrus_parstri	IDP T1:unilateral regions	Heel	0.19	0.06	1.90E-03	9.78E-03
206	26583	aseg_rh_volume_Cortex	aseg:unilateral regions	Total body	-0.16	0.05	1.90E-03	9.23E-03

442	27209	aparc-DKTatlas_lh_volume_fusiform	Desikan Atlas	Total body	-0.17	0.05	1.90E-03	1.87E-02
560	27537	aparc-a2009s_lh_volume_S-oc-temp-med+Lingual	Destrieux Atlas	Lumbar spine	-0.24	0.08	2.00E-03	9.18E-02
1253	27626	aparc-a2009s_rh_thickness_G+S-occipital-inf	Destrieux Atlas	Lumbar spine	-0.22	0.07	2.00E-03	1.48E-01
129	25885	IDP_T1_FAST_ROIs_R_pallidum	IDP T1:unilateral regions	Femoral neck	-0.19	0.06	2.10E-03	1.88E-02
609	27734	aparc-a2009s_rh_volume_G-temp-sup-Plan-tempo	Destrieux Atlas	Total body	-0.16	0.05	2.10E-03	2.04E-02
162	25918	IDP_T1_FAST_ROIs_L_cerebellum_X	IDP T1:unilateral regions	Femoral neck	0.17	0.06	2.20E-03	1.88E-02
938	27395	aparc-a2009s_lh_area_S-postcentral	Destrieux Atlas	Femoral neck	0.23	0.08	2.20E-03	3.26E-01
128	25884	IDP_T1_FAST_ROIs_L_pallidum	IDP T1:unilateral regions	Heel	-0.13	0.04	2.20E-03	1.09E-02
219	26596	aseg_rh_volume_VentralDC	aseg:unilateral regions	Total body	-0.11	0.04	2.20E-03	9.35E-03
370	26815	aparc-Desikan_lh_volume_superiorfrontal	Desikan Atlas	Total body	-0.13	0.04	2.20E-03	2.01E-02
618	27743	aparc-a2009s_rh_volume_S-central	Destrieux Atlas	Total body	-0.14	0.05	2.20E-03	2.04E-02
784	27061	BA-exvivo_lh_area_BA3a	Broadmann Atlas	Total body	-0.15	0.05	2.20E-03	3.08E-02
147	25903	IDP_T1_FAST_ROIs_L_cerebellum_crus_II	IDP T1:unilateral regions	Femoral neck	-0.16	0.05	2.30E-03	1.88E-02
172	26521	aseg_global_volume_EstimatedTotalIntraCranial	aseg:global	Forearm	0.57	0.19	2.30E-03	2.53E-02
932	27389	aparc-a2009s_lh_area_S-oc-temp-med+Lingual	Destrieux Atlas	Lumbar spine	-0.23	0.08	2.40E-03	1.67E-01
675	26748	aparc-Desikan_lh_area_superiorfrontal	Desikan Atlas	Total body	-0.12	0.04	2.40E-03	2.24E-02
934	27391	aparc-a2009s_lh_area_S-orbital-med-olfact	Destrieux Atlas	Total body	-0.14	0.05	2.40E-03	2.54E-02
1201	27426	aparc-a2009s_lh_thickness_G-orbital	Destrieux Atlas	Femoral neck	-0.25	0.08	2.50E-03	1.23E-01
59	25815	IDP_T1_FAST_ROIs_R_postcent_gyrus	IDP T1:unilateral regions	Total body	0.15	0.05	2.50E-03	9.65E-03
56	25812	IDP_T1_FAST_ROIs_L_inf_temp_gyrus_tempocc	IDP T1:unilateral regions	Heel	0.11	0.04	2.70E-03	1.29E-02
54	25810	IDP_T1_FAST_ROIs_L_inf_temp_gyrus_post	IDP T1:unilateral regions	Lumbar spine	0.22	0.07	2.70E-03	6.16E-02
349	26794	aparc-Desikan_lh_volume_fusiform	Desikan Atlas	Total body	-0.17	0.06	2.70E-03	2.30E-02
902	27359	aparc-a2009s_lh_area_G-rectus	Destrieux Atlas	Total body	-0.18	0.06	2.70E-03	2.66E-02
63	25819	IDP_T1_FAST_ROIs_R_supramarg_gyrus_ant	IDP T1:unilateral regions	Heel	0.13	0.04	2.80E-03	1.30E-02
195	26558	aseg_lh_volume_Thalamus-Proper	aseg:unilateral regions	Total body	-0.11	0.04	2.80E-03	1.06E-02
184	26533	aseg_global_volume_CC-Central	aseg:global	Heel	-0.09	0.03	2.90E-03	2.01E-02
597	27722	aparc-a2009s_rh_volume_G-orbital	Destrieux Atlas	Lumbar spine	-0.21	0.07	2.90E-03	9.18E-02
732	26939	aparc-pial_lh_area_paracentral	Desikan Atlas	Total body	-0.14	0.05	2.90E-03	2.56E-02
447	27214	aparc-DKTatlas_lh_volume_lateralorbitofrontal	Desikan Atlas	Lumbar spine	-0.19	0.06	3.00E-03	1.09E-01
684	26824	aparc-Desikan_rh_area_caudalanteriorcingulate	Desikan Atlas	Total body	-0.17	0.06	3.00E-03	2.56E-02
40	25796	IDP_T1_FAST_ROIs_L_temporal_pole	IDP T1:unilateral regions	Lumbar spine	0.19	0.06	3.10E-03	6.16E-02
557	27534	aparc-a2009s_lh_volume_S-oc-sup+transversal	Destrieux Atlas	Lumbar spine	-0.25	0.08	3.10E-03	9.18E-02

646	27771	aparc-a2009s_rh_volume_S-temporal-sup	Destrieux Atlas	Total body	-0.16	0.06	3.20E-03	2.71E-02
695	26835	aparc-Desikan_rh_area_medialorbitofrontal	Desikan Atlas	Lumbar spine	-0.20	0.07	3.30E-03	2.54E-01
852	27247	aparc-DKTatlas_rh_area_medialorbitofrontal	Desikan Atlas	Lumbar spine	-0.20	0.07	3.30E-03	2.54E-01
60	25816	IDP_T1_FAST_ROIs_L_sup_parietal_lobule	IDP T1:unilateral regions	Total body	0.17	0.06	3.30E-03	1.24E-02
716	26923	aparc-pial_lh_area_TotalSurface	Desikan Atlas	Total body	-0.13	0.04	3.30E-03	2.56E-02
750	26957	aparc-pial_rh_area_bankssts	Desikan Atlas	Total body	-0.21	0.07	3.30E-03	2.56E-02
409	26922	aparc-Desikan_rh_volume_insula	Desikan Atlas	Total body	-0.13	0.04	3.30E-03	2.64E-02
581	27706	aparc-a2009s_rh_volume_G+S-cingul-Mid-Post	Destrieux Atlas	Total body	-0.14	0.05	3.30E-03	2.71E-02
34	25790	IDP_T1_FAST_ROIs_L_inf_front_gyrus_parstri	IDP T1:unilateral regions	Femoral neck	0.25	0.08	3.40E-03	2.63E-02
33	25789	IDP_T1_FAST_ROIs_R_mid_front_gyrus	IDP T1:unilateral regions	Heel	0.11	0.04	3.40E-03	1.49E-02
861	27256	aparc-DKTatlas_rh_area_posteriorcingulate	Desikan Atlas	Total body	-0.13	0.05	3.40E-03	2.56E-02
990	27595	aparc-a2009s_rh_area_S-central	Destrieux Atlas	Total body	-0.13	0.04	3.40E-03	3.15E-02
158	25914	IDP_T1_FAST_ROIs_R_cerebellum_VIIIb	IDP T1:unilateral regions	Heel	-0.10	0.03	3.50E-03	1.49E-02
41	25797	IDP_T1_FAST_ROIs_R_temporal_pole	IDP T1:unilateral regions	Heel	0.09	0.03	3.50E-03	1.49E-02
165	26514	aseg_global_volume_BrainSeg	aseg:global	Heel	-0.10	0.03	3.50E-03	2.01E-02
5	25005	IDP_T1_SIENAX_grey_normalised_volume	IDP T1:global	Femoral neck	0.18	0.06	3.60E-03	2.40E-02
1031	26766	aparc-Desikan_lh_thickness_lateralorbitofrontal	Desikan Atlas	Femoral neck	-0.23	0.08	3.60E-03	1.62E-01
607	27732	aparc-a2009s_rh_volume_G-temp-sup-Lateral	Destrieux Atlas	Femoral neck	-0.20	0.07	3.60E-03	5.33E-01
84	25840	IDP_T1_FAST_ROIs_L_cing_gyrus_post	IDP T1:unilateral regions	Total body	-0.14	0.05	3.60E-03	1.32E-02
337	26714	ThalamNuclei_lh_volume_Whole-thalamus	Thalamus Nuclei	Total body	-0.10	0.03	3.60E-03	2.54E-02
306	26683	ThalamNuclei_lh_volume_Pt	Thalamus Nuclei	Total body	-0.12	0.04	3.60E-03	2.54E-02
87	25843	IDP_T1_FAST_ROIs_R_precun_cortex	IDP T1:unilateral regions	Femoral neck	0.18	0.06	3.70E-03	2.71E-02
126	25882	IDP_T1_FAST_ROIs_L_putamen	IDP T1:unilateral regions	Heel	-0.10	0.04	3.70E-03	1.49E-02
145	25901	IDP_T1_FAST_ROIs_V_cerebellum_crus_I	IDP T1:unilateral regions	Heel	0.12	0.04	3.70E-03	1.49E-02
189	26552	aseg_lh_volume_Cortex	aseg:unilateral regions	Lumbar spine	-0.20	0.07	3.70E-03	6.29E-02
29	25785	IDP_T1_FAST_ROIs_R_insular_cortex	IDP T1:unilateral regions	Total body	-0.11	0.04	3.70E-03	1.32E-02
462	27229	aparc-DKTatlas_lh_volume_rostralmiddlefrontal	Desikan Atlas	Total body	-0.12	0.04	3.70E-03	2.79E-02
824	27157	aparc-DKTatlas_lh_area_paracentral	Desikan Atlas	Total body	-0.12	0.04	3.80E-03	2.76E-02
708	26848	aparc-Desikan_rh_area_rostralmiddlefrontal	Desikan Atlas	Lumbar spine	-0.16	0.06	3.90E-03	2.54E-01
293	26670	ThalamNuclei_lh_volume_CM	Thalamus Nuclei	Total body	-0.11	0.04	3.90E-03	2.54E-02
557	27534	aparc-a2009s_lh_volume_S-oc-sup+transversal	Destrieux Atlas	Total body	-0.16	0.06	3.90E-03	3.04E-02
33	25789	IDP_T1_FAST_ROIs_R_mid_front_gyrus	IDP T1:unilateral regions	Femoral neck	0.19	0.07	4.00E-03	2.78E-02
405	26918	aparc-Desikan_rh_volume_superiortemporal	Desikan Atlas	Femoral neck	-0.18	0.06	4.00E-03	5.12E-01

917	27374	aparc-a2009s_lh_area_S-cingul-Marginalis	Destrieux Atlas	Lumbar spine	-0.21	0.07	4.00E-03	1.67E-01
500	27477	aparc-a2009s_lh_volume_G+S-frontomargin	Destrieux Atlas	Heel	-0.09	0.03	4.10E-03	2.02E-01
460	27227	aparc-DKTatlas_lh_volume_precuneus	Desikan Atlas	Lumbar spine	-0.18	0.06	4.10E-03	1.09E-01
415	27092	BA-exvivo_lh_volume_BA4p	Broadmann Atlas	Total body	-0.16	0.06	4.10E-03	3.08E-02
37	25793	IDP_T1_FAST_ROIs_R_inf_front_gyrus_parsop	IDP T1:unilateral regions	Total body	0.19	0.07	4.20E-03	1.46E-02
819	27152	aparc-DKTatlas_lh_area_lateralorbitofrontal	Desikan Atlas	Total body	-0.12	0.04	4.20E-03	2.78E-02
822	27155	aparc-DKTatlas_lh_area_middletemporal	Desikan Atlas	Total body	-0.12	0.04	4.20E-03	2.78E-02
121	25877	IDP_T1_FAST_ROIs_R_occ_pole	IDP T1:unilateral regions	Lumbar spine	0.17	0.06	4.30E-03	7.47E-02
954	27559	aparc-a2009s_rh_area_G-cingul-Post-dorsal	Destrieux Atlas	Lumbar spine	-0.20	0.07	4.30E-03	1.67E-01
749	26956	aparc-pial_rh_area_TotalSurface	Desikan Atlas	Total body	-0.13	0.04	4.30E-03	2.78E-02
621	27746	aparc-a2009s_rh_volume_S-circular-insula-inf	Destrieux Atlas	Total body	-0.13	0.05	4.30E-03	3.18E-02
783	27060	BA-exvivo_lh_area_BA2	Broadmann Atlas	Femoral neck	0.20	0.07	4.40E-03	7.14E-02
778	26985	aparc-pial_rh_area_superiortemporal	Desikan Atlas	Total body	-0.13	0.05	4.40E-03	2.78E-02
429	27134	BA-exvivo_rh_volume_BA4p	Broadmann Atlas	Total body	-0.14	0.05	4.40E-03	3.08E-02
62	25818	IDP_T1_FAST_ROIs_L_supramarg_gyrus_ant	IDP T1:unilateral regions	Femoral neck	0.24	0.09	4.50E-03	2.98E-02
879	27336	aparc-a2009s_lh_area_G+S-cingul-Mid-Post	Destrieux Atlas	Lumbar spine	-0.20	0.07	4.50E-03	1.67E-01
154	25910	IDP_T1_FAST_ROIs_V_cerebellum_VIIIa	IDP T1:unilateral regions	Heel	-0.09	0.03	4.60E-03	1.71E-02
605	27730	aparc-a2009s_rh_volume_G-subcallosal	Destrieux Atlas	Lumbar spine	-0.21	0.07	4.60E-03	1.00E-01
367	26812	aparc-Desikan_lh_volume_precuneus	Desikan Atlas	Lumbar spine	-0.18	0.06	4.60E-03	1.09E-01
449	27216	aparc-DKTatlas_lh_volume_medialorbitofrontal	Desikan Atlas	Total body	-0.14	0.05	4.60E-03	3.17E-02
520	27497	aparc-a2009s_lh_volume_G-oc-temp-lat-fusifor	Destrieux Atlas	Total body	-0.15	0.05	4.60E-03	3.24E-02
156	25912	IDP_T1_FAST_ROIs_L_cerebellum_VIIIb	IDP T1:unilateral regions	Heel	-0.10	0.04	4.70E-03	1.71E-02
141	25897	IDP_T1_FAST_ROIs_L_cerebellum_VI	IDP T1:unilateral regions	Heel	-0.08	0.03	4.70E-03	1.71E-02
9	25009	IDP_T1_SIENAX_brain-normalised_volume	IDP T1:global	Heel	0.09	0.03	4.70E-03	2.75E-02
359	26804	aparc-Desikan_lh_volume_paracentral	Desikan Atlas	Total body	-0.14	0.05	4.70E-03	3.17E-02
100	25856	IDP_T1_FAST_ROIs_L_temp_fusif_cortex_post	IDP T1:unilateral regions	Heel	0.09	0.03	4.80E-03	1.71E-02
210	26587	aseg_rh_volume_Cerebellum-White-Matter	aseg:unilateral regions	Total body	-0.11	0.04	4.80E-03	1.63E-02
1018	27623	aparc-a2009s_rh_area_S-temporal-sup	Destrieux Atlas	Total body	-0.15	0.05	4.90E-03	4.27E-02
412	27089	BA-exvivo_lh_volume_BA3a	Broadmann Atlas	Lumbar spine	-0.20	0.07	5.00E-03	6.72E-02
745	26952	aparc-pial_lh_area_superiortemporal	Desikan Atlas	Total body	-0.13	0.04	5.00E-03	3.06E-02
782	27059	BA-exvivo_lh_area_BA1	Broadmann Atlas	Femoral neck	0.19	0.07	5.10E-03	7.14E-02
1071	26873	aparc-Desikan_rh_thickness_parsopercularis	Desikan Atlas	Femoral neck	-0.22	0.08	5.10E-03	1.62E-01
137	25893	IDP_T1_FAST_ROIs_L_cerebellum_I-IV	IDP T1:unilateral regions	Heel	-0.09	0.03	5.10E-03	1.77E-02

977	27582	aparc-a2009s_rh_area_G-subcallosal	Destrieux Atlas	Heel	-0.14	0.05	5.10E-03	2.52E-01
562	27539	aparc-a2009s_lh_volume_S-orbital-med-olfact	Destrieux Atlas	Lumbar spine	-0.22	0.08	5.10E-03	1.00E-01
237	26614	AmygNuclei_rh_volume_Central-nucleus	Amygdala Nuclei	Total body	-0.13	0.05	5.10E-03	1.02E-01
183	26532	aseg_global_volume_CC-Mid-Posterior	aseg:global	Heel	-0.10	0.03	5.20E-03	2.22E-02
151	25907	IDP_T1_FAST_ROIs_V_cerebellum_VIIb	IDP T1:unilateral regions	Total body	-0.10	0.04	5.20E-03	1.76E-02
548	27525	aparc-a2009s_lh_volume_S-circular-insula-sup	Destrieux Atlas	Total body	-0.13	0.05	5.20E-03	3.50E-02
398	26911	aparc-Desikan_rh_volume_posteriorcingulate	Desikan Atlas	Lumbar spine	-0.24	0.09	5.40E-03	1.09E-01
318	26695	ThalamNuclei_rh_volume_VLa	Thalamus Nuclei	Total body	-0.09	0.03	5.40E-03	3.12E-02
25	25025	IDP_T1_FIRST_brain_stem+4th_ventricle_volume	IDP T1:global	Heel	-0.09	0.03	5.50E-03	2.75E-02
862	27257	aparc-DKTatlas_rh_area_precentral	Desikan Atlas	Total body	-0.11	0.04	5.60E-03	3.29E-02
489	27318	aparc-DKTatlas_rh_volume_posteriorcingulate	Desikan Atlas	Total body	-0.15	0.05	5.60E-03	3.58E-02
535	27512	aparc-a2009s_lh_volume_G-temp-sup-Plan-tempo	Destrieux Atlas	Total body	-0.15	0.05	5.60E-03	3.60E-02
838	27171	aparc-DKTatlas_lh_area_supramarginal	Desikan Atlas	Femoral neck	0.16	0.06	5.70E-03	8.13E-01
697	26837	aparc-Desikan_rh_area_parahippocampal	Desikan Atlas	Total body	-0.12	0.04	5.70E-03	3.29E-02
929	27386	aparc-a2009s_lh_area_S-oc-sup+transversal	Destrieux Atlas	Total body	-0.13	0.05	5.70E-03	4.69E-02
166	26515	aseg_global_volume_BrainSegNotVent	aseg:global	Heel	-0.09	0.03	5.80E-03	2.22E-02
387	26900	aparc-Desikan_rh_volume_lateralorbitofrontal	Desikan Atlas	Lumbar spine	-0.19	0.07	5.80E-03	1.09E-01
1265	27638	aparc-a2009s_rh_thickness_G-front-inf-Triangul	Destrieux Atlas	Femoral neck	-0.20	0.07	5.90E-03	2.16E-01
507	27484	aparc-a2009s_lh_volume_G+S-cingul-Mid-Post	Destrieux Atlas	Lumbar spine	-0.23	0.08	5.90E-03	1.00E-01
478	27307	aparc-DKTatlas_rh_volume_lateralorbitofrontal	Desikan Atlas	Lumbar spine	-0.18	0.07	5.90E-03	1.09E-01
845	27240	aparc-DKTatlas_rh_area_fusiform	Desikan Atlas	Total body	-0.12	0.04	6.00E-03	3.33E-02
266	26643	HippSubfield_rh_volume_subiculum-body	Hippocampus Subfield	Total body	-0.12	0.04	6.00E-03	9.68E-02
90	25846	IDP_T1_FAST_ROIs_L_front_orb_cortex	IDP T1:unilateral regions	Femoral neck	0.18	0.07	6.10E-03	3.77E-02
35	25791	IDP_T1_FAST_ROIs_R_inf_front_gyrus_parstri	IDP T1:unilateral regions	Femoral neck	0.35	0.13	6.10E-03	3.77E-02
150	25906	IDP_T1_FAST_ROIs_L_cerebellum_VIIb	IDP T1:unilateral regions	Heel	-0.11	0.04	6.10E-03	2.07E-02
452	27219	aparc-DKTatlas_lh_volume_paracentral	Desikan Atlas	Lumbar spine	-0.20	0.07	6.20E-03	1.09E-01
814	27147	aparc-DKTatlas_lh_area_fusiform	Desikan Atlas	Total body	-0.14	0.05	6.20E-03	3.33E-02
683	26823	aparc-Desikan_rh_area_bankssts	Desikan Atlas	Total body	-0.14	0.05	6.20E-03	3.33E-02
500	27477	aparc-a2009s_lh_volume_G+S-frontomargin	Destrieux Atlas	Total body	-0.14	0.05	6.20E-03	3.82E-02
25	25025	IDP_T1_FIRST_brain_stem+4th_ventricle_volume	IDP T1:global	Total body	-0.11	0.04	6.20E-03	8.10E-02
96	25852	IDP_T1_FAST_ROIs_L_lingual_gyrus	IDP T1:unilateral regions	Total body	-0.14	0.05	6.30E-03	2.09E-02

481	27310	aparc-DKTatlas_rh_volume_middletemporal	Desikan Atlas	Total body	-0.13	0.05	6.30E-03	3.66E-02
134	25890	IDP_T1_FAST_ROIs_L_ventral_striatum	IDP T1:unilateral regions	Heel	-0.07	0.03	6.40E-03	2.10E-02
211	26588	aseg_rh_volume_Cerebellum-Cortex	aseg:unilateral regions	Heel	-0.10	0.04	6.40E-03	1.09E-01
157	25913	IDP_T1_FAST_ROIs_V_cerebellum_VIIIb	IDP T1:unilateral regions	Heel	-0.07	0.03	6.50E-03	2.10E-02
605	27730	aparc-a2009s_rh_volume_G-subcallosal	Destrieux Atlas	Heel	-0.10	0.04	6.60E-03	2.28E-01
167	26516	aseg_global_volume_BrainSegNotVentSurf	aseg:global	Lumbar spine	-0.18	0.07	6.60E-03	2.30E-02
56	25812	IDP_T1_FAST_ROIs_L_inf_temp_gyrus_tempocc	IDP T1:unilateral regions	Lumbar spine	0.18	0.07	6.60E-03	9.20E-02
100	25856	IDP_T1_FAST_ROIs_L_temp_fusif_cortex_post	IDP T1:unilateral regions	Total body	0.12	0.04	6.60E-03	2.13E-02
664	26737	aparc-Desikan_lh_area_paracentral	Desikan Atlas	Total body	-0.11	0.04	6.60E-03	3.40E-02
37	25793	IDP_T1_FAST_ROIs_R_inf_front_gyrus_parsop	IDP T1:unilateral regions	Femoral neck	0.27	0.10	6.80E-03	3.85E-02
129	25885	IDP_T1_FAST_ROIs_R_pallidum	IDP T1:unilateral regions	Heel	-0.11	0.04	6.80E-03	2.12E-02
152	25908	IDP_T1_FAST_ROIs_R_cerebellum_VIIb	IDP T1:unilateral regions	Heel	-0.11	0.04	6.80E-03	2.12E-02
906	27363	aparc-a2009s_lh_area_G-temp-sup-Plan-polar	Destrieux Atlas	Heel	-0.15	0.06	6.80E-03	2.52E-01
171	26520	aseg_global_volume_SupraTentorialNotVent	aseg:global	Lumbar spine	-0.17	0.06	6.80E-03	2.30E-02
451	27218	aparc-DKTatlas_lh_volume_parahippocampal	Desikan Atlas	Total body	-0.10	0.04	6.80E-03	3.66E-02
126	25882	IDP_T1_FAST_ROIs_L_putamen	IDP T1:unilateral regions	Total body	-0.12	0.04	6.90E-03	2.16E-02
79	25835	IDP_T1_FAST_ROIs_R_subcallosal_cortex	IDP T1:unilateral regions	Total body	-0.10	0.04	6.90E-03	2.16E-02
833	27166	aparc-DKTatlas_lh_area_rostralanteriorcingulate	Desikan Atlas	Total body	-0.11	0.04	6.90E-03	3.46E-02
365	26810	aparc-Desikan_lh_volume_posteriorcingulate	Desikan Atlas	Total body	-0.16	0.06	6.90E-03	3.66E-02
138	25894	IDP_T1_FAST_ROIs_R_cerebellum_I-IV	IDP T1:unilateral regions	Femoral neck	-0.15	0.05	7.00E-03	3.85E-02
170	26519	aseg_global_volume_SupraTentorial	aseg:global	Lumbar spine	-0.17	0.06	7.00E-03	2.30E-02
178	26527	aseg_global_volume_CSF	aseg:global	Total body	-0.11	0.04	7.00E-03	1.61E-02
458	27225	aparc-DKTatlas_lh_volume_posteriorcingulate	Desikan Atlas	Total body	-0.15	0.06	7.00E-03	3.66E-02
377	26890	aparc-Desikan_rh_volume_bankssts	Desikan Atlas	Total body	-0.15	0.06	7.00E-03	3.66E-02
32	25788	IDP_T1_FAST_ROIs_L_mid_front_gyrus	IDP T1:unilateral regions	Heel	0.09	0.04	7.10E-03	2.15E-02
688	26828	aparc-Desikan_rh_area_fusiform	Desikan Atlas	Total body	-0.11	0.04	7.10E-03	3.46E-02
251	26628	HippSubfield_lh_volume_parasubiculum	Hippocampus Subfield	Total body	-0.14	0.05	7.10E-03	9.68E-02
59	25815	IDP_T1_FAST_ROIs_R_postcent_gyrus	IDP T1:unilateral regions	Femoral neck	0.20	0.07	7.20E-03	3.85E-02
429	27134	BA-exvivo_rh_volume_BA4p	Broadmann Atlas	Lumbar spine	-0.18	0.07	7.20E-03	6.72E-02
628	27753	aparc-a2009s_rh_volume_S-interm-prim-Jensen	Destrieux Atlas	Total body	-0.24	0.09	7.20E-03	4.16E-02
1215	27440	aparc-a2009s_lh_thickness_G-temporal-middle	Destrieux Atlas	Femoral neck	-0.18	0.07	7.30E-03	2.16E-01
654	26727	aparc-Desikan_lh_area_fusiform	Desikan Atlas	Total body	-0.13	0.05	7.30E-03	3.46E-02

607	27732	aparc-a2009s_rh_volume_G-temp-sup-Lateral	Destrieux Atlas	Total body	-0.13	0.05	7.30E-03	4.16E-02
1038	26773	aparc-Desikan_lh_thickness_parsorbitalis	Desikan Atlas	Femoral neck	-0.23	0.09	7.50E-03	1.62E-01
402	26915	aparc-Desikan_rh_volume_rostralmiddlefrontal	Desikan Atlas	Lumbar spine	-0.17	0.06	7.50E-03	1.09E-01
864	27259	aparc-DKTatlas_rh_area_rostralanteriorcingulate	Desikan Atlas	Total body	-0.14	0.05	7.50E-03	3.46E-02
109	25865	IDP_T1_FAST_ROIs_R_cent_operc_cortex	IDP T1:unilateral regions	Femoral neck	0.17	0.06	7.60E-03	3.91E-02
93	25849	IDP_T1_FAST_ROIs_R_parahipp_gyrus_ant	IDP T1:unilateral regions	Heel	0.10	0.04	7.60E-03	2.25E-02
33	25789	IDP_T1_FAST_ROIs_R_mid_front_gyrus	IDP T1:unilateral regions	Lumbar spine	0.17	0.06	7.60E-03	9.20E-02
823	27156	aparc-DKTatlas_lh_area_parahippocampal	Desikan Atlas	Lumbar spine	-0.18	0.07	7.60E-03	2.54E-01
705	26845	aparc-Desikan_rh_area_precentral	Desikan Atlas	Total body	-0.10	0.04	7.60E-03	3.46E-02
1000	27605	aparc-a2009s_rh_area_S-interm-prim-Jensen	Destrieux Atlas	Total body	-0.21	0.08	7.60E-03	5.71E-02
534	27511	aparc-a2009s_lh_volume_G-temp-sup-Plan-polar	Destrieux Atlas	Heel	-0.11	0.04	7.70E-03	2.28E-01
354	26799	aparc-Desikan_lh_volume_lateralorbitofrontal	Desikan Atlas	Total body	-0.13	0.05	7.80E-03	3.80E-02
619	27744	aparc-a2009s_rh_volume_S-cingul-Marginalis	Destrieux Atlas	Total body	-0.13	0.05	7.80E-03	4.28E-02
841	27236	aparc-DKTatlas_rh_area_caudalanteriorcingulate	Desikan Atlas	Total body	-0.15	0.06	7.90E-03	3.52E-02
90	25846	IDP_T1_FAST_ROIs_L_front_orb_cortex	IDP T1:unilateral regions	Lumbar spine	0.15	0.06	8.00E-03	9.20E-02
865	27260	aparc-DKTatlas_rh_area_rostralmiddlefrontal	Desikan Atlas	Lumbar spine	-0.15	0.06	8.00E-03	2.54E-01
971	27576	aparc-a2009s_rh_area_G-pariet-inf-Supramar	Destrieux Atlas	Total body	-0.14	0.05	8.00E-03	5.71E-02
500	27477	aparc-a2009s_lh_volume_G+S-frontomargin	Destrieux Atlas	Lumbar spine	-0.21	0.08	8.10E-03	1.00E-01
400	26913	aparc-Desikan_rh_volume_precuneus	Desikan Atlas	Total body	-0.12	0.05	8.10E-03	3.80E-02
966	27571	aparc-a2009s_rh_area_G-oc-temp-lat-fusifor	Destrieux Atlas	Total body	-0.13	0.05	8.10E-03	5.71E-02
1	25001	IDP_T1_SIENAX_peripheral_grey_normalised_volume	IDP T1:global	Total body	0.13	0.05	8.10E-03	8.10E-02
594	27719	aparc-a2009s_rh_volume_G-oc-temp-lat-fusifor	Destrieux Atlas	Lumbar spine	-0.19	0.07	8.20E-03	1.00E-01
41	25797	IDP_T1_FAST_ROIs_R_temporal_pole	IDP T1:unilateral regions	Total body	0.13	0.05	8.20E-03	2.48E-02
678	26751	aparc-Desikan_lh_area_supramarginal	Desikan Atlas	Femoral neck	0.15	0.06	8.30E-03	8.13E-01
160	25916	IDP_T1_FAST_ROIs_V_cerebellum_IX	IDP T1:unilateral regions	Femoral neck	-0.15	0.06	8.40E-03	3.96E-02
71	25827	IDP_T1_FAST_ROIs_R_latocc_cortex_inf	IDP T1:unilateral regions	Heel	0.14	0.05	8.40E-03	2.39E-02
58	25814	IDP_T1_FAST_ROIs_L_postcent_gyrus	IDP T1:unilateral regions	Heel	0.10	0.04	8.40E-03	2.39E-02
369	26814	aparc-Desikan_lh_volume_rostralmiddlefrontal	Desikan Atlas	Total body	-0.11	0.04	8.40E-03	3.80E-02
134	25890	IDP_T1_FAST_ROIs_L_ventral_striatum	IDP T1:unilateral regions	Lumbar spine	-0.15	0.06	8.50E-03	9.20E-02
767	26974	aparc-pial_rh_area_parsorbitalis	Desikan Atlas	Lumbar spine	-0.18	0.07	8.50E-03	2.54E-01
961	27566	aparc-a2009s_rh_area_G-front-sup	Destrieux Atlas	Total body	-0.12	0.04	8.50E-03	5.72E-02
58	25814	IDP_T1_FAST_ROIs_L_postcent_gyrus	IDP T1:unilateral regions	Femoral neck	0.20	0.08	8.60E-03	3.96E-02

136	25892	IDP_T1_FAST_ROIs_brain_stem	IDP T1:unilateral regions	Heel	0.08	0.03	8.60E-03	2.39E-02
97	25853	IDP_T1_FAST_ROIs_R_lingual_gyrus	IDP T1:unilateral regions	Lumbar spine	-0.17	0.06	8.60E-03	9.20E-02
390	26903	aparc-Desikan_rh_volume_middletemporal	Desikan Atlas	Total body	-0.13	0.05	8.60E-03	3.80E-02
136	25892	IDP_T1_FAST_ROIs_brain_stem	IDP T1:unilateral regions	Femoral neck	0.15	0.06	8.70E-03	3.96E-02
159	25915	IDP_T1_FAST_ROIs_L_cerebellum_IX	IDP T1:unilateral regions	Femoral neck	-0.14	0.05	8.70E-03	3.96E-02
45	25801	IDP_T1_FAST_ROIs_R_sup_temp_gyrus_post	IDP T1:unilateral regions	Forearm	-0.43	0.16	8.70E-03	7.65E-01
489	27318	aparc-DKTatlas_rh_volume_posteriorcingulate	Desikan Atlas	Forearm	-0.52	0.20	8.70E-03	6.14E-01
188	26537	aseg_global_volume-ratio_MaskVol-to-eTIV	aseg:global	Heel	-0.15	0.06	8.70E-03	2.67E-02
614	27739	aparc-a2009s_rh_volume_Lat-Fis-post	Destrieux Atlas	Total body	-0.12	0.04	8.70E-03	4.59E-02
968	27573	aparc-a2009s_rh_area_G-oc-temp-med-Parahip	Destrieux Atlas	Heel	-0.09	0.04	8.80E-03	2.60E-01
523	27500	aparc-a2009s_lh_volume_G-orbital	Destrieux Atlas	Lumbar spine	-0.17	0.07	8.80E-03	1.00E-01
634	27759	aparc-a2009s_rh_volume_S-oc-temp-med+Lingual	Destrieux Atlas	Lumbar spine	-0.18	0.07	8.90E-03	1.00E-01
389	26902	aparc-Desikan_rh_volume_medialorbitofrontal	Desikan Atlas	Lumbar spine	-0.19	0.07	8.90E-03	1.09E-01
376	26821	aparc-Desikan_lh_volume_insula	Desikan Atlas	Total body	-0.11	0.04	8.90E-03	3.80E-02
762	26969	aparc-pial_rh_area_medialorbitofrontal	Desikan Atlas	Total body	-0.13	0.05	9.00E-03	3.84E-02
540	27517	aparc-a2009s_lh_volume_Lat-Fis-post	Destrieux Atlas	Total body	-0.11	0.04	9.00E-03	4.59E-02
114	25870	IDP_T1_FAST_ROIs_L_heschl_gyrus	IDP T1:unilateral regions	Heel	0.08	0.03	9.10E-03	2.48E-02
575	27700	aparc-a2009s_rh_volume_G+S-occipital-inf	Destrieux Atlas	Lumbar spine	-0.20	0.07	9.10E-03	1.00E-01
835	27168	aparc-DKTatlas_lh_area_superiorfrontal	Desikan Atlas	Total body	-0.11	0.04	9.10E-03	3.84E-02
715	26855	aparc-Desikan_rh_area_insula	Desikan Atlas	Heel	-0.11	0.04	9.20E-03	4.09E-01
165	26514	aseg_global_volume_BrainSeg	aseg:global	Lumbar spine	-0.17	0.06	9.20E-03	2.61E-02
1235	27460	aparc-a2009s_lh_thickness_S-oc-sup+transversal	Destrieux Atlas	Lumbar spine	-0.19	0.07	9.20E-03	3.31E-01
879	27336	aparc-a2009s_lh_area_G+S-cingul-Mid-Post	Destrieux Atlas	Forearm	-0.46	0.18	9.30E-03	7.88E-01
167	26516	aseg_global_volume_BrainSegNotVentSurf	aseg:global	Heel	-0.08	0.03	9.30E-03	2.67E-02
683	26823	aparc-Desikan_rh_area_bankssts	Desikan Atlas	Heel	-0.09	0.03	9.40E-03	4.09E-01
530	27507	aparc-a2009s_lh_volume_G-rectus	Destrieux Atlas	Lumbar spine	-0.21	0.08	9.50E-03	1.00E-01
681	26754	aparc-Desikan_lh_area_insula	Desikan Atlas	Total body	-0.12	0.04	9.50E-03	3.84E-02
712	26852	aparc-Desikan_rh_area_supramarginal	Desikan Atlas	Total body	-0.14	0.05	9.50E-03	3.84E-02
358	26803	aparc-Desikan_lh_volume_parahippocampal	Desikan Atlas	Total body	-0.10	0.04	9.50E-03	3.92E-02
398	26911	aparc-Desikan_rh_volume_posteriorcingulate	Desikan Atlas	Forearm	-0.51	0.20	9.60E-03	6.14E-01
7	25007	IDP_T1_SIENAX_white_normalised_volume	IDP T1:global	Femoral neck	0.16	0.06	9.70E-03	4.85E-02
869	27264	aparc-DKTatlas_rh_area_supramarginal	Desikan Atlas	Total body	-0.14	0.05	9.70E-03	3.84E-02

740	26947	aparc-pial_lh_area_precuneus	Desikan Atlas	Lumbar spine	-0.16	0.06	9.80E-03	2.54E-01
27	25783	IDP_T1_FAST_ROIs_R_frontal_pole	IDP T1:unilateral regions	Heel	0.08	0.03	9.90E-03	2.65E-02
853	27248	aparc-DKTatlas_rh_area_middletemporal	Desikan Atlas	Total body	-0.11	0.04	9.90E-03	3.84E-02
473	27302	aparc-DKTatlas_rh_volume_fusiform	Desikan Atlas	Total body	-0.12	0.05	9.90E-03	3.96E-02
1295	27668	aparc-a2009s_rh_thickness_S-calcarine	Destrieux Atlas	Heel	0.11	0.04	1.00E-02	9.59E-01
730	26937	aparc-pial_lh_area_middletemporal	Desikan Atlas	Total body	-0.11	0.04	1.00E-02	3.84E-02
166	26515	aseg_global_volume_BrainSegNotVent	aseg:global	Lumbar spine	-0.17	0.07	1.02E-02	2.61E-02
1216	27441	aparc-a2009s_lh_thickness_Lat-Fis-ant-Horizont	Destrieux Atlas	Lumbar spine	0.21	0.08	1.03E-02	3.31E-01
764	26971	aparc-pial_rh_area_parahippocampal	Desikan Atlas	Total body	-0.11	0.04	1.03E-02	3.88E-02
551	27528	aparc-a2009s_lh_volume_S-front-inf	Destrieux Atlas	Total body	-0.12	0.05	1.03E-02	5.08E-02
449	27216	aparc-DKTatlas_lh_volume_medialorbitofrontal	Desikan Atlas	Lumbar spine	-0.17	0.07	1.04E-02	1.09E-01
248	26625	HippSubfield_lh_volume_presubiculum-head	Hippocampus Subfield	Total body	-0.12	0.05	1.04E-02	9.68E-02
194	26557	aseg_lh_volume_Cerebellum-Cortex	aseg:unilateral regions	Heel	-0.09	0.03	1.05E-02	1.19E-01
417	27094	BA-exvivo_lh_volume_BA44	Broadmann Atlas	Total body	-0.13	0.05	1.05E-02	4.95E-02
60	25816	IDP_T1_FAST_ROIs_L_sup_parietal_lobule	IDP T1:unilateral regions	Femoral neck	0.23	0.09	1.06E-02	4.60E-02
1244	27469	aparc-a2009s_lh_thickness_S-postcentral	Destrieux Atlas	Femoral neck	-0.18	0.07	1.06E-02	2.61E-01
649	26722	aparc-Desikan_lh_area_bankssts	Desikan Atlas	Heel	-0.09	0.03	1.06E-02	4.09E-01
416	27093	BA-exvivo_lh_volume_BA6	Broadmann Atlas	Total body	-0.11	0.04	1.06E-02	4.95E-02
953	27558	aparc-a2009s_rh_area_G+S-cingul-Mid-Post	Destrieux Atlas	Total body	-0.11	0.04	1.06E-02	6.34E-02
496	27325	aparc-DKTatlas_rh_volume_superiortemporal	Desikan Atlas	Femoral neck	-0.17	0.06	1.07E-02	6.85E-01
405	26918	aparc-Desikan_rh_volume_superiortemporal	Desikan Atlas	Lumbar spine	-0.17	0.07	1.07E-02	1.09E-01
122	25878	IDP_T1_FAST_ROIs_L_thalamus	IDP T1:unilateral regions	Total body	-0.10	0.04	1.08E-02	3.19E-02
560	27537	aparc-a2009s_lh_volume_S-oc-temp-med+Lingual	Destrieux Atlas	Total body	-0.14	0.06	1.10E-02	5.25E-02
324	26701	ThalamNuclei_rh_volume_VA	Thalamus Nuclei	Total body	-0.09	0.03	1.10E-02	5.72E-02
786	27063	BA-exvivo_lh_area_BA4a	Broadmann Atlas	Total body	-0.11	0.04	1.12E-02	6.19E-02
764	26971	aparc-pial_rh_area_parahippocampal	Desikan Atlas	Heel	-0.08	0.03	1.14E-02	4.09E-01
592	27717	aparc-a2009s_rh_volume_G-occipital-middle	Destrieux Atlas	Lumbar spine	-0.21	0.08	1.15E-02	1.06E-01
775	26982	aparc-pial_rh_area_rostralmiddlefrontal	Desikan Atlas	Lumbar spine	-0.15	0.06	1.15E-02	2.54E-01
202	26565	aseg_lh_volume_VentralDC	aseg:unilateral regions	Total body	-0.10	0.04	1.15E-02	3.55E-02
878	27335	aparc-a2009s_lh_area_G+S-cingul-Mid-Ant	Destrieux Atlas	Total body	-0.13	0.05	1.15E-02	6.34E-02
594	27719	aparc-a2009s_rh_volume_G-oc-temp-lat-fusifor	Destrieux Atlas	Femoral neck	-0.20	0.08	1.16E-02	5.70E-01
830	27163	aparc-DKTatlas_lh_area_posteriorcingulate	Desikan Atlas	Total body	-0.13	0.05	1.16E-02	4.29E-02
32	25788	IDP_T1_FAST_ROIs_L_mid_front_gyrus	IDP T1:unilateral regions	Femoral neck	0.17	0.07	1.17E-02	4.93E-02

1006	27611	aparc-a2009s_rh_area_S-oc-temp-med+Lingual	Destrieux Atlas	Lumbar spine	-0.18	0.07	1.18E-02	2.57E-01
625	27750	aparc-a2009s_rh_volume_S-front-inf	Destrieux Atlas	Lumbar spine	-0.19	0.08	1.19E-02	1.06E-01
483	27312	aparc-DKTatlas_rh_volume_paracentral	Desikan Atlas	Lumbar spine	-0.18	0.07	1.19E-02	1.09E-01
872	27329	aparc-a2009s_lh_area_G+S-frontomargin	Destrieux Atlas	Lumbar spine	-0.20	0.08	1.19E-02	2.57E-01
382	26895	aparc-Desikan_rh_volume_fusiform	Desikan Atlas	Total body	-0.12	0.05	1.19E-02	4.59E-02
799	27104	BA-exvivo_rh_area_BA3b	Broadmann Atlas	Total body	-0.11	0.04	1.19E-02	6.19E-02
1008	27613	aparc-a2009s_rh_area_S-orbital-med-olfact	Destrieux Atlas	Total body	-0.11	0.05	1.20E-02	6.34E-02
696	26836	aparc-Desikan_rh_area_middletemporal	Desikan Atlas	Total body	-0.11	0.04	1.21E-02	4.39E-02
622	27747	aparc-a2009s_rh_volume_S-circular-insula-sup	Destrieux Atlas	Total body	-0.13	0.05	1.21E-02	5.60E-02
916	27373	aparc-a2009s_lh_area_S-central	Destrieux Atlas	Total body	-0.12	0.05	1.21E-02	6.34E-02
618	27743	aparc-a2009s_rh_volume_S-central	Destrieux Atlas	Lumbar spine	-0.16	0.06	1.22E-02	1.06E-01
473	27302	aparc-DKTatlas_rh_volume_fusiform	Desikan Atlas	Lumbar spine	-0.16	0.06	1.22E-02	1.09E-01
36	25792	IDP_T1_FAST_ROIs_L_inf_front_gyrus_parsop	IDP T1:unilateral regions	Lumbar spine	0.23	0.09	1.22E-02	1.18E-01
128	25884	IDP_T1_FAST_ROIs_L_pallidum	IDP T1:unilateral regions	Total body	-0.12	0.05	1.22E-02	3.53E-02
491	27320	aparc-DKTatlas_rh_volume_precuneus	Desikan Atlas	Total body	-0.11	0.05	1.22E-02	4.59E-02
45	25801	IDP_T1_FAST_ROIs_R_sup_temp_gyrus_post	IDP T1:unilateral regions	Femoral neck	-0.16	0.06	1.23E-02	5.03E-02
1241	27466	aparc-a2009s_lh_thickness_S-orbital-H-Shaped	Destrieux Atlas	Lumbar spine	-0.19	0.08	1.23E-02	3.31E-01
1233	27458	aparc-a2009s_lh_thickness_S-intrapariet+P-trans	Destrieux Atlas	Lumbar spine	-0.17	0.07	1.23E-02	3.31E-01
1210	27435	aparc-a2009s_lh_thickness_G-temp-sup-G-T-transv	Destrieux Atlas	Femoral neck	-0.17	0.07	1.24E-02	2.62E-01
972	27577	aparc-a2009s_rh_area_G-parietal-sup	Destrieux Atlas	Femoral neck	0.19	0.08	1.24E-02	5.59E-01
934	27391	aparc-a2009s_lh_area_S-orbital-med-olfact	Destrieux Atlas	Lumbar spine	-0.16	0.06	1.24E-02	2.57E-01
272	26649	HippSubfield_rh_volume_presubiculum-body	Hippocampus Subfield	Total body	-0.10	0.04	1.24E-02	9.68E-02
608	27733	aparc-a2009s_rh_volume_G-temp-sup-Plan-polar	Destrieux Atlas	Total body	-0.14	0.06	1.25E-02	5.61E-02
703	26843	aparc-Desikan_rh_area_postcentral	Desikan Atlas	Total body	-0.12	0.05	1.26E-02	4.49E-02
458	27225	aparc-DKTatlas_lh_volume_posteriorcingulate	Desikan Atlas	Lumbar spine	-0.21	0.09	1.27E-02	1.09E-01
359	26804	aparc-Desikan_lh_volume_paracentral	Desikan Atlas	Lumbar spine	-0.19	0.07	1.27E-02	1.09E-01
126	25882	IDP_T1_FAST_ROIs_L_putamen	IDP T1:unilateral regions	Lumbar spine	-0.14	0.06	1.27E-02	1.18E-01
322	26699	ThalamNuclei_rh_volume_VAmc	Thalamus Nuclei	Total body	-0.09	0.04	1.27E-02	6.00E-02
909	27366	aparc-a2009s_lh_area_G-temporal-middle	Destrieux Atlas	Total body	-0.11	0.04	1.28E-02	6.34E-02
1006	27611	aparc-a2009s_rh_area_S-oc-temp-med+Lingual	Destrieux Atlas	Total body	-0.11	0.04	1.28E-02	6.34E-02
116	25872	IDP_T1_FAST_ROIs_L_planum_temporale	IDP T1:unilateral regions	Femoral neck	0.16	0.06	1.29E-02	5.12E-02
1065	26867	aparc-Desikan_rh_thickness_lateralorbitofrontal	Desikan Atlas	Femoral neck	-0.20	0.08	1.29E-02	1.62E-01

897	27354	aparc-a2009s_lh_area_G-pariet-inf-Supramar	Destrieux Atlas	Femoral neck	0.14	0.06	1.29E-02	5.59E-01
426	27131	BA-exvivo_rh_volume_BA3a	Broadmann Atlas	Heel	-0.10	0.04	1.29E-02	3.13E-01
356	26801	aparc-Desikan_lh_volume_medialorbitofrontal	Desikan Atlas	Total body	-0.13	0.05	1.29E-02	4.62E-02
901	27358	aparc-a2009s_lh_area_G-precuneus	Destrieux Atlas	Total body	-0.09	0.04	1.29E-02	6.34E-02
298	26675	ThalamNuclei_lh_volume_VAmc	Thalamus Nuclei	Heel	-0.07	0.03	1.30E-02	4.42E-01
153	25909	IDP_T1_FAST_ROIs_L_cerebellum_VIIIa	IDP T1:unilateral regions	Total body	-0.10	0.04	1.30E-02	3.69E-02
352	26797	aparc-Desikan_lh_volume_isthmuscingulate	Desikan Atlas	Total body	-0.11	0.04	1.30E-02	4.62E-02
979	27584	aparc-a2009s_rh_area_G-temp-sup-Lateral	Destrieux Atlas	Total body	-0.13	0.05	1.32E-02	6.34E-02
243	26620	HippSubfield_lh_volume_Hippocampal-tail	Hippocampus Subfield	Total body	-0.10	0.04	1.32E-02	9.68E-02
771	26978	aparc-pial_rh_area_posteriorcingulate	Desikan Atlas	Forearm	-0.46	0.18	1.34E-02	7.96E-01
342	26719	Brainstem_global_volume_Midbrain	Brain Stem	Heel	-0.08	0.03	1.34E-02	6.70E-02
441	27208	aparc-DKTatlas_lh_volume_entorhinal	Desikan Atlas	Total body	-0.12	0.05	1.35E-02	4.67E-02
239	26616	AmygNuclei_rh_volume_Cortical-nucleus	Amygdala Nuclei	Total body	-0.11	0.05	1.35E-02	1.09E-01
917	27374	aparc-a2009s_lh_area_S-cingul-Marginalis	Destrieux Atlas	Total body	-0.12	0.05	1.36E-02	6.34E-02
781	26988	aparc-pial_rh_area_transversetemporal	Desikan Atlas	Total body	-0.11	0.04	1.37E-02	4.75E-02
986	27591	aparc-a2009s_rh_area_Lat-Fis-post	Destrieux Atlas	Total body	-0.11	0.04	1.37E-02	6.34E-02
743	26950	aparc-pial_lh_area_superiorfrontal	Desikan Atlas	Total body	-0.11	0.04	1.38E-02	4.75E-02
977	27582	aparc-a2009s_rh_area_G-subcallosal	Destrieux Atlas	Lumbar spine	-0.27	0.11	1.39E-02	2.57E-01
221	26598	aseg_rh_volume_choroid-plexus	aseg:unilateral regions	Total body	-0.10	0.04	1.41E-02	4.00E-02
408	26921	aparc-Desikan_rh_volume_transversetemporal	Desikan Atlas	Total body	-0.12	0.05	1.41E-02	4.75E-02
670	26743	aparc-Desikan_lh_area_posteriorcingulate	Desikan Atlas	Total body	-0.13	0.05	1.41E-02	4.76E-02
854	27249	aparc-DKTatlas_rh_area_parahippocampal	Desikan Atlas	Heel	-0.09	0.04	1.42E-02	4.09E-01
507	27484	aparc-a2009s_lh_volume_G+S-cingul-Mid-Post	Destrieux Atlas	Total body	-0.13	0.05	1.42E-02	6.18E-02
801	27106	BA-exvivo_rh_area_BA4p	Broadmann Atlas	Total body	-0.11	0.04	1.43E-02	6.19E-02
85	25841	IDP_T1_FAST_ROIs_R_cing_gyrus_post	IDP T1:unilateral regions	Total body	-0.13	0.05	1.44E-02	4.00E-02
718	26925	aparc-pial_lh_area_caudalanteriorcingulate	Desikan Atlas	Heel	-0.11	0.05	1.46E-02	4.09E-01
1075	26877	aparc-Desikan_rh_thickness_postcentral	Desikan Atlas	Femoral neck	-0.18	0.07	1.48E-02	1.62E-01
850	27245	aparc-DKTatlas_rh_area_lateralorbitofrontal	Desikan Atlas	Total body	-0.10	0.04	1.48E-02	4.92E-02
3	25003	IDP_T1_SIENAX_CSF_normalised_volume	IDP T1:global	Total body	-0.09	0.04	1.48E-02	9.87E-02
462	27229	aparc-DKTatlas_lh_volume_rostralmiddlefrontal	Desikan Atlas	Lumbar spine	-0.15	0.06	1.50E-02	1.09E-01
1012	27617	aparc-a2009s_rh_area_S-postcentral	Destrieux Atlas	Femoral neck	0.19	0.08	1.51E-02	5.59E-01
474	27303	aparc-DKTatlas_rh_volume_inferiorparietal	Desikan Atlas	Lumbar spine	-0.16	0.07	1.51E-02	1.09E-01
493	27322	aparc-DKTatlas_rh_volume_rostralmiddlefrontal	Desikan Atlas	Lumbar spine	-0.16	0.07	1.51E-02	1.09E-01

508	27485	aparc-a2009s_lh_volume_G-cingul-Post-dorsal	Destrieux Atlas	Lumbar spine	-0.17	0.07	1.51E-02	1.21E-01
516	27493	aparc-a2009s_lh_volume_G-Ins-lg+S-cent-ins	Destrieux Atlas	Lumbar spine	-0.19	0.08	1.51E-02	1.21E-01
1074	26876	aparc-Desikan_rh_thickness_pericalcarine	Desikan Atlas	Heel	0.11	0.04	1.52E-02	7.41E-01
1073	26875	aparc-Desikan_rh_thickness_parstriangularis	Desikan Atlas	Femoral neck	-0.16	0.07	1.53E-02	1.62E-01
135	25891	IDP_T1_FAST_ROIs_R_ventral_striatum	IDP T1:unilateral regions	Femoral neck	-0.15	0.06	1.55E-02	5.98E-02
114	25870	IDP_T1_FAST_ROIs_L_heschl_gyrus	IDP T1:unilateral regions	Total body	0.11	0.04	1.55E-02	4.22E-02
406	26919	aparc-Desikan_rh_volume_supramarginal	Desikan Atlas	Total body	-0.14	0.06	1.57E-02	5.06E-02
463	27230	aparc-DKTatlas_lh_volume_superiorfrontal	Desikan Atlas	Total body	-0.11	0.05	1.58E-02	5.06E-02
181	26530	aseg_global_volume_Optic-Chiasm	aseg:global	Total body	-0.11	0.05	1.60E-02	3.30E-02
662	26735	aparc-Desikan_lh_area_middletemporal	Desikan Atlas	Total body	-0.10	0.04	1.60E-02	5.23E-02
861	27256	aparc-DKTatlas_rh_area_posteriorcingulate	Desikan Atlas	Forearm	-0.41	0.17	1.62E-02	7.96E-01
603	27728	aparc-a2009s_rh_volume_G-precuneus	Destrieux Atlas	Total body	-0.11	0.05	1.62E-02	6.58E-02
235	26612	AmygNuclei_rh_volume_Accessory-Basal-nucleus	Amygdala Nuclei	Total body	-0.11	0.05	1.63E-02	1.09E-01
797	27102	BA-exvivo_rh_area_BA2	Broadmann Atlas	Femoral neck	0.19	0.08	1.64E-02	1.53E-01
746	26953	aparc-pial_lh_area_supramarginal	Desikan Atlas	Femoral neck	0.15	0.06	1.64E-02	8.80E-01
729	26936	aparc-pial_lh_area_medialorbitofrontal	Desikan Atlas	Total body	-0.15	0.06	1.64E-02	5.27E-02
599	27724	aparc-a2009s_rh_volume_G-pariet-inf-Supramar	Destrieux Atlas	Total body	-0.13	0.05	1.64E-02	6.58E-02
990	27595	aparc-a2009s_rh_area_S-central	Destrieux Atlas	Heel	-0.11	0.05	1.65E-02	3.80E-01
438	27205	aparc-DKTatlas_lh_volume_caudalanteriorcingulate	Desikan Atlas	Lumbar spine	-0.17	0.07	1.65E-02	1.09E-01
1083	26885	aparc-Desikan_rh_thickness_superiortemporal	Desikan Atlas	Femoral neck	-0.15	0.06	1.67E-02	1.62E-01
516	27493	aparc-a2009s_lh_volume_G-Ins-lg+S-cent-ins	Destrieux Atlas	Total body	-0.13	0.06	1.68E-02	6.58E-02
834	27167	aparc-DKTatlas_lh_area_rostralmiddlefrontal	Desikan Atlas	Total body	-0.09	0.04	1.69E-02	5.27E-02
590	27715	aparc-a2009s_rh_volume_G-Ins-lg+S-cent-ins	Destrieux Atlas	Total body	-0.11	0.05	1.69E-02	6.58E-02
646	27771	aparc-a2009s_rh_volume_S-temporal-sup	Destrieux Atlas	Femoral neck	-0.16	0.07	1.70E-02	5.70E-01
306	26683	ThalamNuclei_lh_volume_Pt	Thalamus Nuclei	Heel	-0.08	0.03	1.70E-02	4.42E-01
1031	26766	aparc-Desikan_lh_thickness_lateralorbitofrontal	Desikan Atlas	Lumbar spine	-0.20	0.08	1.70E-02	5.24E-01
701	26841	aparc-Desikan_rh_area_parstriangularis	Desikan Atlas	Total body	-0.12	0.05	1.70E-02	5.27E-02
134	25890	IDP_T1_FAST_ROIs_L_ventral_striatum	IDP T1:unilateral regions	Femoral neck	-0.15	0.06	1.71E-02	6.42E-02
392	26905	aparc-Desikan_rh_volume_paracentral	Desikan Atlas	Lumbar spine	-0.18	0.07	1.71E-02	1.09E-01
868	27263	aparc-DKTatlas_rh_area_superiortemporal	Desikan Atlas	Lumbar spine	-0.14	0.06	1.71E-02	2.54E-01
906	27363	aparc-a2009s_lh_area_G-temp-sup-Plan-polar	Destrieux Atlas	Total body	-0.12	0.05	1.71E-02	7.55E-02

895	27352	aparc-a2009s_lh_area_G-orbital	Destrieux Atlas	Total body	-0.10	0.04	1.71E-02	7.55E-02
175	26524	aseg_global_volume_4th-Ventricle	aseg:global	Total body	-0.08	0.04	1.72E-02	3.30E-02
840	27173	aparc-DKTatlas_lh_area_insula	Desikan Atlas	Total body	-0.10	0.04	1.72E-02	5.27E-02
800	27105	BA-exvivo_rh_area_BA4a	Broadmann Atlas	Total body	-0.11	0.04	1.73E-02	6.19E-02
58	25814	IDP_T1_FAST_ROIs_L_postcent_gyrus	IDP T1:unilateral regions	Total body	0.12	0.05	1.75E-02	4.62E-02
106	25862	IDP_T1_FAST_ROIs_L_front_operc_cortex	IDP T1:unilateral regions	Total body	0.12	0.05	1.76E-02	4.62E-02
1198	27423	aparc-a2009s_lh_thickness_G-oc-temp-lat-fusifor	Destrieux Atlas	Total body	-0.13	0.05	1.76E-02	7.25E-01
31	25787	IDP_T1_FAST_ROIs_R_sup_front_gyrus	IDP T1:unilateral regions	Lumbar spine	0.19	0.08	1.80E-02	1.43E-01
952	27557	aparc-a2009s_rh_area_G+S-cingul-Mid-Ant	Destrieux Atlas	Lumbar spine	-0.14	0.06	1.80E-02	2.96E-01
704	26844	aparc-Desikan_rh_area_posteriorcingulate	Desikan Atlas	Forearm	-0.41	0.17	1.81E-02	7.96E-01
305	26682	ThalamNuclei_lh_volume_PuL	Thalamus Nuclei	Total body	-0.10	0.04	1.82E-02	7.89E-02
244	26621	HippSubfield_lh_volume_subiculum-body	Hippocampus Subfield	Total body	-0.13	0.05	1.82E-02	1.14E-01
480	27309	aparc-DKTatlas_rh_volume_medialorbitofrontal	Desikan Atlas	Total body	-0.12	0.05	1.84E-02	5.74E-02
716	26923	aparc-pial_lh_area_TotalSurface	Desikan Atlas	Lumbar spine	-0.14	0.06	1.85E-02	2.54E-01
611	27736	aparc-a2009s_rh_volume_G-temporal-middle	Destrieux Atlas	Total body	-0.12	0.05	1.85E-02	7.02E-02
161	25917	IDP_T1_FAST_ROIs_R_cerebellum_IX	IDP T1:unilateral regions	Lumbar spine	-0.13	0.05	1.87E-02	1.43E-01
749	26956	aparc-pial_rh_area_TotalSurface	Desikan Atlas	Lumbar spine	-0.15	0.06	1.88E-02	2.54E-01
168	26517	aseg_global_volume_SubCortGray	aseg:global	Total body	-0.09	0.04	1.89E-02	3.34E-02
873	27330	aparc-a2009s_lh_area_G+S-occipital-inf	Destrieux Atlas	Total body	-0.14	0.06	1.89E-02	7.99E-02
497	27326	aparc-DKTatlas_rh_volume_supramarginal	Desikan Atlas	Total body	-0.13	0.06	1.91E-02	5.82E-02
37	25793	IDP_T1_FAST_ROIs_R_inf_front_gyrus_parsop	IDP T1:unilateral regions	Lumbar spine	0.23	0.10	1.92E-02	1.43E-01
698	26838	aparc-Desikan_rh_area_paracentral	Desikan Atlas	Total body	-0.10	0.04	1.92E-02	5.79E-02
973	27578	aparc-a2009s_rh_area_G-postcentral	Destrieux Atlas	Femoral neck	0.16	0.07	1.94E-02	5.74E-01
178	26527	aseg_global_volume_CSF	aseg:global	Femoral neck	-0.12	0.05	1.95E-02	1.50E-01
88	25844	IDP_T1_FAST_ROIs_L_cuneal_cortex	IDP T1:unilateral regions	Lumbar spine	0.16	0.07	1.95E-02	1.43E-01
43	25799	IDP_T1_FAST_ROIs_R_sup_temp_gyrus_ant	IDP T1:unilateral regions	Femoral neck	-0.19	0.08	1.96E-02	7.17E-02
588	27713	aparc-a2009s_rh_volume_G-front-middle	Destrieux Atlas	Lumbar spine	-0.17	0.07	1.96E-02	1.45E-01
796	27101	BA-exvivo_rh_area_BA1	Broadmann Atlas	Total body	-0.12	0.05	1.96E-02	6.19E-02
1222	27447	aparc-a2009s_lh_thickness_S-central	Destrieux Atlas	Total body	-0.11	0.05	1.96E-02	7.25E-01
463	27230	aparc-DKTatlas_lh_volume_superiorfrontal	Desikan Atlas	Heel	-0.08	0.03	1.97E-02	8.29E-01
788	27065	BA-exvivo_lh_area_BA6	Broadmann Atlas	Total body	-0.09	0.04	1.98E-02	6.19E-02
850	27245	aparc-DKTatlas_rh_area_lateralorbitofrontal	Desikan Atlas	Lumbar spine	-0.14	0.06	1.99E-02	2.54E-01
804	27109	BA-exvivo_rh_area_BA45	Broadmann Atlas	Total body	-0.12	0.05	1.99E-02	6.19E-02

697	26837	aparc-Desikan_rh_area_parahippocampal	Desikan Atlas	Heel	-0.08	0.03	2.01E-02	4.09E-01
596	27721	aparc-a2009s_rh_volume_G-oc-temp-med-Parahip	Destrieux Atlas	Heel	-0.08	0.03	2.01E-02	4.96E-01
101	25857	IDP_T1_FAST_ROIs_R_temp_fusif_cortex_post	IDP T1:unilateral regions	Heel	0.07	0.03	2.02E-02	5.30E-02
96	25852	IDP_T1_FAST_ROIs_L_lingual_gyrus	IDP T1:unilateral regions	Femoral neck	-0.15	0.07	2.04E-02	7.27E-02
164	25920	IDP_T1_FAST_ROIs_R_cerebellum_X	IDP T1:unilateral regions	Forearm	0.36	0.15	2.04E-02	7.65E-01
974	27579	aparc-a2009s_rh_area_G-precentral	Destrieux Atlas	Total body	-0.09	0.04	2.04E-02	8.36E-02
489	27318	aparc-DKTatlas_rh_volume_posteriorcingulate	Desikan Atlas	Lumbar spine	-0.19	0.08	2.05E-02	1.20E-01
482	27311	aparc-DKTatlas_rh_volume_parahippocampal	Desikan Atlas	Total body	-0.10	0.04	2.06E-02	5.96E-02
498	27327	aparc-DKTatlas_rh_volume_transversetemporal	Desikan Atlas	Total body	-0.11	0.05	2.06E-02	5.96E-02
496	27325	aparc-DKTatlas_rh_volume_superiortemporal	Desikan Atlas	Lumbar spine	-0.16	0.07	2.07E-02	1.20E-01
750	26957	aparc-pial_rh_area_bankssts	Desikan Atlas	Heel	-0.10	0.04	2.08E-02	4.09E-01
422	27099	BA-exvivo_lh_volume_perirhinal	Broadmann Atlas	Total body	-0.11	0.05	2.08E-02	8.32E-02
872	27329	aparc-a2009s_lh_area_G+S-frontomargin	Destrieux Atlas	Total body	-0.11	0.05	2.09E-02	8.36E-02
1318	27691	aparc-a2009s_rh_thickness_S-postcentral	Destrieux Atlas	Femoral neck	-0.16	0.07	2.10E-02	2.96E-01
327	26704	ThalamNuclei_rh_volume_VM	Thalamus Nuclei	Lumbar spine	0.13	0.06	2.10E-02	5.69E-01
445	27212	aparc-DKTatlas_lh_volume_isthmuscingulate	Desikan Atlas	Total body	-0.10	0.04	2.10E-02	5.96E-02
798	27103	BA-exvivo_rh_area_BA3a	Broadmann Atlas	Heel	-0.10	0.04	2.11E-02	2.26E-01
855	27250	aparc-DKTatlas_rh_area_paracentral	Desikan Atlas	Total body	-0.10	0.04	2.11E-02	6.27E-02
500	27477	aparc-a2009s_lh_volume_G+S-frontomargin	Destrieux Atlas	Forearm	-0.44	0.19	2.12E-02	7.91E-01
529	27506	aparc-a2009s_lh_volume_G-precuneus	Destrieux Atlas	Lumbar spine	-0.15	0.06	2.12E-02	1.49E-01
751	26958	aparc-pial_rh_area_caudalanteriorcingulate	Desikan Atlas	Total body	-0.14	0.06	2.15E-02	6.29E-02
106	25862	IDP_T1_FAST_ROIs_L_front_operc_cortex	IDP T1:unilateral regions	Femoral neck	0.17	0.07	2.17E-02	7.54E-02
960	27565	aparc-a2009s_rh_area_G-front-middle	Destrieux Atlas	Lumbar spine	-0.14	0.06	2.17E-02	3.01E-01
1079	26881	aparc-Desikan_rh_thickness_rostralanteriorcingulate	Desikan Atlas	Heel	0.13	0.06	2.18E-02	7.41E-01
740	26947	aparc-pial_lh_area_precuneus	Desikan Atlas	Total body	-0.09	0.04	2.21E-02	6.37E-02
484	27313	aparc-DKTatlas_rh_volume_parsopercularis	Desikan Atlas	Lumbar spine	-0.16	0.07	2.22E-02	1.24E-01
562	27539	aparc-a2009s_lh_volume_S-orbital-med-olfact	Destrieux Atlas	Femoral neck	-0.20	0.09	2.23E-02	5.70E-01
872	27329	aparc-a2009s_lh_area_G+S-frontomargin	Destrieux Atlas	Forearm	-0.43	0.19	2.23E-02	7.88E-01
499	27328	aparc-DKTatlas_rh_volume_insula	Desikan Atlas	Total body	-0.10	0.04	2.23E-02	5.96E-02
902	27359	aparc-a2009s_lh_area_G-rectus	Destrieux Atlas	Lumbar spine	-0.20	0.09	2.24E-02	3.01E-01

204	26567	aseg_lh_volume_choroid-plexus	aseg:unilateral regions	Total body	-0.09	0.04	2.24E-02	5.86E-02
389	26902	aparc-Desikan_rh_volume_medialorbitofrontal	Desikan Atlas	Total body	-0.12	0.05	2.24E-02	5.96E-02
27	25783	IDP_T1_FAST_ROIs_R_frontal_pole	IDP T1:unilateral regions	Femoral neck	0.14	0.06	2.25E-02	7.63E-02
935	27392	aparc-a2009s_lh_area_S-orbital-H-Shaped	Destrieux Atlas	Heel	0.06	0.03	2.25E-02	3.80E-01
351	26796	aparc-Desikan_lh_volume_inferiortemporal	Desikan Atlas	Total body	-0.11	0.05	2.25E-02	5.96E-02
1275	27648	aparc-a2009s_rh_thickness_G-orbital	Destrieux Atlas	Femoral neck	-0.21	0.09	2.26E-02	2.96E-01
123	25879	IDP_T1_FAST_ROIs_R_thalamus	IDP T1:unilateral regions	Heel	-0.07	0.03	2.26E-02	5.82E-02
34	25790	IDP_T1_FAST_ROIs_L_inf_front_gyrus_parstri	IDP T1:unilateral regions	Lumbar spine	0.19	0.09	2.26E-02	1.44E-01
1034	26769	aparc-Desikan_lh_thickness_middletemporal	Desikan Atlas	Femoral neck	-0.17	0.07	2.27E-02	1.93E-01
808	27113	BA-exvivo_rh_area_perirhinal	Broadmann Atlas	Total body	-0.09	0.04	2.27E-02	6.36E-02
981	27586	aparc-a2009s_rh_area_G-temp-sup-Plan-tempo	Destrieux Atlas	Total body	-0.13	0.06	2.27E-02	8.84E-02
490	27319	aparc-DKTatlas_rh_volume_precentral	Desikan Atlas	Total body	-0.09	0.04	2.28E-02	5.96E-02
158	25914	IDP_T1_FAST_ROIs_R_cerebellum_VIIIb	IDP T1:unilateral regions	Lumbar spine	-0.13	0.06	2.29E-02	1.44E-01
864	27259	aparc-DKTatlas_rh_area_rostralanteriorcingulate	Desikan Atlas	Heel	-0.08	0.03	2.30E-02	4.09E-01
30	25786	IDP_T1_FAST_ROIs_L_sup_front_gyrus	IDP T1:unilateral regions	Femoral neck	0.18	0.08	2.31E-02	7.65E-02
265	26642	HippSubfield_rh_volume_Hippocampal-tail	Hippocampus Subfield	Heel	-0.06	0.03	2.31E-02	8.31E-01
771	26978	aparc-pial_rh_area_posteriorcingulate	Desikan Atlas	Lumbar spine	-0.17	0.07	2.32E-02	2.54E-01
572	27549	aparc-a2009s_lh_volume_S-temporal-sup	Destrieux Atlas	Femoral neck	-0.18	0.08	2.33E-02	5.70E-01
711	26851	aparc-Desikan_rh_area_superiortemporal	Desikan Atlas	Lumbar spine	-0.14	0.06	2.34E-02	2.54E-01
674	26747	aparc-Desikan_lh_area_rostralmiddlefrontal	Desikan Atlas	Total body	-0.08	0.04	2.35E-02	6.68E-02
122	25878	IDP_T1_FAST_ROIs_L_thalamus	IDP T1:unilateral regions	Heel	-0.07	0.03	2.36E-02	5.96E-02
354	26799	aparc-Desikan_lh_volume_lateralorbitofrontal	Desikan Atlas	Lumbar spine	-0.15	0.06	2.37E-02	1.26E-01
366	26811	aparc-Desikan_lh_volume_precentral	Desikan Atlas	Total body	-0.10	0.04	2.37E-02	6.07E-02
573	27550	aparc-a2009s_lh_volume_S-temporal-transverse	Destrieux Atlas	Femoral neck	-0.27	0.12	2.39E-02	5.70E-01
953	27558	aparc-a2009s_rh_area_G+S-cingul-Mid-Post	Destrieux Atlas	Forearm	-0.38	0.17	2.39E-02	7.88E-01
872	27329	aparc-a2009s_lh_area_G+S-frontomargin	Destrieux Atlas	Heel	-0.07	0.03	2.39E-02	3.80E-01
607	27732	aparc-a2009s_rh_volume_G-temp-sup-Lateral	Destrieux Atlas	Lumbar spine	-0.16	0.07	2.39E-02	1.56E-01
835	27168	aparc-DKTatlas_lh_area_superiorfrontal	Desikan Atlas	Heel	-0.09	0.04	2.41E-02	4.09E-01
138	25894	IDP_T1_FAST_ROIs_R_cerebellum_I-IV	IDP T1:unilateral regions	Lumbar spine	-0.13	0.06	2.41E-02	1.44E-01
580	27705	aparc-a2009s_rh_volume_G+S-cingul-Mid-Ant	Destrieux Atlas	Lumbar spine	-0.16	0.07	2.42E-02	1.56E-01
40	25796	IDP_T1_FAST_ROIs_L_temporal_pole	IDP T1:unilateral regions	Femoral neck	0.16	0.07	2.43E-02	7.86E-02
1027	26762	aparc-Desikan_lh_thickness_inferiorparietal	Desikan Atlas	Lumbar spine	-0.16	0.07	2.43E-02	5.24E-01
55	25811	IDP_T1_FAST_ROIs_R_inf_temp_gyrus_post	IDP T1:unilateral regions	Total body	0.10	0.04	2.44E-02	6.23E-02

860	27255	aparc-DKTatlas_rh_area_postcentral	Desikan Atlas	Total body	-0.11	0.05	2.45E-02	6.86E-02
501	27478	aparc-a2009s_lh_volume_G+S-occipital-inf	Destrieux Atlas	Total body	-0.13	0.06	2.45E-02	9.07E-02
65	25821	IDP_T1_FAST_ROIs_R_supramarg_gyrus_post	IDP T1:unilateral regions	Heel	0.11	0.05	2.46E-02	6.11E-02
888	27345	aparc-a2009s_lh_area_G-Ins-lg+S-cent-ins	Destrieux Atlas	Heel	-0.09	0.04	2.46E-02	3.80E-01
650	26723	aparc-Desikan_lh_area_caudalanteriorcingulate	Desikan Atlas	Heel	-0.11	0.05	2.47E-02	4.09E-01
123	25879	IDP_T1_FAST_ROIs_R_thalamus	IDP T1:unilateral regions	Lumbar spine	-0.12	0.05	2.47E-02	1.44E-01
1288	27661	aparc-a2009s_rh_thickness_G-temporal-inf	Destrieux Atlas	Femoral neck	-0.17	0.08	2.48E-02	2.96E-01
1013	27618	aparc-a2009s_rh_area_S-precentral-inf-part	Destrieux Atlas	Forearm	0.50	0.22	2.50E-02	7.88E-01
34	25790	IDP_T1_FAST_ROIs_L_inf_front_gyrus_parstri	IDP T1:unilateral regions	Total body	0.13	0.06	2.50E-02	6.23E-02
1034	26769	aparc-Desikan_lh_thickness_middletemporal	Desikan Atlas	Total body	-0.12	0.05	2.50E-02	6.29E-01
97	25853	IDP_T1_FAST_ROIs_R_lingual_gyrus	IDP T1:unilateral regions	Total body	-0.11	0.05	2.51E-02	6.23E-02
845	27240	aparc-DKTatlas_rh_area_fusiform	Desikan Atlas	Lumbar spine	-0.15	0.07	2.52E-02	2.54E-01
672	26745	aparc-Desikan_lh_area_precuneus	Desikan Atlas	Total body	-0.08	0.04	2.53E-02	6.98E-02
1075	26877	aparc-Desikan_rh_thickness_postcentral	Desikan Atlas	Lumbar spine	-0.14	0.06	2.55E-02	5.24E-01
32	25788	IDP_T1_FAST_ROIs_L_mid_front_gyrus	IDP T1:unilateral regions	Lumbar spine	0.16	0.07	2.56E-02	1.44E-01
1011	27616	aparc-a2009s_rh_area_S-pericallosal	Destrieux Atlas	Heel	-0.08	0.04	2.57E-02	3.80E-01
707	26847	aparc-Desikan_rh_area_rostralanteriorcingulate	Desikan Atlas	Total body	-0.12	0.06	2.57E-02	7.00E-02
96	25852	IDP_T1_FAST_ROIs_L_lingual_gyrus	IDP T1:unilateral regions	Lumbar spine	-0.16	0.07	2.59E-02	1.44E-01
250	26627	HippSubfield_lh_volume_presubiculum-body	Hippocampus Subfield	Lumbar spine	-0.14	0.06	2.60E-02	7.20E-01
636	27761	aparc-a2009s_rh_volume_S-orbital-med-olfact	Destrieux Atlas	Total body	-0.12	0.05	2.60E-02	9.39E-02
121	25877	IDP_T1_FAST_ROIs_R_occ_pole	IDP T1:unilateral regions	Femoral neck	0.13	0.06	2.61E-02	8.25E-02
54	25810	IDP_T1_FAST_ROIs_L_inf_temp_gyrus_post	IDP T1:unilateral regions	Heel	0.12	0.05	2.62E-02	6.39E-02
707	26847	aparc-Desikan_rh_area_rostralanteriorcingulate	Desikan Atlas	Heel	-0.08	0.04	2.62E-02	4.09E-01
486	27315	aparc-DKTatlas_rh_volume_parstriangularis	Desikan Atlas	Total body	-0.14	0.06	2.63E-02	6.27E-02
459	27226	aparc-DKTatlas_lh_volume_precentral	Desikan Atlas	Total body	-0.09	0.04	2.63E-02	6.27E-02
762	26969	aparc-pial_rh_area_medialorbitofrontal	Desikan Atlas	Lumbar spine	-0.17	0.08	2.64E-02	2.54E-01
212	26589	aseg_rh_volume_Thalamus-Proper	aseg:unilateral regions	Total body	-0.08	0.04	2.65E-02	6.44E-02
338	26715	ThalamNuclei_rh_volume_Whole-thalamus	Thalamus Nuclei	Total body	-0.08	0.03	2.65E-02	1.01E-01
688	26828	aparc-Desikan_rh_area_fusiform	Desikan Atlas	Lumbar spine	-0.15	0.07	2.67E-02	2.54E-01
344	26789	aparc-Desikan_lh_volume_bankssts	Desikan Atlas	Heel	-0.07	0.03	2.68E-02	8.29E-01
858	27253	aparc-DKTatlas_rh_area_parstriangularis	Desikan Atlas	Total body	-0.12	0.05	2.68E-02	7.13E-02
941	27398	aparc-a2009s_lh_area_S-suborbital	Destrieux Atlas	Femoral neck	0.22	0.10	2.69E-02	6.64E-01
365	26810	aparc-Desikan_lh_volume_posteriorcingulate	Desikan Atlas	Lumbar spine	-0.20	0.09	2.69E-02	1.33E-01

514	27491	aparc-a2009s_lh_volume_G-front-middle	Destrieux Atlas	Total body	-0.10	0.05	2.69E-02	9.48E-02
399	26912	aparc-Desikan_rh_volume_precentral	Desikan Atlas	Total body	-0.09	0.04	2.70E-02	6.27E-02
856	27251	aparc-DKTatlas_rh_area_parsopercularis	Desikan Atlas	Total body	-0.11	0.05	2.70E-02	7.13E-02
1250	27475	aparc-a2009s_lh_thickness_S-temporal-sup	Destrieux Atlas	Total body	-0.11	0.05	2.70E-02	7.25E-01
787	27064	BA-exvivo_lh_area_BA4p	Broadmann Atlas	Heel	-0.08	0.04	2.71E-02	2.26E-01
770	26977	aparc-pial_rh_area_postcentral	Desikan Atlas	Heel	-0.08	0.04	2.71E-02	4.09E-01
63	25819	IDP_T1_FAST_ROIs_R_supramarg_gyrus_ant	IDP T1:unilateral regions	Total body	0.14	0.06	2.71E-02	6.54E-02
330	26707	ThalamNuclei_rh_volume_VLp	Thalamus Nuclei	Total body	-0.07	0.03	2.72E-02	1.01E-01
92	25848	IDP_T1_FAST_ROIs_L_parahipp_gyrus_ant	IDP T1:unilateral regions	Total body	0.10	0.05	2.73E-02	6.54E-02
765	26972	aparc-pial_rh_area_paracentral	Desikan Atlas	Total body	-0.10	0.05	2.73E-02	7.13E-02
120	25876	IDP_T1_FAST_ROIs_L_occ_pole	IDP T1:unilateral regions	Heel	0.08	0.03	2.74E-02	6.57E-02
382	26895	aparc-Desikan_rh_volume_fusiform	Desikan Atlas	Lumbar spine	-0.14	0.06	2.74E-02	1.33E-01
976	27581	aparc-a2009s_rh_area_G-rectus	Destrieux Atlas	Lumbar spine	-0.17	0.08	2.75E-02	3.31E-01
397	26910	aparc-Desikan_rh_volume_postcentral	Desikan Atlas	Total body	-0.10	0.05	2.75E-02	6.27E-02
468	27235	aparc-DKTatlas_lh_volume_insula	Desikan Atlas	Total body	-0.09	0.04	2.76E-02	6.27E-02
663	26736	aparc-Desikan_lh_area_parahippocampal	Desikan Atlas	Lumbar spine	-0.15	0.07	2.77E-02	2.54E-01
444	27211	aparc-DKTatlas_lh_volume_inferiortemporal	Desikan Atlas	Total body	-0.11	0.05	2.79E-02	6.27E-02
350	26795	aparc-Desikan_lh_volume_inferiorparietal	Desikan Atlas	Lumbar spine	-0.16	0.07	2.80E-02	1.33E-01
1318	27691	aparc-a2009s_rh_thickness_S-postcentral	Destrieux Atlas	Lumbar spine	-0.16	0.07	2.80E-02	5.57E-01
794	27071	BA-exvivo_lh_area_perirhinal	Broadmann Atlas	Total body	-0.09	0.04	2.80E-02	7.13E-02
606	27731	aparc-a2009s_rh_volume_G-temp-sup-G-T-transv	Destrieux Atlas	Total body	-0.12	0.05	2.80E-02	9.64E-02
488	27317	aparc-DKTatlas_rh_volume_postcentral	Desikan Atlas	Total body	-0.10	0.05	2.81E-02	6.27E-02
348	26793	aparc-Desikan_lh_volume_entorhinal	Desikan Atlas	Total body	-0.11	0.05	2.84E-02	6.27E-02
104	25860	IDP_T1_FAST_ROIs_L_occ_fusif_gyrus	IDP T1:unilateral regions	Heel	0.08	0.03	2.85E-02	6.71E-02
432	27137	BA-exvivo_rh_volume_BA45	Broadmann Atlas	Lumbar spine	-0.16	0.07	2.85E-02	2.00E-01
372	26817	aparc-Desikan_lh_volume_superiortemporal	Desikan Atlas	Femoral neck	-0.14	0.06	2.86E-02	7.76E-01
618	27743	aparc-a2009s_rh_volume_S-central	Destrieux Atlas	Heel	-0.08	0.04	2.86E-02	5.49E-01
682	26822	aparc-Desikan_rh_area_TotalSurface	Desikan Atlas	Lumbar spine	-0.13	0.06	2.86E-02	2.54E-01
1307	27680	aparc-a2009s_rh_thickness_S-intrapariet+P-trans	Destrieux Atlas	Femoral neck	-0.15	0.07	2.87E-02	2.96E-01
570	27547	aparc-a2009s_lh_volume_S-subparietal	Destrieux Atlas	Total body	-0.10	0.05	2.87E-02	9.65E-02
1291	27664	aparc-a2009s_rh_thickness_Lat-Fis-ant-Vertical	Destrieux Atlas	Heel	0.11	0.05	2.88E-02	9.59E-01
303	26680	ThalamNuclei_lh_volume_VM	Thalamus Nuclei	Lumbar spine	0.13	0.06	2.88E-02	5.69E-01
111	25867	IDP_T1_FAST_ROIs_R_parietal_operc_cortex	IDP T1:unilateral regions	Femoral neck	0.14	0.06	2.89E-02	8.85E-02

427	27132	BA-exvivo_rh_volume_BA3b	Broadmann Atlas	Heel	-0.07	0.03	2.89E-02	3.13E-01
414	27091	BA-exvivo_lh_volume_BA4a	Broadmann Atlas	Total body	-0.11	0.05	2.89E-02	1.01E-01
284	26661	HippSubfield_rh_volume_Whole-hippocampal-body	Hippocampus Subfield	Total body	-0.10	0.04	2.89E-02	1.42E-01
779	26986	aparc-pial_rh_area_supramarginal	Desikan Atlas	Total body	-0.12	0.05	2.90E-02	7.47E-02
98	25854	IDP_T1_FAST_ROIs_L_temp_fusif_cortex_ant	IDP T1:unilateral regions	Femoral neck	0.16	0.07	2.93E-02	8.85E-02
340	26717	Brainstem_global_volume_Pons	Brain Stem	Heel	-0.06	0.03	2.94E-02	7.35E-02
123	25879	IDP_T1_FAST_ROIs_R_thalamus	IDP T1:unilateral regions	Total body	-0.08	0.04	2.94E-02	6.93E-02
672	26745	aparc-Desikan_lh_area_precuneus	Desikan Atlas	Lumbar spine	-0.14	0.06	2.96E-02	2.54E-01
516	27493	aparc-a2009s_lh_volume_G-Ins-lg+S-cent-ins	Destrieux Atlas	Heel	-0.09	0.04	2.97E-02	5.49E-01
1287	27660	aparc-a2009s_rh_thickness_G-temp-sup-Plan-tempo	Destrieux Atlas	Femoral neck	-0.15	0.07	2.99E-02	2.96E-01
589	27714	aparc-a2009s_rh_volume_G-front-sup	Destrieux Atlas	Total body	-0.10	0.05	2.99E-02	9.83E-02
469	27298	aparc-DKTatlas_rh_volume_caudalanteriorcingulate	Desikan Atlas	Femoral neck	-0.22	0.10	3.01E-02	7.76E-01
997	27602	aparc-a2009s_rh_area_S-front-inf	Destrieux Atlas	Lumbar spine	-0.15	0.07	3.01E-02	3.31E-01
738	26945	aparc-pial_lh_area_posteriorcingulate	Desikan Atlas	Total body	-0.12	0.06	3.01E-02	7.47E-02
727	26934	aparc-pial_lh_area_lateralorbitofrontal	Desikan Atlas	Total body	-0.10	0.04	3.02E-02	7.47E-02
378	26891	aparc-Desikan_rh_volume_caudalanteriorcingulate	Desikan Atlas	Femoral neck	-0.22	0.10	3.03E-02	7.76E-01
59	25815	IDP_T1_FAST_ROIs_R_postcent_gyrus	IDP T1:unilateral regions	Heel	0.09	0.04	3.03E-02	7.02E-02
832	27165	aparc-DKTatlas_lh_area_precuneus	Desikan Atlas	Total body	-0.08	0.04	3.03E-02	7.47E-02
317	26694	ThalamNuclei_rh_volume_CM	Thalamus Nuclei	Total body	-0.08	0.04	3.03E-02	1.05E-01
162	25918	IDP_T1_FAST_ROIs_L_cerebellum_X	IDP T1:unilateral regions	Forearm	0.32	0.15	3.04E-02	7.65E-01
12	25012	IDP_T1_FIRST_right_thalamus_volume	IDP T1:global	Total body	-0.09	0.04	3.04E-02	1.52E-01
763	26970	aparc-pial_rh_area_middletemporal	Desikan Atlas	Total body	-0.10	0.05	3.05E-02	7.47E-02
429	27134	BA-exvivo_rh_volume_BA4p	Broadmann Atlas	Femoral neck	-0.15	0.07	3.08E-02	4.53E-01
1082	26884	aparc-Desikan_rh_thickness_superiorparietal	Desikan Atlas	Lumbar spine	-0.13	0.06	3.08E-02	5.24E-01
778	26985	aparc-pial_rh_area_superiortemporal	Desikan Atlas	Lumbar spine	-0.15	0.07	3.09E-02	2.54E-01
1307	27680	aparc-a2009s_rh_thickness_S-intrapariet+P-trans	Destrieux Atlas	Lumbar spine	-0.15	0.07	3.09E-02	5.57E-01
722	26929	aparc-pial_lh_area_fusiform	Desikan Atlas	Total body	-0.12	0.05	3.09E-02	7.48E-02
1027	26762	aparc-Desikan_lh_thickness_inferiorparietal	Desikan Atlas	Total body	-0.10	0.05	3.09E-02	6.29E-01

1210	27435	aparc-a2009s_lh_thickness_G-temp-sup-G-T-transv	Destrieux Atlas	Forearm	-0.38	0.18	3.10E-02	9.94E-01
383	26896	aparc-Desikan_rh_volume_inferiorparietal	Desikan Atlas	Lumbar spine	-0.15	0.07	3.10E-02	1.40E-01
866	27261	aparc-DKTatlas_rh_area_superiorfrontal	Desikan Atlas	Lumbar spine	-0.12	0.05	3.10E-02	2.54E-01
533	27510	aparc-a2009s_lh_volume_G-temp-sup-Lateral	Destrieux Atlas	Total body	-0.11	0.05	3.13E-02	1.01E-01
60	25816	IDP_T1_FAST_ROIs_L_sup_parietal_lobule	IDP T1:unilateral regions	Heel	0.10	0.05	3.16E-02	7.11E-02
152	25908	IDP_T1_FAST_ROIs_R_cerebellum_VIIb	IDP T1:unilateral regions	Femoral neck	-0.11	0.05	3.17E-02	9.25E-02
117	25873	IDP_T1_FAST_ROIs_R_planum_temporale	IDP T1:unilateral regions	Heel	0.07	0.03	3.17E-02	7.11E-02
801	27106	BA-exvivo_rh_area_BA4p	Broadmann Atlas	Heel	-0.09	0.04	3.17E-02	2.26E-01
772	26979	aparc-pial_rh_area_precentral	Desikan Atlas	Total body	-0.08	0.04	3.17E-02	7.53E-02
369	26814	aparc-Desikan_lh_volume_rostralmiddlefrontal	Desikan Atlas	Lumbar spine	-0.13	0.06	3.18E-02	1.40E-01
563	27540	aparc-a2009s_lh_volume_S-orbital-H-Shaped	Destrieux Atlas	Lumbar spine	-0.15	0.07	3.18E-02	1.87E-01
810	27143	aparc-DKTatlas_lh_area_caudalanteriorcingulate	Desikan Atlas	Lumbar spine	-0.14	0.06	3.19E-02	2.54E-01
706	26846	aparc-Desikan_rh_area_precuneus	Desikan Atlas	Total body	-0.09	0.04	3.19E-02	7.53E-02
834	27167	aparc-DKTatlas_lh_area_rostralmiddlefrontal	Desikan Atlas	Lumbar spine	-0.12	0.06	3.20E-02	2.54E-01
86	25842	IDP_T1_FAST_ROIs_L_precun_cortex	IDP T1:unilateral regions	Total body	0.09	0.04	3.21E-02	7.44E-02
110	25866	IDP_T1_FAST_ROIs_L_parietal_operc_cortex	IDP T1:unilateral regions	Femoral neck	0.15	0.07	3.22E-02	9.25E-02
829	27162	aparc-DKTatlas_lh_area_postcentral	Desikan Atlas	Femoral neck	0.14	0.07	3.23E-02	8.80E-01
522	27499	aparc-a2009s_lh_volume_G-oc-temp-med-Parahip	Destrieux Atlas	Total body	-0.08	0.04	3.23E-02	1.02E-01
832	27165	aparc-DKTatlas_lh_area_precuneus	Desikan Atlas	Lumbar spine	-0.14	0.06	3.24E-02	2.54E-01
949	27554	aparc-a2009s_rh_area_G+S-subcentral	Destrieux Atlas	Total body	-0.11	0.05	3.24E-02	1.13E-01
150	25906	IDP_T1_FAST_ROIs_L_cerebellum_VIIb	IDP T1:unilateral regions	Femoral neck	-0.12	0.06	3.26E-02	9.25E-02
377	26890	aparc-Desikan_rh_volume_bankssts	Desikan Atlas	Heel	-0.07	0.03	3.26E-02	8.29E-01
621	27746	aparc-a2009s_rh_volume_S-circular-insula-inf	Destrieux Atlas	Lumbar spine	-0.16	0.07	3.26E-02	1.87E-01
492	27321	aparc-DKTatlas_rh_volume_rostralanteriorcingulate	Desikan Atlas	Total body	-0.12	0.06	3.26E-02	6.92E-02
725	26932	aparc-pial_lh_area_isthmuscingulate	Desikan Atlas	Total body	-0.09	0.04	3.26E-02	7.53E-02
957	27562	aparc-a2009s_rh_area_G-front-inf-Opercular	Destrieux Atlas	Total body	-0.12	0.06	3.26E-02	1.13E-01
1205	27430	aparc-a2009s_lh_thickness_G-postcentral	Destrieux Atlas	Femoral neck	-0.14	0.06	3.28E-02	2.96E-01
457	27224	aparc-DKTatlas_lh_volume_postcentral	Desikan Atlas	Total body	-0.10	0.05	3.28E-02	6.92E-02
983	27588	aparc-a2009s_rh_area_G-temporal-middle	Destrieux Atlas	Total body	-0.10	0.05	3.28E-02	1.13E-01
280	26657	HippSubfield_rh_volume_CA4-body	Hippocampus Subfield	Total body	-0.11	0.05	3.28E-02	1.42E-01
645	27770	aparc-a2009s_rh_volume_S-temporal-inf	Destrieux Atlas	Lumbar spine	0.16	0.07	3.29E-02	1.87E-01

659	26732	aparc-Desikan_lh_area_lateralorbitofrontal	Desikan Atlas	Total body	-0.09	0.04	3.29E-02	7.53E-02
923	27380	aparc-a2009s_lh_area_S-front-inf	Destrieux Atlas	Total body	-0.10	0.05	3.29E-02	1.13E-01
453	27220	aparc-DKTatlas_lh_volume_parsopercularis	Desikan Atlas	Total body	-0.11	0.05	3.30E-02	6.92E-02
853	27248	aparc-DKTatlas_rh_area_middletemporal	Desikan Atlas	Heel	-0.06	0.03	3.31E-02	4.63E-01
155	25911	IDP_T1_FAST_ROIs_R_cerebellum_VIIIa	IDP T1:unilateral regions	Lumbar spine	-0.12	0.06	3.31E-02	1.69E-01
580	27705	aparc-a2009s_rh_volume_G+S-cingul-Mid-Ant	Destrieux Atlas	Femoral neck	-0.15	0.07	3.32E-02	5.70E-01
770	26977	aparc-pial_rh_area_postcentral	Desikan Atlas	Total body	-0.10	0.05	3.32E-02	7.53E-02
1077	26879	aparc-Desikan_rh_thickness_precentral	Desikan Atlas	Femoral neck	-0.14	0.07	3.33E-02	2.38E-01
1008	27613	aparc-a2009s_rh_area_S-orbital-med-olfact	Destrieux Atlas	Lumbar spine	-0.12	0.06	3.33E-02	3.31E-01
802	27107	BA-exvivo_rh_area_BA6	Broadmann Atlas	Total body	-0.09	0.04	3.34E-02	7.79E-02
912	27369	aparc-a2009s_lh_area_Lat-Fis-post	Destrieux Atlas	Total body	-0.09	0.04	3.34E-02	1.13E-01
56	25812	IDP_T1_FAST_ROIs_L_inf_temp_gyrus_tempocc	IDP T1:unilateral regions	Forearm	0.35	0.16	3.35E-02	7.65E-01
412	27089	BA-exvivo_lh_volume_BA3a	Broadmann Atlas	Heel	-0.09	0.04	3.35E-02	3.13E-01
963	27568	aparc-a2009s_rh_area_G-insular-short	Destrieux Atlas	Heel	-0.08	0.04	3.35E-02	4.06E-01
774	26981	aparc-pial_rh_area_rostralanteriorcingulate	Desikan Atlas	Total body	-0.13	0.06	3.36E-02	7.53E-02
1087	26889	aparc-Desikan_rh_thickness_insula	Desikan Atlas	Heel	0.13	0.06	3.37E-02	7.64E-01
673	26746	aparc-Desikan_lh_area_rostralanteriorcingulate	Desikan Atlas	Total body	-0.09	0.04	3.38E-02	7.53E-02
888	27345	aparc-a2009s_lh_area_G-Ins-lg+S-cent-ins	Destrieux Atlas	Total body	-0.11	0.05	3.38E-02	1.13E-01
79	25835	IDP_T1_FAST_ROIs_R_subcallosal_cortex	IDP T1:unilateral regions	Lumbar spine	-0.12	0.06	3.39E-02	1.69E-01
1251	27476	aparc-a2009s_lh_thickness_S-temporal-transverse	Destrieux Atlas	Lumbar spine	-0.15	0.07	3.39E-02	5.57E-01
106	25862	IDP_T1_FAST_ROIs_L_front_operc_cortex	IDP T1:unilateral regions	Lumbar spine	0.16	0.08	3.40E-02	1.69E-01
929	27386	aparc-a2009s_lh_area_S-oc-sup+transversal	Destrieux Atlas	Lumbar spine	-0.16	0.07	3.41E-02	3.31E-01
391	26904	aparc-Desikan_rh_volume_parahippocampal	Desikan Atlas	Total body	-0.09	0.04	3.42E-02	7.06E-02
992	27597	aparc-a2009s_rh_area_S-circular-insula-ant	Destrieux Atlas	Total body	-0.10	0.05	3.44E-02	1.13E-01
1215	27440	aparc-a2009s_lh_thickness_G-temporal-middle	Destrieux Atlas	Total body	-0.11	0.05	3.44E-02	7.25E-01
190	26553	aseg_lh_volume_CerebralWhiteMatter	aseg:unilateral regions	Heel	-0.05	0.03	3.45E-02	2.24E-01
1276	27649	aparc-a2009s_rh_thickness_G-pariet-inf-Angular	Destrieux Atlas	Femoral neck	-0.14	0.07	3.46E-02	2.96E-01
1255	27628	aparc-a2009s_rh_thickness_G+S-subcentral	Destrieux Atlas	Femoral neck	-0.13	0.06	3.48E-02	2.96E-01
242	26619	AmygNuclei_rh_volume_Whole-amygdala	Amygdala Nuclei	Total body	-0.10	0.05	3.48E-02	1.74E-01
1241	27466	aparc-a2009s_lh_thickness_S-orbital-H-Shaped	Destrieux Atlas	Femoral neck	-0.18	0.08	3.50E-02	2.96E-01
92	25848	IDP_T1_FAST_ROIs_L_parahipp_gyrus_ant	IDP T1:unilateral regions	Heel	0.07	0.03	3.50E-02	7.72E-02
618	27743	aparc-a2009s_rh_volume_S-central	Destrieux Atlas	Femoral neck	-0.14	0.07	3.51E-02	5.70E-01

657	26730	aparc-Desikan_lh_area_isthmuscingulate	Desikan Atlas	Total body	-0.09	0.04	3.51E-02	7.73E-02
265	26642	HippSubfield_rh_volume_Hippocampal-tail	Hippocampus Subfield	Total body	-0.08	0.04	3.53E-02	1.42E-01
62	25818	IDP_T1_FAST_ROIs_L_supramarg_gyrus_ant	IDP T1:unilateral regions	Lumbar spine	0.18	0.09	3.54E-02	1.70E-01
966	27571	aparc-a2009s_rh_area_G-oc-temp-lat-fusifor	Destrieux Atlas	Femoral neck	-0.16	0.08	3.55E-02	7.36E-01
431	27136	BA-exvivo_rh_volume_BA44	Broadmann Atlas	Total body	-0.10	0.05	3.55E-02	1.08E-01
641	27766	aparc-a2009s_rh_volume_S-precentral-inf-part	Destrieux Atlas	Forearm	0.45	0.22	3.56E-02	7.91E-01
286	26663	HippSubfield_rh_volume_Whole-hippocampus	Hippocampus Subfield	Total body	-0.09	0.04	3.56E-02	1.42E-01
124	25880	IDP_T1_FAST_ROIs_L_caudate	IDP T1:unilateral regions	Heel	-0.06	0.03	3.57E-02	7.72E-02
356	26801	aparc-Desikan_lh_volume_medialorbitofrontal	Desikan Atlas	Lumbar spine	-0.16	0.07	3.60E-02	1.54E-01
151	25907	IDP_T1_FAST_ROIs_V_cerebellum_VIIb	IDP T1:unilateral regions	Heel	-0.07	0.03	3.61E-02	7.72E-02
511	27488	aparc-a2009s_lh_volume_G-front-inf-Opercular	Destrieux Atlas	Total body	-0.12	0.06	3.61E-02	1.10E-01
674	26747	aparc-Desikan_lh_area_rostralmiddlefrontal	Desikan Atlas	Lumbar spine	-0.11	0.05	3.62E-02	2.66E-01
580	27705	aparc-a2009s_rh_volume_G+S-cingul-Mid-Ant	Destrieux Atlas	Total body	-0.10	0.05	3.63E-02	1.10E-01
210	26587	aseg_rh_volume_Cerebellum-White-Matter	aseg:unilateral regions	Heel	-0.06	0.03	3.64E-02	2.24E-01
784	27061	BA-exvivo_lh_area_BA3a	Broadmann Atlas	Heel	-0.09	0.04	3.64E-02	2.26E-01
831	27164	aparc-DKTatlas_lh_area_precentral	Desikan Atlas	Total body	-0.09	0.04	3.64E-02	7.93E-02
108	25864	IDP_T1_FAST_ROIs_L_cent_operc_cortex	IDP T1:unilateral regions	Femoral neck	0.15	0.07	3.66E-02	1.02E-01
675	26748	aparc-Desikan_lh_area_superiorfrontal	Desikan Atlas	Lumbar spine	-0.11	0.05	3.66E-02	2.66E-01
973	27578	aparc-a2009s_rh_area_G-postcentral	Destrieux Atlas	Heel	-0.10	0.05	3.68E-02	4.06E-01
671	26744	aparc-Desikan_lh_area_precentral	Desikan Atlas	Total body	-0.09	0.04	3.68E-02	7.93E-02
78	25834	IDP_T1_FAST_ROIs_L_subcallosal_cortex	IDP T1:unilateral regions	Total body	-0.08	0.04	3.68E-02	8.34E-02
297	26674	ThalamNuclei_lh_volume_Pf	Thalamus Nuclei	Total body	-0.08	0.04	3.68E-02	1.20E-01
170	26519	aseg_global_volume_SupraTentorial	aseg:global	Heel	-0.06	0.03	3.70E-02	9.46E-02
67	25823	IDP_T1_FAST_ROIs_R_angular_gyrus	IDP T1:unilateral regions	Total body	0.12	0.06	3.72E-02	8.34E-02
97	25853	IDP_T1_FAST_ROIs_R_lingual_gyrus	IDP T1:unilateral regions	Femoral neck	-0.12	0.06	3.73E-02	1.02E-01
13	25013	IDP_T1_FIRST_left_caudate_volume	IDP T1:global	Heel	-0.05	0.03	3.77E-02	1.51E-01
669	26742	aparc-Desikan_lh_area_postcentral	Desikan Atlas	Femoral neck	0.14	0.07	3.79E-02	8.80E-01
41	25797	IDP_T1_FAST_ROIs_R_temporal_pole	IDP T1:unilateral regions	Lumbar spine	0.13	0.06	3.79E-02	1.76E-01
519	27496	aparc-a2009s_lh_volume_G-occipital-sup	Destrieux Atlas	Lumbar spine	-0.13	0.06	3.79E-02	1.92E-01
364	26809	aparc-Desikan_lh_volume_postcentral	Desikan Atlas	Total body	-0.10	0.05	3.80E-02	7.72E-02
868	27263	aparc-DKTatlas_rh_area_superiortemporal	Desikan Atlas	Heel	-0.10	0.05	3.82E-02	4.99E-01
536	27513	aparc-a2009s_lh_volume_G-temporal-inf	Destrieux Atlas	Total body	-0.10	0.05	3.82E-02	1.13E-01
803	27108	BA-exvivo_rh_area_BA44	Broadmann Atlas	Total body	-0.10	0.05	3.83E-02	8.25E-02

581	27706	aparc-a2009s_rh_volume_G+S-cingul-Mid-Post	Destrieux Atlas	Forearm	-0.35	0.17	3.85E-02	7.91E-01
432	27137	BA-exvivo_rh_volume_BA45	Broadmann Atlas	Total body	-0.11	0.05	3.85E-02	1.08E-01
770	26977	aparc-pial_rh_area_postcentral	Desikan Atlas	Femoral neck	0.14	0.07	3.86E-02	8.80E-01
804	27109	BA-exvivo_rh_area_BA45	Broadmann Atlas	Lumbar spine	-0.14	0.07	3.86E-02	9.07E-01
916	27373	aparc-a2009s_lh_area_S-central	Destrieux Atlas	Heel	-0.08	0.04	3.87E-02	4.06E-01
93	25849	IDP_T1_FAST_ROIs_R_parahipp_gyrus_ant	IDP T1:unilateral regions	Total body	0.10	0.05	3.87E-02	8.47E-02
147	25903	IDP_T1_FAST_ROIs_L_cerebellum_crus_II	IDP T1:unilateral regions	Total body	-0.08	0.04	3.87E-02	8.47E-02
394	26907	aparc-Desikan_rh_volume_parsorbitalis	Desikan Atlas	Lumbar spine	-0.14	0.07	3.92E-02	1.60E-01
590	27715	aparc-a2009s_rh_volume_G-Ins-lg+S-cent-ins	Destrieux Atlas	Lumbar spine	-0.15	0.07	3.93E-02	1.92E-01
215	26592	aseg_rh_volume_Pallidum	aseg:unilateral regions	Heel	-0.06	0.03	3.95E-02	2.24E-01
979	27584	aparc-a2009s_rh_area_G-temp-sup-Lateral	Destrieux Atlas	Lumbar spine	-0.14	0.07	3.95E-02	3.31E-01
395	26908	aparc-Desikan_rh_volume_parstriangularis	Desikan Atlas	Total body	-0.12	0.06	3.95E-02	7.90E-02
1320	27693	aparc-a2009s_rh_thickness_S-precentral-sup-part	Destrieux Atlas	Femoral neck	-0.15	0.07	3.96E-02	2.96E-01
810	27143	aparc-DKTatlas_lh_area_caudalanteriorcingulate	Desikan Atlas	Forearm	-0.32	0.16	3.96E-02	7.96E-01
293	26670	ThalamNuclei_lh_volume_CM	Thalamus Nuclei	Heel	-0.06	0.03	3.96E-02	4.92E-01
581	27706	aparc-a2009s_rh_volume_G+S-cingul-Mid-Post	Destrieux Atlas	Lumbar spine	-0.15	0.07	3.96E-02	1.92E-01
530	27507	aparc-a2009s_lh_volume_G-rectus	Destrieux Atlas	Femoral neck	-0.16	0.08	3.97E-02	5.70E-01
565	27542	aparc-a2009s_lh_volume_S-pericallosal	Destrieux Atlas	Lumbar spine	-0.17	0.08	3.97E-02	1.92E-01
738	26945	aparc-pial_lh_area_posteriorcingulate	Desikan Atlas	Forearm	-0.37	0.18	3.98E-02	7.96E-01
161	25917	IDP_T1_FAST_ROIs_R_cerebellum_IX	IDP T1:unilateral regions	Forearm	-0.26	0.13	3.99E-02	7.65E-01
376	26821	aparc-Desikan_lh_volume_insula	Desikan Atlas	Lumbar spine	-0.13	0.06	4.01E-02	1.60E-01
659	26732	aparc-Desikan_lh_area_lateralorbitofrontal	Desikan Atlas	Femoral neck	0.13	0.06	4.03E-02	8.80E-01
573	27550	aparc-a2009s_lh_volume_S-temporal-transverse	Destrieux Atlas	Lumbar spine	-0.25	0.12	4.03E-02	1.92E-01
796	27101	BA-exvivo_rh_area_BA1	Broadmann Atlas	Heel	-0.10	0.05	4.04E-02	2.26E-01
648	26721	aparc-Desikan_lh_area_TotalSurface	Desikan Atlas	Lumbar spine	-0.12	0.06	4.05E-02	2.81E-01
1015	27620	aparc-a2009s_rh_area_S-suborbital	Destrieux Atlas	Lumbar spine	-0.25	0.12	4.05E-02	3.31E-01
507	27484	aparc-a2009s_lh_volume_G+S-cingul-Mid-Post	Destrieux Atlas	Forearm	-0.35	0.17	4.07E-02	7.91E-01
1	25001	IDP_T1_SIENAX_peripheral_grey_normalised_volume	IDP T1:global	Lumbar spine	0.13	0.06	4.07E-02	5.85E-01
278	26655	HippSubfield_rh_volume_GC-ML-DG-body	Hippocampus Subfield	Total body	-0.11	0.05	4.08E-02	1.50E-01
91	25847	IDP_T1_FAST_ROIs_R_front_orb_cortex	IDP T1:unilateral regions	Lumbar spine	0.13	0.06	4.09E-02	1.81E-01
361	26806	aparc-Desikan_lh_volume_parsorbitalis	Desikan Atlas	Total body	-0.11	0.05	4.10E-02	8.07E-02
860	27255	aparc-DKTatlas_rh_area_postcentral	Desikan Atlas	Femoral neck	0.15	0.07	4.11E-02	8.80E-01

932	27389	aparc-a2009s_lh_area_S-oc-temp-med+Lingual	Destrieux Atlas	Heel	-0.07	0.03	4.11E-02	4.06E-01
886	27343	aparc-a2009s_lh_area_G-front-middle	Destrieux Atlas	Total body	-0.09	0.04	4.11E-02	1.32E-01
129	25885	IDP_T1_FAST_ROIs_R_pallidum	IDP T1:unilateral regions	Total body	-0.10	0.05	4.12E-02	8.81E-02
887	27344	aparc-a2009s_lh_area_G-front-sup	Destrieux Atlas	Lumbar spine	-0.11	0.06	4.13E-02	3.31E-01
944	27401	aparc-a2009s_lh_area_S-temporal-sup	Destrieux Atlas	Heel	-0.07	0.03	4.15E-02	4.06E-01
681	26754	aparc-Desikan_lh_area_insula	Desikan Atlas	Heel	-0.07	0.03	4.16E-02	5.04E-01
742	26949	aparc-pial_lh_area_rostralmiddlefrontal	Desikan Atlas	Lumbar spine	-0.11	0.05	4.16E-02	2.81E-01
158	25914	IDP_T1_FAST_ROIs_R_cerebellum_VIIIb	IDP T1:unilateral regions	Forearm	-0.28	0.14	4.17E-02	7.65E-01
122	25878	IDP_T1_FAST_ROIs_L_thalamus	IDP T1:unilateral regions	Lumbar spine	-0.11	0.05	4.17E-02	1.81E-01
670	26743	aparc-Desikan_lh_area_posteriorcingulate	Desikan Atlas	Forearm	-0.34	0.17	4.18E-02	7.96E-01
360	26805	aparc-Desikan_lh_volume_parsopercularis	Desikan Atlas	Total body	-0.11	0.05	4.18E-02	8.11E-02
577	27702	aparc-a2009s_rh_volume_G+S-subcentral	Destrieux Atlas	Total body	-0.10	0.05	4.21E-02	1.22E-01
1196	27421	aparc-a2009s_lh_thickness_G-occipital-middle	Destrieux Atlas	Femoral neck	-0.15	0.07	4.25E-02	2.96E-01
966	27571	aparc-a2009s_rh_area_G-oc-temp-lat-fusifor	Destrieux Atlas	Lumbar spine	-0.14	0.07	4.25E-02	3.31E-01
1313	27686	aparc-a2009s_rh_thickness_S-orbital-lateral	Destrieux Atlas	Femoral neck	-0.19	0.09	4.27E-02	2.96E-01
530	27507	aparc-a2009s_lh_volume_G-rectus	Destrieux Atlas	Total body	-0.12	0.06	4.27E-02	1.22E-01
494	27323	aparc-DKTatlas_rh_volume_superiorfrontal	Desikan Atlas	Lumbar spine	-0.13	0.06	4.28E-02	1.63E-01
703	26843	aparc-Desikan_rh_area_postcentral	Desikan Atlas	Femoral neck	0.14	0.07	4.29E-02	8.80E-01
314	26691	ThalamNuclei_rh_volume_PuM	Thalamus Nuclei	Total body	-0.08	0.04	4.29E-02	1.31E-01
1324	27697	aparc-a2009s_rh_thickness_S-temporal-sup	Destrieux Atlas	Femoral neck	-0.13	0.07	4.30E-02	2.96E-01
316	26693	ThalamNuclei_rh_volume_VPL	Thalamus Nuclei	Lumbar spine	0.11	0.06	4.30E-02	5.69E-01
942	27399	aparc-a2009s_lh_area_S-subparietal	Destrieux Atlas	Total body	-0.09	0.05	4.30E-02	1.35E-01
566	27543	aparc-a2009s_lh_volume_S-postcentral	Destrieux Atlas	Femoral neck	0.18	0.09	4.31E-02	5.70E-01
169	26518	aseg_global_volume_TotalGray	aseg:global	Heel	-0.07	0.04	4.32E-02	9.94E-02
480	27309	aparc-DKTatlas_rh_volume_medialorbitofrontal	Desikan Atlas	Lumbar spine	-0.15	0.07	4.32E-02	1.63E-01
700	26840	aparc-Desikan_rh_area_parsorbitalis	Desikan Atlas	Lumbar spine	-0.12	0.06	4.33E-02	2.83E-01
144	25900	IDP_T1_FAST_ROIs_L_cerebellum_crus_I	IDP T1:unilateral regions	Lumbar spine	0.11	0.05	4.34E-02	1.83E-01
703	26843	aparc-Desikan_rh_area_postcentral	Desikan Atlas	Heel	-0.08	0.04	4.37E-02	5.04E-01
336	26713	ThalamNuclei_rh_volume_LD	Thalamus Nuclei	Lumbar spine	-0.12	0.06	4.38E-02	5.69E-01
621	27746	aparc-a2009s_rh_volume_S-circular-insula-inf	Destrieux Atlas	Femoral neck	-0.14	0.07	4.39E-02	5.70E-01
537	27514	aparc-a2009s_lh_volume_G-temporal-middle	Destrieux Atlas	Forearm	-0.31	0.15	4.39E-02	7.91E-01
830	27163	aparc-DKTatlas_lh_area_posteriorcingulate	Desikan Atlas	Forearm	-0.33	0.17	4.41E-02	7.96E-01
1049	26784	aparc-Desikan_lh_thickness_superiortemporal	Desikan Atlas	Femoral neck	-0.14	0.07	4.42E-02	2.38E-01

1002	27607	aparc-a2009s_rh_area_S-oc-middle+Lunatus	Destrieux Atlas	Heel	0.06	0.03	4.43E-02	4.06E-01
427	27132	BA-exvivo_rh_volume_BA3b	Broadmann Atlas	Total body	-0.09	0.05	4.43E-02	1.11E-01
564	27541	aparc-a2009s_lh_volume_S-parieto-occipital	Destrieux Atlas	Heel	0.06	0.03	4.44E-02	7.07E-01
1185	27410	aparc-a2009s_lh_thickness_G+S-cingul-Mid-Post	Destrieux Atlas	Heel	0.09	0.04	4.44E-02	9.59E-01
755	26962	aparc-pial_rh_area_fusiform	Desikan Atlas	Total body	-0.09	0.04	4.46E-02	9.50E-02
730	26937	aparc-pial_lh_area_middletemporal	Desikan Atlas	Forearm	-0.29	0.15	4.47E-02	7.96E-01
1244	27469	aparc-a2009s_lh_thickness_S-postcentral	Destrieux Atlas	Total body	-0.11	0.05	4.47E-02	7.25E-01
68	25824	IDP_T1_FAST_ROIs_L_latocc_cortex_sup	IDP T1:unilateral regions	Forearm	0.35	0.17	4.48E-02	7.65E-01
918	27375	aparc-a2009s_lh_area_S-circular-insula-ant	Destrieux Atlas	Femoral neck	0.15	0.07	4.50E-02	7.36E-01
501	27478	aparc-a2009s_lh_volume_G+S-occipital-inf	Destrieux Atlas	Forearm	-0.38	0.19	4.50E-02	7.91E-01
101	25857	IDP_T1_FAST_ROIs_R_temp_fusif_cortex_post	IDP T1:unilateral regions	Femoral neck	0.13	0.07	4.51E-02	1.18E-01
67	25823	IDP_T1_FAST_ROIs_R_angular_gyrus	IDP T1:unilateral regions	Femoral neck	0.17	0.08	4.51E-02	1.18E-01
1027	26762	aparc-Desikan_lh_thickness_inferiorparietal	Desikan Atlas	Femoral neck	-0.13	0.07	4.54E-02	2.38E-01
1298	27671	aparc-a2009s_rh_thickness_S-circular-insula-ant	Destrieux Atlas	Heel	0.08	0.04	4.54E-02	9.59E-01
308	26685	ThalamNuclei_lh_volume_Pc	Thalamus Nuclei	Total body	-0.08	0.04	4.54E-02	1.31E-01
817	27150	aparc-DKTatlas_lh_area_isthmuscingulate	Desikan Atlas	Total body	-0.08	0.04	4.56E-02	9.61E-02
56	25812	IDP_T1_FAST_ROIs_L_inf_temp_gyrus_tempocc	IDP T1:unilateral regions	Femoral neck	0.14	0.07	4.57E-02	1.18E-01
1319	27692	aparc-a2009s_rh_thickness_S-precentral-inf-part	Destrieux Atlas	Femoral neck	-0.15	0.08	4.57E-02	2.96E-01
871	27266	aparc-DKTatlas_rh_area_insula	Desikan Atlas	Heel	-0.06	0.03	4.63E-02	5.04E-01
247	26624	HippSubfield_lh_volume_hippocampal-fissure	Hippocampus Subfield	Heel	-0.08	0.04	4.63E-02	8.31E-01
478	27307	aparc-DKTatlas_rh_volume_lateralorbitofrontal	Desikan Atlas	Total body	-0.10	0.05	4.63E-02	8.85E-02
1056	26858	aparc-Desikan_rh_thickness_caudalanteriorcingulate	Desikan Atlas	Femoral neck	-0.15	0.07	4.66E-02	2.38E-01
937	27394	aparc-a2009s_lh_area_S-pericallosal	Destrieux Atlas	Heel	-0.08	0.04	4.66E-02	4.06E-01
385	26898	aparc-Desikan_rh_volume_isthmuscingulate	Desikan Atlas	Lumbar spine	-0.15	0.07	4.70E-02	1.72E-01
1259	27632	aparc-a2009s_rh_thickness_G+S-cingul-Mid-Post	Destrieux Atlas	Femoral neck	-0.16	0.08	4.73E-02	2.96E-01
357	26802	aparc-Desikan_lh_volume_middletemporal	Desikan Atlas	Forearm	-0.29	0.15	4.75E-02	8.69E-01
392	26905	aparc-Desikan_rh_volume_paracentral	Desikan Atlas	Total body	-0.10	0.05	4.75E-02	8.94E-02
760	26967	aparc-pial_rh_area_lateralorbitofrontal	Desikan Atlas	Lumbar spine	-0.14	0.07	4.77E-02	3.00E-01
866	27261	aparc-DKTatlas_rh_area_superiorfrontal	Desikan Atlas	Total body	-0.09	0.04	4.79E-02	9.84E-02
699	26839	aparc-Desikan_rh_area_parsopercularis	Desikan Atlas	Total body	-0.11	0.05	4.80E-02	9.84E-02

999	27604	aparc-a2009s_rh_area_S-front-sup	Destrieux Atlas	Total body	-0.10	0.05	4.80E-02	1.48E-01
737	26944	aparc-pial_lh_area_postcentral	Desikan Atlas	Femoral neck	0.14	0.07	4.81E-02	8.80E-01
863	27258	aparc-DKTatlas_rh_area_precuneus	Desikan Atlas	Total body	-0.09	0.04	4.82E-02	9.84E-02
431	27136	BA-exvivo_rh_volume_BA44	Broadmann Atlas	Lumbar spine	-0.13	0.06	4.84E-02	2.39E-01
637	27762	aparc-a2009s_rh_volume_S-orbital-H-Shaped	Destrieux Atlas	Lumbar spine	-0.13	0.07	4.86E-02	2.22E-01
1026	26761	aparc-Desikan_lh_thickness_fusiform	Desikan Atlas	Total body	-0.10	0.05	4.86E-02	6.29E-01
234	26611	AmygNuclei_rh_volume_Basal-nucleus	Amygdala Nuclei	Total body	-0.09	0.04	4.87E-02	1.95E-01
1233	27458	aparc-a2009s_lh_thickness_S-intrapariet+P-trans	Destrieux Atlas	Total body	-0.10	0.05	4.87E-02	7.25E-01
1054	26856	aparc-Desikan_rh_thickness_GlobalMeanThickness	Desikan Atlas	Femoral neck	-0.13	0.06	4.88E-02	2.38E-01
516	27493	aparc-a2009s_lh_volume_G-Ins-lg+S-cent-ins	Destrieux Atlas	Femoral neck	-0.15	0.07	4.88E-02	5.70E-01
458	27225	aparc-DKTatlas_lh_volume_posteriorcingulate	Desikan Atlas	Forearm	-0.38	0.19	4.88E-02	8.69E-01
300	26677	ThalamNuclei_lh_volume_CeM	Thalamus Nuclei	Heel	-0.06	0.03	4.88E-02	4.92E-01
1279	27652	aparc-a2009s_rh_thickness_G-postcentral	Destrieux Atlas	Femoral neck	-0.14	0.07	4.89E-02	2.96E-01
1292	27665	aparc-a2009s_rh_thickness_Lat-Fis-post	Destrieux Atlas	Lumbar spine	-0.13	0.07	4.90E-02	5.72E-01
730	26937	aparc-pial_lh_area_middletemporal	Desikan Atlas	Lumbar spine	-0.11	0.05	4.91E-02	3.00E-01
962	27567	aparc-a2009s_rh_area_G-Ins-lg+S-cent-ins	Destrieux Atlas	Lumbar spine	-0.14	0.07	4.91E-02	3.63E-01
450	27217	aparc-DKTatlas_lh_volume_middletemporal	Desikan Atlas	Lumbar spine	-0.12	0.06	4.94E-02	1.76E-01
758	26965	aparc-pial_rh_area_isthmuscingulate	Desikan Atlas	Total body	-0.10	0.05	4.96E-02	1.00E-01
991	27596	aparc-a2009s_rh_area_S-cingul-Marginalis	Destrieux Atlas	Total body	-0.09	0.05	4.96E-02	1.48E-01
629	27754	aparc-a2009s_rh_volume_S-intrapariet+P-trans	Destrieux Atlas	Lumbar spine	-0.14	0.07	4.99E-02	2.22E-01
