# Supplementary material for: Causal associations of brain structure with bone mineral density: a large-scale genetic correlation study
Source: Bone Res. 2023 Jul 20;11:37. doi: 10.1038/s41413-023-00270-z (PMC10359275; doi:10.1038/s41413-023-00270-z)
Supplement: Supplementary file 4 — Supplementary Table 2. Primary MR results of BIDPs on BMD (p less than 0.05) [file 41413_2023_270_MOESM4_ESM.pdf]

Supplemental table 2. Primary MR results of BIDPs on BMD (p less than 0.05)

No.	UKBID	IDP.short.name	Category name	Outcome	Method	SNP	OR	BETA	SE	pval	FDR.p
1	25001	IDP_T1_SIENAX_peripheral_grey_normalised_volume	IDP T1:global	Femoral neck	IVW	9	1.28	0.25	0.11	2.26E-02	1.08E-01
1	25001	IDP_T1_SIENAX_peripheral_grey_normalised_volume	IDP T1:global	Heel	IVW	10	1.11	0.1	0.04	5.74E-03	1.09E-01
5	25005	IDP_T1_SIENAX_grey_normalised_volume	IDP T1:global	Forearm	IVW	7	2.12	0.75	0.37	4.51E-02	4.08E-01
5	25005	IDP_T1_SIENAX_grey_normalised_volume	IDP T1:global	Femoral neck	IVW	7	1.51	0.41	0.12	8.14E-04	7.73E-03
7	25007	IDP_T1_SIENAX_white_normalised_volume	IDP T1:global	Femoral neck	IVW	14	1.22	0.2	0.07	3.55E-03	2.25E-02
7	25007	IDP_T1_SIENAX_white_normalised_volume	IDP T1:global	Lumbar spine	IVW	14	1.22	0.2	0.08	1.67E-02	3.17E-01
9	25009	IDP_T1_SIENAX_brain-normalised_volume	IDP T1:global	Femoral neck	IVW	9	1.30	0.26	0.07	5.85E-04	7.73E-03
11	25011	IDP_T1_FIRST_left_thalamus_volume	IDP T1:global	Femoral neck	IVW	11	1.09	0.09	0.05	4.75E-02	1.50E-01
11	25011	IDP_T1_FIRST_left_thalamus_volume	IDP T1:global	Heel	IVW	9	1.04	0.04	0.02	3.12E-02	2.92E-01
13	25013	IDP_T1_FIRST_left_caudate_volume	IDP T1:global	Femoral neck	IVW	19	0.90	-0.1	0.05	4.41E-02	1.50E-01
23	25023	IDP_T1_FIRST_left_accumbens_volume	IDP T1:global	Forearm	IVW	3	1.46	0.38	0.19	4.15E-02	4.08E-01
27	25783	IDP_T1_FAST_ROIs_R_frontal_pole	IDP T1:unilateral regions	Heel	WR	1	1.12	0.11	0.04	1.07E-02	1.00E-01
36	25792	IDP_T1_FAST_ROIs_L_inf_front_gyrus_parsop	IDP T1:unilateral regions	Femoral neck	WR	1	1.95	0.67	0.16	2.33E-05	3.05E-03
36	25792	IDP_T1_FAST_ROIs_L_inf_front_gyrus_parsop	IDP T1:unilateral regions	Lumbar spine	WR	1	1.77	0.57	0.18	1.92E-03	9.45E-02
36	25792	IDP_T1_FAST_ROIs_L_inf_front_gyrus_parsop	IDP T1:unilateral regions	Total body	WR	1	1.68	0.52	0.12	2.57E-05	3.44E-03
36	25792	IDP_T1_FAST_ROIs_L_inf_front_gyrus_parsop	IDP T1:unilateral regions	Heel	WR	1	1.55	0.44	0.04	1.00E-28	1.31E-26
42	25798	IDP_T1_FAST_ROIs_L_sup_temp_gyrus_ant	IDP T1:unilateral regions	Femoral neck	IVW	2	0.76	-0.28	0.12	2.05E-02	3.21E-01
42	25798	IDP_T1_FAST_ROIs_L_sup_temp_gyrus_ant	IDP T1:unilateral regions	Lumbar spine	IVW	2	0.72	-0.33	0.13	1.37E-02	4.36E-01
43	25799	IDP_T1_FAST_ROIs_R_sup_temp_gyrus_ant	IDP T1:unilateral regions	Heel	WR	1	1.08	0.08	0.03	7.12E-03	7.18E-02
44	25800	IDP_T1_FAST_ROIs_L_sup_temp_gyrus_post	IDP T1:unilateral regions	Heel	WR	1	1.12	0.11	0.04	5.35E-03	5.84E-02
47	25803	IDP_T1_FAST_ROIs_R_mid_temp_gyrus_ant	IDP T1:unilateral regions	Heel	WR	1	1.17	0.16	0.04	1.37E-04	2.12E-03

48	25804	IDP_T1_FAST_ROIs_L_mid_temp_gyrus_post	IDP T1:unilateral regions	Heel	WR	1	1.31	0.27	0.04	5.03E-10	2.64E-08
51	25807	IDP_T1_FAST_ROIs_R_mid_temp_gyrus_tempocc	IDP T1:unilateral regions	Heel	WR	1	1.30	0.26	0.04	1.69E-09	5.53E-08
52	25808	IDP_T1_FAST_ROIs_L_inf_temp_gyrus_ant	IDP T1:unilateral regions	Heel	WR	1	1.17	0.16	0.04	1.37E-04	2.12E-03
55	25811	IDP_T1_FAST_ROIs_R_inf_temp_gyrus_post	IDP T1:unilateral regions	Heel	IVW	2	1.20	0.18	0.04	6.88E-05	1.80E-03
55	25811	IDP_T1_FAST_ROIs_R_inf_temp_gyrus_post	IDP T1:unilateral regions	Total body	IVW	2	1.20	0.18	0.09	4.28E-02	7.89E-01
66	25822	IDP_T1_FAST_ROIs_L_angular_gyrus	IDP T1:unilateral regions	Total body	IVW	5	0.87	-0.14	0.06	1.85E-02	7.89E-01
70	25826	IDP_T1_FAST_ROIs_L_latocc_cortex_inf	IDP T1:unilateral regions	Heel	WR	1	1.26	0.23	0.04	5.03E-10	2.64E-08
74	25830	IDP_T1_FAST_ROIs_L_front_med_cortex	IDP T1:unilateral regions	Total body	IVW	2	1.23	0.21	0.09	2.09E-02	7.89E-01
75	25831	IDP_T1_FAST_ROIs_R_front_med_cortex	IDP T1:unilateral regions	Lumbar spine	WR	1	1.45	0.37	0.17	2.91E-02	5.45E-01
75	25831	IDP_T1_FAST_ROIs_R_front_med_cortex	IDP T1:unilateral regions	Femoral neck	WR	1	1.45	0.37	0.15	1.14E-02	3.08E-01
79	25835	IDP_T1_FAST_ROIs_R_subcallosal_cortex	IDP T1:unilateral regions	Femoral neck	IVW	12	1.15	0.14	0.06	1.41E-02	3.08E-01
82	25838	IDP_T1_FAST_ROIs_L_cing_gyrus_ant	IDP T1:unilateral regions	Total body	WR	1	1.36	0.31	0.15	3.83E-02	7.89E-01
82	25838	IDP_T1_FAST_ROIs_L_cing_gyrus_ant	IDP T1:unilateral regions	Heel	WR	1	1.19	0.17	0.04	9.87E-05	2.12E-03
83	25839	IDP_T1_FAST_ROIs_R_cing_gyrus_ant	IDP T1:unilateral regions	Heel	IVW	2	1.14	0.13	0.03	1.23E-04	2.12E-03
85	25841	IDP_T1_FAST_ROIs_R_cing_gyrus_post	IDP T1:unilateral regions	Lumbar spine	IVW	4	0.79	-0.23	0.10	2.06E-02	4.50E-01
89	25845	IDP_T1_FAST_ROIs_R_cuneal_cortex	IDP T1:unilateral regions	Heel	IVW	4	0.96	-0.04	0.02	3.94E-02	2.46E-01
90	25846	IDP_T1_FAST_ROIs_L_front_orb_cortex	IDP T1:unilateral	Femoral neck	IVW	8	1.39	0.33	0.14	2.20E-02	3.21E-01

			regions								
91	25847	IDP_T1_FAST_ROIs_R_front_orb_cortex	IDP T1:unilateral regions	Femoral neck	IVW	9	1.32	0.28	0.13	2.89E-02	3.63E-01
93	25849	IDP_T1_FAST_ROIs_R_parahipp_gyrus_ant	IDP T1:unilateral regions	Femoral neck	IVW	2	1.90	0.64	0.26	1.35E-02	3.08E-01
93	25849	IDP_T1_FAST_ROIs_R_parahipp_gyrus_ant	IDP T1:unilateral regions	Lumbar spine	IVW	2	1.80	0.59	0.15	6.24E-05	8.17E-03
96	25852	IDP_T1_FAST_ROIs_L_lingual_gyrus	IDP T1:unilateral regions	Femoral neck	IVW	5	1.16	0.15	0.07	4.62E-02	4.33E-01
103	25859	IDP_T1_FAST_ROIs_R_temp_occ_fusif_cortex	IDP T1:unilateral regions	Heel	IVW	5	1.05	0.05	0.02	1.70E-02	1.31E-01
109	25865	IDP_T1_FAST_ROIs_R_cent_operc_cortex	IDP T1:unilateral regions	Femoral neck	IVW	2	1.55	0.44	0.12	1.93E-04	1.26E-02
120	25876	IDP_T1_FAST_ROIs_L_occ_pole	IDP T1:unilateral regions	Heel	IVW	6	0.94	-0.06	0.03	1.59E-02	1.30E-01
121	25877	IDP_T1_FAST_ROIs_R_occ_pole	IDP T1:unilateral regions	Lumbar spine	IVW	12	1.16	0.15	0.06	1.67E-02	4.36E-01
124	25880	IDP_T1_FAST_ROIs_L_caudate	IDP T1:unilateral regions	Heel	IVW	16	0.92	-0.08	0.03	4.04E-03	4.81E-02
125	25881	IDP_T1_FAST_ROIs_R_caudate	IDP T1:unilateral regions	Heel	IVW	17	0.96	-0.04	0.02	2.05E-02	1.49E-01
127	25883	IDP_T1_FAST_ROIs_R_putamen	IDP T1:unilateral regions	Heel	IVW	20	0.96	-0.04	0.01	1.20E-02	1.05E-01
129	25885	IDP_T1_FAST_ROIs_R_pallidum	IDP T1:unilateral regions	Femoral neck	IVW	3	0.83	-0.19	0.09	3.78E-02	4.13E-01
133	25889	IDP_T1_FAST_ROIs_R_amygdala	IDP T1:unilateral regions	Femoral neck	IVW	3	1.27	0.24	0.11	3.05E-02	3.63E-01
134	25890	IDP_T1_FAST_ROIs_L_ventral_striatum	IDP T1:unilateral regions	Lumbar spine	IVW	17	1.06	0.06	0.03	3.64E-02	5.95E-01
137	25893	IDP_T1_FAST_ROIs_L_cerebellum_I-IV	IDP T1:unilateral regions	Heel	IVW	9	0.96	-0.04	0.02	3.69E-02	2.42E-01
139	25895	IDP_T1_FAST_ROIs_L_cerebellum_V	IDP T1:unilateral regions	Heel	IVW	14	0.94	-0.06	0.03	2.56E-02	1.76E-01

157	25913	IDP_T1_FAST_ROIs_V_cerebellum_VIIIb	IDP T1:unilateral regions	Heel	IVW	18	0.96	-0.04	0.01	1.73E-03	2.26E-02
160	25916	IDP_T1_FAST_ROIs_V_cerebellum_IX	IDP T1:unilateral regions	Femoral neck	IVW	15	1.06	0.06	0.03	4.41E-02	4.33E-01
162	25918	IDP_T1_FAST_ROIs_L_cerebellum_X	IDP T1:unilateral regions	Femoral neck	IVW	9	1.34	0.29	0.09	2.08E-03	9.09E-02
162	25918	IDP_T1_FAST_ROIs_L_cerebellum_X	IDP T1:unilateral regions	Lumbar spine	IVW	9	1.30	0.26	0.08	2.16E-03	9.45E-02
164	25920	IDP_T1_FAST_ROIs_R_cerebellum_X	IDP T1:unilateral regions	Total body	IVW	12	1.42	0.35	0.18	4.68E-02	7.89E-01
164	25920	IDP_T1_FAST_ROIs_R_cerebellum_X	IDP T1:unilateral regions	Femoral neck	IVW	12	1.22	0.2	0.08	1.66E-02	3.10E-01
165	26514	aseg_global_volume_BrainSeg	aseg:global	Total body	IVW	16	0.72	-0.33	0.12	6.70E-03	3.01E-02
166	26515	aseg_global_volume_BrainSegNotVent	aseg:global	Total body	IVW	14	0.68	-0.39	0.12	1.26E-03	1.45E-02
167	26516	aseg_global_volume_BrainSegNotVentSurf	aseg:global	Total body	IVW	14	0.71	-0.34	0.12	4.43E-03	3.01E-02
169	26518	aseg_global_volume_TotalGray	aseg:global	Total body	IVW	10	0.66	-0.41	0.15	5.82E-03	3.01E-02
170	26519	aseg_global_volume_SupraTentorial	aseg:global	Total body	IVW	13	0.76	-0.27	0.12	2.24E-02	6.90E-02
171	26520	aseg_global_volume_SupraTentorialNotVent	aseg:global	Total body	IVW	12	0.75	-0.29	0.14	3.56E-02	9.10E-02
172	26521	aseg_global_volume_EstimatedTotalIntraCranial	aseg:global	Heel	IVW	6	2.29	0.83	0.30	5.47E-03	6.29E-02
172	26521	aseg_global_volume_EstimatedTotalIntraCranial	aseg:global	Forearm	IVW	7	2.25	0.81	0.30	5.95E-03	6.84E-02
172	26521	aseg_global_volume_EstimatedTotalIntraCranial	aseg:global	Total body	IVW	7	1.84	0.61	0.23	7.85E-03	3.01E-02
172	26521	aseg_global_volume_EstimatedTotalIntraCranial	aseg:global	Lumbar spine	IVW	7	1.45	0.37	0.13	5.10E-03	5.87E-02
172	26521	aseg_global_volume_EstimatedTotalIntraCranial	aseg:global	Femoral neck	IVW	7	1.42	0.35	0.09	1.92E-04	2.20E-03
179	26528	aseg_global_volume_WM-hypointensities	aseg:global	Total body	IVW	13	0.93	-0.07	0.03	2.40E-02	6.90E-02
184	26533	aseg_global_volume_CC-Central	aseg:global	Heel	IVW	8	1.03	0.03	0.01	3.29E-02	2.52E-01
184	26533	aseg_global_volume_CC-Central	aseg:global	Lumbar spine	IVW	8	0.85	-0.16	0.07	2.53E-02	1.94E-01
185	26534	aseg_global_volume_CC-Mid-Anterior	aseg:global	Forearm	IVW	13	0.84	-0.17	0.09	4.92E-02	3.77E-01
187	26536	aseg_global_volume-ratio_BrainSegVol-to-eTIV	aseg:global	Femoral neck	IVW	15	1.50	0.40	0.08	7.35E-08	1.69E-06
187	26536	aseg_global_volume-ratio_BrainSegVol-to-eTIV	aseg:global	Lumbar spine	IVW	15	1.69	0.53	0.09	4.52E-09	1.04E-07
187	26536	aseg_global_volume-ratio_BrainSegVol-to-eTIV	aseg:global	Forearm	IVW	15	1.96	0.67	0.12	1.11E-08	2.55E-07
187	26536	aseg_global_volume-ratio_BrainSegVol-to-eTIV	aseg:global	Heel	IVW	15	2.01	0.70	0.11	3.04E-10	6.99E-09
187	26536	aseg_global_volume-ratio_BrainSegVol-to-eTIV	aseg:global	Total body	IVW	17	2.29	0.83	0.08	4.00E-28	9.20E-27
189	26552	aseg_lh_volume_Cortex	aseg:unilateral	Heel	IVW	5	0.79	-0.24	0.11	2.87E-02	5.82E-01

			regions								
189	26552	aseg_lh_volume_Cortex	aseg:unilateral regions	Lumbar spine	IVW	5	0.73	-0.32	0.11	3.73E-03	7.51E-02
189	26552	aseg_lh_volume_Cortex	aseg:unilateral regions	Total body	IVW	5	0.68	-0.39	0.16	1.68E-02	1.90E-01
194	26557	aseg_lh_volume_Cerebellum-Cortex	aseg:unilateral regions	Total body	IVW	25	0.93	-0.07	0.02	6.18E-03	1.05E-01
199	26562	aseg_lh_volume_Hippocampus	aseg:unilateral regions	Forearm	IVW	12	0.87	-0.14	0.07	4.95E-02	8.87E-01
202	26565	aseg_lh_volume_VentralDC	aseg:unilateral regions	Total body	IVW	17	1.08	0.08	0.04	2.38E-02	2.02E-01
204	26567	aseg_lh_volume_choroid-plexus	aseg:unilateral regions	Femoral neck	IVW	10	1.15	0.14	0.05	8.62E-03	2.85E-01
206	26583	aseg_rh_volume_Cortex	aseg:unilateral regions	Lumbar spine	IVW	4	0.74	-0.3	0.11	4.55E-03	7.51E-02
213	26590	aseg_rh_volume_Caudate	aseg:unilateral regions	Femoral neck	IVW	20	0.91	-0.09	0.04	2.40E-02	3.95E-01
219	26596	aseg_rh_volume_VentralDC	aseg:unilateral regions	Total body	IVW	17	1.11	0.1	0.03	2.50E-03	8.49E-02
219	26596	aseg_rh_volume_VentralDC	aseg:unilateral regions	Femoral neck	IVW	16	1.09	0.09	0.04	3.99E-02	4.39E-01
219	26596	aseg_rh_volume_VentralDC	aseg:unilateral regions	Heel	IVW	16	1.05	0.05	0.02	3.45E-02	5.82E-01
225	26602	AmygNuclei_lh_volume_Accessory-Basal-nucleus	Amygdala Nuclei	Heel	IVW	3	0.91	-0.09	0.03	7.51E-03	5.00E-02
227	26604	AmygNuclei_lh_volume_Central-nucleus	Amygdala Nuclei	Heel	IVW	2	0.93	-0.07	0.01	2.05E-06	4.10E-05
229	26606	AmygNuclei_lh_volume_Cortical-nucleus	Amygdala Nuclei	Total body	IVW	3	1.12	0.11	0.05	4.22E-02	6.47E-01
229	26606	AmygNuclei_lh_volume_Cortical-nucleus	Amygdala Nuclei	Heel	IVW	3	0.93	-0.07	0.02	5.78E-04	5.78E-03
230	26607	AmygNuclei_lh_volume_Corticoamygdaloid-transitio	Amygdala Nuclei	Forearm	IVW	4	0.68	-0.39	0.16	1.51E-02	3.01E-01
244	26621	HippSubfield_lh_volume_subiculum-body	Hippocampus Subfield	Femoral neck	IVW	11	1.14	0.13	0.04	2.64E-03	1.13E-01
245	26622	HippSubfield_lh_volume_CA1-body	Hippocampus Subfield	Heel	IVW	5	0.97	-0.03	0.02	4.36E-02	9.75E-01
247	26624	HippSubfield_lh_volume_hippocampal-fissure	Hippocampus	Total body	IVW	3	1.15	0.14	0.07	3.57E-02	7.06E-01

			Subfield								
249	26626	HippSubfield_lh_volume_CA1-head	Hippocampus Subfield	Forearm	IVW	12	0.83	-0.19	0.07	1.14E-02	4.89E-01
252	26629	HippSubfield_lh_volume_molecular-layer-HP-head	Hippocampus Subfield	Forearm	IVW	8	0.79	-0.24	0.11	2.62E-02	5.63E-01
260	26637	HippSubfield_lh_volume_CA3-head	Hippocampus Subfield	Femoral neck	IVW	6	1.12	0.11	0.05	3.52E-02	3.50E-01
268	26645	HippSubfield_rh_volume_subiculum-head	Hippocampus Subfield	Femoral neck	IVW	7	1.13	0.12	0.05	1.44E-02	2.06E-01
275	26652	HippSubfield_rh_volume_molecular-layer-HP-body	Hippocampus Subfield	Lumbar spine	IVW	6	0.80	-0.22	0.08	6.36E-03	2.74E-01
278	26655	HippSubfield_rh_volume_GC-ML-DG-body	Hippocampus Subfield	Femoral neck	IVW	8	1.13	0.12	0.05	1.40E-02	2.06E-01
280	26657	HippSubfield_rh_volume_CA4-body	Hippocampus Subfield	Femoral neck	IVW	9	1.09	0.09	0.05	4.31E-02	3.50E-01
285	26662	HippSubfield_rh_volume_Whole-hippocampal-head	Hippocampus Subfield	Femoral neck	IVW	8	1.08	0.08	0.04	4.89E-02	3.50E-01
294	26671	ThalamNuclei_lh_volume_VLa	Thalamus Nuclei	Total body	IVW	14	0.91	-0.09	0.04	2.01E-02	5.42E-01
297	26674	ThalamNuclei_lh_volume_Pf	Thalamus Nuclei	Forearm	IVW	11	1.21	0.19	0.09	4.31E-02	8.77E-01
300	26677	ThalamNuclei_lh_volume_CeM	Thalamus Nuclei	Lumbar spine	IVW	8	1.28	0.25	0.08	1.37E-03	6.98E-02
301	26678	ThalamNuclei_lh_volume_VA	Thalamus Nuclei	Heel	IVW	12	0.94	-0.06	0.03	3.49E-02	2.68E-01
305	26682	ThalamNuclei_lh_volume_PuL	Thalamus Nuclei	Heel	WR	1	0.84	-0.17	0.04	1.46E-05	2.67E-04
307	26684	ThalamNuclei_lh_volume_AV	Thalamus Nuclei	Heel	IVW	2	0.87	-0.14	0.06	1.99E-02	2.59E-01
311	26688	ThalamNuclei_rh_volume_LGN	Thalamus Nuclei	Heel	WR	1	0.84	-0.17	0.04	1.54E-05	2.67E-04
312	26689	ThalamNuclei_rh_volume_MGN	Thalamus Nuclei	Heel	IVW	2	1.11	0.1	0.02	9.70E-07	5.04E-05
320	26697	ThalamNuclei_rh_volume_MDm	Thalamus Nuclei	Lumbar spine	IVW	4	0.83	-0.19	0.09	3.23E-02	7.06E-01
321	26698	ThalamNuclei_rh_volume_Pf	Thalamus Nuclei	Total body	IVW	9	1.11	0.1	0.05	4.04E-02	5.42E-01
321	26698	ThalamNuclei_rh_volume_Pf	Thalamus Nuclei	Heel	IVW	7	1.09	0.09	0.04	4.13E-02	2.68E-01
324	26701	ThalamNuclei_rh_volume_VA	Thalamus Nuclei	Heel	IVW	8	0.95	-0.05	0.03	3.69E-02	2.68E-01
327	26704	ThalamNuclei_rh_volume_VM	Thalamus Nuclei	Heel	IVW	9	1.05	0.05	0.02	3.36E-02	2.68E-01
328	26705	ThalamNuclei_rh_volume_PuL	Thalamus Nuclei	Total body	IVW	4	0.88	-0.13	0.06	3.84E-02	5.42E-01
336	26713	ThalamNuclei_rh_volume_LD	Thalamus Nuclei	Total body	IVW	10	1.14	0.13	0.07	4.62E-02	5.42E-01
648	26721	aparc-Desikan_lh_area_TotalSurface	Desikan Atlas	Forearm	IVW	14	0.81	-0.21	0.08	7.85E-03	4.94E-01

651	26724	aparc-Desikan_lh_area_caudalmiddlefrontal	Desikan Atlas	Forearm	IVW	3	1.54	0.43	0.16	5.46E-03	4.94E-01
651	26724	aparc-Desikan_lh_area_caudalmiddlefrontal	Desikan Atlas	Femoral neck	IVW	3	1.30	0.26	0.12	2.52E-02	4.73E-01
651	26724	aparc-Desikan_lh_area_caudalmiddlefrontal	Desikan Atlas	Total body	IVW	3	1.27	0.24	0.09	5.05E-03	2.38E-01
651	26724	aparc-Desikan_lh_area_caudalmiddlefrontal	Desikan Atlas	Lumbar spine	IVW	3	1.23	0.21	0.09	2.49E-02	3.14E-01
654	26727	aparc-Desikan_lh_area_fusiform	Desikan Atlas	Femoral neck	IVW	3	1.21	0.19	0.09	2.95E-02	4.73E-01
656	26729	aparc-Desikan_lh_area_inferiortemporal	Desikan Atlas	Lumbar spine	WR	1	2.10	0.74	0.26	4.05E-03	1.14E-01
656	26729	aparc-Desikan_lh_area_inferiortemporal	Desikan Atlas	Heel	WR	1	1.55	0.44	0.04	7.69E-26	4.82E-24
656	26729	aparc-Desikan_lh_area_inferiortemporal	Desikan Atlas	Total body	WR	1	1.42	0.35	0.15	2.28E-02	6.12E-01
659	26732	aparc-Desikan_lh_area_lateralorbitofrontal	Desikan Atlas	Forearm	IVW	13	1.30	0.26	0.09	5.22E-03	4.94E-01
659	26732	aparc-Desikan_lh_area_lateralorbitofrontal	Desikan Atlas	Femoral neck	IVW	13	1.12	0.11	0.04	1.62E-02	4.41E-01
661	26734	aparc-Desikan_lh_area_medialorbitofrontal	Desikan Atlas	Heel	WR	1	0.61	-0.5	0.04	1.45E-34	2.73E-32
661	26734	aparc-Desikan_lh_area_medialorbitofrontal	Desikan Atlas	Lumbar spine	WR	1	0.53	-0.63	0.19	1.10E-03	6.57E-02
661	26734	aparc-Desikan_lh_area_medialorbitofrontal	Desikan Atlas	Total body	WR	1	0.39	-0.95	0.13	6.38E-14	1.20E-11
670	26743	aparc-Desikan_lh_area_posteriorcingulate	Desikan Atlas	Heel	IVW	2	0.82	-0.2	0.06	4.55E-04	1.03E-02
671	26744	aparc-Desikan_lh_area_precentral	Desikan Atlas	Femoral neck	IVW	6	1.08	0.08	0.04	4.61E-02	5.65E-01
673	26746	aparc-Desikan_lh_area_rostralanteriorcingulate	Desikan Atlas	Heel	WR	1	1.08	0.08	0.04	3.77E-02	4.16E-01
674	26747	aparc-Desikan_lh_area_rostralmiddlefrontal	Desikan Atlas	Lumbar spine	IVW	4	0.77	-0.26	0.10	6.71E-03	1.43E-01
674	26747	aparc-Desikan_lh_area_rostralmiddlefrontal	Desikan Atlas	Forearm	IVW	4	0.68	-0.39	0.17	2.20E-02	6.43E-01
679	26752	aparc-Desikan_lh_area_frontalpole	Desikan Atlas	Heel	WR	1	0.89	-0.12	0.03	5.34E-05	1.43E-03
1020	26755	aparc-Desikan_lh_thickness_GlobalMeanThickness	Desikan Atlas	Lumbar spine	IVW	12	0.86	-0.15	0.07	3.81E-02	2.00E-01
1026	26761	aparc-Desikan_lh_thickness_fusiform	Desikan Atlas	Lumbar spine	IVW	4	0.82	-0.2	0.09	2.87E-02	1.65E-01
1028	26763	aparc-Desikan_lh_thickness_inferiortemporal	Desikan Atlas	Total body	IVW	4	0.86	-0.15	0.06	1.47E-02	1.54E-01
1028	26763	aparc-Desikan_lh_thickness_inferiortemporal	Desikan Atlas	Heel	IVW	4	0.85	-0.16	0.07	1.48E-02	2.33E-01
1028	26763	aparc-Desikan_lh_thickness_inferiortemporal	Desikan Atlas	Lumbar spine	IVW	4	0.64	-0.44	0.09	1.61E-06	1.01E-04
1028	26763	aparc-Desikan_lh_thickness_inferiortemporal	Desikan Atlas	Forearm	IVW	4	0.58	-0.54	0.18	2.27E-03	1.43E-01
1030	26765	aparc-Desikan_lh_thickness_lateraloccipital	Desikan Atlas	Lumbar spine	IVW	8	0.81	-0.21	0.10	2.87E-02	1.65E-01
1032	26767	aparc-Desikan_lh_thickness_lingual	Desikan Atlas	Heel	IVW	4	1.11	0.1	0.02	1.98E-05	4.16E-04
1033	26768	aparc-Desikan_lh_thickness_medialorbitofrontal	Desikan Atlas	Femoral neck	WR	1	0.66	-0.41	0.19	3.65E-02	4.08E-01
1033	26768	aparc-Desikan_lh_thickness_medialorbitofrontal	Desikan Atlas	Heel	WR	1	0.66	-0.41	0.04	9.89E-26	3.12E-24
1033	26768	aparc-Desikan_lh_thickness_medialorbitofrontal	Desikan Atlas	Lumbar spine	WR	1	0.43	-0.85	0.25	5.36E-04	1.37E-02
1038	26773	aparc-Desikan_lh_thickness_parsorbitalis	Desikan Atlas	Heel	WR	1	0.64	-0.45	0.04	7.20E-26	3.12E-24
1038	26773	aparc-Desikan_lh_thickness_parsorbitalis	Desikan Atlas	Total body	WR	1	0.59	-0.53	0.18	4.19E-03	8.74E-02
1038	26773	aparc-Desikan_lh_thickness_parsorbitalis	Desikan Atlas	Lumbar spine	WR	1	0.39	-0.95	0.28	6.53E-04	1.37E-02

1039	26774	aparc-Desikan_lh_thickness_parstriangularis	Desikan Atlas	Femoral neck	IVW	4	0.84	-0.18	0.09	3.89E-02	4.08E-01
1042	26777	aparc-Desikan_lh_thickness_posteriorcingulate	Desikan Atlas	Total body	IVW	2	0.83	-0.19	0.09	3.96E-02	2.31E-01
1044	26779	aparc-Desikan_lh_thickness_precuneus	Desikan Atlas	Total body	IVW	8	0.91	-0.09	0.05	4.53E-02	2.38E-01
1044	26779	aparc-Desikan_lh_thickness_precuneus	Desikan Atlas	Femoral neck	IVW	8	0.88	-0.13	0.06	1.81E-02	4.08E-01
1044	26779	aparc-Desikan_lh_thickness_precuneus	Desikan Atlas	Lumbar spine	IVW	8	0.84	-0.18	0.08	1.47E-02	1.46E-01
1046	26781	aparc-Desikan_lh_thickness_rostralmiddlefrontal	Desikan Atlas	Femoral neck	IVW	9	0.89	-0.12	0.06	3.62E-02	4.08E-01
1046	26781	aparc-Desikan_lh_thickness_rostralmiddlefrontal	Desikan Atlas	Lumbar spine	IVW	9	0.86	-0.15	0.06	1.82E-02	1.46E-01
1053	26788	aparc-Desikan_lh_thickness_insula	Desikan Atlas	Forearm	IVW	2	0.56	-0.58	0.29	4.32E-02	5.44E-01
346	26791	aparc-Desikan_lh_volume_caudalmiddlefrontal	Desikan Atlas	Femoral neck	IVW	6	1.32	0.28	0.12	1.77E-02	3.83E-01
346	26791	aparc-Desikan_lh_volume_caudalmiddlefrontal	Desikan Atlas	Total body	IVW	6	1.25	0.22	0.09	1.49E-02	3.16E-01
350	26795	aparc-Desikan_lh_volume_inferiorparietal	Desikan Atlas	Heel	IVW	4	0.91	-0.09	0.05	4.15E-02	3.23E-01
351	26796	aparc-Desikan_lh_volume_inferiortemporal	Desikan Atlas	Heel	WR	1	1.15	0.14	0.04	8.67E-04	1.07E-02
354	26799	aparc-Desikan_lh_volume_lateralorbitofrontal	Desikan Atlas	Femoral neck	IVW	5	1.32	0.28	0.08	8.90E-04	1.04E-01
354	26799	aparc-Desikan_lh_volume_lateralorbitofrontal	Desikan Atlas	Heel	IVW	5	1.07	0.07	0.03	3.33E-02	3.00E-01
356	26801	aparc-Desikan_lh_volume_medialorbitofrontal	Desikan Atlas	Lumbar spine	WR	1	1.84	0.61	0.20	2.11E-03	2.47E-01
356	26801	aparc-Desikan_lh_volume_medialorbitofrontal	Desikan Atlas	Heel	WR	1	1.60	0.47	0.04	3.00E-28	3.51E-26
356	26801	aparc-Desikan_lh_volume_medialorbitofrontal	Desikan Atlas	Total body	WR	1	1.51	0.41	0.13	1.61E-03	9.41E-02
356	26801	aparc-Desikan_lh_volume_medialorbitofrontal	Desikan Atlas	Femoral neck	WR	1	1.45	0.37	0.17	2.97E-02	3.83E-01
360	26805	aparc-Desikan_lh_volume_parsopercularis	Desikan Atlas	Femoral neck	WR	1	0.81	-0.21	0.10	3.60E-02	3.83E-01
373	26818	aparc-Desikan_lh_volume_supramarginal	Desikan Atlas	Lumbar spine	IVW	5	1.20	0.18	0.09	4.55E-02	5.07E-01
682	26822	aparc-Desikan_rh_area_TotalSurface	Desikan Atlas	Forearm	IVW	16	0.83	-0.19	0.08	1.73E-02	6.43E-01
688	26828	aparc-Desikan_rh_area_fusiform	Desikan Atlas	Heel	IVW	4	1.16	0.15	0.05	1.65E-03	2.52E-02
688	26828	aparc-Desikan_rh_area_fusiform	Desikan Atlas	Total body	IVW	4	1.12	0.11	0.06	4.98E-02	7.27E-01
691	26831	aparc-Desikan_rh_area_isthmuscingulate	Desikan Atlas	Heel	IVW	5	0.93	-0.07	0.02	2.22E-03	2.98E-02
696	26836	aparc-Desikan_rh_area_middletemporal	Desikan Atlas	Forearm	IVW	6	1.34	0.29	0.14	3.65E-02	6.43E-01
698	26838	aparc-Desikan_rh_area_paracentral	Desikan Atlas	Forearm	IVW	3	0.66	-0.41	0.19	3.45E-02	6.43E-01
707	26847	aparc-Desikan_rh_area_rostralanteriorcingulate	Desikan Atlas	Heel	IVW	2	1.17	0.16	0.03	4.56E-10	1.71E-08
708	26848	aparc-Desikan_rh_area_rostralmiddlefrontal	Desikan Atlas	Femoral neck	IVW	5	0.85	-0.16	0.07	2.11E-02	4.73E-01
712	26852	aparc-Desikan_rh_area_supramarginal	Desikan Atlas	Lumbar spine	IVW	9	1.11	0.1	0.05	3.34E-02	3.71E-01
713	26853	aparc-Desikan_rh_area_frontalpole	Desikan Atlas	Lumbar spine	WR	1	0.66	-0.41	0.19	3.18E-02	3.71E-01
714	26854	aparc-Desikan_rh_area_transversetemporal	Desikan Atlas	Total body	IVW	2	1.21	0.19	0.08	2.81E-02	6.61E-01
1054	26856	aparc-Desikan_rh_thickness_GlobalMeanThickness	Desikan Atlas	Total body	IVW	15	0.90	-0.1	0.03	5.55E-03	8.74E-02
1054	26856	aparc-Desikan_rh_thickness_GlobalMeanThickness	Desikan Atlas	Lumbar spine	IVW	13	0.88	-0.13	0.06	2.28E-02	1.60E-01

1056	26858	aparc-Desikan_rh_thickness_caudalanteriorcingulate	Desikan Atlas	Heel	WR	1	1.08	0.08	0.04	3.00E-02	2.70E-01
1060	26862	aparc-Desikan_rh_thickness_fusiform	Desikan Atlas	Total body	IVW	5	0.85	-0.16	0.07	1.34E-02	1.54E-01
1060	26862	aparc-Desikan_rh_thickness_fusiform	Desikan Atlas	Lumbar spine	IVW	5	0.72	-0.33	0.11	2.65E-03	4.17E-02
1060	26862	aparc-Desikan_rh_thickness_fusiform	Desikan Atlas	Forearm	IVW	5	0.65	-0.43	0.17	1.29E-02	2.03E-01
1062	26864	aparc-Desikan_rh_thickness_inferiortemporal	Desikan Atlas	Total body	IVW	8	0.84	-0.17	0.05	2.29E-04	7.22E-03
1063	26865	aparc-Desikan_rh_thickness_isthmuscingulate	Desikan Atlas	Total body	IVW	4	1.27	0.24	0.06	1.74E-04	7.22E-03
1064	26866	aparc-Desikan_rh_thickness_lateraloccipital	Desikan Atlas	Total body	IVW	11	0.93	-0.07	0.03	4.03E-02	2.31E-01
1065	26867	aparc-Desikan_rh_thickness_lateralorbitofrontal	Desikan Atlas	Total body	IVW	4	0.87	-0.14	0.06	2.32E-02	1.81E-01
1066	26868	aparc-Desikan_rh_thickness_lingual	Desikan Atlas	Lumbar spine	IVW	6	0.86	-0.15	0.07	1.85E-02	1.46E-01
1068	26870	aparc-Desikan_rh_thickness_middletemporal	Desikan Atlas	Femoral neck	IVW	4	0.83	-0.19	0.08	2.12E-02	4.08E-01
1068	26870	aparc-Desikan_rh_thickness_middletemporal	Desikan Atlas	Lumbar spine	IVW	4	0.73	-0.32	0.13	1.68E-02	1.46E-01
1069	26871	aparc-Desikan_rh_thickness_parahippocampal	Desikan Atlas	Total body	IVW	3	1.19	0.17	0.07	2.59E-02	1.81E-01
1076	26878	aparc-Desikan_rh_thickness_posteriorcingulate	Desikan Atlas	Heel	IVW	4	1.04	0.04	0.02	2.55E-02	2.70E-01
1078	26880	aparc-Desikan_rh_thickness_precuneus	Desikan Atlas	Total body	IVW	10	0.91	-0.09	0.04	2.00E-02	1.80E-01
1078	26880	aparc-Desikan_rh_thickness_precuneus	Desikan Atlas	Femoral neck	IVW	9	0.85	-0.16	0.06	6.72E-03	4.08E-01
1079	26881	aparc-Desikan_rh_thickness_rostralanteriorcingulate	Desikan Atlas	Heel	IVW	2	0.88	-0.13	0.06	2.70E-02	2.70E-01
1080	26882	aparc-Desikan_rh_thickness_rostralmiddlefrontal	Desikan Atlas	Lumbar spine	IVW	3	0.82	-0.2	0.10	4.75E-02	2.30E-01
1084	26886	aparc-Desikan_rh_thickness_supramarginal	Desikan Atlas	Forearm	IVW	6	0.71	-0.34	0.14	1.21E-02	2.03E-01
1087	26889	aparc-Desikan_rh_thickness_insula	Desikan Atlas	Forearm	IVW	4	0.59	-0.52	0.20	9.38E-03	2.03E-01
378	26891	aparc-Desikan_rh_volume_caudalanteriorcingulate	Desikan Atlas	Total body	IVW	2	0.73	-0.31	0.14	2.96E-02	4.08E-01
378	26891	aparc-Desikan_rh_volume_caudalanteriorcingulate	Desikan Atlas	Heel	IVW	2	0.70	-0.36	0.08	4.91E-06	1.15E-04
378	26891	aparc-Desikan_rh_volume_caudalanteriorcingulate	Desikan Atlas	Lumbar spine	IVW	2	0.68	-0.38	0.15	8.52E-03	3.32E-01
379	26892	aparc-Desikan_rh_volume_caudalmiddlefrontal	Desikan Atlas	Forearm	IVW	3	1.68	0.52	0.20	1.01E-02	3.85E-01
379	26892	aparc-Desikan_rh_volume_caudalmiddlefrontal	Desikan Atlas	Total body	IVW	3	1.31	0.27	0.10	8.52E-03	2.49E-01
380	26893	aparc-Desikan_rh_volume_cuneus	Desikan Atlas	Forearm	IVW	22	1.15	0.14	0.06	2.54E-02	4.95E-01
381	26894	aparc-Desikan_rh_volume_entorhinal	Desikan Atlas	Forearm	WR	1	0.55	-0.6	0.30	4.18E-02	5.44E-01
382	26895	aparc-Desikan_rh_volume_fusiform	Desikan Atlas	Lumbar spine	IVW	4	1.32	0.28	0.13	2.89E-02	5.07E-01
382	26895	aparc-Desikan_rh_volume_fusiform	Desikan Atlas	Total body	IVW	4	1.14	0.13	0.07	4.22E-02	4.08E-01
384	26897	aparc-Desikan_rh_volume_inferiortemporal	Desikan Atlas	Heel	IVW	4	1.11	0.1	0.05	4.71E-02	3.34E-01
385	26898	aparc-Desikan_rh_volume_isthmuscingulate	Desikan Atlas	Heel	IVW	2	0.86	-0.15	0.06	1.24E-02	1.32E-01
389	26902	aparc-Desikan_rh_volume_medialorbitofrontal	Desikan Atlas	Femoral neck	IVW	3	1.26	0.23	0.09	1.29E-02	3.83E-01
390	26903	aparc-Desikan_rh_volume_middletemporal	Desikan Atlas	Heel	WR	1	1.28	0.25	0.04	5.03E-10	1.96E-08
392	26905	aparc-Desikan_rh_volume_paracentral	Desikan Atlas	Total body	WR	1	0.75	-0.29	0.14	3.90E-02	4.08E-01

393	26906	aparc-Desikan_rh_volume_parsopercularis	Desikan Atlas	Femoral neck	IVW	2	0.86	-0.15	0.07	2.93E-02	3.83E-01
398	26911	aparc-Desikan_rh_volume_posteriorcingulate	Desikan Atlas	Heel	WR	1	1.09	0.09	0.04	3.60E-02	3.01E-01
399	26912	aparc-Desikan_rh_volume_precentral	Desikan Atlas	Femoral neck	IVW	4	1.08	0.08	0.04	3.42E-02	3.83E-01
400	26913	aparc-Desikan_rh_volume_precuneus	Desikan Atlas	Lumbar spine	IVW	12	0.90	-0.11	0.06	4.77E-02	5.07E-01
402	26915	aparc-Desikan_rh_volume_rostralmiddlefrontal	Desikan Atlas	Lumbar spine	IVW	6	0.68	-0.38	0.14	5.81E-03	3.32E-01
402	26915	aparc-Desikan_rh_volume_rostralmiddlefrontal	Desikan Atlas	Forearm	IVW	6	0.67	-0.4	0.18	2.33E-02	4.95E-01
402	26915	aparc-Desikan_rh_volume_rostralmiddlefrontal	Desikan Atlas	Total body	IVW	6	0.65	-0.43	0.19	2.28E-02	3.82E-01
407	26920	aparc-Desikan_rh_volume_frontalpole	Desikan Atlas	Heel	WR	1	0.86	-0.15	0.04	2.61E-04	3.81E-03
716	26923	aparc-pial_lh_area_TotalSurface	Desikan Atlas	Total body	IVW	11	0.76	-0.28	0.12	1.63E-02	5.11E-01
719	26926	aparc-pial_lh_area_caudalmiddlefrontal	Desikan Atlas	Forearm	IVW	5	1.42	0.35	0.15	1.60E-02	6.43E-01
719	26926	aparc-pial_lh_area_caudalmiddlefrontal	Desikan Atlas	Total body	IVW	5	1.21	0.19	0.08	1.60E-02	5.11E-01
722	26929	aparc-pial_lh_area_fusiform	Desikan Atlas	Femoral neck	IVW	4	1.20	0.18	0.07	1.16E-02	4.40E-01
723	26930	aparc-pial_lh_area_inferiorparietal	Desikan Atlas	Heel	IVW	5	0.95	-0.05	0.02	5.09E-03	6.38E-02
723	26930	aparc-pial_lh_area_inferiorparietal	Desikan Atlas	Lumbar spine	IVW	4	0.80	-0.22	0.09	1.11E-02	1.74E-01
725	26932	aparc-pial_lh_area_isthmuscingulate	Desikan Atlas	Femoral neck	IVW	4	0.80	-0.22	0.09	1.63E-02	4.41E-01
732	26939	aparc-pial_lh_area_paracentral	Desikan Atlas	Femoral neck	IVW	3	1.25	0.22	0.09	2.25E-02	4.73E-01
733	26940	aparc-pial_lh_area_parsopercularis	Desikan Atlas	Femoral neck	IVW	2	0.79	-0.23	0.09	1.10E-02	4.40E-01
741	26948	aparc-pial_lh_area_rostralanteriorcingulate	Desikan Atlas	Heel	IVW	2	1.17	0.16	0.08	4.19E-02	4.34E-01
742	26949	aparc-pial_lh_area_rostralmiddlefrontal	Desikan Atlas	Lumbar spine	IVW	5	0.76	-0.27	0.08	1.61E-03	6.57E-02
745	26952	aparc-pial_lh_area_superiortemporal	Desikan Atlas	Lumbar spine	WR	1	0.56	-0.58	0.17	9.46E-04	6.57E-02
748	26955	aparc-pial_lh_area_transversetemporal	Desikan Atlas	Lumbar spine	IVW	4	0.81	-0.21	0.09	2.49E-02	3.14E-01
749	26956	aparc-pial_rh_area_TotalSurface	Desikan Atlas	Total body	IVW	14	0.79	-0.23	0.11	4.67E-02	7.27E-01
755	26962	aparc-pial_rh_area_fusiform	Desikan Atlas	Lumbar spine	IVW	4	1.32	0.28	0.10	4.23E-03	1.14E-01
757	26964	aparc-pial_rh_area_inferiortemporal	Desikan Atlas	Heel	IVW	2	1.20	0.18	0.05	7.87E-04	1.48E-02
758	26965	aparc-pial_rh_area_isthmuscingulate	Desikan Atlas	Heel	IVW	3	0.94	-0.06	0.02	8.86E-03	1.04E-01
758	26965	aparc-pial_rh_area_isthmuscingulate	Desikan Atlas	Femoral neck	IVW	3	0.84	-0.18	0.09	4.90E-02	5.65E-01
761	26968	aparc-pial_rh_area_lingual	Desikan Atlas	Forearm	IVW	15	1.16	0.15	0.07	3.74E-02	6.43E-01
766	26973	aparc-pial_rh_area_parsopercularis	Desikan Atlas	Femoral neck	IVW	2	0.84	-0.17	0.08	3.00E-02	4.73E-01
783	27060	BA-exvivo_lh_area_BA2	Broadmann Atlas	Femoral neck	IVW	5	0.86	-0.15	0.06	7.26E-03	2.03E-01
411	27088	BA-exvivo_lh_volume_BA2	Broadmann Atlas	Lumbar spine	WR	1	1.23	0.21	0.11	4.27E-02	4.05E-01
412	27089	BA-exvivo_lh_volume_BA3a	Broadmann Atlas	Femoral neck	WR	1	1.14	0.13	0.06	4.29E-02	2.49E-01
796	27101	BA-exvivo_rh_area_BA1	Broadmann Atlas	Heel	IVW	7	0.94	-0.06	0.02	3.93E-03	7.14E-02
807	27112	BA-exvivo_rh_area_MT	Broadmann Atlas	Forearm	IVW	6	1.38	0.32	0.12	7.19E-03	2.01E-01

807	27112	BA-exvivo_rh_area_MT	Broadmann Atlas	Lumbar spine	IVW	6	1.19	0.17	0.08	3.78E-02	6.97E-01
807	27112	BA-exvivo_rh_area_MT	Broadmann Atlas	Heel	IVW	6	1.14	0.13	0.04	5.10E-03	7.14E-02
807	27112	BA-exvivo_rh_area_MT	Broadmann Atlas	Total body	IVW	7	1.13	0.12	0.04	5.37E-03	1.50E-01
430	27135	BA-exvivo_rh_volume_BA6	Broadmann Atlas	Femoral neck	IVW	8	1.15	0.14	0.07	3.29E-02	2.49E-01
431	27136	BA-exvivo_rh_volume_BA44	Broadmann Atlas	Lumbar spine	WR	1	1.60	0.47	0.21	2.65E-02	4.05E-01
431	27136	BA-exvivo_rh_volume_BA44	Broadmann Atlas	Heel	WR	1	1.12	0.11	0.04	8.08E-03	2.18E-01
435	27140	BA-exvivo_rh_volume_MT	Broadmann Atlas	Lumbar spine	IVW	4	1.36	0.31	0.16	4.93E-02	4.05E-01
435	27140	BA-exvivo_rh_volume_MT	Broadmann Atlas	Femoral neck	IVW	4	1.27	0.24	0.10	1.47E-02	2.49E-01
810	27143	aparc-DKTatlas_lh_area_caudalanteriorcingulate	Desikan Atlas	Forearm	WR	1	1.73	0.55	0.27	4.08E-02	6.43E-01
810	27143	aparc-DKTatlas_lh_area_caudalanteriorcingulate	Desikan Atlas	Femoral neck	WR	1	1.65	0.5	0.13	1.25E-04	2.37E-02
810	27143	aparc-DKTatlas_lh_area_caudalanteriorcingulate	Desikan Atlas	Total body	WR	1	1.54	0.43	0.10	1.30E-05	1.22E-03
810	27143	aparc-DKTatlas_lh_area_caudalanteriorcingulate	Desikan Atlas	Lumbar spine	WR	1	1.51	0.41	0.15	6.80E-03	1.43E-01
810	27143	aparc-DKTatlas_lh_area_caudalanteriorcingulate	Desikan Atlas	Heel	WR	1	1.22	0.2	0.03	4.51E-10	1.71E-08
814	27147	aparc-DKTatlas_lh_area_fusiform	Desikan Atlas	Femoral neck	IVW	4	1.21	0.19	0.07	7.34E-03	4.40E-01
816	27149	aparc-DKTatlas_lh_area_inferiortemporal	Desikan Atlas	Lumbar spine	WR	1	2.14	0.76	0.20	1.69E-04	3.20E-02
816	27149	aparc-DKTatlas_lh_area_inferiortemporal	Desikan Atlas	Heel	WR	1	1.52	0.42	0.04	5.68E-26	4.82E-24
823	27156	aparc-DKTatlas_lh_area_parahippocampal	Desikan Atlas	Heel	IVW	3	1.19	0.17	0.09	4.39E-02	4.34E-01
825	27158	aparc-DKTatlas_lh_area_parsopercularis	Desikan Atlas	Total body	IVW	4	0.87	-0.14	0.06	3.63E-02	7.27E-01
833	27166	aparc-DKTatlas_lh_area_rostralanteriorcingulate	Desikan Atlas	Heel	IVW	5	1.14	0.13	0.04	4.93E-04	1.03E-02
840	27173	aparc-DKTatlas_lh_area_insula	Desikan Atlas	Lumbar spine	IVW	10	0.86	-0.15	0.06	2.44E-02	3.14E-01
439	27206	aparc-DKTatlas_lh_volume_caudalmiddlefrontal	Desikan Atlas	Femoral neck	IVW	6	1.32	0.28	0.12	1.93E-02	3.83E-01
439	27206	aparc-DKTatlas_lh_volume_caudalmiddlefrontal	Desikan Atlas	Total body	IVW	6	1.23	0.21	0.09	1.62E-02	3.16E-01
441	27208	aparc-DKTatlas_lh_volume_entorhinal	Desikan Atlas	Lumbar spine	WR	1	0.66	-0.42	0.21	4.35E-02	5.07E-01
441	27208	aparc-DKTatlas_lh_volume_entorhinal	Desikan Atlas	Forearm	WR	1	0.40	-0.92	0.36	1.01E-02	3.85E-01
442	27209	aparc-DKTatlas_lh_volume_fusiform	Desikan Atlas	Forearm	WR	1	0.51	-0.67	0.33	4.05E-02	5.44E-01
444	27211	aparc-DKTatlas_lh_volume_inferiortemporal	Desikan Atlas	Heel	WR	1	1.15	0.14	0.04	8.67E-04	1.07E-02
453	27220	aparc-DKTatlas_lh_volume_parsopercularis	Desikan Atlas	Femoral neck	IVW	2	0.80	-0.22	0.09	1.11E-02	3.83E-01
454	27221	aparc-DKTatlas_lh_volume_parsorbitalis	Desikan Atlas	Lumbar spine	IVW	2	0.73	-0.32	0.13	1.24E-02	3.63E-01
841	27236	aparc-DKTatlas_rh_area_caudalanteriorcingulate	Desikan Atlas	Lumbar spine	IVW	2	0.72	-0.33	0.13	1.08E-02	1.74E-01
845	27240	aparc-DKTatlas_rh_area_fusiform	Desikan Atlas	Heel	IVW	4	1.16	0.15	0.05	1.74E-03	2.52E-02
848	27243	aparc-DKTatlas_rh_area_isthmuscingulate	Desikan Atlas	Heel	IVW	6	0.91	-0.09	0.03	1.59E-03	2.52E-02
855	27250	aparc-DKTatlas_rh_area_paracentral	Desikan Atlas	Lumbar spine	IVW	5	0.77	-0.26	0.10	9.35E-03	1.74E-01
855	27250	aparc-DKTatlas_rh_area_paracentral	Desikan Atlas	Forearm	IVW	5	0.72	-0.33	0.15	3.13E-02	6.43E-01

862	27257	aparc-DKTatlas_rh_area_precentral	Desikan Atlas	Femoral neck	IVW	6	1.11	0.1	0.05	3.31E-02	4.82E-01
864	27259	aparc-DKTatlas_rh_area_rostralanteriorcingulate	Desikan Atlas	Total body	WR	1	1.25	0.22	0.11	4.55E-02	7.27E-01
864	27259	aparc-DKTatlas_rh_area_rostralanteriorcingulate	Desikan Atlas	Heel	WR	1	1.16	0.15	0.03	8.17E-07	2.56E-05
868	27263	aparc-DKTatlas_rh_area_superiortemporal	Desikan Atlas	Lumbar spine	IVW	6	0.82	-0.2	0.10	4.63E-02	4.86E-01
870	27265	aparc-DKTatlas_rh_area_transversetemporal	Desikan Atlas	Forearm	IVW	4	1.51	0.41	0.18	2.56E-02	6.43E-01
870	27265	aparc-DKTatlas_rh_area_transversetemporal	Desikan Atlas	Femoral neck	IVW	4	1.26	0.23	0.08	7.28E-03	4.40E-01
871	27266	aparc-DKTatlas_rh_area_insula	Desikan Atlas	Total body	IVW	15	0.90	-0.1	0.03	9.06E-04	5.68E-02
871	27266	aparc-DKTatlas_rh_area_insula	Desikan Atlas	Lumbar spine	IVW	15	0.85	-0.16	0.05	1.74E-03	6.57E-02
469	27298	aparc-DKTatlas_rh_volume_caudalanteriorcingulate	Desikan Atlas	Heel	WR	1	0.66	-0.41	0.04	1.11E-24	6.49E-23
469	27298	aparc-DKTatlas_rh_volume_caudalanteriorcingulate	Desikan Atlas	Total body	WR	1	0.66	-0.41	0.12	7.56E-04	8.85E-02
469	27298	aparc-DKTatlas_rh_volume_caudalanteriorcingulate	Desikan Atlas	Lumbar spine	WR	1	0.65	-0.43	0.19	2.03E-02	4.75E-01
470	27299	aparc-DKTatlas_rh_volume_caudalmiddlefrontal	Desikan Atlas	Forearm	IVW	3	1.67	0.51	0.20	1.02E-02	3.85E-01
470	27299	aparc-DKTatlas_rh_volume_caudalmiddlefrontal	Desikan Atlas	Total body	IVW	3	1.31	0.27	0.10	8.36E-03	2.49E-01
472	27301	aparc-DKTatlas_rh_volume_entorhinal	Desikan Atlas	Forearm	WR	1	0.49	-0.72	0.29	1.32E-02	3.85E-01
473	27302	aparc-DKTatlas_rh_volume_fusiform	Desikan Atlas	Forearm	IVW	2	1.67	0.51	0.25	3.86E-02	5.44E-01
475	27304	aparc-DKTatlas_rh_volume_inferiortemporal	Desikan Atlas	Total body	WR	1	1.31	0.27	0.13	3.87E-02	4.08E-01
475	27304	aparc-DKTatlas_rh_volume_inferiortemporal	Desikan Atlas	Heel	WR	1	1.27	0.24	0.04	3.51E-08	1.03E-06
478	27307	aparc-DKTatlas_rh_volume_lateralorbitofrontal	Desikan Atlas	Heel	IVW	7	1.15	0.14	0.04	1.60E-04	2.67E-03
480	27309	aparc-DKTatlas_rh_volume_medialorbitofrontal	Desikan Atlas	Femoral neck	IVW	3	1.21	0.19	0.09	3.08E-02	3.83E-01
483	27312	aparc-DKTatlas_rh_volume_paracentral	Desikan Atlas	Total body	IVW	2	0.82	-0.2	0.10	3.65E-02	4.08E-01
483	27312	aparc-DKTatlas_rh_volume_paracentral	Desikan Atlas	Lumbar spine	IVW	2	0.73	-0.32	0.15	3.94E-02	5.07E-01
484	27313	aparc-DKTatlas_rh_volume_parsopercularis	Desikan Atlas	Femoral neck	IVW	2	0.86	-0.15	0.07	2.92E-02	3.83E-01
492	27321	aparc-DKTatlas_rh_volume_rostralanteriorcingulate	Desikan Atlas	Heel	WR	1	1.17	0.16	0.04	1.37E-04	2.67E-03
495	27324	aparc-DKTatlas_rh_volume_superiorparietal	Desikan Atlas	Total body	IVW	10	0.93	-0.07	0.04	4.54E-02	4.08E-01
497	27326	aparc-DKTatlas_rh_volume_supramarginal	Desikan Atlas	Lumbar spine	IVW	6	1.12	0.11	0.05	3.56E-02	5.07E-01
499	27328	aparc-DKTatlas_rh_volume_insula	Desikan Atlas	Heel	IVW	9	0.90	-0.1	0.05	3.08E-02	3.00E-01
873	27330	aparc-a2009s_lh_area_G+S-occipital-inf	Destrieux Atlas	Lumbar spine	IVW	2	0.73	-0.32	0.13	1.81E-02	3.30E-01
879	27336	aparc-a2009s_lh_area_G+S-cingul-Mid-Post	Destrieux Atlas	Forearm	IVW	3	0.66	-0.42	0.20	3.52E-02	6.71E-01
880	27337	aparc-a2009s_lh_area_G-cingul-Post-dorsal	Destrieux Atlas	Lumbar spine	IVW	2	1.35	0.3	0.14	3.07E-02	4.08E-01
880	27337	aparc-a2009s_lh_area_G-cingul-Post-dorsal	Destrieux Atlas	Femoral neck	IVW	2	1.26	0.23	0.11	4.28E-02	4.27E-01
884	27341	aparc-a2009s_lh_area_G-front-inf-Orbital	Destrieux Atlas	Total body	WR	1	1.39	0.33	0.12	6.73E-03	2.92E-01
884	27341	aparc-a2009s_lh_area_G-front-inf-Orbital	Destrieux Atlas	Heel	WR	1	1.16	0.15	0.04	1.69E-04	5.37E-03
888	27345	aparc-a2009s_lh_area_G-Ins-lg+S-cent-ins	Destrieux Atlas	Lumbar spine	WR	1	0.66	-0.42	0.20	3.93E-02	4.08E-01

896	27353	aparc-a2009s_lh_area_G-pariet-inf-Angular	Destrieux Atlas	Lumbar spine	IVW	3	1.48	0.39	0.18	3.25E-02	4.08E-01
900	27357	aparc-a2009s_lh_area_G-precentral	Destrieux Atlas	Femoral neck	IVW	3	1.11	0.1	0.04	1.55E-02	4.27E-01
902	27359	aparc-a2009s_lh_area_G-rectus	Destrieux Atlas	Heel	WR	1	1.14	0.13	0.04	1.27E-03	2.68E-02
917	27374	aparc-a2009s_lh_area_S-cingul-Marginalis	Destrieux Atlas	Femoral neck	IVW	2	1.34	0.29	0.12	1.51E-02	4.27E-01
918	27375	aparc-a2009s_lh_area_S-circular-insula-ant	Destrieux Atlas	Femoral neck	IVW	3	1.49	0.4	0.09	2.57E-05	3.29E-03
918	27375	aparc-a2009s_lh_area_S-circular-insula-ant	Destrieux Atlas	Heel	IVW	3	1.23	0.21	0.08	9.43E-03	1.50E-01
920	27377	aparc-a2009s_lh_area_S-circular-insula-sup	Destrieux Atlas	Lumbar spine	IVW	7	0.84	-0.18	0.09	4.87E-02	4.08E-01
926	27383	aparc-a2009s_lh_area_S-interm-prim-Jensen	Destrieux Atlas	Femoral neck	WR	1	0.62	-0.48	0.21	2.17E-02	4.27E-01
933	27390	aparc-a2009s_lh_area_S-orbital-lateral	Destrieux Atlas	Forearm	WR	1	2.01	0.7	0.27	1.04E-02	6.65E-01
933	27390	aparc-a2009s_lh_area_S-orbital-lateral	Destrieux Atlas	Heel	WR	1	0.84	-0.18	0.03	9.08E-09	5.77E-07
935	27392	aparc-a2009s_lh_area_S-orbital-H-Shaped	Destrieux Atlas	Heel	IVW	7	1.16	0.15	0.04	3.02E-04	7.66E-03
935	27392	aparc-a2009s_lh_area_S-orbital-H-Shaped	Destrieux Atlas	Femoral neck	IVW	9	1.16	0.15	0.07	4.20E-02	4.27E-01
938	27395	aparc-a2009s_lh_area_S-postcentral	Destrieux Atlas	Lumbar spine	IVW	5	1.15	0.14	0.07	3.67E-02	4.08E-01
939	27396	aparc-a2009s_lh_area_S-precentral-inf-part	Destrieux Atlas	Femoral neck	WR	1	1.34	0.29	0.14	4.29E-02	4.27E-01
940	27397	aparc-a2009s_lh_area_S-precentral-sup-part	Destrieux Atlas	Femoral neck	WR	1	1.22	0.2	0.09	2.69E-02	4.27E-01
943	27400	aparc-a2009s_lh_area_S-temporal-inf	Destrieux Atlas	Heel	WR	1	0.90	-0.1	0.04	1.37E-02	1.90E-01
943	27400	aparc-a2009s_lh_area_S-temporal-inf	Destrieux Atlas	Total body	WR	1	0.64	-0.45	0.12	2.39E-04	3.11E-02
943	27400	aparc-a2009s_lh_area_S-temporal-inf	Destrieux Atlas	Lumbar spine	WR	1	0.54	-0.61	0.19	1.54E-03	9.83E-02
1179	27404	aparc-a2009s_lh_thickness_G+S-occipital-inf	Destrieux Atlas	Lumbar spine	IVW	5	0.75	-0.29	0.09	2.29E-03	4.00E-02
1180	27405	aparc-a2009s_lh_thickness_G+S-paracentral	Destrieux Atlas	Heel	IVW	3	1.08	0.08	0.04	4.88E-02	1.97E-01
1181	27406	aparc-a2009s_lh_thickness_G+S-subcentral	Destrieux Atlas	Femoral neck	IVW	6	0.83	-0.19	0.07	4.72E-03	2.48E-01
1181	27406	aparc-a2009s_lh_thickness_G+S-subcentral	Destrieux Atlas	Forearm	IVW	6	0.72	-0.33	0.14	1.58E-02	4.47E-01
1183	27408	aparc-a2009s_lh_thickness_G+S-cingul-Ant	Destrieux Atlas	Femoral neck	WR	1	0.73	-0.32	0.15	3.65E-02	2.78E-01
1183	27408	aparc-a2009s_lh_thickness_G+S-cingul-Ant	Destrieux Atlas	Heel	WR	1	0.73	-0.32	0.03	9.89E-26	5.98E-24
1183	27408	aparc-a2009s_lh_thickness_G+S-cingul-Ant	Destrieux Atlas	Lumbar spine	WR	1	0.52	-0.66	0.19	5.36E-04	1.31E-02
1185	27410	aparc-a2009s_lh_thickness_G+S-cingul-Mid-Post	Destrieux Atlas	Heel	WR	1	0.79	-0.23	0.04	2.54E-08	3.41E-07
1186	27411	aparc-a2009s_lh_thickness_G-cingul-Post-dorsal	Destrieux Atlas	Heel	WR	1	1.16	0.15	0.04	8.95E-05	6.77E-04
1187	27412	aparc-a2009s_lh_thickness_G-cingul-Post-ventral	Destrieux Atlas	Lumbar spine	IVW	3	0.80	-0.22	0.10	2.92E-02	2.29E-01
1188	27413	aparc-a2009s_lh_thickness_G-cuneus	Destrieux Atlas	Heel	IVW	5	1.06	0.06	0.03	2.50E-02	1.26E-01
1190	27415	aparc-a2009s_lh_thickness_G-front-inf-Orbital	Destrieux Atlas	Heel	WR	1	1.39	0.33	0.04	2.33E-17	4.70E-16
1195	27420	aparc-a2009s_lh_thickness_G-insular-short	Destrieux Atlas	Forearm	WR	1	1.90	0.64	0.31	3.73E-02	4.50E-01
1196	27421	aparc-a2009s_lh_thickness_G-occipital-middle	Destrieux Atlas	Total body	IVW	7	0.85	-0.16	0.06	8.30E-03	2.28E-01
1196	27421	aparc-a2009s_lh_thickness_G-occipital-middle	Destrieux Atlas	Lumbar spine	IVW	6	0.79	-0.24	0.12	4.41E-02	2.99E-01

1197	27422	aparc-a2009s_lh_thickness_G-occipital-sup	Destrieux Atlas	Heel	IVW	3	1.09	0.09	0.03	5.94E-03	3.59E-02
1197	27422	aparc-a2009s_lh_thickness_G-occipital-sup	Destrieux Atlas	Femoral neck	IVW	4	0.83	-0.19	0.08	1.36E-02	2.48E-01
1198	27423	aparc-a2009s_lh_thickness_G-oc-temp-lat-fusifor	Destrieux Atlas	Femoral neck	IVW	4	0.82	-0.2	0.09	2.67E-02	2.68E-01
1198	27423	aparc-a2009s_lh_thickness_G-oc-temp-lat-fusifor	Destrieux Atlas	Forearm	IVW	4	0.60	-0.51	0.16	1.58E-03	1.97E-01
1199	27424	aparc-a2009s_lh_thickness_G-oc-temp-med-Lingual	Destrieux Atlas	Heel	WR	1	1.16	0.15	0.03	4.36E-06	4.06E-05
1200	27425	aparc-a2009s_lh_thickness_G-oc-temp-med-Parahip	Destrieux Atlas	Lumbar spine	IVW	2	1.35	0.3	0.14	3.00E-02	2.29E-01
1203	27428	aparc-a2009s_lh_thickness_G-pariet-inf-Supramar	Destrieux Atlas	Forearm	IVW	5	0.69	-0.37	0.18	4.35E-02	4.50E-01
1205	27430	aparc-a2009s_lh_thickness_G-postcentral	Destrieux Atlas	Femoral neck	IVW	5	1.08	0.08	0.04	2.11E-02	2.57E-01
1206	27431	aparc-a2009s_lh_thickness_G-precentral	Destrieux Atlas	Heel	IVW	5	1.06	0.06	0.03	3.25E-02	1.51E-01
1206	27431	aparc-a2009s_lh_thickness_G-precentral	Destrieux Atlas	Femoral neck	IVW	5	0.85	-0.16	0.07	2.33E-02	2.58E-01
1207	27432	aparc-a2009s_lh_thickness_G-precuneus	Destrieux Atlas	Femoral neck	IVW	6	0.81	-0.21	0.07	3.45E-03	2.48E-01
1210	27435	aparc-a2009s_lh_thickness_G-temp-sup-G-T-transv	Destrieux Atlas	Heel	WR	1	1.16	0.15	0.03	4.36E-06	4.06E-05
1212	27437	aparc-a2009s_lh_thickness_G-temp-sup-Plan-polar	Destrieux Atlas	Femoral neck	WR	1	1.52	0.42	0.17	1.63E-02	2.48E-01
1214	27439	aparc-a2009s_lh_thickness_G-temporal-inf	Destrieux Atlas	Heel	IVW	5	0.86	-0.15	0.07	2.91E-02	1.41E-01
1214	27439	aparc-a2009s_lh_thickness_G-temporal-inf	Destrieux Atlas	Lumbar spine	IVW	5	0.66	-0.41	0.10	6.02E-05	7.34E-03
1219	27444	aparc-a2009s_lh_thickness_Pole-occipital	Destrieux Atlas	Heel	IVW	2	1.11	0.1	0.02	9.58E-07	1.16E-05
1219	27444	aparc-a2009s_lh_thickness_Pole-occipital	Destrieux Atlas	Lumbar spine	IVW	2	0.80	-0.22	0.09	2.14E-02	2.05E-01
1220	27445	aparc-a2009s_lh_thickness_Pole-temporal	Destrieux Atlas	Forearm	IVW	2	0.55	-0.6	0.30	4.61E-02	4.50E-01
1221	27446	aparc-a2009s_lh_thickness_S-calcarine	Destrieux Atlas	Heel	WR	1	1.21	0.19	0.04	4.36E-06	4.06E-05
1222	27447	aparc-a2009s_lh_thickness_S-central	Destrieux Atlas	Heel	IVW	2	1.12	0.11	0.04	1.17E-02	6.15E-02
1224	27449	aparc-a2009s_lh_thickness_S-circular-insula-ant	Destrieux Atlas	Lumbar spine	WR	1	1.79	0.58	0.23	1.19E-02	1.45E-01
1224	27449	aparc-a2009s_lh_thickness_S-circular-insula-ant	Destrieux Atlas	Total body	WR	1	1.68	0.52	0.19	4.90E-03	2.28E-01
1226	27451	aparc-a2009s_lh_thickness_S-circular-insula-sup	Destrieux Atlas	Heel	IVW	4	1.09	0.09	0.04	4.39E-02	1.83E-01
1230	27455	aparc-a2009s_lh_thickness_S-front-middle	Destrieux Atlas	Heel	IVW	2	0.91	-0.09	0.04	9.70E-03	5.59E-02
1234	27459	aparc-a2009s_lh_thickness_S-oc-middle+Lunatus	Destrieux Atlas	Forearm	IVW	2	0.66	-0.42	0.21	4.91E-02	4.50E-01
1237	27462	aparc-a2009s_lh_thickness_S-oc-temp-lat	Destrieux Atlas	Total body	WR	1	0.75	-0.29	0.11	1.07E-02	2.28E-01
1237	27462	aparc-a2009s_lh_thickness_S-oc-temp-lat	Destrieux Atlas	Heel	WR	1	0.72	-0.33	0.03	3.74E-25	1.29E-23
1237	27462	aparc-a2009s_lh_thickness_S-oc-temp-lat	Destrieux Atlas	Lumbar spine	WR	1	0.54	-0.61	0.16	1.74E-04	8.51E-03
1241	27466	aparc-a2009s_lh_thickness_S-orbital-H-Shaped	Destrieux Atlas	Heel	IVW	2	0.76	-0.28	0.03	3.78E-17	6.53E-16
1242	27467	aparc-a2009s_lh_thickness_S-parieto-occipital	Destrieux Atlas	Femoral neck	IVW	5	0.84	-0.17	0.08	3.16E-02	2.68E-01
1242	27467	aparc-a2009s_lh_thickness_S-parieto-occipital	Destrieux Atlas	Forearm	IVW	6	0.71	-0.34	0.14	1.80E-02	4.47E-01
1243	27468	aparc-a2009s_lh_thickness_S-pericallosal	Destrieux Atlas	Lumbar spine	IVW	3	0.79	-0.24	0.11	2.33E-02	2.05E-01
1245	27470	aparc-a2009s_lh_thickness_S-precentral-inf-part	Destrieux Atlas	Heel	WR	1	1.11	0.1	0.04	1.06E-02	5.81E-02

1245	27470	aparc-a2009s_lh_thickness_S-precentral-inf-part	Destrieux Atlas	Femoral neck	WR	1	0.64	-0.44	0.16	6.88E-03	2.48E-01
1247	27472	aparc-a2009s_lh_thickness_S-suborbital	Destrieux Atlas	Heel	WR	1	0.68	-0.38	0.04	5.86E-24	1.42E-22
1247	27472	aparc-a2009s_lh_thickness_S-suborbital	Destrieux Atlas	Lumbar spine	WR	1	0.58	-0.54	0.24	2.35E-02	2.05E-01
1249	27474	aparc-a2009s_lh_thickness_S-temporal-inf	Destrieux Atlas	Total body	WR	1	0.70	-0.35	0.14	1.07E-02	2.28E-01
1249	27474	aparc-a2009s_lh_thickness_S-temporal-inf	Destrieux Atlas	Heel	WR	1	0.67	-0.4	0.04	3.74E-25	1.29E-23
1249	27474	aparc-a2009s_lh_thickness_S-temporal-inf	Destrieux Atlas	Lumbar spine	WR	1	0.47	-0.75	0.20	1.74E-04	8.51E-03
501	27478	aparc-a2009s_lh_volume_G+S-occipital-inf	Destrieux Atlas	Lumbar spine	IVW	2	0.73	-0.32	0.13	1.63E-02	4.94E-01
503	27480	aparc-a2009s_lh_volume_G+S-subcentral	Destrieux Atlas	Femoral neck	IVW	2	1.14	0.13	0.06	3.24E-02	2.74E-01
505	27482	aparc-a2009s_lh_volume_G+S-cingul-Ant	Destrieux Atlas	Femoral neck	WR	1	1.84	0.61	0.28	2.83E-02	2.74E-01
505	27482	aparc-a2009s_lh_volume_G+S-cingul-Ant	Destrieux Atlas	Total body	WR	1	1.73	0.55	0.14	5.25E-05	3.26E-03
505	27482	aparc-a2009s_lh_volume_G+S-cingul-Ant	Destrieux Atlas	Heel	WR	1	1.27	0.24	0.04	2.79E-09	1.11E-07
506	27483	aparc-a2009s_lh_volume_G+S-cingul-Mid-Ant	Destrieux Atlas	Forearm	WR	1	1.92	0.65	0.32	4.08E-02	4.49E-01
506	27483	aparc-a2009s_lh_volume_G+S-cingul-Mid-Ant	Destrieux Atlas	Femoral neck	WR	1	1.80	0.59	0.15	1.25E-04	1.52E-02
506	27483	aparc-a2009s_lh_volume_G+S-cingul-Mid-Ant	Destrieux Atlas	Total body	WR	1	1.65	0.5	0.12	1.30E-05	1.61E-03
506	27483	aparc-a2009s_lh_volume_G+S-cingul-Mid-Ant	Destrieux Atlas	Lumbar spine	WR	1	1.62	0.48	0.18	6.80E-03	4.12E-01
506	27483	aparc-a2009s_lh_volume_G+S-cingul-Mid-Ant	Destrieux Atlas	Heel	WR	1	1.27	0.24	0.04	4.51E-10	2.68E-08
508	27485	aparc-a2009s_lh_volume_G-cingul-Post-dorsal	Destrieux Atlas	Femoral neck	WR	1	0.75	-0.29	0.14	3.49E-02	2.74E-01
510	27487	aparc-a2009s_lh_volume_G-cuneus	Destrieux Atlas	Femoral neck	IVW	15	1.09	0.09	0.04	3.58E-02	2.74E-01
514	27491	aparc-a2009s_lh_volume_G-front-middle	Destrieux Atlas	Forearm	IVW	6	1.72	0.54	0.19	4.98E-03	1.29E-01
523	27500	aparc-a2009s_lh_volume_G-orbital	Destrieux Atlas	Femoral neck	WR	1	1.57	0.45	0.16	3.97E-03	1.60E-01
523	27500	aparc-a2009s_lh_volume_G-orbital	Destrieux Atlas	Heel	WR	1	0.91	-0.09	0.04	1.57E-02	1.04E-01
524	27501	aparc-a2009s_lh_volume_G-pariet-inf-Angular	Destrieux Atlas	Heel	IVW	4	1.08	0.08	0.03	2.47E-03	2.10E-02
531	27508	aparc-a2009s_lh_volume_G-subcallosal	Destrieux Atlas	Heel	WR	1	1.19	0.17	0.04	6.79E-05	1.15E-03
533	27510	aparc-a2009s_lh_volume_G-temp-sup-Lateral	Destrieux Atlas	Heel	WR	1	1.13	0.12	0.04	8.62E-04	1.14E-02
534	27511	aparc-a2009s_lh_volume_G-temp-sup-Plan-polar	Destrieux Atlas	Femoral neck	WR	1	1.77	0.57	0.19	2.48E-03	1.50E-01
534	27511	aparc-a2009s_lh_volume_G-temp-sup-Plan-polar	Destrieux Atlas	Lumbar spine	WR	1	1.77	0.57	0.22	1.10E-02	4.46E-01
535	27512	aparc-a2009s_lh_volume_G-temp-sup-Plan-tempo	Destrieux Atlas	Heel	WR	1	1.15	0.14	0.04	1.03E-03	1.23E-02
537	27514	aparc-a2009s_lh_volume_G-temporal-middle	Destrieux Atlas	Femoral neck	IVW	2	1.34	0.29	0.13	3.10E-02	2.74E-01
538	27515	aparc-a2009s_lh_volume_Lat-Fis-ant-Horizont	Destrieux Atlas	Lumbar spine	WR	1	0.68	-0.39	0.19	4.27E-02	6.08E-01
546	27523	aparc-a2009s_lh_volume_S-circular-insula-ant	Destrieux Atlas	Femoral neck	IVW	3	1.32	0.28	0.12	1.44E-02	2.74E-01
553	27530	aparc-a2009s_lh_volume_S-front-sup	Destrieux Atlas	Lumbar spine	WR	1	0.66	-0.42	0.21	4.27E-02	6.08E-01
562	27539	aparc-a2009s_lh_volume_S-orbital-med-olfact	Destrieux Atlas	Forearm	IVW	2	1.62	0.48	0.24	4.96E-02	4.93E-01
562	27539	aparc-a2009s_lh_volume_S-orbital-med-olfact	Destrieux Atlas	Total body	IVW	2	1.27	0.24	0.09	5.16E-03	2.08E-01

563	27540	aparc-a2009s_lh_volume_S-orbital-H-Shaped	Destrieux Atlas	Heel	IVW	6	1.12	0.11	0.04	1.93E-03	1.77E-02
564	27541	aparc-a2009s_lh_volume_S-parieto-occipital	Destrieux Atlas	Lumbar spine	IVW	16	0.91	-0.09	0.05	4.11E-02	6.08E-01
567	27544	aparc-a2009s_lh_volume_S-precentral-inf-part	Destrieux Atlas	Femoral neck	WR	1	1.36	0.31	0.15	4.29E-02	2.89E-01
568	27545	aparc-a2009s_lh_volume_S-precentral-sup-part	Destrieux Atlas	Femoral neck	IVW	2	1.20	0.18	0.08	2.45E-02	2.74E-01
947	27552	aparc-a2009s_rh_area_G+S-occipital-inf	Destrieux Atlas	Forearm	IVW	4	1.45	0.37	0.17	3.02E-02	6.71E-01
952	27557	aparc-a2009s_rh_area_G+S-cingul-Mid-Ant	Destrieux Atlas	Femoral neck	IVW	3	1.27	0.24	0.12	4.67E-02	4.27E-01
954	27559	aparc-a2009s_rh_area_G-cingul-Post-dorsal	Destrieux Atlas	Femoral neck	WR	1	0.69	-0.37	0.17	2.66E-02	4.27E-01
956	27561	aparc-a2009s_rh_area_G-cuneus	Destrieux Atlas	Femoral neck	IVW	19	1.08	0.08	0.04	3.74E-02	4.27E-01
957	27562	aparc-a2009s_rh_area_G-front-inf-Opercular	Destrieux Atlas	Femoral neck	WR	1	0.72	-0.33	0.16	3.82E-02	4.27E-01
958	27563	aparc-a2009s_rh_area_G-front-inf-Orbital	Destrieux Atlas	Lumbar spine	IVW	3	0.74	-0.3	0.11	5.55E-03	1.92E-01
962	27567	aparc-a2009s_rh_area_G-Ins-lg+S-cent-ins	Destrieux Atlas	Heel	WR	1	1.08	0.08	0.03	2.72E-02	2.88E-01
963	27568	aparc-a2009s_rh_area_G-insular-short	Destrieux Atlas	Total body	WR	1	0.74	-0.3	0.15	4.35E-02	6.61E-01
964	27569	aparc-a2009s_rh_area_G-occipital-middle	Destrieux Atlas	Total body	IVW	7	1.12	0.11	0.05	4.07E-02	6.61E-01
968	27573	aparc-a2009s_rh_area_G-oc-temp-med-Parahip	Destrieux Atlas	Heel	IVW	2	1.06	0.06	0.03	3.16E-02	3.09E-01
972	27577	aparc-a2009s_rh_area_G-parietal-sup	Destrieux Atlas	Forearm	IVW	7	0.79	-0.23	0.11	4.19E-02	6.71E-01
974	27579	aparc-a2009s_rh_area_G-precentral	Destrieux Atlas	Femoral neck	IVW	4	1.08	0.08	0.04	4.36E-02	4.27E-01
979	27584	aparc-a2009s_rh_area_G-temp-sup-Lateral	Destrieux Atlas	Forearm	IVW	5	0.65	-0.43	0.15	4.23E-03	5.41E-01
984	27589	aparc-a2009s_rh_area_Lat-Fis-ant-Horizont	Destrieux Atlas	Lumbar spine	WR	1	0.66	-0.41	0.20	4.27E-02	4.08E-01
993	27598	aparc-a2009s_rh_area_S-circular-insula-inf	Destrieux Atlas	Lumbar spine	IVW	2	0.71	-0.34	0.14	1.17E-02	2.49E-01
994	27599	aparc-a2009s_rh_area_S-circular-insula-sup	Destrieux Atlas	Heel	IVW	6	0.88	-0.13	0.05	1.52E-02	1.90E-01
994	27599	aparc-a2009s_rh_area_S-circular-insula-sup	Destrieux Atlas	Total body	IVW	6	0.84	-0.17	0.05	5.66E-04	3.68E-02
995	27600	aparc-a2009s_rh_area_S-collat-transv-ant	Destrieux Atlas	Forearm	IVW	7	1.22	0.2	0.10	3.91E-02	6.71E-01
995	27600	aparc-a2009s_rh_area_S-collat-transv-ant	Destrieux Atlas	Lumbar spine	IVW	7	1.19	0.17	0.06	5.99E-03	1.92E-01
995	27600	aparc-a2009s_rh_area_S-collat-transv-ant	Destrieux Atlas	Total body	IVW	7	1.12	0.11	0.05	3.00E-02	6.61E-01
996	27601	aparc-a2009s_rh_area_S-collat-transv-post	Destrieux Atlas	Lumbar spine	WR	1	1.90	0.64	0.24	7.98E-03	2.04E-01
1002	27607	aparc-a2009s_rh_area_S-oc-middle+Lunatus	Destrieux Atlas	Total body	IVW	9	1.15	0.14	0.06	1.90E-02	6.17E-01
1005	27610	aparc-a2009s_rh_area_S-oc-temp-lat	Destrieux Atlas	Lumbar spine	IVW	2	0.75	-0.29	0.15	4.78E-02	4.08E-01
1005	27610	aparc-a2009s_rh_area_S-oc-temp-lat	Destrieux Atlas	Forearm	IVW	2	0.52	-0.66	0.30	2.69E-02	6.71E-01
1008	27613	aparc-a2009s_rh_area_S-orbital-med-olfact	Destrieux Atlas	Forearm	IVW	5	1.30	0.26	0.12	2.56E-02	6.71E-01
1009	27614	aparc-a2009s_rh_area_S-orbital-H-Shaped	Destrieux Atlas	Heel	IVW	3	1.20	0.18	0.07	1.65E-02	1.90E-01
1011	27616	aparc-a2009s_rh_area_S-pericallosal	Destrieux Atlas	Heel	IVW	3	0.94	-0.06	0.02	4.50E-03	8.17E-02
1012	27617	aparc-a2009s_rh_area_S-postcentral	Destrieux Atlas	Lumbar spine	IVW	5	1.16	0.15	0.07	3.42E-02	4.08E-01
1013	27618	aparc-a2009s_rh_area_S-precentral-inf-part	Destrieux Atlas	Lumbar spine	WR	1	2.64	0.97	0.26	2.29E-04	2.93E-02

1013	27618	aparc-a2009s_rh_area_S-precentral-inf-part	Destrieux Atlas	Heel	WR	1	1.45	0.37	0.04	8.42E-19	1.07E-16
1013	27618	aparc-a2009s_rh_area_S-precentral-inf-part	Destrieux Atlas	Total body	WR	1	1.38	0.32	0.16	4.27E-02	6.61E-01
1014	27619	aparc-a2009s_rh_area_S-precentral-sup-part	Destrieux Atlas	Femoral neck	WR	1	1.21	0.19	0.09	2.69E-02	4.27E-01
1017	27622	aparc-a2009s_rh_area_S-temporal-inf	Destrieux Atlas	Heel	WR	1	0.83	-0.19	0.04	1.19E-05	5.04E-04
1252	27625	aparc-a2009s_rh_thickness_G+S-frontomargin	Destrieux Atlas	Total body	WR	1	0.71	-0.34	0.17	4.37E-02	4.01E-01
1252	27625	aparc-a2009s_rh_thickness_G+S-frontomargin	Destrieux Atlas	Femoral neck	WR	1	0.59	-0.52	0.24	3.30E-02	2.68E-01
1252	27625	aparc-a2009s_rh_thickness_G+S-frontomargin	Destrieux Atlas	Lumbar spine	WR	1	0.37	-0.99	0.28	3.66E-04	1.12E-02
1253	27626	aparc-a2009s_rh_thickness_G+S-occipital-inf	Destrieux Atlas	Total body	IVW	4	0.85	-0.16	0.06	1.10E-02	2.28E-01
1253	27626	aparc-a2009s_rh_thickness_G+S-occipital-inf	Destrieux Atlas	Lumbar spine	IVW	4	0.82	-0.2	0.09	3.49E-02	2.51E-01
1254	27627	aparc-a2009s_rh_thickness_G+S-paracentral	Destrieux Atlas	Heel	WR	1	1.13	0.12	0.03	4.36E-06	4.06E-05
1257	27630	aparc-a2009s_rh_thickness_G+S-cingul-Ant	Destrieux Atlas	Heel	WR	1	0.90	-0.11	0.03	6.60E-04	4.21E-03
1271	27644	aparc-a2009s_rh_thickness_G-occipital-sup	Destrieux Atlas	Femoral neck	IVW	3	0.83	-0.19	0.08	1.56E-02	2.48E-01
1274	27647	aparc-a2009s_rh_thickness_G-oc-temp-med-Parahip	Destrieux Atlas	Forearm	WR	1	0.40	-0.92	0.37	1.32E-02	4.47E-01
1276	27649	aparc-a2009s_rh_thickness_G-pariet-inf-Angular	Destrieux Atlas	Total body	IVW	5	0.84	-0.17	0.07	1.97E-02	3.08E-01
1276	27649	aparc-a2009s_rh_thickness_G-pariet-inf-Angular	Destrieux Atlas	Forearm	IVW	4	0.66	-0.42	0.18	2.18E-02	4.50E-01
1277	27650	aparc-a2009s_rh_thickness_G-pariet-inf-Supramar	Destrieux Atlas	Forearm	IVW	2	0.60	-0.51	0.24	3.26E-02	4.50E-01
1281	27654	aparc-a2009s_rh_thickness_G-precuneus	Destrieux Atlas	Heel	IVW	10	1.06	0.06	0.03	3.54E-02	1.56E-01
1284	27657	aparc-a2009s_rh_thickness_G-temp-sup-G-T-transv	Destrieux Atlas	Femoral neck	IVW	2	0.82	-0.2	0.10	4.80E-02	3.44E-01
1284	27657	aparc-a2009s_rh_thickness_G-temp-sup-G-T-transv	Destrieux Atlas	Lumbar spine	IVW	2	0.71	-0.34	0.17	4.77E-02	3.05E-01
1288	27661	aparc-a2009s_rh_thickness_G-temporal-inf	Destrieux Atlas	Total body	IVW	5	0.85	-0.16	0.06	1.50E-02	2.67E-01
1289	27662	aparc-a2009s_rh_thickness_G-temporal-middle	Destrieux Atlas	Femoral neck	IVW	4	0.81	-0.21	0.09	1.63E-02	2.48E-01
1290	27663	aparc-a2009s_rh_thickness_Lat-Fis-ant-Horizont	Destrieux Atlas	Heel	WR	1	1.08	0.08	0.04	3.62E-02	1.56E-01
1293	27666	aparc-a2009s_rh_thickness_Pole-occipital	Destrieux Atlas	Heel	WR	1	1.09	0.09	0.02	4.36E-06	4.06E-05
1299	27672	aparc-a2009s_rh_thickness_S-circular-insula-inf	Destrieux Atlas	Forearm	WR	1	0.41	-0.9	0.43	3.77E-02	4.50E-01
1300	27673	aparc-a2009s_rh_thickness_S-circular-insula-sup	Destrieux Atlas	Lumbar spine	IVW	3	0.70	-0.35	0.14	1.18E-02	1.45E-01
1301	27674	aparc-a2009s_rh_thickness_S-collat-transv-ant	Destrieux Atlas	Forearm	WR	1	5.16	1.64	0.76	3.03E-02	4.50E-01
1301	27674	aparc-a2009s_rh_thickness_S-collat-transv-ant	Destrieux Atlas	Total body	IVW	2	0.66	-0.42	0.15	5.82E-03	2.28E-01
1304	27677	aparc-a2009s_rh_thickness_S-front-middle	Destrieux Atlas	Lumbar spine	IVW	2	0.72	-0.33	0.14	1.47E-02	1.63E-01
1305	27678	aparc-a2009s_rh_thickness_S-front-sup	Destrieux Atlas	Forearm	IVW	2	1.79	0.58	0.24	1.33E-02	4.47E-01
1307	27680	aparc-a2009s_rh_thickness_S-intrapariet+P-trans	Destrieux Atlas	Femoral neck	IVW	6	0.85	-0.16	0.07	2.01E-02	2.57E-01
1312	27685	aparc-a2009s_rh_thickness_S-oc-temp-med+Lingual	Destrieux Atlas	Heel	WR	1	1.15	0.14	0.04	5.72E-04	3.84E-03
1315	27688	aparc-a2009s_rh_thickness_S-orbital-H-Shaped	Destrieux Atlas	Heel	WR	1	0.76	-0.27	0.04	3.12E-12	4.72E-11
1317	27690	aparc-a2009s_rh_thickness_S-pericallosal	Destrieux Atlas	Femoral neck	IVW	4	0.84	-0.17	0.08	3.29E-02	2.68E-01

1322	27695	aparc-a2009s_rh_thickness_S-subparietal	Destrieux Atlas	Heel	IVW	4	0.91	-0.09	0.03	5.61E-04	3.84E-03
1324	27697	aparc-a2009s_rh_thickness_S-temporal-sup	Destrieux Atlas	Heel	WR	1	0.72	-0.33	0.03	8.38E-26	5.98E-24
1324	27697	aparc-a2009s_rh_thickness_S-temporal-sup	Destrieux Atlas	Lumbar spine	WR	1	0.54	-0.61	0.20	1.75E-03	3.56E-02
1325	27698	aparc-a2009s_rh_thickness_S-temporal-transverse	Destrieux Atlas	Total body	WR	1	0.76	-0.28	0.14	4.38E-02	4.01E-01
1325	27698	aparc-a2009s_rh_thickness_S-temporal-transverse	Destrieux Atlas	Femoral neck	WR	1	0.65	-0.43	0.17	1.30E-02	2.48E-01
1325	27698	aparc-a2009s_rh_thickness_S-temporal-transverse	Destrieux Atlas	Lumbar spine	WR	1	0.58	-0.55	0.20	6.48E-03	9.88E-02
576	27701	aparc-a2009s_rh_volume_G+S-paracentral	Destrieux Atlas	Heel	WR	1	1.20	0.18	0.04	4.36E-06	8.65E-05
581	27706	aparc-a2009s_rh_volume_G+S-cingul-Mid-Post	Destrieux Atlas	Femoral neck	IVW	2	1.51	0.41	0.20	4.25E-02	2.89E-01
584	27709	aparc-a2009s_rh_volume_G-cuneus	Destrieux Atlas	Forearm	IVW	20	1.22	0.2	0.07	2.90E-03	1.17E-01
585	27710	aparc-a2009s_rh_volume_G-front-inf-Opercular	Destrieux Atlas	Femoral neck	IVW	2	0.82	-0.2	0.10	4.91E-02	3.12E-01
586	27711	aparc-a2009s_rh_volume_G-front-inf-Orbital	Destrieux Atlas	Forearm	WR	1	2.46	0.9	0.36	1.26E-02	2.28E-01
586	27711	aparc-a2009s_rh_volume_G-front-inf-Orbital	Destrieux Atlas	Heel	WR	1	0.79	-0.24	0.04	9.21E-09	2.74E-07
589	27714	aparc-a2009s_rh_volume_G-front-sup	Destrieux Atlas	Heel	IVW	3	0.93	-0.07	0.02	5.58E-03	4.43E-02
590	27715	aparc-a2009s_rh_volume_G-Ins-lg+S-cent-ins	Destrieux Atlas	Heel	WR	1	0.86	-0.15	0.04	9.87E-05	1.47E-03
590	27715	aparc-a2009s_rh_volume_G-Ins-lg+S-cent-ins	Destrieux Atlas	Total body	WR	1	0.76	-0.28	0.13	3.83E-02	5.94E-01
591	27716	aparc-a2009s_rh_volume_G-insular-short	Destrieux Atlas	Lumbar spine	WR	1	1.52	0.42	0.19	2.86E-02	5.77E-01
591	27716	aparc-a2009s_rh_volume_G-insular-short	Destrieux Atlas	Forearm	WR	1	0.50	-0.69	0.34	3.99E-02	4.49E-01
594	27719	aparc-a2009s_rh_volume_G-oc-temp-lat-fusifor	Destrieux Atlas	Femoral neck	IVW	3	1.26	0.23	0.10	2.22E-02	2.74E-01
596	27721	aparc-a2009s_rh_volume_G-oc-temp-med-Parahip	Destrieux Atlas	Femoral neck	IVW	3	1.25	0.22	0.10	2.31E-02	2.74E-01
597	27722	aparc-a2009s_rh_volume_G-orbital	Destrieux Atlas	Forearm	IVW	2	2.25	0.81	0.24	9.67E-04	5.85E-02
597	27722	aparc-a2009s_rh_volume_G-orbital	Destrieux Atlas	Heel	IVW	2	0.94	-0.06	0.03	2.66E-02	1.58E-01
604	27729	aparc-a2009s_rh_volume_G-rectus	Destrieux Atlas	Total body	WR	1	1.40	0.34	0.13	9.49E-03	2.35E-01
604	27729	aparc-a2009s_rh_volume_G-rectus	Destrieux Atlas	Heel	WR	1	1.32	0.28	0.04	6.52E-12	7.76E-10
609	27734	aparc-a2009s_rh_volume_G-temp-sup-Plan-tempo	Destrieux Atlas	Forearm	IVW	2	0.49	-0.72	0.26	5.34E-03	1.29E-01
614	27739	aparc-a2009s_rh_volume_Lat-Fis-post	Destrieux Atlas	Total body	IVW	2	0.81	-0.21	0.09	1.54E-02	3.17E-01
616	27741	aparc-a2009s_rh_volume_Pole-temporal	Destrieux Atlas	Heel	WR	1	1.23	0.21	0.04	2.48E-07	5.90E-06
618	27743	aparc-a2009s_rh_volume_S-central	Destrieux Atlas	Femoral neck	IVW	3	1.07	0.07	0.03	3.63E-02	2.74E-01
620	27745	aparc-a2009s_rh_volume_S-circular-insula-ant	Destrieux Atlas	Heel	IVW	2	1.06	0.06	0.03	1.49E-02	1.04E-01
622	27747	aparc-a2009s_rh_volume_S-circular-insula-sup	Destrieux Atlas	Total body	IVW	2	0.83	-0.19	0.08	2.22E-02	3.94E-01
622	27747	aparc-a2009s_rh_volume_S-circular-insula-sup	Destrieux Atlas	Heel	IVW	2	0.77	-0.26	0.10	7.27E-03	5.41E-02
623	27748	aparc-a2009s_rh_volume_S-collat-transv-ant	Destrieux Atlas	Lumbar spine	IVW	2	1.35	0.3	0.10	3.11E-03	3.76E-01
624	27749	aparc-a2009s_rh_volume_S-collat-transv-post	Destrieux Atlas	Heel	WR	1	0.91	-0.09	0.04	2.30E-02	1.44E-01
626	27751	aparc-a2009s_rh_volume_S-front-middle	Destrieux Atlas	Forearm	WR	1	0.51	-0.68	0.33	3.73E-02	4.49E-01

628	27753	aparc-a2009s_rh_volume_S-interm-prim-Jensen	Destrieux Atlas	Femoral neck	WR	1	0.70	-0.35	0.16	3.32E-02	2.74E-01
630	27755	aparc-a2009s_rh_volume_S-oc-middle+Lunatus	Destrieux Atlas	Total body	IVW	6	1.17	0.16	0.06	6.70E-03	2.08E-01
630	27755	aparc-a2009s_rh_volume_S-oc-middle+Lunatus	Destrieux Atlas	Femoral neck	IVW	6	1.16	0.15	0.07	2.11E-02	2.74E-01
633	27758	aparc-a2009s_rh_volume_S-oc-temp-lat	Destrieux Atlas	Forearm	WR	1	0.47	-0.76	0.33	2.37E-02	3.59E-01
636	27761	aparc-a2009s_rh_volume_S-orbital-med-olfact	Destrieux Atlas	Heel	IVW	3	0.91	-0.09	0.03	1.87E-03	1.77E-02
637	27762	aparc-a2009s_rh_volume_S-orbital-H-Shaped	Destrieux Atlas	Heel	IVW	4	1.26	0.23	0.07	1.37E-03	1.49E-02
639	27764	aparc-a2009s_rh_volume_S-pericallosal	Destrieux Atlas	Forearm	WR	1	0.39	-0.95	0.38	1.32E-02	2.28E-01
640	27765	aparc-a2009s_rh_volume_S-postcentral	Destrieux Atlas	Lumbar spine	IVW	3	1.19	0.17	0.08	2.62E-02	5.77E-01
641	27766	aparc-a2009s_rh_volume_S-precentral-inf-part	Destrieux Atlas	Forearm	IVW	2	0.38	-0.97	0.25	9.26E-05	1.12E-02
642	27767	aparc-a2009s_rh_volume_S-precentral-sup-part	Destrieux Atlas	Femoral neck	WR	1	1.22	0.2	0.09	2.69E-02	2.74E-01
