# Supplementary material for: Causal associations of brain structure with bone mineral density: a large-scale genetic correlation study
Source: Bone Res. 2023 Jul 20;11:37. doi: 10.1038/s41413-023-00270-z (PMC10359275; doi:10.1038/s41413-023-00270-z)
Supplement: Supplementary file 5 — Supplementary Table 3. Results of sensitivity analysis of MR [file 41413_2023_270_MOESM5_ESM.pdf]

Supplemental table 3. Results of sensitivity analysis of MR

NO.	UKB ID	IDP short name	Category name	outcome	method	nsnp	OR	CI95L	CI95U	pval
661	26734	aparc-Desikan_lh_area_medialorbitofrontal	Desikan Atlas	Lumbar spine BMD	Wald ratio	1	0.53	0.36	0.78	1.10E-03
661	26734	aparc-Desikan_lh_area_medialorbitofrontal	Desikan Atlas	Femoral neck BMD	Wald ratio	1	0.87	0.63	1.20	3.91E-01
661	26734	aparc-Desikan_lh_area_medialorbitofrontal	Desikan Atlas	Forearm BMD	Wald ratio	1	0.64	0.32	1.25	1.92E-01
661	26734	aparc-Desikan_lh_area_medialorbitofrontal	Desikan Atlas	Heel BMD	Wald ratio	1	0.61	0.56	0.66	1.45E-34
661	26734	aparc-Desikan_lh_area_medialorbitofrontal	Desikan Atlas	Total body BMD	Wald ratio	1	0.38	0.30	0.49	6.38E-14
187	26536	aseg_global_volume-ratio_BrainSegVol-to-eTIV	aseg:global	Lumbar spine BMD	IVW	15	1.70	1.42	2.04	1.08E-08
187	26536	aseg_global_volume-ratio_BrainSegVol-to-eTIV	aseg:global	Lumbar spine BMD	MR Egger	15	1.64	0.94	2.85	1.01E-01
187	26536	aseg_global_volume-ratio_BrainSegVol-to-eTIV	aseg:global	Lumbar spine BMD	Simple mode	15	1.77	1.36	2.30	5.83E-04
187	26536	aseg_global_volume-ratio_BrainSegVol-to-eTIV	aseg:global	Lumbar spine BMD	Weighted median	15	1.65	1.46	1.87	5.15E-15
187	26536	aseg_global_volume-ratio_BrainSegVol-to-eTIV	aseg:global	Lumbar spine BMD	Weighted mode	15	1.64	1.45	1.86	9.18E-07
187	26536	aseg_global_volume-ratio_BrainSegVol-to-eTIV	aseg:global	Femoral neck BMD	IVW	15	1.51	1.30	1.75	4.08E-08
187	26536	aseg_global_volume-ratio_BrainSegVol-to-eTIV	aseg:global	Femoral neck BMD	MR Egger	15	1.47	0.94	2.32	1.13E-01
187	26536	aseg_global_volume-ratio_BrainSegVol-to-eTIV	aseg:global	Femoral neck BMD	Simple mode	15	1.70	1.31	2.19	8.91E-04
187	26536	aseg_global_volume-ratio_BrainSegVol-to-eTIV	aseg:global	Femoral neck BMD	Weighted median	15	1.50	1.35	1.67	1.07E-13
187	26536	aseg_global_volume-ratio_BrainSegVol-to-eTIV	aseg:global	Femoral neck BMD	Weighted mode	15	1.53	1.33	1.76	2.01E-05
187	26536	aseg_global_volume-ratio_BrainSegVol-to-eTIV	aseg:global	Forearm BMD	IVW	15	1.97	1.56	2.48	7.98E-09
187	26536	aseg_global_volume-ratio_BrainSegVol-to-eTIV	aseg:global	Forearm BMD	MR Egger	15	3.95	2.17	7.19	4.19E-04
187	26536	aseg_global_volume-ratio_BrainSegVol-to-eTIV	aseg:global	Forearm BMD	Simple mode	15	1.40	0.92	2.11	1.33E-01
187	26536	aseg_global_volume-ratio_BrainSegVol-to-eTIV	aseg:global	Forearm BMD	Weighted median	15	1.56	1.24	1.96	1.56E-04
187	26536	aseg_global_volume-ratio_BrainSegVol-to-eTIV	aseg:global	Forearm BMD	Weighted mode	15	1.38	0.84	2.28	2.27E-01
187	26536	aseg_global_volume-ratio_BrainSegVol-to-eTIV	aseg:global	Heel BMD	IVW	15	2.01	1.62	2.50	3.04E-10
187	26536	aseg_global_volume-ratio_BrainSegVol-to-eTIV	aseg:global	Heel BMD	MR Egger	15	3.08	1.79	5.31	1.03E-03
187	26536	aseg_global_volume-ratio_BrainSegVol-to-eTIV	aseg:global	Heel BMD	Simple mode	15	1.76	1.42	2.17	7.99E-05
187	26536	aseg_global_volume-ratio_BrainSegVol-to-eTIV	aseg:global	Heel BMD	Weighted median	15	1.35	1.27	1.44	1.72E-20
187	26536	aseg_global_volume-ratio_BrainSegVol-to-eTIV	aseg:global	Heel BMD	Weighted mode	15	1.33	1.26	1.41	1.76E-08
187	26536	aseg_global_volume-ratio_BrainSegVol-to-eTIV	aseg:global	Total body BMD	IVW	17	2.29	1.98	2.66	4.49E-28
187	26536	aseg_global_volume-ratio_BrainSegVol-to-eTIV	aseg:global	Total body BMD	MR Egger	17	2.97	2.04	4.32	2.65E-05
187	26536	aseg_global_volume-ratio_BrainSegVol-to-eTIV	aseg:global	Total body BMD	Simple mode	17	2.45	1.93	3.11	7.10E-07
187	26536	aseg_global_volume-ratio_BrainSegVol-to-eTIV	aseg:global	Total body BMD	Weighted median	17	2.17	1.89	2.48	2.54E-29
187	26536	aseg_global_volume-ratio_BrainSegVol-to-eTIV	aseg:global	Total body BMD	Weighted mode	17	2.36	1.98	2.80	1.25E-08

36	25792	IDP_T1_FAST_ROIs_L_inf_front_gyrus_parsop	IDP T1:unilateral regions	Lumbar spine BMD	Wald ratio	1	1.75	1.22	2.53	1.92E-03
36	25792	IDP_T1_FAST_ROIs_L_inf_front_gyrus_parsop	IDP T1:unilateral regions	Femoral neck BMD	Wald ratio	1	1.96	1.43	2.70	2.33E-05
36	25792	IDP_T1_FAST_ROIs_L_inf_front_gyrus_parsop	IDP T1:unilateral regions	Forearm BMD	Wald ratio	1	1.79	0.93	3.43	8.03E-02
36	25792	IDP_T1_FAST_ROIs_L_inf_front_gyrus_parsop	IDP T1:unilateral regions	Heel BMD	Wald ratio	1	1.52	1.39	1.65	6.53E-23
36	25792	IDP_T1_FAST_ROIs_L_inf_front_gyrus_parsop	IDP T1:unilateral regions	Total body BMD	Wald ratio	1	1.69	1.33	2.16	2.57E-05
356	26801	aparc-Desikan_lh_volume_medialorbitofrontal	Desikan Atlas	Lumbar spine BMD	Wald ratio	1	1.85	1.26	2.72	2.11E-03
356	26801	aparc-Desikan_lh_volume_medialorbitofrontal	Desikan Atlas	Femoral neck BMD	Wald ratio	1	1.45	1.04	2.03	2.97E-02
356	26801	aparc-Desikan_lh_volume_medialorbitofrontal	Desikan Atlas	Forearm BMD	Wald ratio	1	1.00	0.50	2.00	9.96E-01
356	26801	aparc-Desikan_lh_volume_medialorbitofrontal	Desikan Atlas	Heel BMD	Wald ratio	1	1.59	1.47	1.72	3.36E-28
356	26801	aparc-Desikan_lh_volume_medialorbitofrontal	Desikan Atlas	Total body BMD	Wald ratio	1	1.52	1.17	1.97	1.61E-03
1033	26768	aparc-Desikan_lh_thickness_medialorbitofrontal	Desikan Atlas	Lumbar spine BMD	Wald ratio	1	0.43	0.26	0.69	5.36E-04
1033	26768	aparc-Desikan_lh_thickness_medialorbitofrontal	Desikan Atlas	Femoral neck BMD	Wald ratio	1	0.67	0.46	0.97	3.65E-02
1033	26768	aparc-Desikan_lh_thickness_medialorbitofrontal	Desikan Atlas	Forearm BMD	Wald ratio	1	0.67	0.22	2.05	4.87E-01
1033	26768	aparc-Desikan_lh_thickness_medialorbitofrontal	Desikan Atlas	Heel BMD	Wald ratio	1	0.66	0.61	0.71	2.01E-25
1033	26768	aparc-Desikan_lh_thickness_medialorbitofrontal	Desikan Atlas	Total body BMD	Wald ratio	1	0.76	0.57	1.03	7.67E-02
1038	26773	aparc-Desikan_lh_thickness_parsorbitalis	Desikan Atlas	Lumbar spine BMD	Wald ratio	1	0.39	0.23	0.67	6.53E-04
1038	26773	aparc-Desikan_lh_thickness_parsorbitalis	Desikan Atlas	Femoral neck BMD	Wald ratio	1	0.68	0.43	1.10	1.20E-01
1038	26773	aparc-Desikan_lh_thickness_parsorbitalis	Desikan Atlas	Forearm BMD	Wald ratio	1	0.61	0.18	2.09	4.32E-01
1038	26773	aparc-Desikan_lh_thickness_parsorbitalis	Desikan Atlas	Heel BMD	Wald ratio	1	0.64	0.58	0.69	7.20E-26
1038	26773	aparc-Desikan_lh_thickness_parsorbitalis	Desikan Atlas	Total body BMD	Wald ratio	1	0.59	0.41	0.85	4.19E-03
656	26729	aparc-Desikan_lh_area_inferiortemporal	Desikan Atlas	Lumbar spine BMD	Wald ratio	1	2.08	1.26	3.44	4.05E-03
656	26729	aparc-Desikan_lh_area_inferiortemporal	Desikan Atlas	Femoral neck BMD	Wald ratio	1	1.39	0.93	2.08	1.16E-01
656	26729	aparc-Desikan_lh_area_inferiortemporal	Desikan Atlas	Forearm BMD	Wald ratio	1	1.30	0.39	4.35	6.75E-01
656	26729	aparc-Desikan_lh_area_inferiortemporal	Desikan Atlas	Heel BMD	Wald ratio	1	1.54	1.42	1.66	7.69E-26
656	26729	aparc-Desikan_lh_area_inferiortemporal	Desikan Atlas	Total body BMD	Wald ratio	1	1.43	1.06	1.93	2.28E-02
816	27149	aparc-DKTatlas_lh_area_inferiortemporal	Desikan Atlas	Lumbar spine BMD	Wald ratio	1	2.44	1.52	3.92	2.90E-04
816	27149	aparc-DKTatlas_lh_area_inferiortemporal	Desikan Atlas	Femoral neck BMD	Wald ratio	1	1.47	1.01	2.15	4.90E-02
816	27149	aparc-DKTatlas_lh_area_inferiortemporal	Desikan Atlas	Forearm BMD	Wald ratio	1	1.72	0.53	5.56	3.61E-01

816	27149	aparc-DKTatlas_lh_area_inferiortemporal	Desikan Atlas	Heel BMD	Wald ratio	1	1.52	1.40	1.63	7.41E-26
1183	27408	aparc-a2009s_lh_thickness_G+S-cingul-Ant	Destrieux Atlas	Lumbar spine BMD	Wald ratio	1	0.52	0.35	0.75	5.36E-04
1183	27408	aparc-a2009s_lh_thickness_G+S-cingul-Ant	Destrieux Atlas	Femoral neck BMD	Wald ratio	1	0.73	0.54	0.98	3.65E-02
1183	27408	aparc-a2009s_lh_thickness_G+S-cingul-Ant	Destrieux Atlas	Forearm BMD	Wald ratio	1	0.74	0.31	1.75	4.87E-01
1183	27408	aparc-a2009s_lh_thickness_G+S-cingul-Ant	Destrieux Atlas	Heel BMD	Wald ratio	1	0.72	0.68	0.77	9.89E-26
1183	27408	aparc-a2009s_lh_thickness_G+S-cingul-Ant	Destrieux Atlas	Total body BMD	Wald ratio	1	0.81	0.65	1.02	7.67E-02
1324	27697	aparc-a2009s_rh_thickness_S-temporal-sup	Destrieux Atlas	Lumbar spine BMD	Wald ratio	1	0.54	0.37	0.80	1.75E-03
1324	27697	aparc-a2009s_rh_thickness_S-temporal-sup	Destrieux Atlas	Femoral neck BMD	Wald ratio	1	0.81	0.57	1.16	2.59E-01
1324	27697	aparc-a2009s_rh_thickness_S-temporal-sup	Destrieux Atlas	Forearm BMD	Wald ratio	1	0.68	0.32	1.41	2.95E-01
1324	27697	aparc-a2009s_rh_thickness_S-temporal-sup	Destrieux Atlas	Heel BMD	Wald ratio	1	0.72	0.68	0.76	8.38E-26
1324	27697	aparc-a2009s_rh_thickness_S-temporal-sup	Destrieux Atlas	Total body BMD	Wald ratio	1	0.81	0.54	1.21	2.95E-01
1237	27462	aparc-a2009s_lh_thickness_S-oc-temp-lat	Destrieux Atlas	Lumbar spine BMD	Wald ratio	1	0.54	0.39	0.74	1.74E-04
1237	27462	aparc-a2009s_lh_thickness_S-oc-temp-lat	Destrieux Atlas	Femoral neck BMD	Wald ratio	1	0.78	0.60	1.02	7.04E-02
1237	27462	aparc-a2009s_lh_thickness_S-oc-temp-lat	Destrieux Atlas	Forearm BMD	Wald ratio	1	0.80	0.43	1.50	4.83E-01
1237	27462	aparc-a2009s_lh_thickness_S-oc-temp-lat	Destrieux Atlas	Heel BMD	Wald ratio	1	0.72	0.67	0.77	3.74E-25
1237	27462	aparc-a2009s_lh_thickness_S-oc-temp-lat	Destrieux Atlas	Total body BMD	Wald ratio	1	0.75	0.60	0.94	1.07E-02
1249	27474	aparc-a2009s_lh_thickness_S-temporal-inf	Destrieux Atlas	Lumbar spine BMD	Wald ratio	1	0.47	0.32	0.70	1.74E-04
1249	27474	aparc-a2009s_lh_thickness_S-temporal-inf	Destrieux Atlas	Femoral neck BMD	Wald ratio	1	0.74	0.53	1.03	7.04E-02
1249	27474	aparc-a2009s_lh_thickness_S-temporal-inf	Destrieux Atlas	Forearm BMD	Wald ratio	1	0.76	0.35	1.63	4.83E-01
1249	27474	aparc-a2009s_lh_thickness_S-temporal-inf	Destrieux Atlas	Heel BMD	Wald ratio	1	0.67	0.62	0.72	3.74E-25
1249	27474	aparc-a2009s_lh_thickness_S-temporal-inf	Destrieux Atlas	Total body BMD	Wald ratio	1	0.70	0.54	0.92	1.07E-02
469	27298	aparc-DKTatlas_rh_volume_caudalanteriorcingulate	Desikan Atlas	Lumbar spine BMD	Wald ratio	1	0.65	0.45	0.94	2.03E-02
469	27298	aparc-DKTatlas_rh_volume_caudalanteriorcingulate	Desikan Atlas	Femoral neck BMD	Wald ratio	1	0.87	0.63	1.19	3.81E-01
469	27298	aparc-DKTatlas_rh_volume_caudalanteriorcingulate	Desikan Atlas	Forearm BMD	Wald ratio	1	0.88	0.46	1.70	7.10E-01
469	27298	aparc-DKTatlas_rh_volume_caudalanteriorcingulate	Desikan Atlas	Heel BMD	Wald ratio	1	0.67	0.62	0.72	1.11E-24
469	27298	aparc-DKTatlas_rh_volume_caudalanteriorcingulate	Desikan Atlas	Total body BMD	Wald ratio	1	0.66	0.52	0.84	7.56E-04
1247	27472	aparc-a2009s_lh_thickness_S-suborbital	Destrieux Atlas	Lumbar spine BMD	Wald ratio	1	0.58	0.36	0.93	2.35E-02
1247	27472	aparc-a2009s_lh_thickness_S-suborbital	Destrieux Atlas	Femoral neck BMD	Wald ratio	1	0.83	0.55	1.26	4.02E-01
1247	27472	aparc-a2009s_lh_thickness_S-suborbital	Destrieux Atlas	Forearm BMD	Wald ratio	1	0.66	0.23	1.88	4.35E-01
1247	27472	aparc-a2009s_lh_thickness_S-suborbital	Destrieux Atlas	Heel BMD	Wald ratio	1	0.68	0.63	0.73	5.33E-24
1247	27472	aparc-a2009s_lh_thickness_S-suborbital	Destrieux Atlas	Total body BMD	Wald ratio	1	0.76	0.57	1.03	7.56E-02
1013	27618	aparc-a2009s_rh_area_S-precentral-inf-part	Destrieux Atlas	Lumbar spine BMD	Wald ratio	1	2.63	1.59	4.36	2.29E-04
1013	27618	aparc-a2009s_rh_area_S-precentral-inf-part	Destrieux Atlas	Femoral neck BMD	Wald ratio	1	1.43	0.94	2.17	9.36E-02

1013	27618	aparc-a2009s_rh_area_S-precentral-inf-part	Destrieux Atlas	Forearm BMD	Wald ratio	1	1.33	0.44	4.00	6.03E-01
1013	27618	aparc-a2009s_rh_area_S-precentral-inf-part	Destrieux Atlas	Heel BMD	Wald ratio	1	1.45	1.34	1.57	8.42E-19
1013	27618	aparc-a2009s_rh_area_S-precentral-inf-part	Destrieux Atlas	Total body BMD	Wald ratio	1	1.37	1.01	1.85	4.27E-02
1190	27415	aparc-a2009s_lh_thickness_G-front-inf-Orbital	Destrieux Atlas	Lumbar spine BMD	Wald ratio	1	1.10	0.76	1.59	6.32E-01
1190	27415	aparc-a2009s_lh_thickness_G-front-inf-Orbital	Destrieux Atlas	Femoral neck BMD	Wald ratio	1	0.91	0.64	1.28	5.90E-01
1190	27415	aparc-a2009s_lh_thickness_G-front-inf-Orbital	Destrieux Atlas	Forearm BMD	Wald ratio	1	1.67	0.87	3.21	1.23E-01
1190	27415	aparc-a2009s_lh_thickness_G-front-inf-Orbital	Destrieux Atlas	Heel BMD	Wald ratio	1	1.39	1.29	1.50	2.33E-17
1190	27415	aparc-a2009s_lh_thickness_G-front-inf-Orbital	Destrieux Atlas	Total body BMD	Wald ratio	1	1.10	0.87	1.39	4.66E-01
1241	27466	aparc-a2009s_lh_thickness_S-orbital-H-Shaped	Destrieux Atlas	Lumbar spine BMD	IVW	2	0.66	0.41	1.08	9.74E-02
1241	27466	aparc-a2009s_lh_thickness_S-orbital-H-Shaped	Destrieux Atlas	Femoral neck BMD	IVW	2	0.92	0.64	1.31	6.27E-01
1241	27466	aparc-a2009s_lh_thickness_S-orbital-H-Shaped	Destrieux Atlas	Forearm BMD	IVW	2	0.80	0.48	1.33	3.87E-01
1241	27466	aparc-a2009s_lh_thickness_S-orbital-H-Shaped	Destrieux Atlas	Heel BMD	IVW	2	0.75	0.70	0.80	3.78E-17
1241	27466	aparc-a2009s_lh_thickness_S-orbital-H-Shaped	Destrieux Atlas	Total body BMD	IVW	2	0.93	0.57	1.50	7.49E-01
1315	27688	aparc-a2009s_rh_thickness_S-orbital-H-Shaped	Destrieux Atlas	Lumbar spine BMD	Wald ratio	1	0.93	0.61	1.41	7.08E-01
1315	27688	aparc-a2009s_rh_thickness_S-orbital-H-Shaped	Destrieux Atlas	Femoral neck BMD	Wald ratio	1	1.11	0.76	1.62	5.82E-01
1315	27688	aparc-a2009s_rh_thickness_S-orbital-H-Shaped	Destrieux Atlas	Forearm BMD	Wald ratio	1	0.72	0.37	1.41	3.42E-01
1315	27688	aparc-a2009s_rh_thickness_S-orbital-H-Shaped	Destrieux Atlas	Heel BMD	Wald ratio	1	0.76	0.71	0.82	3.12E-12
1315	27688	aparc-a2009s_rh_thickness_S-orbital-H-Shaped	Destrieux Atlas	Total body BMD	Wald ratio	1	1.18	0.92	1.50	1.82E-01
604	27729	aparc-a2009s_rh_volume_G-rectus	Destrieux Atlas	Lumbar spine BMD	Wald ratio	1	0.86	0.56	1.32	4.82E-01
604	27729	aparc-a2009s_rh_volume_G-rectus	Destrieux Atlas	Femoral neck BMD	Wald ratio	1	1.05	0.74	1.50	7.94E-01
604	27729	aparc-a2009s_rh_volume_G-rectus	Destrieux Atlas	Forearm BMD	Wald ratio	1	0.78	0.35	1.74	5.51E-01
604	27729	aparc-a2009s_rh_volume_G-rectus	Destrieux Atlas	Heel BMD	Wald ratio	1	1.32	1.22	1.42	6.52E-12
604	27729	aparc-a2009s_rh_volume_G-rectus	Destrieux Atlas	Total body BMD	Wald ratio	1	1.41	1.09	1.82	9.49E-03
707	26847	aparc-Desikan_rh_area_rostralanteriorcingulate	Desikan Atlas	Lumbar spine BMD	IVW	2	1.10	0.72	1.67	6.75E-01
707	26847	aparc-Desikan_rh_area_rostralanteriorcingulate	Desikan Atlas	Femoral neck BMD	IVW	2	1.12	0.90	1.40	3.17E-01
707	26847	aparc-Desikan_rh_area_rostralanteriorcingulate	Desikan Atlas	Forearm BMD	IVW	2	1.00	0.57	1.75	9.97E-01
707	26847	aparc-Desikan_rh_area_rostralanteriorcingulate	Desikan Atlas	Heel BMD	IVW	2	1.18	1.12	1.23	4.56E-10
707	26847	aparc-Desikan_rh_area_rostralanteriorcingulate	Desikan Atlas	Total body BMD	IVW	2	1.15	0.90	1.46	2.43E-01
810	27143	aparc-DKTatlas_lh_area_caudalanteriorcingulate	Desikan Atlas	Lumbar spine BMD	Wald ratio	1	1.49	1.11	2.01	6.80E-03
810	27143	aparc-DKTatlas_lh_area_caudalanteriorcingulate	Desikan Atlas	Femoral neck BMD	Wald ratio	1	1.64	1.27	2.11	1.25E-04
810	27143	aparc-DKTatlas_lh_area_caudalanteriorcingulate	Desikan Atlas	Forearm BMD	Wald ratio	1	1.72	1.02	2.93	4.08E-02
810	27143	aparc-DKTatlas_lh_area_caudalanteriorcingulate	Desikan Atlas	Heel BMD	Wald ratio	1	1.22	1.15	1.30	4.51E-10
810	27143	aparc-DKTatlas_lh_area_caudalanteriorcingulate	Desikan Atlas	Total body BMD	Wald ratio	1	1.54	1.27	1.86	1.30E-05

390	26903	aparc-Desikan_rh_volume_middletemporal	Desikan Atlas	Lumbar spine BMD	IVW	2	0.83	0.55	1.25	3.87E-01
390	26903	aparc-Desikan_rh_volume_middletemporal	Desikan Atlas	Femoral neck BMD	IVW	2	0.89	0.58	1.37	6.06E-01
390	26903	aparc-Desikan_rh_volume_middletemporal	Desikan Atlas	Forearm BMD	IVW	2	0.79	0.27	2.33	6.79E-01
390	26903	aparc-Desikan_rh_volume_middletemporal	Desikan Atlas	Heel BMD	Wald ratio	1	1.28	1.19	1.38	5.03E-10
390	26903	aparc-Desikan_rh_volume_middletemporal	Desikan Atlas	Total body BMD	IVW	2	0.96	0.77	1.20	7.32E-01
48	25804	IDP_T1_FAST_ROIs_L_mid_temp_gyrus_post	IDP T1:unilateral regions	Lumbar spine BMD	Wald ratio	1	1.03	0.70	1.53	8.63E-01
48	25804	IDP_T1_FAST_ROIs_L_mid_temp_gyrus_post	IDP T1:unilateral regions	Femoral neck BMD	Wald ratio	1	1.12	0.80	1.58	5.03E-01
48	25804	IDP_T1_FAST_ROIs_L_mid_temp_gyrus_post	IDP T1:unilateral regions	Forearm BMD	Wald ratio	1	1.43	0.71	2.86	3.11E-01
48	25804	IDP_T1_FAST_ROIs_L_mid_temp_gyrus_post	IDP T1:unilateral regions	Heel BMD	Wald ratio	1	1.30	1.20	1.40	5.03E-10
48	25804	IDP_T1_FAST_ROIs_L_mid_temp_gyrus_post	IDP T1:unilateral regions	Total body BMD	Wald ratio	1	1.09	0.83	1.42	5.26E-01
70	25826	IDP_T1_FAST_ROIs_L_latocc_cortex_inf	IDP T1:unilateral regions	Lumbar spine BMD	Wald ratio	1	1.03	0.73	1.45	8.63E-01
70	25826	IDP_T1_FAST_ROIs_L_latocc_cortex_inf	IDP T1:unilateral regions	Femoral neck BMD	Wald ratio	1	1.11	0.83	1.49	5.03E-01
70	25826	IDP_T1_FAST_ROIs_L_latocc_cortex_inf	IDP T1:unilateral regions	Forearm BMD	Wald ratio	1	1.37	0.76	2.47	3.11E-01
70	25826	IDP_T1_FAST_ROIs_L_latocc_cortex_inf	IDP T1:unilateral regions	Heel BMD	Wald ratio	1	1.25	1.17	1.34	5.03E-10
70	25826	IDP_T1_FAST_ROIs_L_latocc_cortex_inf	IDP T1:unilateral regions	Total body BMD	Wald ratio	1	1.08	0.86	1.35	5.26E-01
506	27483	aparc-a2009s_lh_volume_G+S-cingul-Mid-Ant	Destrieux Atlas	Lumbar spine BMD	Wald ratio	1	1.61	1.14	2.28	6.80E-03
506	27483	aparc-a2009s_lh_volume_G+S-cingul-Mid-Ant	Destrieux Atlas	Femoral neck BMD	Wald ratio	1	1.79	1.32	2.42	1.25E-04
506	27483	aparc-a2009s_lh_volume_G+S-cingul-Mid-Ant	Destrieux Atlas	Forearm BMD	Wald ratio	1	1.92	1.03	3.58	4.08E-02
506	27483	aparc-a2009s_lh_volume_G+S-cingul-Mid-Ant	Destrieux Atlas	Heel BMD	Wald ratio	1	1.27	1.17	1.37	4.51E-10
506	27483	aparc-a2009s_lh_volume_G+S-cingul-Mid-Ant	Destrieux Atlas	Total body BMD	Wald ratio	1	1.67	1.32	2.10	1.30E-05
51	25807	IDP_T1_FAST_ROIs_R_mid_temp_gyrus_tempocc	IDP T1:unilateral regions	Lumbar spine BMD	Wald ratio	1	1.09	0.72	1.63	6.76E-01
51	25807	IDP_T1_FAST_ROIs_R_mid_temp_gyrus_tempocc	IDP T1:unilateral	Femoral neck BMD	Wald ratio	1	1.09	0.77	1.54	6.62E-01

			regions							
51	25807	IDP_T1_FAST_ROIs_R_mid_temp_gyrus_tempocc	IDP T1:unilateral regions	Forearm BMD	Wald ratio	1	1.35	0.66	2.79	4.18E-01
51	25807	IDP_T1_FAST_ROIs_R_mid_temp_gyrus_tempocc	IDP T1:unilateral regions	Heel BMD	Wald ratio	1	1.30	1.19	1.41	1.69E-09
51	25807	IDP_T1_FAST_ROIs_R_mid_temp_gyrus_tempocc	IDP T1:unilateral regions	Total body BMD	Wald ratio	1	1.09	0.82	1.43	5.72E-01
505	27482	aparc-a2009s_lh_volume_G+S-cingul-Ant	Destrieux Atlas	Lumbar spine BMD	Wald ratio	1	1.45	0.80	2.62	2.15E-01
505	27482	aparc-a2009s_lh_volume_G+S-cingul-Ant	Destrieux Atlas	Femoral neck BMD	Wald ratio	1	1.85	1.06	3.22	2.83E-02
505	27482	aparc-a2009s_lh_volume_G+S-cingul-Ant	Destrieux Atlas	Forearm BMD	Wald ratio	1	2.33	0.82	6.63	1.13E-01
505	27482	aparc-a2009s_lh_volume_G+S-cingul-Ant	Destrieux Atlas	Heel BMD	Wald ratio	1	1.30	1.19	1.41	4.51E-10
505	27482	aparc-a2009s_lh_volume_G+S-cingul-Ant	Destrieux Atlas	Total body BMD	Wald ratio	1	1.72	1.32	2.25	5.25E-05
586	27711	aparc-a2009s_rh_volume_G-front-inf-Orbital	Destrieux Atlas	Lumbar spine BMD	Wald ratio	1	1.12	0.76	1.67	5.54E-01
586	27711	aparc-a2009s_rh_volume_G-front-inf-Orbital	Destrieux Atlas	Femoral neck BMD	Wald ratio	1	1.32	0.94	1.84	1.07E-01
586	27711	aparc-a2009s_rh_volume_G-front-inf-Orbital	Destrieux Atlas	Forearm BMD	Wald ratio	1	2.50	1.23	5.06	1.26E-02
586	27711	aparc-a2009s_rh_volume_G-front-inf-Orbital	Destrieux Atlas	Heel BMD	Wald ratio	1	0.79	0.73	0.86	9.21E-09
586	27711	aparc-a2009s_rh_volume_G-front-inf-Orbital	Destrieux Atlas	Total body BMD	Wald ratio	1	1.10	0.85	1.42	4.60E-01
1185	27410	aparc-a2009s_lh_thickness_G+S-cingul-Mid-Post	Destrieux Atlas	Lumbar spine BMD	Wald ratio	1	1.02	0.67	1.56	9.17E-01
1185	27410	aparc-a2009s_lh_thickness_G+S-cingul-Mid-Post	Destrieux Atlas	Femoral neck BMD	Wald ratio	1	0.94	0.63	1.40	7.90E-01
1185	27410	aparc-a2009s_lh_thickness_G+S-cingul-Mid-Post	Destrieux Atlas	Forearm BMD	Wald ratio	1	0.97	0.48	1.95	9.44E-01
1185	27410	aparc-a2009s_lh_thickness_G+S-cingul-Mid-Post	Destrieux Atlas	Heel BMD	Wald ratio	1	0.79	0.73	0.86	2.54E-08
1185	27410	aparc-a2009s_lh_thickness_G+S-cingul-Mid-Post	Destrieux Atlas	Total body BMD	Wald ratio	1	1.05	0.81	1.36	6.83E-01
933	27390	aparc-a2009s_lh_area_S-orbital-lateral	Destrieux Atlas	Lumbar spine BMD	Wald ratio	1	1.09	0.81	1.45	5.79E-01
933	27390	aparc-a2009s_lh_area_S-orbital-lateral	Destrieux Atlas	Femoral neck BMD	Wald ratio	1	1.23	0.96	1.59	1.06E-01
933	27390	aparc-a2009s_lh_area_S-orbital-lateral	Destrieux Atlas	Forearm BMD	Wald ratio	1	2.04	1.19	3.49	1.04E-02
933	27390	aparc-a2009s_lh_area_S-orbital-lateral	Destrieux Atlas	Heel BMD	Wald ratio	1	0.83	0.79	0.88	9.08E-09
933	27390	aparc-a2009s_lh_area_S-orbital-lateral	Destrieux Atlas	Total body BMD	Wald ratio	1	1.06	0.87	1.29	5.15E-01
475	27304	aparc-DKTatlas_rh_volume_inferiortemporal	Desikan Atlas	Lumbar spine BMD	Wald ratio	1	1.08	0.73	1.59	7.23E-01
475	27304	aparc-DKTatlas_rh_volume_inferiortemporal	Desikan Atlas	Femoral neck BMD	Wald ratio	1	1.05	0.75	1.47	7.56E-01
475	27304	aparc-DKTatlas_rh_volume_inferiortemporal	Desikan Atlas	Forearm BMD	Wald ratio	1	0.78	0.38	1.56	4.69E-01
475	27304	aparc-DKTatlas_rh_volume_inferiortemporal	Desikan Atlas	Heel BMD	Wald ratio	1	1.27	1.17	1.37	3.51E-08
475	27304	aparc-DKTatlas_rh_volume_inferiortemporal	Desikan Atlas	Total body BMD	Wald ratio	1	1.32	1.02	1.70	3.87E-02
616	27741	aparc-a2009s_rh_volume_Pole-temporal	Destrieux Atlas	Lumbar spine BMD	Wald ratio	1	1.10	0.75	1.61	6.28E-01

616	27741	aparc-a2009s_rh_volume_Pole-temporal	Destrieux Atlas	Femoral neck BMD	Wald ratio	1	1.22	0.88	1.70	2.33E-01
616	27741	aparc-a2009s_rh_volume_Pole-temporal	Destrieux Atlas	Forearm BMD	Wald ratio	1	0.56	0.28	1.09	9.01E-02
616	27741	aparc-a2009s_rh_volume_Pole-temporal	Destrieux Atlas	Heel BMD	Wald ratio	1	1.23	1.14	1.34	2.48E-07
616	27741	aparc-a2009s_rh_volume_Pole-temporal	Destrieux Atlas	Total body BMD	Wald ratio	1	1.14	0.89	1.45	3.09E-01
1219	27444	aparc-a2009s_lh_thickness_Pole-occipital	Destrieux Atlas	Lumbar spine BMD	IVW	2	0.81	0.67	0.97	2.14E-02
1219	27444	aparc-a2009s_lh_thickness_Pole-occipital	Destrieux Atlas	Femoral neck BMD	IVW	2	0.87	0.74	1.02	7.48E-02
1219	27444	aparc-a2009s_lh_thickness_Pole-occipital	Destrieux Atlas	Forearm BMD	IVW	2	0.88	0.64	1.22	4.57E-01
1219	27444	aparc-a2009s_lh_thickness_Pole-occipital	Destrieux Atlas	Heel BMD	IVW	2	1.11	1.07	1.15	9.58E-07
1219	27444	aparc-a2009s_lh_thickness_Pole-occipital	Destrieux Atlas	Total body BMD	IVW	2	0.92	0.80	1.05	1.77E-01
864	27259	aparc-DKTatlas_rh_area_rostralanteriorcingulate	Desikan Atlas	Lumbar spine BMD	Wald ratio	1	1.27	0.93	1.72	1.29E-01
864	27259	aparc-DKTatlas_rh_area_rostralanteriorcingulate	Desikan Atlas	Femoral neck BMD	Wald ratio	1	1.18	0.91	1.52	2.08E-01
864	27259	aparc-DKTatlas_rh_area_rostralanteriorcingulate	Desikan Atlas	Forearm BMD	Wald ratio	1	1.23	0.72	2.13	4.49E-01
864	27259	aparc-DKTatlas_rh_area_rostralanteriorcingulate	Desikan Atlas	Heel BMD	Wald ratio	1	1.16	1.09	1.24	8.17E-07
864	27259	aparc-DKTatlas_rh_area_rostralanteriorcingulate	Desikan Atlas	Total body BMD	Wald ratio	1	1.25	1.00	1.56	4.55E-02
1199	27424	aparc-a2009s_lh_thickness_G-oc-temp-med-Lingual	Destrieux Atlas	Lumbar spine BMD	Wald ratio	1	0.79	0.59	1.06	1.25E-01
1199	27424	aparc-a2009s_lh_thickness_G-oc-temp-med-Lingual	Destrieux Atlas	Femoral neck BMD	Wald ratio	1	0.85	0.66	1.09	1.98E-01
1199	27424	aparc-a2009s_lh_thickness_G-oc-temp-med-Lingual	Destrieux Atlas	Forearm BMD	Wald ratio	1	0.82	0.50	1.35	4.44E-01
1199	27424	aparc-a2009s_lh_thickness_G-oc-temp-med-Lingual	Destrieux Atlas	Heel BMD	Wald ratio	1	1.16	1.09	1.24	4.36E-06
1199	27424	aparc-a2009s_lh_thickness_G-oc-temp-med-Lingual	Destrieux Atlas	Total body BMD	Wald ratio	1	0.88	0.72	1.09	2.53E-01
1210	27435	aparc-a2009s_lh_thickness_G-temp-sup-G-T-transv	Destrieux Atlas	Lumbar spine BMD	Wald ratio	1	0.79	0.59	1.06	1.25E-01
1210	27435	aparc-a2009s_lh_thickness_G-temp-sup-G-T-transv	Destrieux Atlas	Femoral neck BMD	Wald ratio	1	0.85	0.66	1.09	1.98E-01
1210	27435	aparc-a2009s_lh_thickness_G-temp-sup-G-T-transv	Destrieux Atlas	Forearm BMD	Wald ratio	1	0.82	0.49	1.36	4.44E-01
1210	27435	aparc-a2009s_lh_thickness_G-temp-sup-G-T-transv	Destrieux Atlas	Heel BMD	Wald ratio	1	1.16	1.09	1.24	4.36E-06
1210	27435	aparc-a2009s_lh_thickness_G-temp-sup-G-T-transv	Destrieux Atlas	Total body BMD	IVW	2	0.75	0.50	1.12	1.59E-01
1221	27446	aparc-a2009s_lh_thickness_S-calcarine	Destrieux Atlas	Lumbar spine BMD	Wald ratio	1	0.75	0.52	1.09	1.25E-01
1221	27446	aparc-a2009s_lh_thickness_S-calcarine	Destrieux Atlas	Femoral neck BMD	Wald ratio	1	0.81	0.60	1.11	1.98E-01
1221	27446	aparc-a2009s_lh_thickness_S-calcarine	Destrieux Atlas	Forearm BMD	Wald ratio	1	0.78	0.41	1.47	4.44E-01
1221	27446	aparc-a2009s_lh_thickness_S-calcarine	Destrieux Atlas	Heel BMD	Wald ratio	1	1.20	1.11	1.30	4.36E-06
1221	27446	aparc-a2009s_lh_thickness_S-calcarine	Destrieux Atlas	Total body BMD	Wald ratio	1	0.85	0.65	1.12	2.53E-01
1254	27627	aparc-a2009s_rh_thickness_G+S-paracentral	Destrieux Atlas	Lumbar spine BMD	Wald ratio	1	0.83	0.65	1.05	1.25E-01
1254	27627	aparc-a2009s_rh_thickness_G+S-paracentral	Destrieux Atlas	Femoral neck BMD	Wald ratio	1	0.87	0.71	1.07	1.98E-01
1254	27627	aparc-a2009s_rh_thickness_G+S-paracentral	Destrieux Atlas	Forearm BMD	Wald ratio	1	0.85	0.56	1.28	4.44E-01
1254	27627	aparc-a2009s_rh_thickness_G+S-paracentral	Destrieux Atlas	Heel BMD	Wald ratio	1	1.14	1.08	1.20	4.36E-06

1254	27627	aparc-a2009s_rh_thickness_G+S-paracentral	Destrieux Atlas	Total body BMD	IVW	2	0.93	0.78	1.09	3.86E-01
1293	27666	aparc-a2009s_rh_thickness_Pole-occipital	Destrieux Atlas	Lumbar spine BMD	Wald ratio	1	0.87	0.73	1.04	1.25E-01
1293	27666	aparc-a2009s_rh_thickness_Pole-occipital	Destrieux Atlas	Femoral neck BMD	Wald ratio	1	0.91	0.78	1.06	1.98E-01
1293	27666	aparc-a2009s_rh_thickness_Pole-occipital	Destrieux Atlas	Forearm BMD	Wald ratio	1	0.88	0.65	1.20	4.44E-01
1293	27666	aparc-a2009s_rh_thickness_Pole-occipital	Destrieux Atlas	Heel BMD	Wald ratio	1	1.10	1.06	1.14	4.36E-06
1293	27666	aparc-a2009s_rh_thickness_Pole-occipital	Destrieux Atlas	Total body BMD	Wald ratio	1	0.93	0.82	1.05	2.53E-01
227	26604	AmygNuclei_lh_volume_Central-nucleus	Amygdala Nuclei	Lumbar spine BMD	IVW	2	1.11	0.98	1.26	1.05E-01
227	26604	AmygNuclei_lh_volume_Central-nucleus	Amygdala Nuclei	Femoral neck BMD	IVW	2	1.05	0.91	1.22	4.92E-01
227	26604	AmygNuclei_lh_volume_Central-nucleus	Amygdala Nuclei	Forearm BMD	IVW	2	1.00	0.60	1.65	9.85E-01
227	26604	AmygNuclei_lh_volume_Central-nucleus	Amygdala Nuclei	Heel BMD	IVW	2	0.93	0.91	0.96	2.05E-06
227	26604	AmygNuclei_lh_volume_Central-nucleus	Amygdala Nuclei	Total body BMD	IVW	2	1.06	0.96	1.18	2.07E-01
312	26689	ThalamNuclei_rh_volume_MGN	Thalamus Nuclei	Lumbar spine BMD	IVW	2	0.90	0.72	1.13	3.66E-01
312	26689	ThalamNuclei_rh_volume_MGN	Thalamus Nuclei	Femoral neck BMD	IVW	2	0.95	0.74	1.22	6.89E-01
312	26689	ThalamNuclei_rh_volume_MGN	Thalamus Nuclei	Forearm BMD	IVW	2	0.85	0.61	1.19	3.53E-01
312	26689	ThalamNuclei_rh_volume_MGN	Thalamus Nuclei	Heel BMD	IVW	2	1.11	1.07	1.15	9.70E-07
312	26689	ThalamNuclei_rh_volume_MGN	Thalamus Nuclei	Total body BMD	IVW	2	0.97	0.79	1.19	7.79E-01
576	27701	aparc-a2009s_rh_volume_G+S-paracentral	Destrieux Atlas	Lumbar spine BMD	Wald ratio	1	0.75	0.52	1.08	1.25E-01
576	27701	aparc-a2009s_rh_volume_G+S-paracentral	Destrieux Atlas	Femoral neck BMD	Wald ratio	1	0.81	0.60	1.11	1.98E-01
576	27701	aparc-a2009s_rh_volume_G+S-paracentral	Destrieux Atlas	Forearm BMD	Wald ratio	1	0.78	0.42	1.46	4.44E-01
576	27701	aparc-a2009s_rh_volume_G+S-paracentral	Destrieux Atlas	Heel BMD	Wald ratio	1	1.20	1.11	1.30	4.36E-06
576	27701	aparc-a2009s_rh_volume_G+S-paracentral	Destrieux Atlas	Total body BMD	Wald ratio	1	0.85	0.66	1.11	2.53E-01
1028	26763	aparc-Desikan_lh_thickness_inferiortemporal	Desikan Atlas	Lumbar spine BMD	IVW	4	0.65	0.54	0.77	1.61E-06
1028	26763	aparc-Desikan_lh_thickness_inferiortemporal	Desikan Atlas	Lumbar spine BMD	MR Egger	4	0.46	0.23	0.93	1.64E-01
1028	26763	aparc-Desikan_lh_thickness_inferiortemporal	Desikan Atlas	Lumbar spine BMD	Simple mode	4	0.60	0.42	0.85	6.73E-02
1028	26763	aparc-Desikan_lh_thickness_inferiortemporal	Desikan Atlas	Lumbar spine BMD	Weighted median	4	0.63	0.50	0.79	4.71E-05
1028	26763	aparc-Desikan_lh_thickness_inferiortemporal	Desikan Atlas	Lumbar spine BMD	Weighted mode	4	0.58	0.41	0.83	5.90E-02
1028	26763	aparc-Desikan_lh_thickness_inferiortemporal	Desikan Atlas	Femoral neck BMD	IVW	4	0.79	0.62	1.01	5.92E-02
1028	26763	aparc-Desikan_lh_thickness_inferiortemporal	Desikan Atlas	Femoral neck BMD	MR Egger	4	0.61	0.20	1.82	4.69E-01
1028	26763	aparc-Desikan_lh_thickness_inferiortemporal	Desikan Atlas	Femoral neck BMD	Simple mode	4	0.71	0.56	0.90	6.82E-02
1028	26763	aparc-Desikan_lh_thickness_inferiortemporal	Desikan Atlas	Femoral neck BMD	Weighted median	4	0.74	0.60	0.90	3.07E-03
1028	26763	aparc-Desikan_lh_thickness_inferiortemporal	Desikan Atlas	Femoral neck BMD	Weighted mode	4	0.71	0.56	0.90	6.95E-02
1028	26763	aparc-Desikan_lh_thickness_inferiortemporal	Desikan Atlas	Forearm BMD	IVW	4	0.58	0.41	0.83	2.27E-03
1028	26763	aparc-Desikan_lh_thickness_inferiortemporal	Desikan Atlas	Forearm BMD	MR Egger	4	0.42	0.08	2.28	4.23E-01

1028	26763	aparc-Desikan_lh_thickness_inferiortemporal	Desikan Atlas	Forearm BMD	Simple mode	4	0.50	0.27	0.90	1.06E-01
1028	26763	aparc-Desikan_lh_thickness_inferiortemporal	Desikan Atlas	Forearm BMD	Weighted median	4	0.52	0.35	0.79	2.31E-03
1028	26763	aparc-Desikan_lh_thickness_inferiortemporal	Desikan Atlas	Forearm BMD	Weighted mode	4	0.49	0.26	0.94	1.19E-01
1028	26763	aparc-Desikan_lh_thickness_inferiortemporal	Desikan Atlas	Heel BMD	IVW	4	0.85	0.75	0.97	1.48E-02
1028	26763	aparc-Desikan_lh_thickness_inferiortemporal	Desikan Atlas	Heel BMD	MR Egger	4	0.70	0.42	1.18	3.14E-01
1028	26763	aparc-Desikan_lh_thickness_inferiortemporal	Desikan Atlas	Heel BMD	Simple mode	4	0.97	0.80	1.18	7.86E-01
1028	26763	aparc-Desikan_lh_thickness_inferiortemporal	Desikan Atlas	Heel BMD	Weighted median	4	0.90	0.85	0.95	4.10E-04
1028	26763	aparc-Desikan_lh_thickness_inferiortemporal	Desikan Atlas	Heel BMD	Weighted mode	4	0.98	0.92	1.04	5.69E-01
1028	26763	aparc-Desikan_lh_thickness_inferiortemporal	Desikan Atlas	Total body BMD	IVW	4	0.86	0.77	0.97	1.47E-02
1028	26763	aparc-Desikan_lh_thickness_inferiortemporal	Desikan Atlas	Total body BMD	MR Egger	4	0.67	0.43	1.04	2.17E-01
1028	26763	aparc-Desikan_lh_thickness_inferiortemporal	Desikan Atlas	Total body BMD	Simple mode	4	0.83	0.66	1.04	2.04E-01
1028	26763	aparc-Desikan_lh_thickness_inferiortemporal	Desikan Atlas	Total body BMD	Weighted median	4	0.85	0.73	0.98	2.87E-02
1028	26763	aparc-Desikan_lh_thickness_inferiortemporal	Desikan Atlas	Total body BMD	Weighted mode	4	0.81	0.66	1.00	1.43E-01
378	26891	aparc-Desikan_rh_volume_caudalanteriorcingulate	Desikan Atlas	Lumbar spine BMD	IVW	2	0.68	0.51	0.91	8.52E-03
378	26891	aparc-Desikan_rh_volume_caudalanteriorcingulate	Desikan Atlas	Femoral neck BMD	IVW	2	0.80	0.63	1.02	7.32E-02
378	26891	aparc-Desikan_rh_volume_caudalanteriorcingulate	Desikan Atlas	Forearm BMD	IVW	2	0.72	0.44	1.18	1.94E-01
378	26891	aparc-Desikan_rh_volume_caudalanteriorcingulate	Desikan Atlas	Heel BMD	IVW	2	0.70	0.60	0.82	4.91E-06
378	26891	aparc-Desikan_rh_volume_caudalanteriorcingulate	Desikan Atlas	Total body BMD	IVW	2	0.73	0.55	0.97	2.96E-02
305	26682	ThalamNuclei_lh_volume_PuL	Thalamus Nuclei	Lumbar spine BMD	IVW	3	0.89	0.72	1.11	3.19E-01
305	26682	ThalamNuclei_lh_volume_PuL	Thalamus Nuclei	Lumbar spine BMD	MR Egger	3	1.69	0.03	101.30	8.45E-01
305	26682	ThalamNuclei_lh_volume_PuL	Thalamus Nuclei	Lumbar spine BMD	Simple mode	3	0.95	0.70	1.30	7.91E-01
305	26682	ThalamNuclei_lh_volume_PuL	Thalamus Nuclei	Lumbar spine BMD	Weighted median	3	0.93	0.72	1.22	6.25E-01
305	26682	ThalamNuclei_lh_volume_PuL	Thalamus Nuclei	Lumbar spine BMD	Weighted mode	3	0.95	0.71	1.29	7.85E-01
305	26682	ThalamNuclei_lh_volume_PuL	Thalamus Nuclei	Femoral neck BMD	IVW	3	0.99	0.82	1.20	9.56E-01
305	26682	ThalamNuclei_lh_volume_PuL	Thalamus Nuclei	Femoral neck BMD	MR Egger	3	0.94	0.03	32.48	9.79E-01
305	26682	ThalamNuclei_lh_volume_PuL	Thalamus Nuclei	Femoral neck BMD	Simple mode	3	1.03	0.81	1.32	8.26E-01
305	26682	ThalamNuclei_lh_volume_PuL	Thalamus Nuclei	Femoral neck BMD	Weighted median	3	1.01	0.80	1.27	9.41E-01
305	26682	ThalamNuclei_lh_volume_PuL	Thalamus Nuclei	Femoral neck BMD	Weighted mode	3	1.03	0.80	1.34	8.44E-01
305	26682	ThalamNuclei_lh_volume_PuL	Thalamus Nuclei	Forearm BMD	IVW	3	0.93	0.63	1.37	7.11E-01
305	26682	ThalamNuclei_lh_volume_PuL	Thalamus Nuclei	Forearm BMD	MR Egger	3	4.00	0.00	5881.12	7.72E-01
305	26682	ThalamNuclei_lh_volume_PuL	Thalamus Nuclei	Forearm BMD	Simple mode	3	0.88	0.53	1.45	6.58E-01
305	26682	ThalamNuclei_lh_volume_PuL	Thalamus Nuclei	Forearm BMD	Weighted median	3	0.89	0.56	1.42	6.47E-01
305	26682	ThalamNuclei_lh_volume_PuL	Thalamus Nuclei	Forearm BMD	Weighted mode	3	0.88	0.52	1.49	6.69E-01

305	26682	ThalamNuclei_lh_volume_PuL	Thalamus Nuclei	Heel BMD	Wald ratio	1	0.85	0.79	0.91	2.31E-05
305	26682	ThalamNuclei_lh_volume_PuL	Thalamus Nuclei	Total body BMD	IVW	2	0.91	0.72	1.15	4.13E-01
311	26688	ThalamNuclei_rh_volume_LGN	Thalamus Nuclei	Forearm BMD	Wald ratio	1	0.76	0.40	1.47	4.19E-01
311	26688	ThalamNuclei_rh_volume_LGN	Thalamus Nuclei	Heel BMD	Wald ratio	1	0.85	0.79	0.91	1.54E-05
311	26688	ThalamNuclei_rh_volume_LGN	Thalamus Nuclei	Total body BMD	IVW	2	1.02	0.85	1.23	8.30E-01
1032	26767	aparc-Desikan_lh_thickness_lingual	Desikan Atlas	Lumbar spine BMD	IVW	4	0.89	0.63	1.27	5.31E-01
1032	26767	aparc-Desikan_lh_thickness_lingual	Desikan Atlas	Lumbar spine BMD	MR Egger	4	0.57	0.16	2.10	4.88E-01
1032	26767	aparc-Desikan_lh_thickness_lingual	Desikan Atlas	Lumbar spine BMD	Simple mode	4	0.79	0.60	1.04	1.83E-01
1032	26767	aparc-Desikan_lh_thickness_lingual	Desikan Atlas	Lumbar spine BMD	Weighted median	4	0.82	0.65	1.04	1.06E-01
1032	26767	aparc-Desikan_lh_thickness_lingual	Desikan Atlas	Lumbar spine BMD	Weighted mode	4	0.79	0.60	1.03	1.82E-01
1032	26767	aparc-Desikan_lh_thickness_lingual	Desikan Atlas	Femoral neck BMD	IVW	4	0.85	0.68	1.08	1.85E-01
1032	26767	aparc-Desikan_lh_thickness_lingual	Desikan Atlas	Femoral neck BMD	MR Egger	4	0.76	0.30	1.93	6.19E-01
1032	26767	aparc-Desikan_lh_thickness_lingual	Desikan Atlas	Femoral neck BMD	Simple mode	4	0.75	0.57	0.98	1.32E-01
1032	26767	aparc-Desikan_lh_thickness_lingual	Desikan Atlas	Femoral neck BMD	Weighted median	4	0.80	0.66	0.97	2.60E-02
1032	26767	aparc-Desikan_lh_thickness_lingual	Desikan Atlas	Femoral neck BMD	Weighted mode	4	0.78	0.61	1.00	1.42E-01
1032	26767	aparc-Desikan_lh_thickness_lingual	Desikan Atlas	Forearm BMD	IVW	4	0.88	0.64	1.22	4.48E-01
1032	26767	aparc-Desikan_lh_thickness_lingual	Desikan Atlas	Forearm BMD	MR Egger	4	0.69	0.24	1.99	5.64E-01
1032	26767	aparc-Desikan_lh_thickness_lingual	Desikan Atlas	Forearm BMD	Simple mode	4	0.83	0.51	1.37	5.28E-01
1032	26767	aparc-Desikan_lh_thickness_lingual	Desikan Atlas	Forearm BMD	Weighted median	4	0.83	0.57	1.21	3.52E-01
1032	26767	aparc-Desikan_lh_thickness_lingual	Desikan Atlas	Forearm BMD	Weighted mode	4	0.83	0.53	1.28	4.63E-01
1032	26767	aparc-Desikan_lh_thickness_lingual	Desikan Atlas	Heel BMD	IVW	4	1.11	1.06	1.17	1.98E-05
1032	26767	aparc-Desikan_lh_thickness_lingual	Desikan Atlas	Heel BMD	MR Egger	4	1.27	1.11	1.44	7.54E-02
1032	26767	aparc-Desikan_lh_thickness_lingual	Desikan Atlas	Heel BMD	Simple mode	4	1.08	0.99	1.17	1.91E-01
1032	26767	aparc-Desikan_lh_thickness_lingual	Desikan Atlas	Heel BMD	Weighted median	4	1.09	1.03	1.14	6.82E-04
1032	26767	aparc-Desikan_lh_thickness_lingual	Desikan Atlas	Heel BMD	Weighted mode	4	1.08	0.99	1.17	2.02E-01
1032	26767	aparc-Desikan_lh_thickness_lingual	Desikan Atlas	Total body BMD	IVW	4	0.97	0.86	1.10	6.66E-01
1032	26767	aparc-Desikan_lh_thickness_lingual	Desikan Atlas	Total body BMD	MR Egger	4	0.74	0.48	1.15	3.10E-01
1032	26767	aparc-Desikan_lh_thickness_lingual	Desikan Atlas	Total body BMD	Simple mode	4	0.94	0.76	1.16	6.08E-01
1032	26767	aparc-Desikan_lh_thickness_lingual	Desikan Atlas	Total body BMD	Weighted median	4	0.95	0.82	1.10	4.72E-01
1032	26767	aparc-Desikan_lh_thickness_lingual	Desikan Atlas	Total body BMD	Weighted mode	4	0.93	0.77	1.13	5.63E-01
1017	27622	aparc-a2009s_rh_area_S-temporal-inf	Destrieux Atlas	Lumbar spine BMD	Wald ratio	1	0.96	0.65	1.42	8.64E-01
1017	27622	aparc-a2009s_rh_area_S-temporal-inf	Destrieux Atlas	Femoral neck BMD	Wald ratio	1	1.03	0.73	1.45	8.49E-01
1017	27622	aparc-a2009s_rh_area_S-temporal-inf	Destrieux Atlas	Forearm BMD	Wald ratio	1	1.14	0.56	2.32	7.25E-01

1017	27622	aparc-a2009s_rh_area_S-temporal-inf	Destrieux Atlas	Heel BMD	Wald ratio	1	0.83	0.77	0.91	1.19E-05
1017	27622	aparc-a2009s_rh_area_S-temporal-inf	Destrieux Atlas	Total body BMD	Wald ratio	1	0.90	0.70	1.17	4.30E-01
1186	27411	aparc-a2009s_lh_thickness_G-cingul-Post-dorsal	Destrieux Atlas	Lumbar spine BMD	Wald ratio	1	0.75	0.52	1.08	1.25E-01
1186	27411	aparc-a2009s_lh_thickness_G-cingul-Post-dorsal	Destrieux Atlas	Femoral neck BMD	Wald ratio	1	0.81	0.60	1.11	1.98E-01
1186	27411	aparc-a2009s_lh_thickness_G-cingul-Post-dorsal	Destrieux Atlas	Forearm BMD	Wald ratio	1	0.78	0.42	1.47	4.44E-01
1186	27411	aparc-a2009s_lh_thickness_G-cingul-Post-dorsal	Destrieux Atlas	Heel BMD	Wald ratio	1	1.16	1.08	1.25	8.95E-05
1186	27411	aparc-a2009s_lh_thickness_G-cingul-Post-dorsal	Destrieux Atlas	Total body BMD	IVW	2	0.85	0.67	1.08	1.87E-01
531	27508	aparc-a2009s_lh_volume_G-subcallosal	Destrieux Atlas	Lumbar spine BMD	Wald ratio	1	1.14	0.77	1.67	5.18E-01
531	27508	aparc-a2009s_lh_volume_G-subcallosal	Destrieux Atlas	Femoral neck BMD	Wald ratio	1	1.04	0.75	1.45	8.09E-01
531	27508	aparc-a2009s_lh_volume_G-subcallosal	Destrieux Atlas	Forearm BMD	Wald ratio	1	1.16	0.58	2.32	6.60E-01
531	27508	aparc-a2009s_lh_volume_G-subcallosal	Destrieux Atlas	Heel BMD	Wald ratio	1	1.18	1.08	1.28	6.79E-05
531	27508	aparc-a2009s_lh_volume_G-subcallosal	Destrieux Atlas	Total body BMD	Wald ratio	1	1.28	1.00	1.65	5.49E-02
679	26752	aparc-Desikan_lh_area_frontalpole	Desikan Atlas	Lumbar spine BMD	Wald ratio	1	1.09	0.82	1.45	5.43E-01
679	26752	aparc-Desikan_lh_area_frontalpole	Desikan Atlas	Femoral neck BMD	Wald ratio	1	0.93	0.73	1.19	6.02E-01
679	26752	aparc-Desikan_lh_area_frontalpole	Desikan Atlas	Forearm BMD	Wald ratio	1	1.06	0.63	1.79	8.16E-01
679	26752	aparc-Desikan_lh_area_frontalpole	Desikan Atlas	Heel BMD	Wald ratio	1	0.88	0.84	0.94	5.34E-05
679	26752	aparc-Desikan_lh_area_frontalpole	Desikan Atlas	Total body BMD	Wald ratio	1	1.15	0.96	1.38	1.59E-01
590	27715	aparc-a2009s_rh_volume_G-Ins-lg+S-cent-ins	Destrieux Atlas	Heel BMD	Wald ratio	1	0.86	0.80	0.93	9.87E-05
590	27715	aparc-a2009s_rh_volume_G-Ins-lg+S-cent-ins	Destrieux Atlas	Total body BMD	Wald ratio	1	0.76	0.58	0.99	3.83E-02
55	25811	IDP_T1_FAST_ROIs_R_inf_temp_gyrus_post	IDP T1:unilateral regions	Lumbar spine BMD	IVW	2	1.02	0.78	1.33	8.66E-01
55	25811	IDP_T1_FAST_ROIs_R_inf_temp_gyrus_post	IDP T1:unilateral regions	Femoral neck BMD	IVW	2	0.96	0.77	1.21	7.06E-01
55	25811	IDP_T1_FAST_ROIs_R_inf_temp_gyrus_post	IDP T1:unilateral regions	Forearm BMD	IVW	2	0.73	0.46	1.16	1.89E-01
55	25811	IDP_T1_FAST_ROIs_R_inf_temp_gyrus_post	IDP T1:unilateral regions	Heel BMD	IVW	2	1.19	1.10	1.29	6.88E-05
55	25811	IDP_T1_FAST_ROIs_R_inf_temp_gyrus_post	IDP T1:unilateral regions	Total body BMD	IVW	2	1.19	1.01	1.41	4.28E-02
47	25803	IDP_T1_FAST_ROIs_R_mid_temp_gyrus_ant	IDP T1:unilateral regions	Lumbar spine BMD	Wald ratio	1	0.84	0.55	1.28	4.06E-01
47	25803	IDP_T1_FAST_ROIs_R_mid_temp_gyrus_ant	IDP T1:unilateral regions	Femoral neck BMD	Wald ratio	1	1.00	0.70	1.43	9.85E-01

47	25803	IDP_T1_FAST_ROIs_R_mid_temp_gyrus_ant	IDP T1:unilateral regions	Forearm BMD	Wald ratio	1	0.70	0.34	1.47	3.49E-01
47	25803	IDP_T1_FAST_ROIs_R_mid_temp_gyrus_ant	IDP T1:unilateral regions	Heel BMD	Wald ratio	1	1.18	1.08	1.28	1.37E-04
47	25803	IDP_T1_FAST_ROIs_R_mid_temp_gyrus_ant	IDP T1:unilateral regions	Total body BMD	Wald ratio	1	1.00	0.75	1.34	9.88E-01
52	25808	IDP_T1_FAST_ROIs_L_inf_temp_gyrus_ant	IDP T1:unilateral regions	Lumbar spine BMD	Wald ratio	1	0.84	0.56	1.26	4.06E-01
52	25808	IDP_T1_FAST_ROIs_L_inf_temp_gyrus_ant	IDP T1:unilateral regions	Femoral neck BMD	Wald ratio	1	1.00	0.71	1.40	9.85E-01
52	25808	IDP_T1_FAST_ROIs_L_inf_temp_gyrus_ant	IDP T1:unilateral regions	Forearm BMD	Wald ratio	1	0.71	0.35	1.45	3.49E-01
52	25808	IDP_T1_FAST_ROIs_L_inf_temp_gyrus_ant	IDP T1:unilateral regions	Heel BMD	Wald ratio	1	1.16	1.07	1.26	1.37E-04
52	25808	IDP_T1_FAST_ROIs_L_inf_temp_gyrus_ant	IDP T1:unilateral regions	Total body BMD	Wald ratio	1	1.00	0.76	1.32	9.88E-01
82	25838	IDP_T1_FAST_ROIs_L_cing_gyrus_ant	IDP T1:unilateral regions	Heel BMD	Wald ratio	1	1.19	1.10	1.29	9.87E-05
82	25838	IDP_T1_FAST_ROIs_L_cing_gyrus_ant	IDP T1:unilateral regions	Total body BMD	Wald ratio	1	1.37	1.03	1.83	3.83E-02
83	25839	IDP_T1_FAST_ROIs_R_cing_gyrus_ant	IDP T1:unilateral regions	Lumbar spine BMD	IVW	2	1.20	0.64	2.28	5.72E-01
83	25839	IDP_T1_FAST_ROIs_R_cing_gyrus_ant	IDP T1:unilateral regions	Femoral neck BMD	IVW	2	1.39	0.78	2.48	2.63E-01
83	25839	IDP_T1_FAST_ROIs_R_cing_gyrus_ant	IDP T1:unilateral regions	Forearm BMD	IVW	2	1.10	0.49	2.48	8.10E-01
83	25839	IDP_T1_FAST_ROIs_R_cing_gyrus_ant	IDP T1:unilateral regions	Heel BMD	IVW	2	1.14	1.06	1.22	1.23E-04
83	25839	IDP_T1_FAST_ROIs_R_cing_gyrus_ant	IDP T1:unilateral regions	Total body BMD	IVW	2	1.25	0.86	1.82	2.51E-01
172	26521	aseg_global_volume_EstimatedTotalIntraCranial	aseg:global	Lumbar spine BMD	IVW	7	1.45	1.12	1.88	5.10E-03
172	26521	aseg_global_volume_EstimatedTotalIntraCranial	aseg:global	Lumbar spine BMD	MR Egger	7	2.44	1.42	4.20	2.33E-02
172	26521	aseg_global_volume_EstimatedTotalIntraCranial	aseg:global	Lumbar spine BMD	Simple mode	7	1.56	1.10	2.21	4.19E-02

172	26521	aseg_global_volume_EstimatedTotalIntraCranial	aseg:global	Lumbar spine BMD	Weighted median	7	1.61	1.35	1.92	2.52E-07
172	26521	aseg_global_volume_EstimatedTotalIntraCranial	aseg:global	Lumbar spine BMD	Weighted mode	7	1.72	1.44	2.07	1.02E-03
172	26521	aseg_global_volume_EstimatedTotalIntraCranial	aseg:global	Femoral neck BMD	IVW	7	1.43	1.18	1.73	1.92E-04
172	26521	aseg_global_volume_EstimatedTotalIntraCranial	aseg:global	Femoral neck BMD	MR Egger	7	1.67	1.01	2.75	9.48E-02
172	26521	aseg_global_volume_EstimatedTotalIntraCranial	aseg:global	Femoral neck BMD	Simple mode	7	1.11	0.85	1.46	4.55E-01
172	26521	aseg_global_volume_EstimatedTotalIntraCranial	aseg:global	Femoral neck BMD	Weighted median	7	1.47	1.24	1.74	7.38E-06
172	26521	aseg_global_volume_EstimatedTotalIntraCranial	aseg:global	Femoral neck BMD	Weighted mode	7	1.56	1.30	1.88	3.13E-03
172	26521	aseg_global_volume_EstimatedTotalIntraCranial	aseg:global	Forearm BMD	IVW	7	2.27	1.28	4.04	5.95E-03
172	26521	aseg_global_volume_EstimatedTotalIntraCranial	aseg:global	Forearm BMD	MR Egger	7	4.76	1.12	20.31	9.17E-02
172	26521	aseg_global_volume_EstimatedTotalIntraCranial	aseg:global	Forearm BMD	Simple mode	7	1.22	0.62	2.39	5.92E-01
172	26521	aseg_global_volume_EstimatedTotalIntraCranial	aseg:global	Forearm BMD	Weighted median	7	1.92	1.15	3.20	1.12E-02
172	26521	aseg_global_volume_EstimatedTotalIntraCranial	aseg:global	Forearm BMD	Weighted mode	7	3.85	2.12	6.97	3.81E-03
172	26521	aseg_global_volume_EstimatedTotalIntraCranial	aseg:global	Heel BMD	IVW	6	2.33	1.29	4.19	5.47E-03
172	26521	aseg_global_volume_EstimatedTotalIntraCranial	aseg:global	Heel BMD	MR Egger	6	9.09	3.77	21.89	1.02E-02
172	26521	aseg_global_volume_EstimatedTotalIntraCranial	aseg:global	Heel BMD	Simple mode	6	1.08	1.02	1.13	3.84E-02
172	26521	aseg_global_volume_EstimatedTotalIntraCranial	aseg:global	Heel BMD	Weighted median	6	1.10	1.04	1.16	3.08E-04
172	26521	aseg_global_volume_EstimatedTotalIntraCranial	aseg:global	Heel BMD	Weighted mode	6	1.09	1.04	1.14	2.19E-02
172	26521	aseg_global_volume_EstimatedTotalIntraCranial	aseg:global	Total body BMD	IVW	7	1.82	1.17	2.83	7.85E-03
172	26521	aseg_global_volume_EstimatedTotalIntraCranial	aseg:global	Total body BMD	MR Egger	7	5.00	2.29	10.93	9.50E-03
172	26521	aseg_global_volume_EstimatedTotalIntraCranial	aseg:global	Total body BMD	Simple mode	7	1.56	0.85	2.86	1.97E-01
172	26521	aseg_global_volume_EstimatedTotalIntraCranial	aseg:global	Total body BMD	Weighted median	7	1.27	1.04	1.54	1.74E-02
172	26521	aseg_global_volume_EstimatedTotalIntraCranial	aseg:global	Total body BMD	Weighted mode	7	0.93	0.78	1.11	4.51E-01
478	27307	aparc-DKTatlas_rh_volume_lateralorbitofrontal	Desikan Atlas	Lumbar spine BMD	IVW	6	0.99	0.81	1.22	9.32E-01
478	27307	aparc-DKTatlas_rh_volume_lateralorbitofrontal	Desikan Atlas	Lumbar spine BMD	MR Egger	6	7.14	1.00	51.26	1.25E-01
478	27307	aparc-DKTatlas_rh_volume_lateralorbitofrontal	Desikan Atlas	Lumbar spine BMD	Simple mode	6	0.83	0.63	1.08	2.30E-01
478	27307	aparc-DKTatlas_rh_volume_lateralorbitofrontal	Desikan Atlas	Lumbar spine BMD	Weighted median	6	0.89	0.74	1.08	2.19E-01
478	27307	aparc-DKTatlas_rh_volume_lateralorbitofrontal	Desikan Atlas	Lumbar spine BMD	Weighted mode	6	0.83	0.60	1.14	2.93E-01
478	27307	aparc-DKTatlas_rh_volume_lateralorbitofrontal	Desikan Atlas	Femoral neck BMD	IVW	6	1.14	0.94	1.37	1.80E-01
478	27307	aparc-DKTatlas_rh_volume_lateralorbitofrontal	Desikan Atlas	Femoral neck BMD	MR Egger	6	0.72	0.06	8.39	8.06E-01
478	27307	aparc-DKTatlas_rh_volume_lateralorbitofrontal	Desikan Atlas	Femoral neck BMD	Simple mode	6	1.03	0.83	1.28	8.05E-01
478	27307	aparc-DKTatlas_rh_volume_lateralorbitofrontal	Desikan Atlas	Femoral neck BMD	Weighted median	6	1.06	0.90	1.25	4.69E-01
478	27307	aparc-DKTatlas_rh_volume_lateralorbitofrontal	Desikan Atlas	Femoral neck BMD	Weighted mode	6	1.02	0.83	1.25	8.85E-01
478	27307	aparc-DKTatlas_rh_volume_lateralorbitofrontal	Desikan Atlas	Forearm BMD	IVW	6	1.00	0.78	1.28	9.86E-01

478	27307	aparc-DKTatlas_rh_volume_lateralorbitofrontal	Desikan Atlas	Forearm BMD	MR Egger	6	0.72	0.04	14.39	8.39E-01
478	27307	aparc-DKTatlas_rh_volume_lateralorbitofrontal	Desikan Atlas	Forearm BMD	Simple mode	6	0.88	0.56	1.41	6.26E-01
478	27307	aparc-DKTatlas_rh_volume_lateralorbitofrontal	Desikan Atlas	Forearm BMD	Weighted median	6	0.91	0.68	1.22	5.22E-01
478	27307	aparc-DKTatlas_rh_volume_lateralorbitofrontal	Desikan Atlas	Forearm BMD	Weighted mode	6	0.88	0.57	1.35	5.82E-01
478	27307	aparc-DKTatlas_rh_volume_lateralorbitofrontal	Desikan Atlas	Heel BMD	IVW	7	1.15	1.07	1.23	1.60E-04
478	27307	aparc-DKTatlas_rh_volume_lateralorbitofrontal	Desikan Atlas	Heel BMD	MR Egger	7	1.89	0.72	4.94	2.45E-01
478	27307	aparc-DKTatlas_rh_volume_lateralorbitofrontal	Desikan Atlas	Heel BMD	Simple mode	7	1.16	1.08	1.25	4.50E-03
478	27307	aparc-DKTatlas_rh_volume_lateralorbitofrontal	Desikan Atlas	Heel BMD	Weighted median	7	1.16	1.10	1.22	2.20E-09
478	27307	aparc-DKTatlas_rh_volume_lateralorbitofrontal	Desikan Atlas	Heel BMD	Weighted mode	7	1.16	1.10	1.23	2.82E-03
478	27307	aparc-DKTatlas_rh_volume_lateralorbitofrontal	Desikan Atlas	Total body BMD	IVW	7	0.96	0.78	1.19	7.38E-01
478	27307	aparc-DKTatlas_rh_volume_lateralorbitofrontal	Desikan Atlas	Total body BMD	MR Egger	7	1.01	0.05	19.95	9.93E-01
478	27307	aparc-DKTatlas_rh_volume_lateralorbitofrontal	Desikan Atlas	Total body BMD	Simple mode	7	0.92	0.72	1.17	5.38E-01
478	27307	aparc-DKTatlas_rh_volume_lateralorbitofrontal	Desikan Atlas	Total body BMD	Weighted median	7	0.93	0.82	1.07	3.04E-01
478	27307	aparc-DKTatlas_rh_volume_lateralorbitofrontal	Desikan Atlas	Total body BMD	Weighted mode	7	0.91	0.75	1.09	3.64E-01
492	27321	aparc-DKTatlas_rh_volume_rostralanteriorcingulate	Desikan Atlas	Lumbar spine BMD	Wald ratio	1	0.84	0.56	1.26	4.06E-01
492	27321	aparc-DKTatlas_rh_volume_rostralanteriorcingulate	Desikan Atlas	Femoral neck BMD	Wald ratio	1	1.00	0.70	1.42	9.85E-01
492	27321	aparc-DKTatlas_rh_volume_rostralanteriorcingulate	Desikan Atlas	Forearm BMD	Wald ratio	1	0.71	0.35	1.45	3.49E-01
492	27321	aparc-DKTatlas_rh_volume_rostralanteriorcingulate	Desikan Atlas	Heel BMD	Wald ratio	1	1.18	1.08	1.28	1.37E-04
492	27321	aparc-DKTatlas_rh_volume_rostralanteriorcingulate	Desikan Atlas	Total body BMD	Wald ratio	1	1.00	0.76	1.32	9.88E-01
918	27375	aparc-a2009s_lh_area_S-circular-insula-ant	Destrieux Atlas	Lumbar spine BMD	IVW	3	1.18	0.95	1.46	1.29E-01
918	27375	aparc-a2009s_lh_area_S-circular-insula-ant	Destrieux Atlas	Lumbar spine BMD	MR Egger	3	0.73	0.04	14.51	8.71E-01
918	27375	aparc-a2009s_lh_area_S-circular-insula-ant	Destrieux Atlas	Lumbar spine BMD	Simple mode	3	1.30	0.94	1.80	2.61E-01
918	27375	aparc-a2009s_lh_area_S-circular-insula-ant	Destrieux Atlas	Lumbar spine BMD	Weighted median	3	1.28	0.96	1.72	9.43E-02
918	27375	aparc-a2009s_lh_area_S-circular-insula-ant	Destrieux Atlas	Lumbar spine BMD	Weighted mode	3	1.30	0.93	1.82	2.73E-01
918	27375	aparc-a2009s_lh_area_S-circular-insula-ant	Destrieux Atlas	Femoral neck BMD	IVW	3	1.49	1.24	1.80	2.57E-05
918	27375	aparc-a2009s_lh_area_S-circular-insula-ant	Destrieux Atlas	Femoral neck BMD	MR Egger	3	0.75	0.06	9.89	8.64E-01
918	27375	aparc-a2009s_lh_area_S-circular-insula-ant	Destrieux Atlas	Femoral neck BMD	Simple mode	3	1.61	1.17	2.22	9.54E-02
918	27375	aparc-a2009s_lh_area_S-circular-insula-ant	Destrieux Atlas	Femoral neck BMD	Weighted median	3	1.52	1.19	1.93	6.46E-04
918	27375	aparc-a2009s_lh_area_S-circular-insula-ant	Destrieux Atlas	Femoral neck BMD	Weighted mode	3	1.61	1.19	2.19	9.45E-02
918	27375	aparc-a2009s_lh_area_S-circular-insula-ant	Destrieux Atlas	Forearm BMD	IVW	3	0.90	0.61	1.33	5.91E-01
918	27375	aparc-a2009s_lh_area_S-circular-insula-ant	Destrieux Atlas	Forearm BMD	MR Egger	3	8.33	0.06	1075.69	5.53E-01
918	27375	aparc-a2009s_lh_area_S-circular-insula-ant	Destrieux Atlas	Forearm BMD	Simple mode	3	0.75	0.41	1.35	4.40E-01
918	27375	aparc-a2009s_lh_area_S-circular-insula-ant	Destrieux Atlas	Forearm BMD	Weighted median	3	0.75	0.45	1.23	2.52E-01

918	27375	aparc-a2009s_lh_area_S-circular-insula-ant	Destrieux Atlas	Forearm BMD	Weighted mode	3	0.75	0.41	1.35	4.38E-01
918	27375	aparc-a2009s_lh_area_S-circular-insula-ant	Destrieux Atlas	Heel BMD	IVW	3	1.23	1.05	1.45	9.43E-03
918	27375	aparc-a2009s_lh_area_S-circular-insula-ant	Destrieux Atlas	Heel BMD	MR Egger	3	0.37	0.08	1.76	4.28E-01
918	27375	aparc-a2009s_lh_area_S-circular-insula-ant	Destrieux Atlas	Heel BMD	Simple mode	3	1.33	1.17	1.51	4.85E-02
918	27375	aparc-a2009s_lh_area_S-circular-insula-ant	Destrieux Atlas	Heel BMD	Weighted median	3	1.18	1.10	1.26	3.76E-06
918	27375	aparc-a2009s_lh_area_S-circular-insula-ant	Destrieux Atlas	Heel BMD	Weighted mode	3	1.06	0.97	1.16	3.20E-01
918	27375	aparc-a2009s_lh_area_S-circular-insula-ant	Destrieux Atlas	Total body BMD	IVW	3	1.06	0.78	1.45	6.90E-01
918	27375	aparc-a2009s_lh_area_S-circular-insula-ant	Destrieux Atlas	Total body BMD	MR Egger	3	0.07	0.01	0.43	2.12E-01
918	27375	aparc-a2009s_lh_area_S-circular-insula-ant	Destrieux Atlas	Total body BMD	Simple mode	3	0.91	0.74	1.12	4.65E-01
918	27375	aparc-a2009s_lh_area_S-circular-insula-ant	Destrieux Atlas	Total body BMD	Weighted median	3	0.91	0.74	1.12	3.72E-01
918	27375	aparc-a2009s_lh_area_S-circular-insula-ant	Destrieux Atlas	Total body BMD	Weighted mode	3	0.91	0.73	1.14	4.83E-01
407	26920	aparc-Desikan_rh_volume_frontalpole	Desikan Atlas	Lumbar spine BMD	Wald ratio	1	1.15	0.77	1.72	4.92E-01
407	26920	aparc-Desikan_rh_volume_frontalpole	Desikan Atlas	Femoral neck BMD	Wald ratio	1	1.35	0.96	1.91	7.90E-02
407	26920	aparc-Desikan_rh_volume_frontalpole	Desikan Atlas	Forearm BMD	Wald ratio	1	1.96	0.97	3.98	5.89E-02
407	26920	aparc-Desikan_rh_volume_frontalpole	Desikan Atlas	Heel BMD	Wald ratio	1	0.86	0.79	0.94	2.61E-04
407	26920	aparc-Desikan_rh_volume_frontalpole	Desikan Atlas	Total body BMD	Wald ratio	1	1.19	0.92	1.54	1.87E-01
1312	27685	aparc-a2009s_rh_thickness_S-oc-temp-med+Lingual	Destrieux Atlas	Lumbar spine BMD	IVW	2	1.11	0.80	1.54	5.34E-01
1312	27685	aparc-a2009s_rh_thickness_S-oc-temp-med+Lingual	Destrieux Atlas	Femoral neck BMD	IVW	2	1.18	0.88	1.57	2.81E-01
1312	27685	aparc-a2009s_rh_thickness_S-oc-temp-med+Lingual	Destrieux Atlas	Forearm BMD	IVW	2	0.94	0.51	1.75	8.60E-01
1312	27685	aparc-a2009s_rh_thickness_S-oc-temp-med+Lingual	Destrieux Atlas	Heel BMD	Wald ratio	1	1.15	1.06	1.25	5.72E-04
1312	27685	aparc-a2009s_rh_thickness_S-oc-temp-med+Lingual	Destrieux Atlas	Total body BMD	IVW	2	0.96	0.69	1.35	8.41E-01
1322	27695	aparc-a2009s_rh_thickness_S-subparietal	Destrieux Atlas	Lumbar spine BMD	IVW	4	0.85	0.68	1.05	1.24E-01
1322	27695	aparc-a2009s_rh_thickness_S-subparietal	Destrieux Atlas	Lumbar spine BMD	MR Egger	4	6.67	0.51	87.43	2.68E-01
1322	27695	aparc-a2009s_rh_thickness_S-subparietal	Destrieux Atlas	Lumbar spine BMD	Simple mode	4	0.95	0.68	1.34	7.82E-01
1322	27695	aparc-a2009s_rh_thickness_S-subparietal	Destrieux Atlas	Lumbar spine BMD	Weighted median	4	0.91	0.72	1.15	4.34E-01
1322	27695	aparc-a2009s_rh_thickness_S-subparietal	Destrieux Atlas	Lumbar spine BMD	Weighted mode	4	0.95	0.70	1.29	7.89E-01
1322	27695	aparc-a2009s_rh_thickness_S-subparietal	Destrieux Atlas	Femoral neck BMD	IVW	4	0.88	0.75	1.03	1.13E-01
1322	27695	aparc-a2009s_rh_thickness_S-subparietal	Destrieux Atlas	Femoral neck BMD	MR Egger	4	1.30	0.17	10.14	8.27E-01
1322	27695	aparc-a2009s_rh_thickness_S-subparietal	Destrieux Atlas	Femoral neck BMD	Simple mode	4	0.91	0.71	1.16	4.94E-01
1322	27695	aparc-a2009s_rh_thickness_S-subparietal	Destrieux Atlas	Femoral neck BMD	Weighted median	4	0.90	0.75	1.09	2.66E-01
1322	27695	aparc-a2009s_rh_thickness_S-subparietal	Destrieux Atlas	Femoral neck BMD	Weighted mode	4	0.91	0.71	1.16	4.79E-01
1322	27695	aparc-a2009s_rh_thickness_S-subparietal	Destrieux Atlas	Forearm BMD	IVW	4	0.88	0.63	1.23	4.59E-01
1322	27695	aparc-a2009s_rh_thickness_S-subparietal	Destrieux Atlas	Forearm BMD	MR Egger	4	0.63	0.00	94.75	8.72E-01

1322	27695	aparc-a2009s_rh_thickness_S-subparietal	Destrieux Atlas	Forearm BMD	Simple mode	4	0.93	0.52	1.69	8.43E-01
1322	27695	aparc-a2009s_rh_thickness_S-subparietal	Destrieux Atlas	Forearm BMD	Weighted median	4	0.91	0.60	1.39	6.55E-01
1322	27695	aparc-a2009s_rh_thickness_S-subparietal	Destrieux Atlas	Forearm BMD	Weighted mode	4	0.94	0.52	1.71	8.52E-01
1322	27695	aparc-a2009s_rh_thickness_S-subparietal	Destrieux Atlas	Heel BMD	IVW	4	0.92	0.87	0.96	5.61E-04
1322	27695	aparc-a2009s_rh_thickness_S-subparietal	Destrieux Atlas	Heel BMD	MR Egger	4	0.52	0.31	0.86	1.27E-01
1322	27695	aparc-a2009s_rh_thickness_S-subparietal	Destrieux Atlas	Heel BMD	Simple mode	4	0.94	0.87	1.02	2.58E-01
1322	27695	aparc-a2009s_rh_thickness_S-subparietal	Destrieux Atlas	Heel BMD	Weighted median	4	0.93	0.89	0.98	8.77E-03
1322	27695	aparc-a2009s_rh_thickness_S-subparietal	Destrieux Atlas	Heel BMD	Weighted mode	4	0.94	0.87	1.02	2.58E-01
1322	27695	aparc-a2009s_rh_thickness_S-subparietal	Destrieux Atlas	Total body BMD	IVW	4	1.05	0.91	1.21	4.35E-01
1322	27695	aparc-a2009s_rh_thickness_S-subparietal	Destrieux Atlas	Total body BMD	MR Egger	4	1.72	0.35	8.50	5.81E-01
1322	27695	aparc-a2009s_rh_thickness_S-subparietal	Destrieux Atlas	Total body BMD	Simple mode	4	1.06	0.86	1.32	6.33E-01
1322	27695	aparc-a2009s_rh_thickness_S-subparietal	Destrieux Atlas	Total body BMD	Weighted median	4	1.08	0.91	1.27	3.97E-01
1322	27695	aparc-a2009s_rh_thickness_S-subparietal	Destrieux Atlas	Total body BMD	Weighted mode	4	1.06	0.89	1.27	5.39E-01
1257	27630	aparc-a2009s_rh_thickness_G+S-cingul-Ant	Destrieux Atlas	Lumbar spine BMD	Wald ratio	1	0.79	0.54	1.15	2.17E-01
1257	27630	aparc-a2009s_rh_thickness_G+S-cingul-Ant	Destrieux Atlas	Femoral neck BMD	Wald ratio	1	0.78	0.57	1.05	1.04E-01
1257	27630	aparc-a2009s_rh_thickness_G+S-cingul-Ant	Destrieux Atlas	Forearm BMD	Wald ratio	1	1.11	0.52	2.36	7.72E-01
1257	27630	aparc-a2009s_rh_thickness_G+S-cingul-Ant	Destrieux Atlas	Heel BMD	Wald ratio	1	0.90	0.85	0.96	6.60E-04
1257	27630	aparc-a2009s_rh_thickness_G+S-cingul-Ant	Destrieux Atlas	Total body BMD	Wald ratio	1	0.80	0.64	1.00	5.04E-02
884	27341	aparc-a2009s_lh_area_G-front-inf-Orbital	Destrieux Atlas	Lumbar spine BMD	Wald ratio	1	1.45	1.00	2.09	5.17E-02
884	27341	aparc-a2009s_lh_area_G-front-inf-Orbital	Destrieux Atlas	Femoral neck BMD	Wald ratio	1	1.32	0.95	1.82	9.19E-02
884	27341	aparc-a2009s_lh_area_G-front-inf-Orbital	Destrieux Atlas	Forearm BMD	Wald ratio	1	1.28	0.67	2.47	4.73E-01
884	27341	aparc-a2009s_lh_area_G-front-inf-Orbital	Destrieux Atlas	Heel BMD	Wald ratio	1	1.16	1.08	1.25	1.69E-04
884	27341	aparc-a2009s_lh_area_G-front-inf-Orbital	Destrieux Atlas	Total body BMD	Wald ratio	1	1.39	1.09	1.77	6.73E-03
229	26606	AmygNuclei_lh_volume_Cortical-nucleus	Amygdala Nuclei	Lumbar spine BMD	IVW	3	1.06	0.86	1.32	6.00E-01
229	26606	AmygNuclei_lh_volume_Cortical-nucleus	Amygdala Nuclei	Lumbar spine BMD	MR Egger	3	1.32	0.98	1.76	3.14E-01
229	26606	AmygNuclei_lh_volume_Cortical-nucleus	Amygdala Nuclei	Lumbar spine BMD	Simple mode	3	1.10	0.85	1.43	5.54E-01
229	26606	AmygNuclei_lh_volume_Cortical-nucleus	Amygdala Nuclei	Lumbar spine BMD	Weighted median	3	1.11	0.94	1.32	2.34E-01
229	26606	AmygNuclei_lh_volume_Cortical-nucleus	Amygdala Nuclei	Lumbar spine BMD	Weighted mode	3	1.14	0.95	1.35	2.86E-01
229	26606	AmygNuclei_lh_volume_Cortical-nucleus	Amygdala Nuclei	Femoral neck BMD	IVW	3	1.14	1.00	1.30	5.11E-02
229	26606	AmygNuclei_lh_volume_Cortical-nucleus	Amygdala Nuclei	Femoral neck BMD	MR Egger	3	1.08	0.85	1.37	6.42E-01
229	26606	AmygNuclei_lh_volume_Cortical-nucleus	Amygdala Nuclei	Femoral neck BMD	Simple mode	3	1.19	1.00	1.41	1.94E-01
229	26606	AmygNuclei_lh_volume_Cortical-nucleus	Amygdala Nuclei	Femoral neck BMD	Weighted median	3	1.14	0.99	1.30	6.75E-02
229	26606	AmygNuclei_lh_volume_Cortical-nucleus	Amygdala Nuclei	Femoral neck BMD	Weighted mode	3	1.11	0.95	1.30	3.20E-01

229	26606	AmygNuclei_lh_volume_Cortical-nucleus	Amygdala Nuclei	Forearm BMD	IVW	3	1.18	0.91	1.53	2.37E-01
229	26606	AmygNuclei_lh_volume_Cortical-nucleus	Amygdala Nuclei	Forearm BMD	MR Egger	3	1.08	0.66	1.76	8.19E-01
229	26606	AmygNuclei_lh_volume_Cortical-nucleus	Amygdala Nuclei	Forearm BMD	Simple mode	3	1.14	0.79	1.64	5.65E-01
229	26606	AmygNuclei_lh_volume_Cortical-nucleus	Amygdala Nuclei	Forearm BMD	Weighted median	3	1.14	0.87	1.49	3.56E-01
229	26606	AmygNuclei_lh_volume_Cortical-nucleus	Amygdala Nuclei	Forearm BMD	Weighted mode	3	1.14	0.84	1.54	5.00E-01
229	26606	AmygNuclei_lh_volume_Cortical-nucleus	Amygdala Nuclei	Heel BMD	IVW	3	0.93	0.89	0.97	5.78E-04
229	26606	AmygNuclei_lh_volume_Cortical-nucleus	Amygdala Nuclei	Heel BMD	MR Egger	3	0.89	0.82	0.97	2.24E-01
229	26606	AmygNuclei_lh_volume_Cortical-nucleus	Amygdala Nuclei	Heel BMD	Simple mode	3	0.92	0.86	0.98	1.34E-01
229	26606	AmygNuclei_lh_volume_Cortical-nucleus	Amygdala Nuclei	Heel BMD	Weighted median	3	0.92	0.88	0.95	1.35E-05
229	26606	AmygNuclei_lh_volume_Cortical-nucleus	Amygdala Nuclei	Heel BMD	Weighted mode	3	0.91	0.87	0.95	4.75E-02
229	26606	AmygNuclei_lh_volume_Cortical-nucleus	Amygdala Nuclei	Total body BMD	IVW	3	1.11	1.00	1.23	4.22E-02
229	26606	AmygNuclei_lh_volume_Cortical-nucleus	Amygdala Nuclei	Total body BMD	MR Egger	3	1.04	0.85	1.27	7.73E-01
229	26606	AmygNuclei_lh_volume_Cortical-nucleus	Amygdala Nuclei	Total body BMD	Simple mode	3	1.09	0.92	1.28	3.95E-01
229	26606	AmygNuclei_lh_volume_Cortical-nucleus	Amygdala Nuclei	Total body BMD	Weighted median	3	1.09	0.97	1.22	1.35E-01
229	26606	AmygNuclei_lh_volume_Cortical-nucleus	Amygdala Nuclei	Total body BMD	Weighted mode	3	1.09	0.94	1.25	3.57E-01
1062	26864	aparc-Desikan_rh_thickness_inferiortemporal	Desikan Atlas	Lumbar spine BMD	IVW	7	0.79	0.61	1.01	6.41E-02
1062	26864	aparc-Desikan_rh_thickness_inferiortemporal	Desikan Atlas	Lumbar spine BMD	MR Egger	7	0.45	0.18	1.14	1.52E-01
1062	26864	aparc-Desikan_rh_thickness_inferiortemporal	Desikan Atlas	Lumbar spine BMD	Simple mode	7	0.68	0.52	0.90	3.33E-02
1062	26864	aparc-Desikan_rh_thickness_inferiortemporal	Desikan Atlas	Lumbar spine BMD	Weighted median	7	0.70	0.57	0.88	1.83E-03
1062	26864	aparc-Desikan_rh_thickness_inferiortemporal	Desikan Atlas	Lumbar spine BMD	Weighted mode	7	0.69	0.53	0.90	3.28E-02
1062	26864	aparc-Desikan_rh_thickness_inferiortemporal	Desikan Atlas	Femoral neck BMD	IVW	7	0.90	0.75	1.08	2.75E-01
1062	26864	aparc-Desikan_rh_thickness_inferiortemporal	Desikan Atlas	Femoral neck BMD	MR Egger	7	0.59	0.31	1.12	1.71E-01
1062	26864	aparc-Desikan_rh_thickness_inferiortemporal	Desikan Atlas	Femoral neck BMD	Simple mode	7	0.79	0.63	0.98	7.50E-02
1062	26864	aparc-Desikan_rh_thickness_inferiortemporal	Desikan Atlas	Femoral neck BMD	Weighted median	7	0.81	0.68	0.97	2.51E-02
1062	26864	aparc-Desikan_rh_thickness_inferiortemporal	Desikan Atlas	Femoral neck BMD	Weighted mode	7	0.79	0.62	1.01	1.10E-01
1062	26864	aparc-Desikan_rh_thickness_inferiortemporal	Desikan Atlas	Forearm BMD	IVW	8	0.82	0.65	1.04	1.07E-01
1062	26864	aparc-Desikan_rh_thickness_inferiortemporal	Desikan Atlas	Forearm BMD	MR Egger	8	0.30	0.12	0.77	4.64E-02
1062	26864	aparc-Desikan_rh_thickness_inferiortemporal	Desikan Atlas	Forearm BMD	Simple mode	8	0.71	0.43	1.18	2.28E-01
1062	26864	aparc-Desikan_rh_thickness_inferiortemporal	Desikan Atlas	Forearm BMD	Weighted median	8	0.74	0.54	1.01	5.63E-02
1062	26864	aparc-Desikan_rh_thickness_inferiortemporal	Desikan Atlas	Forearm BMD	Weighted mode	8	0.71	0.45	1.14	1.97E-01
1062	26864	aparc-Desikan_rh_thickness_inferiortemporal	Desikan Atlas	Heel BMD	IVW	6	0.92	0.79	1.07	2.75E-01
1062	26864	aparc-Desikan_rh_thickness_inferiortemporal	Desikan Atlas	Heel BMD	MR Egger	6	0.93	0.49	1.76	8.27E-01
1062	26864	aparc-Desikan_rh_thickness_inferiortemporal	Desikan Atlas	Heel BMD	Simple mode	6	0.96	0.86	1.07	5.51E-01

1062	26864	aparc-Desikan_rh_thickness_inferiortemporal	Desikan Atlas	Heel BMD	Weighted median	6	0.96	0.91	1.01	1.31E-01
1062	26864	aparc-Desikan_rh_thickness_inferiortemporal	Desikan Atlas	Heel BMD	Weighted mode	6	0.97	0.90	1.04	4.40E-01
1062	26864	aparc-Desikan_rh_thickness_inferiortemporal	Desikan Atlas	Total body BMD	IVW	8	0.84	0.77	0.92	2.29E-04
1062	26864	aparc-Desikan_rh_thickness_inferiortemporal	Desikan Atlas	Total body BMD	MR Egger	8	0.84	0.58	1.21	3.77E-01
1062	26864	aparc-Desikan_rh_thickness_inferiortemporal	Desikan Atlas	Total body BMD	Simple mode	8	0.86	0.72	1.03	1.46E-01
1062	26864	aparc-Desikan_rh_thickness_inferiortemporal	Desikan Atlas	Total body BMD	Weighted median	8	0.85	0.76	0.96	7.54E-03
1062	26864	aparc-Desikan_rh_thickness_inferiortemporal	Desikan Atlas	Total body BMD	Weighted mode	8	0.88	0.74	1.04	1.82E-01
1063	26865	aparc-Desikan_rh_thickness_isthmuscingulate	Desikan Atlas	Lumbar spine BMD	IVW	4	1.01	0.70	1.45	9.76E-01
1063	26865	aparc-Desikan_rh_thickness_isthmuscingulate	Desikan Atlas	Lumbar spine BMD	MR Egger	4	4.55	0.99	20.83	1.97E-01
1063	26865	aparc-Desikan_rh_thickness_isthmuscingulate	Desikan Atlas	Lumbar spine BMD	Simple mode	4	0.88	0.67	1.14	4.17E-01
1063	26865	aparc-Desikan_rh_thickness_isthmuscingulate	Desikan Atlas	Lumbar spine BMD	Weighted median	4	0.91	0.72	1.14	3.99E-01
1063	26865	aparc-Desikan_rh_thickness_isthmuscingulate	Desikan Atlas	Lumbar spine BMD	Weighted mode	4	0.89	0.68	1.17	4.49E-01
1063	26865	aparc-Desikan_rh_thickness_isthmuscingulate	Desikan Atlas	Femoral neck BMD	IVW	4	0.95	0.79	1.14	6.22E-01
1063	26865	aparc-Desikan_rh_thickness_isthmuscingulate	Desikan Atlas	Femoral neck BMD	MR Egger	4	0.93	0.26	3.35	9.20E-01
1063	26865	aparc-Desikan_rh_thickness_isthmuscingulate	Desikan Atlas	Femoral neck BMD	Simple mode	4	1.08	0.77	1.49	6.85E-01
1063	26865	aparc-Desikan_rh_thickness_isthmuscingulate	Desikan Atlas	Femoral neck BMD	Weighted median	4	0.98	0.80	1.20	8.20E-01
1063	26865	aparc-Desikan_rh_thickness_isthmuscingulate	Desikan Atlas	Femoral neck BMD	Weighted mode	4	1.08	0.77	1.50	7.07E-01
1063	26865	aparc-Desikan_rh_thickness_isthmuscingulate	Desikan Atlas	Forearm BMD	IVW	4	1.05	0.74	1.49	7.93E-01
1063	26865	aparc-Desikan_rh_thickness_isthmuscingulate	Desikan Atlas	Forearm BMD	MR Egger	4	0.52	0.06	4.20	6.00E-01
1063	26865	aparc-Desikan_rh_thickness_isthmuscingulate	Desikan Atlas	Forearm BMD	Simple mode	4	0.95	0.56	1.63	8.70E-01
1063	26865	aparc-Desikan_rh_thickness_isthmuscingulate	Desikan Atlas	Forearm BMD	Weighted median	4	0.98	0.66	1.45	9.29E-01
1063	26865	aparc-Desikan_rh_thickness_isthmuscingulate	Desikan Atlas	Forearm BMD	Weighted mode	4	0.96	0.57	1.63	8.97E-01
1063	26865	aparc-Desikan_rh_thickness_isthmuscingulate	Desikan Atlas	Heel BMD	IVW	4	1.00	0.96	1.05	9.44E-01
1063	26865	aparc-Desikan_rh_thickness_isthmuscingulate	Desikan Atlas	Heel BMD	MR Egger	4	1.06	0.78	1.45	7.36E-01
1063	26865	aparc-Desikan_rh_thickness_isthmuscingulate	Desikan Atlas	Heel BMD	Simple mode	4	1.01	0.94	1.09	8.74E-01
1063	26865	aparc-Desikan_rh_thickness_isthmuscingulate	Desikan Atlas	Heel BMD	Weighted median	4	1.00	0.95	1.05	9.63E-01
1063	26865	aparc-Desikan_rh_thickness_isthmuscingulate	Desikan Atlas	Heel BMD	Weighted mode	4	1.00	0.92	1.09	9.83E-01
1063	26865	aparc-Desikan_rh_thickness_isthmuscingulate	Desikan Atlas	Total body BMD	IVW	4	1.28	1.13	1.46	1.74E-04
1063	26865	aparc-Desikan_rh_thickness_isthmuscingulate	Desikan Atlas	Total body BMD	MR Egger	4	2.17	1.07	4.42	1.71E-01
1063	26865	aparc-Desikan_rh_thickness_isthmuscingulate	Desikan Atlas	Total body BMD	Simple mode	4	1.32	1.05	1.65	1.07E-01
1063	26865	aparc-Desikan_rh_thickness_isthmuscingulate	Desikan Atlas	Total body BMD	Weighted median	4	1.28	1.09	1.50	2.30E-03
1063	26865	aparc-Desikan_rh_thickness_isthmuscingulate	Desikan Atlas	Total body BMD	Weighted mode	4	1.30	1.03	1.63	1.18E-01
1214	27439	aparc-a2009s_lh_thickness_G-temporal-inf	Destrieux Atlas	Lumbar spine BMD	IVW	5	0.66	0.54	0.81	6.02E-05

1214	27439	aparc-a2009s_lh_thickness_G-temporal-inf	Destrieux Atlas	Lumbar spine BMD	MR Egger	5	0.43	0.16	1.22	2.10E-01
1214	27439	aparc-a2009s_lh_thickness_G-temporal-inf	Destrieux Atlas	Lumbar spine BMD	Simple mode	5	0.73	0.50	1.06	1.76E-01
1214	27439	aparc-a2009s_lh_thickness_G-temporal-inf	Destrieux Atlas	Lumbar spine BMD	Weighted median	5	0.68	0.53	0.88	3.03E-03
1214	27439	aparc-a2009s_lh_thickness_G-temporal-inf	Destrieux Atlas	Lumbar spine BMD	Weighted mode	5	0.75	0.51	1.12	2.25E-01
1214	27439	aparc-a2009s_lh_thickness_G-temporal-inf	Destrieux Atlas	Femoral neck BMD	IVW	5	0.83	0.69	1.01	5.70E-02
1214	27439	aparc-a2009s_lh_thickness_G-temporal-inf	Destrieux Atlas	Femoral neck BMD	MR Egger	5	0.69	0.24	2.00	5.49E-01
1214	27439	aparc-a2009s_lh_thickness_G-temporal-inf	Destrieux Atlas	Femoral neck BMD	Simple mode	5	0.81	0.63	1.05	1.79E-01
1214	27439	aparc-a2009s_lh_thickness_G-temporal-inf	Destrieux Atlas	Femoral neck BMD	Weighted median	5	0.80	0.66	0.97	1.98E-02
1214	27439	aparc-a2009s_lh_thickness_G-temporal-inf	Destrieux Atlas	Femoral neck BMD	Weighted mode	5	0.81	0.62	1.04	1.82E-01
1214	27439	aparc-a2009s_lh_thickness_G-temporal-inf	Destrieux Atlas	Forearm BMD	IVW	5	0.74	0.48	1.14	1.63E-01
1214	27439	aparc-a2009s_lh_thickness_G-temporal-inf	Destrieux Atlas	Forearm BMD	MR Egger	5	0.32	0.03	2.87	3.83E-01
1214	27439	aparc-a2009s_lh_thickness_G-temporal-inf	Destrieux Atlas	Forearm BMD	Simple mode	5	0.56	0.28	1.13	1.80E-01
1214	27439	aparc-a2009s_lh_thickness_G-temporal-inf	Destrieux Atlas	Forearm BMD	Weighted median	5	0.71	0.46	1.10	1.25E-01
1214	27439	aparc-a2009s_lh_thickness_G-temporal-inf	Destrieux Atlas	Forearm BMD	Weighted mode	5	0.60	0.30	1.18	2.11E-01
1214	27439	aparc-a2009s_lh_thickness_G-temporal-inf	Destrieux Atlas	Heel BMD	IVW	5	0.86	0.75	0.98	2.91E-02
1214	27439	aparc-a2009s_lh_thickness_G-temporal-inf	Destrieux Atlas	Heel BMD	MR Egger	5	0.74	0.35	1.57	4.80E-01
1214	27439	aparc-a2009s_lh_thickness_G-temporal-inf	Destrieux Atlas	Heel BMD	Simple mode	5	0.97	0.91	1.03	3.72E-01
1214	27439	aparc-a2009s_lh_thickness_G-temporal-inf	Destrieux Atlas	Heel BMD	Weighted median	5	0.95	0.90	1.01	1.12E-01
1214	27439	aparc-a2009s_lh_thickness_G-temporal-inf	Destrieux Atlas	Heel BMD	Weighted mode	5	0.97	0.92	1.03	3.55E-01
1214	27439	aparc-a2009s_lh_thickness_G-temporal-inf	Destrieux Atlas	Total body BMD	IVW	5	0.90	0.80	1.02	7.37E-02
1214	27439	aparc-a2009s_lh_thickness_G-temporal-inf	Destrieux Atlas	Total body BMD	MR Egger	5	0.81	0.46	1.43	5.21E-01
1214	27439	aparc-a2009s_lh_thickness_G-temporal-inf	Destrieux Atlas	Total body BMD	Simple mode	5	0.85	0.70	1.04	2.05E-01
1214	27439	aparc-a2009s_lh_thickness_G-temporal-inf	Destrieux Atlas	Total body BMD	Weighted median	5	0.90	0.77	1.05	1.67E-01
1214	27439	aparc-a2009s_lh_thickness_G-temporal-inf	Destrieux Atlas	Total body BMD	Weighted mode	5	0.88	0.72	1.09	3.20E-01
935	27392	aparc-a2009s_lh_area_S-orbital-H-Shaped	Destrieux Atlas	Lumbar spine BMD	IVW	9	1.12	0.93	1.35	2.02E-01
935	27392	aparc-a2009s_lh_area_S-orbital-H-Shaped	Destrieux Atlas	Lumbar spine BMD	MR Egger	9	3.33	1.71	6.50	8.60E-03
935	27392	aparc-a2009s_lh_area_S-orbital-H-Shaped	Destrieux Atlas	Lumbar spine BMD	Simple mode	9	0.96	0.73	1.27	7.62E-01
935	27392	aparc-a2009s_lh_area_S-orbital-H-Shaped	Destrieux Atlas	Lumbar spine BMD	Weighted median	9	1.09	0.91	1.31	3.95E-01
935	27392	aparc-a2009s_lh_area_S-orbital-H-Shaped	Destrieux Atlas	Lumbar spine BMD	Weighted mode	9	1.00	0.76	1.31	9.73E-01
935	27392	aparc-a2009s_lh_area_S-orbital-H-Shaped	Destrieux Atlas	Femoral neck BMD	IVW	9	1.16	1.01	1.34	4.20E-02
935	27392	aparc-a2009s_lh_area_S-orbital-H-Shaped	Destrieux Atlas	Femoral neck BMD	MR Egger	9	1.19	0.54	2.64	6.86E-01
935	27392	aparc-a2009s_lh_area_S-orbital-H-Shaped	Destrieux Atlas	Femoral neck BMD	Simple mode	9	1.05	0.88	1.26	5.74E-01
935	27392	aparc-a2009s_lh_area_S-orbital-H-Shaped	Destrieux Atlas	Femoral neck BMD	Weighted median	9	1.11	0.96	1.28	1.53E-01

935	27392	aparc-a2009s_lh_area_S-orbital-H-Shaped	Destrieux Atlas	Femoral neck BMD	Weighted mode	9	1.05	0.88	1.26	5.95E-01
935	27392	aparc-a2009s_lh_area_S-orbital-H-Shaped	Destrieux Atlas	Forearm BMD	IVW	9	1.02	0.82	1.28	8.46E-01
935	27392	aparc-a2009s_lh_area_S-orbital-H-Shaped	Destrieux Atlas	Forearm BMD	MR Egger	9	1.14	0.31	4.16	8.52E-01
935	27392	aparc-a2009s_lh_area_S-orbital-H-Shaped	Destrieux Atlas	Forearm BMD	Simple mode	9	1.19	0.74	1.91	4.80E-01
935	27392	aparc-a2009s_lh_area_S-orbital-H-Shaped	Destrieux Atlas	Forearm BMD	Weighted median	9	1.14	0.85	1.51	3.86E-01
935	27392	aparc-a2009s_lh_area_S-orbital-H-Shaped	Destrieux Atlas	Forearm BMD	Weighted mode	9	1.18	0.75	1.85	5.01E-01
935	27392	aparc-a2009s_lh_area_S-orbital-H-Shaped	Destrieux Atlas	Heel BMD	IVW	7	1.16	1.07	1.26	3.02E-04
935	27392	aparc-a2009s_lh_area_S-orbital-H-Shaped	Destrieux Atlas	Heel BMD	MR Egger	7	1.69	1.29	2.23	1.29E-02
935	27392	aparc-a2009s_lh_area_S-orbital-H-Shaped	Destrieux Atlas	Heel BMD	Simple mode	7	1.19	1.03	1.38	5.79E-02
935	27392	aparc-a2009s_lh_area_S-orbital-H-Shaped	Destrieux Atlas	Heel BMD	Weighted median	7	1.12	1.07	1.18	7.03E-06
935	27392	aparc-a2009s_lh_area_S-orbital-H-Shaped	Destrieux Atlas	Heel BMD	Weighted mode	7	1.05	0.92	1.20	5.01E-01
935	27392	aparc-a2009s_lh_area_S-orbital-H-Shaped	Destrieux Atlas	Total body BMD	IVW	9	0.97	0.85	1.11	6.51E-01
935	27392	aparc-a2009s_lh_area_S-orbital-H-Shaped	Destrieux Atlas	Total body BMD	MR Egger	9	1.35	0.60	3.06	5.03E-01
935	27392	aparc-a2009s_lh_area_S-orbital-H-Shaped	Destrieux Atlas	Total body BMD	Simple mode	9	1.05	0.91	1.22	5.40E-01
935	27392	aparc-a2009s_lh_area_S-orbital-H-Shaped	Destrieux Atlas	Total body BMD	Weighted median	9	1.03	0.93	1.15	6.24E-01
935	27392	aparc-a2009s_lh_area_S-orbital-H-Shaped	Destrieux Atlas	Total body BMD	Weighted mode	9	1.04	0.89	1.22	5.91E-01
5	25005	IDP_T1_SIENAX_grey_normalised_volume	IDP T1:global	Lumbar spine BMD	IVW	7	1.43	0.94	2.18	9.60E-02
5	25005	IDP_T1_SIENAX_grey_normalised_volume	IDP T1:global	Lumbar spine BMD	MR Egger	7	2.13	0.28	16.06	4.89E-01
5	25005	IDP_T1_SIENAX_grey_normalised_volume	IDP T1:global	Lumbar spine BMD	Simple mode	7	1.32	0.83	2.08	2.85E-01
5	25005	IDP_T1_SIENAX_grey_normalised_volume	IDP T1:global	Lumbar spine BMD	Weighted median	7	1.43	1.12	1.82	4.09E-03
5	25005	IDP_T1_SIENAX_grey_normalised_volume	IDP T1:global	Lumbar spine BMD	Weighted mode	7	1.37	1.04	1.81	6.20E-02
5	25005	IDP_T1_SIENAX_grey_normalised_volume	IDP T1:global	Femoral neck BMD	IVW	7	1.52	1.19	1.93	8.14E-04
5	25005	IDP_T1_SIENAX_grey_normalised_volume	IDP T1:global	Femoral neck BMD	MR Egger	7	3.33	1.33	8.33	4.91E-02
5	25005	IDP_T1_SIENAX_grey_normalised_volume	IDP T1:global	Femoral neck BMD	Simple mode	7	1.64	1.13	2.37	3.94E-02
5	25005	IDP_T1_SIENAX_grey_normalised_volume	IDP T1:global	Femoral neck BMD	Weighted median	7	1.45	1.18	1.79	5.91E-04
5	25005	IDP_T1_SIENAX_grey_normalised_volume	IDP T1:global	Femoral neck BMD	Weighted mode	7	1.64	1.24	2.18	1.43E-02
5	25005	IDP_T1_SIENAX_grey_normalised_volume	IDP T1:global	Forearm BMD	IVW	7	2.13	1.03	4.39	4.51E-02
5	25005	IDP_T1_SIENAX_grey_normalised_volume	IDP T1:global	Forearm BMD	MR Egger	7	25.00	1.67	375.00	6.66E-02
5	25005	IDP_T1_SIENAX_grey_normalised_volume	IDP T1:global	Forearm BMD	Simple mode	7	0.96	0.60	1.55	8.62E-01
5	25005	IDP_T1_SIENAX_grey_normalised_volume	IDP T1:global	Forearm BMD	Weighted median	7	1.18	0.76	1.82	4.75E-01
5	25005	IDP_T1_SIENAX_grey_normalised_volume	IDP T1:global	Forearm BMD	Weighted mode	7	0.92	0.60	1.41	6.98E-01
5	25005	IDP_T1_SIENAX_grey_normalised_volume	IDP T1:global	Heel BMD	IVW	8	1.49	0.99	2.26	5.76E-02
5	25005	IDP_T1_SIENAX_grey_normalised_volume	IDP T1:global	Heel BMD	MR Egger	8	16.67	7.45	37.27	9.08E-04

5	25005	IDP_T1_SIENAX_grey_normalised_volume	IDP T1:global	Heel BMD	Simple mode	8	1.19	1.11	1.28	1.94E-03
5	25005	IDP_T1_SIENAX_grey_normalised_volume	IDP T1:global	Heel BMD	Weighted median	8	1.14	1.07	1.21	3.77E-05
5	25005	IDP_T1_SIENAX_grey_normalised_volume	IDP T1:global	Heel BMD	Weighted mode	8	1.14	1.07	1.21	3.90E-03
5	25005	IDP_T1_SIENAX_grey_normalised_volume	IDP T1:global	Total body BMD	IVW	8	1.54	0.84	2.82	1.54E-01
5	25005	IDP_T1_SIENAX_grey_normalised_volume	IDP T1:global	Total body BMD	MR Egger	8	16.67	2.10	132.29	5.75E-02
5	25005	IDP_T1_SIENAX_grey_normalised_volume	IDP T1:global	Total body BMD	Simple mode	8	0.95	0.82	1.10	5.09E-01
5	25005	IDP_T1_SIENAX_grey_normalised_volume	IDP T1:global	Total body BMD	Weighted median	8	0.95	0.83	1.09	4.69E-01
5	25005	IDP_T1_SIENAX_grey_normalised_volume	IDP T1:global	Total body BMD	Weighted mode	8	0.93	0.82	1.06	3.36E-01
9	25009	IDP_T1_SIENAX_brain-normalised_volume	IDP T1:global	Lumbar spine BMD	IVW	9	1.23	0.95	1.60	1.01E-01
9	25009	IDP_T1_SIENAX_brain-normalised_volume	IDP T1:global	Lumbar spine BMD	MR Egger	9	2.38	1.01	5.64	8.75E-02
9	25009	IDP_T1_SIENAX_brain-normalised_volume	IDP T1:global	Lumbar spine BMD	Simple mode	9	1.56	1.32	1.85	6.27E-04
9	25009	IDP_T1_SIENAX_brain-normalised_volume	IDP T1:global	Lumbar spine BMD	Weighted median	9	1.43	1.21	1.68	3.99E-05
9	25009	IDP_T1_SIENAX_brain-normalised_volume	IDP T1:global	Lumbar spine BMD	Weighted mode	9	1.56	1.30	1.88	1.11E-03
9	25009	IDP_T1_SIENAX_brain-normalised_volume	IDP T1:global	Femoral neck BMD	IVW	9	1.30	1.12	1.51	5.85E-04
9	25009	IDP_T1_SIENAX_brain-normalised_volume	IDP T1:global	Femoral neck BMD	MR Egger	9	1.47	0.84	2.57	2.15E-01
9	25009	IDP_T1_SIENAX_brain-normalised_volume	IDP T1:global	Femoral neck BMD	Simple mode	9	1.32	1.05	1.65	4.54E-02
9	25009	IDP_T1_SIENAX_brain-normalised_volume	IDP T1:global	Femoral neck BMD	Weighted median	9	1.30	1.13	1.49	8.98E-05
9	25009	IDP_T1_SIENAX_brain-normalised_volume	IDP T1:global	Femoral neck BMD	Weighted mode	9	1.32	1.05	1.64	3.70E-02
9	25009	IDP_T1_SIENAX_brain-normalised_volume	IDP T1:global	Forearm BMD	IVW	9	1.43	0.82	2.48	2.05E-01
9	25009	IDP_T1_SIENAX_brain-normalised_volume	IDP T1:global	Forearm BMD	MR Egger	9	2.94	0.36	24.25	3.39E-01
9	25009	IDP_T1_SIENAX_brain-normalised_volume	IDP T1:global	Forearm BMD	Simple mode	9	0.86	0.59	1.26	4.69E-01
9	25009	IDP_T1_SIENAX_brain-normalised_volume	IDP T1:global	Forearm BMD	Weighted median	9	0.90	0.68	1.19	4.77E-01
9	25009	IDP_T1_SIENAX_brain-normalised_volume	IDP T1:global	Forearm BMD	Weighted mode	9	0.88	0.62	1.24	4.74E-01
9	25009	IDP_T1_SIENAX_brain-normalised_volume	IDP T1:global	Heel BMD	IVW	9	1.32	0.98	1.77	7.69E-02
9	25009	IDP_T1_SIENAX_brain-normalised_volume	IDP T1:global	Heel BMD	MR Egger	9	2.13	0.67	6.77	2.41E-01
9	25009	IDP_T1_SIENAX_brain-normalised_volume	IDP T1:global	Heel BMD	Simple mode	9	1.09	1.01	1.17	4.23E-02
9	25009	IDP_T1_SIENAX_brain-normalised_volume	IDP T1:global	Heel BMD	Weighted median	9	1.09	1.04	1.14	9.49E-05
9	25009	IDP_T1_SIENAX_brain-normalised_volume	IDP T1:global	Heel BMD	Weighted mode	9	1.08	1.02	1.13	2.52E-02
9	25009	IDP_T1_SIENAX_brain-normalised_volume	IDP T1:global	Total body BMD	IVW	9	1.23	0.80	1.90	3.49E-01
9	25009	IDP_T1_SIENAX_brain-normalised_volume	IDP T1:global	Total body BMD	MR Egger	9	2.13	0.37	12.15	4.10E-01
9	25009	IDP_T1_SIENAX_brain-normalised_volume	IDP T1:global	Total body BMD	Simple mode	9	0.99	0.85	1.15	8.70E-01
9	25009	IDP_T1_SIENAX_brain-normalised_volume	IDP T1:global	Total body BMD	Weighted median	9	0.96	0.86	1.08	4.92E-01
9	25009	IDP_T1_SIENAX_brain-normalised_volume	IDP T1:global	Total body BMD	Weighted mode	9	0.98	0.87	1.10	7.39E-01

93	25849	IDP_T1_FAST_ROIs_R_parahipp_gyrus_ant	IDP T1:unilateral regions	Lumbar spine BMD	IVW	2	1.82	1.35	2.44	6.24E-05
93	25849	IDP_T1_FAST_ROIs_R_parahipp_gyrus_ant	IDP T1:unilateral regions	Femoral neck BMD	IVW	2	1.89	1.14	3.13	1.35E-02
93	25849	IDP_T1_FAST_ROIs_R_parahipp_gyrus_ant	IDP T1:unilateral regions	Forearm BMD	IVW	2	5.88	0.95	36.42	6.94E-02
93	25849	IDP_T1_FAST_ROIs_R_parahipp_gyrus_ant	IDP T1:unilateral regions	Heel BMD	IVW	2	3.03	0.83	11.02	8.99E-02
93	25849	IDP_T1_FAST_ROIs_R_parahipp_gyrus_ant	IDP T1:unilateral regions	Total body BMD	IVW	2	4.00	0.96	16.65	5.69E-02
