# Supplementary material for: Causal associations of brain structure with bone mineral density: a large-scale genetic correlation study
Source: Bone Res. 2023 Jul 20;11:37. doi: 10.1038/s41413-023-00270-z (PMC10359275; doi:10.1038/s41413-023-00270-z)
Supplement: Supplementary file 6 — Supplementary Table 4. Results of the Cochran’s Q, MR-Egger-intercept, MR-PRESSO and MR steiger tests [file 41413_2023_270_MOESM6_ESM.pdf]

Supplemental table 4. Results of the Cochran's Q, MR-Egger-intercept, MR-PRESSO and MR steiger tests

No.	UKB ID	IDP.short.name	Category name	Outcome.name	SNP	mean.F.statistics	Q.heterogeneity	Q_df.heterogeneity	Q_pval.heterogeneity	egger_intercept.pleiotropy	se.pleiotropy	pval.pleiotropy	PRESSO_beta	PRESSO_sd	PRESSO_p	PRESSO_Outliers_corrected_beta	PRESSO_Outliers_corrected_sd	PRESSO_Outliers_corrected_p	PRESSO_Global_Test_RSSobs	PRSSobs	PRESS_Distortion_Test_beta	PRESSO_Distortion_Test_p	steiger-correct_causal_direction	steiger-steiger_pval
5	25005	IDP_T1_SIENAX_grey_normalised_volume	IDP T1:global	Femoral neck BMD	7	44.0	29.6	6	4.59E-05	4.52E-02	2.59E-02	1.41E-01	-4.14E-01	1.24E-01	1.55E-02	-4.68E-01	1.23E-01	3.16E-02	42.3	2.00E-03	11.5	NA	TRUE	3.47E-14
9	25009	IDP_T1_SIENAX_brain-normalised_volume	IDP T1:global	Femoral neck BMD	9	49.7	24.9	9	3.09E-03	8.30E-03	1.81E-02	6.58E-01	-2.56E-01	7.46E-02	7.41E-03	-2.17E-01	5.67E-02	6.44E-03	32.4	3.00E-03	-18.1	NA	TRUE	1.02E-29
36	25792	IDP_T1_FAST_ROIs_L_inf_front_gyrus_parsop	IDP T1:unilateral regions	Femoral neck BMD	1	33.5	NA	NA	NA	NA	NA	NA	NA	NA	NA	NA	NA	NA	NA	NA	NA	NA	TRUE	1.01E-11
36	25792	IDP_T1_FAST_ROIs_L_inf_front_gyrus_parsop	IDP T1:unilateral regions	Heel BMD	1	33.5	NA	NA	NA	NA	NA	NA	NA	NA	NA	NA	NA	NA	NA	NA	NA	NA	NA	NA
36	25792	IDP_T1_FAST_ROIs_L_inf_front_gyrus_parsop	IDP T1:unilateral regions	Total body BMD	1	37.4	NA	NA	NA	NA	NA	NA	NA	NA	NA	NA	NA	NA	NA	NA	NA	NA	TRUE	1.60E-16
47	25803	IDP_T1_FAST_ROIs_R_mid_temp_gyrus_ant	IDP T1:unilateral regions	Heel BMD	1	31.0	NA	NA	NA	NA	NA	NA	NA	NA	NA	NA	NA	NA	NA	NA	NA	NA	NA	NA
48	25804	IDP_T1_FAST_ROIs_L_mid_temp_gyrus_post	IDP T1:unilateral regions	Heel BMD	1	31.2	NA	NA	NA	NA	NA	NA	NA	NA	NA	NA	NA	NA	NA	NA	NA	NA	NA	NA
51	25807	IDP_T1_FAST_ROIs_R_mid_temp_gyrus_tempocc	IDP T1:unilateral regions	Heel BMD	1	30.1	NA	NA	NA	NA	NA	NA	NA	NA	NA	NA	NA	NA	NA	NA	NA	NA	NA	NA
52	25808	IDP_T1_FAST_ROIs_L_inf_temp_gyrus_ant	IDP T1:unilateral regions	Heel BMD	1	34.0	NA	NA	NA	NA	NA	NA	NA	NA	NA	NA	NA	NA	NA	NA	NA	NA	NA	NA
55	25811	IDP_T1_FAST_ROIs_R_inf_temp_gyrus_post	IDP T1:unilateral regions	Heel BMD	2	33.8	NA	NA	NA	NA	NA	NA	NA	NA	NA	NA	NA	NA	NA	NA	NA	NA	NA	NA
70	25826	IDP_T1_FAST_ROIs_L_latocc_cortex_inf	IDP T1:unilateral regions	Heel BMD	1	38.1	NA	NA	NA	NA	NA	NA	NA	NA	NA	NA	NA	NA	NA	NA	NA	NA	TRUE	3.94E-31
82	25838	IDP_T1_FAST_ROIs_L_cing_gyrus_ant	IDP T1:unilateral regions	Heel BMD	1	30.8	NA	NA	NA	NA	NA	NA	NA	NA	NA	NA	NA	NA	NA	NA	NA	NA	TRUE	5.42E-22
83	25839	IDP_T1_FAST_ROIs_R_cing_gyrus_ant	IDP T1:unilateral regions	Heel BMD	2	31.5	NA	NA	NA	NA	NA	NA	NA	NA	NA	NA	NA	NA	NA	NA	NA	NA	TRUE	5.42E-22
93	25849	IDP_T1_FAST_ROIs_R_parahipp_gyrus_ant	IDP T1:unilateral regions	Lumbar spine BMD	2	46.1	NA	NA	NA	NA	NA	NA	NA	NA	NA	NA	NA	NA	NA	NA	NA	NA	TRUE	3.58E-08
172	26521	aseg_global_volume_EstimatedTotalIntraCranial	aseg:global	Femoral neck	7	55.2	23.2	6	7.36E-04	1.20E-02	1.69E-02	5.08E-01	-3.53E-01	9.47E-02	9.74E-03	-1.45E-01	6.14E-02	7.79E-02	35.4	3.40E-02	-144.2	NA	TRUE	5.43E-26

				BMD																				
187	26536	aseg_global_volume-ratio_BrainSegVol-to-eTIV	aseg:global	Femoral neck BMD	15	51.8	94.2	16	4.27E-13	1.60E-03	1.50E-02	9.16E-01	4.11E-01	7.50E-02	4.97E-05	4.25E-01	5.14E-02	2.69E-06	105.3	<0.001	-3.2	NA	TRUE	2.55E-37
187	26536	aseg_global_volume-ratio_BrainSegVol-to-eTIV	aseg:global	Total body BMD	17	51.7	181.2	18	5.57E-29	-1.85E-02	1.26E-02	1.61E-01	8.29E-01	7.55E-02	2.06E-09	8.41E-01	6.33E-02	1.06E-09	202.6	<0.001	-1.4	NA	TRUE	9.58E-03
187	26536	aseg_global_volume-ratio_BrainSegVol-to-eTIV	aseg:global	Forearm BMD	15	51.8	52.9	16	7.93E-06	-4.82E-02	1.98E-02	2.82E-02	6.77E-01	1.17E-01	2.88E-05	4.87E-01	1.02E-01	2.46E-04	71.6	<0.001	39.0	NA	TRUE	5.49E-04
187	26536	aseg_global_volume-ratio_BrainSegVol-to-eTIV	aseg:global	Lumbar spine BMD	15	51.8	104.3	16	5.29E-15	2.45E-03	1.84E-02	8.96E-01	5.30E-01	9.27E-02	3.18E-05	5.52E-01	6.73E-02	1.02E-06	115.5	<0.001	-4.1	NA	TRUE	5.26E-29
187	26536	aseg_global_volume-ratio_BrainSegVol-to-eTIV	aseg:global	Heel BMD	15	54.0	3627.4	16	0.00E+00	-3.12E-02	1.87E-02	1.17E-01	6.99E-01	1.11E-01	1.06E-05	6.58E-01	5.84E-02	1.32E-06	4831.4	<0.001	6.2	NA	NA	NA
227	26604	AmygNuclei_lh_volume_Central-nucleus	Amygdala Nuclei	Heel BMD	2	122.1	NA	NA	NA	NA	NA	NA	NA	NA	NA	NA	NA	NA	NA	NA	NA	NA	TRUE	2.35E-49
229	26606	AmygNuclei_lh_volume_Cortical-nucleus	Amygdala Nuclei	Heel BMD	3	66.6	3.6	2	1.67E-01	-3.28E-03	3.14E-03	4.86E-01	4.52E-03	4.90E-02	9.30E-01	NA	NA	NA	8.1	3.73E-01	NA	NA	TRUE	4.69E-34
305	26682	ThalamNuclei_lh_volume_PuL	Thalamus Nuclei	Heel BMD	1	36.3	NA	NA	NA	NA	NA	NA	NA	NA	NA	NA	NA	NA	NA	NA	NA	NA	NA	NA
311	26688	ThalamNuclei_rh_volume_LGN	Thalamus Nuclei	Heel BMD	1	35.4	NA	NA	NA	NA	NA	NA	NA	NA	NA	NA	NA	NA	NA	NA	NA	NA	TRUE	3.62E-67
312	26689	ThalamNuclei_rh_volume_MGN	Thalamus Nuclei	Heel BMD	2	48.1	NA	NA	NA	NA	NA	NA	NA	NA	NA	NA	NA	NA	NA	NA	NA	NA	TRUE	3.62E-67
356	26801	aparc-Desikan_lh_volume_medialorbitofrontal	Desikan Atlas	Heel BMD	1	31.7	NA	NA	NA	NA	NA	NA	NA	NA	NA	NA	NA	NA	NA	NA	NA	NA	TRUE	5.52E-90
378	26891	aparc-Desikan_rh_volume_caudalanteriorcingulate	Desikan Atlas	Heel BMD	2	30.4	NA	NA	NA	NA	NA	NA	NA	NA	NA	NA	NA	NA	NA	NA	NA	NA	TRUE	6.03E-51
390	26903	aparc-Desikan_rh_volume_middletemporal	Desikan Atlas	Heel BMD	1	34.4	NA	NA	NA	NA	NA	NA	NA	NA	NA	NA	NA	NA	NA	NA	NA	NA	TRUE	4.07E-26
407	26920	aparc-Desikan_rh_volume_frontalpole	Desikan Atlas	Heel BMD	1	32.2	NA	NA	NA	NA	NA	NA	NA	NA	NA	NA	NA	NA	NA	NA	NA	NA	TRUE	1.59E-72
469	27298	aparc-DKTatlas_rh_volume_caudalanteriorcingulate	Desikan Atlas	Heel BMD	1	32.6	NA	NA	NA	NA	NA	NA	NA	NA	NA	NA	NA	NA	NA	NA	NA	NA	TRUE	1.25E-81
475	27304	aparc-DKTatlas_rh_volume_inferiortemporal	Desikan Atlas	Heel BMD	1	29.9	NA	NA	NA	NA	NA	NA	NA	NA	NA	NA	NA	NA	NA	NA	NA	NA	TRUE	2.18E-35
478	27307	aparc-DKTatlas_rh_volume_lateralorbitofrontal	Desikan Atlas	Heel BMD	7	39.6	43.6	6	8.93E-08	2.68E-02	2.60E-02	3.50E-01	-1.41E-01	3.73E-02	9.23E-03	-1.58E-01	2.01E-02	1.41E-03	59.0	1.00E-03	10.9	NA	TRUE	1.00E-42
492	27321	aparc-DKTatlas_rh_volume_rostralanteriorcingulate	Desikan Atlas	Heel BMD	1	31.8	NA	NA	NA	NA	NA	NA	NA	NA	NA	NA	NA	NA	NA	NA	NA	NA	TRUE	6.95E-70
505	27482	aparc-a2009s_lh_volume_G+S-cingul-Ant	Destrieux Atlas	Total body BMD	1	31.7	NA	NA	NA	NA	NA	NA	NA	NA	NA	NA	NA	NA	NA	NA	NA	NA	TRUE	4.18E-34
505	27482	aparc-a2009s_lh_volume_G+S-cingul-Ant	Destrieux Atlas	Heel BMD	1	31.7	NA	NA	NA	NA	NA	NA	NA	NA	NA	NA	NA	NA	NA	NA	NA	NA	TRUE	1.33E-48
506	27483	aparc-a2009s_lh_volume_G+S-cingul-Mid-Ant	Destrieux Atlas	Heel BMD	1	34.3	NA	NA	NA	NA	NA	NA	NA	NA	NA	NA	NA	NA	NA	NA	NA	NA	TRUE	1.33E-48
506	27483	aparc-a2009s_lh_volume_G+S-cingul-Mid-Ant	Destrieux Atlas	Total body BMD	1	34.3	NA	NA	NA	NA	NA	NA	NA	NA	NA	NA	NA	NA	NA	NA	NA	NA	TRUE	4.18E-34
531	27508	aparc-a2009s_lh_volume_G-subcallosal	Destrieux Atlas	Heel BMD	1	31.5	NA	NA	NA	NA	NA	NA	NA	NA	NA	NA	NA	NA	NA	NA	NA	NA	TRUE	6.95E-91
576	27701	aparc-a2009s_rh_volume_G+S-paracentral	Destrieux Atlas	Heel BMD	1	31.8	NA	NA	NA	NA	NA	NA	NA	NA	NA	NA	NA	NA	NA	NA	NA	NA	TRUE	4.16E-45
586	27711	aparc-a2009s_rh_volume_G-front-inf-Orbital	Destrieux Atlas	Heel BMD	1	30.3	NA	NA	NA	NA	NA	NA	NA	NA	NA	NA	NA	NA	NA	NA	NA	NA	TRUE	1.11E-153
590	27715	aparc-a2009s_rh_volume_G-Ins-lg+S-cent-ins	Destrieux Atlas	Heel BMD	1	36.8	NA	NA	NA	NA	NA	NA	NA	NA	NA	NA	NA	NA	NA	NA	NA	NA	TRUE	4.14E-24
604	27729	aparc-a2009s_rh_volume_G-rectus	Destrieux Atlas	Heel BMD	1	32.1	NA	NA	NA	NA	NA	NA	NA	NA	NA	NA	NA	NA	NA	NA	NA	NA	TRUE	5.58E-57

616	27741	aparc-a2009s_rh_volume_Pole-temporal	Destrieux Atlas	Heel BMD	1	32.3	NA	NA	NA	NA	NA	NA	NA	NA	NA	NA	NA	NA	NA	NA	NA	NA	TRUE	1.06E-39
656	26729	aparc-Desikan_lh_area_inferiortemporal	Desikan Atlas	Heel BMD	1	31.7	NA	NA	NA	NA	NA	NA	NA	NA	NA	NA	NA	NA	NA	NA	NA	NA	TRUE	2.52E-42
661	26734	aparc-Desikan_lh_area_medialorbitofrontal	Desikan Atlas	Total body BMD	1	32.2	NA	NA	NA	NA	NA	NA	NA	NA	NA	NA	NA	NA	NA	NA	NA	NA	TRUE	2.23E-92
661	26734	aparc-Desikan_lh_area_medialorbitofrontal	Desikan Atlas	Heel BMD	1	32.2	NA	NA	NA	NA	NA	NA	NA	NA	NA	NA	NA	NA	NA	NA	NA	NA	TRUE	2.22E-151
679	26752	aparc-Desikan_lh_area_frontalpole	Desikan Atlas	Heel BMD	1	56.8	NA	NA	NA	NA	NA	NA	NA	NA	NA	NA	NA	NA	NA	NA	NA	NA	TRUE	3.27E-65
707	26847	aparc-Desikan_rh_area_rostralanteriorcingulate	Desikan Atlas	Heel BMD	2	40.4	NA	NA	NA	NA	NA	NA	NA	NA	NA	NA	NA	NA	NA	NA	NA	NA	TRUE	8.95E-61
810	27143	aparc-DKTatlas_lh_area_caudalanteriorcingulate	Desikan Atlas	Total body BMD	1	45.7	NA	NA	NA	NA	NA	NA	NA	NA	NA	NA	NA	NA	NA	NA	NA	NA	TRUE	2.48E-26
810	27143	aparc-DKTatlas_lh_area_caudalanteriorcingulate	Desikan Atlas	Heel BMD	1	45.7	NA	NA	NA	NA	NA	NA	NA	NA	NA	NA	NA	NA	NA	NA	NA	NA	TRUE	6.19E-29
816	27149	aparc-DKTatlas_lh_area_inferiortemporal	Desikan Atlas	Heel BMD	1	34.3	NA	NA	NA	NA	NA	NA	NA	NA	NA	NA	NA	NA	NA	NA	NA	NA	NA	NA
864	27259	aparc-DKTatlas_rh_area_rostralanteriorcingulate	Desikan Atlas	Heel BMD	1	62.2	NA	NA	NA	NA	NA	NA	NA	NA	NA	NA	NA	NA	NA	NA	NA	NA	TRUE	7.63E-58
884	27341	aparc-a2009s_lh_area_G-front-inf-Orbital	Destrieux Atlas	Heel BMD	1	35.8	NA	NA	NA	NA	NA	NA	NA	NA	NA	NA	NA	NA	NA	NA	NA	NA	TRUE	5.38E-161
918	27375	aparc-a2009s_lh_area_S-circular-insula-ant	Destrieux Atlas	Femoral neck BMD	3	33.0	1.6	2	4.56E-01	-3.53E-02	6.77E-02	6.94E-01	3.77E-02	8.17E-02	6.64E-01	NA	NA	NA	140.8	7.00E-02	NA	NA	TRUE	4.51E-08
933	27390	aparc-a2009s_lh_area_S-orbital-lateral	Destrieux Atlas	Heel BMD	1	47.4	NA	NA	NA	NA	NA	NA	NA	NA	NA	NA	NA	NA	NA	NA	NA	NA	NA	NA
935	27392	aparc-a2009s_lh_area_S-orbital-H-Shaped	Destrieux Atlas	Heel BMD	7	41.0	60.7	6	3.18E-11	2.11E-02	7.71E-03	4.07E-02	-1.53E-01	4.23E-02	1.12E-02	-1.55E-01	3.24E-02	1.73E-02	88.5	<0.001	1.5	NA	TRUE	2.37E-47
1013	27618	aparc-a2009s_rh_area_S-precentral-inf-part	Destrieux Atlas	Heel BMD	1	30.0	NA	NA	NA	NA	NA	NA	NA	NA	NA	NA	NA	NA	NA	NA	NA	NA	TRUE	1.43E-61
1017	27622	aparc-a2009s_rh_area_S-temporal-inf	Destrieux Atlas	Heel BMD	1	29.9	NA	NA	NA	NA	NA	NA	NA	NA	NA	NA	NA	NA	NA	NA	NA	NA	TRUE	1.11E-56
1028	26763	aparc-Desikan_lh_thickness_inferiortemporal	Desikan Atlas	Lumbar spine BMD	4	45.0	2.5	3	4.81E-01	-2.14E-02	2.24E-02	4.40E-01	4.41E-01	8.34E-02	1.32E-02	NA	NA	NA	4.7	5.60E-01	NA	NA	TRUE	9.01E-10
1032	26767	aparc-Desikan_lh_thickness_lingual	Desikan Atlas	Heel BMD	4	35.4	4.7	3	1.94E-01	7.52E-03	3.79E-03	1.86E-01	-1.03E-01	2.41E-02	2.36E-02	NA	NA	NA	9.1	3.24E-01	NA	NA	TRUE	1.92E-27
1033	26768	aparc-Desikan_lh_thickness_medialorbitofrontal	Desikan Atlas	Heel BMD	1	35.1	NA	NA	NA	NA	NA	NA	NA	NA	NA	NA	NA	NA	NA	NA	NA	NA	TRUE	1.92E-27
1038	26773	aparc-Desikan_lh_thickness_parsorbitalis	Desikan Atlas	Heel BMD	1	29.8	NA	NA	NA	NA	NA	NA	NA	NA	NA	NA	NA	NA	NA	NA	NA	NA	TRUE	8.40E-51
1062	26864	aparc-Desikan_rh_thickness_inferiortemporal	Desikan Atlas	Total body BMD	8	35.2	2.2	7	9.45E-01	-3.66E-04	1.05E-02	9.73E-01	1.72E-01	2.63E-02	3.31E-04	NA	NA	NA	3.1	9.48E-01	NA	NA	TRUE	1.03E-23
1063	26865	aparc-Desikan_rh_thickness_isthmuscingulate	Desikan Atlas	Total body BMD	4	34.9	3.2	3	3.67E-01	2.89E-02	1.98E-02	2.83E-01	-2.43E-01	6.48E-02	3.30E-02	NA	NA	NA	5.6	4.49E-01	NA	NA	TRUE	1.17E-09
1183	27408	aparc-a2009s_lh_thickness_G+S-cingul-Ant	Destrieux Atlas	Heel BMD	1	58.8	NA	NA	NA	NA	NA	NA	NA	NA	NA	NA	NA	NA	NA	NA	NA	NA	TRUE	2.58E-47
1185	27410	aparc-a2009s_lh_thickness_G+S-cingul-Mid-Post	Destrieux Atlas	Heel BMD	1	31.7	NA	NA	NA	NA	NA	NA	NA	NA	NA	NA	NA	NA	NA	NA	NA	NA	TRUE	2.58E-47
1186	27411	aparc-a2009s_lh_thickness_G-cingul-Post-dorsal	Destrieux Atlas	Heel BMD	1	34.9	NA	NA	NA	NA	NA	NA	NA	NA	NA	NA	NA	NA	NA	NA	NA	NA	TRUE	2.58E-47
1190	27415	aparc-a2009s_lh_thickness_G-front-inf-Orbital	Destrieux Atlas	Heel BMD	1	34.2	NA	NA	NA	NA	NA	NA	NA	NA	NA	NA	NA	NA	NA	NA	NA	NA	TRUE	1.42E-42
1199	27424	aparc-a2009s_lh_thickness_G-oc-temp-med-Lingua	Destrieux Atlas	Heel BMD	1	40.1	NA	NA	NA	NA	NA	NA	NA	NA	NA	NA	NA	NA	NA	NA	NA	NA	TRUE	5.97E-34

		l																						
1210	27435	aparc-a2009s_lh_thickness_G-temp-sup-G-T-transv	Destrieux Atlas	Heel BMD	1	48.9	NA	NA	NA	NA	NA	NA	NA	NA	NA	NA	NA	NA	NA	NA	NA	NA	TRUE	3.08E-41
1214	27439	aparc-a2009s_lh_thickness_G-temporal-inf	Destrieux Atlas	Lumbar spine BMD	5	35.1	6.0	4	2.02E-01	-2.42E-02	2.94E-02	4.70E-01	4.10E-01	1.02E-01	1.60E-02	NA	NA	NA	9.7	2.80E-01	NA	NA	TRUE	7.52E-11
1219	27444	aparc-a2009s_lh_thickness_Pole-occipital	Destrieux Atlas	Heel BMD	2	63.1	NA	NA	NA	NA	NA	NA	NA	NA	NA	NA	NA	NA	NA	NA	NA	NA	TRUE	1.24E-29
1221	27446	aparc-a2009s_lh_thickness_S-calcarine	Destrieux Atlas	Heel BMD	1	31.0	NA	NA	NA	NA	NA	NA	NA	NA	NA	NA	NA	NA	NA	NA	NA	NA	TRUE	1.24E-29
1237	27462	aparc-a2009s_lh_thickness_S-oc-temp-lat	Destrieux Atlas	Heel BMD	1	54.6	NA	NA	NA	NA	NA	NA	NA	NA	NA	NA	NA	NA	NA	NA	NA	NA	TRUE	8.92E-33
1237	27462	aparc-a2009s_lh_thickness_S-oc-temp-lat	Destrieux Atlas	Lumbar spine BMD	1	54.6	NA	NA	NA	NA	NA	NA	NA	NA	NA	NA	NA	NA	NA	NA	NA	NA	TRUE	8.94E-15
1241	27466	aparc-a2009s_lh_thickness_S-orbital-H-Shaped	Destrieux Atlas	Heel BMD	2	52.7	NA	NA	NA	NA	NA	NA	NA	NA	NA	NA	NA	NA	NA	NA	NA	NA	TRUE	3.57E-18
1247	27472	aparc-a2009s_lh_thickness_S-suborbital	Destrieux Atlas	Heel BMD	1	34.6	NA	NA	NA	NA	NA	NA	NA	NA	NA	NA	NA	NA	NA	NA	NA	NA	TRUE	1.94E-29
1249	27474	aparc-a2009s_lh_thickness_S-temporal-inf	Destrieux Atlas	Heel BMD	1	36.7	NA	NA	NA	NA	NA	NA	NA	NA	NA	NA	NA	NA	NA	NA	NA	NA	TRUE	1.90E-28
1249	27474	aparc-a2009s_lh_thickness_S-temporal-inf	Destrieux Atlas	Lumbar spine BMD	1	36.7	NA	NA	NA	NA	NA	NA	NA	NA	NA	NA	NA	NA	NA	NA	NA	NA	TRUE	4.38E-15
1254	27627	aparc-a2009s_rh_thickness_G+S-paracentral	Destrieux Atlas	Heel BMD	1	72.1	NA	NA	NA	NA	NA	NA	NA	NA	NA	NA	NA	NA	NA	NA	NA	NA	TRUE	4.67E-24
1257	27630	aparc-a2009s_rh_thickness_G+S-cingul-Ant	Destrieux Atlas	Heel BMD	1	48.5	NA	NA	NA	NA	NA	NA	NA	NA	NA	NA	NA	NA	NA	NA	NA	NA	TRUE	2.34E-24
1293	27666	aparc-a2009s_rh_thickness_Pole-occipital	Destrieux Atlas	Heel BMD	1	136.2	NA	NA	NA	NA	NA	NA	NA	NA	NA	NA	NA	NA	NA	NA	NA	NA	TRUE	3.13E-41
1312	27685	aparc-a2009s_rh_thickness_S-oc-temp-med+Lingua l	Destrieux Atlas	Heel BMD	1	32.1	NA	NA	NA	NA	NA	NA	NA	NA	NA	NA	NA	NA	NA	NA	NA	NA	TRUE	1.18E-18
1315	27688	aparc-a2009s_rh_thickness_S-orbital-H-Shaped	Destrieux Atlas	Heel BMD	1	36.0	NA	NA	NA	NA	NA	NA	NA	NA	NA	NA	NA	NA	NA	NA	NA	NA	TRUE	1.18E-18
1322	27695	aparc-a2009s_rh_thickness_S-subparietal	Destrieux Atlas	Heel BMD	4	33.0	4.9	3	1.81E-01	-2.67E-02	1.22E-02	1.59E-01	8.83E-02	2.56E-02	4.09E-02	NA	NA	NA	9.0	2.97E-01	NA	NA	TRUE	7.52E-22
1324	27697	aparc-a2009s_rh_thickness_S-temporal-sup	Destrieux Atlas	Heel BMD	1	42.8	NA	NA	NA	NA	NA	NA	NA	NA	NA	NA	NA	NA	NA	NA	NA	NA	TRUE	7.52E-22
