# Supplementary material for: Causal associations of brain structure with bone mineral density: a large-scale genetic correlation study
Source: Bone Res. 2023 Jul 20;11:37. doi: 10.1038/s41413-023-00270-z (PMC10359275; doi:10.1038/s41413-023-00270-z)
Supplement: Supplementary file 7 — Supplementary Table 5. Results of confirmatory 2sMR between BIDPs and osteoporosis [file 41413_2023_270_MOESM7_ESM.pdf]

Supplemental table 5. Results of confirmatory 2sMR between BIDPs and osteoporosis

NO.	UKB ID	IDP short name	Outcome	Method	nsnp	OR	b	se	pval	FDR p
187	26536	aseg_global_volume-ratio_BrainSegVol-to-eTIV	OP	IVW	15	0.45	-0.80	0.16	1.03E-06	2.28E-05
166	26515	aseg_global_volume_BrainSegNotVent	OP	IVW	10	1.84	0.61	0.21	3.43E-03	3.77E-02
62	25818	IDP_T1_FAST_ROIs_L_supramarg_gyrus_ant	OP	IVW	4	0.35	-1.05	0.30	3.51E-04	4.67E-02
538	27515	aparc-a2009s_lh_volume_Lat-Fis-ant-Horizont	OP	WR	1	5.81	1.76	0.52	6.47E-04	7.02E-02
635	27760	aparc-a2009s_rh_volume_S-orbital-lateral	OP	WR	1	5.64	1.73	0.53	1.18E-03	7.02E-02
324	26701	ThalamNuclei_rh_volume_VA	OP	IVW	7	1.99	0.69	0.22	1.43E-03	7.46E-02
56	25812	IDP_T1_FAST_ROIs_L_inf_temp_gyrus_tempocc	OP	WR	1	0.17	-1.79	0.56	1.28E-03	8.51E-02
793	27070	BA-exvivo_lh_area_MT	OP	IVW	5	2.12	0.75	0.27	5.46E-03	1.53E-01
58	25814	IDP_T1_FAST_ROIs_L_postcent_gyrus	OP	IVW	4	0.34	-1.08	0.37	3.61E-03	1.60E-01
617	27742	aparc-a2009s_rh_volume_S-calcarine	OP	IVW	26	1.38	0.32	0.11	4.21E-03	1.67E-01
1248	27473	aparc-a2009s_lh_thickness_S-subparietal	OP	IVW	5	2.18	0.78	0.25	1.54E-03	1.68E-01
68	25824	IDP_T1_FAST_ROIs_L_latocc_cortex_sup	OP	WR	1	4.31	1.46	0.54	6.46E-03	2.15E-01
136	25892	IDP_T1_FAST_ROIs_brain_stem	OP	IVW	9	1.70	0.53	0.20	8.70E-03	2.31E-01
983	27588	aparc-a2009s_rh_area_G-temporal-middle	OP	IVW	4	0.39	-0.95	0.31	1.93E-03	2.51E-01
913	27370	aparc-a2009s_lh_area_Pole-occipital	OP	IVW	5	2.20	0.79	0.29	5.78E-03	2.62E-01
972	27577	aparc-a2009s_rh_area_G-parietal-sup	OP	IVW	6	0.53	-0.64	0.23	6.04E-03	2.62E-01
172	26521	aseg_global_volume_EstimatedTotalIntraCranial	OP	IVW	7	0.59	-0.52	0.25	3.73E-02	2.74E-01
506	27483	aparc-a2009s_lh_volume_G+S-cingul-Mid-Ant	OP	IVW	2	0.31	-1.16	0.45	9.75E-03	2.90E-01
598	27723	aparc-a2009s_rh_volume_G-pariet-inf-Angular	OP	IVW	2	0.39	-0.93	0.38	1.52E-02	3.01E-01
620	27745	aparc-a2009s_rh_volume_S-circular-insula-ant	OP	IVW	2	3.56	1.27	0.51	1.35E-02	3.01E-01
583	27708	aparc-a2009s_rh_volume_G-cingul-Post-ventral	OP	IVW	7	1.63	0.49	0.21	1.82E-02	3.09E-01
947	27552	aparc-a2009s_rh_area_G+S-occipital-inf	OP	IVW	4	2.20	0.79	0.31	1.05E-02	3.41E-01
1297	27670	aparc-a2009s_rh_thickness_S-cingul-Marginalis	OP	WR	1	4.90	1.59	0.58	6.46E-03	3.52E-01
783	27060	BA-exvivo_lh_area_BA2	OP	IVW	8	0.57	-0.56	0.25	2.61E-02	3.66E-01
801	27106	BA-exvivo_rh_area_BA4p	OP	IVW	7	1.52	0.42	0.20	3.98E-02	3.71E-01
1142	27200	aparc-DKTatlas_lh_thickness_superiorparietal	OP	IVW	6	2.12	0.75	0.25	3.19E-03	4.12E-01
340	26717	Brainstem_global_volume_Pons	OP	IVW	35	1.11	0.1	0.09	2.91E-01	4.36E-01
341	26718	Brainstem_global_volume_SCP	OP	IVW	5	0.71	-0.34	0.24	1.64E-01	4.36E-01
343	26720	Brainstem_global_volume_Whole-brainstem	OP	IVW	31	1.12	0.11	0.09	2.41E-01	4.36E-01
343	26720	Brainstem_global_volume_Whole-brainstem	OP	IVW	31	1.12	0.11	0.09	2.41E-01	4.36E-01

595	27720	aparc-a2009s_rh_volume_G-oc-temp-med-Lingual	OP	IVW	8	1.62	0.48	0.22	2.93E-02	4.36E-01
791	27068	BA-exvivo_lh_area_V1	OP	IVW	34	1.19	0.17	0.09	6.79E-02	4.75E-01
936	27393	aparc-a2009s_lh_area_S-parieto-occipital	OP	IVW	14	1.42	0.35	0.15	2.02E-02	5.25E-01
51	25807	IDP_T1_FAST_ROIs_R_mid_temp_gyrus_tempocc	OP	WR	1	4.35	1.47	0.70	3.59E-02	5.30E-01
87	25843	IDP_T1_FAST_ROIs_R_precun_cortex	OP	IVW	5	0.49	-0.72	0.32	2.71E-02	5.30E-01
99	25855	IDP_T1_FAST_ROIs_R_temp_fusif_cortex_ant	OP	IVW	3	0.49	-0.72	0.34	3.43E-02	5.30E-01
113	25869	IDP_T1_FAST_ROIs_R_planum_polare	OP	IVW	5	1.86	0.62	0.29	3.08E-02	5.30E-01
158	25914	IDP_T1_FAST_ROIs_R_cerebellum_VIIIb	OP	IVW	12	1.49	0.4	0.20	4.08E-02	5.43E-01
580	27705	aparc-a2009s_rh_volume_G+S-cingul-Mid-Ant	OP	IVW	2	0.43	-0.84	0.41	4.13E-02	5.46E-01
167	26516	aseg_global_volume_BrainSegNotVentSurf	OP	IVW	12	1.32	0.28	0.18	1.24E-01	5.47E-01
171	26520	aseg_global_volume_SupraTentorialNotVent	OP	IVW	9	1.40	0.34	0.21	1.02E-01	5.47E-01
290	26667	ThalamNuclei_lh_volume_PuM	OP	IVW	7	1.63	0.49	0.22	2.48E-02	5.55E-01
304	26681	ThalamNuclei_lh_volume_CL	OP	IVW	7	1.60	0.47	0.22	3.20E-02	5.55E-01
32	25788	IDP_T1_FAST_ROIs_L_mid_front_gyrus	OP	IVW	5	0.64	-0.44	0.26	9.02E-02	5.79E-01
36	25792	IDP_T1_FAST_ROIs_L_inf_front_gyrus_parsop	OP	IVW	2	0.52	-0.65	0.39	9.50E-02	5.79E-01
41	25797	IDP_T1_FAST_ROIs_R_temporal_pole	OP	IVW	3	0.59	-0.52	0.30	8.47E-02	5.79E-01
43	25799	IDP_T1_FAST_ROIs_R_sup_temp_gyrus_ant	OP	WR	1	0.33	-1.1	0.62	7.29E-02	5.79E-01
48	25804	IDP_T1_FAST_ROIs_L_mid_temp_gyrus_post	OP	WR	1	3.03	1.11	0.66	9.06E-02	5.79E-01
75	25831	IDP_T1_FAST_ROIs_R_front_med_cortex	OP	IVW	2	0.51	-0.68	0.41	1.00E-01	5.79E-01
90	25846	IDP_T1_FAST_ROIs_L_front_orb_cortex	OP	IVW	9	0.68	-0.38	0.20	5.45E-02	5.79E-01
92	25848	IDP_T1_FAST_ROIs_L_parahipp_gyrus_ant	OP	IVW	4	0.63	-0.47	0.27	7.85E-02	5.79E-01
98	25854	IDP_T1_FAST_ROIs_L_temp_fusif_cortex_ant	OP	IVW	2	0.49	-0.72	0.40	7.15E-02	5.79E-01
114	25870	IDP_T1_FAST_ROIs_L_heschl_gyrus	OP	IVW	4	2.27	0.82	0.44	6.09E-02	5.79E-01
137	25893	IDP_T1_FAST_ROIs_L_cerebellum_I-IV	OP	IVW	7	1.43	0.36	0.22	1.00E-01	5.79E-01
141	25897	IDP_T1_FAST_ROIs_L_cerebellum_VI	OP	IVW	15	1.40	0.34	0.19	7.20E-02	5.79E-01
154	25910	IDP_T1_FAST_ROIs_V_cerebellum_VIIIa	OP	IVW	20	0.78	-0.25	0.14	7.22E-02	5.79E-01
419	27096	BA-exvivo_lh_volume_V1	OP	IVW	19	1.28	0.25	0.13	4.56E-02	6.15E-01
422	27099	BA-exvivo_lh_volume_perirhinal	OP	WR	1	0.37	-0.99	0.50	4.55E-02	6.15E-01
245	26622	HippSubfield_lh_volume_CA1-body	OP	IVW	5	1.51	0.41	0.24	8.78E-02	6.29E-01
253	26630	HippSubfield_lh_volume_molecular-layer-HP-body	OP	IVW	5	0.58	-0.55	0.32	8.40E-02	6.29E-01
258	26635	HippSubfield_lh_volume_CA4-body	OP	IVW	10	0.70	-0.36	0.19	5.82E-02	6.29E-01
264	26641	HippSubfield_lh_volume_Whole-hippocampus	OP	IVW	16	0.76	-0.28	0.15	7.52E-02	6.29E-01
270	26647	HippSubfield_rh_volume_presubiculum-head	OP	IVW	5	1.60	0.47	0.26	7.29E-02	6.29E-01

273	26650	HippSubfield_rh_volume_parasubiculum	OP	WR	1	3.74	1.32	0.58	2.30E-02	6.29E-01
1048	26783	aparc-Desikan_lh_thickness_superiorparietal	OP	IVW	6	2.08	0.73	0.29	1.35E-02	6.33E-01
1122	27180	aparc-DKTatlas_lh_thickness_inferiortemporal	OP	IVW	3	0.38	-0.96	0.39	1.47E-02	6.33E-01
878	27335	aparc-a2009s_lh_area_G+S-cingul-Mid-Ant	OP	IVW	3	0.52	-0.65	0.33	4.64E-02	6.59E-01
887	27344	aparc-a2009s_lh_area_G-front-sup	OP	IVW	4	0.53	-0.63	0.32	5.07E-02	6.59E-01
890	27347	aparc-a2009s_lh_area_G-occipital-middle	OP	IVW	2	2.77	1.02	0.48	3.53E-02	6.59E-01
1001	27606	aparc-a2009s_rh_area_S-intrapariet+P-trans	OP	IVW	5	0.57	-0.56	0.28	4.44E-02	6.59E-01
1012	27617	aparc-a2009s_rh_area_S-postcentral	OP	IVW	6	0.61	-0.5	0.25	4.84E-02	6.59E-01
1187	27412	aparc-a2009s_lh_thickness_G-cingul-Post-ventral	OP	IVW	3	2.34	0.85	0.38	2.45E-02	6.64E-01
1204	27429	aparc-a2009s_lh_thickness_G-parietal-sup	OP	IVW	3	2.12	0.75	0.38	4.74E-02	6.64E-01
1228	27453	aparc-a2009s_lh_thickness_S-collat-transv-post	OP	IVW	2	2.25	0.81	0.41	4.57E-02	6.64E-01
1233	27458	aparc-a2009s_lh_thickness_S-intrapariet+P-trans	OP	IVW	4	2.10	0.74	0.32	2.11E-02	6.64E-01
1242	27467	aparc-a2009s_lh_thickness_S-parieto-occipital	OP	IVW	5	1.68	0.52	0.26	4.12E-02	6.64E-01
1262	27635	aparc-a2009s_rh_thickness_G-cuneus	OP	IVW	4	1.62	0.48	0.26	6.70E-02	6.64E-01
1263	27636	aparc-a2009s_rh_thickness_G-front-inf-Opercular	OP	IVW	5	0.59	-0.53	0.28	5.80E-02	6.64E-01
1271	27644	aparc-a2009s_rh_thickness_G-occipital-sup	OP	IVW	3	2.01	0.7	0.36	5.28E-02	6.64E-01
1322	27695	aparc-a2009s_rh_thickness_S-subparietal	OP	IVW	3	1.86	0.62	0.34	6.37E-02	6.64E-01
77	25833	IDP_T1_FAST_ROIs_R_juxtapos_lobule_cortex	OP	WR	1	0.41	-0.9	0.60	1.30E-01	7.09E-01
84	25840	IDP_T1_FAST_ROIs_L_cing_gyrus_post	OP	IVW	3	0.52	-0.65	0.45	1.49E-01	7.09E-01
91	25847	IDP_T1_FAST_ROIs_R_front_orb_cortex	OP	IVW	11	0.78	-0.25	0.17	1.43E-01	7.09E-01
127	25883	IDP_T1_FAST_ROIs_R_putamen	OP	IVW	20	1.25	0.22	0.15	1.41E-01	7.09E-01
148	25904	IDP_T1_FAST_ROIs_V_cerebellum_crus_II	OP	IVW	21	1.20	0.18	0.12	1.49E-01	7.09E-01
37	25793	IDP_T1_FAST_ROIs_R_inf_front_gyrus_parsop	OP	IVW	2	1.80	0.59	0.46	1.96E-01	7.33E-01
69	25825	IDP_T1_FAST_ROIs_R_latocc_cortex_sup	OP	IVW	2	2.64	0.97	0.73	1.86E-01	7.33E-01
71	25827	IDP_T1_FAST_ROIs_R_latocc_cortex_inf	OP	IVW	3	1.57	0.45	0.35	1.96E-01	7.33E-01
83	25839	IDP_T1_FAST_ROIs_R_cing_gyrus_ant	OP	IVW	2	0.53	-0.63	0.46	1.68E-01	7.33E-01
96	25852	IDP_T1_FAST_ROIs_L_lingual_gyrus	OP	IVW	4	1.45	0.37	0.29	1.98E-01	7.33E-01
109	25865	IDP_T1_FAST_ROIs_R_cent_operc_cortex	OP	IVW	2	0.57	-0.56	0.42	1.84E-01	7.33E-01
132	25888	IDP_T1_FAST_ROIs_L_amygdala	OP	IVW	4	0.66	-0.41	0.31	1.88E-01	7.33E-01
133	25889	IDP_T1_FAST_ROIs_R_amygdala	OP	IVW	4	0.67	-0.4	0.30	1.77E-01	7.33E-01
581	27706	aparc-a2009s_rh_volume_G+S-cingul-Mid-Post	OP	IVW	3	0.53	-0.63	0.34	6.80E-02	7.36E-01
622	27747	aparc-a2009s_rh_volume_S-circular-insula-sup	OP	IVW	3	1.65	0.5	0.27	6.53E-02	7.36E-01
342	26719	Brainstem_global_volume_Midbrain	OP	IVW	20	1.06	0.06	0.13	6.16E-01	7.40E-01

1232	27457	aparc-a2009s_lh_thickness_S-interm-prim-Jensen	OP	IVW	2	0.53	-0.64	0.37	8.20E-02	7.45E-01
912	27369	aparc-a2009s_lh_area_Lat-Fis-post	OP	IVW	7	1.48	0.39	0.22	7.51E-02	7.45E-01
914	27371	aparc-a2009s_lh_area_Pole-temporal	OP	IVW	2	0.50	-0.69	0.39	7.40E-02	7.45E-01
986	27591	aparc-a2009s_rh_area_Lat-Fis-post	OP	IVW	6	1.52	0.42	0.24	8.03E-02	7.45E-01
1008	27613	aparc-a2009s_rh_area_S-orbital-med-olfact	OP	IVW	4	0.67	-0.4	0.22	6.92E-02	7.45E-01
142	25898	IDP_T1_FAST_ROIs_V_cerebellum_VI	OP	IVW	17	0.84	-0.18	0.14	2.09E-01	7.48E-01
162	25918	IDP_T1_FAST_ROIs_L_cerebellum_X	OP	IVW	14	0.82	-0.2	0.16	2.14E-01	7.48E-01
27	25783	IDP_T1_FAST_ROIs_R_frontal_pole	OP	IVW	2	0.63	-0.46	0.41	2.59E-01	7.48E-01
70	25826	IDP_T1_FAST_ROIs_L_latocc_cortex_inf	OP	IVW	2	2.23	0.8	0.68	2.40E-01	7.48E-01
72	25828	IDP_T1_FAST_ROIs_L_intracalc_cortex	OP	IVW	28	1.14	0.13	0.11	2.47E-01	7.48E-01
79	25835	IDP_T1_FAST_ROIs_R_subcallosal_cortex	OP	IVW	12	0.76	-0.28	0.23	2.35E-01	7.48E-01
81	25837	IDP_T1_FAST_ROIs_R_paracing_gyrus	OP	IVW	3	0.63	-0.47	0.40	2.43E-01	7.48E-01
102	25858	IDP_T1_FAST_ROIs_L_temp_occ_fusif_cortex	OP	IVW	4	0.68	-0.39	0.34	2.50E-01	7.48E-01
110	25866	IDP_T1_FAST_ROIs_L_parietal_operc_cortex	OP	IVW	3	1.45	0.37	0.32	2.38E-01	7.48E-01
149	25905	IDP_T1_FAST_ROIs_R_cerebellum_crus_II	OP	IVW	26	1.14	0.13	0.12	2.58E-01	7.48E-01
953	27558	aparc-a2009s_rh_area_G+S-cingul-Mid-Post	OP	IVW	4	0.58	-0.54	0.32	8.63E-02	7.48E-01
256	26633	HippSubfield_lh_volume_GC-ML-DG-body	OP	IVW	8	0.74	-0.3	0.20	1.41E-01	7.71E-01
265	26642	HippSubfield_rh_volume_Hippocampal-tail	OP	IVW	17	0.83	-0.19	0.13	1.61E-01	7.71E-01
266	26643	HippSubfield_rh_volume_subiculum-body	OP	IVW	12	0.76	-0.27	0.19	1.52E-01	7.71E-01
510	27487	aparc-a2009s_lh_volume_G-cuneus	OP	IVW	15	1.30	0.26	0.15	8.65E-02	7.73E-01
511	27488	aparc-a2009s_lh_volume_G-front-inf-Opercular	OP	WR	1	2.41	0.88	0.63	1.60E-01	7.73E-01
515	27492	aparc-a2009s_lh_volume_G-front-sup	OP	IVW	2	0.59	-0.52	0.38	1.70E-01	7.73E-01
516	27493	aparc-a2009s_lh_volume_G-Ins-lg+S-cent-ins	OP	IVW	3	1.86	0.62	0.49	2.01E-01	7.73E-01
517	27494	aparc-a2009s_lh_volume_G-insular-short	OP	IVW	6	0.65	-0.43	0.27	1.13E-01	7.73E-01
519	27496	aparc-a2009s_lh_volume_G-occipital-sup	OP	IVW	4	1.62	0.48	0.32	1.32E-01	7.73E-01
523	27500	aparc-a2009s_lh_volume_G-orbital	OP	IVW	3	1.65	0.5	0.34	1.44E-01	7.73E-01
524	27501	aparc-a2009s_lh_volume_G-pariet-inf-Angular	OP	IVW	4	0.63	-0.47	0.34	1.59E-01	7.73E-01
526	27503	aparc-a2009s_lh_volume_G-parietal-sup	OP	IVW	10	0.71	-0.34	0.22	1.21E-01	7.73E-01
531	27508	aparc-a2009s_lh_volume_G-subcallosal	OP	WR	1	0.43	-0.85	0.60	1.62E-01	7.73E-01
539	27516	aparc-a2009s_lh_volume_Lat-Fis-ant-Vertical	OP	WR	1	2.01	0.7	0.54	1.96E-01	7.73E-01
542	27519	aparc-a2009s_lh_volume_Pole-temporal	OP	IVW	3	0.61	-0.5	0.33	1.34E-01	7.73E-01
543	27520	aparc-a2009s_lh_volume_S-calcarine	OP	IVW	21	1.17	0.16	0.12	1.99E-01	7.73E-01
555	27532	aparc-a2009s_lh_volume_S-intrapariet+P-trans	OP	IVW	8	0.76	-0.28	0.20	1.58E-01	7.73E-01

564	27541	aparc-a2009s_lh_volume_S-parieto-occipital	OP	IVW	16	1.25	0.22	0.16	1.50E-01	7.73E-01
567	27544	aparc-a2009s_lh_volume_S-precentral-inf-part	OP	WR	1	0.49	-0.71	0.55	1.96E-01	7.73E-01
584	27709	aparc-a2009s_rh_volume_G-cuneus	OP	IVW	18	1.21	0.19	0.13	1.38E-01	7.73E-01
600	27725	aparc-a2009s_rh_volume_G-parietal-sup	OP	IVW	5	0.63	-0.46	0.34	1.79E-01	7.73E-01
605	27730	aparc-a2009s_rh_volume_G-subcallosal	OP	IVW	2	1.73	0.55	0.36	1.33E-01	7.73E-01
624	27749	aparc-a2009s_rh_volume_S-collat-transv-post	OP	WR	1	2.27	0.82	0.61	1.80E-01	7.73E-01
295	26672	ThalamNuclei_lh_volume_PuA	OP	IVW	4	1.51	0.41	0.29	1.51E-01	7.76E-01
297	26674	ThalamNuclei_lh_volume_Pf	OP	IVW	12	0.76	-0.28	0.18	1.17E-01	7.76E-01
301	26678	ThalamNuclei_lh_volume_VA	OP	IVW	12	1.35	0.3	0.19	1.08E-01	7.76E-01
306	26683	ThalamNuclei_lh_volume_Pt	OP	IVW	4	0.67	-0.4	0.28	1.62E-01	7.76E-01
312	26689	ThalamNuclei_rh_volume_MGN	OP	IVW	3	0.57	-0.56	0.38	1.37E-01	7.76E-01
317	26694	ThalamNuclei_rh_volume_CM	OP	IVW	16	0.79	-0.24	0.15	1.12E-01	7.76E-01
320	26697	ThalamNuclei_rh_volume_MDm	OP	IVW	2	1.62	0.48	0.37	1.94E-01	7.76E-01
321	26698	ThalamNuclei_rh_volume_Pf	OP	IVW	7	0.76	-0.28	0.21	1.91E-01	7.76E-01
332	26709	ThalamNuclei_rh_volume_Pt	OP	IVW	7	0.70	-0.36	0.22	9.89E-02	7.76E-01
336	26713	ThalamNuclei_rh_volume_LD	OP	IVW	9	0.68	-0.38	0.28	1.83E-01	7.76E-01
761	26968	aparc-pial_rh_area_lingual	OP	IVW	12	1.62	0.48	0.17	4.15E-03	7.76E-01
347	26792	aparc-Desikan_lh_volume_cuneus	OP	IVW	19	1.30	0.26	0.13	4.36E-02	7.84E-01
361	26806	aparc-Desikan_lh_volume_parsorbitalis	OP	IVW	4	2.51	0.92	0.40	2.05E-02	7.84E-01
381	26894	aparc-Desikan_rh_volume_entorhinal	OP	IVW	2	0.45	-0.79	0.39	4.52E-02	7.84E-01
440	27207	aparc-DKTatlas_lh_volume_cuneus	OP	IVW	16	1.48	0.39	0.14	6.71E-03	7.84E-01
454	27221	aparc-DKTatlas_lh_volume_parsorbitalis	OP	IVW	2	2.16	0.77	0.39	4.61E-02	7.84E-01
462	27229	aparc-DKTatlas_lh_volume_rostralmiddlefrontal	OP	IVW	5	1.70	0.53	0.26	4.47E-02	7.84E-01
498	27327	aparc-DKTatlas_rh_volume_transversetemporal	OP	IVW	3	2.08	0.73	0.34	3.30E-02	7.84E-01
85	25841	IDP_T1_FAST_ROIs_R_cing_gyrus_post	OP	IVW	4	1.43	0.36	0.34	2.89E-01	7.85E-01
97	25853	IDP_T1_FAST_ROIs_R_lingual_gyrus	OP	IVW	7	1.30	0.26	0.24	2.80E-01	7.85E-01
120	25876	IDP_T1_FAST_ROIs_L_occ_pole	OP	IVW	6	1.32	0.28	0.26	2.86E-01	7.85E-01
899	27356	aparc-a2009s_lh_area_G-postcentral	OP	IVW	5	0.68	-0.39	0.24	1.07E-01	7.87E-01
984	27589	aparc-a2009s_rh_area_Lat-Fis-ant-Horizont	OP	WR	1	2.56	0.94	0.58	1.07E-01	7.87E-01
1004	27609	aparc-a2009s_rh_area_S-occipital-ant	OP	IVW	4	1.68	0.52	0.32	1.09E-01	7.87E-01
553	27530	aparc-a2009s_lh_volume_S-front-sup	OP	IVW	2	2.97	1.09	0.89	2.20E-01	7.95E-01
559	27536	aparc-a2009s_lh_volume_S-oc-temp-lat	OP	IVW	2	1.65	0.5	0.40	2.17E-01	7.95E-01
805	27110	BA-exvivo_rh_area_V1	OP	IVW	32	1.17	0.16	0.11	1.42E-01	7.98E-01

784	27061	BA-exvivo_lh_area_BA3a	OP	IVW	4	1.46	0.38	0.32	2.35E-01	8.08E-01
785	27062	BA-exvivo_lh_area_BA3b	OP	IVW	8	1.27	0.24	0.21	2.59E-01	8.08E-01
787	27064	BA-exvivo_lh_area_BA4p	OP	IVW	6	1.36	0.31	0.28	2.70E-01	8.08E-01
792	27069	BA-exvivo_lh_area_V2	OP	IVW	24	1.13	0.12	0.11	3.07E-01	8.08E-01
797	27102	BA-exvivo_rh_area_BA2	OP	IVW	5	0.78	-0.25	0.25	3.18E-01	8.08E-01
806	27111	BA-exvivo_rh_area_V2	OP	IVW	23	1.17	0.16	0.14	2.69E-01	8.08E-01
170	26519	aseg_global_volume_SupraTentorial	OP	IVW	11	1.20	0.18	0.18	3.03E-01	8.10E-01
173	26522	aseg_global_volume_VentricleChoroid	OP	IVW	15	1.17	0.16	0.15	2.78E-01	8.10E-01
176	26525	aseg_global_volume_5th-Ventricle	OP	IVW	2	1.05	0.05	0.06	3.68E-01	8.10E-01
178	26527	aseg_global_volume_CSF	OP	IVW	11	0.82	-0.2	0.17	2.29E-01	8.10E-01
184	26533	aseg_global_volume_CC-Central	OP	IVW	8	0.83	-0.19	0.20	3.42E-01	8.10E-01
1098	27083	BA-exvivo_lh_thickness_V2	OP	IVW	7	1.58	0.46	0.22	3.81E-02	8.17E-01
1106	27119	BA-exvivo_rh_thickness_BA4a	OP	IVW	5	1.55	0.44	0.24	6.28E-02	8.17E-01
509	27486	aparc-a2009s_lh_volume_G-cingul-Post-ventral	OP	IVW	5	1.28	0.25	0.26	3.40E-01	8.25E-01
514	27491	aparc-a2009s_lh_volume_G-front-middle	OP	IVW	6	1.39	0.33	0.34	3.32E-01	8.25E-01
534	27511	aparc-a2009s_lh_volume_G-temp-sup-Plan-polar	OP	WR	1	0.55	-0.6	0.61	3.25E-01	8.25E-01
540	27517	aparc-a2009s_lh_volume_Lat-Fis-post	OP	IVW	5	1.28	0.25	0.25	3.02E-01	8.25E-01
545	27522	aparc-a2009s_lh_volume_S-cingul-Marginalis	OP	IVW	4	0.73	-0.32	0.27	2.45E-01	8.25E-01
566	27543	aparc-a2009s_lh_volume_S-postcentral	OP	IVW	4	0.73	-0.31	0.32	3.32E-01	8.25E-01
577	27702	aparc-a2009s_rh_volume_G+S-subcentral	OP	IVW	5	0.66	-0.42	0.43	3.32E-01	8.25E-01
577	27702	aparc-a2009s_rh_volume_G+S-subcentral	OP	IVW	5	0.66	-0.42	0.43	3.32E-01	8.25E-01
586	27711	aparc-a2009s_rh_volume_G-front-inf-Orbital	OP	WR	1	0.55	-0.6	0.60	3.11E-01	8.25E-01
587	27712	aparc-a2009s_rh_volume_G-front-inf-Triangul	OP	IVW	4	1.60	0.47	0.48	3.29E-01	8.25E-01
592	27717	aparc-a2009s_rh_volume_G-occipital-middle	OP	IVW	5	1.38	0.32	0.27	2.42E-01	8.25E-01
593	27718	aparc-a2009s_rh_volume_G-occipital-sup	OP	IVW	5	1.30	0.26	0.26	3.11E-01	8.25E-01
606	27731	aparc-a2009s_rh_volume_G-temp-sup-G-T-transv	OP	WR	1	1.95	0.67	0.60	2.63E-01	8.25E-01
621	27746	aparc-a2009s_rh_volume_S-circular-insula-inf	OP	WR	1	1.86	0.62	0.61	3.13E-01	8.25E-01
628	27753	aparc-a2009s_rh_volume_S-interm-prim-Jensen	OP	WR	1	1.90	0.64	0.56	2.53E-01	8.25E-01
644	27769	aparc-a2009s_rh_volume_S-subparietal	OP	IVW	5	1.32	0.28	0.26	2.80E-01	8.25E-01
28	25784	IDP_T1_FAST_ROIs_L_insular_cortex	OP	IVW	15	1.19	0.17	0.17	3.36E-01	8.28E-01
49	25805	IDP_T1_FAST_ROIs_R_mid_temp_gyrus_post	OP	WR	1	0.31	-1.16	1.18	3.26E-01	8.28E-01
63	25819	IDP_T1_FAST_ROIs_R_supramarg_gyrus_ant	OP	IVW	2	0.63	-0.47	0.47	3.20E-01	8.28E-01
126	25882	IDP_T1_FAST_ROIs_L_putamen	OP	IVW	27	1.12	0.11	0.11	3.24E-01	8.28E-01

129	25885	IDP_T1_FAST_ROIs_R_pallidum	OP	IVW	4	1.40	0.34	0.35	3.33E-01	8.28E-01
169	26518	aseg_global_volume_TotalGray	OP	IVW	9	1.14	0.13	0.24	5.92E-01	8.30E-01
174	26523	aseg_global_volume_3rd-Ventricle	OP	IVW	17	1.08	0.08	0.12	5.18E-01	8.30E-01
177	26526	aseg_global_volume_Brain-Stem	OP	IVW	32	1.05	0.05	0.09	6.04E-01	8.30E-01
179	26528	aseg_global_volume_WM-hypointensities	OP	IVW	13	1.15	0.14	0.19	4.52E-01	8.30E-01
182	26531	aseg_global_volume_CC-Posterior	OP	IVW	21	1.09	0.09	0.15	5.38E-01	8.30E-01
186	26535	aseg_global_volume_CC-Anterior	OP	IVW	13	1.11	0.1	0.16	5.30E-01	8.30E-01
93	25849	IDP_T1_FAST_ROIs_R_parahipp_gyrus_ant	OP	IVW	3	0.71	-0.34	0.37	3.51E-01	8.32E-01
100	25856	IDP_T1_FAST_ROIs_L_temp_fusif_cortex_post	OP	IVW	9	1.28	0.25	0.27	3.47E-01	8.32E-01
140	25896	IDP_T1_FAST_ROIs_R_cerebellum_V	OP	IVW	11	0.83	-0.19	0.21	3.56E-01	8.32E-01
410	27087	BA-exvivo_lh_volume_BA1	OP	IVW	3	1.34	0.29	0.37	4.28E-01	8.33E-01
411	27088	BA-exvivo_lh_volume_BA2	OP	IVW	3	0.44	-0.82	0.76	2.79E-01	8.33E-01
412	27089	BA-exvivo_lh_volume_BA3a	OP	IVW	2	0.58	-0.55	0.73	4.53E-01	8.33E-01
413	27090	BA-exvivo_lh_volume_BA3b	OP	IVW	3	0.54	-0.61	0.64	3.43E-01	8.33E-01
414	27091	BA-exvivo_lh_volume_BA4a	OP	IVW	2	0.61	-0.49	0.51	3.28E-01	8.33E-01
415	27092	BA-exvivo_lh_volume_BA4p	OP	IVW	2	1.93	0.66	0.40	9.88E-02	8.33E-01
416	27093	BA-exvivo_lh_volume_BA6	OP	IVW	7	0.86	-0.15	0.20	4.65E-01	8.33E-01
425	27130	BA-exvivo_rh_volume_BA2	OP	IVW	2	0.76	-0.28	0.39	4.76E-01	8.33E-01
426	27131	BA-exvivo_rh_volume_BA3a	OP	IVW	4	0.63	-0.46	0.34	1.82E-01	8.33E-01
427	27132	BA-exvivo_rh_volume_BA3b	OP	IVW	8	1.16	0.15	0.19	4.43E-01	8.33E-01
431	27136	BA-exvivo_rh_volume_BA44	OP	IVW	2	1.72	0.54	0.41	1.87E-01	8.33E-01
433	27138	BA-exvivo_rh_volume_V1	OP	IVW	15	1.11	0.1	0.15	4.94E-01	8.33E-01
434	27139	BA-exvivo_rh_volume_V2	OP	IVW	15	1.20	0.18	0.17	2.88E-01	8.33E-01
436	27141	BA-exvivo_rh_volume_perirhinal	OP	IVW	2	0.51	-0.68	0.65	2.93E-01	8.33E-01
86	25842	IDP_T1_FAST_ROIs_L_precun_cortex	OP	IVW	4	0.69	-0.37	0.41	3.76E-01	8.33E-01
143	25899	IDP_T1_FAST_ROIs_R_cerebellum_VI	OP	IVW	15	1.21	0.19	0.21	3.70E-01	8.33E-01
151	25907	IDP_T1_FAST_ROIs_V_cerebellum_VIIb	OP	IVW	12	1.16	0.15	0.16	3.71E-01	8.33E-01
533	27510	aparc-a2009s_lh_volume_G-temp-sup-Lateral	OP	IVW	2	0.61	-0.5	0.54	3.57E-01	8.34E-01
585	27710	aparc-a2009s_rh_volume_G-front-inf-Opercular	OP	IVW	2	0.68	-0.38	0.41	3.56E-01	8.34E-01
990	27595	aparc-a2009s_rh_area_S-central	OP	IVW	6	1.43	0.36	0.23	1.24E-01	8.46E-01
345	26790	aparc-Desikan_lh_volume_caudalanteriorcingulate	OP	WR	1	2.69	0.99	0.56	7.48E-02	8.63E-01
356	26801	aparc-Desikan_lh_volume_medialorbitofrontal	OP	WR	1	0.36	-1.02	0.56	7.01E-02	8.63E-01
369	26814	aparc-Desikan_lh_volume_rostralmiddlefrontal	OP	IVW	7	1.79	0.58	0.35	9.43E-02	8.63E-01

374	26819	aparc-Desikan_lh_volume_frontalpole	OP	WR	1	0.39	-0.95	0.57	9.43E-02	8.63E-01
380	26893	aparc-Desikan_rh_volume_cuneus	OP	IVW	21	1.22	0.2	0.12	9.40E-02	8.63E-01
471	27300	aparc-DKTatlas_rh_volume_cuneus	OP	IVW	17	1.27	0.24	0.13	7.23E-02	8.63E-01
34	25790	IDP_T1_FAST_ROIs_L_inf_front_gyrus_parstri	OP	IVW	4	1.51	0.41	0.49	4.03E-01	8.75E-01
116	25872	IDP_T1_FAST_ROIs_L_planum_temporale	OP	IVW	7	1.19	0.17	0.20	4.08E-01	8.75E-01
915	27372	aparc-a2009s_lh_area_S-calcarine	OP	IVW	29	1.16	0.15	0.10	1.35E-01	8.77E-01
420	27097	BA-exvivo_lh_volume_V2	OP	IVW	11	1.09	0.09	0.17	5.76E-01	8.80E-01
421	27098	BA-exvivo_lh_volume_MT	OP	IVW	3	1.21	0.19	0.35	5.87E-01	8.80E-01
424	27129	BA-exvivo_rh_volume_BA1	OP	WR	1	1.27	0.24	0.48	6.19E-01	8.80E-01
476	27305	aparc-DKTatlas_rh_volume_isthmuscingulate	OP	IVW	2	1.97	0.68	0.42	1.04E-01	8.86E-01
604	27729	aparc-a2009s_rh_volume_G-rectus	OP	WR	1	1.84	0.61	0.71	3.90E-01	8.92E-01
882	27339	aparc-a2009s_lh_area_G-cuneus	OP	IVW	21	1.19	0.17	0.12	1.49E-01	8.93E-01
891	27348	aparc-a2009s_lh_area_G-occipital-sup	OP	IVW	2	1.92	0.65	0.48	1.77E-01	8.93E-01
895	27352	aparc-a2009s_lh_area_G-orbital	OP	IVW	6	1.34	0.29	0.24	2.28E-01	8.93E-01
896	27353	aparc-a2009s_lh_area_G-pariet-inf-Angular	OP	IVW	3	0.58	-0.54	0.45	2.34E-01	8.93E-01
898	27355	aparc-a2009s_lh_area_G-parietal-sup	OP	IVW	9	0.77	-0.26	0.20	1.87E-01	8.93E-01
907	27364	aparc-a2009s_lh_area_G-temp-sup-Plan-tempo	OP	WR	1	0.42	-0.86	0.68	2.08E-01	8.93E-01
931	27388	aparc-a2009s_lh_area_S-oc-temp-lat	OP	WR	1	1.72	0.54	0.38	1.58E-01	8.93E-01
943	27400	aparc-a2009s_lh_area_S-temporal-inf	OP	WR	1	2.03	0.71	0.58	2.20E-01	8.93E-01
949	27554	aparc-a2009s_rh_area_G+S-subcentral	OP	IVW	5	0.61	-0.5	0.38	1.91E-01	8.93E-01
956	27561	aparc-a2009s_rh_area_G-cuneus	OP	IVW	21	1.15	0.14	0.12	2.29E-01	8.93E-01
960	27565	aparc-a2009s_rh_area_G-front-middle	OP	IVW	3	1.52	0.42	0.33	2.09E-01	8.93E-01
974	27579	aparc-a2009s_rh_area_G-precentral	OP	IVW	3	1.51	0.41	0.29	1.53E-01	8.93E-01
994	27599	aparc-a2009s_rh_area_S-circular-insula-sup	OP	IVW	7	1.42	0.35	0.27	1.92E-01	8.93E-01
1017	27622	aparc-a2009s_rh_area_S-temporal-inf	OP	WR	1	0.44	-0.82	0.60	1.68E-01	8.93E-01
1020	26755	aparc-Desikan_lh_thickness_GlobalMeanThickness	OP	IVW	9	1.12	0.11	0.20	5.84E-01	8.95E-01
1021	26756	aparc-Desikan_lh_thickness_bankssts	OP	WR	1	2.46	0.9	0.60	1.32E-01	8.95E-01
1023	26758	aparc-Desikan_lh_thickness_caudalmiddlefrontal	OP	IVW	3	0.90	-0.1	0.27	7.08E-01	8.95E-01
1024	26759	aparc-Desikan_lh_thickness_cuneus	OP	IVW	3	1.55	0.44	0.36	2.23E-01	8.95E-01
1026	26761	aparc-Desikan_lh_thickness_fusiform	OP	IVW	3	1.32	0.28	0.28	3.20E-01	8.95E-01
1028	26763	aparc-Desikan_lh_thickness_inferiortemporal	OP	IVW	2	0.78	-0.25	0.51	6.19E-01	8.95E-01
1029	26764	aparc-Desikan_lh_thickness_isthmuscingulate	OP	IVW	4	1.49	0.4	0.32	2.22E-01	8.95E-01
1031	26766	aparc-Desikan_lh_thickness_lateralorbitofrontal	OP	IVW	3	0.85	-0.16	0.41	6.98E-01	8.95E-01

1032	26767	aparc-Desikan_lh_thickness_lingual	OP	IVW	3	0.57	-0.56	0.38	1.45E-01	8.95E-01
1035	26770	aparc-Desikan_lh_thickness_parahippocampal	OP	IVW	2	1.55	0.44	0.79	5.78E-01	8.95E-01
1036	26771	aparc-Desikan_lh_thickness_paracentral	OP	IVW	5	1.36	0.31	0.25	2.10E-01	8.95E-01
1040	26775	aparc-Desikan_lh_thickness_pericalcarine	OP	IVW	2	1.40	0.34	0.55	5.42E-01	8.95E-01
1041	26776	aparc-Desikan_lh_thickness_postcentral	OP	IVW	5	1.22	0.2	0.24	4.15E-01	8.95E-01
1042	26777	aparc-Desikan_lh_thickness_posteriorcingulate	OP	WR	1	0.52	-0.66	0.59	2.64E-01	8.95E-01
1043	26778	aparc-Desikan_lh_thickness_precentral	OP	IVW	3	0.80	-0.22	0.33	5.03E-01	8.95E-01
1044	26779	aparc-Desikan_lh_thickness_precuneus	OP	IVW	7	1.35	0.3	0.25	2.28E-01	8.95E-01
1046	26781	aparc-Desikan_lh_thickness_rostralmiddlefrontal	OP	IVW	7	0.90	-0.1	0.21	6.19E-01	8.95E-01
1047	26782	aparc-Desikan_lh_thickness_superiorfrontal	OP	IVW	8	0.92	-0.08	0.22	6.98E-01	8.95E-01
1049	26784	aparc-Desikan_lh_thickness_superiortemporal	OP	IVW	2	1.36	0.31	0.50	5.35E-01	8.95E-01
1051	26786	aparc-Desikan_lh_thickness_frontalpole	OP	WR	1	0.49	-0.71	0.57	2.12E-01	8.95E-01
1052	26787	aparc-Desikan_lh_thickness_transversetemporal	OP	IVW	3	1.82	0.6	0.36	9.70E-02	8.95E-01
1053	26788	aparc-Desikan_lh_thickness_insula	OP	IVW	2	0.60	-0.51	1.22	6.78E-01	8.95E-01
1055	26857	aparc-Desikan_rh_thickness_bankssts	OP	WR	1	1.90	0.64	0.59	2.82E-01	8.95E-01
1056	26858	aparc-Desikan_rh_thickness_caudalanteriorcingulate	OP	IVW	3	1.58	0.46	0.42	2.77E-01	8.95E-01
1057	26859	aparc-Desikan_rh_thickness_caudalmiddlefrontal	OP	IVW	2	0.84	-0.17	0.41	6.81E-01	8.95E-01
1058	26860	aparc-Desikan_rh_thickness_cuneus	OP	IVW	6	1.54	0.43	0.23	6.62E-02	8.95E-01
1060	26862	aparc-Desikan_rh_thickness_fusiform	OP	IVW	3	0.72	-0.33	0.75	6.56E-01	8.95E-01
1062	26864	aparc-Desikan_rh_thickness_inferiortemporal	OP	IVW	5	1.22	0.2	0.30	5.20E-01	8.95E-01
1063	26865	aparc-Desikan_rh_thickness_isthmuscingulate	OP	IVW	3	1.75	0.56	0.42	1.86E-01	8.95E-01
1064	26866	aparc-Desikan_rh_thickness_lateraloccipital	OP	IVW	7	1.60	0.47	0.22	3.18E-02	8.95E-01
1065	26867	aparc-Desikan_rh_thickness_lateralorbitofrontal	OP	IVW	4	0.81	-0.21	0.41	6.17E-01	8.95E-01
1066	26868	aparc-Desikan_rh_thickness_lingual	OP	IVW	6	0.68	-0.39	0.28	1.62E-01	8.95E-01
1067	26869	aparc-Desikan_rh_thickness_medialorbitofrontal	OP	IVW	3	1.15	0.14	0.35	6.85E-01	8.95E-01
1068	26870	aparc-Desikan_rh_thickness_middletemporal	OP	IVW	2	1.82	0.6	0.42	1.55E-01	8.95E-01
1070	26872	aparc-Desikan_rh_thickness_paracentral	OP	IVW	3	1.27	0.24	0.33	4.66E-01	8.95E-01
1071	26873	aparc-Desikan_rh_thickness_parsopercularis	OP	IVW	2	1.17	0.16	0.38	6.69E-01	8.95E-01
1073	26875	aparc-Desikan_rh_thickness_parstriangularis	OP	WR	1	0.52	-0.65	0.58	2.64E-01	8.95E-01
1074	26876	aparc-Desikan_rh_thickness_pericalcarine	OP	IVW	3	0.70	-0.35	0.36	3.31E-01	8.95E-01
1075	26877	aparc-Desikan_rh_thickness_postcentral	OP	IVW	4	1.12	0.11	0.27	6.91E-01	8.95E-01
1077	26879	aparc-Desikan_rh_thickness_precentral	OP	IVW	7	0.85	-0.16	0.27	5.54E-01	8.95E-01
1078	26880	aparc-Desikan_rh_thickness_precuneus	OP	IVW	8	1.21	0.19	0.20	3.36E-01	8.95E-01

1079	26881	aparc-Desikan_rh_thickness_rostralanteriorcingulate	OP	WR	1	1.34	0.29	0.59	6.22E-01	8.95E-01
1080	26882	aparc-Desikan_rh_thickness_rostralmiddlefrontal	OP	IVW	4	0.69	-0.37	0.28	1.92E-01	8.95E-01
1082	26884	aparc-Desikan_rh_thickness_superiorparietal	OP	IVW	7	1.25	0.22	0.28	4.28E-01	8.95E-01
1085	26887	aparc-Desikan_rh_thickness_frontalpole	OP	WR	1	0.59	-0.53	0.51	3.01E-01	8.95E-01
1086	26888	aparc-Desikan_rh_thickness_transversetemporal	OP	WR	1	2.34	0.85	1.21	4.84E-01	8.95E-01
1087	26889	aparc-Desikan_rh_thickness_insula	OP	IVW	4	1.26	0.23	0.32	4.67E-01	8.95E-01
1118	27176	aparc-DKTatlas_lh_thickness_cuneus	OP	IVW	3	1.77	0.57	0.36	1.13E-01	8.95E-01
1120	27178	aparc-DKTatlas_lh_thickness_fusiform	OP	IVW	2	1.27	0.24	0.35	5.01E-01	8.95E-01
1123	27181	aparc-DKTatlas_lh_thickness_isthmuscingulate	OP	IVW	3	1.43	0.36	0.39	3.55E-01	8.95E-01
1125	27183	aparc-DKTatlas_lh_thickness_lateralorbitofrontal	OP	IVW	2	1.32	0.28	0.51	5.87E-01	8.95E-01
1126	27184	aparc-DKTatlas_lh_thickness_lingual	OP	IVW	4	0.69	-0.37	0.32	2.47E-01	8.95E-01
1128	27186	aparc-DKTatlas_lh_thickness_middletemporal	OP	WR	1	0.42	-0.86	1.01	3.91E-01	8.95E-01
1130	27188	aparc-DKTatlas_lh_thickness_paracentral	OP	IVW	6	1.30	0.26	0.29	3.80E-01	8.95E-01
1131	27189	aparc-DKTatlas_lh_thickness_parsopercularis	OP	IVW	5	1.11	0.1	0.25	6.97E-01	8.95E-01
1132	27190	aparc-DKTatlas_lh_thickness_parsorbitalis	OP	WR	1	1.49	0.4	0.48	4.02E-01	8.95E-01
1133	27191	aparc-DKTatlas_lh_thickness_parstriangularis	OP	WR	1	0.78	-0.25	0.42	5.49E-01	8.95E-01
1135	27193	aparc-DKTatlas_lh_thickness_postcentral	OP	IVW	5	1.32	0.28	0.24	2.46E-01	8.95E-01
1136	27194	aparc-DKTatlas_lh_thickness_posteriorcingulate	OP	IVW	2	0.57	-0.57	0.50	2.60E-01	8.95E-01
1137	27195	aparc-DKTatlas_lh_thickness_precentral	OP	IVW	3	0.80	-0.22	0.33	5.05E-01	8.95E-01
1138	27196	aparc-DKTatlas_lh_thickness_precuneus	OP	IVW	7	1.32	0.28	0.24	2.48E-01	8.95E-01
1139	27197	aparc-DKTatlas_lh_thickness_rostralanteriorcingulate	OP	WR	1	1.57	0.45	0.59	4.48E-01	8.95E-01
1140	27198	aparc-DKTatlas_lh_thickness_rostralmiddlefrontal	OP	IVW	2	0.71	-0.34	0.42	4.26E-01	8.95E-01
1141	27199	aparc-DKTatlas_lh_thickness_superiorfrontal	OP	IVW	8	0.83	-0.19	0.21	3.60E-01	8.95E-01
1143	27201	aparc-DKTatlas_lh_thickness_superiortemporal	OP	IVW	2	1.36	0.31	0.52	5.51E-01	8.95E-01
1144	27202	aparc-DKTatlas_lh_thickness_supramarginal	OP	IVW	4	0.78	-0.25	0.32	4.34E-01	8.95E-01
1145	27203	aparc-DKTatlas_lh_thickness_transversetemporal	OP	IVW	3	1.82	0.6	0.37	1.02E-01	8.95E-01
1146	27204	aparc-DKTatlas_lh_thickness_insula	OP	IVW	4	0.76	-0.28	0.50	5.78E-01	8.95E-01
1147	27267	aparc-DKTatlas_rh_thickness_caudalanteriorcingulate	OP	IVW	3	1.60	0.47	0.43	2.77E-01	8.95E-01
1148	27268	aparc-DKTatlas_rh_thickness_caudalmiddlefrontal	OP	IVW	2	0.84	-0.17	0.41	6.85E-01	8.95E-01
1149	27269	aparc-DKTatlas_rh_thickness_cuneus	OP	IVW	7	1.32	0.28	0.21	1.91E-01	8.95E-01
1152	27272	aparc-DKTatlas_rh_thickness_inferiorparietal	OP	IVW	4	0.89	-0.12	0.31	6.90E-01	8.95E-01
1153	27273	aparc-DKTatlas_rh_thickness_inferiortemporal	OP	IVW	6	1.12	0.11	0.28	6.87E-01	8.95E-01
1154	27274	aparc-DKTatlas_rh_thickness_isthmuscingulate	OP	IVW	5	1.55	0.44	0.30	1.46E-01	8.95E-01

1155	27275	aparc-DKTatlas_rh_thickness_lateraloccipital	OP	IVW	10	1.35	0.3	0.18	9.42E-02	8.95E-01
1156	27276	aparc-DKTatlas_rh_thickness_lateralorbitofrontal	OP	IVW	5	0.67	-0.4	0.32	2.15E-01	8.95E-01
1157	27277	aparc-DKTatlas_rh_thickness_lingual	OP	IVW	5	0.57	-0.56	0.28	4.26E-02	8.95E-01
1158	27278	aparc-DKTatlas_rh_thickness_medialorbitofrontal	OP	IVW	3	1.67	0.51	0.36	1.51E-01	8.95E-01
1159	27279	aparc-DKTatlas_rh_thickness_middletemporal	OP	IVW	3	1.17	0.16	0.40	6.79E-01	8.95E-01
1162	27282	aparc-DKTatlas_rh_thickness_parsopercularis	OP	IVW	2	1.19	0.17	0.33	6.16E-01	8.95E-01
1163	27283	aparc-DKTatlas_rh_thickness_parsorbitalis	OP	WR	1	0.87	-0.14	0.32	6.61E-01	8.95E-01
1164	27284	aparc-DKTatlas_rh_thickness_parstriangularis	OP	IVW	2	0.54	-0.61	0.40	1.27E-01	8.95E-01
1165	27285	aparc-DKTatlas_rh_thickness_pericalcarine	OP	IVW	2	0.73	-0.32	0.51	5.34E-01	8.95E-01
1166	27286	aparc-DKTatlas_rh_thickness_postcentral	OP	IVW	4	1.12	0.11	0.28	6.90E-01	8.95E-01
1168	27288	aparc-DKTatlas_rh_thickness_precentral	OP	IVW	7	0.85	-0.16	0.27	5.62E-01	8.95E-01
1169	27289	aparc-DKTatlas_rh_thickness_precuneus	OP	IVW	9	1.27	0.24	0.19	2.04E-01	8.95E-01
1170	27290	aparc-DKTatlas_rh_thickness_rostralanteriorcingulate	OP	IVW	2	0.84	-0.18	0.47	7.03E-01	8.95E-01
1171	27291	aparc-DKTatlas_rh_thickness_rostralmiddlefrontal	OP	IVW	2	0.65	-0.43	0.55	4.33E-01	8.95E-01
1172	27292	aparc-DKTatlas_rh_thickness_superiorfrontal	OP	IVW	5	0.87	-0.14	0.26	5.90E-01	8.95E-01
1173	27293	aparc-DKTatlas_rh_thickness_superiorparietal	OP	IVW	8	1.26	0.23	0.28	4.13E-01	8.95E-01
1174	27294	aparc-DKTatlas_rh_thickness_superiortemporal	OP	IVW	2	0.76	-0.28	0.38	4.74E-01	8.95E-01
1175	27295	aparc-DKTatlas_rh_thickness_supramarginal	OP	IVW	5	0.62	-0.48	0.30	1.19E-01	8.95E-01
1176	27296	aparc-DKTatlas_rh_thickness_transversetemporal	OP	WR	1	2.34	0.85	1.22	4.84E-01	8.95E-01
1177	27297	aparc-DKTatlas_rh_thickness_insula	OP	IVW	3	0.76	-0.27	0.67	6.93E-01	8.95E-01
1178	27403	aparc-a2009s_lh_thickness_G+S-frontomargin	OP	IVW	2	0.59	-0.53	0.52	3.06E-01	8.95E-01
1179	27404	aparc-a2009s_lh_thickness_G+S-occipital-inf	OP	IVW	3	1.77	0.57	0.37	1.30E-01	8.95E-01
1180	27405	aparc-a2009s_lh_thickness_G+S-paracentral	OP	IVW	4	1.57	0.45	0.28	1.17E-01	8.95E-01
1182	27407	aparc-a2009s_lh_thickness_G+S-transv-frontopol	OP	WR	1	0.51	-0.68	0.54	2.12E-01	8.95E-01
1183	27408	aparc-a2009s_lh_thickness_G+S-cingul-Ant	OP	WR	1	0.43	-0.85	1.00	3.91E-01	8.95E-01
601	27726	aparc-a2009s_rh_volume_G-postcentral	OP	IVW	5	1.26	0.23	0.27	4.02E-01	9.02E-01
30	25786	IDP_T1_FAST_ROIs_L_sup_front_gyrus	OP	WR	1	0.69	-0.37	0.48	4.40E-01	9.03E-01
30	25786	IDP_T1_FAST_ROIs_L_sup_front_gyrus	OP	WR	1	0.69	-0.37	0.48	4.40E-01	9.03E-01
78	25834	IDP_T1_FAST_ROIs_L_subcallosal_cortex	OP	IVW	10	0.82	-0.2	0.26	4.48E-01	9.03E-01
117	25873	IDP_T1_FAST_ROIs_R_planum_temporale	OP	IVW	3	1.36	0.31	0.40	4.34E-01	9.03E-01
952	27557	aparc-a2009s_rh_area_G+S-cingul-Mid-Ant	OP	IVW	3	0.64	-0.45	0.39	2.43E-01	9.04E-01
191	26554	aseg_lh_volume_Lateral-Ventricle	OP	IVW	13	1.39	0.33	0.16	3.76E-02	9.05E-01
194	26557	aseg_lh_volume_Cerebellum-Cortex	OP	IVW	24	1.23	0.21	0.12	7.98E-02	9.05E-01

211	26588	aseg_rh_volume_Cerebellum-Cortex	OP	IVW	26	1.23	0.21	0.11	6.51E-02	9.05E-01
1210	27435	aparc-a2009s_lh_thickness_G-temp-sup-G-T-transv	OP	IVW	2	2.32	0.84	0.52	1.08E-01	9.09E-01
188	26537	aseg_global_volume-ratio_MaskVol-to-eTIV	OP	IVW	3	1.28	0.25	0.84	7.64E-01	9.10E-01
189	26552	aseg_lh_volume_Cortex	OP	IVW	5	1.23	0.21	0.36	5.59E-01	9.10E-01
190	26553	aseg_lh_volume_CerebralWhiteMatter	OP	IVW	18	1.14	0.13	0.15	3.88E-01	9.10E-01
192	26555	aseg_lh_volume_Inf-Lat-Vent	OP	IVW	6	1.08	0.08	0.23	7.20E-01	9.10E-01
193	26556	aseg_lh_volume_Cerebellum-White-Matter	OP	IVW	21	0.92	-0.08	0.12	4.93E-01	9.10E-01
195	26558	aseg_lh_volume_Thalamus-Proper	OP	IVW	8	0.84	-0.17	0.19	3.91E-01	9.10E-01
196	26559	aseg_lh_volume_Caudate	OP	IVW	17	1.06	0.06	0.14	6.68E-01	9.10E-01
197	26560	aseg_lh_volume_Putamen	OP	IVW	21	0.94	-0.06	0.12	6.17E-01	9.10E-01
198	26561	aseg_lh_volume_Pallidum	OP	IVW	14	0.92	-0.08	0.18	6.35E-01	9.10E-01
199	26562	aseg_lh_volume_Hippocampus	OP	IVW	12	0.90	-0.11	0.17	4.99E-01	9.10E-01
200	26563	aseg_lh_volume_Amygdala	OP	IVW	10	0.92	-0.08	0.18	6.55E-01	9.10E-01
201	26564	aseg_lh_volume_Accumbens-area	OP	IVW	6	1.04	0.04	0.24	8.81E-01	9.10E-01
202	26565	aseg_lh_volume_VentralDC	OP	IVW	17	0.95	-0.05	0.12	6.93E-01	9.10E-01
204	26567	aseg_lh_volume_choroid-plexus	OP	IVW	9	1.03	0.03	0.19	8.83E-01	9.10E-01
206	26583	aseg_rh_volume_Cortex	OP	IVW	4	1.17	0.16	0.33	6.18E-01	9.10E-01
207	26584	aseg_rh_volume_CerebralWhiteMatter	OP	IVW	18	1.20	0.18	0.14	1.97E-01	9.10E-01
208	26585	aseg_rh_volume_Lateral-Ventricle	OP	IVW	12	1.12	0.11	0.17	5.25E-01	9.10E-01
209	26586	aseg_rh_volume_Inf-Lat-Vent	OP	IVW	2	0.93	-0.07	0.42	8.65E-01	9.10E-01
210	26587	aseg_rh_volume_Cerebellum-White-Matter	OP	IVW	13	1.13	0.12	0.13	3.63E-01	9.10E-01
212	26589	aseg_rh_volume_Thalamus-Proper	OP	IVW	9	0.84	-0.18	0.18	3.04E-01	9.10E-01
213	26590	aseg_rh_volume_Caudate	OP	IVW	18	1.21	0.19	0.13	1.48E-01	9.10E-01
214	26591	aseg_rh_volume_Putamen	OP	IVW	22	1.08	0.08	0.12	5.34E-01	9.10E-01
215	26592	aseg_rh_volume_Pallidum	OP	IVW	12	0.88	-0.13	0.17	4.46E-01	9.10E-01
216	26593	aseg_rh_volume_Hippocampus	OP	IVW	12	0.84	-0.17	0.16	2.71E-01	9.10E-01
217	26594	aseg_rh_volume_Amygdala	OP	IVW	7	0.82	-0.2	0.24	4.07E-01	9.10E-01
218	26595	aseg_rh_volume_Accumbens-area	OP	IVW	7	0.93	-0.07	0.26	8.03E-01	9.10E-01
219	26596	aseg_rh_volume_VentralDC	OP	IVW	18	0.96	-0.04	0.12	7.61E-01	9.10E-01
221	26598	aseg_rh_volume_choroid-plexus	OP	IVW	9	1.12	0.11	0.19	5.86E-01	9.10E-01
222	26599	aseg_rh_number_HolesBeforeFixing	OP	IVW	6	0.95	-0.05	0.22	8.38E-01	9.10E-01
223	26600	AmygNuclei_lh_volume_Lateral-nucleus	OP	IVW	10	0.82	-0.2	0.19	2.81E-01	9.10E-01
503	27480	aparc-a2009s_lh_volume_G+S-subcentral	OP	IVW	2	0.78	-0.25	0.41	5.48E-01	9.12E-01

513	27490	aparc-a2009s_lh_volume_G-front-inf-Triangul	OP	IVW	3	1.28	0.25	0.40	5.22E-01	9.12E-01
529	27506	aparc-a2009s_lh_volume_G-precuneus	OP	IVW	11	1.23	0.21	0.28	4.45E-01	9.12E-01
532	27509	aparc-a2009s_lh_volume_G-temp-sup-G-T-transv	OP	WR	1	0.65	-0.43	0.59	4.60E-01	9.12E-01
541	27518	aparc-a2009s_lh_volume_Pole-occipital	OP	IVW	2	1.65	0.5	0.79	5.22E-01	9.12E-01
544	27521	aparc-a2009s_lh_volume_S-central	OP	IVW	5	0.89	-0.12	0.21	5.46E-01	9.12E-01
548	27525	aparc-a2009s_lh_volume_S-circular-insula-sup	OP	IVW	4	1.20	0.18	0.30	5.46E-01	9.12E-01
560	27537	aparc-a2009s_lh_volume_S-oc-temp-med+Lingual	OP	IVW	4	1.25	0.22	0.38	5.58E-01	9.12E-01
568	27545	aparc-a2009s_lh_volume_S-precentral-sup-part	OP	IVW	2	0.76	-0.28	0.36	4.35E-01	9.12E-01
570	27547	aparc-a2009s_lh_volume_S-subparietal	OP	IVW	7	0.88	-0.13	0.23	5.67E-01	9.12E-01
588	27713	aparc-a2009s_rh_volume_G-front-middle	OP	IVW	3	1.40	0.34	0.43	4.38E-01	9.12E-01
591	27716	aparc-a2009s_rh_volume_G-insular-short	OP	WR	1	0.66	-0.41	0.56	4.65E-01	9.12E-01
602	27727	aparc-a2009s_rh_volume_G-precentral	OP	IVW	4	0.82	-0.2	0.28	4.85E-01	9.12E-01
609	27734	aparc-a2009s_rh_volume_G-temp-sup-Plan-tempo	OP	IVW	2	0.54	-0.62	1.00	5.33E-01	9.12E-01
611	27736	aparc-a2009s_rh_volume_G-temporal-middle	OP	IVW	5	0.79	-0.24	0.40	5.54E-01	9.12E-01
614	27739	aparc-a2009s_rh_volume_Lat-Fis-post	OP	WR	1	0.69	-0.37	0.62	5.51E-01	9.12E-01
615	27740	aparc-a2009s_rh_volume_Pole-occipital	OP	IVW	7	1.17	0.16	0.24	5.08E-01	9.12E-01
618	27743	aparc-a2009s_rh_volume_S-central	OP	IVW	6	0.89	-0.12	0.19	5.18E-01	9.12E-01
632	27757	aparc-a2009s_rh_volume_S-occipital-ant	OP	IVW	2	1.63	0.49	0.70	4.81E-01	9.12E-01
633	27758	aparc-a2009s_rh_volume_S-oc-temp-lat	OP	WR	1	1.51	0.41	0.71	5.66E-01	9.12E-01
634	27759	aparc-a2009s_rh_volume_S-oc-temp-med+Lingual	OP	IVW	3	1.26	0.23	0.34	4.89E-01	9.12E-01
507	27484	aparc-a2009s_lh_volume_G+S-cingul-Mid-Post	OP	WR	1	1.34	0.29	0.52	5.79E-01	9.13E-01
535	27512	aparc-a2009s_lh_volume_G-temp-sup-Plan-tempo	OP	WR	1	1.39	0.33	0.61	5.90E-01	9.13E-01
638	27763	aparc-a2009s_rh_volume_S-parieto-occipital	OP	IVW	10	0.87	-0.14	0.25	5.89E-01	9.13E-01
175	26524	aseg_global_volume_4th-Ventricle	OP	IVW	20	0.95	-0.05	0.14	7.06E-01	9.13E-01
1088	27073	BA-exvivo_lh_thickness_BA1	OP	IVW	5	1.19	0.17	0.26	5.15E-01	9.15E-01
1089	27074	BA-exvivo_lh_thickness_BA2	OP	IVW	2	1.84	0.61	0.45	1.78E-01	9.15E-01
1090	27075	BA-exvivo_lh_thickness_BA3a	OP	IVW	4	1.31	0.27	0.39	4.81E-01	9.15E-01
1091	27076	BA-exvivo_lh_thickness_BA3b	OP	IVW	2	1.13	0.12	0.39	7.66E-01	9.15E-01
1092	27077	BA-exvivo_lh_thickness_BA4a	OP	IVW	4	1.40	0.34	0.28	2.34E-01	9.15E-01
1094	27079	BA-exvivo_lh_thickness_BA6	OP	IVW	10	1.12	0.11	0.17	5.16E-01	9.15E-01
1095	27080	BA-exvivo_lh_thickness_BA44	OP	IVW	5	1.08	0.08	0.24	7.27E-01	9.15E-01
1096	27081	BA-exvivo_lh_thickness_BA45	OP	IVW	3	0.64	-0.45	0.37	2.19E-01	9.15E-01
1097	27082	BA-exvivo_lh_thickness_V1	OP	WR	1	2.32	0.84	1.20	4.84E-01	9.15E-01

1099	27084	BA-exvivo_lh_thickness_MT	OP	IVW	5	1.43	0.36	0.32	2.67E-01	9.15E-01
1101	27086	BA-exvivo_lh_thickness_entorhinal	OP	WR	1	0.77	-0.26	0.55	6.34E-01	9.15E-01
1102	27115	BA-exvivo_rh_thickness_BA1	OP	WR	1	0.63	-0.46	0.63	4.65E-01	9.15E-01
1103	27116	BA-exvivo_rh_thickness_BA2	OP	IVW	2	1.06	0.06	0.32	8.59E-01	9.15E-01
1104	27117	BA-exvivo_rh_thickness_BA3a	OP	WR	1	2.01	0.7	1.25	5.74E-01	9.15E-01
1105	27118	BA-exvivo_rh_thickness_BA3b	OP	IVW	2	1.13	0.12	0.79	8.78E-01	9.15E-01
1107	27120	BA-exvivo_rh_thickness_BA4p	OP	IVW	3	0.92	-0.08	0.41	8.49E-01	9.15E-01
1108	27121	BA-exvivo_rh_thickness_BA6	OP	IVW	7	0.88	-0.13	0.24	5.85E-01	9.15E-01
1109	27122	BA-exvivo_rh_thickness_BA44	OP	IVW	4	1.04	0.04	0.26	8.79E-01	9.15E-01
1110	27123	BA-exvivo_rh_thickness_BA45	OP	IVW	3	0.88	-0.13	0.47	7.86E-01	9.15E-01
1111	27124	BA-exvivo_rh_thickness_V1	OP	IVW	3	0.92	-0.08	0.35	8.19E-01	9.15E-01
1112	27125	BA-exvivo_rh_thickness_V2	OP	IVW	9	0.94	-0.06	0.20	7.58E-01	9.15E-01
1113	27126	BA-exvivo_rh_thickness_MT	OP	IVW	2	1.35	0.3	0.50	5.50E-01	9.15E-01
1115	27128	BA-exvivo_rh_thickness_entorhinal	OP	IVW	2	0.81	-0.21	0.45	6.45E-01	9.15E-01
224	26601	AmygNuclei_lh_volume_Basal-nucleus	OP	IVW	9	0.76	-0.28	0.19	1.42E-01	9.15E-01
226	26603	AmygNuclei_lh_volume_Anterior-amygdaloid-area-AAA	OP	IVW	5	0.82	-0.2	0.26	4.32E-01	9.15E-01
230	26607	AmygNuclei_lh_volume_Corticoamygdaloid-transitio	OP	IVW	4	1.34	0.29	0.27	2.77E-01	9.15E-01
232	26609	AmygNuclei_lh_volume_Whole-amygdala	OP	IVW	10	0.79	-0.24	0.18	1.93E-01	9.15E-01
233	26610	AmygNuclei_rh_volume_Lateral-nucleus	OP	IVW	12	0.77	-0.26	0.16	1.10E-01	9.15E-01
238	26615	AmygNuclei_rh_volume_Medial-nucleus	OP	IVW	6	0.76	-0.28	0.33	3.94E-01	9.15E-01
239	26616	AmygNuclei_rh_volume_Cortical-nucleus	OP	IVW	3	0.58	-0.54	0.56	3.35E-01	9.15E-01
241	26618	AmygNuclei_rh_volume_Paralaminar-nucleus	OP	IVW	6	0.79	-0.23	0.24	3.46E-01	9.15E-01
242	26619	AmygNuclei_rh_volume_Whole-amygdala	OP	IVW	9	0.86	-0.15	0.19	4.34E-01	9.15E-01
165	26514	aseg_global_volume_BrainSeg	OP	IVW	13	1.03	0.03	0.16	8.35E-01	9.18E-01
168	26517	aseg_global_volume_SubCortGray	OP	IVW	12	0.96	-0.04	0.16	8.12E-01	9.18E-01
181	26530	aseg_global_volume_Optic-Chiasm	OP	IVW	4	0.90	-0.1	0.33	7.62E-01	9.18E-01
1230	27455	aparc-a2009s_lh_thickness_S-front-middle	OP	IVW	2	1.60	0.47	0.32	1.43E-01	9.25E-01
1266	27639	aparc-a2009s_rh_thickness_G-front-middle	OP	IVW	5	0.63	-0.46	0.30	1.24E-01	9.25E-01
1278	27651	aparc-a2009s_rh_thickness_G-parietal-sup	OP	IVW	3	0.61	-0.5	0.35	1.50E-01	9.25E-01
1296	27669	aparc-a2009s_rh_thickness_S-central	OP	IVW	4	0.66	-0.41	0.29	1.53E-01	9.25E-01
1307	27680	aparc-a2009s_rh_thickness_S-intrapariet+P-trans	OP	IVW	6	1.52	0.42	0.28	1.41E-01	9.25E-01
796	27101	BA-exvivo_rh_area_BA1	OP	IVW	7	0.83	-0.19	0.22	3.97E-01	9.25E-01
799	27104	BA-exvivo_rh_area_BA3b	OP	IVW	8	0.79	-0.23	0.29	4.30E-01	9.26E-01

423	27100	BA-exvivo_lh_volume_entorhinal	OP	IVW	6	0.90	-0.1	0.24	6.88E-01	9.29E-01
247	26624	HippSubfield_lh_volume_hippocampal-fissure	OP	IVW	3	0.64	-0.44	0.41	2.79E-01	9.29E-01
248	26625	HippSubfield_lh_volume_presubiculum-head	OP	IVW	4	1.36	0.31	0.30	3.03E-01	9.29E-01
262	26639	HippSubfield_lh_volume_Whole-hippocampal-body	OP	IVW	15	0.82	-0.2	0.18	2.83E-01	9.29E-01
280	26657	HippSubfield_rh_volume_CA4-body	OP	IVW	11	0.83	-0.19	0.17	2.79E-01	9.29E-01
286	26663	HippSubfield_rh_volume_Whole-hippocampus	OP	IVW	21	0.88	-0.13	0.13	2.85E-01	9.29E-01
183	26532	aseg_global_volume_CC-Mid-Posterior	OP	IVW	9	0.97	-0.03	0.21	8.87E-01	9.30E-01
547	27524	aparc-a2009s_lh_volume_S-circular-insula-inf	OP	IVW	3	1.27	0.24	0.47	6.17E-01	9.31E-01
563	27540	aparc-a2009s_lh_volume_S-orbital-H-Shaped	OP	IVW	8	0.90	-0.11	0.23	6.21E-01	9.31E-01
640	27765	aparc-a2009s_rh_volume_S-postcentral	OP	WR	1	1.40	0.34	0.69	6.26E-01	9.31E-01
1033	26768	aparc-Desikan_lh_thickness_medialorbitofrontal	OP	WR	1	0.76	-0.28	0.90	7.58E-01	9.31E-01
1161	27281	aparc-DKTatlas_rh_thickness_paracentral	OP	IVW	3	1.13	0.12	0.37	7.53E-01	9.31E-01
1181	27406	aparc-a2009s_lh_thickness_G+S-subcentral	OP	IVW	6	0.92	-0.08	0.26	7.50E-01	9.31E-01
1076	26878	aparc-Desikan_rh_thickness_posteriorcingulate	OP	IVW	2	1.12	0.11	0.38	7.73E-01	9.31E-01
1083	26885	aparc-Desikan_rh_thickness_superiortemporal	OP	WR	1	0.86	-0.15	0.49	7.67E-01	9.31E-01
38	25794	IDP_T1_FAST_ROIs_L_precentral_gyrus	OP	IVW	2	1.36	0.31	0.44	4.80E-01	9.32E-01
74	25830	IDP_T1_FAST_ROIs_L_front_med_cortex	OP	IVW	2	0.70	-0.36	0.53	4.95E-01	9.32E-01
115	25871	IDP_T1_FAST_ROIs_R_heschl_gyrus	OP	IVW	3	1.34	0.29	0.41	4.85E-01	9.32E-01
123	25879	IDP_T1_FAST_ROIs_R_thalamus	OP	IVW	14	1.11	0.1	0.15	4.97E-01	9.32E-01
157	25913	IDP_T1_FAST_ROIs_V_cerebellum_VIIIb	OP	IVW	18	1.09	0.09	0.13	4.78E-01	9.32E-01
418	27095	BA-exvivo_lh_volume_BA45	OP	IVW	5	0.92	-0.08	0.25	7.44E-01	9.36E-01
432	27137	BA-exvivo_rh_volume_BA45	OP	IVW	5	1.07	0.07	0.27	7.97E-01	9.36E-01
437	27142	BA-exvivo_rh_volume_entorhinal	OP	IVW	7	1.07	0.07	0.24	7.63E-01	9.36E-01
205	26568	aseg_lh_number_HolesBeforeFixing	OP	IVW	8	0.98	-0.02	0.21	9.36E-01	9.36E-01
691	26831	aparc-Desikan_rh_area_isthmuscingulate	OP	IVW	4	1.86	0.62	0.27	2.18E-02	9.37E-01
720	26927	aparc-pial_lh_area_cuneus	OP	IVW	22	1.32	0.28	0.13	2.50E-02	9.37E-01
755	26962	aparc-pial_rh_area_fusiform	OP	IVW	4	1.93	0.66	0.29	2.46E-02	9.37E-01
759	26966	aparc-pial_rh_area_lateraloccipital	OP	IVW	14	1.48	0.39	0.16	1.58E-02	9.37E-01
225	26602	AmygNuclei_lh_volume_Accessory-Basal-nucleus	OP	IVW	3	1.07	0.07	0.35	8.43E-01	9.38E-01
227	26604	AmygNuclei_lh_volume_Central-nucleus	OP	IVW	3	0.80	-0.22	0.51	6.64E-01	9.38E-01
228	26605	AmygNuclei_lh_volume_Medial-nucleus	OP	IVW	2	1.52	0.42	1.07	6.95E-01	9.38E-01
229	26606	AmygNuclei_lh_volume_Cortical-nucleus	OP	IVW	2	1.16	0.15	0.49	7.60E-01	9.38E-01
231	26608	AmygNuclei_lh_volume_Paralaminar-nucleus	OP	IVW	6	0.92	-0.08	0.27	7.71E-01	9.38E-01

234	26611	AmygNuclei_rh_volume_Basal-nucleus	OP	IVW	7	0.97	-0.03	0.24	9.05E-01	9.38E-01
235	26612	AmygNuclei_rh_volume_Accessory-Basal-nucleus	OP	IVW	6	1.05	0.05	0.24	8.26E-01	9.38E-01
236	26613	AmygNuclei_rh_volume_Anterior-amygdaloid-area-AAA	OP	IVW	2	1.15	0.14	0.41	7.39E-01	9.38E-01
237	26614	AmygNuclei_rh_volume_Central-nucleus	OP	IVW	5	1.02	0.02	0.25	9.38E-01	9.38E-01
240	26617	AmygNuclei_rh_volume_Corticoamygdaloid-transitio	OP	IVW	3	0.90	-0.11	0.37	7.60E-01	9.38E-01
596	27721	aparc-a2009s_rh_volume_G-oc-temp-med-Parahip	OP	IVW	5	0.87	-0.14	0.29	6.39E-01	9.39E-01
873	27330	aparc-a2009s_lh_area_G+S-occipital-inf	OP	IVW	3	1.54	0.43	0.45	3.43E-01	9.42E-01
881	27338	aparc-a2009s_lh_area_G-cingul-Post-ventral	OP	IVW	8	1.22	0.2	0.24	3.87E-01	9.42E-01
893	27350	aparc-a2009s_lh_area_G-oc-temp-med-Lingual	OP	IVW	12	1.16	0.15	0.17	3.72E-01	9.42E-01
904	27361	aparc-a2009s_lh_area_G-temp-sup-G-T-transv	OP	IVW	3	0.59	-0.52	0.51	3.13E-01	9.42E-01
906	27363	aparc-a2009s_lh_area_G-temp-sup-Plan-polar	OP	WR	1	0.60	-0.51	0.55	3.47E-01	9.42E-01
911	27368	aparc-a2009s_lh_area_Lat-Fis-ant-Vertical	OP	IVW	4	1.67	0.51	0.49	2.92E-01	9.42E-01
922	27379	aparc-a2009s_lh_area_S-collat-transv-post	OP	WR	1	2.18	0.78	0.91	3.91E-01	9.42E-01
926	27383	aparc-a2009s_lh_area_S-interm-prim-Jensen	OP	WR	1	0.55	-0.59	0.62	3.41E-01	9.42E-01
928	27385	aparc-a2009s_lh_area_S-oc-middle+Lunatus	OP	IVW	6	0.78	-0.25	0.24	3.11E-01	9.42E-01
932	27389	aparc-a2009s_lh_area_S-oc-temp-med+Lingual	OP	IVW	3	1.38	0.32	0.34	3.60E-01	9.42E-01
942	27399	aparc-a2009s_lh_area_S-subparietal	OP	IVW	5	0.78	-0.25	0.22	2.70E-01	9.42E-01
954	27559	aparc-a2009s_rh_area_G-cingul-Post-dorsal	OP	WR	1	0.61	-0.5	0.58	3.89E-01	9.42E-01
955	27560	aparc-a2009s_rh_area_G-cingul-Post-ventral	OP	IVW	7	1.22	0.2	0.23	3.77E-01	9.42E-01
967	27572	aparc-a2009s_rh_area_G-oc-temp-med-Lingual	OP	IVW	12	1.20	0.18	0.17	3.02E-01	9.42E-01
979	27584	aparc-a2009s_rh_area_G-temp-sup-Lateral	OP	IVW	5	1.25	0.22	0.25	3.85E-01	9.42E-01
992	27597	aparc-a2009s_rh_area_S-circular-insula-ant	OP	IVW	4	1.67	0.51	0.49	2.97E-01	9.42E-01
997	27602	aparc-a2009s_rh_area_S-front-inf	OP	IVW	2	0.66	-0.42	0.48	3.83E-01	9.42E-01
998	27603	aparc-a2009s_rh_area_S-front-middle	OP	IVW	2	1.43	0.36	0.38	3.35E-01	9.42E-01
999	27604	aparc-a2009s_rh_area_S-front-sup	OP	WR	1	1.75	0.56	0.61	3.66E-01	9.42E-01
537	27514	aparc-a2009s_lh_volume_G-temporal-middle	OP	IVW	3	1.31	0.27	0.62	6.63E-01	9.42E-01
571	27548	aparc-a2009s_lh_volume_S-temporal-inf	OP	WR	1	1.31	0.27	0.63	6.65E-01	9.42E-01
594	27719	aparc-a2009s_rh_volume_G-oc-temp-lat-fusifor	OP	IVW	2	1.25	0.22	0.48	6.51E-01	9.42E-01
465	27232	aparc-DKTatlas_lh_volume_superiortemporal	OP	IVW	2	0.40	-0.91	0.60	1.27E-01	9.43E-01
499	27328	aparc-DKTatlas_rh_volume_insula	OP	IVW	9	1.34	0.29	0.19	1.21E-01	9.43E-01
1188	27413	aparc-a2009s_lh_thickness_G-cuneus	OP	IVW	5	1.51	0.41	0.30	1.74E-01	9.43E-01
1189	27414	aparc-a2009s_lh_thickness_G-front-inf-Opercular	OP	IVW	3	0.87	-0.14	0.36	7.00E-01	9.43E-01
1190	27415	aparc-a2009s_lh_thickness_G-front-inf-Orbital	OP	WR	1	1.79	0.58	0.49	2.36E-01	9.43E-01

1191	27416	aparc-a2009s_lh_thickness_G-front-inf-Triangul	OP	IVW	2	0.60	-0.51	0.46	2.64E-01	9.43E-01
1192	27417	aparc-a2009s_lh_thickness_G-front-middle	OP	IVW	5	0.84	-0.17	0.25	4.99E-01	9.43E-01
1194	27419	aparc-a2009s_lh_thickness_G-Ins-lg+S-cent-ins	OP	WR	1	1.52	0.42	0.67	5.29E-01	9.43E-01
1196	27421	aparc-a2009s_lh_thickness_G-occipital-middle	OP	IVW	6	0.83	-0.19	0.27	4.79E-01	9.43E-01
1197	27422	aparc-a2009s_lh_thickness_G-occipital-sup	OP	IVW	4	1.22	0.2	0.32	5.29E-01	9.43E-01
1198	27423	aparc-a2009s_lh_thickness_G-oc-temp-lat-fusifor	OP	IVW	3	1.25	0.22	0.33	5.03E-01	9.43E-01
1199	27424	aparc-a2009s_lh_thickness_G-oc-temp-med-Lingual	OP	WR	1	1.28	0.25	1.07	8.16E-01	9.43E-01
1201	27426	aparc-a2009s_lh_thickness_G-orbital	OP	IVW	3	0.71	-0.34	0.44	4.44E-01	9.43E-01
1202	27427	aparc-a2009s_lh_thickness_G-pariet-inf-Angular	OP	IVW	10	1.05	0.05	0.22	8.39E-01	9.43E-01
1203	27428	aparc-a2009s_lh_thickness_G-pariet-inf-Supramar	OP	IVW	3	1.11	0.1	0.35	7.72E-01	9.43E-01
1205	27430	aparc-a2009s_lh_thickness_G-postcentral	OP	IVW	4	1.17	0.16	0.26	5.34E-01	9.43E-01
1206	27431	aparc-a2009s_lh_thickness_G-precentral	OP	IVW	4	0.75	-0.29	0.28	2.97E-01	9.43E-01
1207	27432	aparc-a2009s_lh_thickness_G-precuneus	OP	IVW	6	1.22	0.2	0.21	3.49E-01	9.43E-01
1208	27433	aparc-a2009s_lh_thickness_G-rectus	OP	WR	1	0.70	-0.36	0.64	5.81E-01	9.43E-01
1211	27436	aparc-a2009s_lh_thickness_G-temp-sup-Lateral	OP	IVW	3	0.90	-0.11	0.35	7.65E-01	9.43E-01
1212	27437	aparc-a2009s_lh_thickness_G-temp-sup-Plan-polar	OP	WR	1	0.54	-0.62	0.59	2.94E-01	9.43E-01
1214	27439	aparc-a2009s_lh_thickness_G-temporal-inf	OP	IVW	3	1.12	0.11	0.36	7.53E-01	9.43E-01
1218	27443	aparc-a2009s_lh_thickness_Lat-Fis-post	OP	IVW	4	1.26	0.23	0.29	4.27E-01	9.43E-01
1219	27444	aparc-a2009s_lh_thickness_Pole-occipital	OP	WR	1	1.55	0.44	0.57	4.35E-01	9.43E-01
1221	27446	aparc-a2009s_lh_thickness_S-calcarine	OP	WR	1	1.30	0.26	1.10	8.16E-01	9.43E-01
1222	27447	aparc-a2009s_lh_thickness_S-central	OP	IVW	4	1.21	0.19	0.34	5.78E-01	9.43E-01
1223	27448	aparc-a2009s_lh_thickness_S-cingul-Marginalis	OP	IVW	3	1.14	0.13	0.58	8.28E-01	9.43E-01
1225	27450	aparc-a2009s_lh_thickness_S-circular-insula-inf	OP	WR	1	0.25	-1.37	1.34	3.05E-01	9.43E-01
1227	27452	aparc-a2009s_lh_thickness_S-collat-transv-ant	OP	IVW	2	0.93	-0.07	0.38	8.48E-01	9.43E-01
1231	27456	aparc-a2009s_lh_thickness_S-front-sup	OP	IVW	4	0.77	-0.26	0.30	3.81E-01	9.43E-01
1234	27459	aparc-a2009s_lh_thickness_S-oc-middle+Lunatus	OP	WR	1	0.73	-0.31	1.26	8.03E-01	9.43E-01
1235	27460	aparc-a2009s_lh_thickness_S-oc-sup+transversal	OP	WR	1	0.73	-0.31	1.25	8.03E-01	9.43E-01
1238	27463	aparc-a2009s_lh_thickness_S-oc-temp-med+Lingual	OP	IVW	2	1.12	0.11	0.34	7.40E-01	9.43E-01
1240	27465	aparc-a2009s_lh_thickness_S-orbital-med-olfact	OP	IVW	2	0.70	-0.36	0.40	3.68E-01	9.43E-01
1241	27466	aparc-a2009s_lh_thickness_S-orbital-H-Shaped	OP	WR	1	0.58	-0.54	0.69	4.39E-01	9.43E-01
1243	27468	aparc-a2009s_lh_thickness_S-pericallosal	OP	IVW	3	1.28	0.25	0.33	4.53E-01	9.43E-01
1244	27469	aparc-a2009s_lh_thickness_S-postcentral	OP	IVW	5	1.62	0.48	0.40	2.33E-01	9.43E-01
1246	27471	aparc-a2009s_lh_thickness_S-precentral-sup-part	OP	IVW	2	0.92	-0.08	0.37	8.29E-01	9.43E-01

1247	27472	aparc-a2009s_lh_thickness_S-suborbital	OP	WR	1	0.35	-1.06	1.02	3.00E-01	9.43E-01
1249	27474	aparc-a2009s_lh_thickness_S-temporal-inf	OP	WR	1	0.76	-0.28	0.89	7.58E-01	9.43E-01
1250	27475	aparc-a2009s_lh_thickness_S-temporal-sup	OP	IVW	2	2.08	0.73	0.69	2.87E-01	9.43E-01
1252	27625	aparc-a2009s_rh_thickness_G+S-frontomargin	OP	WR	1	0.77	-0.26	0.86	7.58E-01	9.43E-01
1253	27626	aparc-a2009s_rh_thickness_G+S-occipital-inf	OP	IVW	4	1.40	0.34	0.32	2.90E-01	9.43E-01
1254	27627	aparc-a2009s_rh_thickness_G+S-paracentral	OP	WR	1	0.38	-0.97	1.21	4.22E-01	9.43E-01
1257	27630	aparc-a2009s_rh_thickness_G+S-cingul-Ant	OP	IVW	3	0.90	-0.11	0.35	7.55E-01	9.43E-01
1259	27632	aparc-a2009s_rh_thickness_G+S-cingul-Mid-Post	OP	WR	1	1.34	0.29	0.58	6.22E-01	9.43E-01
1260	27633	aparc-a2009s_rh_thickness_G-cingul-Post-dorsal	OP	IVW	2	0.69	-0.37	0.42	3.70E-01	9.43E-01
1261	27634	aparc-a2009s_rh_thickness_G-cingul-Post-ventral	OP	IVW	2	1.52	0.42	0.64	5.07E-01	9.43E-01
1264	27637	aparc-a2009s_rh_thickness_G-front-inf-Orbital	OP	WR	1	0.68	-0.39	0.57	4.90E-01	9.43E-01
1267	27640	aparc-a2009s_rh_thickness_G-front-sup	OP	IVW	5	0.79	-0.23	0.24	3.54E-01	9.43E-01
1270	27643	aparc-a2009s_rh_thickness_G-occipital-middle	OP	IVW	5	0.92	-0.08	0.31	7.99E-01	9.43E-01
1273	27646	aparc-a2009s_rh_thickness_G-oc-temp-med-Lingual	OP	IVW	4	0.69	-0.37	0.40	3.61E-01	9.43E-01
1274	27647	aparc-a2009s_rh_thickness_G-oc-temp-med-Parahip	OP	WR	1	1.21	0.19	0.77	8.03E-01	9.43E-01
1275	27648	aparc-a2009s_rh_thickness_G-orbital	OP	IVW	3	0.73	-0.32	0.62	6.08E-01	9.43E-01
1276	27649	aparc-a2009s_rh_thickness_G-pariet-inf-Angular	OP	IVW	4	1.13	0.12	0.25	6.33E-01	9.43E-01
1277	27650	aparc-a2009s_rh_thickness_G-pariet-inf-Supramar	OP	IVW	2	0.54	-0.62	0.88	4.82E-01	9.43E-01
1279	27652	aparc-a2009s_rh_thickness_G-postcentral	OP	IVW	6	1.09	0.09	0.28	7.44E-01	9.43E-01
1280	27653	aparc-a2009s_rh_thickness_G-precentral	OP	IVW	4	1.16	0.15	0.76	8.40E-01	9.43E-01
1281	27654	aparc-a2009s_rh_thickness_G-precuneus	OP	IVW	8	1.22	0.2	0.19	3.06E-01	9.43E-01
1284	27657	aparc-a2009s_rh_thickness_G-temp-sup-G-T-transv	OP	IVW	2	1.48	0.39	0.62	5.34E-01	9.43E-01
1285	27658	aparc-a2009s_rh_thickness_G-temp-sup-Lateral	OP	WR	1	0.81	-0.21	0.63	7.35E-01	9.43E-01
1288	27661	aparc-a2009s_rh_thickness_G-temporal-inf	OP	IVW	4	1.42	0.35	0.33	2.84E-01	9.43E-01
1289	27662	aparc-a2009s_rh_thickness_G-temporal-middle	OP	IVW	4	1.07	0.07	0.31	8.32E-01	9.43E-01
1290	27663	aparc-a2009s_rh_thickness_Lat-Fis-ant-Horizont	OP	WR	1	0.19	-1.67	1.81	3.55E-01	9.43E-01
1292	27665	aparc-a2009s_rh_thickness_Lat-Fis-post	OP	IVW	2	1.22	0.2	0.42	6.30E-01	9.43E-01
1293	27666	aparc-a2009s_rh_thickness_Pole-occipital	OP	WR	1	2.41	0.88	1.15	4.44E-01	9.43E-01
1295	27668	aparc-a2009s_rh_thickness_S-calcarine	OP	IVW	4	0.90	-0.1	0.45	8.22E-01	9.43E-01
1299	27672	aparc-a2009s_rh_thickness_S-circular-insula-inf	OP	WR	1	1.34	0.29	0.66	6.66E-01	9.43E-01
1300	27673	aparc-a2009s_rh_thickness_S-circular-insula-sup	OP	IVW	2	0.66	-0.42	1.09	7.03E-01	9.43E-01
1301	27674	aparc-a2009s_rh_thickness_S-collat-transv-ant	OP	WR	1	0.44	-0.83	0.97	3.91E-01	9.43E-01
1302	27675	aparc-a2009s_rh_thickness_S-collat-transv-post	OP	WR	1	2.89	1.06	1.39	4.44E-01	9.43E-01

1304	27677	aparc-a2009s_rh_thickness_S-front-middle	OP	IVW	3	0.92	-0.08	0.42	8.40E-01	9.43E-01
1305	27678	aparc-a2009s_rh_thickness_S-front-sup	OP	IVW	3	0.89	-0.12	0.53	8.15E-01	9.43E-01
1308	27681	aparc-a2009s_rh_thickness_S-oc-middle+Lunatus	OP	IVW	2	1.86	0.62	0.52	2.32E-01	9.43E-01
1309	27682	aparc-a2009s_rh_thickness_S-oc-sup+transversal	OP	WR	1	1.27	0.24	1.03	8.16E-01	9.43E-01
1314	27687	aparc-a2009s_rh_thickness_S-orbital-med-olfact	OP	IVW	2	1.34	0.29	0.35	4.08E-01	9.43E-01
1316	27689	aparc-a2009s_rh_thickness_S-parieto-occipital	OP	WR	1	0.61	-0.5	0.58	3.86E-01	9.43E-01
1318	27691	aparc-a2009s_rh_thickness_S-postcentral	OP	IVW	4	1.22	0.2	0.51	7.00E-01	9.43E-01
1319	27692	aparc-a2009s_rh_thickness_S-precentral-inf-part	OP	IVW	2	1.32	0.28	0.53	5.89E-01	9.43E-01
1320	27693	aparc-a2009s_rh_thickness_S-precentral-sup-part	OP	IVW	2	1.70	0.53	1.22	6.64E-01	9.43E-01
1324	27697	aparc-a2009s_rh_thickness_S-temporal-sup	OP	WR	1	0.42	-0.87	1.02	3.91E-01	9.43E-01
1325	27698	aparc-a2009s_rh_thickness_S-temporal-transverse	OP	WR	1	1.51	0.41	0.61	5.01E-01	9.43E-01
782	27059	BA-exvivo_lh_area_BA1	OP	IVW	10	1.13	0.12	0.22	5.91E-01	9.44E-01
786	27063	BA-exvivo_lh_area_BA4a	OP	IVW	7	1.14	0.13	0.21	5.48E-01	9.44E-01
788	27065	BA-exvivo_lh_area_BA6	OP	IVW	7	0.88	-0.13	0.23	5.75E-01	9.44E-01
789	27066	BA-exvivo_lh_area_BA44	OP	WR	1	0.78	-0.25	0.59	6.76E-01	9.44E-01
790	27067	BA-exvivo_lh_area_BA45	OP	IVW	9	1.03	0.03	0.19	8.71E-01	9.44E-01
794	27071	BA-exvivo_lh_area_perirhinal	OP	IVW	3	0.92	-0.08	0.38	8.38E-01	9.44E-01
795	27072	BA-exvivo_lh_area_entorhinal	OP	IVW	8	1.04	0.04	0.21	8.33E-01	9.44E-01
800	27105	BA-exvivo_rh_area_BA4a	OP	IVW	4	0.84	-0.17	0.29	5.47E-01	9.44E-01
803	27108	BA-exvivo_rh_area_BA44	OP	IVW	3	1.06	0.06	0.38	8.76E-01	9.44E-01
804	27109	BA-exvivo_rh_area_BA45	OP	IVW	4	0.93	-0.07	0.28	7.99E-01	9.44E-01
807	27112	BA-exvivo_rh_area_MT	OP	IVW	8	0.90	-0.1	0.28	7.14E-01	9.44E-01
808	27113	BA-exvivo_rh_area_perirhinal	OP	IVW	4	0.94	-0.06	0.27	8.13E-01	9.44E-01
809	27114	BA-exvivo_rh_area_entorhinal	OP	IVW	4	0.92	-0.08	0.44	8.62E-01	9.44E-01
875	27332	aparc-a2009s_lh_area_G+S-subcentral	OP	IVW	4	0.74	-0.3	0.48	5.39E-01	9.46E-01
889	27346	aparc-a2009s_lh_area_G-insular-short	OP	IVW	3	0.76	-0.27	0.36	4.53E-01	9.46E-01
902	27359	aparc-a2009s_lh_area_G-rectus	OP	WR	1	0.69	-0.37	0.63	5.54E-01	9.46E-01
905	27362	aparc-a2009s_lh_area_G-temp-sup-Lateral	OP	WR	1	1.49	0.4	0.59	4.90E-01	9.46E-01
910	27367	aparc-a2009s_lh_area_Lat-Fis-ant-Horizont	OP	IVW	2	1.92	0.65	1.09	5.54E-01	9.46E-01
916	27373	aparc-a2009s_lh_area_S-central	OP	IVW	8	0.90	-0.11	0.15	4.80E-01	9.46E-01
918	27375	aparc-a2009s_lh_area_S-circular-insula-ant	OP	IVW	3	0.76	-0.28	0.34	4.03E-01	9.46E-01
930	27387	aparc-a2009s_lh_area_S-occipital-ant	OP	WR	1	0.72	-0.33	0.59	5.77E-01	9.46E-01
933	27390	aparc-a2009s_lh_area_S-orbital-lateral	OP	WR	1	0.66	-0.42	0.60	4.88E-01	9.46E-01

935	27392	aparc-a2009s_lh_area_S-orbital-H-Shaped	OP	IVW	9	0.90	-0.11	0.19	5.56E-01	9.46E-01
940	27397	aparc-a2009s_lh_area_S-precentral-sup-part	OP	WR	1	1.42	0.35	0.53	5.02E-01	9.46E-01
948	27553	aparc-a2009s_rh_area_G+S-paracentral	OP	IVW	4	0.87	-0.14	0.25	5.82E-01	9.46E-01
957	27562	aparc-a2009s_rh_area_G-front-inf-Opercular	OP	WR	1	1.38	0.32	0.47	4.99E-01	9.46E-01
958	27563	aparc-a2009s_rh_area_G-front-inf-Orbital	OP	IVW	3	1.72	0.54	0.78	4.88E-01	9.46E-01
959	27564	aparc-a2009s_rh_area_G-front-inf-Triangul	OP	IVW	4	1.52	0.42	0.51	4.12E-01	9.46E-01
961	27566	aparc-a2009s_rh_area_G-front-sup	OP	IVW	2	0.76	-0.27	0.46	5.57E-01	9.46E-01
964	27569	aparc-a2009s_rh_area_G-occipital-middle	OP	IVW	6	0.83	-0.19	0.35	5.81E-01	9.46E-01
968	27573	aparc-a2009s_rh_area_G-oc-temp-med-Parahip	OP	IVW	3	0.82	-0.2	0.33	5.35E-01	9.46E-01
969	27574	aparc-a2009s_rh_area_G-orbital	OP	IVW	3	1.28	0.25	0.34	4.68E-01	9.46E-01
975	27580	aparc-a2009s_rh_area_G-precuneus	OP	IVW	8	0.87	-0.14	0.20	4.96E-01	9.46E-01
976	27581	aparc-a2009s_rh_area_G-rectus	OP	IVW	3	1.22	0.2	0.33	5.42E-01	9.46E-01
981	27586	aparc-a2009s_rh_area_G-temp-sup-Plan-tempo	OP	WR	1	1.54	0.43	0.57	4.56E-01	9.46E-01
995	27600	aparc-a2009s_rh_area_S-collat-transv-ant	OP	IVW	5	1.17	0.16	0.26	5.21E-01	9.46E-01
1007	27612	aparc-a2009s_rh_area_S-orbital-lateral	OP	IVW	2	1.97	0.68	1.15	5.57E-01	9.46E-01
1018	27623	aparc-a2009s_rh_area_S-temporal-sup	OP	IVW	5	1.23	0.21	0.38	5.80E-01	9.46E-01
1019	27624	aparc-a2009s_rh_area_S-temporal-transverse	OP	WR	1	0.59	-0.53	0.72	4.64E-01	9.46E-01
1045	26780	aparc-Desikan_lh_thickness_rostralanteriorcingulate	OP	IVW	2	0.80	-0.22	0.88	8.02E-01	9.48E-01
1121	27179	aparc-DKTatlas_lh_thickness_inferiorparietal	OP	IVW	8	0.94	-0.06	0.26	8.08E-01	9.48E-01
1134	27192	aparc-DKTatlas_lh_thickness_pericalcarine	OP	WR	1	0.73	-0.32	1.29	8.03E-01	9.48E-01
94	25850	IDP_T1_FAST_ROIs_L_parahipp_gyrus_post	OP	IVW	3	0.70	-0.36	0.55	5.13E-01	9.48E-01
1186	27411	aparc-a2009s_lh_thickness_G-cingul-Post-dorsal	OP	WR	1	1.28	0.25	1.07	8.16E-01	9.48E-01
660	26733	aparc-Desikan_lh_area_lingual	OP	IVW	17	1.34	0.29	0.15	5.96E-02	9.49E-01
661	26734	aparc-Desikan_lh_area_medialorbitofrontal	OP	WR	1	2.14	0.76	0.58	1.85E-01	9.49E-01
671	26744	aparc-Desikan_lh_area_precentral	OP	IVW	7	0.64	-0.45	0.24	6.65E-02	9.49E-01
678	26751	aparc-Desikan_lh_area_supramarginal	OP	IVW	5	0.74	-0.3	0.27	2.55E-01	9.49E-01
682	26822	aparc-Desikan_rh_area_TotalSurface	OP	IVW	15	0.77	-0.26	0.16	1.10E-01	9.49E-01
692	26832	aparc-Desikan_rh_area_lateraloccipital	OP	IVW	16	1.19	0.17	0.15	2.42E-01	9.49E-01
693	26833	aparc-Desikan_rh_area_lateralorbitofrontal	OP	IVW	7	0.65	-0.43	0.21	4.52E-02	9.49E-01
695	26835	aparc-Desikan_rh_area_medialorbitofrontal	OP	IVW	3	1.43	0.36	0.31	2.54E-01	9.49E-01
700	26840	aparc-Desikan_rh_area_parsorbitalis	OP	WR	1	2.10	0.74	0.44	9.06E-02	9.49E-01
702	26842	aparc-Desikan_rh_area_pericalcarine	OP	IVW	32	1.13	0.12	0.11	2.62E-01	9.49E-01
703	26843	aparc-Desikan_rh_area_postcentral	OP	IVW	7	0.64	-0.45	0.24	6.06E-02	9.49E-01

718	26925	aparc-pial_lh_area_caudalanteriorcingulate	OP	WR	1	2.69	0.99	0.56	7.48E-02	9.49E-01
719	26926	aparc-pial_lh_area_caudalmiddlefrontal	OP	IVW	4	0.73	-0.31	0.25	2.01E-01	9.49E-01
727	26934	aparc-pial_lh_area_lateralorbitofrontal	OP	IVW	9	0.76	-0.27	0.20	1.85E-01	9.49E-01
728	26935	aparc-pial_lh_area_lingual	OP	IVW	17	1.23	0.21	0.15	1.40E-01	9.49E-01
731	26938	aparc-pial_lh_area_parahippocampal	OP	WR	1	0.49	-0.71	0.62	2.55E-01	9.49E-01
734	26941	aparc-pial_lh_area_parsorbitalis	OP	IVW	3	2.20	0.79	0.61	1.94E-01	9.49E-01
742	26949	aparc-pial_lh_area_rostralmiddlefrontal	OP	IVW	6	1.75	0.56	0.38	1.42E-01	9.49E-01
744	26951	aparc-pial_lh_area_superiorparietal	OP	IVW	14	0.83	-0.19	0.14	1.93E-01	9.49E-01
745	26952	aparc-pial_lh_area_superiortemporal	OP	IVW	3	1.55	0.44	0.35	2.04E-01	9.49E-01
746	26953	aparc-pial_lh_area_supramarginal	OP	IVW	4	0.66	-0.42	0.26	1.09E-01	9.49E-01
749	26956	aparc-pial_rh_area_TotalSurface	OP	IVW	9	0.80	-0.22	0.20	2.57E-01	9.49E-01
753	26960	aparc-pial_rh_area_cuneus	OP	IVW	22	1.17	0.16	0.12	1.70E-01	9.49E-01
754	26961	aparc-pial_rh_area_entorhinal	OP	IVW	4	0.57	-0.57	0.30	6.16E-02	9.49E-01
760	26967	aparc-pial_rh_area_lateralorbitofrontal	OP	IVW	9	0.75	-0.29	0.25	2.46E-01	9.49E-01
769	26976	aparc-pial_rh_area_pericalcarine	OP	IVW	32	1.16	0.15	0.10	1.36E-01	9.49E-01
771	26978	aparc-pial_rh_area_posteriorcingulate	OP	IVW	4	1.73	0.55	0.29	6.06E-02	9.49E-01
775	26982	aparc-pial_rh_area_rostralmiddlefrontal	OP	IVW	3	1.55	0.44	0.32	1.71E-01	9.49E-01
812	27145	aparc-DKTatlas_lh_area_cuneus	OP	IVW	13	1.42	0.35	0.16	3.33E-02	9.49E-01
820	27153	aparc-DKTatlas_lh_area_lingual	OP	IVW	16	1.21	0.19	0.15	2.02E-01	9.49E-01
823	27156	aparc-DKTatlas_lh_area_parahippocampal	OP	IVW	2	0.53	-0.63	0.42	1.36E-01	9.49E-01
826	27159	aparc-DKTatlas_lh_area_parsorbitalis	OP	IVW	7	1.39	0.33	0.22	1.31E-01	9.49E-01
828	27161	aparc-DKTatlas_lh_area_pericalcarine	OP	IVW	32	1.12	0.11	0.10	2.42E-01	9.49E-01
831	27164	aparc-DKTatlas_lh_area_precentral	OP	IVW	9	0.69	-0.37	0.21	8.55E-02	9.49E-01
833	27166	aparc-DKTatlas_lh_area_rostralanteriorcingulate	OP	IVW	4	0.68	-0.38	0.27	1.60E-01	9.49E-01
839	27172	aparc-DKTatlas_lh_area_transversetemporal	OP	IVW	4	0.60	-0.51	0.34	1.35E-01	9.49E-01
844	27239	aparc-DKTatlas_rh_area_entorhinal	OP	IVW	3	1.52	0.42	0.38	2.64E-01	9.49E-01
848	27243	aparc-DKTatlas_rh_area_isthmuscingulate	OP	IVW	6	1.46	0.38	0.31	2.16E-01	9.49E-01
849	27244	aparc-DKTatlas_rh_area_lateraloccipital	OP	IVW	16	1.28	0.25	0.15	9.93E-02	9.49E-01
850	27245	aparc-DKTatlas_rh_area_lateralorbitofrontal	OP	IVW	7	0.68	-0.39	0.22	7.63E-02	9.49E-01
851	27246	aparc-DKTatlas_rh_area_lingual	OP	IVW	17	1.23	0.21	0.14	1.45E-01	9.49E-01
852	27247	aparc-DKTatlas_rh_area_medialorbitofrontal	OP	IVW	4	0.69	-0.37	0.29	1.99E-01	9.49E-01
860	27255	aparc-DKTatlas_rh_area_postcentral	OP	IVW	7	0.78	-0.25	0.21	2.32E-01	9.49E-01
862	27257	aparc-DKTatlas_rh_area_precentral	OP	IVW	5	0.66	-0.42	0.31	1.71E-01	9.49E-01

865	27260	aparc-DKTatlas_rh_area_rostralmiddlefrontal	OP	IVW	5	1.36	0.31	0.26	2.36E-01	9.49E-01
869	27264	aparc-DKTatlas_rh_area_supramarginal	OP	IVW	7	0.76	-0.27	0.21	1.93E-01	9.49E-01
871	27266	aparc-DKTatlas_rh_area_insula	OP	IVW	13	1.25	0.22	0.16	1.80E-01	9.49E-01
1027	26762	aparc-Desikan_lh_thickness_inferiorparietal	OP	IVW	9	0.95	-0.05	0.24	8.29E-01	9.49E-01
1054	26856	aparc-Desikan_rh_thickness_GlobalMeanThickness	OP	IVW	12	0.96	-0.04	0.18	8.32E-01	9.49E-01
315	26692	ThalamNuclei_rh_volume_L-Sg	OP	IVW	3	1.48	0.39	0.34	2.56E-01	9.51E-01
185	26534	aseg_global_volume_CC-Mid-Anterior	OP	IVW	13	0.99	-0.01	0.20	9.52E-01	9.52E-01
694	26834	aparc-Desikan_rh_area_lingual	OP	IVW	18	1.16	0.15	0.14	2.72E-01	9.52E-01
810	27143	aparc-DKTatlas_lh_area_caudalanteriorcingulate	OP	IVW	2	0.58	-0.55	0.51	2.80E-01	9.52E-01
830	27163	aparc-DKTatlas_lh_area_posteriorcingulate	OP	IVW	3	0.66	-0.41	0.37	2.77E-01	9.52E-01
880	27337	aparc-a2009s_lh_area_G-cingul-Post-dorsal	OP	IVW	2	0.77	-0.26	0.50	5.94E-01	9.53E-01
1193	27418	aparc-a2009s_lh_thickness_G-front-sup	OP	IVW	11	0.97	-0.03	0.18	8.85E-01	9.53E-01
1200	27425	aparc-a2009s_lh_thickness_G-oc-temp-med-Parahip	OP	IVW	3	0.91	-0.09	0.59	8.74E-01	9.53E-01
1251	27476	aparc-a2009s_lh_thickness_S-temporal-transverse	OP	WR	1	1.13	0.12	0.87	8.92E-01	9.53E-01
1255	27628	aparc-a2009s_rh_thickness_G+S-subcentral	OP	IVW	5	0.94	-0.06	0.37	8.79E-01	9.53E-01
649	26722	aparc-Desikan_lh_area_bankssts	OP	IVW	5	1.34	0.29	0.35	4.11E-01	9.54E-01
651	26724	aparc-Desikan_lh_area_caudalmiddlefrontal	OP	IVW	2	0.61	-0.49	0.57	3.92E-01	9.54E-01
652	26725	aparc-Desikan_lh_area_cuneus	OP	IVW	16	1.13	0.12	0.17	4.89E-01	9.54E-01
657	26730	aparc-Desikan_lh_area_isthmuscingulate	OP	IVW	2	1.36	0.31	0.53	5.64E-01	9.54E-01
658	26731	aparc-Desikan_lh_area_lateraloccipital	OP	IVW	11	0.88	-0.13	0.18	4.98E-01	9.54E-01
659	26732	aparc-Desikan_lh_area_lateralorbitofrontal	OP	IVW	11	0.86	-0.15	0.18	4.08E-01	9.54E-01
663	26736	aparc-Desikan_lh_area_parahippocampal	OP	WR	1	1.82	0.6	0.58	3.06E-01	9.54E-01
665	26738	aparc-Desikan_lh_area_parsopercularis	OP	IVW	3	1.25	0.22	0.32	4.88E-01	9.54E-01
667	26740	aparc-Desikan_lh_area_parstriangularis	OP	IVW	9	1.19	0.17	0.18	3.57E-01	9.54E-01
668	26741	aparc-Desikan_lh_area_pericalcarine	OP	IVW	34	1.09	0.09	0.10	3.52E-01	9.54E-01
669	26742	aparc-Desikan_lh_area_postcentral	OP	IVW	9	0.79	-0.23	0.33	4.81E-01	9.54E-01
672	26745	aparc-Desikan_lh_area_precuneus	OP	IVW	13	0.90	-0.1	0.16	5.33E-01	9.54E-01
674	26747	aparc-Desikan_lh_area_rostralmiddlefrontal	OP	IVW	7	1.40	0.34	0.41	3.97E-01	9.54E-01
676	26749	aparc-Desikan_lh_area_superiorparietal	OP	IVW	12	0.84	-0.17	0.19	3.69E-01	9.54E-01
677	26750	aparc-Desikan_lh_area_superiortemporal	OP	IVW	5	1.23	0.21	0.25	3.97E-01	9.54E-01
679	26752	aparc-Desikan_lh_area_frontalpole	OP	WR	1	1.60	0.47	0.57	4.05E-01	9.54E-01
680	26753	aparc-Desikan_lh_area_transversetemporal	OP	IVW	5	0.82	-0.2	0.31	5.10E-01	9.54E-01
683	26823	aparc-Desikan_rh_area_bankssts	OP	WR	1	1.57	0.45	0.58	4.45E-01	9.54E-01

684	26824	aparc-Desikan_rh_area_caudalanteriorcingulate	OP	IVW	4	0.73	-0.31	0.39	4.22E-01	9.54E-01
690	26830	aparc-Desikan_rh_area_inferiortemporal	OP	IVW	7	0.84	-0.17	0.23	4.76E-01	9.54E-01
698	26838	aparc-Desikan_rh_area_paracentral	OP	IVW	4	0.80	-0.22	0.32	4.85E-01	9.54E-01
699	26839	aparc-Desikan_rh_area_parsopercularis	OP	IVW	2	1.34	0.29	0.46	5.19E-01	9.54E-01
704	26844	aparc-Desikan_rh_area_posteriorcingulate	OP	IVW	3	0.72	-0.33	0.60	5.76E-01	9.54E-01
706	26846	aparc-Desikan_rh_area_precuneus	OP	IVW	13	1.15	0.14	0.23	5.28E-01	9.54E-01
707	26847	aparc-Desikan_rh_area_rostralanteriorcingulate	OP	IVW	2	1.31	0.27	0.40	4.91E-01	9.54E-01
710	26850	aparc-Desikan_rh_area_superiorparietal	OP	IVW	11	0.90	-0.1	0.18	5.67E-01	9.54E-01
712	26852	aparc-Desikan_rh_area_supramarginal	OP	IVW	8	0.80	-0.22	0.21	2.94E-01	9.54E-01
726	26933	aparc-pial_lh_area_lateraloccipital	OP	IVW	6	1.15	0.14	0.26	5.75E-01	9.54E-01
739	26946	aparc-pial_lh_area_precentral	OP	IVW	5	0.81	-0.21	0.23	3.76E-01	9.54E-01
751	26958	aparc-pial_rh_area_caudalanteriorcingulate	OP	IVW	3	0.63	-0.46	0.60	4.48E-01	9.54E-01
756	26963	aparc-pial_rh_area_inferiorparietal	OP	IVW	8	1.16	0.15	0.22	5.12E-01	9.54E-01
758	26965	aparc-pial_rh_area_isthmuscingulate	OP	IVW	3	1.43	0.36	0.38	3.52E-01	9.54E-01
763	26970	aparc-pial_rh_area_middletemporal	OP	IVW	4	0.66	-0.42	0.59	4.70E-01	9.54E-01
764	26971	aparc-pial_rh_area_parahippocampal	OP	IVW	5	0.81	-0.21	0.35	5.52E-01	9.54E-01
766	26973	aparc-pial_rh_area_parsopercularis	OP	WR	1	0.63	-0.46	0.66	4.85E-01	9.54E-01
770	26977	aparc-pial_rh_area_postcentral	OP	IVW	6	0.86	-0.15	0.24	5.49E-01	9.54E-01
773	26980	aparc-pial_rh_area_precuneus	OP	IVW	13	1.23	0.21	0.21	3.20E-01	9.54E-01
777	26984	aparc-pial_rh_area_superiorparietal	OP	IVW	8	0.86	-0.15	0.26	5.76E-01	9.54E-01
779	26986	aparc-pial_rh_area_supramarginal	OP	IVW	7	0.84	-0.17	0.22	4.39E-01	9.54E-01
781	26988	aparc-pial_rh_area_transversetemporal	OP	IVW	4	1.42	0.35	0.53	5.13E-01	9.54E-01
811	27144	aparc-DKTatlas_lh_area_caudalmiddlefrontal	OP	IVW	3	0.79	-0.23	0.38	5.54E-01	9.54E-01
815	27148	aparc-DKTatlas_lh_area_inferiorparietal	OP	IVW	5	1.25	0.22	0.29	4.43E-01	9.54E-01
818	27151	aparc-DKTatlas_lh_area_lateraloccipital	OP	IVW	12	1.17	0.16	0.17	3.59E-01	9.54E-01
819	27152	aparc-DKTatlas_lh_area_lateralorbitofrontal	OP	IVW	9	1.21	0.19	0.19	3.01E-01	9.54E-01
821	27154	aparc-DKTatlas_lh_area_medialorbitofrontal	OP	IVW	4	1.17	0.16	0.27	5.50E-01	9.54E-01
829	27162	aparc-DKTatlas_lh_area_postcentral	OP	IVW	9	0.80	-0.22	0.32	4.85E-01	9.54E-01
834	27167	aparc-DKTatlas_lh_area_rostralmiddlefrontal	OP	IVW	8	1.39	0.33	0.35	3.55E-01	9.54E-01
837	27170	aparc-DKTatlas_lh_area_superiortemporal	OP	IVW	5	1.20	0.18	0.24	4.43E-01	9.54E-01
838	27171	aparc-DKTatlas_lh_area_supramarginal	OP	IVW	5	0.78	-0.25	0.28	3.79E-01	9.54E-01
842	27237	aparc-DKTatlas_rh_area_caudalmiddlefrontal	OP	IVW	5	1.31	0.27	0.32	3.94E-01	9.54E-01
843	27238	aparc-DKTatlas_rh_area_cuneus	OP	IVW	21	0.91	-0.09	0.12	4.69E-01	9.54E-01

853	27248	aparc-DKTatlas_rh_area_middletemporal	OP	IVW	9	0.82	-0.2	0.29	5.03E-01	9.54E-01
855	27250	aparc-DKTatlas_rh_area_paracentral	OP	IVW	5	0.85	-0.16	0.27	5.61E-01	9.54E-01
856	27251	aparc-DKTatlas_rh_area_parsopercularis	OP	IVW	2	1.34	0.29	0.45	5.19E-01	9.54E-01
859	27254	aparc-DKTatlas_rh_area_pericalcarine	OP	IVW	32	1.09	0.09	0.10	3.58E-01	9.54E-01
861	27256	aparc-DKTatlas_rh_area_posteriorcingulate	OP	IVW	3	1.42	0.35	0.40	3.90E-01	9.54E-01
867	27262	aparc-DKTatlas_rh_area_superiorparietal	OP	IVW	7	0.84	-0.17	0.23	4.55E-01	9.54E-01
870	27265	aparc-DKTatlas_rh_area_transversetemporal	OP	IVW	3	0.74	-0.3	0.43	4.83E-01	9.54E-01
722	26929	aparc-pial_lh_area_fusiform	OP	IVW	3	1.21	0.19	0.36	5.92E-01	9.54E-01
748	26955	aparc-pial_lh_area_transversetemporal	OP	IVW	4	0.76	-0.28	0.51	5.82E-01	9.54E-01
817	27150	aparc-DKTatlas_lh_area_isthmuscingulate	OP	IVW	4	1.25	0.22	0.41	5.88E-01	9.54E-01
654	26727	aparc-Desikan_lh_area_fusiform	OP	IVW	3	1.20	0.18	0.36	6.08E-01	9.54E-01
733	26940	aparc-pial_lh_area_parsopercularis	OP	IVW	2	0.82	-0.2	0.39	6.12E-01	9.54E-01
814	27147	aparc-DKTatlas_lh_area_fusiform	OP	IVW	4	1.16	0.15	0.30	6.12E-01	9.54E-01
854	27249	aparc-DKTatlas_rh_area_parahippocampal	OP	IVW	3	0.73	-0.32	0.62	6.08E-01	9.54E-01
243	26620	HippSubfield_lh_volume_Hippocampal-tail	OP	IVW	15	0.95	-0.05	0.13	6.84E-01	9.56E-01
244	26621	HippSubfield_lh_volume_subiculum-body	OP	IVW	13	0.91	-0.09	0.17	6.02E-01	9.56E-01
246	26623	HippSubfield_lh_volume_subiculum-head	OP	IVW	6	1.22	0.2	0.22	3.70E-01	9.56E-01
249	26626	HippSubfield_lh_volume_CA1-head	OP	IVW	11	1.12	0.11	0.15	4.58E-01	9.56E-01
250	26627	HippSubfield_lh_volume_presubiculum-body	OP	IVW	8	0.93	-0.07	0.27	7.91E-01	9.56E-01
251	26628	HippSubfield_lh_volume_parasubiculum	OP	IVW	2	0.79	-0.23	0.46	6.16E-01	9.56E-01
252	26629	HippSubfield_lh_volume_molecular-layer-HP-head	OP	IVW	5	0.84	-0.18	0.28	5.11E-01	9.56E-01
254	26631	HippSubfield_lh_volume_GC-ML-DG-head	OP	IVW	6	1.07	0.07	0.22	7.61E-01	9.56E-01
257	26634	HippSubfield_lh_volume_CA4-head	OP	IVW	8	0.96	-0.04	0.19	8.45E-01	9.56E-01
259	26636	HippSubfield_lh_volume_fimbria	OP	IVW	2	0.70	-0.36	0.86	6.80E-01	9.56E-01
260	26637	HippSubfield_lh_volume_CA3-head	OP	IVW	7	1.05	0.05	0.23	8.23E-01	9.56E-01
263	26640	HippSubfield_lh_volume_Whole-hippocampal-head	OP	IVW	9	1.16	0.15	0.19	4.15E-01	9.56E-01
267	26644	HippSubfield_rh_volume_CA1-body	OP	IVW	5	1.11	0.1	0.26	6.82E-01	9.56E-01
268	26645	HippSubfield_rh_volume_subiculum-head	OP	IVW	9	1.06	0.06	0.17	7.23E-01	9.56E-01
271	26648	HippSubfield_rh_volume_CA1-head	OP	IVW	11	1.08	0.08	0.15	5.82E-01	9.56E-01
272	26649	HippSubfield_rh_volume_presubiculum-body	OP	IVW	7	0.86	-0.15	0.29	6.08E-01	9.56E-01
274	26651	HippSubfield_rh_volume_molecular-layer-HP-head	OP	IVW	7	1.09	0.09	0.24	6.95E-01	9.56E-01
275	26652	HippSubfield_rh_volume_molecular-layer-HP-body	OP	IVW	5	0.84	-0.17	0.27	5.28E-01	9.56E-01
277	26654	HippSubfield_rh_volume_CA3-body	OP	IVW	5	1.09	0.09	0.27	7.24E-01	9.56E-01

278	26655	HippSubfield_rh_volume_GC-ML-DG-body	OP	IVW	9	0.88	-0.13	0.21	5.23E-01	9.56E-01
279	26656	HippSubfield_rh_volume_CA4-head	OP	IVW	10	1.05	0.05	0.16	7.45E-01	9.56E-01
282	26659	HippSubfield_rh_volume_CA3-head	OP	IVW	7	1.20	0.18	0.22	4.11E-01	9.56E-01
283	26660	HippSubfield_rh_volume_HATA	OP	IVW	3	1.22	0.2	0.34	5.65E-01	9.56E-01
284	26661	HippSubfield_rh_volume_Whole-hippocampal-body	OP	IVW	9	0.96	-0.04	0.19	8.28E-01	9.56E-01
877	27334	aparc-a2009s_lh_area_G+S-cingul-Ant	OP	IVW	2	1.30	0.26	0.53	6.27E-01	9.57E-01
886	27343	aparc-a2009s_lh_area_G-front-middle	OP	IVW	4	1.14	0.13	0.46	7.80E-01	9.57E-01
894	27351	aparc-a2009s_lh_area_G-oc-temp-med-Parahip	OP	IVW	3	1.14	0.13	0.39	7.35E-01	9.57E-01
900	27357	aparc-a2009s_lh_area_G-precentral	OP	IVW	4	0.85	-0.16	0.36	6.53E-01	9.57E-01
901	27358	aparc-a2009s_lh_area_G-precuneus	OP	IVW	9	0.91	-0.09	0.22	7.03E-01	9.57E-01
909	27366	aparc-a2009s_lh_area_G-temporal-middle	OP	IVW	5	1.17	0.16	0.51	7.61E-01	9.57E-01
919	27376	aparc-a2009s_lh_area_S-circular-insula-inf	OP	IVW	5	0.90	-0.1	0.29	7.25E-01	9.57E-01
920	27377	aparc-a2009s_lh_area_S-circular-insula-sup	OP	IVW	7	1.08	0.08	0.20	6.76E-01	9.57E-01
921	27378	aparc-a2009s_lh_area_S-collat-transv-ant	OP	IVW	6	1.07	0.07	0.24	7.65E-01	9.57E-01
925	27382	aparc-a2009s_lh_area_S-front-sup	OP	IVW	3	1.39	0.33	0.82	6.89E-01	9.57E-01
938	27395	aparc-a2009s_lh_area_S-postcentral	OP	IVW	5	0.90	-0.1	0.21	6.52E-01	9.57E-01
944	27401	aparc-a2009s_lh_area_S-temporal-sup	OP	IVW	7	1.13	0.12	0.32	6.95E-01	9.57E-01
946	27551	aparc-a2009s_rh_area_G+S-frontomargin	OP	WR	1	0.71	-0.34	0.87	6.93E-01	9.57E-01
950	27555	aparc-a2009s_rh_area_G+S-transv-frontopol	OP	IVW	2	1.22	0.2	0.48	6.74E-01	9.57E-01
951	27556	aparc-a2009s_rh_area_G+S-cingul-Ant	OP	IVW	2	0.64	-0.45	0.89	6.15E-01	9.57E-01
962	27567	aparc-a2009s_rh_area_G-Ins-lg+S-cent-ins	OP	WR	1	0.84	-0.17	0.51	7.36E-01	9.57E-01
963	27568	aparc-a2009s_rh_area_G-insular-short	OP	WR	1	0.85	-0.16	0.55	7.68E-01	9.57E-01
970	27575	aparc-a2009s_rh_area_G-pariet-inf-Angular	OP	IVW	2	0.77	-0.26	0.53	6.16E-01	9.57E-01
971	27576	aparc-a2009s_rh_area_G-pariet-inf-Supramar	OP	IVW	7	0.90	-0.1	0.22	6.39E-01	9.57E-01
973	27578	aparc-a2009s_rh_area_G-postcentral	OP	IVW	4	0.91	-0.09	0.25	7.06E-01	9.57E-01
989	27594	aparc-a2009s_rh_area_S-calcarine	OP	IVW	30	0.96	-0.04	0.10	7.08E-01	9.57E-01
996	27601	aparc-a2009s_rh_area_S-collat-transv-post	OP	WR	1	1.32	0.28	0.90	7.58E-01	9.57E-01
1005	27610	aparc-a2009s_rh_area_S-oc-temp-lat	OP	IVW	2	1.28	0.25	0.56	6.52E-01	9.57E-01
1006	27611	aparc-a2009s_rh_area_S-oc-temp-med+Lingual	OP	IVW	6	1.12	0.11	0.38	7.79E-01	9.57E-01
1011	27616	aparc-a2009s_rh_area_S-pericallosal	OP	IVW	4	1.17	0.16	0.48	7.44E-01	9.57E-01
1226	27451	aparc-a2009s_lh_thickness_S-circular-insula-sup	OP	IVW	5	1.05	0.05	0.51	9.18E-01	9.57E-01
1272	27645	aparc-a2009s_rh_thickness_G-oc-temp-lat-fusifor	OP	IVW	2	1.06	0.06	0.48	9.09E-01	9.57E-01
1312	27685	aparc-a2009s_rh_thickness_S-oc-temp-med+Lingual	OP	IVW	3	1.03	0.03	0.33	9.22E-01	9.57E-01

428	27133	BA-exvivo_rh_volume_BA4a	OP	IVW	2	0.95	-0.05	0.48	9.24E-01	9.60E-01
429	27134	BA-exvivo_rh_volume_BA4p	OP	IVW	2	1.04	0.04	0.34	9.06E-01	9.60E-01
435	27140	BA-exvivo_rh_volume_MT	OP	IVW	7	1.04	0.04	0.22	8.62E-01	9.60E-01
500	27477	aparc-a2009s_lh_volume_G+S-frontomargin	OP	WR	1	1.27	0.24	0.58	6.86E-01	9.60E-01
1294	27667	aparc-a2009s_rh_thickness_Pole-temporal	OP	IVW	4	0.98	-0.02	0.29	9.36E-01	9.62E-01
261	26638	HippSubfield_lh_volume_HATA	OP	IVW	2	0.95	-0.05	0.40	9.03E-01	9.62E-01
269	26646	HippSubfield_rh_volume_hippocampal-fissure	OP	IVW	8	1.03	0.03	0.34	9.18E-01	9.62E-01
276	26653	HippSubfield_rh_volume_GC-ML-DG-head	OP	IVW	9	1.03	0.03	0.19	8.92E-01	9.62E-01
161	25917	IDP_T1_FAST_ROIs_R_cerebellum_IX	OP	IVW	19	1.09	0.09	0.14	5.29E-01	9.63E-01
1317	27690	aparc-a2009s_rh_thickness_S-pericallosal	OP	IVW	6	1.01	0.01	0.21	9.45E-01	9.63E-01
717	26924	aparc-pial_lh_area_bankssts	OP	IVW	4	1.20	0.18	0.37	6.30E-01	9.66E-01
863	27258	aparc-DKTatlas_rh_area_precuneus	OP	IVW	11	1.14	0.13	0.27	6.29E-01	9.66E-01
1127	27185	aparc-DKTatlas_lh_thickness_medialorbitofrontal	OP	WR	1	1.19	0.17	0.90	8.54E-01	9.67E-01
648	26721	aparc-Desikan_lh_area_TotalSurface	OP	IVW	11	0.93	-0.07	0.19	7.13E-01	9.68E-01
656	26729	aparc-Desikan_lh_area_inferiortemporal	OP	WR	1	1.23	0.21	0.91	8.13E-01	9.68E-01
666	26739	aparc-Desikan_lh_area_parsorbitalis	OP	IVW	6	1.17	0.16	0.38	6.77E-01	9.68E-01
670	26743	aparc-Desikan_lh_area_posteriorcingulate	OP	IVW	3	0.94	-0.06	0.36	8.62E-01	9.68E-01
673	26746	aparc-Desikan_lh_area_rostralanteriorcingulate	OP	IVW	2	1.14	0.13	0.50	7.89E-01	9.68E-01
675	26748	aparc-Desikan_lh_area_superiorfrontal	OP	IVW	7	0.93	-0.07	0.30	8.19E-01	9.68E-01
681	26754	aparc-Desikan_lh_area_insula	OP	IVW	5	1.09	0.09	0.30	7.79E-01	9.68E-01
685	26825	aparc-Desikan_rh_area_caudalmiddlefrontal	OP	IVW	6	0.93	-0.07	0.30	8.24E-01	9.68E-01
686	26826	aparc-Desikan_rh_area_cuneus	OP	IVW	20	1.02	0.02	0.12	8.69E-01	9.68E-01
687	26827	aparc-Desikan_rh_area_entorhinal	OP	IVW	2	1.16	0.15	0.43	7.30E-01	9.68E-01
688	26828	aparc-Desikan_rh_area_fusiform	OP	IVW	2	0.84	-0.18	1.09	8.66E-01	9.68E-01
689	26829	aparc-Desikan_rh_area_inferiorparietal	OP	IVW	11	1.07	0.07	0.21	7.50E-01	9.68E-01
696	26836	aparc-Desikan_rh_area_middletemporal	OP	IVW	7	0.88	-0.13	0.38	7.34E-01	9.68E-01
697	26837	aparc-Desikan_rh_area_parahippocampal	OP	IVW	3	0.90	-0.1	0.64	8.81E-01	9.68E-01
705	26845	aparc-Desikan_rh_area_precentral	OP	IVW	7	1.05	0.05	0.31	8.84E-01	9.68E-01
709	26849	aparc-Desikan_rh_area_superiorfrontal	OP	IVW	3	0.80	-0.22	0.49	6.56E-01	9.68E-01
711	26851	aparc-Desikan_rh_area_superiortemporal	OP	IVW	3	1.15	0.14	0.46	7.65E-01	9.68E-01
714	26854	aparc-Desikan_rh_area_transversetemporal	OP	IVW	2	0.79	-0.23	0.78	7.66E-01	9.68E-01
715	26855	aparc-Desikan_rh_area_insula	OP	IVW	3	1.13	0.12	0.51	8.12E-01	9.68E-01
716	26923	aparc-pial_lh_area_TotalSurface	OP	IVW	7	0.91	-0.09	0.26	7.17E-01	9.68E-01

725	26932	aparc-pial_lh_area_isthmuscingulate	OP	IVW	4	1.13	0.12	0.32	7.14E-01	9.68E-01
730	26937	aparc-pial_lh_area_middletemporal	OP	IVW	6	1.15	0.14	0.45	7.59E-01	9.68E-01
732	26939	aparc-pial_lh_area_paracentral	OP	IVW	2	0.93	-0.07	0.41	8.69E-01	9.68E-01
735	26942	aparc-pial_lh_area_parstriangularis	OP	IVW	7	1.04	0.04	0.21	8.64E-01	9.68E-01
736	26943	aparc-pial_lh_area_pericalcarine	OP	IVW	21	0.95	-0.05	0.12	6.66E-01	9.68E-01
737	26944	aparc-pial_lh_area_postcentral	OP	IVW	6	1.08	0.08	0.25	7.54E-01	9.68E-01
738	26945	aparc-pial_lh_area_posteriorcingulate	OP	IVW	3	1.16	0.15	0.36	6.79E-01	9.68E-01
741	26948	aparc-pial_lh_area_rostralanteriorcingulate	OP	IVW	2	0.87	-0.14	0.92	8.75E-01	9.68E-01
743	26950	aparc-pial_lh_area_superiorfrontal	OP	IVW	6	0.89	-0.12	0.33	7.26E-01	9.68E-01
752	26959	aparc-pial_rh_area_caudalmiddlefrontal	OP	IVW	4	0.85	-0.16	0.37	6.77E-01	9.68E-01
757	26964	aparc-pial_rh_area_inferiortemporal	OP	IVW	2	0.90	-0.1	0.55	8.52E-01	9.68E-01
765	26972	aparc-pial_rh_area_paracentral	OP	IVW	2	0.88	-0.13	0.51	7.94E-01	9.68E-01
767	26974	aparc-pial_rh_area_parsorbitalis	OP	IVW	2	1.70	0.53	1.25	6.69E-01	9.68E-01
772	26979	aparc-pial_rh_area_precentral	OP	IVW	7	0.93	-0.07	0.27	7.85E-01	9.68E-01
776	26983	aparc-pial_rh_area_superiorfrontal	OP	IVW	8	0.94	-0.06	0.23	8.10E-01	9.68E-01
778	26985	aparc-pial_rh_area_superiortemporal	OP	IVW	4	0.93	-0.07	0.30	8.19E-01	9.68E-01
813	27146	aparc-DKTatlas_lh_area_entorhinal	OP	IVW	4	1.08	0.08	0.26	7.61E-01	9.68E-01
816	27149	aparc-DKTatlas_lh_area_inferiortemporal	OP	WR	1	1.32	0.28	0.91	7.58E-01	9.68E-01
822	27155	aparc-DKTatlas_lh_area_middletemporal	OP	IVW	10	1.12	0.11	0.24	6.48E-01	9.68E-01
825	27158	aparc-DKTatlas_lh_area_parsopercularis	OP	IVW	4	1.05	0.05	0.29	8.59E-01	9.68E-01
827	27160	aparc-DKTatlas_lh_area_parstriangularis	OP	IVW	10	1.07	0.07	0.18	6.78E-01	9.68E-01
836	27169	aparc-DKTatlas_lh_area_superiorparietal	OP	IVW	12	0.97	-0.03	0.17	8.49E-01	9.68E-01
840	27173	aparc-DKTatlas_lh_area_insula	OP	IVW	10	1.03	0.03	0.17	8.53E-01	9.68E-01
841	27236	aparc-DKTatlas_rh_area_caudalanteriorcingulate	OP	IVW	3	0.95	-0.05	0.36	8.85E-01	9.68E-01
845	27240	aparc-DKTatlas_rh_area_fusiform	OP	IVW	3	1.13	0.12	0.66	8.55E-01	9.68E-01
846	27241	aparc-DKTatlas_rh_area_inferiorparietal	OP	IVW	9	0.92	-0.08	0.21	7.11E-01	9.68E-01
847	27242	aparc-DKTatlas_rh_area_inferiortemporal	OP	IVW	4	0.91	-0.09	0.32	7.75E-01	9.68E-01
857	27252	aparc-DKTatlas_rh_area_parsorbitalis	OP	IVW	4	1.12	0.11	0.35	7.41E-01	9.68E-01
858	27253	aparc-DKTatlas_rh_area_parstriangularis	OP	IVW	4	0.92	-0.08	0.28	7.67E-01	9.68E-01
662	26735	aparc-Desikan_lh_area_middletemporal	OP	IVW	9	1.03	0.03	0.29	9.11E-01	9.70E-01
723	26930	aparc-pial_lh_area_inferiorparietal	OP	IVW	5	0.97	-0.03	0.26	9.11E-01	9.70E-01
824	27157	aparc-DKTatlas_lh_area_paracentral	OP	IVW	3	1.05	0.05	0.40	8.98E-01	9.70E-01
832	27165	aparc-DKTatlas_lh_area_precuneus	OP	IVW	15	1.02	0.02	0.18	9.13E-01	9.70E-01

864	27259	aparc-DKTatlas_rh_area_rostralanteriorcingulate	OP	WR	1	1.07	0.07	0.60	9.08E-01	9.70E-01
394	26907	aparc-Desikan_rh_volume_parsorbitalis	OP	IVW	2	2.89	1.06	0.72	1.39E-01	9.71E-01
339	26716	Brainstem_global_volume_Medulla	OP	IVW	23	1.00	0	0.12	9.71E-01	9.71E-01
740	26947	aparc-pial_lh_area_precuneus	OP	IVW	11	0.98	-0.02	0.23	9.21E-01	9.73E-01
108	25864	IDP_T1_FAST_ROIs_L_cent_operc_cortex	OP	WR	1	1.31	0.27	0.46	5.56E-01	9.73E-01
153	25909	IDP_T1_FAST_ROIs_L_cerebellum_VIIIa	OP	IVW	18	0.93	-0.07	0.12	5.49E-01	9.73E-01
155	25911	IDP_T1_FAST_ROIs_R_cerebellum_VIIIa	OP	IVW	14	0.90	-0.1	0.16	5.50E-01	9.73E-01
255	26632	HippSubfield_lh_volume_CA3-body	OP	IVW	6	1.02	0.02	0.28	9.51E-01	9.74E-01
1	25001	IDP_T1_SIENAX_peripheral_grey_normalised_volume	OP	IVW	8	1.01	0.01	0.20	9.70E-01	9.76E-01
2	25002	IDP_T1_SIENAX_peripheral_grey_unnormalised_volume	OP	IVW	7	0.93	-0.07	0.22	7.57E-01	9.76E-01
3	25003	IDP_T1_SIENAX_CSF_normalised_volume	OP	IVW	16	1.02	0.02	0.15	9.02E-01	9.76E-01
4	25004	IDP_T1_SIENAX_CSF_unnormalised_volume	OP	IVW	17	1.04	0.04	0.15	7.61E-01	9.76E-01
5	25005	IDP_T1_SIENAX_grey_normalised_volume	OP	IVW	5	1.08	0.08	0.39	8.46E-01	9.76E-01
6	25006	IDP_T1_SIENAX_grey_unnormalised_volume	OP	IVW	6	1.13	0.12	0.33	7.16E-01	9.76E-01
7	25007	IDP_T1_SIENAX_white_normalised_volume	OP	IVW	19	1.15	0.14	0.15	3.26E-01	9.76E-01
8	25008	IDP_T1_SIENAX_white_unnormalised_volume	OP	IVW	18	1.12	0.11	0.14	4.54E-01	9.76E-01
9	25009	IDP_T1_SIENAX_brain-normalised_volume	OP	IVW	9	0.96	-0.04	0.23	8.47E-01	9.76E-01
10	25010	IDP_T1_SIENAX_brain-unnormalised_volume	OP	IVW	10	0.84	-0.18	0.19	3.66E-01	9.76E-01
11	25011	IDP_T1_FIRST_left_thalamus_volume	OP	IVW	11	1.17	0.16	0.18	3.80E-01	9.76E-01
12	25012	IDP_T1_FIRST_right_thalamus_volume	OP	IVW	8	1.08	0.08	0.25	7.49E-01	9.76E-01
13	25013	IDP_T1_FIRST_left_caudate_volume	OP	IVW	21	1.07	0.07	0.13	6.05E-01	9.76E-01
14	25014	IDP_T1_FIRST_right_caudate_volume	OP	IVW	23	1.25	0.22	0.12	7.03E-02	9.76E-01
15	25015	IDP_T1_FIRST_left_putamen_volume	OP	IVW	10	0.89	-0.12	0.17	4.97E-01	9.76E-01
16	25016	IDP_T1_FIRST_right_putamen_volume	OP	IVW	12	1.05	0.05	0.16	7.48E-01	9.76E-01
17	25017	IDP_T1_FIRST_left_pallidum_volume	OP	IVW	8	0.91	-0.09	0.21	6.62E-01	9.76E-01
18	25018	IDP_T1_FIRST_right_pallidum_volume	OP	IVW	7	0.84	-0.18	0.22	4.22E-01	9.76E-01
19	25019	IDP_T1_FIRST_left_hippocampus_volume	OP	IVW	6	0.99	-0.01	0.22	9.76E-01	9.76E-01
20	25020	IDP_T1_FIRST_right_hippocampus_volume	OP	IVW	7	0.82	-0.2	0.22	3.65E-01	9.76E-01
22	25022	IDP_T1_FIRST_right_amygdala_volume	OP	IVW	2	1.39	0.33	0.37	3.72E-01	9.76E-01
23	25023	IDP_T1_FIRST_left_accumbens_volume	OP	IVW	3	1.40	0.34	0.36	3.44E-01	9.76E-01
24	25024	IDP_T1_FIRST_right_accumbens_volume	OP	IVW	4	1.03	0.03	0.40	9.33E-01	9.76E-01
25	25025	IDP_T1_FIRST_brain_stem+4th_ventricle_volume	OP	IVW	20	0.97	-0.03	0.14	8.32E-01	9.76E-01
1039	26774	aparc-Desikan_lh_thickness_parstriangularis	OP	IVW	2	1.07	0.07	0.41	8.72E-01	9.78E-01

917	27374	aparc-a2009s_lh_area_S-cingul-Marginalis	OP	IVW	3	0.93	-0.07	0.33	8.36E-01	9.79E-01
923	27380	aparc-a2009s_lh_area_S-front-inf	OP	IVW	2	1.14	0.13	0.59	8.32E-01	9.79E-01
927	27384	aparc-a2009s_lh_area_S-intrapariet+P-trans	OP	IVW	5	0.93	-0.07	0.32	8.36E-01	9.79E-01
945	27402	aparc-a2009s_lh_area_S-temporal-transverse	OP	WR	1	0.87	-0.14	0.59	8.17E-01	9.79E-01
1009	27614	aparc-a2009s_rh_area_S-orbital-H-Shaped	OP	IVW	4	0.94	-0.06	0.28	8.36E-01	9.79E-01
897	27354	aparc-a2009s_lh_area_G-pariet-inf-Supramar	OP	IVW	8	0.96	-0.04	0.20	8.52E-01	9.80E-01
1010	27615	aparc-a2009s_rh_area_S-parieto-occipital	OP	IVW	13	1.03	0.03	0.18	8.50E-01	9.80E-01
655	26728	aparc-Desikan_lh_area_inferiorparietal	OP	IVW	5	1.02	0.02	0.28	9.33E-01	9.80E-01
1093	27078	BA-exvivo_lh_thickness_BA4p	OP	IVW	2	1.01	0.01	0.58	9.80E-01	9.80E-01
344	26789	aparc-Desikan_lh_volume_bankssts	OP	IVW	6	1.22	0.2	0.26	4.41E-01	9.80E-01
346	26791	aparc-Desikan_lh_volume_caudalmiddlefrontal	OP	IVW	5	0.76	-0.28	0.23	2.31E-01	9.80E-01
350	26795	aparc-Desikan_lh_volume_inferiorparietal	OP	IVW	4	1.34	0.29	0.30	3.31E-01	9.80E-01
357	26802	aparc-Desikan_lh_volume_middletemporal	OP	IVW	7	1.21	0.19	0.24	4.31E-01	9.80E-01
363	26808	aparc-Desikan_lh_volume_pericalcarine	OP	IVW	23	1.13	0.12	0.11	2.90E-01	9.80E-01
366	26811	aparc-Desikan_lh_volume_precentral	OP	IVW	3	1.22	0.2	0.27	4.63E-01	9.80E-01
367	26812	aparc-Desikan_lh_volume_precuneus	OP	IVW	8	1.16	0.15	0.20	4.78E-01	9.80E-01
368	26813	aparc-Desikan_lh_volume_rostralanteriorcingulate	OP	IVW	2	0.50	-0.69	0.74	3.49E-01	9.80E-01
371	26816	aparc-Desikan_lh_volume_superiorparietal	OP	IVW	15	0.84	-0.18	0.15	2.38E-01	9.80E-01
372	26817	aparc-Desikan_lh_volume_superiortemporal	OP	IVW	4	0.71	-0.34	0.34	3.19E-01	9.80E-01
373	26818	aparc-Desikan_lh_volume_supramarginal	OP	IVW	2	0.60	-0.51	0.69	4.61E-01	9.80E-01
376	26821	aparc-Desikan_lh_volume_insula	OP	IVW	8	1.26	0.23	0.19	2.24E-01	9.80E-01
382	26895	aparc-Desikan_rh_volume_fusiform	OP	IVW	4	1.28	0.25	0.32	4.44E-01	9.80E-01
383	26896	aparc-Desikan_rh_volume_inferiorparietal	OP	IVW	5	1.23	0.21	0.31	4.96E-01	9.80E-01
384	26897	aparc-Desikan_rh_volume_inferiortemporal	OP	IVW	4	1.45	0.37	0.39	3.41E-01	9.80E-01
385	26898	aparc-Desikan_rh_volume_isthmuscingulate	OP	WR	1	2.05	0.72	0.59	2.22E-01	9.80E-01
388	26901	aparc-Desikan_rh_volume_lingual	OP	IVW	10	1.26	0.23	0.19	2.37E-01	9.80E-01
392	26905	aparc-Desikan_rh_volume_paracentral	OP	WR	1	1.58	0.46	0.65	4.84E-01	9.80E-01
397	26910	aparc-Desikan_rh_volume_postcentral	OP	IVW	5	0.57	-0.56	0.44	2.01E-01	9.80E-01
402	26915	aparc-Desikan_rh_volume_rostralmiddlefrontal	OP	IVW	7	1.19	0.17	0.21	4.09E-01	9.80E-01
404	26917	aparc-Desikan_rh_volume_superiorparietal	OP	IVW	6	0.80	-0.22	0.30	4.68E-01	9.80E-01
408	26921	aparc-Desikan_rh_volume_transversetemporal	OP	IVW	2	1.77	0.57	0.40	1.53E-01	9.80E-01
409	26922	aparc-Desikan_rh_volume_insula	OP	IVW	4	1.36	0.31	0.28	2.59E-01	9.80E-01
438	27205	aparc-DKTatlas_lh_volume_caudalanteriorcingulate	OP	IVW	3	0.77	-0.26	0.39	5.00E-01	9.80E-01

439	27206	aparc-DKTatlas_lh_volume_caudalmiddlefrontal	OP	IVW	5	0.76	-0.28	0.23	2.20E-01	9.80E-01
441	27208	aparc-DKTatlas_lh_volume_entorhinal	OP	IVW	2	0.51	-0.67	0.60	2.62E-01	9.80E-01
447	27214	aparc-DKTatlas_lh_volume_lateralorbitofrontal	OP	IVW	5	1.38	0.32	0.26	2.07E-01	9.80E-01
448	27215	aparc-DKTatlas_lh_volume_lingual	OP	IVW	12	0.87	-0.14	0.17	3.88E-01	9.80E-01
450	27217	aparc-DKTatlas_lh_volume_middletemporal	OP	IVW	8	1.36	0.31	0.22	1.62E-01	9.80E-01
456	27223	aparc-DKTatlas_lh_volume_pericalcarine	OP	IVW	24	1.13	0.12	0.11	2.98E-01	9.80E-01
459	27226	aparc-DKTatlas_lh_volume_precentral	OP	IVW	4	1.19	0.17	0.25	4.95E-01	9.80E-01
466	27233	aparc-DKTatlas_lh_volume_supramarginal	OP	IVW	2	0.61	-0.5	0.71	4.81E-01	9.80E-01
468	27235	aparc-DKTatlas_lh_volume_insula	OP	IVW	10	1.25	0.22	0.25	3.74E-01	9.80E-01
469	27298	aparc-DKTatlas_rh_volume_caudalanteriorcingulate	OP	WR	1	0.68	-0.38	0.57	5.03E-01	9.80E-01
472	27301	aparc-DKTatlas_rh_volume_entorhinal	OP	IVW	2	0.59	-0.53	0.51	2.95E-01	9.80E-01
473	27302	aparc-DKTatlas_rh_volume_fusiform	OP	IVW	2	1.65	0.5	0.49	3.09E-01	9.80E-01
475	27304	aparc-DKTatlas_rh_volume_inferiortemporal	OP	WR	1	0.59	-0.53	0.58	3.54E-01	9.80E-01
479	27308	aparc-DKTatlas_rh_volume_lingual	OP	IVW	10	1.20	0.18	0.19	3.31E-01	9.80E-01
480	27309	aparc-DKTatlas_rh_volume_medialorbitofrontal	OP	IVW	3	1.40	0.34	0.34	3.22E-01	9.80E-01
483	27312	aparc-DKTatlas_rh_volume_paracentral	OP	IVW	2	1.68	0.52	0.44	2.35E-01	9.80E-01
485	27314	aparc-DKTatlas_rh_volume_parsorbitalis	OP	IVW	3	1.63	0.49	0.72	4.99E-01	9.80E-01
487	27316	aparc-DKTatlas_rh_volume_pericalcarine	OP	IVW	24	1.11	0.1	0.11	3.81E-01	9.80E-01
488	27317	aparc-DKTatlas_rh_volume_postcentral	OP	IVW	3	0.79	-0.23	0.33	4.89E-01	9.80E-01
495	27324	aparc-DKTatlas_rh_volume_superiorparietal	OP	IVW	7	0.76	-0.27	0.24	2.69E-01	9.80E-01
664	26737	aparc-Desikan_lh_area_paracentral	OP	IVW	2	0.98	-0.02	0.46	9.68E-01	9.82E-01
701	26841	aparc-Desikan_rh_area_parstriangularis	OP	IVW	3	0.98	-0.02	0.31	9.51E-01	9.82E-01
708	26848	aparc-Desikan_rh_area_rostralmiddlefrontal	OP	IVW	5	0.99	-0.01	0.27	9.63E-01	9.82E-01
713	26853	aparc-Desikan_rh_area_frontalpole	OP	WR	1	0.96	-0.04	0.82	9.62E-01	9.82E-01
768	26975	aparc-pial_rh_area_parstriangularis	OP	IVW	3	0.99	-0.01	0.32	9.71E-01	9.82E-01
835	27168	aparc-DKTatlas_lh_area_superiorfrontal	OP	IVW	4	1.02	0.02	0.40	9.62E-01	9.82E-01
866	27261	aparc-DKTatlas_rh_area_superiorfrontal	OP	IVW	4	0.98	-0.02	0.40	9.64E-01	9.82E-01
1220	27445	aparc-a2009s_lh_thickness_Pole-temporal	OP	IVW	2	0.99	-0.01	0.45	9.83E-01	9.83E-01
1269	27642	aparc-a2009s_rh_thickness_G-insular-short	OP	IVW	3	1.01	0.01	0.33	9.80E-01	9.83E-01
288	26665	ThalamNuclei_lh_volume_LGN	OP	IVW	4	1.03	0.03	0.56	9.59E-01	9.85E-01
289	26666	ThalamNuclei_lh_volume_PuI	OP	IVW	3	0.80	-0.22	0.94	8.12E-01	9.85E-01
291	26668	ThalamNuclei_lh_volume_L-Sg	OP	IVW	3	1.02	0.02	0.37	9.53E-01	9.85E-01
292	26669	ThalamNuclei_lh_volume_VPL	OP	IVW	8	0.85	-0.16	0.21	4.48E-01	9.85E-01

293	26670	ThalamNuclei_lh_volume_CM	OP	IVW	16	1.01	0.01	0.16	9.66E-01	9.85E-01
294	26671	ThalamNuclei_lh_volume_VLa	OP	IVW	15	1.12	0.11	0.15	4.75E-01	9.85E-01
296	26673	ThalamNuclei_lh_volume_MDm	OP	IVW	3	1.25	0.22	0.44	6.18E-01	9.85E-01
298	26675	ThalamNuclei_lh_volume_VAmc	OP	IVW	8	0.98	-0.02	0.25	9.28E-01	9.85E-01
299	26676	ThalamNuclei_lh_volume_MDl	OP	IVW	4	0.74	-0.3	0.38	4.31E-01	9.85E-01
300	26677	ThalamNuclei_lh_volume_CeM	OP	IVW	7	0.94	-0.06	0.22	7.83E-01	9.85E-01
302	26679	ThalamNuclei_lh_volume_MV(Re)	OP	IVW	7	0.82	-0.2	0.22	3.59E-01	9.85E-01
303	26680	ThalamNuclei_lh_volume_VM	OP	IVW	5	0.86	-0.15	0.25	5.44E-01	9.85E-01
305	26682	ThalamNuclei_lh_volume_PuL	OP	IVW	3	0.81	-0.21	0.34	5.33E-01	9.85E-01
307	26684	ThalamNuclei_lh_volume_AV	OP	IVW	2	0.93	-0.07	0.74	9.26E-01	9.85E-01
308	26685	ThalamNuclei_lh_volume_Pc	OP	IVW	6	1.13	0.12	0.26	6.44E-01	9.85E-01
309	26686	ThalamNuclei_lh_volume_VLp	OP	IVW	16	1.06	0.06	0.14	6.81E-01	9.85E-01
310	26687	ThalamNuclei_lh_volume_LP	OP	IVW	9	1.14	0.13	0.24	5.70E-01	9.85E-01
311	26688	ThalamNuclei_rh_volume_LGN	OP	IVW	2	1.11	0.1	0.41	8.13E-01	9.85E-01
313	26690	ThalamNuclei_rh_volume_PuI	OP	IVW	8	1.04	0.04	0.27	8.74E-01	9.85E-01
314	26691	ThalamNuclei_rh_volume_PuM	OP	IVW	7	1.15	0.14	0.28	6.15E-01	9.85E-01
316	26693	ThalamNuclei_rh_volume_VPL	OP	IVW	9	1.14	0.13	0.24	5.89E-01	9.85E-01
318	26695	ThalamNuclei_rh_volume_VLa	OP	IVW	14	1.08	0.08	0.19	6.73E-01	9.85E-01
319	26696	ThalamNuclei_rh_volume_PuA	OP	IVW	3	1.06	0.06	0.38	8.71E-01	9.85E-01
322	26699	ThalamNuclei_rh_volume_VAmc	OP	IVW	9	1.17	0.16	0.25	5.20E-01	9.85E-01
323	26700	ThalamNuclei_rh_volume_MDl	OP	IVW	4	1.31	0.27	0.35	4.40E-01	9.85E-01
325	26702	ThalamNuclei_rh_volume_MV(Re)	OP	IVW	8	1.13	0.12	0.20	5.56E-01	9.85E-01
326	26703	ThalamNuclei_rh_volume_CeM	OP	IVW	10	1.12	0.11	0.19	5.72E-01	9.85E-01
327	26704	ThalamNuclei_rh_volume_VM	OP	IVW	10	0.99	-0.01	0.19	9.63E-01	9.85E-01
328	26705	ThalamNuclei_rh_volume_PuL	OP	IVW	3	0.94	-0.06	0.48	9.02E-01	9.85E-01
329	26706	ThalamNuclei_rh_volume_CL	OP	IVW	3	0.70	-0.35	0.56	5.35E-01	9.85E-01
330	26707	ThalamNuclei_rh_volume_VLp	OP	IVW	12	0.87	-0.14	0.17	3.96E-01	9.85E-01
331	26708	ThalamNuclei_rh_volume_Pc	OP	IVW	7	1.03	0.03	0.22	8.79E-01	9.85E-01
333	26710	ThalamNuclei_rh_volume_AV	OP	IVW	2	0.91	-0.09	0.60	8.79E-01	9.85E-01
334	26711	ThalamNuclei_rh_volume_LP	OP	IVW	12	1.09	0.09	0.22	6.75E-01	9.85E-01
335	26712	ThalamNuclei_lh_volume_LD	OP	IVW	11	0.98	-0.02	0.23	9.31E-01	9.85E-01
337	26714	ThalamNuclei_lh_volume_Whole-thalamus	OP	IVW	13	1.13	0.12	0.15	4.13E-01	9.85E-01
338	26715	ThalamNuclei_rh_volume_Whole-thalamus	OP	IVW	11	0.94	-0.06	0.17	7.10E-01	9.85E-01

1002	27607	aparc-a2009s_rh_area_S-oc-middle+Lunatus	OP	IVW	8	0.96	-0.04	0.25	8.64E-01	9.85E-01
26	25782	IDP_T1_FAST_ROIs_L_frontal_pole	OP	IVW	2	0.81	-0.21	0.79	7.94E-01	9.86E-01
29	25785	IDP_T1_FAST_ROIs_R_insular_cortex	OP	IVW	11	0.97	-0.03	0.18	8.54E-01	9.86E-01
33	25789	IDP_T1_FAST_ROIs_R_mid_front_gyrus	OP	IVW	5	0.96	-0.04	0.26	8.60E-01	9.86E-01
35	25791	IDP_T1_FAST_ROIs_R_inf_front_gyrus_parstri	OP	IVW	4	0.90	-0.11	0.28	7.02E-01	9.86E-01
39	25795	IDP_T1_FAST_ROIs_R_precentral_gyrus	OP	IVW	2	1.19	0.17	0.34	6.08E-01	9.86E-01
40	25796	IDP_T1_FAST_ROIs_L_temporal_pole	OP	IVW	2	0.94	-0.06	0.36	8.57E-01	9.86E-01
42	25798	IDP_T1_FAST_ROIs_L_sup_temp_gyrus_ant	OP	IVW	2	0.77	-0.26	0.86	7.64E-01	9.86E-01
44	25800	IDP_T1_FAST_ROIs_L_sup_temp_gyrus_post	OP	WR	1	1.32	0.28	0.61	6.44E-01	9.86E-01
50	25806	IDP_T1_FAST_ROIs_L_mid_temp_gyrus_tempocc	OP	WR	1	1.08	0.08	0.62	8.92E-01	9.86E-01
53	25809	IDP_T1_FAST_ROIs_R_inf_temp_gyrus_ant	OP	IVW	2	1.21	0.19	0.74	7.95E-01	9.86E-01
54	25810	IDP_T1_FAST_ROIs_L_inf_temp_gyrus_post	OP	IVW	2	0.79	-0.23	0.76	7.59E-01	9.86E-01
55	25811	IDP_T1_FAST_ROIs_R_inf_temp_gyrus_post	OP	IVW	2	1.28	0.25	0.60	6.76E-01	9.86E-01
59	25815	IDP_T1_FAST_ROIs_R_postcent_gyrus	OP	IVW	4	0.93	-0.07	0.30	8.08E-01	9.86E-01
60	25816	IDP_T1_FAST_ROIs_L_sup_parietal_lobule	OP	IVW	2	1.11	0.1	0.58	8.58E-01	9.86E-01
61	25817	IDP_T1_FAST_ROIs_R_sup_parietal_lobule	OP	IVW	4	0.87	-0.14	0.28	6.11E-01	9.86E-01
64	25820	IDP_T1_FAST_ROIs_L_supramarg_gyrus_post	OP	IVW	2	0.77	-0.26	0.74	7.28E-01	9.86E-01
66	25822	IDP_T1_FAST_ROIs_L_angular_gyrus	OP	IVW	4	1.09	0.09	0.33	7.94E-01	9.86E-01
73	25829	IDP_T1_FAST_ROIs_R_intracalc_cortex	OP	IVW	24	1.00	0	0.13	9.77E-01	9.86E-01
80	25836	IDP_T1_FAST_ROIs_L_paracing_gyrus	OP	IVW	4	0.83	-0.19	0.47	6.93E-01	9.86E-01
82	25838	IDP_T1_FAST_ROIs_L_cing_gyrus_ant	OP	WR	1	1.08	0.08	0.54	8.77E-01	9.86E-01
88	25844	IDP_T1_FAST_ROIs_L_cuneal_cortex	OP	IVW	3	0.73	-0.31	0.75	6.85E-01	9.86E-01
89	25845	IDP_T1_FAST_ROIs_R_cuneal_cortex	OP	IVW	5	1.02	0.02	0.52	9.76E-01	9.86E-01
95	25851	IDP_T1_FAST_ROIs_R_parahipp_gyrus_post	OP	IVW	10	0.89	-0.12	0.25	6.39E-01	9.86E-01
101	25857	IDP_T1_FAST_ROIs_R_temp_fusif_cortex_post	OP	IVW	4	1.12	0.11	0.39	7.85E-01	9.86E-01
103	25859	IDP_T1_FAST_ROIs_R_temp_occ_fusif_cortex	OP	IVW	4	0.96	-0.04	0.44	9.22E-01	9.86E-01
104	25860	IDP_T1_FAST_ROIs_L_occ_fusif_gyrus	OP	IVW	7	0.99	-0.01	0.33	9.70E-01	9.86E-01
105	25861	IDP_T1_FAST_ROIs_R_occ_fusif_gyrus	OP	IVW	2	1.19	0.17	0.46	7.13E-01	9.86E-01
106	25862	IDP_T1_FAST_ROIs_L_front_operc_cortex	OP	IVW	4	0.80	-0.22	0.42	6.05E-01	9.86E-01
107	25863	IDP_T1_FAST_ROIs_R_front_operc_cortex	OP	IVW	2	0.98	-0.02	0.85	9.80E-01	9.86E-01
111	25867	IDP_T1_FAST_ROIs_R_parietal_operc_cortex	OP	IVW	2	0.72	-0.33	0.86	7.03E-01	9.86E-01
112	25868	IDP_T1_FAST_ROIs_L_planum_polare	OP	IVW	5	0.98	-0.02	0.30	9.37E-01	9.86E-01
118	25874	IDP_T1_FAST_ROIs_L_supracalc_cortex	OP	IVW	6	1.09	0.09	0.39	8.15E-01	9.86E-01

119	25875	IDP_T1_FAST_ROIs_R_supracalc_cortex	OP	IVW	7	1.15	0.14	0.41	7.40E-01	9.86E-01
121	25877	IDP_T1_FAST_ROIs_R_occ_pole	OP	IVW	14	1.00	0	0.17	9.85E-01	9.86E-01
122	25878	IDP_T1_FAST_ROIs_L_thalamus	OP	IVW	16	0.93	-0.07	0.14	6.26E-01	9.86E-01
124	25880	IDP_T1_FAST_ROIs_L_caudate	OP	IVW	19	0.95	-0.05	0.17	7.61E-01	9.86E-01
125	25881	IDP_T1_FAST_ROIs_R_caudate	OP	IVW	16	0.93	-0.07	0.21	7.27E-01	9.86E-01
128	25884	IDP_T1_FAST_ROIs_L_pallidum	OP	IVW	5	0.89	-0.12	0.28	6.67E-01	9.86E-01
130	25886	IDP_T1_FAST_ROIs_L_hippocampus	OP	IVW	13	0.93	-0.07	0.17	6.66E-01	9.86E-01
131	25887	IDP_T1_FAST_ROIs_R_hippocampus	OP	IVW	16	0.97	-0.03	0.13	8.11E-01	9.86E-01
134	25890	IDP_T1_FAST_ROIs_L_ventral_striatum	OP	IVW	12	1.02	0.02	0.20	9.24E-01	9.86E-01
135	25891	IDP_T1_FAST_ROIs_R_ventral_striatum	OP	IVW	18	1.00	0	0.14	9.86E-01	9.86E-01
138	25894	IDP_T1_FAST_ROIs_R_cerebellum_I-IV	OP	IVW	11	0.95	-0.05	0.22	8.09E-01	9.86E-01
139	25895	IDP_T1_FAST_ROIs_L_cerebellum_V	OP	IVW	13	0.97	-0.03	0.19	8.96E-01	9.86E-01
144	25900	IDP_T1_FAST_ROIs_L_cerebellum_crus_I	OP	IVW	32	1.00	0	0.12	9.80E-01	9.86E-01
145	25901	IDP_T1_FAST_ROIs_V_cerebellum_crus_I	OP	WR	1	1.12	0.11	0.81	8.88E-01	9.86E-01
146	25902	IDP_T1_FAST_ROIs_R_cerebellum_crus_I	OP	IVW	26	1.04	0.04	0.11	7.45E-01	9.86E-01
147	25903	IDP_T1_FAST_ROIs_L_cerebellum_crus_II	OP	IVW	23	1.02	0.02	0.11	8.59E-01	9.86E-01
150	25906	IDP_T1_FAST_ROIs_L_cerebellum_VIIb	OP	IVW	24	1.04	0.04	0.11	7.34E-01	9.86E-01
152	25908	IDP_T1_FAST_ROIs_R_cerebellum_VIIb	OP	IVW	25	1.05	0.05	0.11	6.66E-01	9.86E-01
156	25912	IDP_T1_FAST_ROIs_L_cerebellum_VIIIb	OP	IVW	11	1.05	0.05	0.18	7.88E-01	9.86E-01
159	25915	IDP_T1_FAST_ROIs_L_cerebellum_IX	OP	IVW	24	1.01	0.01	0.12	9.29E-01	9.86E-01
160	25916	IDP_T1_FAST_ROIs_V_cerebellum_IX	OP	IVW	18	1.00	0	0.14	9.76E-01	9.86E-01
163	25919	IDP_T1_FAST_ROIs_V_cerebellum_X	OP	IVW	10	0.97	-0.03	0.25	9.20E-01	9.86E-01
164	25920	IDP_T1_FAST_ROIs_R_cerebellum_X	OP	IVW	7	0.99	-0.01	0.25	9.78E-01	9.86E-01
1030	26765	aparc-Desikan_lh_thickness_lateraloccipital	OP	IVW	5	0.95	-0.05	0.33	8.91E-01	9.86E-01
1129	27187	aparc-DKTatlas_lh_thickness_parahippocampal	OP	IVW	3	1.08	0.08	0.62	9.02E-01	9.86E-01
1150	27270	aparc-DKTatlas_rh_thickness_entorhinal	OP	WR	1	0.91	-0.09	0.66	8.97E-01	9.86E-01
393	26906	aparc-Desikan_rh_volume_parsopercularis	OP	IVW	2	0.74	-0.3	0.50	5.55E-01	9.86E-01
395	26908	aparc-Desikan_rh_volume_parstriangularis	OP	IVW	4	0.83	-0.19	0.30	5.22E-01	9.86E-01
403	26916	aparc-Desikan_rh_volume_superiorfrontal	OP	IVW	5	0.84	-0.18	0.31	5.57E-01	9.86E-01
457	27224	aparc-DKTatlas_lh_volume_postcentral	OP	IVW	2	1.21	0.19	0.34	5.64E-01	9.86E-01
481	27310	aparc-DKTatlas_rh_volume_middletemporal	OP	IVW	5	1.23	0.21	0.35	5.51E-01	9.86E-01
490	27319	aparc-DKTatlas_rh_volume_precentral	OP	IVW	3	0.83	-0.19	0.31	5.34E-01	9.86E-01
493	27322	aparc-DKTatlas_rh_volume_rostralmiddlefrontal	OP	IVW	5	0.86	-0.15	0.24	5.51E-01	9.86E-01

349	26794	aparc-Desikan_lh_volume_fusiform	OP	WR	1	1.34	0.29	1.11	7.95E-01	9.87E-01
353	26798	aparc-Desikan_lh_volume_lateraloccipital	OP	IVW	8	0.95	-0.05	0.22	8.16E-01	9.87E-01
354	26799	aparc-Desikan_lh_volume_lateralorbitofrontal	OP	IVW	7	0.95	-0.05	0.22	8.08E-01	9.87E-01
355	26800	aparc-Desikan_lh_volume_lingual	OP	IVW	12	1.00	0	0.17	9.87E-01	9.87E-01
358	26803	aparc-Desikan_lh_volume_parahippocampal	OP	IVW	4	1.08	0.08	0.30	7.94E-01	9.87E-01
360	26805	aparc-Desikan_lh_volume_parsopercularis	OP	WR	1	0.94	-0.06	0.47	9.03E-01	9.87E-01
362	26807	aparc-Desikan_lh_volume_parstriangularis	OP	IVW	6	1.02	0.02	0.23	9.45E-01	9.87E-01
364	26809	aparc-Desikan_lh_volume_postcentral	OP	IVW	9	1.01	0.01	0.33	9.77E-01	9.87E-01
370	26815	aparc-Desikan_lh_volume_superiorfrontal	OP	IVW	7	1.11	0.1	0.21	6.40E-01	9.87E-01
375	26820	aparc-Desikan_lh_volume_transversetemporal	OP	IVW	2	0.88	-0.13	0.43	7.61E-01	9.87E-01
378	26891	aparc-Desikan_rh_volume_caudalanteriorcingulate	OP	IVW	2	0.84	-0.18	0.43	6.79E-01	9.87E-01
379	26892	aparc-Desikan_rh_volume_caudalmiddlefrontal	OP	IVW	2	0.82	-0.2	0.86	8.14E-01	9.87E-01
386	26899	aparc-Desikan_rh_volume_lateraloccipital	OP	IVW	7	0.98	-0.02	0.25	9.23E-01	9.87E-01
387	26900	aparc-Desikan_rh_volume_lateralorbitofrontal	OP	IVW	8	1.11	0.1	0.20	6.30E-01	9.87E-01
389	26902	aparc-Desikan_rh_volume_medialorbitofrontal	OP	IVW	4	1.05	0.05	0.30	8.59E-01	9.87E-01
390	26903	aparc-Desikan_rh_volume_middletemporal	OP	IVW	3	1.11	0.1	0.78	8.94E-01	9.87E-01
391	26904	aparc-Desikan_rh_volume_parahippocampal	OP	IVW	4	1.14	0.13	0.36	7.19E-01	9.87E-01
396	26909	aparc-Desikan_rh_volume_pericalcarine	OP	IVW	20	1.03	0.03	0.12	8.06E-01	9.87E-01
398	26911	aparc-Desikan_rh_volume_posteriorcingulate	OP	WR	1	1.31	0.27	0.59	6.52E-01	9.87E-01
399	26912	aparc-Desikan_rh_volume_precentral	OP	IVW	4	0.93	-0.07	0.27	7.98E-01	9.87E-01
400	26913	aparc-Desikan_rh_volume_precuneus	OP	IVW	11	1.03	0.03	0.21	8.76E-01	9.87E-01
405	26918	aparc-Desikan_rh_volume_superiortemporal	OP	IVW	4	1.01	0.01	0.33	9.78E-01	9.87E-01
406	26919	aparc-Desikan_rh_volume_supramarginal	OP	IVW	5	0.86	-0.15	0.27	5.74E-01	9.87E-01
407	26920	aparc-Desikan_rh_volume_frontalpole	OP	WR	1	1.04	0.04	0.59	9.43E-01	9.87E-01
442	27209	aparc-DKTatlas_lh_volume_fusiform	OP	IVW	2	0.97	-0.03	0.51	9.53E-01	9.87E-01
443	27210	aparc-DKTatlas_lh_volume_inferiorparietal	OP	IVW	5	0.96	-0.04	0.36	9.20E-01	9.87E-01
446	27213	aparc-DKTatlas_lh_volume_lateraloccipital	OP	IVW	8	0.92	-0.08	0.23	7.38E-01	9.87E-01
449	27216	aparc-DKTatlas_lh_volume_medialorbitofrontal	OP	IVW	2	1.20	0.18	0.47	7.07E-01	9.87E-01
451	27218	aparc-DKTatlas_lh_volume_parahippocampal	OP	IVW	2	0.89	-0.12	0.38	7.62E-01	9.87E-01
453	27220	aparc-DKTatlas_lh_volume_parsopercularis	OP	IVW	2	0.86	-0.15	0.37	6.85E-01	9.87E-01
455	27222	aparc-DKTatlas_lh_volume_parstriangularis	OP	IVW	4	1.06	0.06	0.26	8.22E-01	9.87E-01
458	27225	aparc-DKTatlas_lh_volume_posteriorcingulate	OP	WR	1	0.89	-0.12	0.65	8.48E-01	9.87E-01
460	27227	aparc-DKTatlas_lh_volume_precuneus	OP	IVW	6	1.12	0.11	0.30	7.11E-01	9.87E-01

461	27228	aparc-DKTatlas_lh_volume_rostralanteriorcingulate	OP	IVW	3	0.82	-0.2	0.46	6.60E-01	9.87E-01
463	27230	aparc-DKTatlas_lh_volume_superiorfrontal	OP	IVW	3	0.90	-0.1	0.41	8.01E-01	9.87E-01
464	27231	aparc-DKTatlas_lh_volume_superiorparietal	OP	IVW	16	0.95	-0.05	0.16	7.61E-01	9.87E-01
467	27234	aparc-DKTatlas_lh_volume_transversetemporal	OP	IVW	4	1.13	0.12	0.33	7.20E-01	9.87E-01
470	27299	aparc-DKTatlas_rh_volume_caudalmiddlefrontal	OP	IVW	2	0.82	-0.2	0.85	8.14E-01	9.87E-01
474	27303	aparc-DKTatlas_rh_volume_inferiorparietal	OP	IVW	7	1.06	0.06	0.25	8.04E-01	9.87E-01
477	27306	aparc-DKTatlas_rh_volume_lateraloccipital	OP	IVW	7	0.97	-0.03	0.27	8.96E-01	9.87E-01
478	27307	aparc-DKTatlas_rh_volume_lateralorbitofrontal	OP	IVW	6	0.95	-0.05	0.23	8.35E-01	9.87E-01
482	27311	aparc-DKTatlas_rh_volume_parahippocampal	OP	IVW	3	1.01	0.01	0.53	9.87E-01	9.87E-01
484	27313	aparc-DKTatlas_rh_volume_parsopercularis	OP	IVW	3	0.87	-0.14	0.38	7.10E-01	9.87E-01
486	27315	aparc-DKTatlas_rh_volume_parstriangularis	OP	IVW	4	0.90	-0.1	0.27	7.15E-01	9.87E-01
491	27320	aparc-DKTatlas_rh_volume_precuneus	OP	IVW	10	1.06	0.06	0.22	7.76E-01	9.87E-01
492	27321	aparc-DKTatlas_rh_volume_rostralanteriorcingulate	OP	WR	1	0.88	-0.13	0.58	8.22E-01	9.87E-01
494	27323	aparc-DKTatlas_rh_volume_superiorfrontal	OP	IVW	4	0.99	-0.01	0.34	9.67E-01	9.87E-01
496	27325	aparc-DKTatlas_rh_volume_superiortemporal	OP	IVW	4	1.01	0.01	0.35	9.75E-01	9.87E-01
497	27326	aparc-DKTatlas_rh_volume_supramarginal	OP	IVW	6	0.94	-0.06	0.24	8.08E-01	9.87E-01
798	27103	BA-exvivo_rh_area_BA3a	OP	IVW	6	1.00	0	0.31	9.88E-01	9.88E-01
802	27107	BA-exvivo_rh_area_BA6	OP	IVW	5	0.99	-0.01	0.34	9.82E-01	9.88E-01
874	27331	aparc-a2009s_lh_area_G+S-paracentral	OP	IVW	4	1.03	0.03	0.30	9.21E-01	9.89E-01
879	27336	aparc-a2009s_lh_area_G+S-cingul-Mid-Post	OP	IVW	2	1.04	0.04	0.45	9.34E-01	9.89E-01
885	27342	aparc-a2009s_lh_area_G-front-inf-Triangul	OP	IVW	3	0.96	-0.04	0.36	9.20E-01	9.89E-01
888	27345	aparc-a2009s_lh_area_G-Ins-lg+S-cent-ins	OP	WR	1	1.02	0.02	0.65	9.79E-01	9.89E-01
892	27349	aparc-a2009s_lh_area_G-oc-temp-lat-fusifor	OP	IVW	4	0.98	-0.02	0.30	9.52E-01	9.89E-01
937	27394	aparc-a2009s_lh_area_S-pericallosal	OP	IVW	4	1.04	0.04	0.37	9.20E-01	9.89E-01
939	27396	aparc-a2009s_lh_area_S-precentral-inf-part	OP	WR	1	1.02	0.02	0.48	9.69E-01	9.89E-01
965	27570	aparc-a2009s_rh_area_G-occipital-sup	OP	IVW	6	0.97	-0.03	0.26	8.96E-01	9.89E-01
966	27571	aparc-a2009s_rh_area_G-oc-temp-lat-fusifor	OP	IVW	2	1.03	0.03	0.52	9.58E-01	9.89E-01
985	27590	aparc-a2009s_rh_area_Lat-Fis-ant-Vertical	OP	WR	1	0.94	-0.06	0.50	9.03E-01	9.89E-01
987	27592	aparc-a2009s_rh_area_Pole-occipital	OP	IVW	15	0.99	-0.01	0.16	9.50E-01	9.89E-01
988	27593	aparc-a2009s_rh_area_Pole-temporal	OP	IVW	6	1.01	0.01	0.31	9.81E-01	9.89E-01
993	27598	aparc-a2009s_rh_area_S-circular-insula-inf	OP	IVW	2	1.12	0.11	1.26	9.29E-01	9.89E-01
1014	27619	aparc-a2009s_rh_area_S-precentral-sup-part	OP	WR	1	1.04	0.04	0.84	9.58E-01	9.89E-01
1016	27621	aparc-a2009s_rh_area_S-subparietal	OP	IVW	5	1.01	0.01	0.29	9.65E-01	9.89E-01

557	27534	aparc-a2009s_lh_volume_S-oc-sup+transversal	OP	WR	1	1.25	0.22	0.60	7.17E-01	9.89E-01
572	27549	aparc-a2009s_lh_volume_S-temporal-sup	OP	IVW	7	1.09	0.09	0.27	7.32E-01	9.89E-01
631	27756	aparc-a2009s_rh_volume_S-oc-sup+transversal	OP	WR	1	0.83	-0.19	0.55	7.31E-01	9.89E-01
549	27526	aparc-a2009s_lh_volume_S-collat-transv-ant	OP	IVW	3	0.90	-0.1	0.31	7.42E-01	9.90E-01
637	27762	aparc-a2009s_rh_volume_S-orbital-H-Shaped	OP	IVW	5	0.92	-0.08	0.26	7.49E-01	9.90E-01
653	26726	aparc-Desikan_lh_area_entorhinal	OP	IVW	4	1.00	0	0.26	9.85E-01	9.90E-01
47	25803	IDP_T1_FAST_ROIs_R_mid_temp_gyrus_ant	OP	WR	1	0.88	-0.13	0.60	8.22E-01	9.92E-01
52	25808	IDP_T1_FAST_ROIs_L_inf_temp_gyrus_ant	OP	WR	1	0.88	-0.13	0.57	8.22E-01	9.92E-01
351	26796	aparc-Desikan_lh_volume_inferiortemporal	OP	WR	1	0.98	-0.02	0.56	9.67E-01	9.95E-01
444	27211	aparc-DKTatlas_lh_volume_inferiortemporal	OP	WR	1	0.98	-0.02	0.57	9.67E-01	9.95E-01
287	26664	ThalamNuclei_lh_volume_MGN	OP	IVW	3	1.00	0	0.45	9.95E-01	9.95E-01
285	26662	HippSubfield_rh_volume_Whole-hippocampal-head	OP	IVW	9	1.00	0	0.19	9.97E-01	9.97E-01
934	27391	aparc-a2009s_lh_area_S-orbital-med-olfact	OP	IVW	12	1.00	0	0.18	9.98E-01	9.98E-01
868	27263	aparc-DKTatlas_rh_area_superiortemporal	OP	IVW	4	1.00	0	0.24	9.98E-01	9.98E-01
1037	26772	aparc-Desikan_lh_thickness_parsopercularis	OP	IVW	6	1.00	0	0.22	9.96E-01	1.00E+00
1050	26785	aparc-Desikan_lh_thickness_supramarginal	OP	IVW	4	0.98	-0.02	0.32	9.43E-01	1.00E+00
1061	26863	aparc-Desikan_rh_thickness_inferiorparietal	OP	IVW	5	1.02	0.02	0.28	9.37E-01	1.00E+00
1069	26871	aparc-Desikan_rh_thickness_parahippocampal	OP	IVW	3	0.96	-0.04	0.57	9.38E-01	1.00E+00
1081	26883	aparc-Desikan_rh_thickness_superiorfrontal	OP	IVW	4	1.02	0.02	0.30	9.47E-01	1.00E+00
1084	26886	aparc-Desikan_rh_thickness_supramarginal	OP	IVW	4	1.00	0	0.37	9.95E-01	1.00E+00
1117	27175	aparc-DKTatlas_lh_thickness_caudalmiddlefrontal	OP	IVW	2	0.99	-0.01	0.31	9.61E-01	1.00E+00
1124	27182	aparc-DKTatlas_lh_thickness_lateraloccipital	OP	IVW	6	1.01	0.01	0.30	9.78E-01	1.00E+00
1151	27271	aparc-DKTatlas_rh_thickness_fusiform	OP	IVW	3	0.99	-0.01	0.70	9.88E-01	1.00E+00
1160	27280	aparc-DKTatlas_rh_thickness_parahippocampal	OP	IVW	3	1.00	0	0.57	1.00E+00	1.00E+00
1167	27287	aparc-DKTatlas_rh_thickness_posteriorcingulate	OP	WR	1	1.01	0.01	0.48	9.79E-01	1.00E+00
430	27135	BA-exvivo_rh_volume_BA6	OP	IVW	6	1.00	0	0.33	1.00E+00	1.00E+00
501	27478	aparc-a2009s_lh_volume_G+S-occipital-inf	OP	WR	1	1.00	0	0.58	1.00E+00	1.00E+00
521	27498	aparc-a2009s_lh_volume_G-oc-temp-med-Lingual	OP	IVW	9	0.98	-0.02	0.20	9.01E-01	1.00E+00
522	27499	aparc-a2009s_lh_volume_G-oc-temp-med-Parahip	OP	IVW	5	1.00	0	0.20	9.82E-01	1.00E+00
527	27504	aparc-a2009s_lh_volume_G-postcentral	OP	IVW	6	0.94	-0.06	0.26	8.12E-01	1.00E+00
528	27505	aparc-a2009s_lh_volume_G-precentral	OP	IVW	5	1.00	0	0.23	9.97E-01	1.00E+00
546	27523	aparc-a2009s_lh_volume_S-circular-insula-ant	OP	IVW	2	1.03	0.03	0.54	9.55E-01	1.00E+00
551	27528	aparc-a2009s_lh_volume_S-front-inf	OP	IVW	2	1.12	0.11	0.58	8.50E-01	1.00E+00

556	27533	aparc-a2009s_lh_volume_S-oc-middle+Lunatus	OP	IVW	6	1.00	0	0.23	9.88E-01	1.00E+00
561	27538	aparc-a2009s_lh_volume_S-orbital-lateral	OP	WR	1	1.09	0.09	0.61	8.83E-01	1.00E+00
562	27539	aparc-a2009s_lh_volume_S-orbital-med-olfact	OP	IVW	2	1.07	0.07	0.36	8.39E-01	1.00E+00
576	27701	aparc-a2009s_rh_volume_G+S-paracentral	OP	WR	1	0.73	-0.32	1.28	8.03E-01	1.00E+00
589	27714	aparc-a2009s_rh_volume_G-front-sup	OP	IVW	5	1.07	0.07	0.31	8.24E-01	1.00E+00
597	27722	aparc-a2009s_rh_volume_G-orbital	OP	IVW	2	0.80	-0.22	0.74	7.69E-01	1.00E+00
599	27724	aparc-a2009s_rh_volume_G-pariet-inf-Supramar	OP	IVW	3	1.03	0.03	0.53	9.61E-01	1.00E+00
603	27728	aparc-a2009s_rh_volume_G-precuneus	OP	IVW	8	1.01	0.01	0.20	9.74E-01	1.00E+00
607	27732	aparc-a2009s_rh_volume_G-temp-sup-Lateral	OP	IVW	2	1.07	0.07	0.39	8.54E-01	1.00E+00
613	27738	aparc-a2009s_rh_volume_Lat-Fis-ant-Vertical	OP	WR	1	0.94	-0.06	0.50	9.03E-01	1.00E+00
616	27741	aparc-a2009s_rh_volume_Pole-temporal	OP	WR	1	0.94	-0.06	0.58	9.17E-01	1.00E+00
623	27748	aparc-a2009s_rh_volume_S-collat-transv-ant	OP	WR	1	1.06	0.06	0.53	9.12E-01	1.00E+00
625	27750	aparc-a2009s_rh_volume_S-front-inf	OP	WR	1	0.98	-0.02	0.43	9.69E-01	1.00E+00
626	27751	aparc-a2009s_rh_volume_S-front-middle	OP	WR	1	0.99	-0.01	0.61	9.92E-01	1.00E+00
629	27754	aparc-a2009s_rh_volume_S-intrapariet+P-trans	OP	WR	1	0.96	-0.04	0.75	9.58E-01	1.00E+00
630	27755	aparc-a2009s_rh_volume_S-oc-middle+Lunatus	OP	IVW	3	0.98	-0.02	0.34	9.56E-01	1.00E+00
636	27761	aparc-a2009s_rh_volume_S-orbital-med-olfact	OP	IVW	4	0.93	-0.07	0.29	8.00E-01	1.00E+00
639	27764	aparc-a2009s_rh_volume_S-pericallosal	OP	WR	1	1.22	0.2	0.80	8.03E-01	1.00E+00
641	27766	aparc-a2009s_rh_volume_S-precentral-inf-part	OP	WR	1	0.89	-0.12	0.60	8.40E-01	1.00E+00
646	27771	aparc-a2009s_rh_volume_S-temporal-sup	OP	IVW	4	1.13	0.12	0.46	7.92E-01	1.00E+00
525	27502	aparc-a2009s_lh_volume_G-pariet-inf-Supramar	OP	WR	1	1.07	0.07	0.45	8.83E-01	1.01E+00
642	27767	aparc-a2009s_rh_volume_S-precentral-sup-part	OP	WR	1	0.94	-0.06	0.43	8.83E-01	1.01E+00
