# Supplementary material for: Causal associations of brain structure with bone mineral density: a large-scale genetic correlation study
Source: Bone Res. 2023 Jul 20;11:37. doi: 10.1038/s41413-023-00270-z (PMC10359275; doi:10.1038/s41413-023-00270-z)
Supplement: Supplementary file 8 — Supplementary Table 6. The UKB IDs and descriptions of the 1325 BIDPs [file 41413_2023_270_MOESM8_ESM.pdf]

Supplemental table 6. The UKB IDs and descriptions of the 1325 BIDPs

No.	UKB ID	IDP short name	Category name	Category group	Units	IDP description	Heritability	Heritability(SE)
1	25001	IDP_T1_SIENAX_peripheral_grey_normalised_volume	IDP T1:global	regional and tissue volume	mm3	Volume of peripheral cortical grey matter (from T1 brain image, normalised for head size)	2.59E-01	2.21E-02
2	25002	IDP_T1_SIENAX_peripheral_grey_unnormalised_volume	IDP T1:global	regional and tissue volume	mm3	Volume of peripheral cortical grey matter (from T1 brain image)	2.57E-01	2.17E-02
3	25003	IDP_T1_SIENAX_CSF_normalised_volume	IDP T1:global	regional and tissue volume	mm3	Volume of ventricular cerebrospinal fluid (from T1 brain image, normalised for head size)	2.88E-01	2.38E-02
4	25004	IDP_T1_SIENAX_CSF_unnormalised_volume	IDP T1:global	regional and tissue volume	mm3	Volume of ventricular cerebrospinal fluid (from T1 brain image)	2.88E-01	2.39E-02
5	25005	IDP_T1_SIENAX_grey_normalised_volume	IDP T1:global	regional and tissue volume	mm3	Volume of grey matter (from T1 brain image, normalised for head size)	2.31E-01	2.19E-02
6	25006	IDP_T1_SIENAX_grey_unnormalised_volume	IDP T1:global	regional and tissue volume	mm3	Volume of grey matter (from T1 brain image)	2.27E-01	2.14E-02
7	25007	IDP_T1_SIENAX_white_normalised_volume	IDP T1:global	regional and tissue volume	mm3	Volume of white matter (from T1 brain image, normalised for head size)	2.91E-01	2.53E-02
8	25008	IDP_T1_SIENAX_white_unnormalised_volume	IDP T1:global	regional and tissue volume	mm3	Volume of white matter (from T1 brain image)	2.89E-01	2.55E-02
9	25009	IDP_T1_SIENAX_brain-normalised_volume	IDP T1:global	regional and tissue volume	mm3	Volume of brain, grey+white, from T1 brain image, normalised for head size	2.51E-01	2.42E-02
10	25010	IDP_T1_SIENAX_brain-unnormalised_volume	IDP T1:global	regional and tissue volume	mm3	Volume of brain, grey+white, from T1 brain image	2.49E-01	2.40E-02
11	25011	IDP_T1_FIRST_left_thalamus_volume	IDP T1:global	regional and tissue volume	mm3	Volume of left thalamus (from T1 brain image)	2.57E-01	2.38E-02
12	25012	IDP_T1_FIRST_right_thalamus_volume	IDP T1:global	regional and tissue volume	mm3	Volume of right thalamus (from T1 brain image)	2.60E-01	2.33E-02
13	25013	IDP_T1_FIRST_left_caudate_volume	IDP T1:global	regional and tissue volume	mm3	Volume of left caudate (from T1 brain image)	3.45E-01	2.56E-02
14	25014	IDP_T1_FIRST_right_caudate_volume	IDP T1:global	regional and tissue volume	mm3	Volume of right caudate (from T1 brain image)	3.47E-01	2.60E-02

15	25015	IDP_T1_FIRST_left_putamen_volume	IDP T1:global	regional and tissue volume	mm3	Volume of left putamen (from T1 brain image)	2.53E-01	2.45E-02
16	25016	IDP_T1_FIRST_right_putamen_volume	IDP T1:global	regional and tissue volume	mm3	Volume of right putamen (from T1 brain image)	2.88E-01	2.60E-02
17	25017	IDP_T1_FIRST_left_pallidum_volume	IDP T1:global	regional and tissue volume	mm3	Volume of left pallidum (from T1 brain image)	2.14E-01	2.18E-02
18	25018	IDP_T1_FIRST_right_pallidum_volume	IDP T1:global	regional and tissue volume	mm3	Volume of right pallidum (from T1 brain image)	2.31E-01	2.30E-02
19	25019	IDP_T1_FIRST_left_hippocampus_volume	IDP T1:global	regional and tissue volume	mm3	Volume of left hippocampus (from T1 brain image)	1.71E-01	1.89E-02
20	25020	IDP_T1_FIRST_right_hippocampus_volume	IDP T1:global	regional and tissue volume	mm3	Volume of right hippocampus (from T1 brain image)	1.67E-01	1.96E-02
21	25021	IDP_T1_FIRST_left_amygdala_volume	IDP T1:global	regional and tissue volume	mm3	Volume of left amygdala (from T1 brain image)	1.09E-01	1.58E-02
22	25022	IDP_T1_FIRST_right_amygdala_volume	IDP T1:global	regional and tissue volume	mm3	Volume of right amygdala (from T1 brain image)	9.61E-02	1.70E-02
23	25023	IDP_T1_FIRST_left_accumbens_volume	IDP T1:global	regional and tissue volume	mm3	Volume of left accumbens (from T1 brain image)	1.51E-01	1.63E-02
24	25024	IDP_T1_FIRST_right_accumbens_volume	IDP T1:global	regional and tissue volume	mm3	Volume of right accumbens (from T1 brain image)	1.78E-01	1.85E-02
25	25025	IDP_T1_FIRST_brain_stem+4th_ventricle_volume	IDP T1:global	regional and tissue volume	mm3	Volume of brain stem + 4th ventricle (from T1 brain image)	2.77E-01	2.50E-02
26	25782	IDP_T1_FAST_ROIs_L_frontal_pole	IDP T1:unilateral regions	regional and tissue volume	mm3	Volume of grey matter in Left Frontal Pole	1.53E-01	1.67E-02
27	25783	IDP_T1_FAST_ROIs_R_frontal_pole	IDP T1:unilateral regions	regional and tissue volume	mm3	Volume of grey matter in Right Frontal Pole	2.08E-01	1.84E-02
28	25784	IDP_T1_FAST_ROIs_L_insular_cortex	IDP T1:unilateral regions	regional and tissue volume	mm3	Volume of grey matter in Left Insular Cortex	2.84E-01	2.20E-02
29	25785	IDP_T1_FAST_ROIs_R_insular_cortex	IDP T1:unilateral	regional and tissue volume	mm3	Volume of grey matter in Right Insular Cortex	2.70E-01	2.18E-02

			regions					
30	25786	IDP_T1_FAST_ROIs_L_sup_front_gyrus	IDP T1:unilateral regions	regional and tissue volume	mm3	Volume of grey matter in Left Superior Frontal Gyrus	1.37E-01	1.75E-02
31	25787	IDP_T1_FAST_ROIs_R_sup_front_gyrus	IDP T1:unilateral regions	regional and tissue volume	mm3	Volume of grey matter in Right Superior Frontal Gyrus	1.26E-01	1.66E-02
32	25788	IDP_T1_FAST_ROIs_L_mid_front_gyrus	IDP T1:unilateral regions	regional and tissue volume	mm3	Volume of grey matter in Left Middle Frontal Gyrus	1.57E-01	1.77E-02
33	25789	IDP_T1_FAST_ROIs_R_mid_front_gyrus	IDP T1:unilateral regions	regional and tissue volume	mm3	Volume of grey matter in Right Middle Frontal Gyrus	1.65E-01	1.66E-02
34	25790	IDP_T1_FAST_ROIs_L_inf_front_gyrus_parstri	IDP T1:unilateral regions	regional and tissue volume	mm3	Volume of grey matter in Left Inferior Frontal Gyrus, pars triangularis	1.02E-01	1.61E-02
35	25791	IDP_T1_FAST_ROIs_R_inf_front_gyrus_parstri	IDP T1:unilateral regions	regional and tissue volume	mm3	Volume of grey matter in Right Inferior Frontal Gyrus, pars triangularis	4.77E-02	1.47E-02
36	25792	IDP_T1_FAST_ROIs_L_inf_front_gyrus_parsop	IDP T1:unilateral regions	regional and tissue volume	mm3	Volume of grey matter in Left Inferior Frontal Gyrus, pars opercularis	8.45E-02	2.12E-02
37	25793	IDP_T1_FAST_ROIs_R_inf_front_gyrus_parsop	IDP T1:unilateral regions	regional and tissue volume	mm3	Volume of grey matter in Right Inferior Frontal Gyrus, pars opercularis	7.28E-02	1.45E-02
38	25794	IDP_T1_FAST_ROIs_L_precentral_gyrus	IDP T1:unilateral regions	regional and tissue volume	mm3	Volume of grey matter in Left Precentral Gyrus	1.47E-01	1.77E-02
39	25795	IDP_T1_FAST_ROIs_R_precentral_gyrus	IDP T1:unilateral regions	regional and tissue volume	mm3	Volume of grey matter in Right Precentral Gyrus	1.61E-01	1.82E-02
40	25796	IDP_T1_FAST_ROIs_L_temporal_pole	IDP	regional and tissue	mm3	Volume of grey matter in Left Temporal Pole	1.98E-01	1.97E-02

			T1:unilateral regions	volume				
41	25797	IDP_T1_FAST_ROIs_R_temporal_pole	IDP T1:unilateral regions	regional and tissue volume	mm3	Volume of grey matter in Right Temporal Pole	1.96E-01	1.87E-02
42	25798	IDP_T1_FAST_ROIs_L_sup_temp_gyrus_ant	IDP T1:unilateral regions	regional and tissue volume	mm3	Volume of grey matter in Left Superior Temporal Gyrus, anterior division	1.36E-01	2.35E-02
43	25799	IDP_T1_FAST_ROIs_R_sup_temp_gyrus_ant	IDP T1:unilateral regions	regional and tissue volume	mm3	Volume of grey matter in Right Superior Temporal Gyrus, anterior division	1.21E-01	1.65E-02
44	25800	IDP_T1_FAST_ROIs_L_sup_temp_gyrus_post	IDP T1:unilateral regions	regional and tissue volume	mm3	Volume of grey matter in Left Superior Temporal Gyrus, posterior division	1.17E-01	1.52E-02
45	25801	IDP_T1_FAST_ROIs_R_sup_temp_gyrus_post	IDP T1:unilateral regions	regional and tissue volume	mm3	Volume of grey matter in Right Superior Temporal Gyrus, posterior division	1.67E-01	1.74E-02
46	25802	IDP_T1_FAST_ROIs_L_mid_temp_gyrus_ant	IDP T1:unilateral regions	regional and tissue volume	mm3	Volume of grey matter in Left Middle Temporal Gyrus, anterior division	1.20E-01	1.66E-02
47	25803	IDP_T1_FAST_ROIs_R_mid_temp_gyrus_ant	IDP T1:unilateral regions	regional and tissue volume	mm3	Volume of grey matter in Right Middle Temporal Gyrus, anterior division	1.48E-01	2.28E-02
48	25804	IDP_T1_FAST_ROIs_L_mid_temp_gyrus_post	IDP T1:unilateral regions	regional and tissue volume	mm3	Volume of grey matter in Left Middle Temporal Gyrus, posterior division	1.61E-01	1.88E-02
49	25805	IDP_T1_FAST_ROIs_R_mid_temp_gyrus_post	IDP T1:unilateral regions	regional and tissue volume	mm3	Volume of grey matter in Right Middle Temporal Gyrus, posterior division	1.56E-01	1.99E-02
50	25806	IDP_T1_FAST_ROIs_L_mid_temp_gyrus_temp occ	IDP T1:unilateral regions	regional and tissue volume	mm3	Volume of grey matter in Left Middle Temporal Gyrus, temporooccipital part	8.30E-02	1.66E-02

51	25807	IDP_T1_FAST_ROIs_R_mid_temp_gyrus_temp occ	IDP T1:unilateral regions	regional and tissue volume	mm3	Volume of grey matter in Right Middle Temporal Gyrus, temporooccipital part	1.09E-01	1.61E-02
52	25808	IDP_T1_FAST_ROIs_L_inf_temp_gyrus_ant	IDP T1:unilateral regions	regional and tissue volume	mm3	Volume of grey matter in Left Inferior Temporal Gyrus, anterior division	1.49E-01	1.72E-02
53	25809	IDP_T1_FAST_ROIs_R_inf_temp_gyrus_ant	IDP T1:unilateral regions	regional and tissue volume	mm3	Volume of grey matter in Right Inferior Temporal Gyrus, anterior division	1.50E-01	1.72E-02
54	25810	IDP_T1_FAST_ROIs_L_inf_temp_gyrus_post	IDP T1:unilateral regions	regional and tissue volume	mm3	Volume of grey matter in Left Inferior Temporal Gyrus, posterior division	1.38E-01	2.01E-02
55	25811	IDP_T1_FAST_ROIs_R_inf_temp_gyrus_post	IDP T1:unilateral regions	regional and tissue volume	mm3	Volume of grey matter in Right Inferior Temporal Gyrus, posterior division	1.62E-01	1.67E-02
56	25812	IDP_T1_FAST_ROIs_L_inf_temp_gyrus_tempo cc	IDP T1:unilateral regions	regional and tissue volume	mm3	Volume of grey matter in Left Inferior Temporal Gyrus, temporooccipital part	1.60E-01	1.82E-02
57	25813	IDP_T1_FAST_ROIs_R_inf_temp_gyrus_tempo cc	IDP T1:unilateral regions	regional and tissue volume	mm3	Volume of grey matter in Right Inferior Temporal Gyrus, temporooccipital part	1.58E-01	1.78E-02
58	25814	IDP_T1_FAST_ROIs_L_postcent_gyrus	IDP T1:unilateral regions	regional and tissue volume	mm3	Volume of grey matter in Left Postcentral Gyrus	1.31E-01	1.62E-02
59	25815	IDP_T1_FAST_ROIs_R_postcent_gyrus	IDP T1:unilateral regions	regional and tissue volume	mm3	Volume of grey matter in Right Postcentral Gyrus	1.33E-01	1.77E-02
60	25816	IDP_T1_FAST_ROIs_L_sup_parietal_lobule	IDP T1:unilateral regions	regional and tissue volume	mm3	Volume of grey matter in Left Superior Parietal Lobule	7.85E-02	1.42E-02
61	25817	IDP_T1_FAST_ROIs_R_sup_parietal_lobule	IDP T1:unilateral regions	regional and tissue volume	mm3	Volume of grey matter in Right Superior Parietal Lobule	5.74E-02	1.43E-02

62	25818	IDP_T1_FAST_ROIs_L_supramarg_gyrus_ant	IDP T1:unilateral regions	regional and tissue volume	mm3	Volume of grey matter in Left Supramarginal Gyrus, anterior division	8.82E-02	1.70E-02
63	25819	IDP_T1_FAST_ROIs_R_supramarg_gyrus_ant	IDP T1:unilateral regions	regional and tissue volume	mm3	Volume of grey matter in Right Supramarginal Gyrus, anterior division	9.48E-02	1.66E-02
64	25820	IDP_T1_FAST_ROIs_L_supramarg_gyrus_post	IDP T1:unilateral regions	regional and tissue volume	mm3	Volume of grey matter in Left Supramarginal Gyrus, posterior division	9.43E-02	1.50E-02
65	25821	IDP_T1_FAST_ROIs_R_supramarg_gyrus_post	IDP T1:unilateral regions	regional and tissue volume	mm3	Volume of grey matter in Right Supramarginal Gyrus, posterior division	6.20E-02	1.50E-02
66	25822	IDP_T1_FAST_ROIs_L_angular_gyrus	IDP T1:unilateral regions	regional and tissue volume	mm3	Volume of grey matter in Left Angular Gyrus	1.30E-01	1.75E-02
67	25823	IDP_T1_FAST_ROIs_R_angular_gyrus	IDP T1:unilateral regions	regional and tissue volume	mm3	Volume of grey matter in Right Angular Gyrus	1.07E-01	1.59E-02
68	25824	IDP_T1_FAST_ROIs_L_latocc_cortex_sup	IDP T1:unilateral regions	regional and tissue volume	mm3	Volume of grey matter in Left Lateral Occipital Cortex, superior division	1.26E-01	1.70E-02
69	25825	IDP_T1_FAST_ROIs_R_latocc_cortex_sup	IDP T1:unilateral regions	regional and tissue volume	mm3	Volume of grey matter in Right Lateral Occipital Cortex, superior division	1.07E-01	1.61E-02
70	25826	IDP_T1_FAST_ROIs_L_latocc_cortex_inf	IDP T1:unilateral regions	regional and tissue volume	mm3	Volume of grey matter in Left Lateral Occipital Cortex, inferior division	1.38E-01	1.83E-02
71	25827	IDP_T1_FAST_ROIs_R_latocc_cortex_inf	IDP T1:unilateral regions	regional and tissue volume	mm3	Volume of grey matter in Right Lateral Occipital Cortex, inferior division	1.05E-01	1.85E-02
72	25828	IDP_T1_FAST_ROIs_L_intracalc_cortex	IDP T1:unilateral regions	regional and tissue volume	mm3	Volume of grey matter in Left Intracalcarine Cortex	3.06E-01	2.61E-02

73	25829	IDP_T1_FAST_ROIs_R_intracalc_cortex	IDP T1:unilateral regions	regional and tissue volume	mm3	Volume of grey matter in Right Intracalcarine Cortex	3.17E-01	2.77E-02
74	25830	IDP_T1_FAST_ROIs_L_front_med_cortex	IDP T1:unilateral regions	regional and tissue volume	mm3	Volume of grey matter in Left Frontal Medial Cortex	1.00E-01	1.45E-02
75	25831	IDP_T1_FAST_ROIs_R_front_med_cortex	IDP T1:unilateral regions	regional and tissue volume	mm3	Volume of grey matter in Right Frontal Medial Cortex	8.23E-02	1.42E-02
76	25832	IDP_T1_FAST_ROIs_L_juxtapos_lobule_cortex	IDP T1:unilateral regions	regional and tissue volume	mm3	Volume of grey matter in Left Juxtapositional Lobule Cortex (formerly Supplementary Motor Cortex)	7.60E-02	1.55E-02
77	25833	IDP_T1_FAST_ROIs_R_juxtapos_lobule_cortex	IDP T1:unilateral regions	regional and tissue volume	mm3	Volume of grey matter in Right Juxtapositional Lobule Cortex (formerly Supplementary Motor Cortex)	6.96E-02	1.52E-02
78	25834	IDP_T1_FAST_ROIs_L_subcallosal_cortex	IDP T1:unilateral regions	regional and tissue volume	mm3	Volume of grey matter in Left Subcallosal Cortex	2.85E-01	2.40E-02
79	25835	IDP_T1_FAST_ROIs_R_subcallosal_cortex	IDP T1:unilateral regions	regional and tissue volume	mm3	Volume of grey matter in Right Subcallosal Cortex	2.76E-01	2.34E-02
80	25836	IDP_T1_FAST_ROIs_L_paracing_gyrus	IDP T1:unilateral regions	regional and tissue volume	mm3	Volume of grey matter in Left Paracingulate Gyrus	1.67E-01	1.75E-02
81	25837	IDP_T1_FAST_ROIs_R_paracing_gyrus	IDP T1:unilateral regions	regional and tissue volume	mm3	Volume of grey matter in Right Paracingulate Gyrus	1.48E-01	1.63E-02
82	25838	IDP_T1_FAST_ROIs_L_cing_gyrus_ant	IDP T1:unilateral regions	regional and tissue volume	mm3	Volume of grey matter in Left Cingulate Gyrus, anterior division	1.51E-01	1.75E-02
83	25839	IDP_T1_FAST_ROIs_R_cing_gyrus_ant	IDP T1:unilateral regions	regional and tissue volume	mm3	Volume of grey matter in Right Cingulate Gyrus, anterior division	1.42E-01	2.05E-02

84	25840	IDP_T1_FAST_ROIs_L_cing_gyrus_post	IDP T1:unilateral regions	regional and tissue volume	mm3	Volume of grey matter in Left Cingulate Gyrus, posterior division	1.67E-01	1.73E-02
85	25841	IDP_T1_FAST_ROIs_R_cing_gyrus_post	IDP T1:unilateral regions	regional and tissue volume	mm3	Volume of grey matter in Right Cingulate Gyrus, posterior division	1.44E-01	1.83E-02
86	25842	IDP_T1_FAST_ROIs_L_precun_cortex	IDP T1:unilateral regions	regional and tissue volume	mm3	Volume of grey matter in Left Precuneous Cortex	2.02E-01	2.15E-02
87	25843	IDP_T1_FAST_ROIs_R_precun_cortex	IDP T1:unilateral regions	regional and tissue volume	mm3	Volume of grey matter in Right Precuneous Cortex	2.05E-01	2.28E-02
88	25844	IDP_T1_FAST_ROIs_L_cuneal_cortex	IDP T1:unilateral regions	regional and tissue volume	mm3	Volume of grey matter in Left Cuneal Cortex	1.58E-01	1.57E-02
89	25845	IDP_T1_FAST_ROIs_R_cuneal_cortex	IDP T1:unilateral regions	regional and tissue volume	mm3	Volume of grey matter in Right Cuneal Cortex	1.51E-01	1.75E-02
90	25846	IDP_T1_FAST_ROIs_L_front_orb_cortex	IDP T1:unilateral regions	regional and tissue volume	mm3	Volume of grey matter in Left Frontal Orbital Cortex	2.01E-01	2.02E-02
91	25847	IDP_T1_FAST_ROIs_R_front_orb_cortex	IDP T1:unilateral regions	regional and tissue volume	mm3	Volume of grey matter in Right Frontal Orbital Cortex	1.94E-01	2.40E-02
92	25848	IDP_T1_FAST_ROIs_L_parahipp_gyrus_ant	IDP T1:unilateral regions	regional and tissue volume	mm3	Volume of grey matter in Left Parahippocampal Gyrus, anterior division	1.87E-01	1.84E-02
93	25849	IDP_T1_FAST_ROIs_R_parahipp_gyrus_ant	IDP T1:unilateral regions	regional and tissue volume	mm3	Volume of grey matter in Right Parahippocampal Gyrus, anterior division	1.98E-01	1.75E-02
94	25850	IDP_T1_FAST_ROIs_L_parahipp_gyrus_post	IDP T1:unilateral regions	regional and tissue volume	mm3	Volume of grey matter in Left Parahippocampal Gyrus, posterior division	2.06E-01	2.06E-02

95	25851	IDP_T1_FAST_ROIs_R_parahipp_gyrus_post	IDP T1:unilateral regions	regional and tissue volume	mm3	Volume of grey matter in Right Parahippocampal Gyrus, posterior division	2.21E-01	1.95E-02
96	25852	IDP_T1_FAST_ROIs_L_lingual_gyrus	IDP T1:unilateral regions	regional and tissue volume	mm3	Volume of grey matter in Left Lingual Gyrus	1.61E-01	1.85E-02
97	25853	IDP_T1_FAST_ROIs_R_lingual_gyrus	IDP T1:unilateral regions	regional and tissue volume	mm3	Volume of grey matter in Right Lingual Gyrus	2.06E-01	2.14E-02
98	25854	IDP_T1_FAST_ROIs_L_temp_fusif_cortex_ant	IDP T1:unilateral regions	regional and tissue volume	mm3	Volume of grey matter in Left Temporal Fusiform Cortex, anterior division	1.37E-01	1.65E-02
99	25855	IDP_T1_FAST_ROIs_R_temp_fusif_cortex_ant	IDP T1:unilateral regions	regional and tissue volume	mm3	Volume of grey matter in Right Temporal Fusiform Cortex, anterior division	1.68E-01	1.69E-02
100	25856	IDP_T1_FAST_ROIs_L_temp_fusif_cortex_post	IDP T1:unilateral regions	regional and tissue volume	mm3	Volume of grey matter in Left Temporal Fusiform Cortex, posterior division	2.14E-01	2.08E-02
101	25857	IDP_T1_FAST_ROIs_R_temp_fusif_cortex_post	IDP T1:unilateral regions	regional and tissue volume	mm3	Volume of grey matter in Right Temporal Fusiform Cortex, posterior division	2.14E-01	1.94E-02
102	25858	IDP_T1_FAST_ROIs_L_temp_occ_fusif_cortex	IDP T1:unilateral regions	regional and tissue volume	mm3	Volume of grey matter in Left Temporal Occipital Fusiform Cortex	1.54E-01	1.82E-02
103	25859	IDP_T1_FAST_ROIs_R_temp_occ_fusif_cortex	IDP T1:unilateral regions	regional and tissue volume	mm3	Volume of grey matter in Right Temporal Occipital Fusiform Cortex	1.84E-01	1.83E-02
104	25860	IDP_T1_FAST_ROIs_L_occ_fusif_gyrus	IDP T1:unilateral regions	regional and tissue volume	mm3	Volume of grey matter in Left Occipital Fusiform Gyrus	1.35E-01	1.78E-02
105	25861	IDP_T1_FAST_ROIs_R_occ_fusif_gyrus	IDP T1:unilateral regions	regional and tissue volume	mm3	Volume of grey matter in Right Occipital Fusiform Gyrus	1.17E-01	1.69E-02

106	25862	IDP_T1_FAST_ROIs_L_front_operc_cortex	IDP T1:unilateral regions	regional and tissue volume	mm3	Volume of grey matter in Left Frontal Operculum Cortex	1.39E-01	1.67E-02
107	25863	IDP_T1_FAST_ROIs_R_front_operc_cortex	IDP T1:unilateral regions	regional and tissue volume	mm3	Volume of grey matter in Right Frontal Operculum Cortex	1.09E-01	1.57E-02
108	25864	IDP_T1_FAST_ROIs_L_cent_operc_cortex	IDP T1:unilateral regions	regional and tissue volume	mm3	Volume of grey matter in Left Central Opercular Cortex	1.48E-01	1.60E-02
109	25865	IDP_T1_FAST_ROIs_R_cent_operc_cortex	IDP T1:unilateral regions	regional and tissue volume	mm3	Volume of grey matter in Right Central Opercular Cortex	1.80E-01	1.92E-02
110	25866	IDP_T1_FAST_ROIs_L_parietal_operc_cortex	IDP T1:unilateral regions	regional and tissue volume	mm3	Volume of grey matter in Left Parietal Operculum Cortex	1.36E-01	1.56E-02
111	25867	IDP_T1_FAST_ROIs_R_parietal_operc_cortex	IDP T1:unilateral regions	regional and tissue volume	mm3	Volume of grey matter in Right Parietal Operculum Cortex	1.72E-01	1.84E-02
112	25868	IDP_T1_FAST_ROIs_L_planum_polare	IDP T1:unilateral regions	regional and tissue volume	mm3	Volume of grey matter in Left Planum Polare	1.76E-01	1.78E-02
113	25869	IDP_T1_FAST_ROIs_R_planum_polare	IDP T1:unilateral regions	regional and tissue volume	mm3	Volume of grey matter in Right Planum Polare	1.83E-01	1.96E-02
114	25870	IDP_T1_FAST_ROIs_L_heschl_gyrus	IDP T1:unilateral regions	regional and tissue volume	mm3	Volume of grey matter in Left Heschl's Gyrus (includes H1 and H2)	1.99E-01	1.83E-02
115	25871	IDP_T1_FAST_ROIs_R_heschl_gyrus	IDP T1:unilateral regions	regional and tissue volume	mm3	Volume of grey matter in Right Heschl's Gyrus (includes H1 and H2)	2.12E-01	1.83E-02
116	25872	IDP_T1_FAST_ROIs_L_planum_temporale	IDP T1:unilateral regions	regional and tissue volume	mm3	Volume of grey matter in Left Planum Temporale	1.76E-01	1.79E-02

117	25873	IDP_T1_FAST_ROIs_R_planum_temporale	IDP T1:unilateral regions	regional and tissue volume	mm3	Volume of grey matter in Right Planum Temporale	1.92E-01	1.80E-02
118	25874	IDP_T1_FAST_ROIs_L_supracalc_cortex	IDP T1:unilateral regions	regional and tissue volume	mm3	Volume of grey matter in Left Supracalcarine Cortex	1.83E-01	1.93E-02
119	25875	IDP_T1_FAST_ROIs_R_supracalc_cortex	IDP T1:unilateral regions	regional and tissue volume	mm3	Volume of grey matter in Right Supracalcarine Cortex	1.86E-01	1.89E-02
120	25876	IDP_T1_FAST_ROIs_L_occ_pole	IDP T1:unilateral regions	regional and tissue volume	mm3	Volume of grey matter in Left Occipital Pole	1.62E-01	1.87E-02
121	25877	IDP_T1_FAST_ROIs_R_occ_pole	IDP T1:unilateral regions	regional and tissue volume	mm3	Volume of grey matter in Right Occipital Pole	2.04E-01	1.95E-02
122	25878	IDP_T1_FAST_ROIs_L_thalamus	IDP T1:unilateral regions	regional and tissue volume	mm3	Volume of grey matter in Left Thalamus	2.70E-01	2.17E-02
123	25879	IDP_T1_FAST_ROIs_R_thalamus	IDP T1:unilateral regions	regional and tissue volume	mm3	Volume of grey matter in Right Thalamus	2.92E-01	2.02E-02
124	25880	IDP_T1_FAST_ROIs_L_caudate	IDP T1:unilateral regions	regional and tissue volume	mm3	Volume of grey matter in Left Caudate	2.70E-01	2.17E-02
125	25881	IDP_T1_FAST_ROIs_R_caudate	IDP T1:unilateral regions	regional and tissue volume	mm3	Volume of grey matter in Right Caudate	2.76E-01	2.25E-02
126	25882	IDP_T1_FAST_ROIs_L_putamen	IDP T1:unilateral regions	regional and tissue volume	mm3	Volume of grey matter in Left Putamen	2.88E-01	2.88E-02
127	25883	IDP_T1_FAST_ROIs_R_putamen	IDP T1:unilateral regions	regional and tissue volume	mm3	Volume of grey matter in Right Putamen	2.87E-01	2.96E-02

128	25884	IDP_T1_FAST_ROIs_L_pallidum	IDP T1:unilateral regions	regional and tissue volume	mm3	Volume of grey matter in Left Pallidum	1.67E-01	1.83E-02
129	25885	IDP_T1_FAST_ROIs_R_pallidum	IDP T1:unilateral regions	regional and tissue volume	mm3	Volume of grey matter in Right Pallidum	1.65E-01	1.81E-02
130	25886	IDP_T1_FAST_ROIs_L_hippocampus	IDP T1:unilateral regions	regional and tissue volume	mm3	Volume of grey matter in Left Hippocampus	2.65E-01	2.30E-02
131	25887	IDP_T1_FAST_ROIs_R_hippocampus	IDP T1:unilateral regions	regional and tissue volume	mm3	Volume of grey matter in Right Hippocampus	2.73E-01	2.48E-02
132	25888	IDP_T1_FAST_ROIs_L_amygdala	IDP T1:unilateral regions	regional and tissue volume	mm3	Volume of grey matter in Left Amygdala	1.87E-01	1.94E-02
133	25889	IDP_T1_FAST_ROIs_R_amygdala	IDP T1:unilateral regions	regional and tissue volume	mm3	Volume of grey matter in Right Amygdala	1.66E-01	1.53E-02
134	25890	IDP_T1_FAST_ROIs_L_ventral_striatum	IDP T1:unilateral regions	regional and tissue volume	mm3	Volume of grey matter in Left Ventral Striatum	2.80E-01	2.92E-02
135	25891	IDP_T1_FAST_ROIs_R_ventral_striatum	IDP T1:unilateral regions	regional and tissue volume	mm3	Volume of grey matter in Right Ventral Striatum	2.76E-01	2.89E-02
136	25892	IDP_T1_FAST_ROIs_brain_stem	IDP T1:unilateral regions	regional and tissue volume	mm3	Volume of grey matter in Brain-Stem	2.51E-01	2.30E-02
137	25893	IDP_T1_FAST_ROIs_L_cerebellum_I-IV	IDP T1:unilateral regions	regional and tissue volume	mm3	Volume of grey matter in Left I-IV Cerebellum	2.83E-01	2.11E-02
138	25894	IDP_T1_FAST_ROIs_R_cerebellum_I-IV	IDP T1:unilateral regions	regional and tissue volume	mm3	Volume of grey matter in Right I-IV Cerebellum	2.84E-01	1.99E-02

139	25895	IDP_T1_FAST_ROIs_L_cerebellum_V	IDP T1:unilateral regions	regional and tissue volume	mm3	Volume of grey matter in Left V Cerebellum	2.80E-01	2.10E-02
140	25896	IDP_T1_FAST_ROIs_R_cerebellum_V	IDP T1:unilateral regions	regional and tissue volume	mm3	Volume of grey matter in Right V Cerebellum	2.74E-01	2.03E-02
141	25897	IDP_T1_FAST_ROIs_L_cerebellum_VI	IDP T1:unilateral regions	regional and tissue volume	mm3	Volume of grey matter in Left VI Cerebellum	2.74E-01	2.39E-02
142	25898	IDP_T1_FAST_ROIs_V_cerebellum_VI	IDP T1:unilateral regions	regional and tissue volume	mm3	Volume of grey matter in Vermis VI Cerebellum	2.58E-01	2.24E-02
143	25899	IDP_T1_FAST_ROIs_R_cerebellum_VI	IDP T1:unilateral regions	regional and tissue volume	mm3	Volume of grey matter in Right VI Cerebellum	2.69E-01	2.26E-02
144	25900	IDP_T1_FAST_ROIs_L_cerebellum_crus_I	IDP T1:unilateral regions	regional and tissue volume	mm3	Volume of grey matter in Left Crus I Cerebellum	3.18E-01	2.35E-02
145	25901	IDP_T1_FAST_ROIs_V_cerebellum_crus_I	IDP T1:unilateral regions	regional and tissue volume	mm3	Volume of grey matter in Vermis Crus I Cerebellum	7.89E-02	1.45E-02
146	25902	IDP_T1_FAST_ROIs_R_cerebellum_crus_I	IDP T1:unilateral regions	regional and tissue volume	mm3	Volume of grey matter in Right Crus I Cerebellum	3.18E-01	2.45E-02
147	25903	IDP_T1_FAST_ROIs_L_cerebellum_crus_II	IDP T1:unilateral regions	regional and tissue volume	mm3	Volume of grey matter in Left Crus II Cerebellum	3.02E-01	2.66E-02
148	25904	IDP_T1_FAST_ROIs_V_cerebellum_crus_II	IDP T1:unilateral regions	regional and tissue volume	mm3	Volume of grey matter in Vermis Crus II Cerebellum	2.32E-01	2.22E-02
149	25905	IDP_T1_FAST_ROIs_R_cerebellum_crus_II	IDP T1:unilateral regions	regional and tissue volume	mm3	Volume of grey matter in Right Crus II Cerebellum	3.29E-01	3.18E-02

150	25906	IDP_T1_FAST_ROIs_L_cerebellum_VIIb	IDP T1:unilateral regions	regional and tissue volume	mm3	Volume of grey matter in Left VIIb Cerebellum	2.87E-01	2.63E-02
151	25907	IDP_T1_FAST_ROIs_V_cerebellum_VIIb	IDP T1:unilateral regions	regional and tissue volume	mm3	Volume of grey matter in Vermis VIIb Cerebellum	2.53E-01	2.11E-02
152	25908	IDP_T1_FAST_ROIs_R_cerebellum_VIIb	IDP T1:unilateral regions	regional and tissue volume	mm3	Volume of grey matter in Right VIIb Cerebellum	3.14E-01	3.02E-02
153	25909	IDP_T1_FAST_ROIs_L_cerebellum_VIIIa	IDP T1:unilateral regions	regional and tissue volume	mm3	Volume of grey matter in Left VIIIa Cerebellum	2.79E-01	2.41E-02
154	25910	IDP_T1_FAST_ROIs_V_cerebellum_VIIIa	IDP T1:unilateral regions	regional and tissue volume	mm3	Volume of grey matter in Vermis VIIIa Cerebellum	3.16E-01	2.50E-02
155	25911	IDP_T1_FAST_ROIs_R_cerebellum_VIIIa	IDP T1:unilateral regions	regional and tissue volume	mm3	Volume of grey matter in Right VIIIa Cerebellum	2.74E-01	2.61E-02
156	25912	IDP_T1_FAST_ROIs_L_cerebellum_VIIIb	IDP T1:unilateral regions	regional and tissue volume	mm3	Volume of grey matter in Left VIIIb Cerebellum	2.84E-01	2.39E-02
157	25913	IDP_T1_FAST_ROIs_V_cerebellum_VIIIb	IDP T1:unilateral regions	regional and tissue volume	mm3	Volume of grey matter in Vermis VIIIb Cerebellum	3.33E-01	2.70E-02
158	25914	IDP_T1_FAST_ROIs_R_cerebellum_VIIIb	IDP T1:unilateral regions	regional and tissue volume	mm3	Volume of grey matter in Right VIIIb Cerebellum	2.55E-01	2.13E-02
159	25915	IDP_T1_FAST_ROIs_L_cerebellum_IX	IDP T1:unilateral regions	regional and tissue volume	mm3	Volume of grey matter in Left IX Cerebellum	3.38E-01	2.63E-02
160	25916	IDP_T1_FAST_ROIs_V_cerebellum_IX	IDP T1:unilateral regions	regional and tissue volume	mm3	Volume of grey matter in Vermis IX Cerebellum	3.10E-01	2.81E-02

161	25917	IDP_T1_FAST_ROIs_R_cerebellum_IX	IDP T1:unilateral regions	regional and tissue volume	mm3	Volume of grey matter in Right IX Cerebellum	3.35E-01	2.67E-02
162	25918	IDP_T1_FAST_ROIs_L_cerebellum_X	IDP T1:unilateral regions	regional and tissue volume	mm3	Volume of grey matter in Left X Cerebellum	2.55E-01	2.14E-02
163	25919	IDP_T1_FAST_ROIs_V_cerebellum_X	IDP T1:unilateral regions	regional and tissue volume	mm3	Volume of grey matter in Vermis X Cerebellum	2.53E-01	2.25E-02
164	25920	IDP_T1_FAST_ROIs_R_cerebellum_X	IDP T1:unilateral regions	regional and tissue volume	mm3	Volume of grey matter in Right X Cerebellum	2.43E-01	2.02E-02
165	26514	aseg_global_volume_BrainSeg	aseg:global	regional and tissue volume	mm3	Volume of BrainSeg in the whole brain generated by subcortical volumetric segmentation (aseg)	2.53E-01	2.46E-02
166	26515	aseg_global_volume_BrainSegNotVent	aseg:global	regional and tissue volume	mm3	Volume of BrainSegNotVent in the whole brain generated by subcortical volumetric segmentation (aseg)	2.49E-01	2.42E-02
167	26516	aseg_global_volume_BrainSegNotVentSurf	aseg:global	regional and tissue volume	mm3	Volume of BrainSegNotVentSurf in the whole brain generated by subcortical volumetric segmentation (aseg)	2.59E-01	2.49E-02
168	26517	aseg_global_volume_SubCortGray	aseg:global	regional and tissue volume	mm3	Volume of SubCortGray in the whole brain generated by subcortical volumetric segmentation (aseg)	3.31E-01	2.51E-02
169	26518	aseg_global_volume_TotalGray	aseg:global	regional and tissue volume	mm3	Volume of TotalGray in the whole brain generated by subcortical volumetric segmentation (aseg)	2.15E-01	2.04E-02
170	26519	aseg_global_volume_SupraTentorial	aseg:global	regional and tissue volume	mm3	Volume of SupraTentorial in the whole brain generated by subcortical volumetric segmentation (aseg)	2.76E-01	2.48E-02
171	26520	aseg_global_volume_SupraTentorialNotVent	aseg:global	regional and tissue volume	mm3	Volume of SupraTentorialNotVent in the whole brain generated by subcortical volumetric segmentation (aseg)	2.74E-01	2.42E-02
172	26521	aseg_global_volume_EstimatedTotalIntraCranial	aseg:global	regional and tissue volume	mm3	Volume of EstimatedTotalIntraCranial in the whole brain generated by subcortical volumetric segmentation (aseg)	1.75E-01	2.23E-02

173	26522	aseg_global_volume_VentricleChoroid	aseg:global	regional and tissue volume	mm3	Volume of VentricleChoroid in the whole brain generated by subcortical volumetric segmentation (aseg)	2.81E-01	2.52E-02
174	26523	aseg_global_volume_3rd-Ventricle	aseg:global	regional and tissue volume	mm3	Volume of 3rd-Ventricle in the whole brain generated by subcortical volumetric segmentation (aseg)	2.81E-01	2.45E-02
175	26524	aseg_global_volume_4th-Ventricle	aseg:global	regional and tissue volume	mm3	Volume of 4th-Ventricle in the whole brain generated by subcortical volumetric segmentation (aseg)	3.40E-01	2.75E-02
176	26525	aseg_global_volume_5th-Ventricle	aseg:global	regional and tissue volume	mm3	Volume of 5th-Ventricle in the whole brain generated by subcortical volumetric segmentation (aseg)	-5.00E-03	1.38E-02
177	26526	aseg_global_volume_Brain-Stem	aseg:global	regional and tissue volume	mm3	Volume of Brain-Stem in the whole brain generated by subcortical volumetric segmentation (aseg)	3.97E-01	3.04E-02
178	26527	aseg_global_volume_CSF	aseg:global	regional and tissue volume	mm3	Volume of CSF in the whole brain generated by subcortical volumetric segmentation (aseg)	2.72E-01	2.27E-02
179	26528	aseg_global_volume_WM-hypointensities	aseg:global	regional and tissue volume	mm3	Volume of WM-hypointensities in the whole brain generated by subcortical volumetric segmentation (aseg)	2.30E-01	2.28E-02
180	26529	aseg_global_volume_non-WM-hypointensities	aseg:global	regional and tissue volume	mm3	Volume of non-WM-hypointensities in the whole brain generated by subcortical volumetric segmentation (aseg)	3.80E-03	1.22E-02
181	26530	aseg_global_volume_Optic-Chiasm	aseg:global	regional and tissue volume	mm3	Volume of Optic-Chiasm in the whole brain generated by subcortical volumetric segmentation (aseg)	1.67E-01	2.66E-02
182	26531	aseg_global_volume_CC-Posterior	aseg:global	regional and tissue volume	mm3	Volume of CC-Posterior in the whole brain generated by subcortical volumetric segmentation (aseg)	3.35E-01	2.43E-02
183	26532	aseg_global_volume_CC-Mid-Posterior	aseg:global	regional and tissue volume	mm3	Volume of CC-Mid-Posterior in the whole brain generated by subcortical volumetric segmentation (aseg)	1.61E-01	1.69E-02
184	26533	aseg_global_volume_CC-Central	aseg:global	regional and tissue volume	mm3	Volume of CC-Central in the whole brain generated by subcortical volumetric segmentation (aseg)	2.18E-01	1.68E-02
185	26534	aseg_global_volume_CC-Mid-Anterior	aseg:global	regional and tissue volume	mm3	Volume of CC-Mid-Anterior in the whole brain generated by subcortical volumetric segmentation (aseg)	2.21E-01	1.95E-02

186	26535	aseg_global_volume_CC-Anterior	aseg:global	regional and tissue volume	mm3	Volume of CC-Anterior in the whole brain generated by subcortical volumetric segmentation (aseg)	2.55E-01	2.24E-02
187	26536	aseg_global_volume-ratio_BrainSegVol-to-eTIV	aseg:global	regional and tissue volume	ratio	Volume-ratio of BrainSegVol-to-eTIV in the whole brain generated by subcortical volumetric segmentation (aseg)	2.34E-01	2.15E-02
188	26537	aseg_global_volume-ratio_MaskVol-to-eTIV	aseg:global	regional and tissue volume	ratio	Volume-ratio of MaskVol-to-eTIV in the whole brain generated by subcortical volumetric segmentation (aseg)	1.03E-01	1.79E-02
189	26552	aseg_lh_volume_Cortex	aseg:unilateral regions	regional and tissue volume	mm3	Volume of Cortex in the left hemisphere generated by subcortical volumetric segmentation (aseg)	2.07E-01	1.94E-02
190	26553	aseg_lh_volume_CerebralWhiteMatter	aseg:unilateral regions	regional and tissue volume	mm3	Volume of CerebralWhiteMatter in the left hemisphere generated by subcortical volumetric segmentation (aseg)	3.35E-01	2.73E-02
191	26554	aseg_lh_volume_Lateral-Ventricle	aseg:unilateral regions	regional and tissue volume	mm3	Volume of Lateral-Ventricle in the left hemisphere generated by subcortical volumetric segmentation (aseg)	2.74E-01	2.44E-02
192	26555	aseg_lh_volume_Inf-Lat-Vent	aseg:unilateral regions	regional and tissue volume	mm3	Volume of Inf-Lat-Vent in the left hemisphere generated by subcortical volumetric segmentation (aseg)	1.40E-01	1.82E-02
193	26556	aseg_lh_volume_Cerebellum-White-Matter	aseg:unilateral regions	regional and tissue volume	mm3	Volume of Cerebellum-White-Matter in the left hemisphere generated by subcortical volumetric segmentation (aseg)	3.22E-01	2.79E-02
194	26557	aseg_lh_volume_Cerebellum-Cortex	aseg:unilateral regions	regional and tissue volume	mm3	Volume of Cerebellum-Cortex in the left hemisphere generated by subcortical volumetric segmentation (aseg)	3.54E-01	2.89E-02
195	26558	aseg_lh_volume_Thalamus-Proper	aseg:unilateral regions	regional and tissue volume	mm3	Volume of Thalamus-Proper in the left hemisphere generated by subcortical volumetric segmentation (aseg)	2.78E-01	2.37E-02
196	26559	aseg_lh_volume_Caudate	aseg:unilateral regions	regional and tissue volume	mm3	Volume of Caudate in the left hemisphere generated by subcortical volumetric segmentation (aseg)	3.49E-01	2.66E-02
197	26560	aseg_lh_volume_Putamen	aseg:unilateral regions	regional and tissue volume	mm3	Volume of Putamen in the left hemisphere generated by subcortical volumetric segmentation (aseg)	3.21E-01	2.74E-02
198	26561	aseg_lh_volume_Pallidum	aseg:unilateral regions	regional and tissue volume	mm3	Volume of Pallidum in the left hemisphere generated by subcortical volumetric segmentation (aseg)	3.03E-01	2.22E-02

199	26562	aseg_lh_volume_Hippocampus	aseg:unilateral regions	regional and tissue volume	mm3	Volume of Hippocampus in the left hemisphere generated by subcortical volumetric segmentation (aseg)	2.64E-01	2.31E-02
200	26563	aseg_lh_volume_Amygdala	aseg:unilateral regions	regional and tissue volume	mm3	Volume of Amygdala in the left hemisphere generated by subcortical volumetric segmentation (aseg)	2.24E-01	1.88E-02
201	26564	aseg_lh_volume_Accumbens-area	aseg:unilateral regions	regional and tissue volume	mm3	Volume of Accumbens-area in the left hemisphere generated by subcortical volumetric segmentation (aseg)	2.37E-01	2.18E-02
202	26565	aseg_lh_volume_VentralDC	aseg:unilateral regions	regional and tissue volume	mm3	Volume of VentralDC in the left hemisphere generated by subcortical volumetric segmentation (aseg)	3.12E-01	2.82E-02
203	26566	aseg_lh_volume_vessel	aseg:unilateral regions	regional and tissue volume	mm3	Volume of vessel in the left hemisphere generated by subcortical volumetric segmentation (aseg)	1.06E-01	1.61E-02
204	26567	aseg_lh_volume_choroid-plexus	aseg:unilateral regions	regional and tissue volume	mm3	Volume of choroid-plexus in the left hemisphere generated by subcortical volumetric segmentation (aseg)	2.39E-01	2.12E-02
205	26568	aseg_lh_number_HolesBeforeFixing	aseg:unilateral regions	regional and tissue volume	count	Number of HolesBeforeFixing in the left hemisphere generated by subcortical volumetric segmentation (aseg)	1.55E-01	1.92E-02
206	26583	aseg_rh_volume_Cortex	aseg:unilateral regions	regional and tissue volume	mm3	Volume of Cortex in the right hemisphere generated by subcortical volumetric segmentation (aseg)	1.92E-01	1.85E-02
207	26584	aseg_rh_volume_CerebralWhiteMatter	aseg:unilateral regions	regional and tissue volume	mm3	Volume of CerebralWhiteMatter in the right hemisphere generated by subcortical volumetric segmentation (aseg)	3.42E-01	2.82E-02
208	26585	aseg_rh_volume_Lateral-Ventricle	aseg:unilateral regions	regional and tissue volume	mm3	Volume of Lateral-Ventricle in the right hemisphere generated by subcortical volumetric segmentation (aseg)	2.63E-01	2.48E-02
209	26586	aseg_rh_volume_Inf-Lat-Vent	aseg:unilateral regions	regional and tissue volume	mm3	Volume of Inf-Lat-Vent in the right hemisphere generated by subcortical volumetric segmentation (aseg)	1.28E-01	1.60E-02
210	26587	aseg_rh_volume_Cerebellum-White-Matter	aseg:unilateral regions	regional and tissue volume	mm3	Volume of Cerebellum-White-Matter in the right hemisphere generated by subcortical volumetric segmentation (aseg)	2.95E-01	2.62E-02
211	26588	aseg_rh_volume_Cerebellum-Cortex	aseg:unilateral regions	regional and tissue volume	mm3	Volume of Cerebellum-Cortex in the right hemisphere generated by subcortical volumetric segmentation (aseg)	3.50E-01	3.01E-02

212	26589	aseg_rh_volume_Thalamus-Proper	aseg:unilateral regions	regional and tissue volume	mm3	Volume of Thalamus-Proper in the right hemisphere generated by subcortical volumetric segmentation (aseg)	3.06E-01	2.40E-02
213	26590	aseg_rh_volume_Caudate	aseg:unilateral regions	regional and tissue volume	mm3	Volume of Caudate in the right hemisphere generated by subcortical volumetric segmentation (aseg)	3.35E-01	2.61E-02
214	26591	aseg_rh_volume_Putamen	aseg:unilateral regions	regional and tissue volume	mm3	Volume of Putamen in the right hemisphere generated by subcortical volumetric segmentation (aseg)	3.38E-01	2.71E-02
215	26592	aseg_rh_volume_Pallidum	aseg:unilateral regions	regional and tissue volume	mm3	Volume of Pallidum in the right hemisphere generated by subcortical volumetric segmentation (aseg)	3.17E-01	2.20E-02
216	26593	aseg_rh_volume_Hippocampus	aseg:unilateral regions	regional and tissue volume	mm3	Volume of Hippocampus in the right hemisphere generated by subcortical volumetric segmentation (aseg)	2.88E-01	2.44E-02
217	26594	aseg_rh_volume_Amygdala	aseg:unilateral regions	regional and tissue volume	mm3	Volume of Amygdala in the right hemisphere generated by subcortical volumetric segmentation (aseg)	2.36E-01	2.17E-02
218	26595	aseg_rh_volume_Accumbens-area	aseg:unilateral regions	regional and tissue volume	mm3	Volume of Accumbens-area in the right hemisphere generated by subcortical volumetric segmentation (aseg)	2.56E-01	2.37E-02
219	26596	aseg_rh_volume_VentralDC	aseg:unilateral regions	regional and tissue volume	mm3	Volume of VentralDC in the right hemisphere generated by subcortical volumetric segmentation (aseg)	3.31E-01	2.68E-02
220	26597	aseg_rh_volume_vessel	aseg:unilateral regions	regional and tissue volume	mm3	Volume of vessel in the right hemisphere generated by subcortical volumetric segmentation (aseg)	1.04E-01	1.87E-02
221	26598	aseg_rh_volume_choroid-plexus	aseg:unilateral regions	regional and tissue volume	mm3	Volume of choroid-plexus in the right hemisphere generated by subcortical volumetric segmentation (aseg)	2.17E-01	2.11E-02
222	26599	aseg_rh_number_HolesBeforeFixing	aseg:unilateral regions	regional and tissue volume	count	Number of HolesBeforeFixing in the right hemisphere generated by subcortical volumetric segmentation (aseg)	1.32E-01	2.07E-02
223	26600	AmygNuclei_lh_volume_Lateral-nucleus	Amygdala Nuclei	regional and tissue volume	mm3	Volume of Lateral-nucleus in the left hemisphere generated by subcortical volumetric sub-segmentation of the Amygdala Nuclei	2.35E-01	2.02E-02
224	26601	AmygNuclei_lh_volume_Basal-nucleus	Amygdala Nuclei	regional and tissue volume	mm3	Volume of Basal-nucleus in the left hemisphere generated by subcortical volumetric sub-segmentation of the Amygdala Nuclei	1.96E-01	1.73E-02

225	26602	AmygNuclei_lh_volume_Accessory-Basal-nucleus	Amygdala Nuclei	regional and tissue volume	mm3	Volume of Accessory-Basal-nucleus in the left hemisphere generated by subcortical volumetric sub-segmentation of the Amygdala Nuclei	1.72E-01	1.71E-02
226	26603	AmygNuclei_lh_volume_Anterior-amygdaloid-area-AAA	Amygdala Nuclei	regional and tissue volume	mm3	Volume of Anterior-amygdaloid-area-AAA in the left hemisphere generated by subcortical volumetric sub-segmentation of the Amygdala Nuclei	1.86E-01	1.88E-02
227	26604	AmygNuclei_lh_volume_Central-nucleus	Amygdala Nuclei	regional and tissue volume	mm3	Volume of Central-nucleus in the left hemisphere generated by subcortical volumetric sub-segmentation of the Amygdala Nuclei	1.38E-01	1.85E-02
228	26605	AmygNuclei_lh_volume_Medial-nucleus	Amygdala Nuclei	regional and tissue volume	mm3	Volume of Medial-nucleus in the left hemisphere generated by subcortical volumetric sub-segmentation of the Amygdala Nuclei	1.37E-01	1.68E-02
229	26606	AmygNuclei_lh_volume_Cortical-nucleus	Amygdala Nuclei	regional and tissue volume	mm3	Volume of Cortical-nucleus in the left hemisphere generated by subcortical volumetric sub-segmentation of the Amygdala Nuclei	1.43E-01	1.80E-02
230	26607	AmygNuclei_lh_volume_Corticoamygdaloid-transitio	Amygdala Nuclei	regional and tissue volume	mm3	Volume of Corticoamygdaloid-transitio in the left hemisphere generated by subcortical volumetric sub-segmentation of the Amygdala Nuclei	1.51E-01	1.75E-02
231	26608	AmygNuclei_lh_volume_Paralaminar-nucleus	Amygdala Nuclei	regional and tissue volume	mm3	Volume of Paralaminar-nucleus in the left hemisphere generated by subcortical volumetric sub-segmentation of the Amygdala Nuclei	1.77E-01	1.81E-02
232	26609	AmygNuclei_lh_volume_Whole-amygdala	Amygdala Nuclei	regional and tissue volume	mm3	Volume of Whole-amygdala in the left hemisphere generated by subcortical volumetric sub-segmentation of the Amygdala Nuclei	2.21E-01	1.81E-02
233	26610	AmygNuclei_rh_volume_Lateral-nucleus	Amygdala Nuclei	regional and tissue volume	mm3	Volume of Lateral-nucleus in the right hemisphere generated by subcortical volumetric sub-segmentation of the Amygdala Nuclei	2.63E-01	2.29E-02
234	26611	AmygNuclei_rh_volume_Basal-nucleus	Amygdala Nuclei	regional and tissue volume	mm3	Volume of Basal-nucleus in the right hemisphere generated by subcortical volumetric sub-segmentation of the Amygdala Nuclei	2.31E-01	2.00E-02
235	26612	AmygNuclei_rh_volume_Accessory-Basal-nucleus	Amygdala Nuclei	regional and tissue volume	mm3	Volume of Accessory-Basal-nucleus in the right hemisphere generated by subcortical volumetric sub-segmentation of the Amygdala Nuclei	1.97E-01	1.95E-02

236	26613	AmygNuclei_rh_volume_Anterior-amygdaloid-area-AAA	Amygdala Nuclei	regional and tissue volume	mm3	Volume of Anterior-amygdaloid-area-AAA in the right hemisphere generated by subcortical volumetric sub-segmentation of the Amygdala Nuclei	1.97E-01	1.89E-02
237	26614	AmygNuclei_rh_volume_Central-nucleus	Amygdala Nuclei	regional and tissue volume	mm3	Volume of Central-nucleus in the right hemisphere generated by subcortical volumetric sub-segmentation of the Amygdala Nuclei	1.56E-01	2.05E-02
238	26615	AmygNuclei_rh_volume_Medial-nucleus	Amygdala Nuclei	regional and tissue volume	mm3	Volume of Medial-nucleus in the right hemisphere generated by subcortical volumetric sub-segmentation of the Amygdala Nuclei	1.46E-01	2.00E-02
239	26616	AmygNuclei_rh_volume_Cortical-nucleus	Amygdala Nuclei	regional and tissue volume	mm3	Volume of Cortical-nucleus in the right hemisphere generated by subcortical volumetric sub-segmentation of the Amygdala Nuclei	1.71E-01	2.07E-02
240	26617	AmygNuclei_rh_volume_Corticoamygdaloid-transitio	Amygdala Nuclei	regional and tissue volume	mm3	Volume of Corticoamygdaloid-transitio in the right hemisphere generated by subcortical volumetric sub-segmentation of the Amygdala Nuclei	1.66E-01	2.06E-02
241	26618	AmygNuclei_rh_volume_Paralaminar-nucleus	Amygdala Nuclei	regional and tissue volume	mm3	Volume of Paralaminar-nucleus in the right hemisphere generated by subcortical volumetric sub-segmentation of the Amygdala Nuclei	1.90E-01	2.11E-02
242	26619	AmygNuclei_rh_volume_Whole-amygdala	Amygdala Nuclei	regional and tissue volume	mm3	Volume of Whole-amygdala in the right hemisphere generated by subcortical volumetric sub-segmentation of the Amygdala Nuclei	2.43E-01	2.03E-02
243	26620	HippSubfield_lh_volume_Hippocampal-tail	Hippocampus Subfield	regional and tissue volume	mm3	Volume of Hippocampal-tail in the left hemisphere generated by subcortical volumetric sub-segmentation of the Hippocampal Subfields	2.18E-01	2.52E-02
244	26621	HippSubfield_lh_volume_subiculum-body	Hippocampus Subfield	regional and tissue volume	mm3	Volume of subiculum-body in the left hemisphere generated by subcortical volumetric sub-segmentation of the Hippocampal Subfields	2.01E-01	3.13E-02
245	26622	HippSubfield_lh_volume_CA1-body	Hippocampus Subfield	regional and tissue volume	mm3	Volume of CA1-body in the left hemisphere generated by subcortical volumetric sub-segmentation of the Hippocampal Subfields	2.35E-01	2.15E-02
246	26623	HippSubfield_lh_volume_subiculum-head	Hippocampus Subfield	regional and tissue volume	mm3	Volume of subiculum-head in the left hemisphere generated by subcortical volumetric sub-segmentation of the Hippocampal Subfields	2.20E-01	1.88E-02

247	26624	HippSubfield_lh_volume_hippocampal-fissure	Hippocampus Subfield	regional and tissue volume	mm3	Volume of hippocampal-fissure in the left hemisphere generated by subcortical volumetric sub-segmentation of the Hippocampal Subfields	1.44E-01	2.09E-02
248	26625	HippSubfield_lh_volume_presubiculum-head	Hippocampus Subfield	regional and tissue volume	mm3	Volume of presubiculum-head in the left hemisphere generated by subcortical volumetric sub-segmentation of the Hippocampal Subfields	1.39E-01	1.79E-02
249	26626	HippSubfield_lh_volume_CA1-head	Hippocampus Subfield	regional and tissue volume	mm3	Volume of CA1-head in the left hemisphere generated by subcortical volumetric sub-segmentation of the Hippocampal Subfields	2.37E-01	2.17E-02
250	26627	HippSubfield_lh_volume_presubiculum-body	Hippocampus Subfield	regional and tissue volume	mm3	Volume of presubiculum-body in the left hemisphere generated by subcortical volumetric sub-segmentation of the Hippocampal Subfields	2.23E-01	2.23E-02
251	26628	HippSubfield_lh_volume_parasubiculum	Hippocampus Subfield	regional and tissue volume	mm3	Volume of parasubiculum in the left hemisphere generated by subcortical volumetric sub-segmentation of the Hippocampal Subfields	1.30E-01	1.68E-02
252	26629	HippSubfield_lh_volume_molecular-layer-HP-head	Hippocampus Subfield	regional and tissue volume	mm3	Volume of molecular-layer-HP-head in the left hemisphere generated by subcortical volumetric sub-segmentation of the Hippocampal Subfields	1.91E-01	2.02E-02
253	26630	HippSubfield_lh_volume_molecular-layer-HP-body	Hippocampus Subfield	regional and tissue volume	mm3	Volume of molecular-layer-HP-body in the left hemisphere generated by subcortical volumetric sub-segmentation of the Hippocampal Subfields	1.50E-01	1.73E-02
254	26631	HippSubfield_lh_volume_GC-ML-DG-head	Hippocampus Subfield	regional and tissue volume	mm3	Volume of GC-ML-DG-head in the left hemisphere generated by subcortical volumetric sub-segmentation of the Hippocampal Subfields	2.19E-01	2.16E-02
255	26632	HippSubfield_lh_volume_CA3-body	Hippocampus Subfield	regional and tissue volume	mm3	Volume of CA3-body in the left hemisphere generated by subcortical volumetric sub-segmentation of the Hippocampal Subfields	2.17E-01	2.27E-02
256	26633	HippSubfield_lh_volume_GC-ML-DG-body	Hippocampus Subfield	regional and tissue volume	mm3	Volume of GC-ML-DG-body in the left hemisphere generated by subcortical volumetric sub-segmentation of the Hippocampal Subfields	2.13E-01	2.48E-02
257	26634	HippSubfield_lh_volume_CA4-head	Hippocampus Subfield	regional and tissue volume	mm3	Volume of CA4-head in the left hemisphere generated by subcortical volumetric sub-segmentation of the Hippocampal Subfields	2.12E-01	2.15E-02

258	26635	HippSubfield_lh_volume_CA4-body	Hippocampus Subfield	regional and tissue volume	mm3	Volume of CA4-body in the left hemisphere generated by subcortical volumetric sub-segmentation of the Hippocampal Subfields	2.27E-01	2.41E-02
259	26636	HippSubfield_lh_volume_fimbria	Hippocampus Subfield	regional and tissue volume	mm3	Volume of fimbria in the left hemisphere generated by subcortical volumetric sub-segmentation of the Hippocampal Subfields	1.53E-01	1.87E-02
260	26637	HippSubfield_lh_volume_CA3-head	Hippocampus Subfield	regional and tissue volume	mm3	Volume of CA3-head in the left hemisphere generated by subcortical volumetric sub-segmentation of the Hippocampal Subfields	2.38E-01	2.20E-02
261	26638	HippSubfield_lh_volume_HATA	Hippocampus Subfield	regional and tissue volume	mm3	Volume of HATA in the left hemisphere generated by subcortical volumetric sub-segmentation of the Hippocampal Subfields	1.31E-01	1.86E-02
262	26639	HippSubfield_lh_volume_Whole-hippocampal-body	Hippocampus Subfield	regional and tissue volume	mm3	Volume of Whole-hippocampal-body in the left hemisphere generated by subcortical volumetric sub-segmentation of the Hippocampal Subfields	2.43E-01	2.72E-02
263	26640	HippSubfield_lh_volume_Whole-hippocampal-head	Hippocampus Subfield	regional and tissue volume	mm3	Volume of Whole-hippocampal-head in the left hemisphere generated by subcortical volumetric sub-segmentation of the Hippocampal Subfields	2.57E-01	2.33E-02
264	26641	HippSubfield_lh_volume_Whole-hippocampus	Hippocampus Subfield	regional and tissue volume	mm3	Volume of Whole-hippocampus in the left hemisphere generated by subcortical volumetric sub-segmentation of the Hippocampal Subfields	2.75E-01	2.51E-02
265	26642	HippSubfield_rh_volume_Hippocampal-tail	Hippocampus Subfield	regional and tissue volume	mm3	Volume of Hippocampal-tail in the right hemisphere generated by subcortical volumetric sub-segmentation of the Hippocampal Subfields	2.47E-01	2.46E-02
266	26643	HippSubfield_rh_volume_subiculum-body	Hippocampus Subfield	regional and tissue volume	mm3	Volume of subiculum-body in the right hemisphere generated by subcortical volumetric sub-segmentation of the Hippocampal Subfields	2.38E-01	2.43E-02
267	26644	HippSubfield_rh_volume_CA1-body	Hippocampus Subfield	regional and tissue volume	mm3	Volume of CA1-body in the right hemisphere generated by subcortical volumetric sub-segmentation of the Hippocampal Subfields	2.10E-01	1.92E-02
268	26645	HippSubfield_rh_volume_subiculum-head	Hippocampus Subfield	regional and tissue volume	mm3	Volume of subiculum-head in the right hemisphere generated by subcortical volumetric sub-segmentation of the Hippocampal Subfields	2.48E-01	2.21E-02

269	26646	HippSubfield_rh_volume_hippocampal-fissure	Hippocampus Subfield	regional and tissue volume	mm3	Volume of hippocampal-fissure in the right hemisphere generated by subcortical volumetric sub-segmentation of the Hippocampal Subfields	1.78E-01	1.92E-02
270	26647	HippSubfield_rh_volume_presubiculum-head	Hippocampus Subfield	regional and tissue volume	mm3	Volume of presubiculum-head in the right hemisphere generated by subcortical volumetric sub-segmentation of the Hippocampal Subfields	1.88E-01	1.88E-02
271	26648	HippSubfield_rh_volume_CA1-head	Hippocampus Subfield	regional and tissue volume	mm3	Volume of CA1-head in the right hemisphere generated by subcortical volumetric sub-segmentation of the Hippocampal Subfields	2.56E-01	2.30E-02
272	26649	HippSubfield_rh_volume_presubiculum-body	Hippocampus Subfield	regional and tissue volume	mm3	Volume of presubiculum-body in the right hemisphere generated by subcortical volumetric sub-segmentation of the Hippocampal Subfields	2.21E-01	1.84E-02
273	26650	HippSubfield_rh_volume_parasubiculum	Hippocampus Subfield	regional and tissue volume	mm3	Volume of parasubiculum in the right hemisphere generated by subcortical volumetric sub-segmentation of the Hippocampal Subfields	1.26E-01	1.58E-02
274	26651	HippSubfield_rh_volume_molecular-layer-HP-head	Hippocampus Subfield	regional and tissue volume	mm3	Volume of molecular-layer-HP-head in the right hemisphere generated by subcortical volumetric sub-segmentation of the Hippocampal Subfields	2.01E-01	1.97E-02
275	26652	HippSubfield_rh_volume_molecular-layer-HP-body	Hippocampus Subfield	regional and tissue volume	mm3	Volume of molecular-layer-HP-body in the right hemisphere generated by subcortical volumetric sub-segmentation of the Hippocampal Subfields	1.78E-01	2.17E-02
276	26653	HippSubfield_rh_volume_GC-ML-DG-head	Hippocampus Subfield	regional and tissue volume	mm3	Volume of GC-ML-DG-head in the right hemisphere generated by subcortical volumetric sub-segmentation of the Hippocampal Subfields	2.42E-01	2.18E-02
277	26654	HippSubfield_rh_volume_CA3-body	Hippocampus Subfield	regional and tissue volume	mm3	Volume of CA3-body in the right hemisphere generated by subcortical volumetric sub-segmentation of the Hippocampal Subfields	2.11E-01	2.12E-02
278	26655	HippSubfield_rh_volume_GC-ML-DG-body	Hippocampus Subfield	regional and tissue volume	mm3	Volume of GC-ML-DG-body in the right hemisphere generated by subcortical volumetric sub-segmentation of the Hippocampal Subfields	2.15E-01	2.34E-02
279	26656	HippSubfield_rh_volume_CA4-head	Hippocampus Subfield	regional and tissue volume	mm3	Volume of CA4-head in the right hemisphere generated by subcortical volumetric sub-segmentation of the Hippocampal Subfields	2.23E-01	2.15E-02

280	26657	HippSubfield_rh_volume_CA4-body	Hippocampus Subfield	regional and tissue volume	mm3	Volume of CA4-body in the right hemisphere generated by subcortical volumetric sub-segmentation of the Hippocampal Subfields	2.27E-01	2.63E-02
281	26658	HippSubfield_rh_volume_fimbria	Hippocampus Subfield	regional and tissue volume	mm3	Volume of fimbria in the right hemisphere generated by subcortical volumetric sub-segmentation of the Hippocampal Subfields	1.46E-01	1.85E-02
282	26659	HippSubfield_rh_volume_CA3-head	Hippocampus Subfield	regional and tissue volume	mm3	Volume of CA3-head in the right hemisphere generated by subcortical volumetric sub-segmentation of the Hippocampal Subfields	2.31E-01	2.18E-02
283	26660	HippSubfield_rh_volume_HATA	Hippocampus Subfield	regional and tissue volume	mm3	Volume of HATA in the right hemisphere generated by subcortical volumetric sub-segmentation of the Hippocampal Subfields	1.45E-01	1.73E-02
284	26661	HippSubfield_rh_volume_Whole-hippocampal-body	Hippocampus Subfield	regional and tissue volume	mm3	Volume of Whole-hippocampal-body in the right hemisphere generated by subcortical volumetric sub-segmentation of the Hippocampal Subfields	2.66E-01	2.50E-02
285	26662	HippSubfield_rh_volume_Whole-hippocampal-head	Hippocampus Subfield	regional and tissue volume	mm3	Volume of Whole-hippocampal-head in the right hemisphere generated by subcortical volumetric sub-segmentation of the Hippocampal Subfields	2.80E-01	2.52E-02
286	26663	HippSubfield_rh_volume_Whole-hippocampus	Hippocampus Subfield	regional and tissue volume	mm3	Volume of Whole-hippocampus in the right hemisphere generated by subcortical volumetric sub-segmentation of the Hippocampal Subfields	3.02E-01	2.67E-02
287	26664	ThalamNuclei_lh_volume_MGN	Thalamus Nuclei	regional and tissue volume	mm3	Volume of MGN in the left hemisphere generated by subcortical volumetric sub-segmentation of the Thalamic Nuclei	1.72E-01	1.93E-02
288	26665	ThalamNuclei_lh_volume_LGN	Thalamus Nuclei	regional and tissue volume	mm3	Volume of LGN in the left hemisphere generated by subcortical volumetric sub-segmentation of the Thalamic Nuclei	1.65E-01	1.85E-02
289	26666	ThalamNuclei_lh_volume_PuI	Thalamus Nuclei	regional and tissue volume	mm3	Volume of PuI in the left hemisphere generated by subcortical volumetric sub-segmentation of the Thalamic Nuclei	2.17E-01	1.74E-02
290	26667	ThalamNuclei_lh_volume_PuM	Thalamus Nuclei	regional and tissue volume	mm3	Volume of PuM in the left hemisphere generated by subcortical volumetric sub-segmentation of the Thalamic Nuclei	2.28E-01	2.01E-02

291	26668	ThalamNuclei_lh_volume_L-Sg	Thalamus Nuclei	regional and tissue volume	mm3	Volume of L-Sg in the left hemisphere generated by subcortical volumetric sub-segmentation of the Thalamic Nuclei	1.99E-01	1.99E-02
292	26669	ThalamNuclei_lh_volume_VPL	Thalamus Nuclei	regional and tissue volume	mm3	Volume of VPL in the left hemisphere generated by subcortical volumetric sub-segmentation of the Thalamic Nuclei	2.38E-01	2.06E-02
293	26670	ThalamNuclei_lh_volume_CM	Thalamus Nuclei	regional and tissue volume	mm3	Volume of CM in the left hemisphere generated by subcortical volumetric sub-segmentation of the Thalamic Nuclei	2.77E-01	2.39E-02
294	26671	ThalamNuclei_lh_volume_VLa	Thalamus Nuclei	regional and tissue volume	mm3	Volume of VLa in the left hemisphere generated by subcortical volumetric sub-segmentation of the Thalamic Nuclei	3.33E-01	2.51E-02
295	26672	ThalamNuclei_lh_volume_PuA	Thalamus Nuclei	regional and tissue volume	mm3	Volume of PuA in the left hemisphere generated by subcortical volumetric sub-segmentation of the Thalamic Nuclei	1.99E-01	1.95E-02
296	26673	ThalamNuclei_lh_volume_MDm	Thalamus Nuclei	regional and tissue volume	mm3	Volume of MDm in the left hemisphere generated by subcortical volumetric sub-segmentation of the Thalamic Nuclei	2.06E-01	2.00E-02
297	26674	ThalamNuclei_lh_volume_Pf	Thalamus Nuclei	regional and tissue volume	mm3	Volume of Pf in the left hemisphere generated by subcortical volumetric sub-segmentation of the Thalamic Nuclei	2.28E-01	2.17E-02
298	26675	ThalamNuclei_lh_volume_VAmc	Thalamus Nuclei	regional and tissue volume	mm3	Volume of VAmc in the left hemisphere generated by subcortical volumetric sub-segmentation of the Thalamic Nuclei	2.50E-01	2.34E-02
299	26676	ThalamNuclei_lh_volume_MDl	Thalamus Nuclei	regional and tissue volume	mm3	Volume of MDl in the left hemisphere generated by subcortical volumetric sub-segmentation of the Thalamic Nuclei	2.13E-01	2.02E-02
300	26677	ThalamNuclei_lh_volume_CeM	Thalamus Nuclei	regional and tissue volume	mm3	Volume of CeM in the left hemisphere generated by subcortical volumetric sub-segmentation of the Thalamic Nuclei	2.52E-01	2.09E-02
301	26678	ThalamNuclei_lh_volume_VA	Thalamus Nuclei	regional and tissue volume	mm3	Volume of VA in the left hemisphere generated by subcortical volumetric sub-segmentation of the Thalamic Nuclei	2.88E-01	2.17E-02

302	26679	ThalamNuclei_lh_volume_MV(Re)	Thalamus Nuclei	regional and tissue volume	mm3	Volume of MV(Re) in the left hemisphere generated by subcortical volumetric sub-segmentation of the Thalamic Nuclei	2.17E-01	1.95E-02
303	26680	ThalamNuclei_lh_volume_VM	Thalamus Nuclei	regional and tissue volume	mm3	Volume of VM in the left hemisphere generated by subcortical volumetric sub-segmentation of the Thalamic Nuclei	2.28E-01	2.03E-02
304	26681	ThalamNuclei_lh_volume_CL	Thalamus Nuclei	regional and tissue volume	mm3	Volume of CL in the left hemisphere generated by subcortical volumetric sub-segmentation of the Thalamic Nuclei	2.21E-01	1.92E-02
305	26682	ThalamNuclei_lh_volume_PuL	Thalamus Nuclei	regional and tissue volume	mm3	Volume of PuL in the left hemisphere generated by subcortical volumetric sub-segmentation of the Thalamic Nuclei	2.22E-01	1.93E-02
306	26683	ThalamNuclei_lh_volume_Pt	Thalamus Nuclei	regional and tissue volume	mm3	Volume of Pt in the left hemisphere generated by subcortical volumetric sub-segmentation of the Thalamic Nuclei	1.94E-01	1.79E-02
307	26684	ThalamNuclei_lh_volume_AV	Thalamus Nuclei	regional and tissue volume	mm3	Volume of AV in the left hemisphere generated by subcortical volumetric sub-segmentation of the Thalamic Nuclei	2.12E-01	1.82E-02
308	26685	ThalamNuclei_lh_volume_Pc	Thalamus Nuclei	regional and tissue volume	mm3	Volume of Pc in the left hemisphere generated by subcortical volumetric sub-segmentation of the Thalamic Nuclei	2.20E-01	2.43E-02
309	26686	ThalamNuclei_lh_volume_VLp	Thalamus Nuclei	regional and tissue volume	mm3	Volume of VLp in the left hemisphere generated by subcortical volumetric sub-segmentation of the Thalamic Nuclei	3.29E-01	2.48E-02
310	26687	ThalamNuclei_lh_volume_LP	Thalamus Nuclei	regional and tissue volume	mm3	Volume of LP in the left hemisphere generated by subcortical volumetric sub-segmentation of the Thalamic Nuclei	2.73E-01	1.97E-02
311	26688	ThalamNuclei_rh_volume_LGN	Thalamus Nuclei	regional and tissue volume	mm3	Volume of LGN in the right hemisphere generated by subcortical volumetric sub-segmentation of the Thalamic Nuclei	1.85E-01	1.81E-02
312	26689	ThalamNuclei_rh_volume_MGN	Thalamus Nuclei	regional and tissue volume	mm3	Volume of MGN in the right hemisphere generated by subcortical volumetric sub-segmentation of the Thalamic Nuclei	1.91E-01	1.83E-02

313	26690	ThalamNuclei_rh_volume_PuI	Thalamus Nuclei	regional and tissue volume	mm3	Volume of PuI in the right hemisphere generated by subcortical volumetric sub-segmentation of the Thalamic Nuclei	2.48E-01	2.04E-02
314	26691	ThalamNuclei_rh_volume_PuM	Thalamus Nuclei	regional and tissue volume	mm3	Volume of PuM in the right hemisphere generated by subcortical volumetric sub-segmentation of the Thalamic Nuclei	2.69E-01	2.11E-02
315	26692	ThalamNuclei_rh_volume_L-Sg	Thalamus Nuclei	regional and tissue volume	mm3	Volume of L-Sg in the right hemisphere generated by subcortical volumetric sub-segmentation of the Thalamic Nuclei	1.62E-01	2.02E-02
316	26693	ThalamNuclei_rh_volume_VPL	Thalamus Nuclei	regional and tissue volume	mm3	Volume of VPL in the right hemisphere generated by subcortical volumetric sub-segmentation of the Thalamic Nuclei	2.67E-01	2.14E-02
317	26694	ThalamNuclei_rh_volume_CM	Thalamus Nuclei	regional and tissue volume	mm3	Volume of CM in the right hemisphere generated by subcortical volumetric sub-segmentation of the Thalamic Nuclei	2.75E-01	2.39E-02
318	26695	ThalamNuclei_rh_volume_VLa	Thalamus Nuclei	regional and tissue volume	mm3	Volume of VLa in the right hemisphere generated by subcortical volumetric sub-segmentation of the Thalamic Nuclei	3.45E-01	2.52E-02
319	26696	ThalamNuclei_rh_volume_PuA	Thalamus Nuclei	regional and tissue volume	mm3	Volume of PuA in the right hemisphere generated by subcortical volumetric sub-segmentation of the Thalamic Nuclei	2.22E-01	1.94E-02
320	26697	ThalamNuclei_rh_volume_MDm	Thalamus Nuclei	regional and tissue volume	mm3	Volume of MDm in the right hemisphere generated by subcortical volumetric sub-segmentation of the Thalamic Nuclei	2.15E-01	2.06E-02
321	26698	ThalamNuclei_rh_volume_Pf	Thalamus Nuclei	regional and tissue volume	mm3	Volume of Pf in the right hemisphere generated by subcortical volumetric sub-segmentation of the Thalamic Nuclei	2.25E-01	2.32E-02
322	26699	ThalamNuclei_rh_volume_VAmc	Thalamus Nuclei	regional and tissue volume	mm3	Volume of VAmc in the right hemisphere generated by subcortical volumetric sub-segmentation of the Thalamic Nuclei	2.61E-01	2.28E-02
323	26700	ThalamNuclei_rh_volume_MDl	Thalamus Nuclei	regional and tissue volume	mm3	Volume of MDl in the right hemisphere generated by subcortical volumetric sub-segmentation of the Thalamic Nuclei	2.07E-01	2.04E-02

324	26701	ThalamNuclei_rh_volume_VA	Thalamus Nuclei	regional and tissue volume	mm3	Volume of VA in the right hemisphere generated by subcortical volumetric sub-segmentation of the Thalamic Nuclei	2.99E-01	2.31E-02
325	26702	ThalamNuclei_rh_volume_MV(Re)	Thalamus Nuclei	regional and tissue volume	mm3	Volume of MV(Re) in the right hemisphere generated by subcortical volumetric sub-segmentation of the Thalamic Nuclei	1.91E-01	1.98E-02
326	26703	ThalamNuclei_rh_volume_CeM	Thalamus Nuclei	regional and tissue volume	mm3	Volume of CeM in the right hemisphere generated by subcortical volumetric sub-segmentation of the Thalamic Nuclei	2.38E-01	2.10E-02
327	26704	ThalamNuclei_rh_volume_VM	Thalamus Nuclei	regional and tissue volume	mm3	Volume of VM in the right hemisphere generated by subcortical volumetric sub-segmentation of the Thalamic Nuclei	2.69E-01	2.35E-02
328	26705	ThalamNuclei_rh_volume_PuL	Thalamus Nuclei	regional and tissue volume	mm3	Volume of PuL in the right hemisphere generated by subcortical volumetric sub-segmentation of the Thalamic Nuclei	2.30E-01	2.07E-02
329	26706	ThalamNuclei_rh_volume_CL	Thalamus Nuclei	regional and tissue volume	mm3	Volume of CL in the right hemisphere generated by subcortical volumetric sub-segmentation of the Thalamic Nuclei	2.01E-01	1.73E-02
330	26707	ThalamNuclei_rh_volume_VLp	Thalamus Nuclei	regional and tissue volume	mm3	Volume of VLp in the right hemisphere generated by subcortical volumetric sub-segmentation of the Thalamic Nuclei	3.35E-01	2.45E-02
331	26708	ThalamNuclei_rh_volume_Pc	Thalamus Nuclei	regional and tissue volume	mm3	Volume of Pc in the right hemisphere generated by subcortical volumetric sub-segmentation of the Thalamic Nuclei	2.13E-01	2.06E-02
332	26709	ThalamNuclei_rh_volume_Pt	Thalamus Nuclei	regional and tissue volume	mm3	Volume of Pt in the right hemisphere generated by subcortical volumetric sub-segmentation of the Thalamic Nuclei	2.16E-01	1.92E-02
333	26710	ThalamNuclei_rh_volume_AV	Thalamus Nuclei	regional and tissue volume	mm3	Volume of AV in the right hemisphere generated by subcortical volumetric sub-segmentation of the Thalamic Nuclei	1.90E-01	1.83E-02
334	26711	ThalamNuclei_rh_volume_LP	Thalamus Nuclei	regional and tissue volume	mm3	Volume of LP in the right hemisphere generated by subcortical volumetric sub-segmentation of the Thalamic Nuclei	2.83E-01	2.14E-02

335	26712	ThalamNuclei_lh_volume_LD	Thalamus Nuclei	regional and tissue volume	mm3	Volume of LD in the left hemisphere generated by subcortical volumetric sub-segmentation of the Thalamic Nuclei	2.47E-01	2.07E-02
336	26713	ThalamNuclei_rh_volume_LD	Thalamus Nuclei	regional and tissue volume	mm3	Volume of LD in the right hemisphere generated by subcortical volumetric sub-segmentation of the Thalamic Nuclei	2.50E-01	1.86E-02
337	26714	ThalamNuclei_lh_volume_Whole-thalamus	Thalamus Nuclei	regional and tissue volume	mm3	Volume of Whole-thalamus in the left hemisphere generated by subcortical volumetric sub-segmentation of the Thalamic Nuclei	2.94E-01	2.29E-02
338	26715	ThalamNuclei_rh_volume_Whole-thalamus	Thalamus Nuclei	regional and tissue volume	mm3	Volume of Whole-thalamus in the right hemisphere generated by subcortical volumetric sub-segmentation of the Thalamic Nuclei	3.24E-01	2.29E-02
339	26716	Brainstem_global_volume_Medulla	Brain Stem	regional and tissue volume	mm3	Volume of Medulla in the whole brain generated by subcortical volumetric sub-segmentation of the Brainstem	4.03E-01	2.97E-02
340	26717	Brainstem_global_volume_Pons	Brain Stem	regional and tissue volume	mm3	Volume of Pons in the whole brain generated by subcortical volumetric sub-segmentation of the Brainstem	4.04E-01	3.18E-02
341	26718	Brainstem_global_volume_SCP	Brain Stem	regional and tissue volume	mm3	Volume of SCP in the whole brain generated by subcortical volumetric sub-segmentation of the Brainstem	2.48E-01	2.08E-02
342	26719	Brainstem_global_volume_Midbrain	Brain Stem	regional and tissue volume	mm3	Volume of Midbrain in the whole brain generated by subcortical volumetric sub-segmentation of the Brainstem	3.69E-01	2.94E-02
343	26720	Brainstem_global_volume_Whole-brainstem	Brain Stem	regional and tissue volume	mm3	Volume of Whole-brainstem in the whole brain generated by subcortical volumetric sub-segmentation of the Brainstem	4.05E-01	3.11E-02
344	26789	aparc-Desikan_lh_volume_bankssts	Desikan Atlas	regional and tissue volume	mm3	Volume of bankssts in the left hemisphere generated by parcellation of the white surface using Desikan-Killiany parcellation	1.49E-01	1.69E-02
345	26790	aparc-Desikan_lh_volume_caudalanteriorcingulate	Desikan Atlas	regional and tissue volume	mm3	Volume of caudalanteriorcingulate in the left hemisphere generated by parcellation of the white surface using Desikan-Killiany parcellation	8.85E-02	1.48E-02
346	26791	aparc-Desikan_lh_volume_caudalmiddlefrontal	Desikan Atlas	regional and tissue volume	mm3	Volume of caudalmiddlefrontal in the left hemisphere generated by parcellation of the white surface using	1.47E-01	2.11E-02

						Desikan-Killiany parcellation		
347	26792	aparc-Desikan_lh_volume_cuneus	Desikan Atlas	regional and tissue volume	mm3	Volume of cuneus in the left hemisphere generated by parcellation of the white surface using Desikan-Killiany parcellation	2.62E-01	2.44E-02
348	26793	aparc-Desikan_lh_volume_entorhinal	Desikan Atlas	regional and tissue volume	mm3	Volume of entorhinal in the left hemisphere generated by parcellation of the white surface using Desikan-Killiany parcellation	1.21E-01	1.63E-02
349	26794	aparc-Desikan_lh_volume_fusiform	Desikan Atlas	regional and tissue volume	mm3	Volume of fusiform in the left hemisphere generated by parcellation of the white surface using Desikan-Killiany parcellation	1.64E-01	1.83E-02
350	26795	aparc-Desikan_lh_volume_inferiorparietal	Desikan Atlas	regional and tissue volume	mm3	Volume of inferiorparietal in the left hemisphere generated by parcellation of the white surface using Desikan-Killiany parcellation	1.90E-01	1.92E-02
351	26796	aparc-Desikan_lh_volume_inferiortemporal	Desikan Atlas	regional and tissue volume	mm3	Volume of inferiortemporal in the left hemisphere generated by parcellation of the white surface using Desikan-Killiany parcellation	1.74E-01	1.79E-02
352	26797	aparc-Desikan_lh_volume_isthmuscingulate	Desikan Atlas	regional and tissue volume	mm3	Volume of isthmuscingulate in the left hemisphere generated by parcellation of the white surface using Desikan-Killiany parcellation	1.68E-01	1.92E-02
353	26798	aparc-Desikan_lh_volume_lateraloccipital	Desikan Atlas	regional and tissue volume	mm3	Volume of lateraloccipital in the left hemisphere generated by parcellation of the white surface using Desikan-Killiany parcellation	1.89E-01	1.89E-02
354	26799	aparc-Desikan_lh_volume_lateralorbitofrontal	Desikan Atlas	regional and tissue volume	mm3	Volume of lateralorbitofrontal in the left hemisphere generated by parcellation of the white surface using Desikan-Killiany parcellation	2.57E-01	2.32E-02
355	26800	aparc-Desikan_lh_volume_lingual	Desikan Atlas	regional and tissue volume	mm3	Volume of lingual in the left hemisphere generated by parcellation of the white surface using Desikan-Killiany parcellation	2.24E-01	2.25E-02
356	26801	aparc-Desikan_lh_volume_medialorbitofrontal	Desikan Atlas	regional and tissue volume	mm3	Volume of medialorbitofrontal in the left hemisphere generated by parcellation of the white surface using Desikan-Killiany parcellation	1.24E-01	1.96E-02

357	26802	aparc-Desikan_lh_volume_middletemporal	Desikan Atlas	regional and tissue volume	mm3	Volume of middletemporal in the left hemisphere generated by parcellation of the white surface using Desikan-Killiany parcellation	1.98E-01	1.98E-02
358	26803	aparc-Desikan_lh_volume_parahippocampal	Desikan Atlas	regional and tissue volume	mm3	Volume of parahippocampal in the left hemisphere generated by parcellation of the white surface using Desikan-Killiany parcellation	2.07E-01	1.94E-02
359	26804	aparc-Desikan_lh_volume_paracentral	Desikan Atlas	regional and tissue volume	mm3	Volume of paracentral in the left hemisphere generated by parcellation of the white surface using Desikan-Killiany parcellation	1.60E-01	1.94E-02
360	26805	aparc-Desikan_lh_volume_parsopercularis	Desikan Atlas	regional and tissue volume	mm3	Volume of parsopercularis in the left hemisphere generated by parcellation of the white surface using Desikan-Killiany parcellation	1.20E-01	1.64E-02
361	26806	aparc-Desikan_lh_volume_parsorbitalis	Desikan Atlas	regional and tissue volume	mm3	Volume of parsorbitalis in the left hemisphere generated by parcellation of the white surface using Desikan-Killiany parcellation	1.51E-01	1.74E-02
362	26807	aparc-Desikan_lh_volume_parstriangularis	Desikan Atlas	regional and tissue volume	mm3	Volume of parstriangularis in the left hemisphere generated by parcellation of the white surface using Desikan-Killiany parcellation	1.25E-01	1.82E-02
363	26808	aparc-Desikan_lh_volume_pericalcarine	Desikan Atlas	regional and tissue volume	mm3	Volume of pericalcarine in the left hemisphere generated by parcellation of the white surface using Desikan-Killiany parcellation	2.87E-01	2.75E-02
364	26809	aparc-Desikan_lh_volume_postcentral	Desikan Atlas	regional and tissue volume	mm3	Volume of postcentral in the left hemisphere generated by parcellation of the white surface using Desikan-Killiany parcellation	1.55E-01	1.98E-02
365	26810	aparc-Desikan_lh_volume_posteriorcingulate	Desikan Atlas	regional and tissue volume	mm3	Volume of posteriorcingulate in the left hemisphere generated by parcellation of the white surface using Desikan-Killiany parcellation	1.22E-01	2.16E-02
366	26811	aparc-Desikan_lh_volume_precentral	Desikan Atlas	regional and tissue volume	mm3	Volume of precentral in the left hemisphere generated by parcellation of the white surface using Desikan-Killiany parcellation	1.87E-01	2.03E-02
367	26812	aparc-Desikan_lh_volume_precuneus	Desikan Atlas	regional and tissue volume	mm3	Volume of precuneus in the left hemisphere generated by parcellation of the white surface using Desikan-Killiany parcellation	2.33E-01	2.21E-02

368	26813	aparc-Desikan_lh_volume_rostralanteriorcingulate	Desikan Atlas	regional and tissue volume	mm3	Volume of rostralanteriorcingulate in the left hemisphere generated by parcellation of the white surface using Desikan-Killiany parcellation	1.75E-01	1.73E-02
369	26814	aparc-Desikan_lh_volume_rostralmiddlefrontal	Desikan Atlas	regional and tissue volume	mm3	Volume of rostralmiddlefrontal in the left hemisphere generated by parcellation of the white surface using Desikan-Killiany parcellation	2.01E-01	1.69E-02
370	26815	aparc-Desikan_lh_volume_superiorfrontal	Desikan Atlas	regional and tissue volume	mm3	Volume of superiorfrontal in the left hemisphere generated by parcellation of the white surface using Desikan-Killiany parcellation	2.12E-01	2.19E-02
371	26816	aparc-Desikan_lh_volume_superiorparietal	Desikan Atlas	regional and tissue volume	mm3	Volume of superiorparietal in the left hemisphere generated by parcellation of the white surface using Desikan-Killiany parcellation	1.87E-01	1.96E-02
372	26817	aparc-Desikan_lh_volume_superiortemporal	Desikan Atlas	regional and tissue volume	mm3	Volume of superiortemporal in the left hemisphere generated by parcellation of the white surface using Desikan-Killiany parcellation	2.23E-01	2.31E-02
373	26818	aparc-Desikan_lh_volume_supramarginal	Desikan Atlas	regional and tissue volume	mm3	Volume of supramarginal in the left hemisphere generated by parcellation of the white surface using Desikan-Killiany parcellation	1.70E-01	1.88E-02
374	26819	aparc-Desikan_lh_volume_frontalpole	Desikan Atlas	regional and tissue volume	mm3	Volume of frontalpole in the left hemisphere generated by parcellation of the white surface using Desikan-Killiany parcellation	7.41E-02	1.68E-02
375	26820	aparc-Desikan_lh_volume_transversetemporal	Desikan Atlas	regional and tissue volume	mm3	Volume of transversetemporal in the left hemisphere generated by parcellation of the white surface using Desikan-Killiany parcellation	1.65E-01	1.83E-02
376	26821	aparc-Desikan_lh_volume_insula	Desikan Atlas	regional and tissue volume	mm3	Volume of insula in the left hemisphere generated by parcellation of the white surface using Desikan-Killiany parcellation	2.37E-01	2.13E-02
377	26890	aparc-Desikan_rh_volume_bankssts	Desikan Atlas	regional and tissue volume	mm3	Volume of bankssts in the right hemisphere generated by parcellation of the white surface using Desikan-Killiany parcellation	1.45E-01	1.90E-02
378	26891	aparc-Desikan_rh_volume_caudalanteriorcingulate	Desikan Atlas	regional and tissue volume	mm3	Volume of caudalanteriorcingulate in the right hemisphere generated by parcellation of the white surface using Desikan-Killiany parcellation	8.10E-02	1.53E-02

379	26892	aparc-Desikan_rh_volume_caudalmiddlefrontal	Desikan Atlas	regional and tissue volume	mm3	Volume of caudalmiddlefrontal in the right hemisphere generated by parcellation of the white surface using Desikan-Killiany parcellation	1.87E-01	1.96E-02
380	26893	aparc-Desikan_rh_volume_cuneus	Desikan Atlas	regional and tissue volume	mm3	Volume of cuneus in the right hemisphere generated by parcellation of the white surface using Desikan-Killiany parcellation	2.67E-01	2.43E-02
381	26894	aparc-Desikan_rh_volume_entorhinal	Desikan Atlas	regional and tissue volume	mm3	Volume of entorhinal in the right hemisphere generated by parcellation of the white surface using Desikan-Killiany parcellation	1.26E-01	1.71E-02
382	26895	aparc-Desikan_rh_volume_fusiform	Desikan Atlas	regional and tissue volume	mm3	Volume of fusiform in the right hemisphere generated by parcellation of the white surface using Desikan-Killiany parcellation	1.91E-01	1.78E-02
383	26896	aparc-Desikan_rh_volume_inferiorparietal	Desikan Atlas	regional and tissue volume	mm3	Volume of inferiorparietal in the right hemisphere generated by parcellation of the white surface using Desikan-Killiany parcellation	1.59E-01	1.86E-02
384	26897	aparc-Desikan_rh_volume_inferiortemporal	Desikan Atlas	regional and tissue volume	mm3	Volume of inferiortemporal in the right hemisphere generated by parcellation of the white surface using Desikan-Killiany parcellation	1.87E-01	1.78E-02
385	26898	aparc-Desikan_rh_volume_isthmuscingulate	Desikan Atlas	regional and tissue volume	mm3	Volume of isthmuscingulate in the right hemisphere generated by parcellation of the white surface using Desikan-Killiany parcellation	1.26E-01	1.88E-02
386	26899	aparc-Desikan_rh_volume_lateraloccipital	Desikan Atlas	regional and tissue volume	mm3	Volume of lateraloccipital in the right hemisphere generated by parcellation of the white surface using Desikan-Killiany parcellation	2.03E-01	2.11E-02
387	26900	aparc-Desikan_rh_volume_lateralorbitofrontal	Desikan Atlas	regional and tissue volume	mm3	Volume of lateralorbitofrontal in the right hemisphere generated by parcellation of the white surface using Desikan-Killiany parcellation	2.06E-01	1.94E-02
388	26901	aparc-Desikan_rh_volume_lingual	Desikan Atlas	regional and tissue volume	mm3	Volume of lingual in the right hemisphere generated by parcellation of the white surface using Desikan-Killiany parcellation	2.24E-01	2.40E-02
389	26902	aparc-Desikan_rh_volume_medialorbitofrontal	Desikan Atlas	regional and tissue volume	mm3	Volume of medialorbitofrontal in the right hemisphere generated by parcellation of the white surface using Desikan-Killiany parcellation	1.59E-01	1.73E-02

390	26903	aparc-Desikan_rh_volume_middletemporal	Desikan Atlas	regional and tissue volume	mm3	Volume of middletemporal in the right hemisphere generated by parcellation of the white surface using Desikan-Killiany parcellation	2.03E-01	2.09E-02
391	26904	aparc-Desikan_rh_volume_parahippocampal	Desikan Atlas	regional and tissue volume	mm3	Volume of parahippocampal in the right hemisphere generated by parcellation of the white surface using Desikan-Killiany parcellation	1.92E-01	1.86E-02
392	26905	aparc-Desikan_rh_volume_paracentral	Desikan Atlas	regional and tissue volume	mm3	Volume of paracentral in the right hemisphere generated by parcellation of the white surface using Desikan-Killiany parcellation	1.29E-01	1.72E-02
393	26906	aparc-Desikan_rh_volume_parsopercularis	Desikan Atlas	regional and tissue volume	mm3	Volume of parsopercularis in the right hemisphere generated by parcellation of the white surface using Desikan-Killiany parcellation	1.28E-01	1.79E-02
394	26907	aparc-Desikan_rh_volume_parsorbitalis	Desikan Atlas	regional and tissue volume	mm3	Volume of parsorbitalis in the right hemisphere generated by parcellation of the white surface using Desikan-Killiany parcellation	1.64E-01	1.75E-02
395	26908	aparc-Desikan_rh_volume_parstriangularis	Desikan Atlas	regional and tissue volume	mm3	Volume of parstriangularis in the right hemisphere generated by parcellation of the white surface using Desikan-Killiany parcellation	1.25E-01	2.05E-02
396	26909	aparc-Desikan_rh_volume_pericalcarine	Desikan Atlas	regional and tissue volume	mm3	Volume of pericalcarine in the right hemisphere generated by parcellation of the white surface using Desikan-Killiany parcellation	3.14E-01	2.93E-02
397	26910	aparc-Desikan_rh_volume_postcentral	Desikan Atlas	regional and tissue volume	mm3	Volume of postcentral in the right hemisphere generated by parcellation of the white surface using Desikan-Killiany parcellation	1.81E-01	1.96E-02
398	26911	aparc-Desikan_rh_volume_posteriorcingulate	Desikan Atlas	regional and tissue volume	mm3	Volume of posteriorcingulate in the right hemisphere generated by parcellation of the white surface using Desikan-Killiany parcellation	1.25E-01	1.71E-02
399	26912	aparc-Desikan_rh_volume_precentral	Desikan Atlas	regional and tissue volume	mm3	Volume of precentral in the right hemisphere generated by parcellation of the white surface using Desikan-Killiany parcellation	2.23E-01	2.20E-02
400	26913	aparc-Desikan_rh_volume_precuneus	Desikan Atlas	regional and tissue volume	mm3	Volume of precuneus in the right hemisphere generated by parcellation of the white surface using Desikan-Killiany parcellation	2.27E-01	2.13E-02

401	26914	aparc-Desikan_rh_volume_rostralanteriorcingulate	Desikan Atlas	regional and tissue volume	mm3	Volume of rostralanteriorcingulate in the right hemisphere generated by parcellation of the white surface using Desikan-Killiany parcellation	1.04E-01	1.40E-02
402	26915	aparc-Desikan_rh_volume_rostralmiddlefrontal	Desikan Atlas	regional and tissue volume	mm3	Volume of rostralmiddlefrontal in the right hemisphere generated by parcellation of the white surface using Desikan-Killiany parcellation	1.90E-01	1.76E-02
403	26916	aparc-Desikan_rh_volume_superiorfrontal	Desikan Atlas	regional and tissue volume	mm3	Volume of superiorfrontal in the right hemisphere generated by parcellation of the white surface using Desikan-Killiany parcellation	1.88E-01	1.75E-02
404	26917	aparc-Desikan_rh_volume_superiorparietal	Desikan Atlas	regional and tissue volume	mm3	Volume of superiorparietal in the right hemisphere generated by parcellation of the white surface using Desikan-Killiany parcellation	1.71E-01	1.97E-02
405	26918	aparc-Desikan_rh_volume_superiortemporal	Desikan Atlas	regional and tissue volume	mm3	Volume of superiortemporal in the right hemisphere generated by parcellation of the white surface using Desikan-Killiany parcellation	2.25E-01	1.78E-02
406	26919	aparc-Desikan_rh_volume_supramarginal	Desikan Atlas	regional and tissue volume	mm3	Volume of supramarginal in the right hemisphere generated by parcellation of the white surface using Desikan-Killiany parcellation	1.54E-01	1.77E-02
407	26920	aparc-Desikan_rh_volume_frontalpole	Desikan Atlas	regional and tissue volume	mm3	Volume of frontalpole in the right hemisphere generated by parcellation of the white surface using Desikan-Killiany parcellation	7.18E-02	1.62E-02
408	26921	aparc-Desikan_rh_volume_transversetemporal	Desikan Atlas	regional and tissue volume	mm3	Volume of transversetemporal in the right hemisphere generated by parcellation of the white surface using Desikan-Killiany parcellation	1.47E-01	1.78E-02
409	26922	aparc-Desikan_rh_volume_insula	Desikan Atlas	regional and tissue volume	mm3	Volume of insula in the right hemisphere generated by parcellation of the white surface using Desikan-Killiany parcellation	2.39E-01	2.17E-02
410	27087	BA-exvivo_lh_volume_BA1	Broadmann Atlas	regional and tissue volume	mm3	Volume of BA1 in the left hemisphere generated by parcellation of the white surface using BA_exvivo parcellation	1.57E-01	1.87E-02
411	27088	BA-exvivo_lh_volume_BA2	Broadmann Atlas	regional and tissue volume	mm3	Volume of BA2 in the left hemisphere generated by parcellation of the white surface using BA_exvivo parcellation	1.25E-01	1.84E-02

412	27089	BA-exvivo_lh_volume_BA3a	Broadmann Atlas	regional and tissue volume	mm3	Volume of BA3a in the left hemisphere generated by parcellation of the white surface using BA_exvivo parcellation	1.33E-01	1.77E-02
413	27090	BA-exvivo_lh_volume_BA3b	Broadmann Atlas	regional and tissue volume	mm3	Volume of BA3b in the left hemisphere generated by parcellation of the white surface using BA_exvivo parcellation	1.73E-01	2.23E-02
414	27091	BA-exvivo_lh_volume_BA4a	Broadmann Atlas	regional and tissue volume	mm3	Volume of BA4a in the left hemisphere generated by parcellation of the white surface using BA_exvivo parcellation	1.71E-01	1.72E-02
415	27092	BA-exvivo_lh_volume_BA4p	Broadmann Atlas	regional and tissue volume	mm3	Volume of BA4p in the left hemisphere generated by parcellation of the white surface using BA_exvivo parcellation	1.27E-01	1.78E-02
416	27093	BA-exvivo_lh_volume_BA6	Broadmann Atlas	regional and tissue volume	mm3	Volume of BA6 in the left hemisphere generated by parcellation of the white surface using BA_exvivo parcellation	2.06E-01	2.02E-02
417	27094	BA-exvivo_lh_volume_BA44	Broadmann Atlas	regional and tissue volume	mm3	Volume of BA44 in the left hemisphere generated by parcellation of the white surface using BA_exvivo parcellation	1.32E-01	1.61E-02
418	27095	BA-exvivo_lh_volume_BA45	Broadmann Atlas	regional and tissue volume	mm3	Volume of BA45 in the left hemisphere generated by parcellation of the white surface using BA_exvivo parcellation	1.47E-01	1.82E-02
419	27096	BA-exvivo_lh_volume_V1	Broadmann Atlas	regional and tissue volume	mm3	Volume of V1 in the left hemisphere generated by parcellation of the white surface using BA_exvivo parcellation	2.75E-01	2.62E-02
420	27097	BA-exvivo_lh_volume_V2	Broadmann Atlas	regional and tissue volume	mm3	Volume of V2 in the left hemisphere generated by parcellation of the white surface using BA_exvivo parcellation	2.43E-01	2.25E-02
421	27098	BA-exvivo_lh_volume_MT	Broadmann Atlas	regional and tissue volume	mm3	Volume of MT in the left hemisphere generated by parcellation of the white surface using BA_exvivo parcellation	1.26E-01	1.81E-02
422	27099	BA-exvivo_lh_volume_perirhinal	Broadmann Atlas	regional and tissue volume	mm3	Volume of perirhinal in the left hemisphere generated by parcellation of the white surface using BA_exvivo parcellation	1.29E-01	1.87E-02

423	27100	BA-exvivo_lh_volume_entorhinal	Broadmann Atlas	regional and tissue volume	mm3	Volume of entorhinal in the left hemisphere generated by parcellation of the white surface using BA_exvivo parcellation	1.95E-01	2.10E-02
424	27129	BA-exvivo_rh_volume_BA1	Broadmann Atlas	regional and tissue volume	mm3	Volume of BA1 in the right hemisphere generated by parcellation of the white surface using BA_exvivo parcellation	1.63E-01	1.88E-02
425	27130	BA-exvivo_rh_volume_BA2	Broadmann Atlas	regional and tissue volume	mm3	Volume of BA2 in the right hemisphere generated by parcellation of the white surface using BA_exvivo parcellation	1.20E-01	1.48E-02
426	27131	BA-exvivo_rh_volume_BA3a	Broadmann Atlas	regional and tissue volume	mm3	Volume of BA3a in the right hemisphere generated by parcellation of the white surface using BA_exvivo parcellation	1.68E-01	2.03E-02
427	27132	BA-exvivo_rh_volume_BA3b	Broadmann Atlas	regional and tissue volume	mm3	Volume of BA3b in the right hemisphere generated by parcellation of the white surface using BA_exvivo parcellation	1.85E-01	2.98E-02
428	27133	BA-exvivo_rh_volume_BA4a	Broadmann Atlas	regional and tissue volume	mm3	Volume of BA4a in the right hemisphere generated by parcellation of the white surface using BA_exvivo parcellation	1.50E-01	1.89E-02
429	27134	BA-exvivo_rh_volume_BA4p	Broadmann Atlas	regional and tissue volume	mm3	Volume of BA4p in the right hemisphere generated by parcellation of the white surface using BA_exvivo parcellation	1.66E-01	1.99E-02
430	27135	BA-exvivo_rh_volume_BA6	Broadmann Atlas	regional and tissue volume	mm3	Volume of BA6 in the right hemisphere generated by parcellation of the white surface using BA_exvivo parcellation	2.17E-01	1.96E-02
431	27136	BA-exvivo_rh_volume_BA44	Broadmann Atlas	regional and tissue volume	mm3	Volume of BA44 in the right hemisphere generated by parcellation of the white surface using BA_exvivo parcellation	1.81E-01	1.69E-02
432	27137	BA-exvivo_rh_volume_BA45	Broadmann Atlas	regional and tissue volume	mm3	Volume of BA45 in the right hemisphere generated by parcellation of the white surface using BA_exvivo parcellation	1.33E-01	1.83E-02
433	27138	BA-exvivo_rh_volume_V1	Broadmann Atlas	regional and tissue volume	mm3	Volume of V1 in the right hemisphere generated by parcellation of the white surface using BA_exvivo parcellation	2.85E-01	2.85E-02

434	27139	BA-exvivo_rh_volume_V2	Broadmann Atlas	regional and tissue volume	mm3	Volume of V2 in the right hemisphere generated by parcellation of the white surface using BA_exvivo parcellation	2.63E-01	2.54E-02
435	27140	BA-exvivo_rh_volume_MT	Broadmann Atlas	regional and tissue volume	mm3	Volume of MT in the right hemisphere generated by parcellation of the white surface using BA_exvivo parcellation	1.36E-01	1.72E-02
436	27141	BA-exvivo_rh_volume_perirhinal	Broadmann Atlas	regional and tissue volume	mm3	Volume of perirhinal in the right hemisphere generated by parcellation of the white surface using BA_exvivo parcellation	9.57E-02	1.57E-02
437	27142	BA-exvivo_rh_volume_entorhinal	Broadmann Atlas	regional and tissue volume	mm3	Volume of entorhinal in the right hemisphere generated by parcellation of the white surface using BA_exvivo parcellation	2.02E-01	2.04E-02
438	27205	aparc-DKTatlas_lh_volume_caudalanteriorcingulate	Desikan Atlas	regional and tissue volume	mm3	Volume of caudalanteriorcingulate in the left hemisphere generated by parcellation of the white surface using DKT parcellation	1.77E-01	1.95E-02
439	27206	aparc-DKTatlas_lh_volume_caudalmiddlefrontal	Desikan Atlas	regional and tissue volume	mm3	Volume of caudalmiddlefrontal in the left hemisphere generated by parcellation of the white surface using DKT parcellation	1.48E-01	2.06E-02
440	27207	aparc-DKTatlas_lh_volume_cuneus	Desikan Atlas	regional and tissue volume	mm3	Volume of cuneus in the left hemisphere generated by parcellation of the white surface using DKT parcellation	2.50E-01	2.29E-02
441	27208	aparc-DKTatlas_lh_volume_entorhinal	Desikan Atlas	regional and tissue volume	mm3	Volume of entorhinal in the left hemisphere generated by parcellation of the white surface using DKT parcellation	1.28E-01	1.73E-02
442	27209	aparc-DKTatlas_lh_volume_fusiform	Desikan Atlas	regional and tissue volume	mm3	Volume of fusiform in the left hemisphere generated by parcellation of the white surface using DKT parcellation	1.69E-01	1.80E-02
443	27210	aparc-DKTatlas_lh_volume_inferiorparietal	Desikan Atlas	regional and tissue volume	mm3	Volume of inferiorparietal in the left hemisphere generated by parcellation of the white surface using DKT parcellation	1.97E-01	1.98E-02
444	27211	aparc-DKTatlas_lh_volume_inferiortemporal	Desikan Atlas	regional and tissue volume	mm3	Volume of inferiortemporal in the left hemisphere generated by parcellation of the white surface using DKT parcellation	1.66E-01	1.80E-02
445	27212	aparc-DKTatlas_lh_volume_isthmuscingulate	Desikan Atlas	regional and tissue volume	mm3	Volume of isthmuscingulate in the left hemisphere generated by parcellation of the white surface using DKT	1.77E-01	1.95E-02

						parcellation		
446	27213	aparc-DKTatlas_lh_volume_lateraloccipital	Desikan Atlas	regional and tissue volume	mm3	Volume of lateraloccipital in the left hemisphere generated by parcellation of the white surface using DKT parcellation	1.92E-01	1.90E-02
447	27214	aparc-DKTatlas_lh_volume_lateralorbitofrontal	Desikan Atlas	regional and tissue volume	mm3	Volume of lateralorbitofrontal in the left hemisphere generated by parcellation of the white surface using DKT parcellation	2.42E-01	2.27E-02
448	27215	aparc-DKTatlas_lh_volume_lingual	Desikan Atlas	regional and tissue volume	mm3	Volume of lingual in the left hemisphere generated by parcellation of the white surface using DKT parcellation	2.22E-01	2.26E-02
449	27216	aparc-DKTatlas_lh_volume_medialorbitofrontal	Desikan Atlas	regional and tissue volume	mm3	Volume of medialorbitofrontal in the left hemisphere generated by parcellation of the white surface using DKT parcellation	1.60E-01	1.83E-02
450	27217	aparc-DKTatlas_lh_volume_middletemporal	Desikan Atlas	regional and tissue volume	mm3	Volume of middletemporal in the left hemisphere generated by parcellation of the white surface using DKT parcellation	2.20E-01	2.06E-02
451	27218	aparc-DKTatlas_lh_volume_parahippocampal	Desikan Atlas	regional and tissue volume	mm3	Volume of parahippocampal in the left hemisphere generated by parcellation of the white surface using DKT parcellation	2.10E-01	1.97E-02
452	27219	aparc-DKTatlas_lh_volume_paracentral	Desikan Atlas	regional and tissue volume	mm3	Volume of paracentral in the left hemisphere generated by parcellation of the white surface using DKT parcellation	1.68E-01	1.91E-02
453	27220	aparc-DKTatlas_lh_volume_parsopercularis	Desikan Atlas	regional and tissue volume	mm3	Volume of parsopercularis in the left hemisphere generated by parcellation of the white surface using DKT parcellation	1.13E-01	1.64E-02
454	27221	aparc-DKTatlas_lh_volume_parsorbitalis	Desikan Atlas	regional and tissue volume	mm3	Volume of parsorbitalis in the left hemisphere generated by parcellation of the white surface using DKT parcellation	1.60E-01	1.73E-02
455	27222	aparc-DKTatlas_lh_volume_parstriangularis	Desikan Atlas	regional and tissue volume	mm3	Volume of parstriangularis in the left hemisphere generated by parcellation of the white surface using DKT parcellation	1.36E-01	1.85E-02
456	27223	aparc-DKTatlas_lh_volume_pericalcarine	Desikan Atlas	regional and tissue volume	mm3	Volume of pericalcarine in the left hemisphere generated by parcellation of the white surface using DKT parcellation	2.88E-01	2.77E-02

457	27224	aparc-DKTatlas_lh_volume_postcentral	Desikan Atlas	regional and tissue volume	mm3	Volume of postcentral in the left hemisphere generated by parcellation of the white surface using DKT parcellation	1.54E-01	1.89E-02
458	27225	aparc-DKTatlas_lh_volume_posteriorcingulate	Desikan Atlas	regional and tissue volume	mm3	Volume of posteriorcingulate in the left hemisphere generated by parcellation of the white surface using DKT parcellation	1.33E-01	2.30E-02
459	27226	aparc-DKTatlas_lh_volume_precentral	Desikan Atlas	regional and tissue volume	mm3	Volume of precentral in the left hemisphere generated by parcellation of the white surface using DKT parcellation	1.87E-01	2.04E-02
460	27227	aparc-DKTatlas_lh_volume_precuneus	Desikan Atlas	regional and tissue volume	mm3	Volume of precuneus in the left hemisphere generated by parcellation of the white surface using DKT parcellation	2.35E-01	2.23E-02
461	27228	aparc-DKTatlas_lh_volume_rostralanteriorcingulate	Desikan Atlas	regional and tissue volume	mm3	Volume of rostralanteriorcingulate in the left hemisphere generated by parcellation of the white surface using DKT parcellation	1.70E-01	2.02E-02
462	27229	aparc-DKTatlas_lh_volume_rostralmiddlefrontal	Desikan Atlas	regional and tissue volume	mm3	Volume of rostralmiddlefrontal in the left hemisphere generated by parcellation of the white surface using DKT parcellation	1.86E-01	1.67E-02
463	27230	aparc-DKTatlas_lh_volume_superiorfrontal	Desikan Atlas	regional and tissue volume	mm3	Volume of superiorfrontal in the left hemisphere generated by parcellation of the white surface using DKT parcellation	1.92E-01	2.13E-02
464	27231	aparc-DKTatlas_lh_volume_superiorparietal	Desikan Atlas	regional and tissue volume	mm3	Volume of superiorparietal in the left hemisphere generated by parcellation of the white surface using DKT parcellation	1.84E-01	1.94E-02
465	27232	aparc-DKTatlas_lh_volume_superiortemporal	Desikan Atlas	regional and tissue volume	mm3	Volume of superiortemporal in the left hemisphere generated by parcellation of the white surface using DKT parcellation	2.08E-01	2.14E-02
466	27233	aparc-DKTatlas_lh_volume_supramarginal	Desikan Atlas	regional and tissue volume	mm3	Volume of supramarginal in the left hemisphere generated by parcellation of the white surface using DKT parcellation	1.74E-01	1.76E-02
467	27234	aparc-DKTatlas_lh_volume_transversetemporal	Desikan Atlas	regional and tissue volume	mm3	Volume of transversetemporal in the left hemisphere generated by parcellation of the white surface using DKT parcellation	1.67E-01	1.86E-02
468	27235	aparc-DKTatlas_lh_volume_insula	Desikan Atlas	regional and tissue volume	mm3	Volume of insula in the left hemisphere generated by parcellation of the white surface using DKT parcellation	2.82E-01	2.36E-02

469	27298	aparc-DKTatlas_rh_volume_caudalanteriorcingulate	Desikan Atlas	regional and tissue volume	mm3	Volume of caudalanteriorcingulate in the right hemisphere generated by parcellation of the white surface using DKT parcellation	8.05E-02	1.55E-02
470	27299	aparc-DKTatlas_rh_volume_caudalmiddlefrontal	Desikan Atlas	regional and tissue volume	mm3	Volume of caudalmiddlefrontal in the right hemisphere generated by parcellation of the white surface using DKT parcellation	1.94E-01	1.98E-02
471	27300	aparc-DKTatlas_rh_volume_cuneus	Desikan Atlas	regional and tissue volume	mm3	Volume of cuneus in the right hemisphere generated by parcellation of the white surface using DKT parcellation	2.54E-01	2.36E-02
472	27301	aparc-DKTatlas_rh_volume_entorhinal	Desikan Atlas	regional and tissue volume	mm3	Volume of entorhinal in the right hemisphere generated by parcellation of the white surface using DKT parcellation	1.16E-01	1.77E-02
473	27302	aparc-DKTatlas_rh_volume_fusiform	Desikan Atlas	regional and tissue volume	mm3	Volume of fusiform in the right hemisphere generated by parcellation of the white surface using DKT parcellation	1.87E-01	1.80E-02
474	27303	aparc-DKTatlas_rh_volume_inferiorparietal	Desikan Atlas	regional and tissue volume	mm3	Volume of inferiorparietal in the right hemisphere generated by parcellation of the white surface using DKT parcellation	1.61E-01	1.86E-02
475	27304	aparc-DKTatlas_rh_volume_inferiortemporal	Desikan Atlas	regional and tissue volume	mm3	Volume of inferiortemporal in the right hemisphere generated by parcellation of the white surface using DKT parcellation	1.86E-01	1.80E-02
476	27305	aparc-DKTatlas_rh_volume_isthmuscingulate	Desikan Atlas	regional and tissue volume	mm3	Volume of isthmuscingulate in the right hemisphere generated by parcellation of the white surface using DKT parcellation	1.26E-01	1.86E-02
477	27306	aparc-DKTatlas_rh_volume_lateraloccipital	Desikan Atlas	regional and tissue volume	mm3	Volume of lateraloccipital in the right hemisphere generated by parcellation of the white surface using DKT parcellation	2.11E-01	2.18E-02
478	27307	aparc-DKTatlas_rh_volume_lateralorbitofrontal	Desikan Atlas	regional and tissue volume	mm3	Volume of lateralorbitofrontal in the right hemisphere generated by parcellation of the white surface using DKT parcellation	2.28E-01	2.10E-02
479	27308	aparc-DKTatlas_rh_volume_lingual	Desikan Atlas	regional and tissue volume	mm3	Volume of lingual in the right hemisphere generated by parcellation of the white surface using DKT parcellation	2.24E-01	2.40E-02
480	27309	aparc-DKTatlas_rh_volume_medialorbitofrontal	Desikan Atlas	regional and tissue volume	mm3	Volume of medialorbitofrontal in the right hemisphere generated by parcellation of the white surface using DKT	1.46E-01	1.79E-02

						parcellation		
481	27310	aparc-DKTatlas_rh_volume_middletemporal	Desikan Atlas	regional and tissue volume	mm3	Volume of middletemporal in the right hemisphere generated by parcellation of the white surface using DKT parcellation	2.16E-01	2.10E-02
482	27311	aparc-DKTatlas_rh_volume_parahippocampal	Desikan Atlas	regional and tissue volume	mm3	Volume of parahippocampal in the right hemisphere generated by parcellation of the white surface using DKT parcellation	1.98E-01	1.88E-02
483	27312	aparc-DKTatlas_rh_volume_paracentral	Desikan Atlas	regional and tissue volume	mm3	Volume of paracentral in the right hemisphere generated by parcellation of the white surface using DKT parcellation	1.33E-01	1.79E-02
484	27313	aparc-DKTatlas_rh_volume_parsopercularis	Desikan Atlas	regional and tissue volume	mm3	Volume of parsopercularis in the right hemisphere generated by parcellation of the white surface using DKT parcellation	1.38E-01	1.74E-02
485	27314	aparc-DKTatlas_rh_volume_parsorbitalis	Desikan Atlas	regional and tissue volume	mm3	Volume of parsorbitalis in the right hemisphere generated by parcellation of the white surface using DKT parcellation	1.45E-01	1.83E-02
486	27315	aparc-DKTatlas_rh_volume_parstriangularis	Desikan Atlas	regional and tissue volume	mm3	Volume of parstriangularis in the right hemisphere generated by parcellation of the white surface using DKT parcellation	1.05E-01	1.91E-02
487	27316	aparc-DKTatlas_rh_volume_pericalcarine	Desikan Atlas	regional and tissue volume	mm3	Volume of pericalcarine in the right hemisphere generated by parcellation of the white surface using DKT parcellation	3.11E-01	2.95E-02
488	27317	aparc-DKTatlas_rh_volume_postcentral	Desikan Atlas	regional and tissue volume	mm3	Volume of postcentral in the right hemisphere generated by parcellation of the white surface using DKT parcellation	1.76E-01	1.89E-02
489	27318	aparc-DKTatlas_rh_volume_posteriorcingulate	Desikan Atlas	regional and tissue volume	mm3	Volume of posteriorcingulate in the right hemisphere generated by parcellation of the white surface using DKT parcellation	1.27E-01	1.67E-02
490	27319	aparc-DKTatlas_rh_volume_precentral	Desikan Atlas	regional and tissue volume	mm3	Volume of precentral in the right hemisphere generated by parcellation of the white surface using DKT parcellation	2.20E-01	2.23E-02
491	27320	aparc-DKTatlas_rh_volume_precuneus	Desikan Atlas	regional and tissue volume	mm3	Volume of precuneus in the right hemisphere generated by parcellation of the white surface using DKT parcellation	2.27E-01	2.16E-02

492	27321	aparc-DKTatlas_rh_volume_rostralanteriorcingulate	Desikan Atlas	regional and tissue volume	mm3	Volume of rostralanteriorcingulate in the right hemisphere generated by parcellation of the white surface using DKT parcellation	1.17E-01	1.48E-02
493	27322	aparc-DKTatlas_rh_volume_rostralmiddlefrontal	Desikan Atlas	regional and tissue volume	mm3	Volume of rostralmiddlefrontal in the right hemisphere generated by parcellation of the white surface using DKT parcellation	1.83E-01	1.74E-02
494	27323	aparc-DKTatlas_rh_volume_superiorfrontal	Desikan Atlas	regional and tissue volume	mm3	Volume of superiorfrontal in the right hemisphere generated by parcellation of the white surface using DKT parcellation	1.83E-01	1.72E-02
495	27324	aparc-DKTatlas_rh_volume_superiorparietal	Desikan Atlas	regional and tissue volume	mm3	Volume of superiorparietal in the right hemisphere generated by parcellation of the white surface using DKT parcellation	1.70E-01	1.95E-02
496	27325	aparc-DKTatlas_rh_volume_superiortemporal	Desikan Atlas	regional and tissue volume	mm3	Volume of superiortemporal in the right hemisphere generated by parcellation of the white surface using DKT parcellation	2.13E-01	1.78E-02
497	27326	aparc-DKTatlas_rh_volume_supramarginal	Desikan Atlas	regional and tissue volume	mm3	Volume of supramarginal in the right hemisphere generated by parcellation of the white surface using DKT parcellation	1.55E-01	1.81E-02
498	27327	aparc-DKTatlas_rh_volume_transversetemporal	Desikan Atlas	regional and tissue volume	mm3	Volume of transversetemporal in the right hemisphere generated by parcellation of the white surface using DKT parcellation	1.53E-01	1.76E-02
499	27328	aparc-DKTatlas_rh_volume_insula	Desikan Atlas	regional and tissue volume	mm3	Volume of insula in the right hemisphere generated by parcellation of the white surface using DKT parcellation	2.80E-01	2.30E-02
500	27477	aparc-a2009s_lh_volume_G+S-frontomargin	Destrieux Atlas	regional and tissue volume	mm3	Volume of G+S-frontomargin in the left hemisphere generated by parcellation of the white surface using Destrieux (a2009s) parcellation	1.29E-01	1.83E-02
501	27478	aparc-a2009s_lh_volume_G+S-occipital-inf	Destrieux Atlas	regional and tissue volume	mm3	Volume of G+S-occipital-inf in the left hemisphere generated by parcellation of the white surface using Destrieux (a2009s) parcellation	1.09E-01	1.64E-02
502	27479	aparc-a2009s_lh_volume_G+S-paracentral	Destrieux Atlas	regional and tissue volume	mm3	Volume of G+S-paracentral in the left hemisphere generated by parcellation of the white surface using Destrieux (a2009s) parcellation	1.34E-01	1.69E-02

503	27480	aparc-a2009s_lh_volume_G+S-subcentral	Destrieux Atlas	regional and tissue volume	mm3	Volume of G+S-subcentral in the left hemisphere generated by parcellation of the white surface using Destrieux (a2009s) parcellation	1.55E-01	1.70E-02
504	27481	aparc-a2009s_lh_volume_G+S-transv-frontopol	Destrieux Atlas	regional and tissue volume	mm3	Volume of G+S-transv-frontopol in the left hemisphere generated by parcellation of the white surface using Destrieux (a2009s) parcellation	8.12E-02	1.62E-02
505	27482	aparc-a2009s_lh_volume_G+S-cingul-Ant	Destrieux Atlas	regional and tissue volume	mm3	Volume of G+S-cingul-Ant in the left hemisphere generated by parcellation of the white surface using Destrieux (a2009s) parcellation	1.54E-01	1.94E-02
506	27483	aparc-a2009s_lh_volume_G+S-cingul-Mid-Ant	Destrieux Atlas	regional and tissue volume	mm3	Volume of G+S-cingul-Mid-Ant in the left hemisphere generated by parcellation of the white surface using Destrieux (a2009s) parcellation	1.12E-01	1.61E-02
507	27484	aparc-a2009s_lh_volume_G+S-cingul-Mid-Post	Destrieux Atlas	regional and tissue volume	mm3	Volume of G+S-cingul-Mid-Post in the left hemisphere generated by parcellation of the white surface using Destrieux (a2009s) parcellation	1.30E-01	1.95E-02
508	27485	aparc-a2009s_lh_volume_G-cingul-Post-dorsal	Destrieux Atlas	regional and tissue volume	mm3	Volume of G-cingul-Post-dorsal in the left hemisphere generated by parcellation of the white surface using Destrieux (a2009s) parcellation	1.52E-01	1.79E-02
509	27486	aparc-a2009s_lh_volume_G-cingul-Post-ventral	Destrieux Atlas	regional and tissue volume	mm3	Volume of G-cingul-Post-ventral in the left hemisphere generated by parcellation of the white surface using Destrieux (a2009s) parcellation	1.66E-01	1.94E-02
510	27487	aparc-a2009s_lh_volume_G-cuneus	Destrieux Atlas	regional and tissue volume	mm3	Volume of G-cuneus in the left hemisphere generated by parcellation of the white surface using Destrieux (a2009s) parcellation	2.57E-01	2.33E-02
511	27488	aparc-a2009s_lh_volume_G-front-inf-Opercular	Destrieux Atlas	regional and tissue volume	mm3	Volume of G-front-inf-Opercular in the left hemisphere generated by parcellation of the white surface using Destrieux (a2009s) parcellation	9.94E-02	1.49E-02
512	27489	aparc-a2009s_lh_volume_G-front-inf-Orbital	Destrieux Atlas	regional and tissue volume	mm3	Volume of G-front-inf-Orbital in the left hemisphere generated by parcellation of the white surface using Destrieux (a2009s) parcellation	4.40E-02	1.35E-02

513	27490	aparc-a2009s_lh_volume_G-front-inf-Triangul	Destrieux Atlas	regional and tissue volume	mm3	Volume of G-front-inf-Triangul in the left hemisphere generated by parcellation of the white surface using Destrieux (a2009s) parcellation	1.13E-01	1.72E-02
514	27491	aparc-a2009s_lh_volume_G-front-middle	Destrieux Atlas	regional and tissue volume	mm3	Volume of G-front-middle in the left hemisphere generated by parcellation of the white surface using Destrieux (a2009s) parcellation	1.79E-01	2.00E-02
515	27492	aparc-a2009s_lh_volume_G-front-sup	Destrieux Atlas	regional and tissue volume	mm3	Volume of G-front-sup in the left hemisphere generated by parcellation of the white surface using Destrieux (a2009s) parcellation	1.92E-01	2.07E-02
516	27493	aparc-a2009s_lh_volume_G-Ins-lg+S-cent-ins	Destrieux Atlas	regional and tissue volume	mm3	Volume of G-Ins-lg+S-cent-ins in the left hemisphere generated by parcellation of the white surface using Destrieux (a2009s) parcellation	1.30E-01	1.94E-02
517	27494	aparc-a2009s_lh_volume_G-insular-short	Destrieux Atlas	regional and tissue volume	mm3	Volume of G-insular-short in the left hemisphere generated by parcellation of the white surface using Destrieux (a2009s) parcellation	2.03E-01	2.01E-02
518	27495	aparc-a2009s_lh_volume_G-occipital-middle	Destrieux Atlas	regional and tissue volume	mm3	Volume of G-occipital-middle in the left hemisphere generated by parcellation of the white surface using Destrieux (a2009s) parcellation	1.43E-01	1.85E-02
519	27496	aparc-a2009s_lh_volume_G-occipital-sup	Destrieux Atlas	regional and tissue volume	mm3	Volume of G-occipital-sup in the left hemisphere generated by parcellation of the white surface using Destrieux (a2009s) parcellation	1.80E-01	1.91E-02
520	27497	aparc-a2009s_lh_volume_G-oc-temp-lat-fusifor	Destrieux Atlas	regional and tissue volume	mm3	Volume of G-oc-temp-lat-fusifor in the left hemisphere generated by parcellation of the white surface using Destrieux (a2009s) parcellation	1.64E-01	1.70E-02
521	27498	aparc-a2009s_lh_volume_G-oc-temp-med-Lingu al	Destrieux Atlas	regional and tissue volume	mm3	Volume of G-oc-temp-med-Lingual in the left hemisphere generated by parcellation of the white surface using Destrieux (a2009s) parcellation	2.10E-01	2.26E-02
522	27499	aparc-a2009s_lh_volume_G-oc-temp-med-Parahi p	Destrieux Atlas	regional and tissue volume	mm3	Volume of G-oc-temp-med-Parahip in the left hemisphere generated by parcellation of the white surface using Destrieux (a2009s) parcellation	1.88E-01	2.05E-02

523	27500	aparc-a2009s_lh_volume_G-orbital	Destrieux Atlas	regional and tissue volume	mm3	Volume of G-orbital in the left hemisphere generated by parcellation of the white surface using Destrieux (a2009s) parcellation	2.11E-01	1.93E-02
524	27501	aparc-a2009s_lh_volume_G-pariet-inf-Angular	Destrieux Atlas	regional and tissue volume	mm3	Volume of G-pariet-inf-Angular in the left hemisphere generated by parcellation of the white surface using Destrieux (a2009s) parcellation	1.59E-01	2.07E-02
525	27502	aparc-a2009s_lh_volume_G-pariet-inf-Supramar	Destrieux Atlas	regional and tissue volume	mm3	Volume of G-pariet-inf-Supramar in the left hemisphere generated by parcellation of the white surface using Destrieux (a2009s) parcellation	1.82E-01	1.63E-02
526	27503	aparc-a2009s_lh_volume_G-parietal-sup	Destrieux Atlas	regional and tissue volume	mm3	Volume of G-parietal-sup in the left hemisphere generated by parcellation of the white surface using Destrieux (a2009s) parcellation	1.70E-01	1.91E-02
527	27504	aparc-a2009s_lh_volume_G-postcentral	Destrieux Atlas	regional and tissue volume	mm3	Volume of G-postcentral in the left hemisphere generated by parcellation of the white surface using Destrieux (a2009s) parcellation	1.51E-01	2.07E-02
528	27505	aparc-a2009s_lh_volume_G-precentral	Destrieux Atlas	regional and tissue volume	mm3	Volume of G-precentral in the left hemisphere generated by parcellation of the white surface using Destrieux (a2009s) parcellation	1.79E-01	1.95E-02
529	27506	aparc-a2009s_lh_volume_G-precuneus	Destrieux Atlas	regional and tissue volume	mm3	Volume of G-precuneus in the left hemisphere generated by parcellation of the white surface using Destrieux (a2009s) parcellation	1.96E-01	2.04E-02
530	27507	aparc-a2009s_lh_volume_G-rectus	Destrieux Atlas	regional and tissue volume	mm3	Volume of G-rectus in the left hemisphere generated by parcellation of the white surface using Destrieux (a2009s) parcellation	9.30E-02	1.44E-02
531	27508	aparc-a2009s_lh_volume_G-subcallosal	Destrieux Atlas	regional and tissue volume	mm3	Volume of G-subcallosal in the left hemisphere generated by parcellation of the white surface using Destrieux (a2009s) parcellation	1.23E-01	1.78E-02
532	27509	aparc-a2009s_lh_volume_G-temp-sup-G-T-transv	Destrieux Atlas	regional and tissue volume	mm3	Volume of G-temp-sup-G-T-transv in the left hemisphere generated by parcellation of the white surface using Destrieux (a2009s) parcellation	1.33E-01	1.54E-02

533	27510	aparc-a2009s_lh_volume_G-temp-sup-Lateral	Destrieux Atlas	regional and tissue volume	mm3	Volume of G-temp-sup-Lateral in the left hemisphere generated by parcellation of the white surface using Destrieux (a2009s) parcellation	1.95E-01	2.24E-02
534	27511	aparc-a2009s_lh_volume_G-temp-sup-Plan-polar	Destrieux Atlas	regional and tissue volume	mm3	Volume of G-temp-sup-Plan-polar in the left hemisphere generated by parcellation of the white surface using Destrieux (a2009s) parcellation	1.38E-01	1.70E-02
535	27512	aparc-a2009s_lh_volume_G-temp-sup-Plan-temp o	Destrieux Atlas	regional and tissue volume	mm3	Volume of G-temp-sup-Plan-tempo in the left hemisphere generated by parcellation of the white surface using Destrieux (a2009s) parcellation	9.90E-02	1.51E-02
536	27513	aparc-a2009s_lh_volume_G-temporal-inf	Destrieux Atlas	regional and tissue volume	mm3	Volume of G-temporal-inf in the left hemisphere generated by parcellation of the white surface using Destrieux (a2009s) parcellation	1.60E-01	1.71E-02
537	27514	aparc-a2009s_lh_volume_G-temporal-middle	Destrieux Atlas	regional and tissue volume	mm3	Volume of G-temporal-middle in the left hemisphere generated by parcellation of the white surface using Destrieux (a2009s) parcellation	1.83E-01	1.95E-02
538	27515	aparc-a2009s_lh_volume_Lat-Fis-ant-Horizont	Destrieux Atlas	regional and tissue volume	mm3	Volume of Lat-Fis-ant-Horizont in the left hemisphere generated by parcellation of the white surface using Destrieux (a2009s) parcellation	4.08E-02	1.57E-02
539	27516	aparc-a2009s_lh_volume_Lat-Fis-ant-Vertical	Destrieux Atlas	regional and tissue volume	mm3	Volume of Lat-Fis-ant-Vertical in the left hemisphere generated by parcellation of the white surface using Destrieux (a2009s) parcellation	4.83E-02	1.45E-02
540	27517	aparc-a2009s_lh_volume_Lat-Fis-post	Destrieux Atlas	regional and tissue volume	mm3	Volume of Lat-Fis-post in the left hemisphere generated by parcellation of the white surface using Destrieux (a2009s) parcellation	1.98E-01	1.86E-02
541	27518	aparc-a2009s_lh_volume_Pole-occipital	Destrieux Atlas	regional and tissue volume	mm3	Volume of Pole-occipital in the left hemisphere generated by parcellation of the white surface using Destrieux (a2009s) parcellation	1.35E-01	1.66E-02
542	27519	aparc-a2009s_lh_volume_Pole-temporal	Destrieux Atlas	regional and tissue volume	mm3	Volume of Pole-temporal in the left hemisphere generated by parcellation of the white surface using Destrieux (a2009s) parcellation	1.14E-01	1.67E-02

543	27520	aparc-a2009s_lh_volume_S-calcarine	Destrieux Atlas	regional and tissue volume	mm3	Volume of S-calcarine in the left hemisphere generated by parcellation of the white surface using Destrieux (a2009s) parcellation	2.90E-01	2.65E-02
544	27521	aparc-a2009s_lh_volume_S-central	Destrieux Atlas	regional and tissue volume	mm3	Volume of S-central in the left hemisphere generated by parcellation of the white surface using Destrieux (a2009s) parcellation	1.68E-01	2.03E-02
545	27522	aparc-a2009s_lh_volume_S-cingul-Marginalis	Destrieux Atlas	regional and tissue volume	mm3	Volume of S-cingul-Marginalis in the left hemisphere generated by parcellation of the white surface using Destrieux (a2009s) parcellation	1.40E-01	1.80E-02
546	27523	aparc-a2009s_lh_volume_S-circular-insula-ant	Destrieux Atlas	regional and tissue volume	mm3	Volume of S-circular-insula-ant in the left hemisphere generated by parcellation of the white surface using Destrieux (a2009s) parcellation	1.31E-01	1.69E-02
547	27524	aparc-a2009s_lh_volume_S-circular-insula-inf	Destrieux Atlas	regional and tissue volume	mm3	Volume of S-circular-insula-inf in the left hemisphere generated by parcellation of the white surface using Destrieux (a2009s) parcellation	1.40E-01	1.62E-02
548	27525	aparc-a2009s_lh_volume_S-circular-insula-sup	Destrieux Atlas	regional and tissue volume	mm3	Volume of S-circular-insula-sup in the left hemisphere generated by parcellation of the white surface using Destrieux (a2009s) parcellation	1.70E-01	2.08E-02
549	27526	aparc-a2009s_lh_volume_S-collat-transv-ant	Destrieux Atlas	regional and tissue volume	mm3	Volume of S-collat-transv-ant in the left hemisphere generated by parcellation of the white surface using Destrieux (a2009s) parcellation	1.43E-01	1.68E-02
550	27527	aparc-a2009s_lh_volume_S-collat-transv-post	Destrieux Atlas	regional and tissue volume	mm3	Volume of S-collat-transv-post in the left hemisphere generated by parcellation of the white surface using Destrieux (a2009s) parcellation	9.51E-02	1.63E-02
551	27528	aparc-a2009s_lh_volume_S-front-inf	Destrieux Atlas	regional and tissue volume	mm3	Volume of S-front-inf in the left hemisphere generated by parcellation of the white surface using Destrieux (a2009s) parcellation	1.12E-01	1.61E-02
552	27529	aparc-a2009s_lh_volume_S-front-middle	Destrieux Atlas	regional and tissue volume	mm3	Volume of S-front-middle in the left hemisphere generated by parcellation of the white surface using Destrieux (a2009s) parcellation	8.71E-02	1.33E-02

553	27530	aparc-a2009s_lh_volume_S-front-sup	Destrieux Atlas	regional and tissue volume	mm3	Volume of S-front-sup in the left hemisphere generated by parcellation of the white surface using Destrieux (a2009s) parcellation	1.19E-01	1.67E-02
554	27531	aparc-a2009s_lh_volume_S-interm-prim-Jensen	Destrieux Atlas	regional and tissue volume	mm3	Volume of S-interm-prim-Jensen in the left hemisphere generated by parcellation of the white surface using Destrieux (a2009s) parcellation	4.24E-02	1.32E-02
555	27532	aparc-a2009s_lh_volume_S-intrapariet+P-trans	Destrieux Atlas	regional and tissue volume	mm3	Volume of S-intrapariet+P-trans in the left hemisphere generated by parcellation of the white surface using Destrieux (a2009s) parcellation	1.21E-01	1.80E-02
556	27533	aparc-a2009s_lh_volume_S-oc-middle+Lunatus	Destrieux Atlas	regional and tissue volume	mm3	Volume of S-oc-middle+Lunatus in the left hemisphere generated by parcellation of the white surface using Destrieux (a2009s) parcellation	1.33E-01	1.79E-02
557	27534	aparc-a2009s_lh_volume_S-oc-sup+transversal	Destrieux Atlas	regional and tissue volume	mm3	Volume of S-oc-sup+transversal in the left hemisphere generated by parcellation of the white surface using Destrieux (a2009s) parcellation	1.05E-01	1.85E-02
558	27535	aparc-a2009s_lh_volume_S-occipital-ant	Destrieux Atlas	regional and tissue volume	mm3	Volume of S-occipital-ant in the left hemisphere generated by parcellation of the white surface using Destrieux (a2009s) parcellation	3.82E-02	1.45E-02
559	27536	aparc-a2009s_lh_volume_S-oc-temp-lat	Destrieux Atlas	regional and tissue volume	mm3	Volume of S-oc-temp-lat in the left hemisphere generated by parcellation of the white surface using Destrieux (a2009s) parcellation	8.86E-02	1.47E-02
560	27537	aparc-a2009s_lh_volume_S-oc-temp-med+Lingual	Destrieux Atlas	regional and tissue volume	mm3	Volume of S-oc-temp-med+Lingual in the left hemisphere generated by parcellation of the white surface using Destrieux (a2009s) parcellation	1.13E-01	1.56E-02
561	27538	aparc-a2009s_lh_volume_S-orbital-lateral	Destrieux Atlas	regional and tissue volume	mm3	Volume of S-orbital-lateral in the left hemisphere generated by parcellation of the white surface using Destrieux (a2009s) parcellation	5.46E-02	1.53E-02
562	27539	aparc-a2009s_lh_volume_S-orbital-med-olfact	Destrieux Atlas	regional and tissue volume	mm3	Volume of S-orbital-med-olfact in the left hemisphere generated by parcellation of the white surface using Destrieux (a2009s) parcellation	1.36E-01	1.99E-02

563	27540	aparc-a2009s_lh_volume_S-orbital-H-Shaped	Destrieux Atlas	regional and tissue volume	mm3	Volume of S-orbital-H-Shaped in the left hemisphere generated by parcellation of the white surface using Destrieux (a2009s) parcellation	1.99E-01	1.95E-02
564	27541	aparc-a2009s_lh_volume_S-parieto-occipital	Destrieux Atlas	regional and tissue volume	mm3	Volume of S-parieto-occipital in the left hemisphere generated by parcellation of the white surface using Destrieux (a2009s) parcellation	2.26E-01	1.92E-02
565	27542	aparc-a2009s_lh_volume_S-pericallosal	Destrieux Atlas	regional and tissue volume	mm3	Volume of S-pericallosal in the left hemisphere generated by parcellation of the white surface using Destrieux (a2009s) parcellation	1.23E-01	1.63E-02
566	27543	aparc-a2009s_lh_volume_S-postcentral	Destrieux Atlas	regional and tissue volume	mm3	Volume of S-postcentral in the left hemisphere generated by parcellation of the white surface using Destrieux (a2009s) parcellation	1.07E-01	1.94E-02
567	27544	aparc-a2009s_lh_volume_S-precentral-inf-part	Destrieux Atlas	regional and tissue volume	mm3	Volume of S-precentral-inf-part in the left hemisphere generated by parcellation of the white surface using Destrieux (a2009s) parcellation	9.87E-02	1.64E-02
568	27545	aparc-a2009s_lh_volume_S-precentral-sup-part	Destrieux Atlas	regional and tissue volume	mm3	Volume of S-precentral-sup-part in the left hemisphere generated by parcellation of the white surface using Destrieux (a2009s) parcellation	1.15E-01	1.68E-02
569	27546	aparc-a2009s_lh_volume_S-suborbital	Destrieux Atlas	regional and tissue volume	mm3	Volume of S-suborbital in the left hemisphere generated by parcellation of the white surface using Destrieux (a2009s) parcellation	4.00E-02	1.45E-02
570	27547	aparc-a2009s_lh_volume_S-subparietal	Destrieux Atlas	regional and tissue volume	mm3	Volume of S-subparietal in the left hemisphere generated by parcellation of the white surface using Destrieux (a2009s) parcellation	1.44E-01	1.80E-02
571	27548	aparc-a2009s_lh_volume_S-temporal-inf	Destrieux Atlas	regional and tissue volume	mm3	Volume of S-temporal-inf in the left hemisphere generated by parcellation of the white surface using Destrieux (a2009s) parcellation	1.10E-01	1.62E-02
572	27549	aparc-a2009s_lh_volume_S-temporal-sup	Destrieux Atlas	regional and tissue volume	mm3	Volume of S-temporal-sup in the left hemisphere generated by parcellation of the white surface using Destrieux (a2009s) parcellation	1.84E-01	1.78E-02

573	27550	aparc-a2009s_lh_volume_S-temporal-transverse	Destrieux Atlas	regional and tissue volume	mm3	Volume of S-temporal-transverse in the left hemisphere generated by parcellation of the white surface using Destrieux (a2009s) parcellation	5.95E-02	1.37E-02
574	27699	aparc-a2009s_rh_volume_G+S-frontomargin	Destrieux Atlas	regional and tissue volume	mm3	Volume of G+S-frontomargin in the right hemisphere generated by parcellation of the white surface using Destrieux (a2009s) parcellation	9.40E-02	1.71E-02
575	27700	aparc-a2009s_rh_volume_G+S-occipital-inf	Destrieux Atlas	regional and tissue volume	mm3	Volume of G+S-occipital-inf in the right hemisphere generated by parcellation of the white surface using Destrieux (a2009s) parcellation	1.16E-01	1.67E-02
576	27701	aparc-a2009s_rh_volume_G+S-paracentral	Destrieux Atlas	regional and tissue volume	mm3	Volume of G+S-paracentral in the right hemisphere generated by parcellation of the white surface using Destrieux (a2009s) parcellation	1.18E-01	1.77E-02
577	27702	aparc-a2009s_rh_volume_G+S-subcentral	Destrieux Atlas	regional and tissue volume	mm3	Volume of G+S-subcentral in the right hemisphere generated by parcellation of the white surface using Destrieux (a2009s) parcellation	1.52E-01	1.83E-02
578	27703	aparc-a2009s_rh_volume_G+S-transv-frontopol	Destrieux Atlas	regional and tissue volume	mm3	Volume of G+S-transv-frontopol in the right hemisphere generated by parcellation of the white surface using Destrieux (a2009s) parcellation	1.06E-01	1.66E-02
579	27704	aparc-a2009s_rh_volume_G+S-cingul-Ant	Destrieux Atlas	regional and tissue volume	mm3	Volume of G+S-cingul-Ant in the right hemisphere generated by parcellation of the white surface using Destrieux (a2009s) parcellation	1.80E-01	1.68E-02
580	27705	aparc-a2009s_rh_volume_G+S-cingul-Mid-Ant	Destrieux Atlas	regional and tissue volume	mm3	Volume of G+S-cingul-Mid-Ant in the right hemisphere generated by parcellation of the white surface using Destrieux (a2009s) parcellation	1.55E-01	1.61E-02
581	27706	aparc-a2009s_rh_volume_G+S-cingul-Mid-Post	Destrieux Atlas	regional and tissue volume	mm3	Volume of G+S-cingul-Mid-Post in the right hemisphere generated by parcellation of the white surface using Destrieux (a2009s) parcellation	1.48E-01	1.55E-02
582	27707	aparc-a2009s_rh_volume_G-cingul-Post-dorsal	Destrieux Atlas	regional and tissue volume	mm3	Volume of G-cingul-Post-dorsal in the right hemisphere generated by parcellation of the white surface using Destrieux (a2009s) parcellation	1.01E-01	1.56E-02

583	27708	aparc-a2009s_rh_volume_G-cingul-Post-ventral	Destrieux Atlas	regional and tissue volume	mm3	Volume of G-cingul-Post-ventral in the right hemisphere generated by parcellation of the white surface using Destrieux (a2009s) parcellation	1.73E-01	2.02E-02
584	27709	aparc-a2009s_rh_volume_G-cuneus	Destrieux Atlas	regional and tissue volume	mm3	Volume of G-cuneus in the right hemisphere generated by parcellation of the white surface using Destrieux (a2009s) parcellation	2.44E-01	2.35E-02
585	27710	aparc-a2009s_rh_volume_G-front-inf-Opercular	Destrieux Atlas	regional and tissue volume	mm3	Volume of G-front-inf-Opercular in the right hemisphere generated by parcellation of the white surface using Destrieux (a2009s) parcellation	1.25E-01	1.71E-02
586	27711	aparc-a2009s_rh_volume_G-front-inf-Orbital	Destrieux Atlas	regional and tissue volume	mm3	Volume of G-front-inf-Orbital in the right hemisphere generated by parcellation of the white surface using Destrieux (a2009s) parcellation	4.23E-02	1.57E-02
587	27712	aparc-a2009s_rh_volume_G-front-inf-Triangul	Destrieux Atlas	regional and tissue volume	mm3	Volume of G-front-inf-Triangul in the right hemisphere generated by parcellation of the white surface using Destrieux (a2009s) parcellation	9.56E-02	1.70E-02
588	27713	aparc-a2009s_rh_volume_G-front-middle	Destrieux Atlas	regional and tissue volume	mm3	Volume of G-front-middle in the right hemisphere generated by parcellation of the white surface using Destrieux (a2009s) parcellation	1.54E-01	1.70E-02
589	27714	aparc-a2009s_rh_volume_G-front-sup	Destrieux Atlas	regional and tissue volume	mm3	Volume of G-front-sup in the right hemisphere generated by parcellation of the white surface using Destrieux (a2009s) parcellation	1.64E-01	1.77E-02
590	27715	aparc-a2009s_rh_volume_G-Ins-lg+S-cent-ins	Destrieux Atlas	regional and tissue volume	mm3	Volume of G-Ins-lg+S-cent-ins in the right hemisphere generated by parcellation of the white surface using Destrieux (a2009s) parcellation	1.56E-01	1.97E-02
591	27716	aparc-a2009s_rh_volume_G-insular-short	Destrieux Atlas	regional and tissue volume	mm3	Volume of G-insular-short in the right hemisphere generated by parcellation of the white surface using Destrieux (a2009s) parcellation	1.66E-01	1.74E-02
592	27717	aparc-a2009s_rh_volume_G-occipital-middle	Destrieux Atlas	regional and tissue volume	mm3	Volume of G-occipital-middle in the right hemisphere generated by parcellation of the white surface using Destrieux (a2009s) parcellation	1.06E-01	1.67E-02

593	27718	aparc-a2009s_rh_volume_G-occipital-sup	Destrieux Atlas	regional and tissue volume	mm3	Volume of G-occipital-sup in the right hemisphere generated by parcellation of the white surface using Destrieux (a2009s) parcellation	1.44E-01	1.66E-02
594	27719	aparc-a2009s_rh_volume_G-oc-temp-lat-fusifor	Destrieux Atlas	regional and tissue volume	mm3	Volume of G-oc-temp-lat-fusifor in the right hemisphere generated by parcellation of the white surface using Destrieux (a2009s) parcellation	1.57E-01	1.80E-02
595	27720	aparc-a2009s_rh_volume_G-oc-temp-med-Lingual	Destrieux Atlas	regional and tissue volume	mm3	Volume of G-oc-temp-med-Lingual in the right hemisphere generated by parcellation of the white surface using Destrieux (a2009s) parcellation	2.11E-01	2.29E-02
596	27721	aparc-a2009s_rh_volume_G-oc-temp-med-Parahip	Destrieux Atlas	regional and tissue volume	mm3	Volume of G-oc-temp-med-Parahip in the right hemisphere generated by parcellation of the white surface using Destrieux (a2009s) parcellation	1.76E-01	1.97E-02
597	27722	aparc-a2009s_rh_volume_G-orbital	Destrieux Atlas	regional and tissue volume	mm3	Volume of G-orbital in the right hemisphere generated by parcellation of the white surface using Destrieux (a2009s) parcellation	1.92E-01	2.01E-02
598	27723	aparc-a2009s_rh_volume_G-pariet-inf-Angular	Destrieux Atlas	regional and tissue volume	mm3	Volume of G-pariet-inf-Angular in the right hemisphere generated by parcellation of the white surface using Destrieux (a2009s) parcellation	1.27E-01	1.64E-02
599	27724	aparc-a2009s_rh_volume_G-pariet-inf-Supramar	Destrieux Atlas	regional and tissue volume	mm3	Volume of G-pariet-inf-Supramar in the right hemisphere generated by parcellation of the white surface using Destrieux (a2009s) parcellation	1.52E-01	1.87E-02
600	27725	aparc-a2009s_rh_volume_G-parietal-sup	Destrieux Atlas	regional and tissue volume	mm3	Volume of G-parietal-sup in the right hemisphere generated by parcellation of the white surface using Destrieux (a2009s) parcellation	1.33E-01	1.78E-02
601	27726	aparc-a2009s_rh_volume_G-postcentral	Destrieux Atlas	regional and tissue volume	mm3	Volume of G-postcentral in the right hemisphere generated by parcellation of the white surface using Destrieux (a2009s) parcellation	1.81E-01	2.03E-02
602	27727	aparc-a2009s_rh_volume_G-precentral	Destrieux Atlas	regional and tissue volume	mm3	Volume of G-precentral in the right hemisphere generated by parcellation of the white surface using Destrieux (a2009s) parcellation	1.99E-01	2.02E-02

603	27728	aparc-a2009s_rh_volume_G-precuneus	Destrieux Atlas	regional and tissue volume	mm3	Volume of G-precuneus in the right hemisphere generated by parcellation of the white surface using Destrieux (a2009s) parcellation	1.92E-01	2.06E-02
604	27729	aparc-a2009s_rh_volume_G-rectus	Destrieux Atlas	regional and tissue volume	mm3	Volume of G-rectus in the right hemisphere generated by parcellation of the white surface using Destrieux (a2009s) parcellation	1.17E-01	1.74E-02
605	27730	aparc-a2009s_rh_volume_G-subcallosal	Destrieux Atlas	regional and tissue volume	mm3	Volume of G-subcallosal in the right hemisphere generated by parcellation of the white surface using Destrieux (a2009s) parcellation	1.26E-01	2.05E-02
606	27731	aparc-a2009s_rh_volume_G-temp-sup-G-T-transv	Destrieux Atlas	regional and tissue volume	mm3	Volume of G-temp-sup-G-T-transv in the right hemisphere generated by parcellation of the white surface using Destrieux (a2009s) parcellation	1.24E-01	1.64E-02
607	27732	aparc-a2009s_rh_volume_G-temp-sup-Lateral	Destrieux Atlas	regional and tissue volume	mm3	Volume of G-temp-sup-Lateral in the right hemisphere generated by parcellation of the white surface using Destrieux (a2009s) parcellation	1.88E-01	1.80E-02
608	27733	aparc-a2009s_rh_volume_G-temp-sup-Plan-polar	Destrieux Atlas	regional and tissue volume	mm3	Volume of G-temp-sup-Plan-polar in the right hemisphere generated by parcellation of the white surface using Destrieux (a2009s) parcellation	1.11E-01	1.59E-02
609	27734	aparc-a2009s_rh_volume_G-temp-sup-Plan-tempo	Destrieux Atlas	regional and tissue volume	mm3	Volume of G-temp-sup-Plan-tempo in the right hemisphere generated by parcellation of the white surface using Destrieux (a2009s) parcellation	1.49E-01	1.62E-02
610	27735	aparc-a2009s_rh_volume_G-temporal-inf	Destrieux Atlas	regional and tissue volume	mm3	Volume of G-temporal-inf in the right hemisphere generated by parcellation of the white surface using Destrieux (a2009s) parcellation	1.58E-01	1.72E-02
611	27736	aparc-a2009s_rh_volume_G-temporal-middle	Destrieux Atlas	regional and tissue volume	mm3	Volume of G-temporal-middle in the right hemisphere generated by parcellation of the white surface using Destrieux (a2009s) parcellation	1.72E-01	1.92E-02
612	27737	aparc-a2009s_rh_volume_Lat-Fis-ant-Horizont	Destrieux Atlas	regional and tissue volume	mm3	Volume of Lat-Fis-ant-Horizont in the right hemisphere generated by parcellation of the white surface using Destrieux (a2009s) parcellation	4.02E-02	1.45E-02

613	27738	aparc-a2009s_rh_volume_Lat-Fis-ant-Vertical	Destrieux Atlas	regional and tissue volume	mm3	Volume of Lat-Fis-ant-Vertical in the right hemisphere generated by parcellation of the white surface using Destrieux (a2009s) parcellation	3.66E-02	1.58E-02
614	27739	aparc-a2009s_rh_volume_Lat-Fis-post	Destrieux Atlas	regional and tissue volume	mm3	Volume of Lat-Fis-post in the right hemisphere generated by parcellation of the white surface using Destrieux (a2009s) parcellation	1.69E-01	1.60E-02
615	27740	aparc-a2009s_rh_volume_Pole-occipital	Destrieux Atlas	regional and tissue volume	mm3	Volume of Pole-occipital in the right hemisphere generated by parcellation of the white surface using Destrieux (a2009s) parcellation	2.00E-01	2.26E-02
616	27741	aparc-a2009s_rh_volume_Pole-temporal	Destrieux Atlas	regional and tissue volume	mm3	Volume of Pole-temporal in the right hemisphere generated by parcellation of the white surface using Destrieux (a2009s) parcellation	1.48E-01	1.73E-02
617	27742	aparc-a2009s_rh_volume_S-calcarine	Destrieux Atlas	regional and tissue volume	mm3	Volume of S-calcarine in the right hemisphere generated by parcellation of the white surface using Destrieux (a2009s) parcellation	3.01E-01	2.82E-02
618	27743	aparc-a2009s_rh_volume_S-central	Destrieux Atlas	regional and tissue volume	mm3	Volume of S-central in the right hemisphere generated by parcellation of the white surface using Destrieux (a2009s) parcellation	1.95E-01	2.63E-02
619	27744	aparc-a2009s_rh_volume_S-cingul-Marginalis	Destrieux Atlas	regional and tissue volume	mm3	Volume of S-cingul-Marginalis in the right hemisphere generated by parcellation of the white surface using Destrieux (a2009s) parcellation	1.12E-01	1.62E-02
620	27745	aparc-a2009s_rh_volume_S-circular-insula-ant	Destrieux Atlas	regional and tissue volume	mm3	Volume of S-circular-insula-ant in the right hemisphere generated by parcellation of the white surface using Destrieux (a2009s) parcellation	1.25E-01	1.70E-02
621	27746	aparc-a2009s_rh_volume_S-circular-insula-inf	Destrieux Atlas	regional and tissue volume	mm3	Volume of S-circular-insula-inf in the right hemisphere generated by parcellation of the white surface using Destrieux (a2009s) parcellation	1.47E-01	1.85E-02
622	27747	aparc-a2009s_rh_volume_S-circular-insula-sup	Destrieux Atlas	regional and tissue volume	mm3	Volume of S-circular-insula-sup in the right hemisphere generated by parcellation of the white surface using Destrieux (a2009s) parcellation	1.60E-01	1.86E-02

623	27748	aparc-a2009s_rh_volume_S-collat-transv-ant	Destrieux Atlas	regional and tissue volume	mm3	Volume of S-collat-transv-ant in the right hemisphere generated by parcellation of the white surface using Destrieux (a2009s) parcellation	1.70E-01	1.80E-02
624	27749	aparc-a2009s_rh_volume_S-collat-transv-post	Destrieux Atlas	regional and tissue volume	mm3	Volume of S-collat-transv-post in the right hemisphere generated by parcellation of the white surface using Destrieux (a2009s) parcellation	1.03E-01	1.59E-02
625	27750	aparc-a2009s_rh_volume_S-front-inf	Destrieux Atlas	regional and tissue volume	mm3	Volume of S-front-inf in the right hemisphere generated by parcellation of the white surface using Destrieux (a2009s) parcellation	1.05E-01	1.65E-02
626	27751	aparc-a2009s_rh_volume_S-front-middle	Destrieux Atlas	regional and tissue volume	mm3	Volume of S-front-middle in the right hemisphere generated by parcellation of the white surface using Destrieux (a2009s) parcellation	6.74E-02	1.59E-02
627	27752	aparc-a2009s_rh_volume_S-front-sup	Destrieux Atlas	regional and tissue volume	mm3	Volume of S-front-sup in the right hemisphere generated by parcellation of the white surface using Destrieux (a2009s) parcellation	9.89E-02	1.62E-02
628	27753	aparc-a2009s_rh_volume_S-interm-prim-Jensen	Destrieux Atlas	regional and tissue volume	mm3	Volume of S-interm-prim-Jensen in the right hemisphere generated by parcellation of the white surface using Destrieux (a2009s) parcellation	4.67E-02	1.26E-02
629	27754	aparc-a2009s_rh_volume_S-intrapariet+P-trans	Destrieux Atlas	regional and tissue volume	mm3	Volume of S-intrapariet+P-trans in the right hemisphere generated by parcellation of the white surface using Destrieux (a2009s) parcellation	1.22E-01	1.65E-02
630	27755	aparc-a2009s_rh_volume_S-oc-middle+Lunatus	Destrieux Atlas	regional and tissue volume	mm3	Volume of S-oc-middle+Lunatus in the right hemisphere generated by parcellation of the white surface using Destrieux (a2009s) parcellation	1.19E-01	1.77E-02
631	27756	aparc-a2009s_rh_volume_S-oc-sup+transversal	Destrieux Atlas	regional and tissue volume	mm3	Volume of S-oc-sup+transversal in the right hemisphere generated by parcellation of the white surface using Destrieux (a2009s) parcellation	7.94E-02	1.51E-02
632	27757	aparc-a2009s_rh_volume_S-occipital-ant	Destrieux Atlas	regional and tissue volume	mm3	Volume of S-occipital-ant in the right hemisphere generated by parcellation of the white surface using Destrieux (a2009s) parcellation	8.44E-02	1.54E-02

633	27758	aparc-a2009s_rh_volume_S-oc-temp-lat	Destrieux Atlas	regional and tissue volume	mm3	Volume of S-oc-temp-lat in the right hemisphere generated by parcellation of the white surface using Destrieux (a2009s) parcellation	8.35E-02	1.80E-02
634	27759	aparc-a2009s_rh_volume_S-oc-temp-med+Lingual	Destrieux Atlas	regional and tissue volume	mm3	Volume of S-oc-temp-med+Lingual in the right hemisphere generated by parcellation of the white surface using Destrieux (a2009s) parcellation	1.52E-01	1.76E-02
635	27760	aparc-a2009s_rh_volume_S-orbital-lateral	Destrieux Atlas	regional and tissue volume	mm3	Volume of S-orbital-lateral in the right hemisphere generated by parcellation of the white surface using Destrieux (a2009s) parcellation	6.80E-02	1.42E-02
636	27761	aparc-a2009s_rh_volume_S-orbital-med-olfact	Destrieux Atlas	regional and tissue volume	mm3	Volume of S-orbital-med-olfact in the right hemisphere generated by parcellation of the white surface using Destrieux (a2009s) parcellation	1.16E-01	1.73E-02
637	27762	aparc-a2009s_rh_volume_S-orbital-H-Shaped	Destrieux Atlas	regional and tissue volume	mm3	Volume of S-orbital-H-Shaped in the right hemisphere generated by parcellation of the white surface using Destrieux (a2009s) parcellation	2.18E-01	2.12E-02
638	27763	aparc-a2009s_rh_volume_S-parieto-occipital	Destrieux Atlas	regional and tissue volume	mm3	Volume of S-parieto-occipital in the right hemisphere generated by parcellation of the white surface using Destrieux (a2009s) parcellation	2.39E-01	1.93E-02
639	27764	aparc-a2009s_rh_volume_S-pericallosal	Destrieux Atlas	regional and tissue volume	mm3	Volume of S-pericallosal in the right hemisphere generated by parcellation of the white surface using Destrieux (a2009s) parcellation	1.07E-01	1.42E-02
640	27765	aparc-a2009s_rh_volume_S-postcentral	Destrieux Atlas	regional and tissue volume	mm3	Volume of S-postcentral in the right hemisphere generated by parcellation of the white surface using Destrieux (a2009s) parcellation	1.18E-01	1.65E-02
641	27766	aparc-a2009s_rh_volume_S-precentral-inf-part	Destrieux Atlas	regional and tissue volume	mm3	Volume of S-precentral-inf-part in the right hemisphere generated by parcellation of the white surface using Destrieux (a2009s) parcellation	1.12E-01	1.64E-02
642	27767	aparc-a2009s_rh_volume_S-precentral-sup-part	Destrieux Atlas	regional and tissue volume	mm3	Volume of S-precentral-sup-part in the right hemisphere generated by parcellation of the white surface using Destrieux (a2009s) parcellation	1.04E-01	1.65E-02

643	27768	aparc-a2009s_rh_volume_S-suborbital	Destrieux Atlas	regional and tissue volume	mm3	Volume of S-suborbital in the right hemisphere generated by parcellation of the white surface using Destrieux (a2009s) parcellation	3.48E-02	1.39E-02
644	27769	aparc-a2009s_rh_volume_S-subparietal	Destrieux Atlas	regional and tissue volume	mm3	Volume of S-subparietal in the right hemisphere generated by parcellation of the white surface using Destrieux (a2009s) parcellation	1.18E-01	1.68E-02
645	27770	aparc-a2009s_rh_volume_S-temporal-inf	Destrieux Atlas	regional and tissue volume	mm3	Volume of S-temporal-inf in the right hemisphere generated by parcellation of the white surface using Destrieux (a2009s) parcellation	1.14E-01	1.74E-02
646	27771	aparc-a2009s_rh_volume_S-temporal-sup	Destrieux Atlas	regional and tissue volume	mm3	Volume of S-temporal-sup in the right hemisphere generated by parcellation of the white surface using Destrieux (a2009s) parcellation	1.82E-01	1.85E-02
647	27772	aparc-a2009s_rh_volume_S-temporal-transverse	Destrieux Atlas	regional and tissue volume	mm3	Volume of S-temporal-transverse in the right hemisphere generated by parcellation of the white surface using Destrieux (a2009s) parcellation	1.18E-01	1.40E-02
648	26721	aparc-Desikan_lh_area_TotalSurface	Desikan Atlas	cortical area	mm2	Area of TotalSurface in the left hemisphere generated by parcellation of the white surface using Desikan-Killiany parcellation	3.14E-01	2.36E-02
649	26722	aparc-Desikan_lh_area_bankssts	Desikan Atlas	cortical area	mm2	Area of bankssts in the left hemisphere generated by parcellation of the white surface using Desikan-Killiany parcellation	1.62E-01	1.75E-02
650	26723	aparc-Desikan_lh_area_caudalanteriorcingulate	Desikan Atlas	cortical area	mm2	Area of caudalanteriorcingulate in the left hemisphere generated by parcellation of the white surface using Desikan-Killiany parcellation	1.14E-01	1.63E-02
651	26724	aparc-Desikan_lh_area_caudalmiddlefrontal	Desikan Atlas	cortical area	mm2	Area of caudalmiddlefrontal in the left hemisphere generated by parcellation of the white surface using Desikan-Killiany parcellation	1.81E-01	2.33E-02
652	26725	aparc-Desikan_lh_area_cuneus	Desikan Atlas	cortical area	mm2	Area of cuneus in the left hemisphere generated by parcellation of the white surface using Desikan-Killiany parcellation	2.72E-01	2.36E-02
653	26726	aparc-Desikan_lh_area_entorhinal	Desikan Atlas	cortical area	mm2	Area of entorhinal in the left hemisphere generated by parcellation of the white surface using Desikan-Killiany parcellation	1.76E-01	1.92E-02

654	26727	aparc-Desikan_lh_area_fusiform	Desikan Atlas	cortical area	mm2	Area of fusiform in the left hemisphere generated by parcellation of the white surface using Desikan-Killiany parcellation	1.86E-01	1.90E-02
655	26728	aparc-Desikan_lh_area_inferiorparietal	Desikan Atlas	cortical area	mm2	Area of inferiorparietal in the left hemisphere generated by parcellation of the white surface using Desikan-Killiany parcellation	2.05E-01	1.77E-02
656	26729	aparc-Desikan_lh_area_inferiortemporal	Desikan Atlas	cortical area	mm2	Area of inferiortemporal in the left hemisphere generated by parcellation of the white surface using Desikan-Killiany parcellation	2.00E-01	1.79E-02
657	26730	aparc-Desikan_lh_area_isthmuscingulate	Desikan Atlas	cortical area	mm2	Area of isthmuscingulate in the left hemisphere generated by parcellation of the white surface using Desikan-Killiany parcellation	1.99E-01	2.10E-02
658	26731	aparc-Desikan_lh_area_lateraloccipital	Desikan Atlas	cortical area	mm2	Area of lateraloccipital in the left hemisphere generated by parcellation of the white surface using Desikan-Killiany parcellation	2.14E-01	2.14E-02
659	26732	aparc-Desikan_lh_area_lateralorbitofrontal	Desikan Atlas	cortical area	mm2	Area of lateralorbitofrontal in the left hemisphere generated by parcellation of the white surface using Desikan-Killiany parcellation	2.69E-01	2.21E-02
660	26733	aparc-Desikan_lh_area_lingual	Desikan Atlas	cortical area	mm2	Area of lingual in the left hemisphere generated by parcellation of the white surface using Desikan-Killiany parcellation	2.56E-01	2.30E-02
661	26734	aparc-Desikan_lh_area_medialorbitofrontal	Desikan Atlas	cortical area	mm2	Area of medialorbitofrontal in the left hemisphere generated by parcellation of the white surface using Desikan-Killiany parcellation	1.16E-01	1.84E-02
662	26735	aparc-Desikan_lh_area_middletemporal	Desikan Atlas	cortical area	mm2	Area of middletemporal in the left hemisphere generated by parcellation of the white surface using Desikan-Killiany parcellation	2.29E-01	2.01E-02
663	26736	aparc-Desikan_lh_area_parahippocampal	Desikan Atlas	cortical area	mm2	Area of parahippocampal in the left hemisphere generated by parcellation of the white surface using Desikan-Killiany parcellation	1.64E-01	1.93E-02
664	26737	aparc-Desikan_lh_area_paracentral	Desikan Atlas	cortical area	mm2	Area of paracentral in the left hemisphere generated by parcellation of the white surface using Desikan-Killiany parcellation	2.16E-01	2.07E-02

665	26738	aparc-Desikan_lh_area_parsopercularis	Desikan Atlas	cortical area	mm2	Area of parsopercularis in the left hemisphere generated by parcellation of the white surface using Desikan-Killiany parcellation	1.43E-01	1.64E-02
666	26739	aparc-Desikan_lh_area_parsorbitalis	Desikan Atlas	cortical area	mm2	Area of parsorbitalis in the left hemisphere generated by parcellation of the white surface using Desikan-Killiany parcellation	1.66E-01	1.96E-02
667	26740	aparc-Desikan_lh_area_parstriangularis	Desikan Atlas	cortical area	mm2	Area of parstriangularis in the left hemisphere generated by parcellation of the white surface using Desikan-Killiany parcellation	1.78E-01	1.97E-02
668	26741	aparc-Desikan_lh_area_pericalcarine	Desikan Atlas	cortical area	mm2	Area of pericalcarine in the left hemisphere generated by parcellation of the white surface using Desikan-Killiany parcellation	3.24E-01	2.89E-02
669	26742	aparc-Desikan_lh_area_postcentral	Desikan Atlas	cortical area	mm2	Area of postcentral in the left hemisphere generated by parcellation of the white surface using Desikan-Killiany parcellation	1.79E-01	1.96E-02
670	26743	aparc-Desikan_lh_area_posteriorcingulate	Desikan Atlas	cortical area	mm2	Area of posteriorcingulate in the left hemisphere generated by parcellation of the white surface using Desikan-Killiany parcellation	1.67E-01	3.50E-02
671	26744	aparc-Desikan_lh_area_precentral	Desikan Atlas	cortical area	mm2	Area of precentral in the left hemisphere generated by parcellation of the white surface using Desikan-Killiany parcellation	2.35E-01	2.64E-02
672	26745	aparc-Desikan_lh_area_precuneus	Desikan Atlas	cortical area	mm2	Area of precuneus in the left hemisphere generated by parcellation of the white surface using Desikan-Killiany parcellation	2.61E-01	2.31E-02
673	26746	aparc-Desikan_lh_area_rostralanteriorcingulate	Desikan Atlas	cortical area	mm2	Area of rostralanteriorcingulate in the left hemisphere generated by parcellation of the white surface using Desikan-Killiany parcellation	1.77E-01	1.84E-02
674	26747	aparc-Desikan_lh_area_rostralmiddlefrontal	Desikan Atlas	cortical area	mm2	Area of rostralmiddlefrontal in the left hemisphere generated by parcellation of the white surface using Desikan-Killiany parcellation	2.51E-01	1.92E-02
675	26748	aparc-Desikan_lh_area_superiorfrontal	Desikan Atlas	cortical area	mm2	Area of superiorfrontal in the left hemisphere generated by parcellation of the white surface using Desikan-Killiany parcellation	2.43E-01	2.58E-02

676	26749	aparc-Desikan_lh_area_superiorparietal	Desikan Atlas	cortical area	mm2	Area of superiorparietal in the left hemisphere generated by parcellation of the white surface using Desikan-Killiany parcellation	2.11E-01	2.00E-02
677	26750	aparc-Desikan_lh_area_superiortemporal	Desikan Atlas	cortical area	mm2	Area of superiortemporal in the left hemisphere generated by parcellation of the white surface using Desikan-Killiany parcellation	2.73E-01	3.79E-02
678	26751	aparc-Desikan_lh_area_supramarginal	Desikan Atlas	cortical area	mm2	Area of supramarginal in the left hemisphere generated by parcellation of the white surface using Desikan-Killiany parcellation	1.82E-01	1.81E-02
679	26752	aparc-Desikan_lh_area_frontalpole	Desikan Atlas	cortical area	mm2	Area of frontalpole in the left hemisphere generated by parcellation of the white surface using Desikan-Killiany parcellation	6.15E-02	1.68E-02
680	26753	aparc-Desikan_lh_area_transversetemporal	Desikan Atlas	cortical area	mm2	Area of transversetemporal in the left hemisphere generated by parcellation of the white surface using Desikan-Killiany parcellation	1.97E-01	1.86E-02
681	26754	aparc-Desikan_lh_area_insula	Desikan Atlas	cortical area	mm2	Area of insula in the left hemisphere generated by parcellation of the white surface using Desikan-Killiany parcellation	2.08E-01	1.86E-02
682	26822	aparc-Desikan_rh_area_TotalSurface	Desikan Atlas	cortical area	mm2	Area of TotalSurface in the right hemisphere generated by parcellation of the white surface using Desikan-Killiany parcellation	3.16E-01	2.44E-02
683	26823	aparc-Desikan_rh_area_bankssts	Desikan Atlas	cortical area	mm2	Area of bankssts in the right hemisphere generated by parcellation of the white surface using Desikan-Killiany parcellation	1.64E-01	1.94E-02
684	26824	aparc-Desikan_rh_area_caudalanteriorcingulate	Desikan Atlas	cortical area	mm2	Area of caudalanteriorcingulate in the right hemisphere generated by parcellation of the white surface using Desikan-Killiany parcellation	1.18E-01	1.68E-02
685	26825	aparc-Desikan_rh_area_caudalmiddlefrontal	Desikan Atlas	cortical area	mm2	Area of caudalmiddlefrontal in the right hemisphere generated by parcellation of the white surface using Desikan-Killiany parcellation	2.04E-01	2.35E-02
686	26826	aparc-Desikan_rh_area_cuneus	Desikan Atlas	cortical area	mm2	Area of cuneus in the right hemisphere generated by parcellation of the white surface using Desikan-Killiany parcellation	2.73E-01	2.20E-02

687	26827	aparc-Desikan_rh_area_entorhinal	Desikan Atlas	cortical area	mm2	Area of entorhinal in the right hemisphere generated by parcellation of the white surface using Desikan-Killiany parcellation	1.69E-01	2.09E-02
688	26828	aparc-Desikan_rh_area_fusiform	Desikan Atlas	cortical area	mm2	Area of fusiform in the right hemisphere generated by parcellation of the white surface using Desikan-Killiany parcellation	1.91E-01	2.06E-02
689	26829	aparc-Desikan_rh_area_inferiorparietal	Desikan Atlas	cortical area	mm2	Area of inferiorparietal in the right hemisphere generated by parcellation of the white surface using Desikan-Killiany parcellation	1.89E-01	1.90E-02
690	26830	aparc-Desikan_rh_area_inferiortemporal	Desikan Atlas	cortical area	mm2	Area of inferiortemporal in the right hemisphere generated by parcellation of the white surface using Desikan-Killiany parcellation	2.30E-01	1.81E-02
691	26831	aparc-Desikan_rh_area_isthmuscingulate	Desikan Atlas	cortical area	mm2	Area of isthmuscingulate in the right hemisphere generated by parcellation of the white surface using Desikan-Killiany parcellation	1.87E-01	2.03E-02
692	26832	aparc-Desikan_rh_area_lateraloccipital	Desikan Atlas	cortical area	mm2	Area of lateraloccipital in the right hemisphere generated by parcellation of the white surface using Desikan-Killiany parcellation	2.38E-01	2.26E-02
693	26833	aparc-Desikan_rh_area_lateralorbitofrontal	Desikan Atlas	cortical area	mm2	Area of lateralorbitofrontal in the right hemisphere generated by parcellation of the white surface using Desikan-Killiany parcellation	1.78E-01	1.99E-02
694	26834	aparc-Desikan_rh_area_lingual	Desikan Atlas	cortical area	mm2	Area of lingual in the right hemisphere generated by parcellation of the white surface using Desikan-Killiany parcellation	2.57E-01	2.44E-02
695	26835	aparc-Desikan_rh_area_medialorbitofrontal	Desikan Atlas	cortical area	mm2	Area of medialorbitofrontal in the right hemisphere generated by parcellation of the white surface using Desikan-Killiany parcellation	1.86E-01	1.86E-02
696	26836	aparc-Desikan_rh_area_middletemporal	Desikan Atlas	cortical area	mm2	Area of middletemporal in the right hemisphere generated by parcellation of the white surface using Desikan-Killiany parcellation	2.47E-01	2.24E-02
697	26837	aparc-Desikan_rh_area_parahippocampal	Desikan Atlas	cortical area	mm2	Area of parahippocampal in the right hemisphere generated by parcellation of the white surface using Desikan-Killiany parcellation	1.67E-01	1.82E-02

698	26838	aparc-Desikan_rh_area_paracentral	Desikan Atlas	cortical area	mm2	Area of paracentral in the right hemisphere generated by parcellation of the white surface using Desikan-Killiany parcellation	2.04E-01	2.55E-02
699	26839	aparc-Desikan_rh_area_parsopercularis	Desikan Atlas	cortical area	mm2	Area of parsopercularis in the right hemisphere generated by parcellation of the white surface using Desikan-Killiany parcellation	1.40E-01	1.81E-02
700	26840	aparc-Desikan_rh_area_parsorbitalis	Desikan Atlas	cortical area	mm2	Area of parsorbitalis in the right hemisphere generated by parcellation of the white surface using Desikan-Killiany parcellation	1.85E-01	1.71E-02
701	26841	aparc-Desikan_rh_area_parstriangularis	Desikan Atlas	cortical area	mm2	Area of parstriangularis in the right hemisphere generated by parcellation of the white surface using Desikan-Killiany parcellation	1.52E-01	2.00E-02
702	26842	aparc-Desikan_rh_area_pericalcarine	Desikan Atlas	cortical area	mm2	Area of pericalcarine in the right hemisphere generated by parcellation of the white surface using Desikan-Killiany parcellation	3.42E-01	3.01E-02
703	26843	aparc-Desikan_rh_area_postcentral	Desikan Atlas	cortical area	mm2	Area of postcentral in the right hemisphere generated by parcellation of the white surface using Desikan-Killiany parcellation	1.80E-01	2.00E-02
704	26844	aparc-Desikan_rh_area_posteriorcingulate	Desikan Atlas	cortical area	mm2	Area of posteriorcingulate in the right hemisphere generated by parcellation of the white surface using Desikan-Killiany parcellation	1.79E-01	2.40E-02
705	26845	aparc-Desikan_rh_area_precentral	Desikan Atlas	cortical area	mm2	Area of precentral in the right hemisphere generated by parcellation of the white surface using Desikan-Killiany parcellation	2.39E-01	2.74E-02
706	26846	aparc-Desikan_rh_area_precuneus	Desikan Atlas	cortical area	mm2	Area of precuneus in the right hemisphere generated by parcellation of the white surface using Desikan-Killiany parcellation	2.33E-01	2.07E-02
707	26847	aparc-Desikan_rh_area_rostralanteriorcingulate	Desikan Atlas	cortical area	mm2	Area of rostralanteriorcingulate in the right hemisphere generated by parcellation of the white surface using Desikan-Killiany parcellation	1.19E-01	1.58E-02
708	26848	aparc-Desikan_rh_area_rostralmiddlefrontal	Desikan Atlas	cortical area	mm2	Area of rostralmiddlefrontal in the right hemisphere generated by parcellation of the white surface using Desikan-Killiany parcellation	2.31E-01	1.85E-02

709	26849	aparc-Desikan_rh_area_superiorfrontal	Desikan Atlas	cortical area	mm2	Area of superiorfrontal in the right hemisphere generated by parcellation of the white surface using Desikan-Killiany parcellation	2.16E-01	2.04E-02
710	26850	aparc-Desikan_rh_area_superiorparietal	Desikan Atlas	cortical area	mm2	Area of superiorparietal in the right hemisphere generated by parcellation of the white surface using Desikan-Killiany parcellation	1.87E-01	1.81E-02
711	26851	aparc-Desikan_rh_area_superiortemporal	Desikan Atlas	cortical area	mm2	Area of superiortemporal in the right hemisphere generated by parcellation of the white surface using Desikan-Killiany parcellation	2.58E-01	3.13E-02
712	26852	aparc-Desikan_rh_area_supramarginal	Desikan Atlas	cortical area	mm2	Area of supramarginal in the right hemisphere generated by parcellation of the white surface using Desikan-Killiany parcellation	1.65E-01	1.90E-02
713	26853	aparc-Desikan_rh_area_frontalpole	Desikan Atlas	cortical area	mm2	Area of frontalpole in the right hemisphere generated by parcellation of the white surface using Desikan-Killiany parcellation	7.48E-02	1.52E-02
714	26854	aparc-Desikan_rh_area_transversetemporal	Desikan Atlas	cortical area	mm2	Area of transversetemporal in the right hemisphere generated by parcellation of the white surface using Desikan-Killiany parcellation	2.12E-01	1.95E-02
715	26855	aparc-Desikan_rh_area_insula	Desikan Atlas	cortical area	mm2	Area of insula in the right hemisphere generated by parcellation of the white surface using Desikan-Killiany parcellation	2.12E-01	2.23E-02
716	26923	aparc-pial_lh_area_TotalSurface	Desikan Atlas	cortical area	mm2	Area of TotalSurface in the left hemisphere generated by parcellation of the pial surface using Desikan-Killiany parcellation	2.93E-01	2.09E-02
717	26924	aparc-pial_lh_area_bankssts	Desikan Atlas	cortical area	mm2	Area of bankssts in the left hemisphere generated by parcellation of the pial surface using Desikan-Killiany parcellation	1.00E-01	1.61E-02
718	26925	aparc-pial_lh_area_caudalanteriorcingulate	Desikan Atlas	cortical area	mm2	Area of caudalanteriorcingulate in the left hemisphere generated by parcellation of the pial surface using Desikan-Killiany parcellation	9.60E-02	1.48E-02
719	26926	aparc-pial_lh_area_caudalmiddlefrontal	Desikan Atlas	cortical area	mm2	Area of caudalmiddlefrontal in the left hemisphere generated by parcellation of the pial surface using Desikan-Killiany parcellation	1.51E-01	2.33E-02

720	26927	aparc-pial_lh_area_cuneus	Desikan Atlas	cortical area	mm2	Area of cuneus in the left hemisphere generated by parcellation of the pial surface using Desikan-Killiany parcellation	2.67E-01	2.43E-02
721	26928	aparc-pial_lh_area_entorhinal	Desikan Atlas	cortical area	mm2	Area of entorhinal in the left hemisphere generated by parcellation of the pial surface using Desikan-Killiany parcellation	1.27E-01	1.59E-02
722	26929	aparc-pial_lh_area_fusiform	Desikan Atlas	cortical area	mm2	Area of fusiform in the left hemisphere generated by parcellation of the pial surface using Desikan-Killiany parcellation	1.64E-01	1.87E-02
723	26930	aparc-pial_lh_area_inferiorparietal	Desikan Atlas	cortical area	mm2	Area of inferiorparietal in the left hemisphere generated by parcellation of the pial surface using Desikan-Killiany parcellation	1.89E-01	1.78E-02
724	26931	aparc-pial_lh_area_inferiortemporal	Desikan Atlas	cortical area	mm2	Area of inferiortemporal in the left hemisphere generated by parcellation of the pial surface using Desikan-Killiany parcellation	1.73E-01	1.73E-02
725	26932	aparc-pial_lh_area_isthmuscingulate	Desikan Atlas	cortical area	mm2	Area of isthmuscingulate in the left hemisphere generated by parcellation of the pial surface using Desikan-Killiany parcellation	1.88E-01	2.05E-02
726	26933	aparc-pial_lh_area_lateraloccipital	Desikan Atlas	cortical area	mm2	Area of lateraloccipital in the left hemisphere generated by parcellation of the pial surface using Desikan-Killiany parcellation	1.93E-01	2.00E-02
727	26934	aparc-pial_lh_area_lateralorbitofrontal	Desikan Atlas	cortical area	mm2	Area of lateralorbitofrontal in the left hemisphere generated by parcellation of the pial surface using Desikan-Killiany parcellation	2.56E-01	2.33E-02
728	26935	aparc-pial_lh_area_lingual	Desikan Atlas	cortical area	mm2	Area of lingual in the left hemisphere generated by parcellation of the pial surface using Desikan-Killiany parcellation	2.47E-01	2.25E-02
729	26936	aparc-pial_lh_area_medialorbitofrontal	Desikan Atlas	cortical area	mm2	Area of medialorbitofrontal in the left hemisphere generated by parcellation of the pial surface using Desikan-Killiany parcellation	9.97E-02	1.77E-02
730	26937	aparc-pial_lh_area_middletemporal	Desikan Atlas	cortical area	mm2	Area of middletemporal in the left hemisphere generated by parcellation of the pial surface using Desikan-Killiany parcellation	2.19E-01	1.94E-02

731	26938	aparc-pial_lh_area_parahippocampal	Desikan Atlas	cortical area	mm2	Area of parahippocampal in the left hemisphere generated by parcellation of the pial surface using Desikan-Killiany parcellation	1.68E-01	1.90E-02
732	26939	aparc-pial_lh_area_paracentral	Desikan Atlas	cortical area	mm2	Area of paracentral in the left hemisphere generated by parcellation of the pial surface using Desikan-Killiany parcellation	1.73E-01	2.08E-02
733	26940	aparc-pial_lh_area_parsopercularis	Desikan Atlas	cortical area	mm2	Area of parsopercularis in the left hemisphere generated by parcellation of the pial surface using Desikan-Killiany parcellation	1.12E-01	1.53E-02
734	26941	aparc-pial_lh_area_parsorbitalis	Desikan Atlas	cortical area	mm2	Area of parsorbitalis in the left hemisphere generated by parcellation of the pial surface using Desikan-Killiany parcellation	1.39E-01	1.77E-02
735	26942	aparc-pial_lh_area_parstriangularis	Desikan Atlas	cortical area	mm2	Area of parstriangularis in the left hemisphere generated by parcellation of the pial surface using Desikan-Killiany parcellation	1.31E-01	1.87E-02
736	26943	aparc-pial_lh_area_pericalcarine	Desikan Atlas	cortical area	mm2	Area of pericalcarine in the left hemisphere generated by parcellation of the pial surface using Desikan-Killiany parcellation	2.97E-01	2.80E-02
737	26944	aparc-pial_lh_area_postcentral	Desikan Atlas	cortical area	mm2	Area of postcentral in the left hemisphere generated by parcellation of the pial surface using Desikan-Killiany parcellation	1.62E-01	1.97E-02
738	26945	aparc-pial_lh_area_posteriorcingulate	Desikan Atlas	cortical area	mm2	Area of posteriorcingulate in the left hemisphere generated by parcellation of the pial surface using Desikan-Killiany parcellation	1.44E-01	3.02E-02
739	26946	aparc-pial_lh_area_precentral	Desikan Atlas	cortical area	mm2	Area of precentral in the left hemisphere generated by parcellation of the pial surface using Desikan-Killiany parcellation	2.30E-01	2.55E-02
740	26947	aparc-pial_lh_area_precuneus	Desikan Atlas	cortical area	mm2	Area of precuneus in the left hemisphere generated by parcellation of the pial surface using Desikan-Killiany parcellation	2.52E-01	2.10E-02
741	26948	aparc-pial_lh_area_rostralanteriorcingulate	Desikan Atlas	cortical area	mm2	Area of rostralanteriorcingulate in the left hemisphere generated by parcellation of the pial surface using Desikan-Killiany parcellation	1.76E-01	1.77E-02

742	26949	aparc-pial_lh_area_rostralmiddlefrontal	Desikan Atlas	cortical area	mm2	Area of rostralmiddlefrontal in the left hemisphere generated by parcellation of the pial surface using Desikan-Killiany parcellation	2.42E-01	1.91E-02
743	26950	aparc-pial_lh_area_superiorfrontal	Desikan Atlas	cortical area	mm2	Area of superiorfrontal in the left hemisphere generated by parcellation of the pial surface using Desikan-Killiany parcellation	2.24E-01	2.50E-02
744	26951	aparc-pial_lh_area_superiorparietal	Desikan Atlas	cortical area	mm2	Area of superiorparietal in the left hemisphere generated by parcellation of the pial surface using Desikan-Killiany parcellation	1.99E-01	1.98E-02
745	26952	aparc-pial_lh_area_superiortemporal	Desikan Atlas	cortical area	mm2	Area of superiortemporal in the left hemisphere generated by parcellation of the pial surface using Desikan-Killiany parcellation	2.44E-01	3.71E-02
746	26953	aparc-pial_lh_area_supramarginal	Desikan Atlas	cortical area	mm2	Area of supramarginal in the left hemisphere generated by parcellation of the pial surface using Desikan-Killiany parcellation	1.71E-01	1.80E-02
747	26954	aparc-pial_lh_area_frontalpole	Desikan Atlas	cortical area	mm2	Area of frontalpole in the left hemisphere generated by parcellation of the pial surface using Desikan-Killiany parcellation	6.62E-02	1.59E-02
748	26955	aparc-pial_lh_area_transversetemporal	Desikan Atlas	cortical area	mm2	Area of transversetemporal in the left hemisphere generated by parcellation of the pial surface using Desikan-Killiany parcellation	1.80E-01	1.84E-02
749	26956	aparc-pial_rh_area_TotalSurface	Desikan Atlas	cortical area	mm2	Area of TotalSurface in the right hemisphere generated by parcellation of the pial surface using Desikan-Killiany parcellation	2.87E-01	2.17E-02
750	26957	aparc-pial_rh_area_bankssts	Desikan Atlas	cortical area	mm2	Area of bankssts in the right hemisphere generated by parcellation of the pial surface using Desikan-Killiany parcellation	8.62E-02	1.54E-02
751	26958	aparc-pial_rh_area_caudalanteriorcingulate	Desikan Atlas	cortical area	mm2	Area of caudalanteriorcingulate in the right hemisphere generated by parcellation of the pial surface using Desikan-Killiany parcellation	1.00E-01	1.74E-02
752	26959	aparc-pial_rh_area_caudalmiddlefrontal	Desikan Atlas	cortical area	mm2	Area of caudalmiddlefrontal in the right hemisphere generated by parcellation of the pial surface using Desikan-Killiany parcellation	1.92E-01	2.25E-02

753	26960	aparc-pial_rh_area_cuneus	Desikan Atlas	cortical area	mm2	Area of cuneus in the right hemisphere generated by parcellation of the pial surface using Desikan-Killiany parcellation	2.71E-01	2.33E-02
754	26961	aparc-pial_rh_area_entorhinal	Desikan Atlas	cortical area	mm2	Area of entorhinal in the right hemisphere generated by parcellation of the pial surface using Desikan-Killiany parcellation	1.27E-01	1.70E-02
755	26962	aparc-pial_rh_area_fusiform	Desikan Atlas	cortical area	mm2	Area of fusiform in the right hemisphere generated by parcellation of the pial surface using Desikan-Killiany parcellation	1.79E-01	1.90E-02
756	26963	aparc-pial_rh_area_inferiorparietal	Desikan Atlas	cortical area	mm2	Area of inferiorparietal in the right hemisphere generated by parcellation of the pial surface using Desikan-Killiany parcellation	1.75E-01	1.82E-02
757	26964	aparc-pial_rh_area_inferiortemporal	Desikan Atlas	cortical area	mm2	Area of inferiortemporal in the right hemisphere generated by parcellation of the pial surface using Desikan-Killiany parcellation	1.87E-01	1.72E-02
758	26965	aparc-pial_rh_area_isthmuscingulate	Desikan Atlas	cortical area	mm2	Area of isthmuscingulate in the right hemisphere generated by parcellation of the pial surface using Desikan-Killiany parcellation	1.56E-01	1.92E-02
759	26966	aparc-pial_rh_area_lateraloccipital	Desikan Atlas	cortical area	mm2	Area of lateraloccipital in the right hemisphere generated by parcellation of the pial surface using Desikan-Killiany parcellation	2.18E-01	2.15E-02
760	26967	aparc-pial_rh_area_lateralorbitofrontal	Desikan Atlas	cortical area	mm2	Area of lateralorbitofrontal in the right hemisphere generated by parcellation of the pial surface using Desikan-Killiany parcellation	1.89E-01	2.06E-02
761	26968	aparc-pial_rh_area_lingual	Desikan Atlas	cortical area	mm2	Area of lingual in the right hemisphere generated by parcellation of the pial surface using Desikan-Killiany parcellation	2.46E-01	2.32E-02
762	26969	aparc-pial_rh_area_medialorbitofrontal	Desikan Atlas	cortical area	mm2	Area of medialorbitofrontal in the right hemisphere generated by parcellation of the pial surface using Desikan-Killiany parcellation	1.63E-01	1.84E-02
763	26970	aparc-pial_rh_area_middletemporal	Desikan Atlas	cortical area	mm2	Area of middletemporal in the right hemisphere generated by parcellation of the pial surface using Desikan-Killiany parcellation	2.06E-01	2.07E-02

764	26971	aparc-pial_rh_area_parahippocampal	Desikan Atlas	cortical area	mm2	Area of parahippocampal in the right hemisphere generated by parcellation of the pial surface using Desikan-Killiany parcellation	1.79E-01	1.84E-02
765	26972	aparc-pial_rh_area_paracentral	Desikan Atlas	cortical area	mm2	Area of paracentral in the right hemisphere generated by parcellation of the pial surface using Desikan-Killiany parcellation	1.48E-01	2.29E-02
766	26973	aparc-pial_rh_area_parsopercularis	Desikan Atlas	cortical area	mm2	Area of parsopercularis in the right hemisphere generated by parcellation of the pial surface using Desikan-Killiany parcellation	1.12E-01	1.72E-02
767	26974	aparc-pial_rh_area_parsorbitalis	Desikan Atlas	cortical area	mm2	Area of parsorbitalis in the right hemisphere generated by parcellation of the pial surface using Desikan-Killiany parcellation	1.45E-01	1.67E-02
768	26975	aparc-pial_rh_area_parstriangularis	Desikan Atlas	cortical area	mm2	Area of parstriangularis in the right hemisphere generated by parcellation of the pial surface using Desikan-Killiany parcellation	1.35E-01	1.94E-02
769	26976	aparc-pial_rh_area_pericalcarine	Desikan Atlas	cortical area	mm2	Area of pericalcarine in the right hemisphere generated by parcellation of the pial surface using Desikan-Killiany parcellation	3.15E-01	2.95E-02
770	26977	aparc-pial_rh_area_postcentral	Desikan Atlas	cortical area	mm2	Area of postcentral in the right hemisphere generated by parcellation of the pial surface using Desikan-Killiany parcellation	1.73E-01	1.93E-02
771	26978	aparc-pial_rh_area_posteriorcingulate	Desikan Atlas	cortical area	mm2	Area of posteriorcingulate in the right hemisphere generated by parcellation of the pial surface using Desikan-Killiany parcellation	1.58E-01	2.41E-02
772	26979	aparc-pial_rh_area_precentral	Desikan Atlas	cortical area	mm2	Area of precentral in the right hemisphere generated by parcellation of the pial surface using Desikan-Killiany parcellation	2.59E-01	3.05E-02
773	26980	aparc-pial_rh_area_precuneus	Desikan Atlas	cortical area	mm2	Area of precuneus in the right hemisphere generated by parcellation of the pial surface using Desikan-Killiany parcellation	2.06E-01	1.91E-02
774	26981	aparc-pial_rh_area_rostralanteriorcingulate	Desikan Atlas	cortical area	mm2	Area of rostralanteriorcingulate in the right hemisphere generated by parcellation of the pial surface using Desikan-Killiany parcellation	9.38E-02	1.44E-02

775	26982	aparc-pial_rh_area_rostralmiddlefrontal	Desikan Atlas	cortical area	mm2	Area of rostralmiddlefrontal in the right hemisphere generated by parcellation of the pial surface using Desikan-Killiany parcellation	2.30E-01	1.92E-02
776	26983	aparc-pial_rh_area_superiorfrontal	Desikan Atlas	cortical area	mm2	Area of superiorfrontal in the right hemisphere generated by parcellation of the pial surface using Desikan-Killiany parcellation	1.94E-01	1.88E-02
777	26984	aparc-pial_rh_area_superiorparietal	Desikan Atlas	cortical area	mm2	Area of superiorparietal in the right hemisphere generated by parcellation of the pial surface using Desikan-Killiany parcellation	1.82E-01	1.84E-02
778	26985	aparc-pial_rh_area_superiortemporal	Desikan Atlas	cortical area	mm2	Area of superiortemporal in the right hemisphere generated by parcellation of the pial surface using Desikan-Killiany parcellation	2.24E-01	2.75E-02
779	26986	aparc-pial_rh_area_supramarginal	Desikan Atlas	cortical area	mm2	Area of supramarginal in the right hemisphere generated by parcellation of the pial surface using Desikan-Killiany parcellation	1.60E-01	1.81E-02
780	26987	aparc-pial_rh_area_frontalpole	Desikan Atlas	cortical area	mm2	Area of frontalpole in the right hemisphere generated by parcellation of the pial surface using Desikan-Killiany parcellation	5.95E-02	1.41E-02
781	26988	aparc-pial_rh_area_transversetemporal	Desikan Atlas	cortical area	mm2	Area of transversetemporal in the right hemisphere generated by parcellation of the pial surface using Desikan-Killiany parcellation	1.79E-01	1.84E-02
782	27059	BA-exvivo_lh_area_BA1	Broadmann Atlas	cortical area	mm2	Area of BA1 in the left hemisphere generated by parcellation of the white surface using BA_exvivo parcellation	1.54E-01	1.84E-02
783	27060	BA-exvivo_lh_area_BA2	Broadmann Atlas	cortical area	mm2	Area of BA2 in the left hemisphere generated by parcellation of the white surface using BA_exvivo parcellation	1.53E-01	1.79E-02
784	27061	BA-exvivo_lh_area_BA3a	Broadmann Atlas	cortical area	mm2	Area of BA3a in the left hemisphere generated by parcellation of the white surface using BA_exvivo parcellation	2.07E-01	2.34E-02
785	27062	BA-exvivo_lh_area_BA3b	Broadmann Atlas	cortical area	mm2	Area of BA3b in the left hemisphere generated by parcellation of the white surface using BA_exvivo parcellation	2.07E-01	2.24E-02

786	27063	BA-exvivo_lh_area_BA4a	Broadmann Atlas	cortical area	mm2	Area of BA4a in the left hemisphere generated by parcellation of the white surface using BA_exvivo parcellation	2.20E-01	2.15E-02
787	27064	BA-exvivo_lh_area_BA4p	Broadmann Atlas	cortical area	mm2	Area of BA4p in the left hemisphere generated by parcellation of the white surface using BA_exvivo parcellation	2.20E-01	2.44E-02
788	27065	BA-exvivo_lh_area_BA6	Broadmann Atlas	cortical area	mm2	Area of BA6 in the left hemisphere generated by parcellation of the white surface using BA_exvivo parcellation	2.45E-01	2.96E-02
789	27066	BA-exvivo_lh_area_BA44	Broadmann Atlas	cortical area	mm2	Area of BA44 in the left hemisphere generated by parcellation of the white surface using BA_exvivo parcellation	1.33E-01	1.79E-02
790	27067	BA-exvivo_lh_area_BA45	Broadmann Atlas	cortical area	mm2	Area of BA45 in the left hemisphere generated by parcellation of the white surface using BA_exvivo parcellation	1.74E-01	1.84E-02
791	27068	BA-exvivo_lh_area_V1	Broadmann Atlas	cortical area	mm2	Area of V1 in the left hemisphere generated by parcellation of the white surface using BA_exvivo parcellation	3.27E-01	2.83E-02
792	27069	BA-exvivo_lh_area_V2	Broadmann Atlas	cortical area	mm2	Area of V2 in the left hemisphere generated by parcellation of the white surface using BA_exvivo parcellation	2.98E-01	2.40E-02
793	27070	BA-exvivo_lh_area_MT	Broadmann Atlas	cortical area	mm2	Area of MT in the left hemisphere generated by parcellation of the white surface using BA_exvivo parcellation	1.44E-01	1.73E-02
794	27071	BA-exvivo_lh_area_perirhinal	Broadmann Atlas	cortical area	mm2	Area of perirhinal in the left hemisphere generated by parcellation of the white surface using BA_exvivo parcellation	1.77E-01	1.92E-02
795	27072	BA-exvivo_lh_area_entorhinal	Broadmann Atlas	cortical area	mm2	Area of entorhinal in the left hemisphere generated by parcellation of the white surface using BA_exvivo parcellation	2.18E-01	2.19E-02
796	27101	BA-exvivo_rh_area_BA1	Broadmann Atlas	cortical area	mm2	Area of BA1 in the right hemisphere generated by parcellation of the white surface using BA_exvivo parcellation	1.70E-01	1.97E-02

797	27102	BA-exvivo_rh_area_BA2	Broadmann Atlas	cortical area	mm2	Area of BA2 in the right hemisphere generated by parcellation of the white surface using BA_exvivo parcellation	1.40E-01	1.78E-02
798	27103	BA-exvivo_rh_area_BA3a	Broadmann Atlas	cortical area	mm2	Area of BA3a in the right hemisphere generated by parcellation of the white surface using BA_exvivo parcellation	2.33E-01	2.81E-02
799	27104	BA-exvivo_rh_area_BA3b	Broadmann Atlas	cortical area	mm2	Area of BA3b in the right hemisphere generated by parcellation of the white surface using BA_exvivo parcellation	2.30E-01	3.24E-02
800	27105	BA-exvivo_rh_area_BA4a	Broadmann Atlas	cortical area	mm2	Area of BA4a in the right hemisphere generated by parcellation of the white surface using BA_exvivo parcellation	2.20E-01	2.48E-02
801	27106	BA-exvivo_rh_area_BA4p	Broadmann Atlas	cortical area	mm2	Area of BA4p in the right hemisphere generated by parcellation of the white surface using BA_exvivo parcellation	2.37E-01	2.63E-02
802	27107	BA-exvivo_rh_area_BA6	Broadmann Atlas	cortical area	mm2	Area of BA6 in the right hemisphere generated by parcellation of the white surface using BA_exvivo parcellation	2.27E-01	2.57E-02
803	27108	BA-exvivo_rh_area_BA44	Broadmann Atlas	cortical area	mm2	Area of BA44 in the right hemisphere generated by parcellation of the white surface using BA_exvivo parcellation	1.68E-01	1.83E-02
804	27109	BA-exvivo_rh_area_BA45	Broadmann Atlas	cortical area	mm2	Area of BA45 in the right hemisphere generated by parcellation of the white surface using BA_exvivo parcellation	1.45E-01	1.91E-02
805	27110	BA-exvivo_rh_area_V1	Broadmann Atlas	cortical area	mm2	Area of V1 in the right hemisphere generated by parcellation of the white surface using BA_exvivo parcellation	3.45E-01	3.07E-02
806	27111	BA-exvivo_rh_area_V2	Broadmann Atlas	cortical area	mm2	Area of V2 in the right hemisphere generated by parcellation of the white surface using BA_exvivo parcellation	3.10E-01	2.62E-02
807	27112	BA-exvivo_rh_area_MT	Broadmann Atlas	cortical area	mm2	Area of MT in the right hemisphere generated by parcellation of the white surface using BA_exvivo parcellation	1.61E-01	1.95E-02

808	27113	BA-exvivo_rh_area_perirhinal	Broadmann Atlas	cortical area	mm2	Area of perirhinal in the right hemisphere generated by parcellation of the white surface using BA_exvivo parcellation	1.86E-01	2.11E-02
809	27114	BA-exvivo_rh_area_entorhinal	Broadmann Atlas	cortical area	mm2	Area of entorhinal in the right hemisphere generated by parcellation of the white surface using BA_exvivo parcellation	1.88E-01	2.04E-02
810	27143	aparc-DKTatlas_lh_area_caudalanteriorcingulate	Desikan Atlas	cortical area	mm2	Area of caudalanteriorcingulate in the left hemisphere generated by parcellation of the white surface using DKT parcellation	2.19E-01	2.82E-02
811	27144	aparc-DKTatlas_lh_area_caudalmiddlefrontal	Desikan Atlas	cortical area	mm2	Area of caudalmiddlefrontal in the left hemisphere generated by parcellation of the white surface using DKT parcellation	1.85E-01	2.31E-02
812	27145	aparc-DKTatlas_lh_area_cuneus	Desikan Atlas	cortical area	mm2	Area of cuneus in the left hemisphere generated by parcellation of the white surface using DKT parcellation	2.60E-01	2.22E-02
813	27146	aparc-DKTatlas_lh_area_entorhinal	Desikan Atlas	cortical area	mm2	Area of entorhinal in the left hemisphere generated by parcellation of the white surface using DKT parcellation	1.81E-01	2.01E-02
814	27147	aparc-DKTatlas_lh_area_fusiform	Desikan Atlas	cortical area	mm2	Area of fusiform in the left hemisphere generated by parcellation of the white surface using DKT parcellation	1.83E-01	1.96E-02
815	27148	aparc-DKTatlas_lh_area_inferiorparietal	Desikan Atlas	cortical area	mm2	Area of inferiorparietal in the left hemisphere generated by parcellation of the white surface using DKT parcellation	2.12E-01	1.81E-02
816	27149	aparc-DKTatlas_lh_area_inferiortemporal	Desikan Atlas	cortical area	mm2	Area of inferiortemporal in the left hemisphere generated by parcellation of the white surface using DKT parcellation	2.02E-01	1.76E-02
817	27150	aparc-DKTatlas_lh_area_isthmuscingulate	Desikan Atlas	cortical area	mm2	Area of isthmuscingulate in the left hemisphere generated by parcellation of the white surface using DKT parcellation	2.10E-01	2.13E-02
818	27151	aparc-DKTatlas_lh_area_lateraloccipital	Desikan Atlas	cortical area	mm2	Area of lateraloccipital in the left hemisphere generated by parcellation of the white surface using DKT parcellation	2.23E-01	2.15E-02
819	27152	aparc-DKTatlas_lh_area_lateralorbitofrontal	Desikan Atlas	cortical area	mm2	Area of lateralorbitofrontal in the left hemisphere generated by parcellation of the white surface using DKT parcellation	2.50E-01	2.17E-02

820	27153	aparc-DKTatlas_lh_area_lingual	Desikan Atlas	cortical area	mm2	Area of lingual in the left hemisphere generated by parcellation of the white surface using DKT parcellation	2.56E-01	2.30E-02
821	27154	aparc-DKTatlas_lh_area_medialorbitofrontal	Desikan Atlas	cortical area	mm2	Area of medialorbitofrontal in the left hemisphere generated by parcellation of the white surface using DKT parcellation	1.56E-01	1.73E-02
822	27155	aparc-DKTatlas_lh_area_middletemporal	Desikan Atlas	cortical area	mm2	Area of middletemporal in the left hemisphere generated by parcellation of the white surface using DKT parcellation	2.53E-01	2.02E-02
823	27156	aparc-DKTatlas_lh_area_parahippocampal	Desikan Atlas	cortical area	mm2	Area of parahippocampal in the left hemisphere generated by parcellation of the white surface using DKT parcellation	1.80E-01	1.99E-02
824	27157	aparc-DKTatlas_lh_area_paracentral	Desikan Atlas	cortical area	mm2	Area of paracentral in the left hemisphere generated by parcellation of the white surface using DKT parcellation	2.21E-01	2.14E-02
825	27158	aparc-DKTatlas_lh_area_parsopercularis	Desikan Atlas	cortical area	mm2	Area of parsopercularis in the left hemisphere generated by parcellation of the white surface using DKT parcellation	1.38E-01	1.66E-02
826	27159	aparc-DKTatlas_lh_area_parsorbitalis	Desikan Atlas	cortical area	mm2	Area of parsorbitalis in the left hemisphere generated by parcellation of the white surface using DKT parcellation	1.94E-01	1.91E-02
827	27160	aparc-DKTatlas_lh_area_parstriangularis	Desikan Atlas	cortical area	mm2	Area of parstriangularis in the left hemisphere generated by parcellation of the white surface using DKT parcellation	1.72E-01	1.90E-02
828	27161	aparc-DKTatlas_lh_area_pericalcarine	Desikan Atlas	cortical area	mm2	Area of pericalcarine in the left hemisphere generated by parcellation of the white surface using DKT parcellation	3.26E-01	2.91E-02
829	27162	aparc-DKTatlas_lh_area_postcentral	Desikan Atlas	cortical area	mm2	Area of postcentral in the left hemisphere generated by parcellation of the white surface using DKT parcellation	1.78E-01	1.93E-02
830	27163	aparc-DKTatlas_lh_area_posteriorcingulate	Desikan Atlas	cortical area	mm2	Area of posteriorcingulate in the left hemisphere generated by parcellation of the white surface using DKT parcellation	1.72E-01	3.63E-02
831	27164	aparc-DKTatlas_lh_area_precentral	Desikan Atlas	cortical area	mm2	Area of precentral in the left hemisphere generated by parcellation of the white surface using DKT parcellation	2.36E-01	2.67E-02
832	27165	aparc-DKTatlas_lh_area_precuneus	Desikan Atlas	cortical area	mm2	Area of precuneus in the left hemisphere generated by parcellation of the white surface using DKT parcellation	2.61E-01	2.32E-02

833	27166	aparc-DKTatlas_lh_area_rostralanteriorcingulate	Desikan Atlas	cortical area	mm2	Area of rostralanteriorcingulate in the left hemisphere generated by parcellation of the white surface using DKT parcellation	1.97E-01	2.19E-02
834	27167	aparc-DKTatlas_lh_area_rostralmiddlefrontal	Desikan Atlas	cortical area	mm2	Area of rostralmiddlefrontal in the left hemisphere generated by parcellation of the white surface using DKT parcellation	2.30E-01	1.91E-02
835	27168	aparc-DKTatlas_lh_area_superiorfrontal	Desikan Atlas	cortical area	mm2	Area of superiorfrontal in the left hemisphere generated by parcellation of the white surface using DKT parcellation	2.45E-01	2.22E-02
836	27169	aparc-DKTatlas_lh_area_superiorparietal	Desikan Atlas	cortical area	mm2	Area of superiorparietal in the left hemisphere generated by parcellation of the white surface using DKT parcellation	2.02E-01	2.00E-02
837	27170	aparc-DKTatlas_lh_area_superiortemporal	Desikan Atlas	cortical area	mm2	Area of superiortemporal in the left hemisphere generated by parcellation of the white surface using DKT parcellation	2.63E-01	3.69E-02
838	27171	aparc-DKTatlas_lh_area_supramarginal	Desikan Atlas	cortical area	mm2	Area of supramarginal in the left hemisphere generated by parcellation of the white surface using DKT parcellation	1.84E-01	1.80E-02
839	27172	aparc-DKTatlas_lh_area_transversetemporal	Desikan Atlas	cortical area	mm2	Area of transversetemporal in the left hemisphere generated by parcellation of the white surface using DKT parcellation	2.02E-01	1.93E-02
840	27173	aparc-DKTatlas_lh_area_insula	Desikan Atlas	cortical area	mm2	Area of insula in the left hemisphere generated by parcellation of the white surface using DKT parcellation	2.88E-01	2.21E-02
841	27236	aparc-DKTatlas_rh_area_caudalanteriorcingulate	Desikan Atlas	cortical area	mm2	Area of caudalanteriorcingulate in the right hemisphere generated by parcellation of the white surface using DKT parcellation	1.15E-01	1.65E-02
842	27237	aparc-DKTatlas_rh_area_caudalmiddlefrontal	Desikan Atlas	cortical area	mm2	Area of caudalmiddlefrontal in the right hemisphere generated by parcellation of the white surface using DKT parcellation	2.14E-01	2.49E-02
843	27238	aparc-DKTatlas_rh_area_cuneus	Desikan Atlas	cortical area	mm2	Area of cuneus in the right hemisphere generated by parcellation of the white surface using DKT parcellation	2.63E-01	2.19E-02
844	27239	aparc-DKTatlas_rh_area_entorhinal	Desikan Atlas	cortical area	mm2	Area of entorhinal in the right hemisphere generated by parcellation of the white surface using DKT parcellation	1.65E-01	2.13E-02

845	27240	aparc-DKTatlas_rh_area_fusiform	Desikan Atlas	cortical area	mm2	Area of fusiform in the right hemisphere generated by parcellation of the white surface using DKT parcellation	1.87E-01	2.08E-02
846	27241	aparc-DKTatlas_rh_area_inferiorparietal	Desikan Atlas	cortical area	mm2	Area of inferiorparietal in the right hemisphere generated by parcellation of the white surface using DKT parcellation	1.94E-01	1.91E-02
847	27242	aparc-DKTatlas_rh_area_inferiortemporal	Desikan Atlas	cortical area	mm2	Area of inferiortemporal in the right hemisphere generated by parcellation of the white surface using DKT parcellation	2.34E-01	1.81E-02
848	27243	aparc-DKTatlas_rh_area_isthmuscingulate	Desikan Atlas	cortical area	mm2	Area of isthmuscingulate in the right hemisphere generated by parcellation of the white surface using DKT parcellation	1.82E-01	2.01E-02
849	27244	aparc-DKTatlas_rh_area_lateraloccipital	Desikan Atlas	cortical area	mm2	Area of lateraloccipital in the right hemisphere generated by parcellation of the white surface using DKT parcellation	2.44E-01	2.28E-02
850	27245	aparc-DKTatlas_rh_area_lateralorbitofrontal	Desikan Atlas	cortical area	mm2	Area of lateralorbitofrontal in the right hemisphere generated by parcellation of the white surface using DKT parcellation	2.34E-01	2.14E-02
851	27246	aparc-DKTatlas_rh_area_lingual	Desikan Atlas	cortical area	mm2	Area of lingual in the right hemisphere generated by parcellation of the white surface using DKT parcellation	2.55E-01	2.43E-02
852	27247	aparc-DKTatlas_rh_area_medialorbitofrontal	Desikan Atlas	cortical area	mm2	Area of medialorbitofrontal in the right hemisphere generated by parcellation of the white surface using DKT parcellation	1.70E-01	1.96E-02
853	27248	aparc-DKTatlas_rh_area_middletemporal	Desikan Atlas	cortical area	mm2	Area of middletemporal in the right hemisphere generated by parcellation of the white surface using DKT parcellation	2.54E-01	2.25E-02
854	27249	aparc-DKTatlas_rh_area_parahippocampal	Desikan Atlas	cortical area	mm2	Area of parahippocampal in the right hemisphere generated by parcellation of the white surface using DKT parcellation	1.60E-01	1.79E-02
855	27250	aparc-DKTatlas_rh_area_paracentral	Desikan Atlas	cortical area	mm2	Area of paracentral in the right hemisphere generated by parcellation of the white surface using DKT parcellation	2.10E-01	2.56E-02
856	27251	aparc-DKTatlas_rh_area_parsopercularis	Desikan Atlas	cortical area	mm2	Area of parsopercularis in the right hemisphere generated by parcellation of the white surface using DKT parcellation	1.47E-01	1.83E-02
857	27252	aparc-DKTatlas_rh_area_parsorbitalis	Desikan Atlas	cortical area	mm2	Area of parsorbitalis in the right hemisphere generated by parcellation of the white surface using DKT parcellation	1.33E-01	1.78E-02

858	27253	aparc-DKTatlas_rh_area_parstriangularis	Desikan Atlas	cortical area	mm2	Area of parstriangularis in the right hemisphere generated by parcellation of the white surface using DKT parcellation	1.34E-01	1.91E-02
859	27254	aparc-DKTatlas_rh_area_pericalcarine	Desikan Atlas	cortical area	mm2	Area of pericalcarine in the right hemisphere generated by parcellation of the white surface using DKT parcellation	3.41E-01	3.06E-02
860	27255	aparc-DKTatlas_rh_area_postcentral	Desikan Atlas	cortical area	mm2	Area of postcentral in the right hemisphere generated by parcellation of the white surface using DKT parcellation	1.76E-01	1.99E-02
861	27256	aparc-DKTatlas_rh_area_posteriorcingulate	Desikan Atlas	cortical area	mm2	Area of posteriorcingulate in the right hemisphere generated by parcellation of the white surface using DKT parcellation	1.86E-01	2.38E-02
862	27257	aparc-DKTatlas_rh_area_precentral	Desikan Atlas	cortical area	mm2	Area of precentral in the right hemisphere generated by parcellation of the white surface using DKT parcellation	2.34E-01	2.83E-02
863	27258	aparc-DKTatlas_rh_area_precuneus	Desikan Atlas	cortical area	mm2	Area of precuneus in the right hemisphere generated by parcellation of the white surface using DKT parcellation	2.33E-01	2.07E-02
864	27259	aparc-DKTatlas_rh_area_rostralanteriorcingulate	Desikan Atlas	cortical area	mm2	Area of rostralanteriorcingulate in the right hemisphere generated by parcellation of the white surface using DKT parcellation	1.34E-01	1.69E-02
865	27260	aparc-DKTatlas_rh_area_rostralmiddlefrontal	Desikan Atlas	cortical area	mm2	Area of rostralmiddlefrontal in the right hemisphere generated by parcellation of the white surface using DKT parcellation	2.20E-01	1.84E-02
866	27261	aparc-DKTatlas_rh_area_superiorfrontal	Desikan Atlas	cortical area	mm2	Area of superiorfrontal in the right hemisphere generated by parcellation of the white surface using DKT parcellation	2.17E-01	2.02E-02
867	27262	aparc-DKTatlas_rh_area_superiorparietal	Desikan Atlas	cortical area	mm2	Area of superiorparietal in the right hemisphere generated by parcellation of the white surface using DKT parcellation	1.83E-01	1.79E-02
868	27263	aparc-DKTatlas_rh_area_superiortemporal	Desikan Atlas	cortical area	mm2	Area of superiortemporal in the right hemisphere generated by parcellation of the white surface using DKT parcellation	2.63E-01	3.04E-02
869	27264	aparc-DKTatlas_rh_area_supramarginal	Desikan Atlas	cortical area	mm2	Area of supramarginal in the right hemisphere generated by parcellation of the white surface using DKT parcellation	1.67E-01	1.90E-02
870	27265	aparc-DKTatlas_rh_area_transversetemporal	Desikan Atlas	cortical area	mm2	Area of transversetemporal in the right hemisphere generated by parcellation of the white surface using DKT	2.20E-01	2.02E-02

						parcellation		
871	27266	aparc-DKTatlas_rh_area_insula	Desikan Atlas	cortical area	mm2	Area of insula in the right hemisphere generated by parcellation of the white surface using DKT parcellation	2.97E-01	2.40E-02
872	27329	aparc-a2009s_lh_area_G+S-frontomargin	Destrieux Atlas	cortical area	mm2	Area of G+S-frontomargin in the left hemisphere generated by parcellation of the white surface using Destrieux (a2009s) parcellation	1.35E-01	1.59E-02
873	27330	aparc-a2009s_lh_area_G+S-occipital-inf	Destrieux Atlas	cortical area	mm2	Area of G+S-occipital-inf in the left hemisphere generated by parcellation of the white surface using Destrieux (a2009s) parcellation	9.43E-02	1.71E-02
874	27331	aparc-a2009s_lh_area_G+S-paracentral	Destrieux Atlas	cortical area	mm2	Area of G+S-paracentral in the left hemisphere generated by parcellation of the white surface using Destrieux (a2009s) parcellation	1.68E-01	1.81E-02
875	27332	aparc-a2009s_lh_area_G+S-subcentral	Destrieux Atlas	cortical area	mm2	Area of G+S-subcentral in the left hemisphere generated by parcellation of the white surface using Destrieux (a2009s) parcellation	1.60E-01	1.85E-02
876	27333	aparc-a2009s_lh_area_G+S-transv-frontopol	Destrieux Atlas	cortical area	mm2	Area of G+S-transv-frontopol in the left hemisphere generated by parcellation of the white surface using Destrieux (a2009s) parcellation	1.11E-01	1.83E-02
877	27334	aparc-a2009s_lh_area_G+S-cingul-Ant	Destrieux Atlas	cortical area	mm2	Area of G+S-cingul-Ant in the left hemisphere generated by parcellation of the white surface using Destrieux (a2009s) parcellation	1.72E-01	1.99E-02
878	27335	aparc-a2009s_lh_area_G+S-cingul-Mid-Ant	Destrieux Atlas	cortical area	mm2	Area of G+S-cingul-Mid-Ant in the left hemisphere generated by parcellation of the white surface using Destrieux (a2009s) parcellation	1.52E-01	2.33E-02
879	27336	aparc-a2009s_lh_area_G+S-cingul-Mid-Post	Destrieux Atlas	cortical area	mm2	Area of G+S-cingul-Mid-Post in the left hemisphere generated by parcellation of the white surface using Destrieux (a2009s) parcellation	1.37E-01	2.82E-02
880	27337	aparc-a2009s_lh_area_G-cingul-Post-dorsal	Destrieux Atlas	cortical area	mm2	Area of G-cingul-Post-dorsal in the left hemisphere generated by parcellation of the white surface using Destrieux (a2009s) parcellation	1.62E-01	1.85E-02

881	27338	aparc-a2009s_lh_area_G-cingul-Post-ventral	Destrieux Atlas	cortical area	mm2	Area of G-cingul-Post-ventral in the left hemisphere generated by parcellation of the white surface using Destrieux (a2009s) parcellation	1.91E-01	1.85E-02
882	27339	aparc-a2009s_lh_area_G-cuneus	Destrieux Atlas	cortical area	mm2	Area of G-cuneus in the left hemisphere generated by parcellation of the white surface using Destrieux (a2009s) parcellation	2.88E-01	2.38E-02
883	27340	aparc-a2009s_lh_area_G-front-inf-Opercular	Destrieux Atlas	cortical area	mm2	Area of G-front-inf-Opercular in the left hemisphere generated by parcellation of the white surface using Destrieux (a2009s) parcellation	8.94E-02	1.48E-02
884	27341	aparc-a2009s_lh_area_G-front-inf-Orbital	Destrieux Atlas	cortical area	mm2	Area of G-front-inf-Orbital in the left hemisphere generated by parcellation of the white surface using Destrieux (a2009s) parcellation	7.59E-02	1.61E-02
885	27342	aparc-a2009s_lh_area_G-front-inf-Triangul	Destrieux Atlas	cortical area	mm2	Area of G-front-inf-Triangul in the left hemisphere generated by parcellation of the white surface using Destrieux (a2009s) parcellation	1.37E-01	1.77E-02
886	27343	aparc-a2009s_lh_area_G-front-middle	Destrieux Atlas	cortical area	mm2	Area of G-front-middle in the left hemisphere generated by parcellation of the white surface using Destrieux (a2009s) parcellation	2.21E-01	2.17E-02
887	27344	aparc-a2009s_lh_area_G-front-sup	Destrieux Atlas	cortical area	mm2	Area of G-front-sup in the left hemisphere generated by parcellation of the white surface using Destrieux (a2009s) parcellation	2.28E-01	2.56E-02
888	27345	aparc-a2009s_lh_area_G-Ins-lg+S-cent-ins	Destrieux Atlas	cortical area	mm2	Area of G-Ins-lg+S-cent-ins in the left hemisphere generated by parcellation of the white surface using Destrieux (a2009s) parcellation	1.43E-01	1.85E-02
889	27346	aparc-a2009s_lh_area_G-insular-short	Destrieux Atlas	cortical area	mm2	Area of G-insular-short in the left hemisphere generated by parcellation of the white surface using Destrieux (a2009s) parcellation	1.55E-01	1.70E-02
890	27347	aparc-a2009s_lh_area_G-occipital-middle	Destrieux Atlas	cortical area	mm2	Area of G-occipital-middle in the left hemisphere generated by parcellation of the white surface using Destrieux (a2009s) parcellation	1.56E-01	1.95E-02
891	27348	aparc-a2009s_lh_area_G-occipital-sup	Destrieux Atlas	cortical area	mm2	Area of G-occipital-sup in the left hemisphere generated by parcellation of the white surface using Destrieux (a2009s)	1.91E-01	2.06E-02

						parcellation		
892	27349	aparc-a2009s_lh_area_G-oc-temp-lat-fusifor	Destrieux Atlas	cortical area	mm2	Area of G-oc-temp-lat-fusifor in the left hemisphere generated by parcellation of the white surface using Destrieux (a2009s) parcellation	1.49E-01	1.73E-02
893	27350	aparc-a2009s_lh_area_G-oc-temp-med-Lingual	Destrieux Atlas	cortical area	mm2	Area of G-oc-temp-med-Lingual in the left hemisphere generated by parcellation of the white surface using Destrieux (a2009s) parcellation	2.42E-01	2.40E-02
894	27351	aparc-a2009s_lh_area_G-oc-temp-med-Parahip	Destrieux Atlas	cortical area	mm2	Area of G-oc-temp-med-Parahip in the left hemisphere generated by parcellation of the white surface using Destrieux (a2009s) parcellation	1.96E-01	2.00E-02
895	27352	aparc-a2009s_lh_area_G-orbital	Destrieux Atlas	cortical area	mm2	Area of G-orbital in the left hemisphere generated by parcellation of the white surface using Destrieux (a2009s) parcellation	2.26E-01	2.04E-02
896	27353	aparc-a2009s_lh_area_G-pariet-inf-Angular	Destrieux Atlas	cortical area	mm2	Area of G-pariet-inf-Angular in the left hemisphere generated by parcellation of the white surface using Destrieux (a2009s) parcellation	1.67E-01	1.90E-02
897	27354	aparc-a2009s_lh_area_G-pariet-inf-Supramar	Destrieux Atlas	cortical area	mm2	Area of G-pariet-inf-Supramar in the left hemisphere generated by parcellation of the white surface using Destrieux (a2009s) parcellation	1.89E-01	1.78E-02
898	27355	aparc-a2009s_lh_area_G-parietal-sup	Destrieux Atlas	cortical area	mm2	Area of G-parietal-sup in the left hemisphere generated by parcellation of the white surface using Destrieux (a2009s) parcellation	1.81E-01	1.83E-02
899	27356	aparc-a2009s_lh_area_G-postcentral	Destrieux Atlas	cortical area	mm2	Area of G-postcentral in the left hemisphere generated by parcellation of the white surface using Destrieux (a2009s) parcellation	1.38E-01	1.80E-02
900	27357	aparc-a2009s_lh_area_G-precentral	Destrieux Atlas	cortical area	mm2	Area of G-precentral in the left hemisphere generated by parcellation of the white surface using Destrieux (a2009s) parcellation	2.05E-01	2.52E-02
901	27358	aparc-a2009s_lh_area_G-precuneus	Destrieux Atlas	cortical area	mm2	Area of G-precuneus in the left hemisphere generated by parcellation of the white surface using Destrieux (a2009s)	2.14E-01	2.14E-02

						parcellation		
902	27359	aparc-a2009s_lh_area_G-rectus	Destrieux Atlas	cortical area	mm2	Area of G-rectus in the left hemisphere generated by parcellation of the white surface using Destrieux (a2009s) parcellation	1.00E-01	1.79E-02
903	27360	aparc-a2009s_lh_area_G-subcallosal	Destrieux Atlas	cortical area	mm2	Area of G-subcallosal in the left hemisphere generated by parcellation of the white surface using Destrieux (a2009s) parcellation	6.21E-02	1.50E-02
904	27361	aparc-a2009s_lh_area_G-temp-sup-G-T-transv	Destrieux Atlas	cortical area	mm2	Area of G-temp-sup-G-T-transv in the left hemisphere generated by parcellation of the white surface using Destrieux (a2009s) parcellation	1.61E-01	1.72E-02
905	27362	aparc-a2009s_lh_area_G-temp-sup-Lateral	Destrieux Atlas	cortical area	mm2	Area of G-temp-sup-Lateral in the left hemisphere generated by parcellation of the white surface using Destrieux (a2009s) parcellation	2.11E-01	3.54E-02
906	27363	aparc-a2009s_lh_area_G-temp-sup-Plan-polar	Destrieux Atlas	cortical area	mm2	Area of G-temp-sup-Plan-polar in the left hemisphere generated by parcellation of the white surface using Destrieux (a2009s) parcellation	1.26E-01	2.23E-02
907	27364	aparc-a2009s_lh_area_G-temp-sup-Plan-tempo	Destrieux Atlas	cortical area	mm2	Area of G-temp-sup-Plan-tempo in the left hemisphere generated by parcellation of the white surface using Destrieux (a2009s) parcellation	1.07E-01	1.48E-02
908	27365	aparc-a2009s_lh_area_G-temporal-inf	Destrieux Atlas	cortical area	mm2	Area of G-temporal-inf in the left hemisphere generated by parcellation of the white surface using Destrieux (a2009s) parcellation	1.66E-01	1.67E-02
909	27366	aparc-a2009s_lh_area_G-temporal-middle	Destrieux Atlas	cortical area	mm2	Area of G-temporal-middle in the left hemisphere generated by parcellation of the white surface using Destrieux (a2009s) parcellation	1.98E-01	1.87E-02
910	27367	aparc-a2009s_lh_area_Lat-Fis-ant-Horizont	Destrieux Atlas	cortical area	mm2	Area of Lat-Fis-ant-Horizont in the left hemisphere generated by parcellation of the white surface using Destrieux (a2009s) parcellation	8.52E-02	1.69E-02

911	27368	aparc-a2009s_lh_area_Lat-Fis-ant-Vertical	Destrieux Atlas	cortical area	mm2	Area of Lat-Fis-ant-Vertical in the left hemisphere generated by parcellation of the white surface using Destrieux (a2009s) parcellation	6.32E-02	1.48E-02
912	27369	aparc-a2009s_lh_area_Lat-Fis-post	Destrieux Atlas	cortical area	mm2	Area of Lat-Fis-post in the left hemisphere generated by parcellation of the white surface using Destrieux (a2009s) parcellation	2.27E-01	1.92E-02
913	27370	aparc-a2009s_lh_area_Pole-occipital	Destrieux Atlas	cortical area	mm2	Area of Pole-occipital in the left hemisphere generated by parcellation of the white surface using Destrieux (a2009s) parcellation	1.63E-01	1.95E-02
914	27371	aparc-a2009s_lh_area_Pole-temporal	Destrieux Atlas	cortical area	mm2	Area of Pole-temporal in the left hemisphere generated by parcellation of the white surface using Destrieux (a2009s) parcellation	1.62E-01	2.07E-02
915	27372	aparc-a2009s_lh_area_S-calcarine	Destrieux Atlas	cortical area	mm2	Area of S-calcarine in the left hemisphere generated by parcellation of the white surface using Destrieux (a2009s) parcellation	3.23E-01	2.78E-02
916	27373	aparc-a2009s_lh_area_S-central	Destrieux Atlas	cortical area	mm2	Area of S-central in the left hemisphere generated by parcellation of the white surface using Destrieux (a2009s) parcellation	2.34E-01	2.76E-02
917	27374	aparc-a2009s_lh_area_S-cingul-Marginalis	Destrieux Atlas	cortical area	mm2	Area of S-cingul-Marginalis in the left hemisphere generated by parcellation of the white surface using Destrieux (a2009s) parcellation	1.65E-01	1.83E-02
918	27375	aparc-a2009s_lh_area_S-circular-insula-ant	Destrieux Atlas	cortical area	mm2	Area of S-circular-insula-ant in the left hemisphere generated by parcellation of the white surface using Destrieux (a2009s) parcellation	1.39E-01	1.68E-02
919	27376	aparc-a2009s_lh_area_S-circular-insula-inf	Destrieux Atlas	cortical area	mm2	Area of S-circular-insula-inf in the left hemisphere generated by parcellation of the white surface using Destrieux (a2009s) parcellation	1.95E-01	1.81E-02
920	27377	aparc-a2009s_lh_area_S-circular-insula-sup	Destrieux Atlas	cortical area	mm2	Area of S-circular-insula-sup in the left hemisphere generated by parcellation of the white surface using Destrieux (a2009s) parcellation	2.17E-01	2.12E-02

921	27378	aparc-a2009s_lh_area_S-collat-transv-ant	Destrieux Atlas	cortical area	mm2	Area of S-collat-transv-ant in the left hemisphere generated by parcellation of the white surface using Destrieux (a2009s) parcellation	2.05E-01	1.84E-02
922	27379	aparc-a2009s_lh_area_S-collat-transv-post	Destrieux Atlas	cortical area	mm2	Area of S-collat-transv-post in the left hemisphere generated by parcellation of the white surface using Destrieux (a2009s) parcellation	1.00E-01	1.62E-02
923	27380	aparc-a2009s_lh_area_S-front-inf	Destrieux Atlas	cortical area	mm2	Area of S-front-inf in the left hemisphere generated by parcellation of the white surface using Destrieux (a2009s) parcellation	1.40E-01	1.70E-02
924	27381	aparc-a2009s_lh_area_S-front-middle	Destrieux Atlas	cortical area	mm2	Area of S-front-middle in the left hemisphere generated by parcellation of the white surface using Destrieux (a2009s) parcellation	1.28E-01	1.47E-02
925	27382	aparc-a2009s_lh_area_S-front-sup	Destrieux Atlas	cortical area	mm2	Area of S-front-sup in the left hemisphere generated by parcellation of the white surface using Destrieux (a2009s) parcellation	1.57E-01	1.84E-02
926	27383	aparc-a2009s_lh_area_S-interm-prim-Jensen	Destrieux Atlas	cortical area	mm2	Area of S-interm-prim-Jensen in the left hemisphere generated by parcellation of the white surface using Destrieux (a2009s) parcellation	4.71E-02	1.26E-02
927	27384	aparc-a2009s_lh_area_S-intrapariet+P-trans	Destrieux Atlas	cortical area	mm2	Area of S-intrapariet+P-trans in the left hemisphere generated by parcellation of the white surface using Destrieux (a2009s) parcellation	1.28E-01	1.86E-02
928	27385	aparc-a2009s_lh_area_S-oc-middle+Lunatus	Destrieux Atlas	cortical area	mm2	Area of S-oc-middle+Lunatus in the left hemisphere generated by parcellation of the white surface using Destrieux (a2009s) parcellation	1.56E-01	1.83E-02
929	27386	aparc-a2009s_lh_area_S-oc-sup+transversal	Destrieux Atlas	cortical area	mm2	Area of S-oc-sup+transversal in the left hemisphere generated by parcellation of the white surface using Destrieux (a2009s) parcellation	1.27E-01	1.63E-02
930	27387	aparc-a2009s_lh_area_S-occipital-ant	Destrieux Atlas	cortical area	mm2	Area of S-occipital-ant in the left hemisphere generated by parcellation of the white surface using Destrieux (a2009s) parcellation	6.09E-02	1.33E-02

931	27388	aparc-a2009s_lh_area_S-oc-temp-lat	Destrieux Atlas	cortical area	mm2	Area of S-oc-temp-lat in the left hemisphere generated by parcellation of the white surface using Destrieux (a2009s) parcellation	1.09E-01	1.56E-02
932	27389	aparc-a2009s_lh_area_S-oc-temp-med+Lingual	Destrieux Atlas	cortical area	mm2	Area of S-oc-temp-med+Lingual in the left hemisphere generated by parcellation of the white surface using Destrieux (a2009s) parcellation	1.33E-01	1.60E-02
933	27390	aparc-a2009s_lh_area_S-orbital-lateral	Destrieux Atlas	cortical area	mm2	Area of S-orbital-lateral in the left hemisphere generated by parcellation of the white surface using Destrieux (a2009s) parcellation	7.29E-02	1.63E-02
934	27391	aparc-a2009s_lh_area_S-orbital-med-olfact	Destrieux Atlas	cortical area	mm2	Area of S-orbital-med-olfact in the left hemisphere generated by parcellation of the white surface using Destrieux (a2009s) parcellation	2.12E-01	2.36E-02
935	27392	aparc-a2009s_lh_area_S-orbital-H-Shaped	Destrieux Atlas	cortical area	mm2	Area of S-orbital-H-Shaped in the left hemisphere generated by parcellation of the white surface using Destrieux (a2009s) parcellation	2.44E-01	2.05E-02
936	27393	aparc-a2009s_lh_area_S-parieto-occipital	Destrieux Atlas	cortical area	mm2	Area of S-parieto-occipital in the left hemisphere generated by parcellation of the white surface using Destrieux (a2009s) parcellation	2.19E-01	1.93E-02
937	27394	aparc-a2009s_lh_area_S-pericallosal	Destrieux Atlas	cortical area	mm2	Area of S-pericallosal in the left hemisphere generated by parcellation of the white surface using Destrieux (a2009s) parcellation	1.72E-01	1.84E-02
938	27395	aparc-a2009s_lh_area_S-postcentral	Destrieux Atlas	cortical area	mm2	Area of S-postcentral in the left hemisphere generated by parcellation of the white surface using Destrieux (a2009s) parcellation	1.39E-01	1.84E-02
939	27396	aparc-a2009s_lh_area_S-precentral-inf-part	Destrieux Atlas	cortical area	mm2	Area of S-precentral-inf-part in the left hemisphere generated by parcellation of the white surface using Destrieux (a2009s) parcellation	1.09E-01	1.61E-02
940	27397	aparc-a2009s_lh_area_S-precentral-sup-part	Destrieux Atlas	cortical area	mm2	Area of S-precentral-sup-part in the left hemisphere generated by parcellation of the white surface using Destrieux (a2009s) parcellation	1.21E-01	1.70E-02

941	27398	aparc-a2009s_lh_area_S-suborbital	Destrieux Atlas	cortical area	mm2	Area of S-suborbital in the left hemisphere generated by parcellation of the white surface using Destrieux (a2009s) parcellation	5.36E-02	1.45E-02
942	27399	aparc-a2009s_lh_area_S-subparietal	Destrieux Atlas	cortical area	mm2	Area of S-subparietal in the left hemisphere generated by parcellation of the white surface using Destrieux (a2009s) parcellation	1.51E-01	1.93E-02
943	27400	aparc-a2009s_lh_area_S-temporal-inf	Destrieux Atlas	cortical area	mm2	Area of S-temporal-inf in the left hemisphere generated by parcellation of the white surface using Destrieux (a2009s) parcellation	1.34E-01	1.74E-02
944	27401	aparc-a2009s_lh_area_S-temporal-sup	Destrieux Atlas	cortical area	mm2	Area of S-temporal-sup in the left hemisphere generated by parcellation of the white surface using Destrieux (a2009s) parcellation	2.19E-01	1.88E-02
945	27402	aparc-a2009s_lh_area_S-temporal-transverse	Destrieux Atlas	cortical area	mm2	Area of S-temporal-transverse in the left hemisphere generated by parcellation of the white surface using Destrieux (a2009s) parcellation	1.09E-01	1.45E-02
946	27551	aparc-a2009s_rh_area_G+S-frontomargin	Destrieux Atlas	cortical area	mm2	Area of G+S-frontomargin in the right hemisphere generated by parcellation of the white surface using Destrieux (a2009s) parcellation	1.03E-01	1.73E-02
947	27552	aparc-a2009s_rh_area_G+S-occipital-inf	Destrieux Atlas	cortical area	mm2	Area of G+S-occipital-inf in the right hemisphere generated by parcellation of the white surface using Destrieux (a2009s) parcellation	1.15E-01	1.64E-02
948	27553	aparc-a2009s_rh_area_G+S-paracentral	Destrieux Atlas	cortical area	mm2	Area of G+S-paracentral in the right hemisphere generated by parcellation of the white surface using Destrieux (a2009s) parcellation	1.83E-01	2.14E-02
949	27554	aparc-a2009s_rh_area_G+S-subcentral	Destrieux Atlas	cortical area	mm2	Area of G+S-subcentral in the right hemisphere generated by parcellation of the white surface using Destrieux (a2009s) parcellation	1.63E-01	1.92E-02
950	27555	aparc-a2009s_rh_area_G+S-transv-frontopol	Destrieux Atlas	cortical area	mm2	Area of G+S-transv-frontopol in the right hemisphere generated by parcellation of the white surface using Destrieux (a2009s) parcellation	1.21E-01	1.64E-02
951	27556	aparc-a2009s_rh_area_G+S-cingul-Ant	Destrieux Atlas	cortical area	mm2	Area of G+S-cingul-Ant in the right hemisphere generated by parcellation of the white surface using Destrieux	1.90E-01	1.99E-02

						(a2009s) parcellation		
952	27557	aparc-a2009s_rh_area_G+S-cingul-Mid-Ant	Destrieux Atlas	cortical area	mm2	Area of G+S-cingul-Mid-Ant in the right hemisphere generated by parcellation of the white surface using Destrieux (a2009s) parcellation	2.01E-01	1.81E-02
953	27558	aparc-a2009s_rh_area_G+S-cingul-Mid-Post	Destrieux Atlas	cortical area	mm2	Area of G+S-cingul-Mid-Post in the right hemisphere generated by parcellation of the white surface using Destrieux (a2009s) parcellation	1.76E-01	1.97E-02
954	27559	aparc-a2009s_rh_area_G-cingul-Post-dorsal	Destrieux Atlas	cortical area	mm2	Area of G-cingul-Post-dorsal in the right hemisphere generated by parcellation of the white surface using Destrieux (a2009s) parcellation	1.52E-01	1.76E-02
955	27560	aparc-a2009s_rh_area_G-cingul-Post-ventral	Destrieux Atlas	cortical area	mm2	Area of G-cingul-Post-ventral in the right hemisphere generated by parcellation of the white surface using Destrieux (a2009s) parcellation	2.00E-01	1.91E-02
956	27561	aparc-a2009s_rh_area_G-cuneus	Destrieux Atlas	cortical area	mm2	Area of G-cuneus in the right hemisphere generated by parcellation of the white surface using Destrieux (a2009s) parcellation	2.74E-01	2.29E-02
957	27562	aparc-a2009s_rh_area_G-front-inf-Opercular	Destrieux Atlas	cortical area	mm2	Area of G-front-inf-Opercular in the right hemisphere generated by parcellation of the white surface using Destrieux (a2009s) parcellation	1.15E-01	1.66E-02
958	27563	aparc-a2009s_rh_area_G-front-inf-Orbital	Destrieux Atlas	cortical area	mm2	Area of G-front-inf-Orbital in the right hemisphere generated by parcellation of the white surface using Destrieux (a2009s) parcellation	7.33E-02	1.55E-02
959	27564	aparc-a2009s_rh_area_G-front-inf-Triangul	Destrieux Atlas	cortical area	mm2	Area of G-front-inf-Triangul in the right hemisphere generated by parcellation of the white surface using Destrieux (a2009s) parcellation	1.01E-01	1.64E-02
960	27565	aparc-a2009s_rh_area_G-front-middle	Destrieux Atlas	cortical area	mm2	Area of G-front-middle in the right hemisphere generated by parcellation of the white surface using Destrieux (a2009s) parcellation	2.00E-01	2.20E-02

961	27566	aparc-a2009s_rh_area_G-front-sup	Destrieux Atlas	cortical area	mm2	Area of G-front-sup in the right hemisphere generated by parcellation of the white surface using Destrieux (a2009s) parcellation	1.84E-01	1.99E-02
962	27567	aparc-a2009s_rh_area_G-Ins-lg+S-cent-ins	Destrieux Atlas	cortical area	mm2	Area of G-Ins-lg+S-cent-ins in the right hemisphere generated by parcellation of the white surface using Destrieux (a2009s) parcellation	1.38E-01	1.95E-02
963	27568	aparc-a2009s_rh_area_G-insular-short	Destrieux Atlas	cortical area	mm2	Area of G-insular-short in the right hemisphere generated by parcellation of the white surface using Destrieux (a2009s) parcellation	1.18E-01	1.61E-02
964	27569	aparc-a2009s_rh_area_G-occipital-middle	Destrieux Atlas	cortical area	mm2	Area of G-occipital-middle in the right hemisphere generated by parcellation of the white surface using Destrieux (a2009s) parcellation	1.24E-01	1.84E-02
965	27570	aparc-a2009s_rh_area_G-occipital-sup	Destrieux Atlas	cortical area	mm2	Area of G-occipital-sup in the right hemisphere generated by parcellation of the white surface using Destrieux (a2009s) parcellation	1.54E-01	1.71E-02
966	27571	aparc-a2009s_rh_area_G-oc-temp-lat-fusifor	Destrieux Atlas	cortical area	mm2	Area of G-oc-temp-lat-fusifor in the right hemisphere generated by parcellation of the white surface using Destrieux (a2009s) parcellation	1.46E-01	1.69E-02
967	27572	aparc-a2009s_rh_area_G-oc-temp-med-Lingual	Destrieux Atlas	cortical area	mm2	Area of G-oc-temp-med-Lingual in the right hemisphere generated by parcellation of the white surface using Destrieux (a2009s) parcellation	2.40E-01	2.22E-02
968	27573	aparc-a2009s_rh_area_G-oc-temp-med-Parahip	Destrieux Atlas	cortical area	mm2	Area of G-oc-temp-med-Parahip in the right hemisphere generated by parcellation of the white surface using Destrieux (a2009s) parcellation	1.69E-01	1.93E-02
969	27574	aparc-a2009s_rh_area_G-orbital	Destrieux Atlas	cortical area	mm2	Area of G-orbital in the right hemisphere generated by parcellation of the white surface using Destrieux (a2009s) parcellation	1.99E-01	2.01E-02
970	27575	aparc-a2009s_rh_area_G-pariet-inf-Angular	Destrieux Atlas	cortical area	mm2	Area of G-pariet-inf-Angular in the right hemisphere generated by parcellation of the white surface using Destrieux (a2009s) parcellation	1.40E-01	1.68E-02

971	27576	aparc-a2009s_rh_area_G-pariet-inf-Supramar	Destrieux Atlas	cortical area	mm2	Area of G-pariet-inf-Supramar in the right hemisphere generated by parcellation of the white surface using Destrieux (a2009s) parcellation	1.59E-01	2.01E-02
972	27577	aparc-a2009s_rh_area_G-parietal-sup	Destrieux Atlas	cortical area	mm2	Area of G-parietal-sup in the right hemisphere generated by parcellation of the white surface using Destrieux (a2009s) parcellation	1.42E-01	1.65E-02
973	27578	aparc-a2009s_rh_area_G-postcentral	Destrieux Atlas	cortical area	mm2	Area of G-postcentral in the right hemisphere generated by parcellation of the white surface using Destrieux (a2009s) parcellation	1.49E-01	1.96E-02
974	27579	aparc-a2009s_rh_area_G-precentral	Destrieux Atlas	cortical area	mm2	Area of G-precentral in the right hemisphere generated by parcellation of the white surface using Destrieux (a2009s) parcellation	2.08E-01	2.57E-02
975	27580	aparc-a2009s_rh_area_G-precuneus	Destrieux Atlas	cortical area	mm2	Area of G-precuneus in the right hemisphere generated by parcellation of the white surface using Destrieux (a2009s) parcellation	1.95E-01	1.95E-02
976	27581	aparc-a2009s_rh_area_G-rectus	Destrieux Atlas	cortical area	mm2	Area of G-rectus in the right hemisphere generated by parcellation of the white surface using Destrieux (a2009s) parcellation	1.26E-01	1.95E-02
977	27582	aparc-a2009s_rh_area_G-subcallosal	Destrieux Atlas	cortical area	mm2	Area of G-subcallosal in the right hemisphere generated by parcellation of the white surface using Destrieux (a2009s) parcellation	6.31E-02	1.69E-02
978	27583	aparc-a2009s_rh_area_G-temp-sup-G-T-transv	Destrieux Atlas	cortical area	mm2	Area of G-temp-sup-G-T-transv in the right hemisphere generated by parcellation of the white surface using Destrieux (a2009s) parcellation	1.58E-01	1.81E-02
979	27584	aparc-a2009s_rh_area_G-temp-sup-Lateral	Destrieux Atlas	cortical area	mm2	Area of G-temp-sup-Lateral in the right hemisphere generated by parcellation of the white surface using Destrieux (a2009s) parcellation	1.98E-01	3.02E-02
980	27585	aparc-a2009s_rh_area_G-temp-sup-Plan-polar	Destrieux Atlas	cortical area	mm2	Area of G-temp-sup-Plan-polar in the right hemisphere generated by parcellation of the white surface using Destrieux (a2009s) parcellation	9.02E-02	1.53E-02

981	27586	aparc-a2009s_rh_area_G-temp-sup-Plan-tempo	Destrieux Atlas	cortical area	mm2	Area of G-temp-sup-Plan-tempo in the right hemisphere generated by parcellation of the white surface using Destrieux (a2009s) parcellation	1.43E-01	1.81E-02
982	27587	aparc-a2009s_rh_area_G-temporal-inf	Destrieux Atlas	cortical area	mm2	Area of G-temporal-inf in the right hemisphere generated by parcellation of the white surface using Destrieux (a2009s) parcellation	1.83E-01	1.54E-02
983	27588	aparc-a2009s_rh_area_G-temporal-middle	Destrieux Atlas	cortical area	mm2	Area of G-temporal-middle in the right hemisphere generated by parcellation of the white surface using Destrieux (a2009s) parcellation	2.01E-01	2.01E-02
984	27589	aparc-a2009s_rh_area_Lat-Fis-ant-Horizont	Destrieux Atlas	cortical area	mm2	Area of Lat-Fis-ant-Horizont in the right hemisphere generated by parcellation of the white surface using Destrieux (a2009s) parcellation	7.74E-02	1.51E-02
985	27590	aparc-a2009s_rh_area_Lat-Fis-ant-Vertical	Destrieux Atlas	cortical area	mm2	Area of Lat-Fis-ant-Vertical in the right hemisphere generated by parcellation of the white surface using Destrieux (a2009s) parcellation	5.18E-02	1.57E-02
986	27591	aparc-a2009s_rh_area_Lat-Fis-post	Destrieux Atlas	cortical area	mm2	Area of Lat-Fis-post in the right hemisphere generated by parcellation of the white surface using Destrieux (a2009s) parcellation	2.00E-01	2.01E-02
987	27592	aparc-a2009s_rh_area_Pole-occipital	Destrieux Atlas	cortical area	mm2	Area of Pole-occipital in the right hemisphere generated by parcellation of the white surface using Destrieux (a2009s) parcellation	2.76E-01	2.52E-02
988	27593	aparc-a2009s_rh_area_Pole-temporal	Destrieux Atlas	cortical area	mm2	Area of Pole-temporal in the right hemisphere generated by parcellation of the white surface using Destrieux (a2009s) parcellation	2.01E-01	1.77E-02
989	27594	aparc-a2009s_rh_area_S-calcarine	Destrieux Atlas	cortical area	mm2	Area of S-calcarine in the right hemisphere generated by parcellation of the white surface using Destrieux (a2009s) parcellation	3.25E-01	2.96E-02
990	27595	aparc-a2009s_rh_area_S-central	Destrieux Atlas	cortical area	mm2	Area of S-central in the right hemisphere generated by parcellation of the white surface using Destrieux (a2009s) parcellation	2.44E-01	3.23E-02

991	27596	aparc-a2009s_rh_area_S-cingul-Marginalis	Destrieux Atlas	cortical area	mm2	Area of S-cingul-Marginalis in the right hemisphere generated by parcellation of the white surface using Destrieux (a2009s) parcellation	1.55E-01	1.74E-02
992	27597	aparc-a2009s_rh_area_S-circular-insula-ant	Destrieux Atlas	cortical area	mm2	Area of S-circular-insula-ant in the right hemisphere generated by parcellation of the white surface using Destrieux (a2009s) parcellation	1.37E-01	1.74E-02
993	27598	aparc-a2009s_rh_area_S-circular-insula-inf	Destrieux Atlas	cortical area	mm2	Area of S-circular-insula-inf in the right hemisphere generated by parcellation of the white surface using Destrieux (a2009s) parcellation	1.77E-01	2.00E-02
994	27599	aparc-a2009s_rh_area_S-circular-insula-sup	Destrieux Atlas	cortical area	mm2	Area of S-circular-insula-sup in the right hemisphere generated by parcellation of the white surface using Destrieux (a2009s) parcellation	2.02E-01	2.11E-02
995	27600	aparc-a2009s_rh_area_S-collat-transv-ant	Destrieux Atlas	cortical area	mm2	Area of S-collat-transv-ant in the right hemisphere generated by parcellation of the white surface using Destrieux (a2009s) parcellation	2.07E-01	2.02E-02
996	27601	aparc-a2009s_rh_area_S-collat-transv-post	Destrieux Atlas	cortical area	mm2	Area of S-collat-transv-post in the right hemisphere generated by parcellation of the white surface using Destrieux (a2009s) parcellation	9.90E-02	1.60E-02
997	27602	aparc-a2009s_rh_area_S-front-inf	Destrieux Atlas	cortical area	mm2	Area of S-front-inf in the right hemisphere generated by parcellation of the white surface using Destrieux (a2009s) parcellation	1.23E-01	1.79E-02
998	27603	aparc-a2009s_rh_area_S-front-middle	Destrieux Atlas	cortical area	mm2	Area of S-front-middle in the right hemisphere generated by parcellation of the white surface using Destrieux (a2009s) parcellation	1.21E-01	1.61E-02
999	27604	aparc-a2009s_rh_area_S-front-sup	Destrieux Atlas	cortical area	mm2	Area of S-front-sup in the right hemisphere generated by parcellation of the white surface using Destrieux (a2009s) parcellation	1.25E-01	1.74E-02
1000	27605	aparc-a2009s_rh_area_S-interm-prim-Jensen	Destrieux Atlas	cortical area	mm2	Area of S-interm-prim-Jensen in the right hemisphere generated by parcellation of the white surface using Destrieux (a2009s) parcellation	5.85E-02	1.35E-02

1001	27606	aparc-a2009s_rh_area_S-intrapariet+P-trans	Destrieux Atlas	cortical area	mm2	Area of S-intrapariet+P-trans in the right hemisphere generated by parcellation of the white surface using Destrieux (a2009s) parcellation	1.28E-01	1.64E-02
1002	27607	aparc-a2009s_rh_area_S-oc-middle+Lunatus	Destrieux Atlas	cortical area	mm2	Area of S-oc-middle+Lunatus in the right hemisphere generated by parcellation of the white surface using Destrieux (a2009s) parcellation	1.50E-01	1.84E-02
1003	27608	aparc-a2009s_rh_area_S-oc-sup+transversal	Destrieux Atlas	cortical area	mm2	Area of S-oc-sup+transversal in the right hemisphere generated by parcellation of the white surface using Destrieux (a2009s) parcellation	9.55E-02	1.59E-02
1004	27609	aparc-a2009s_rh_area_S-occipital-ant	Destrieux Atlas	cortical area	mm2	Area of S-occipital-ant in the right hemisphere generated by parcellation of the white surface using Destrieux (a2009s) parcellation	1.06E-01	1.82E-02
1005	27610	aparc-a2009s_rh_area_S-oc-temp-lat	Destrieux Atlas	cortical area	mm2	Area of S-oc-temp-lat in the right hemisphere generated by parcellation of the white surface using Destrieux (a2009s) parcellation	1.08E-01	2.26E-02
1006	27611	aparc-a2009s_rh_area_S-oc-temp-med+Lingual	Destrieux Atlas	cortical area	mm2	Area of S-oc-temp-med+Lingual in the right hemisphere generated by parcellation of the white surface using Destrieux (a2009s) parcellation	1.60E-01	1.74E-02
1007	27612	aparc-a2009s_rh_area_S-orbital-lateral	Destrieux Atlas	cortical area	mm2	Area of S-orbital-lateral in the right hemisphere generated by parcellation of the white surface using Destrieux (a2009s) parcellation	1.01E-01	1.44E-02
1008	27613	aparc-a2009s_rh_area_S-orbital-med-olfact	Destrieux Atlas	cortical area	mm2	Area of S-orbital-med-olfact in the right hemisphere generated by parcellation of the white surface using Destrieux (a2009s) parcellation	1.75E-01	1.78E-02
1009	27614	aparc-a2009s_rh_area_S-orbital-H-Shaped	Destrieux Atlas	cortical area	mm2	Area of S-orbital-H-Shaped in the right hemisphere generated by parcellation of the white surface using Destrieux (a2009s) parcellation	2.62E-01	2.16E-02
1010	27615	aparc-a2009s_rh_area_S-parieto-occipital	Destrieux Atlas	cortical area	mm2	Area of S-parieto-occipital in the right hemisphere generated by parcellation of the white surface using Destrieux (a2009s) parcellation	2.29E-01	1.93E-02

1011	27616	aparc-a2009s_rh_area_S-pericallosal	Destrieux Atlas	cortical area	mm2	Area of S-pericallosal in the right hemisphere generated by parcellation of the white surface using Destrieux (a2009s) parcellation	1.35E-01	1.92E-02
1012	27617	aparc-a2009s_rh_area_S-postcentral	Destrieux Atlas	cortical area	mm2	Area of S-postcentral in the right hemisphere generated by parcellation of the white surface using Destrieux (a2009s) parcellation	1.45E-01	1.81E-02
1013	27618	aparc-a2009s_rh_area_S-precentral-inf-part	Destrieux Atlas	cortical area	mm2	Area of S-precentral-inf-part in the right hemisphere generated by parcellation of the white surface using Destrieux (a2009s) parcellation	1.14E-01	1.63E-02
1014	27619	aparc-a2009s_rh_area_S-precentral-sup-part	Destrieux Atlas	cortical area	mm2	Area of S-precentral-sup-part in the right hemisphere generated by parcellation of the white surface using Destrieux (a2009s) parcellation	1.10E-01	1.69E-02
1015	27620	aparc-a2009s_rh_area_S-suborbital	Destrieux Atlas	cortical area	mm2	Area of S-suborbital in the right hemisphere generated by parcellation of the white surface using Destrieux (a2009s) parcellation	4.82E-02	1.58E-02
1016	27621	aparc-a2009s_rh_area_S-subparietal	Destrieux Atlas	cortical area	mm2	Area of S-subparietal in the right hemisphere generated by parcellation of the white surface using Destrieux (a2009s) parcellation	1.40E-01	1.72E-02
1017	27622	aparc-a2009s_rh_area_S-temporal-inf	Destrieux Atlas	cortical area	mm2	Area of S-temporal-inf in the right hemisphere generated by parcellation of the white surface using Destrieux (a2009s) parcellation	1.40E-01	1.82E-02
1018	27623	aparc-a2009s_rh_area_S-temporal-sup	Destrieux Atlas	cortical area	mm2	Area of S-temporal-sup in the right hemisphere generated by parcellation of the white surface using Destrieux (a2009s) parcellation	1.86E-01	1.85E-02
1019	27624	aparc-a2009s_rh_area_S-temporal-transverse	Destrieux Atlas	cortical area	mm2	Area of S-temporal-transverse in the right hemisphere generated by parcellation of the white surface using Destrieux (a2009s) parcellation	1.55E-01	1.81E-02
1020	26755	aparc-Desikan_lh_thickness_GlobalMeanThickness	Desikan Atlas	cortical thickness	mm	Mean thickness of GlobalMeanMean thickness in the left hemisphere generated by parcellation of the white surface using Desikan-Killiany parcellation	2.11E-01	2.11E-02
1021	26756	aparc-Desikan_lh_thickness_bankssts	Desikan Atlas	cortical thickness	mm	Mean thickness of bankssts in the left hemisphere generated by parcellation of the white surface using	1.18E-01	1.83E-02

						Desikan-Killiany parcellation		
1022	26757	aparc-Desikan_lh_thickness_caudalanteriorcingulate	Desikan Atlas	cortical thickness	mm	Mean thickness of caudalanteriorcingulate in the left hemisphere generated by parcellation of the white surface using Desikan-Killiany parcellation	9.20E-02	1.60E-02
1023	26758	aparc-Desikan_lh_thickness_caudalmiddlefrontal	Desikan Atlas	cortical thickness	mm	Mean thickness of caudalmiddlefrontal in the left hemisphere generated by parcellation of the white surface using Desikan-Killiany parcellation	2.03E-01	1.98E-02
1024	26759	aparc-Desikan_lh_thickness_cuneus	Desikan Atlas	cortical thickness	mm	Mean thickness of cuneus in the left hemisphere generated by parcellation of the white surface using Desikan-Killiany parcellation	1.92E-01	1.74E-02
1025	26760	aparc-Desikan_lh_thickness_entorhinal	Desikan Atlas	cortical thickness	mm	Mean thickness of entorhinal in the left hemisphere generated by parcellation of the white surface using Desikan-Killiany parcellation	9.81E-02	1.59E-02
1026	26761	aparc-Desikan_lh_thickness_fusiform	Desikan Atlas	cortical thickness	mm	Mean thickness of fusiform in the left hemisphere generated by parcellation of the white surface using Desikan-Killiany parcellation	1.54E-01	2.04E-02
1027	26762	aparc-Desikan_lh_thickness_inferiorparietal	Desikan Atlas	cortical thickness	mm	Mean thickness of inferiorparietal in the left hemisphere generated by parcellation of the white surface using Desikan-Killiany parcellation	1.99E-01	2.03E-02
1028	26763	aparc-Desikan_lh_thickness_inferiortemporal	Desikan Atlas	cortical thickness	mm	Mean thickness of inferiortemporal in the left hemisphere generated by parcellation of the white surface using Desikan-Killiany parcellation	1.28E-01	1.68E-02
1029	26764	aparc-Desikan_lh_thickness_isthmuscingulate	Desikan Atlas	cortical thickness	mm	Mean thickness of isthmuscingulate in the left hemisphere generated by parcellation of the white surface using Desikan-Killiany parcellation	1.86E-01	1.96E-02
1030	26765	aparc-Desikan_lh_thickness_lateraloccipital	Desikan Atlas	cortical thickness	mm	Mean thickness of lateraloccipital in the left hemisphere generated by parcellation of the white surface using Desikan-Killiany parcellation	1.76E-01	1.87E-02
1031	26766	aparc-Desikan_lh_thickness_lateralorbitofrontal	Desikan Atlas	cortical thickness	mm	Mean thickness of lateralorbitofrontal in the left hemisphere generated by parcellation of the white surface using Desikan-Killiany parcellation	1.19E-01	1.56E-02

1032	26767	aparc-Desikan_lh_thickness_lingual	Desikan Atlas	cortical thickness	mm	Mean thickness of lingual in the left hemisphere generated by parcellation of the white surface using Desikan-Killiany parcellation	1.86E-01	1.94E-02
1033	26768	aparc-Desikan_lh_thickness_medialorbitofrontal	Desikan Atlas	cortical thickness	mm	Mean thickness of medialorbitofrontal in the left hemisphere generated by parcellation of the white surface using Desikan-Killiany parcellation	1.10E-01	1.86E-02
1034	26769	aparc-Desikan_lh_thickness_middletemporal	Desikan Atlas	cortical thickness	mm	Mean thickness of middletemporal in the left hemisphere generated by parcellation of the white surface using Desikan-Killiany parcellation	1.25E-01	1.65E-02
1035	26770	aparc-Desikan_lh_thickness_parahippocampal	Desikan Atlas	cortical thickness	mm	Mean thickness of parahippocampal in the left hemisphere generated by parcellation of the white surface using Desikan-Killiany parcellation	1.83E-01	1.90E-02
1036	26771	aparc-Desikan_lh_thickness_paracentral	Desikan Atlas	cortical thickness	mm	Mean thickness of paracentral in the left hemisphere generated by parcellation of the white surface using Desikan-Killiany parcellation	2.19E-01	2.31E-02
1037	26772	aparc-Desikan_lh_thickness_parsopercularis	Desikan Atlas	cortical thickness	mm	Mean thickness of parsopercularis in the left hemisphere generated by parcellation of the white surface using Desikan-Killiany parcellation	1.69E-01	1.89E-02
1038	26773	aparc-Desikan_lh_thickness_parsorbitalis	Desikan Atlas	cortical thickness	mm	Mean thickness of parsorbitalis in the left hemisphere generated by parcellation of the white surface using Desikan-Killiany parcellation	8.81E-02	1.55E-02
1039	26774	aparc-Desikan_lh_thickness_parstriangularis	Desikan Atlas	cortical thickness	mm	Mean thickness of parstriangularis in the left hemisphere generated by parcellation of the white surface using Desikan-Killiany parcellation	1.51E-01	1.71E-02
1040	26775	aparc-Desikan_lh_thickness_pericalcarine	Desikan Atlas	cortical thickness	mm	Mean thickness of pericalcarine in the left hemisphere generated by parcellation of the white surface using Desikan-Killiany parcellation	1.25E-01	1.64E-02
1041	26776	aparc-Desikan_lh_thickness_postcentral	Desikan Atlas	cortical thickness	mm	Mean thickness of postcentral in the left hemisphere generated by parcellation of the white surface using Desikan-Killiany parcellation	1.83E-01	1.89E-02
1042	26777	aparc-Desikan_lh_thickness_posteriorcingulate	Desikan Atlas	cortical thickness	mm	Mean thickness of posteriorcingulate in the left hemisphere generated by parcellation of the white surface using Desikan-Killiany parcellation	1.31E-01	1.72E-02

1043	26778	aparc-Desikan_lh_thickness_precentral	Desikan Atlas	cortical thickness	mm	Mean thickness of precentral in the left hemisphere generated by parcellation of the white surface using Desikan-Killiany parcellation	2.00E-01	2.24E-02
1044	26779	aparc-Desikan_lh_thickness_precuneus	Desikan Atlas	cortical thickness	mm	Mean thickness of precuneus in the left hemisphere generated by parcellation of the white surface using Desikan-Killiany parcellation	2.07E-01	2.02E-02
1045	26780	aparc-Desikan_lh_thickness_rostralanteriorcingulate	Desikan Atlas	cortical thickness	mm	Mean thickness of rostralanteriorcingulate in the left hemisphere generated by parcellation of the white surface using Desikan-Killiany parcellation	1.25E-01	1.66E-02
1046	26781	aparc-Desikan_lh_thickness_rostralmiddlefrontal	Desikan Atlas	cortical thickness	mm	Mean thickness of rostralmiddlefrontal in the left hemisphere generated by parcellation of the white surface using Desikan-Killiany parcellation	2.06E-01	1.93E-02
1047	26782	aparc-Desikan_lh_thickness_superiorfrontal	Desikan Atlas	cortical thickness	mm	Mean thickness of superiorfrontal in the left hemisphere generated by parcellation of the white surface using Desikan-Killiany parcellation	2.18E-01	2.23E-02
1048	26783	aparc-Desikan_lh_thickness_superiorparietal	Desikan Atlas	cortical thickness	mm	Mean thickness of superiorparietal in the left hemisphere generated by parcellation of the white surface using Desikan-Killiany parcellation	2.09E-01	1.98E-02
1049	26784	aparc-Desikan_lh_thickness_superiortemporal	Desikan Atlas	cortical thickness	mm	Mean thickness of superiortemporal in the left hemisphere generated by parcellation of the white surface using Desikan-Killiany parcellation	1.82E-01	1.92E-02
1050	26785	aparc-Desikan_lh_thickness_supramarginal	Desikan Atlas	cortical thickness	mm	Mean thickness of supramarginal in the left hemisphere generated by parcellation of the white surface using Desikan-Killiany parcellation	2.13E-01	2.52E-02
1051	26786	aparc-Desikan_lh_thickness_frontalpole	Desikan Atlas	cortical thickness	mm	Mean thickness of frontalpole in the left hemisphere generated by parcellation of the white surface using Desikan-Killiany parcellation	7.89E-02	1.48E-02
1052	26787	aparc-Desikan_lh_thickness_transversetemporal	Desikan Atlas	cortical thickness	mm	Mean thickness of transversetemporal in the left hemisphere generated by parcellation of the white surface using Desikan-Killiany parcellation	1.75E-01	1.94E-02
1053	26788	aparc-Desikan_lh_thickness_insula	Desikan Atlas	cortical thickness	mm	Mean thickness of insula in the left hemisphere generated by parcellation of the white surface using Desikan-Killiany parcellation	1.35E-01	1.94E-02

1054	26856	aparc-Desikan_rh_thickness_GlobalMeanThickness	Desikan Atlas	cortical thickness	mm	Mean thickness of GlobalMeanMean thickness in the right hemisphere generated by parcellation of the white surface using Desikan-Killiany parcellation	2.23E-01	2.29E-02
1055	26857	aparc-Desikan_rh_thickness_bankssts	Desikan Atlas	cortical thickness	mm	Mean thickness of bankssts in the right hemisphere generated by parcellation of the white surface using Desikan-Killiany parcellation	1.38E-01	1.84E-02
1056	26858	aparc-Desikan_rh_thickness_caudalanteriorcingulate	Desikan Atlas	cortical thickness	mm	Mean thickness of caudalanteriorcingulate in the right hemisphere generated by parcellation of the white surface using Desikan-Killiany parcellation	1.06E-01	1.61E-02
1057	26859	aparc-Desikan_rh_thickness_caudalmiddlefrontal	Desikan Atlas	cortical thickness	mm	Mean thickness of caudalmiddlefrontal in the right hemisphere generated by parcellation of the white surface using Desikan-Killiany parcellation	1.83E-01	1.94E-02
1058	26860	aparc-Desikan_rh_thickness_cuneus	Desikan Atlas	cortical thickness	mm	Mean thickness of cuneus in the right hemisphere generated by parcellation of the white surface using Desikan-Killiany parcellation	1.77E-01	1.87E-02
1059	26861	aparc-Desikan_rh_thickness_entorhinal	Desikan Atlas	cortical thickness	mm	Mean thickness of entorhinal in the right hemisphere generated by parcellation of the white surface using Desikan-Killiany parcellation	6.42E-02	1.62E-02
1060	26862	aparc-Desikan_rh_thickness_fusiform	Desikan Atlas	cortical thickness	mm	Mean thickness of fusiform in the right hemisphere generated by parcellation of the white surface using Desikan-Killiany parcellation	1.56E-01	2.03E-02
1061	26863	aparc-Desikan_rh_thickness_inferiorparietal	Desikan Atlas	cortical thickness	mm	Mean thickness of inferiorparietal in the right hemisphere generated by parcellation of the white surface using Desikan-Killiany parcellation	2.13E-01	2.23E-02
1062	26864	aparc-Desikan_rh_thickness_inferiortemporal	Desikan Atlas	cortical thickness	mm	Mean thickness of inferiortemporal in the right hemisphere generated by parcellation of the white surface using Desikan-Killiany parcellation	1.58E-01	1.68E-02
1063	26865	aparc-Desikan_rh_thickness_isthmuscingulate	Desikan Atlas	cortical thickness	mm	Mean thickness of isthmuscingulate in the right hemisphere generated by parcellation of the white surface using Desikan-Killiany parcellation	1.88E-01	1.83E-02
1064	26866	aparc-Desikan_rh_thickness_lateraloccipital	Desikan Atlas	cortical thickness	mm	Mean thickness of lateraloccipital in the right hemisphere generated by parcellation of the white surface using	2.14E-01	2.06E-02

						Desikan-Killiany parcellation		
1065	26867	aparc-Desikan_rh_thickness_lateralorbitofrontal	Desikan Atlas	cortical thickness	mm	Mean thickness of lateralorbitofrontal in the right hemisphere generated by parcellation of the white surface using Desikan-Killiany parcellation	1.15E-01	1.60E-02
1066	26868	aparc-Desikan_rh_thickness_lingual	Desikan Atlas	cortical thickness	mm	Mean thickness of lingual in the right hemisphere generated by parcellation of the white surface using Desikan-Killiany parcellation	1.90E-01	1.98E-02
1067	26869	aparc-Desikan_rh_thickness_medialorbitofrontal	Desikan Atlas	cortical thickness	mm	Mean thickness of medialorbitofrontal in the right hemisphere generated by parcellation of the white surface using Desikan-Killiany parcellation	1.04E-01	1.44E-02
1068	26870	aparc-Desikan_rh_thickness_middletemporal	Desikan Atlas	cortical thickness	mm	Mean thickness of middletemporal in the right hemisphere generated by parcellation of the white surface using Desikan-Killiany parcellation	1.52E-01	1.68E-02
1069	26871	aparc-Desikan_rh_thickness_parahippocampal	Desikan Atlas	cortical thickness	mm	Mean thickness of parahippocampal in the right hemisphere generated by parcellation of the white surface using Desikan-Killiany parcellation	1.62E-01	1.79E-02
1070	26872	aparc-Desikan_rh_thickness_paracentral	Desikan Atlas	cortical thickness	mm	Mean thickness of paracentral in the right hemisphere generated by parcellation of the white surface using Desikan-Killiany parcellation	1.99E-01	2.39E-02
1071	26873	aparc-Desikan_rh_thickness_parsopercularis	Desikan Atlas	cortical thickness	mm	Mean thickness of parsopercularis in the right hemisphere generated by parcellation of the white surface using Desikan-Killiany parcellation	1.42E-01	1.78E-02
1072	26874	aparc-Desikan_rh_thickness_parsorbitalis	Desikan Atlas	cortical thickness	mm	Mean thickness of parsorbitalis in the right hemisphere generated by parcellation of the white surface using Desikan-Killiany parcellation	9.14E-02	1.71E-02
1073	26875	aparc-Desikan_rh_thickness_parstriangularis	Desikan Atlas	cortical thickness	mm	Mean thickness of parstriangularis in the right hemisphere generated by parcellation of the white surface using Desikan-Killiany parcellation	1.77E-01	1.83E-02
1074	26876	aparc-Desikan_rh_thickness_pericalcarine	Desikan Atlas	cortical thickness	mm	Mean thickness of pericalcarine in the right hemisphere generated by parcellation of the white surface using Desikan-Killiany parcellation	1.23E-01	1.80E-02

1075	26877	aparc-Desikan_rh_thickness_postcentral	Desikan Atlas	cortical thickness	mm	Mean thickness of postcentral in the right hemisphere generated by parcellation of the white surface using Desikan-Killiany parcellation	1.68E-01	2.06E-02
1076	26878	aparc-Desikan_rh_thickness_posteriorcingulate	Desikan Atlas	cortical thickness	mm	Mean thickness of posteriorcingulate in the right hemisphere generated by parcellation of the white surface using Desikan-Killiany parcellation	1.57E-01	1.95E-02
1077	26879	aparc-Desikan_rh_thickness_precentral	Desikan Atlas	cortical thickness	mm	Mean thickness of precentral in the right hemisphere generated by parcellation of the white surface using Desikan-Killiany parcellation	2.03E-01	2.10E-02
1078	26880	aparc-Desikan_rh_thickness_precuneus	Desikan Atlas	cortical thickness	mm	Mean thickness of precuneus in the right hemisphere generated by parcellation of the white surface using Desikan-Killiany parcellation	2.12E-01	2.37E-02
1079	26881	aparc-Desikan_rh_thickness_rostralanteriorcingulate	Desikan Atlas	cortical thickness	mm	Mean thickness of rostralanteriorcingulate in the right hemisphere generated by parcellation of the white surface using Desikan-Killiany parcellation	8.06E-02	1.48E-02
1080	26882	aparc-Desikan_rh_thickness_rostralmiddlefrontal	Desikan Atlas	cortical thickness	mm	Mean thickness of rostralmiddlefrontal in the right hemisphere generated by parcellation of the white surface using Desikan-Killiany parcellation	1.81E-01	1.95E-02
1081	26883	aparc-Desikan_rh_thickness_superiorfrontal	Desikan Atlas	cortical thickness	mm	Mean thickness of superiorfrontal in the right hemisphere generated by parcellation of the white surface using Desikan-Killiany parcellation	2.28E-01	2.09E-02
1082	26884	aparc-Desikan_rh_thickness_superiorparietal	Desikan Atlas	cortical thickness	mm	Mean thickness of superiorparietal in the right hemisphere generated by parcellation of the white surface using Desikan-Killiany parcellation	1.66E-01	2.05E-02
1083	26885	aparc-Desikan_rh_thickness_superiortemporal	Desikan Atlas	cortical thickness	mm	Mean thickness of superiortemporal in the right hemisphere generated by parcellation of the white surface using Desikan-Killiany parcellation	2.05E-01	2.04E-02
1084	26886	aparc-Desikan_rh_thickness_supramarginal	Desikan Atlas	cortical thickness	mm	Mean thickness of supramarginal in the right hemisphere generated by parcellation of the white surface using Desikan-Killiany parcellation	2.07E-01	2.27E-02
1085	26887	aparc-Desikan_rh_thickness_frontalpole	Desikan Atlas	cortical thickness	mm	Mean thickness of frontalpole in the right hemisphere generated by parcellation of the white surface using Desikan-Killiany parcellation	6.66E-02	1.49E-02

1086	26888	aparc-Desikan_rh_thickness_transversetemporal	Desikan Atlas	cortical thickness	mm	Mean thickness of transversetemporal in the right hemisphere generated by parcellation of the white surface using Desikan-Killiany parcellation	1.53E-01	2.21E-02
1087	26889	aparc-Desikan_rh_thickness_insula	Desikan Atlas	cortical thickness	mm	Mean thickness of insula in the right hemisphere generated by parcellation of the white surface using Desikan-Killiany parcellation	1.43E-01	2.49E-02
1088	27073	BA-exvivo_lh_thickness_BA1	Broadmann Atlas	cortical thickness	mm	Mean thickness of BA1 in the left hemisphere generated by parcellation of the white surface using BA_exvivo parcellation	1.75E-01	1.88E-02
1089	27074	BA-exvivo_lh_thickness_BA2	Broadmann Atlas	cortical thickness	mm	Mean thickness of BA2 in the left hemisphere generated by parcellation of the white surface using BA_exvivo parcellation	1.73E-01	1.88E-02
1090	27075	BA-exvivo_lh_thickness_BA3a	Broadmann Atlas	cortical thickness	mm	Mean thickness of BA3a in the left hemisphere generated by parcellation of the white surface using BA_exvivo parcellation	1.57E-01	1.95E-02
1091	27076	BA-exvivo_lh_thickness_BA3b	Broadmann Atlas	cortical thickness	mm	Mean thickness of BA3b in the left hemisphere generated by parcellation of the white surface using BA_exvivo parcellation	1.39E-01	1.68E-02
1092	27077	BA-exvivo_lh_thickness_BA4a	Broadmann Atlas	cortical thickness	mm	Mean thickness of BA4a in the left hemisphere generated by parcellation of the white surface using BA_exvivo parcellation	1.98E-01	1.87E-02
1093	27078	BA-exvivo_lh_thickness_BA4p	Broadmann Atlas	cortical thickness	mm	Mean thickness of BA4p in the left hemisphere generated by parcellation of the white surface using BA_exvivo parcellation	1.53E-01	1.93E-02
1094	27079	BA-exvivo_lh_thickness_BA6	Broadmann Atlas	cortical thickness	mm	Mean thickness of BA6 in the left hemisphere generated by parcellation of the white surface using BA_exvivo parcellation	2.29E-01	2.32E-02
1095	27080	BA-exvivo_lh_thickness_BA44	Broadmann Atlas	cortical thickness	mm	Mean thickness of BA44 in the left hemisphere generated by parcellation of the white surface using BA_exvivo parcellation	1.67E-01	1.91E-02
1096	27081	BA-exvivo_lh_thickness_BA45	Broadmann Atlas	cortical thickness	mm	Mean thickness of BA45 in the left hemisphere generated by parcellation of the white surface using BA_exvivo parcellation	1.74E-01	1.75E-02

1097	27082	BA-exvivo_lh_thickness_V1	Broadmann Atlas	cortical thickness	mm	Mean thickness of V1 in the left hemisphere generated by parcellation of the white surface using BA_exvivo parcellation	1.33E-01	1.57E-02
1098	27083	BA-exvivo_lh_thickness_V2	Broadmann Atlas	cortical thickness	mm	Mean thickness of V2 in the left hemisphere generated by parcellation of the white surface using BA_exvivo parcellation	1.99E-01	1.87E-02
1099	27084	BA-exvivo_lh_thickness_MT	Broadmann Atlas	cortical thickness	mm	Mean thickness of MT in the left hemisphere generated by parcellation of the white surface using BA_exvivo parcellation	1.48E-01	1.84E-02
1100	27085	BA-exvivo_lh_thickness_perirhinal	Broadmann Atlas	cortical thickness	mm	Mean thickness of perirhinal in the left hemisphere generated by parcellation of the white surface using BA_exvivo parcellation	7.81E-02	1.52E-02
1101	27086	BA-exvivo_lh_thickness_entorhinal	Broadmann Atlas	cortical thickness	mm	Mean thickness of entorhinal in the left hemisphere generated by parcellation of the white surface using BA_exvivo parcellation	1.25E-01	1.71E-02
1102	27115	BA-exvivo_rh_thickness_BA1	Broadmann Atlas	cortical thickness	mm	Mean thickness of BA1 in the right hemisphere generated by parcellation of the white surface using BA_exvivo parcellation	1.54E-01	1.87E-02
1103	27116	BA-exvivo_rh_thickness_BA2	Broadmann Atlas	cortical thickness	mm	Mean thickness of BA2 in the right hemisphere generated by parcellation of the white surface using BA_exvivo parcellation	1.61E-01	2.07E-02
1104	27117	BA-exvivo_rh_thickness_BA3a	Broadmann Atlas	cortical thickness	mm	Mean thickness of BA3a in the right hemisphere generated by parcellation of the white surface using BA_exvivo parcellation	1.28E-01	2.05E-02
1105	27118	BA-exvivo_rh_thickness_BA3b	Broadmann Atlas	cortical thickness	mm	Mean thickness of BA3b in the right hemisphere generated by parcellation of the white surface using BA_exvivo parcellation	1.13E-01	1.76E-02
1106	27119	BA-exvivo_rh_thickness_BA4a	Broadmann Atlas	cortical thickness	mm	Mean thickness of BA4a in the right hemisphere generated by parcellation of the white surface using BA_exvivo parcellation	1.79E-01	2.04E-02
1107	27120	BA-exvivo_rh_thickness_BA4p	Broadmann Atlas	cortical thickness	mm	Mean thickness of BA4p in the right hemisphere generated by parcellation of the white surface using BA_exvivo parcellation	1.59E-01	1.87E-02

1108	27121	BA-exvivo_rh_thickness_BA6	Broadmann Atlas	cortical thickness	mm	Mean thickness of BA6 in the right hemisphere generated by parcellation of the white surface using BA_exvivo parcellation	2.30E-01	2.26E-02
1109	27122	BA-exvivo_rh_thickness_BA44	Broadmann Atlas	cortical thickness	mm	Mean thickness of BA44 in the right hemisphere generated by parcellation of the white surface using BA_exvivo parcellation	1.79E-01	2.01E-02
1110	27123	BA-exvivo_rh_thickness_BA45	Broadmann Atlas	cortical thickness	mm	Mean thickness of BA45 in the right hemisphere generated by parcellation of the white surface using BA_exvivo parcellation	1.96E-01	1.92E-02
1111	27124	BA-exvivo_rh_thickness_V1	Broadmann Atlas	cortical thickness	mm	Mean thickness of V1 in the right hemisphere generated by parcellation of the white surface using BA_exvivo parcellation	1.21E-01	1.70E-02
1112	27125	BA-exvivo_rh_thickness_V2	Broadmann Atlas	cortical thickness	mm	Mean thickness of V2 in the right hemisphere generated by parcellation of the white surface using BA_exvivo parcellation	2.26E-01	1.99E-02
1113	27126	BA-exvivo_rh_thickness_MT	Broadmann Atlas	cortical thickness	mm	Mean thickness of MT in the right hemisphere generated by parcellation of the white surface using BA_exvivo parcellation	1.68E-01	1.98E-02
1114	27127	BA-exvivo_rh_thickness_perirhinal	Broadmann Atlas	cortical thickness	mm	Mean thickness of perirhinal in the right hemisphere generated by parcellation of the white surface using BA_exvivo parcellation	6.68E-02	1.58E-02
1115	27128	BA-exvivo_rh_thickness_entorhinal	Broadmann Atlas	cortical thickness	mm	Mean thickness of entorhinal in the right hemisphere generated by parcellation of the white surface using BA_exvivo parcellation	1.10E-01	1.87E-02
1116	27174	aparc-DKTatlas_lh_thickness_caudalanteriorcingulate	Desikan Atlas	cortical thickness	mm	Mean thickness of caudalanteriorcingulate in the left hemisphere generated by parcellation of the white surface using DKT parcellation	9.95E-02	1.56E-02
1117	27175	aparc-DKTatlas_lh_thickness_caudalmiddlefrontal	Desikan Atlas	cortical thickness	mm	Mean thickness of caudalmiddlefrontal in the left hemisphere generated by parcellation of the white surface using DKT parcellation	2.04E-01	1.97E-02
1118	27176	aparc-DKTatlas_lh_thickness_cuneus	Desikan Atlas	cortical thickness	mm	Mean thickness of cuneus in the left hemisphere generated by parcellation of the white surface using DKT parcellation	1.90E-01	1.73E-02

1119	27177	aparc-DKTatlas_lh_thickness_entorhinal	Desikan Atlas	cortical thickness	mm	Mean thickness of entorhinal in the left hemisphere generated by parcellation of the white surface using DKT parcellation	1.02E-01	1.58E-02
1120	27178	aparc-DKTatlas_lh_thickness_fusiform	Desikan Atlas	cortical thickness	mm	Mean thickness of fusiform in the left hemisphere generated by parcellation of the white surface using DKT parcellation	1.56E-01	2.03E-02
1121	27179	aparc-DKTatlas_lh_thickness_inferiorparietal	Desikan Atlas	cortical thickness	mm	Mean thickness of inferiorparietal in the left hemisphere generated by parcellation of the white surface using DKT parcellation	2.01E-01	2.04E-02
1122	27180	aparc-DKTatlas_lh_thickness_inferiortemporal	Desikan Atlas	cortical thickness	mm	Mean thickness of inferiortemporal in the left hemisphere generated by parcellation of the white surface using DKT parcellation	1.14E-01	1.68E-02
1123	27181	aparc-DKTatlas_lh_thickness_isthmuscingulate	Desikan Atlas	cortical thickness	mm	Mean thickness of isthmuscingulate in the left hemisphere generated by parcellation of the white surface using DKT parcellation	1.85E-01	1.99E-02
1124	27182	aparc-DKTatlas_lh_thickness_lateraloccipital	Desikan Atlas	cortical thickness	mm	Mean thickness of lateraloccipital in the left hemisphere generated by parcellation of the white surface using DKT parcellation	1.79E-01	1.88E-02
1125	27183	aparc-DKTatlas_lh_thickness_lateralorbitofrontal	Desikan Atlas	cortical thickness	mm	Mean thickness of lateralorbitofrontal in the left hemisphere generated by parcellation of the white surface using DKT parcellation	1.10E-01	1.53E-02
1126	27184	aparc-DKTatlas_lh_thickness_lingual	Desikan Atlas	cortical thickness	mm	Mean thickness of lingual in the left hemisphere generated by parcellation of the white surface using DKT parcellation	1.86E-01	1.95E-02
1127	27185	aparc-DKTatlas_lh_thickness_medialorbitofrontal	Desikan Atlas	cortical thickness	mm	Mean thickness of medialorbitofrontal in the left hemisphere generated by parcellation of the white surface using DKT parcellation	1.10E-01	2.27E-02
1128	27186	aparc-DKTatlas_lh_thickness_middletemporal	Desikan Atlas	cortical thickness	mm	Mean thickness of middletemporal in the left hemisphere generated by parcellation of the white surface using DKT parcellation	1.36E-01	1.67E-02
1129	27187	aparc-DKTatlas_lh_thickness_parahippocampal	Desikan Atlas	cortical thickness	mm	Mean thickness of parahippocampal in the left hemisphere generated by parcellation of the white surface using DKT parcellation	1.83E-01	1.91E-02

1130	27188	aparc-DKTatlas_lh_thickness_paracentral	Desikan Atlas	cortical thickness	mm	Mean thickness of paracentral in the left hemisphere generated by parcellation of the white surface using DKT parcellation	2.22E-01	2.41E-02
1131	27189	aparc-DKTatlas_lh_thickness_parsopercularis	Desikan Atlas	cortical thickness	mm	Mean thickness of parsopercularis in the left hemisphere generated by parcellation of the white surface using DKT parcellation	1.64E-01	1.86E-02
1132	27190	aparc-DKTatlas_lh_thickness_parsorbitalis	Desikan Atlas	cortical thickness	mm	Mean thickness of parsorbitalis in the left hemisphere generated by parcellation of the white surface using DKT parcellation	1.13E-01	1.71E-02
1133	27191	aparc-DKTatlas_lh_thickness_parstriangularis	Desikan Atlas	cortical thickness	mm	Mean thickness of parstriangularis in the left hemisphere generated by parcellation of the white surface using DKT parcellation	1.58E-01	1.79E-02
1134	27192	aparc-DKTatlas_lh_thickness_pericalcarine	Desikan Atlas	cortical thickness	mm	Mean thickness of pericalcarine in the left hemisphere generated by parcellation of the white surface using DKT parcellation	1.26E-01	1.64E-02
1135	27193	aparc-DKTatlas_lh_thickness_postcentral	Desikan Atlas	cortical thickness	mm	Mean thickness of postcentral in the left hemisphere generated by parcellation of the white surface using DKT parcellation	1.84E-01	1.92E-02
1136	27194	aparc-DKTatlas_lh_thickness_posteriorcingulate	Desikan Atlas	cortical thickness	mm	Mean thickness of posteriorcingulate in the left hemisphere generated by parcellation of the white surface using DKT parcellation	1.46E-01	1.79E-02
1137	27195	aparc-DKTatlas_lh_thickness_precentral	Desikan Atlas	cortical thickness	mm	Mean thickness of precentral in the left hemisphere generated by parcellation of the white surface using DKT parcellation	1.99E-01	2.23E-02
1138	27196	aparc-DKTatlas_lh_thickness_precuneus	Desikan Atlas	cortical thickness	mm	Mean thickness of precuneus in the left hemisphere generated by parcellation of the white surface using DKT parcellation	2.08E-01	2.02E-02
1139	27197	aparc-DKTatlas_lh_thickness_rostralanteriorcingulate	Desikan Atlas	cortical thickness	mm	Mean thickness of rostralanteriorcingulate in the left hemisphere generated by parcellation of the white surface using DKT parcellation	1.43E-01	1.68E-02
1140	27198	aparc-DKTatlas_lh_thickness_rostralmiddlefrontal	Desikan Atlas	cortical thickness	mm	Mean thickness of rostralmiddlefrontal in the left hemisphere generated by parcellation of the white surface using DKT parcellation	2.01E-01	1.90E-02

1141	27199	aparc-DKTatlas_lh_thickness_superiorfrontal	Desikan Atlas	cortical thickness	mm	Mean thickness of superiorfrontal in the left hemisphere generated by parcellation of the white surface using DKT parcellation	2.15E-01	2.22E-02
1142	27200	aparc-DKTatlas_lh_thickness_superiorparietal	Desikan Atlas	cortical thickness	mm	Mean thickness of superiorparietal in the left hemisphere generated by parcellation of the white surface using DKT parcellation	2.07E-01	1.96E-02
1143	27201	aparc-DKTatlas_lh_thickness_superiortemporal	Desikan Atlas	cortical thickness	mm	Mean thickness of superiortemporal in the left hemisphere generated by parcellation of the white surface using DKT parcellation	1.71E-01	1.89E-02
1144	27202	aparc-DKTatlas_lh_thickness_supramarginal	Desikan Atlas	cortical thickness	mm	Mean thickness of supramarginal in the left hemisphere generated by parcellation of the white surface using DKT parcellation	2.09E-01	2.44E-02
1145	27203	aparc-DKTatlas_lh_thickness_transversetemporal	Desikan Atlas	cortical thickness	mm	Mean thickness of transversetemporal in the left hemisphere generated by parcellation of the white surface using DKT parcellation	1.76E-01	1.96E-02
1146	27204	aparc-DKTatlas_lh_thickness_insula	Desikan Atlas	cortical thickness	mm	Mean thickness of insula in the left hemisphere generated by parcellation of the white surface using DKT parcellation	1.50E-01	1.85E-02
1147	27267	aparc-DKTatlas_rh_thickness_caudalanteriorcingulate	Desikan Atlas	cortical thickness	mm	Mean thickness of caudalanteriorcingulate in the right hemisphere generated by parcellation of the white surface using DKT parcellation	1.09E-01	1.62E-02
1148	27268	aparc-DKTatlas_rh_thickness_caudalmiddlefrontal	Desikan Atlas	cortical thickness	mm	Mean thickness of caudalmiddlefrontal in the right hemisphere generated by parcellation of the white surface using DKT parcellation	1.87E-01	1.94E-02
1149	27269	aparc-DKTatlas_rh_thickness_cuneus	Desikan Atlas	cortical thickness	mm	Mean thickness of cuneus in the right hemisphere generated by parcellation of the white surface using DKT parcellation	1.81E-01	1.92E-02
1150	27270	aparc-DKTatlas_rh_thickness_entorhinal	Desikan Atlas	cortical thickness	mm	Mean thickness of entorhinal in the right hemisphere generated by parcellation of the white surface using DKT parcellation	7.22E-02	1.60E-02
1151	27271	aparc-DKTatlas_rh_thickness_fusiform	Desikan Atlas	cortical thickness	mm	Mean thickness of fusiform in the right hemisphere generated by parcellation of the white surface using DKT parcellation	1.59E-01	2.17E-02

1152	27272	aparc-DKTatlas_rh_thickness_inferiorparietal	Desikan Atlas	cortical thickness	mm	Mean thickness of inferiorparietal in the right hemisphere generated by parcellation of the white surface using DKT parcellation	2.13E-01	2.21E-02
1153	27273	aparc-DKTatlas_rh_thickness_inferiortemporal	Desikan Atlas	cortical thickness	mm	Mean thickness of inferiortemporal in the right hemisphere generated by parcellation of the white surface using DKT parcellation	1.60E-01	1.67E-02
1154	27274	aparc-DKTatlas_rh_thickness_isthmuscingulate	Desikan Atlas	cortical thickness	mm	Mean thickness of isthmuscingulate in the right hemisphere generated by parcellation of the white surface using DKT parcellation	1.82E-01	1.82E-02
1155	27275	aparc-DKTatlas_rh_thickness_lateraloccipital	Desikan Atlas	cortical thickness	mm	Mean thickness of lateraloccipital in the right hemisphere generated by parcellation of the white surface using DKT parcellation	2.16E-01	2.05E-02
1156	27276	aparc-DKTatlas_rh_thickness_lateralorbitofrontal	Desikan Atlas	cortical thickness	mm	Mean thickness of lateralorbitofrontal in the right hemisphere generated by parcellation of the white surface using DKT parcellation	1.15E-01	1.69E-02
1157	27277	aparc-DKTatlas_rh_thickness_lingual	Desikan Atlas	cortical thickness	mm	Mean thickness of lingual in the right hemisphere generated by parcellation of the white surface using DKT parcellation	1.91E-01	1.98E-02
1158	27278	aparc-DKTatlas_rh_thickness_medialorbitofrontal	Desikan Atlas	cortical thickness	mm	Mean thickness of medialorbitofrontal in the right hemisphere generated by parcellation of the white surface using DKT parcellation	8.37E-02	1.48E-02
1159	27279	aparc-DKTatlas_rh_thickness_middletemporal	Desikan Atlas	cortical thickness	mm	Mean thickness of middletemporal in the right hemisphere generated by parcellation of the white surface using DKT parcellation	1.69E-01	1.74E-02
1160	27280	aparc-DKTatlas_rh_thickness_parahippocampal	Desikan Atlas	cortical thickness	mm	Mean thickness of parahippocampal in the right hemisphere generated by parcellation of the white surface using DKT parcellation	1.62E-01	1.75E-02
1161	27281	aparc-DKTatlas_rh_thickness_paracentral	Desikan Atlas	cortical thickness	mm	Mean thickness of paracentral in the right hemisphere generated by parcellation of the white surface using DKT parcellation	2.04E-01	2.49E-02
1162	27282	aparc-DKTatlas_rh_thickness_parsopercularis	Desikan Atlas	cortical thickness	mm	Mean thickness of parsopercularis in the right hemisphere generated by parcellation of the white surface using DKT parcellation	1.52E-01	1.78E-02

1163	27283	aparc-DKTatlas_rh_thickness_parsorbitalis	Desikan Atlas	cortical thickness	mm	Mean thickness of parsorbitalis in the right hemisphere generated by parcellation of the white surface using DKT parcellation	1.30E-01	1.73E-02
1164	27284	aparc-DKTatlas_rh_thickness_parstriangularis	Desikan Atlas	cortical thickness	mm	Mean thickness of parstriangularis in the right hemisphere generated by parcellation of the white surface using DKT parcellation	1.64E-01	1.84E-02
1165	27285	aparc-DKTatlas_rh_thickness_pericalcarine	Desikan Atlas	cortical thickness	mm	Mean thickness of pericalcarine in the right hemisphere generated by parcellation of the white surface using DKT parcellation	1.22E-01	1.80E-02
1166	27286	aparc-DKTatlas_rh_thickness_postcentral	Desikan Atlas	cortical thickness	mm	Mean thickness of postcentral in the right hemisphere generated by parcellation of the white surface using DKT parcellation	1.66E-01	2.05E-02
1167	27287	aparc-DKTatlas_rh_thickness_posteriorcingulate	Desikan Atlas	cortical thickness	mm	Mean thickness of posteriorcingulate in the right hemisphere generated by parcellation of the white surface using DKT parcellation	1.60E-01	1.99E-02
1168	27288	aparc-DKTatlas_rh_thickness_precentral	Desikan Atlas	cortical thickness	mm	Mean thickness of precentral in the right hemisphere generated by parcellation of the white surface using DKT parcellation	2.02E-01	2.14E-02
1169	27289	aparc-DKTatlas_rh_thickness_precuneus	Desikan Atlas	cortical thickness	mm	Mean thickness of precuneus in the right hemisphere generated by parcellation of the white surface using DKT parcellation	2.12E-01	2.34E-02
1170	27290	aparc-DKTatlas_rh_thickness_rostralanteriorcingulate	Desikan Atlas	cortical thickness	mm	Mean thickness of rostralanteriorcingulate in the right hemisphere generated by parcellation of the white surface using DKT parcellation	9.05E-02	1.59E-02
1171	27291	aparc-DKTatlas_rh_thickness_rostralmiddlefrontal	Desikan Atlas	cortical thickness	mm	Mean thickness of rostralmiddlefrontal in the right hemisphere generated by parcellation of the white surface using DKT parcellation	1.75E-01	1.86E-02
1172	27292	aparc-DKTatlas_rh_thickness_superiorfrontal	Desikan Atlas	cortical thickness	mm	Mean thickness of superiorfrontal in the right hemisphere generated by parcellation of the white surface using DKT parcellation	2.24E-01	2.01E-02
1173	27293	aparc-DKTatlas_rh_thickness_superiorparietal	Desikan Atlas	cortical thickness	mm	Mean thickness of superiorparietal in the right hemisphere generated by parcellation of the white surface using DKT parcellation	1.62E-01	2.05E-02

1174	27294	aparc-DKTatlas_rh_thickness_superiortemporal	Desikan Atlas	cortical thickness	mm	Mean thickness of superiortemporal in the right hemisphere generated by parcellation of the white surface using DKT parcellation	1.95E-01	1.98E-02
1175	27295	aparc-DKTatlas_rh_thickness_supramarginal	Desikan Atlas	cortical thickness	mm	Mean thickness of supramarginal in the right hemisphere generated by parcellation of the white surface using DKT parcellation	2.05E-01	2.27E-02
1176	27296	aparc-DKTatlas_rh_thickness_transversetemporal	Desikan Atlas	cortical thickness	mm	Mean thickness of transversetemporal in the right hemisphere generated by parcellation of the white surface using DKT parcellation	1.52E-01	2.11E-02
1177	27297	aparc-DKTatlas_rh_thickness_insula	Desikan Atlas	cortical thickness	mm	Mean thickness of insula in the right hemisphere generated by parcellation of the white surface using DKT parcellation	1.89E-01	2.23E-02
1178	27403	aparc-a2009s_lh_thickness_G+S-frontomargin	Destrieux Atlas	cortical thickness	mm	Mean thickness of G+S-frontomargin in the left hemisphere generated by parcellation of the white surface using Destrieux (a2009s) parcellation	1.08E-01	1.56E-02
1179	27404	aparc-a2009s_lh_thickness_G+S-occipital-inf	Destrieux Atlas	cortical thickness	mm	Mean thickness of G+S-occipital-inf in the left hemisphere generated by parcellation of the white surface using Destrieux (a2009s) parcellation	1.46E-01	1.86E-02
1180	27405	aparc-a2009s_lh_thickness_G+S-paracentral	Destrieux Atlas	cortical thickness	mm	Mean thickness of G+S-paracentral in the left hemisphere generated by parcellation of the white surface using Destrieux (a2009s) parcellation	1.80E-01	1.75E-02
1181	27406	aparc-a2009s_lh_thickness_G+S-subcentral	Destrieux Atlas	cortical thickness	mm	Mean thickness of G+S-subcentral in the left hemisphere generated by parcellation of the white surface using Destrieux (a2009s) parcellation	2.10E-01	2.05E-02
1182	27407	aparc-a2009s_lh_thickness_G+S-transv-frontopol	Destrieux Atlas	cortical thickness	mm	Mean thickness of G+S-transv-frontopol in the left hemisphere generated by parcellation of the white surface using Destrieux (a2009s) parcellation	1.12E-01	1.65E-02
1183	27408	aparc-a2009s_lh_thickness_G+S-cingul-Ant	Destrieux Atlas	cortical thickness	mm	Mean thickness of G+S-cingul-Ant in the left hemisphere generated by parcellation of the white surface using Destrieux (a2009s) parcellation	1.32E-01	1.83E-02

1184	27409	aparc-a2009s_lh_thickness_G+S-cingul-Mid-Ant	Destrieux Atlas	cortical thickness	mm	Mean thickness of G+S-cingul-Mid-Ant in the left hemisphere generated by parcellation of the white surface using Destrieux (a2009s) parcellation	7.28E-02	1.69E-02
1185	27410	aparc-a2009s_lh_thickness_G+S-cingul-Mid-Pos t	Destrieux Atlas	cortical thickness	mm	Mean thickness of G+S-cingul-Mid-Post in the left hemisphere generated by parcellation of the white surface using Destrieux (a2009s) parcellation	1.41E-01	1.77E-02
1186	27411	aparc-a2009s_lh_thickness_G-cingul-Post-dorsal	Destrieux Atlas	cortical thickness	mm	Mean thickness of G-cingul-Post-dorsal in the left hemisphere generated by parcellation of the white surface using Destrieux (a2009s) parcellation	1.39E-01	1.71E-02
1187	27412	aparc-a2009s_lh_thickness_G-cingul-Post-ventra l	Destrieux Atlas	cortical thickness	mm	Mean thickness of G-cingul-Post-ventral in the left hemisphere generated by parcellation of the white surface using Destrieux (a2009s) parcellation	1.19E-01	1.70E-02
1188	27413	aparc-a2009s_lh_thickness_G-cuneus	Destrieux Atlas	cortical thickness	mm	Mean thickness of G-cuneus in the left hemisphere generated by parcellation of the white surface using Destrieux (a2009s) parcellation	1.90E-01	1.76E-02
1189	27414	aparc-a2009s_lh_thickness_G-front-inf-Opercula r	Destrieux Atlas	cortical thickness	mm	Mean thickness of G-front-inf-Opercular in the left hemisphere generated by parcellation of the white surface using Destrieux (a2009s) parcellation	1.51E-01	1.82E-02
1190	27415	aparc-a2009s_lh_thickness_G-front-inf-Orbital	Destrieux Atlas	cortical thickness	mm	Mean thickness of G-front-inf-Orbital in the left hemisphere generated by parcellation of the white surface using Destrieux (a2009s) parcellation	1.04E-01	1.64E-02
1191	27416	aparc-a2009s_lh_thickness_G-front-inf-Triangul	Destrieux Atlas	cortical thickness	mm	Mean thickness of G-front-inf-Triangul in the left hemisphere generated by parcellation of the white surface using Destrieux (a2009s) parcellation	1.62E-01	1.78E-02
1192	27417	aparc-a2009s_lh_thickness_G-front-middle	Destrieux Atlas	cortical thickness	mm	Mean thickness of G-front-middle in the left hemisphere generated by parcellation of the white surface using Destrieux (a2009s) parcellation	2.11E-01	2.05E-02
1193	27418	aparc-a2009s_lh_thickness_G-front-sup	Destrieux Atlas	cortical thickness	mm	Mean thickness of G-front-sup in the left hemisphere generated by parcellation of the white surface using Destrieux (a2009s) parcellation	2.20E-01	2.28E-02

1194	27419	aparc-a2009s_lh_thickness_G-Ins-lg+S-cent-ins	Destrieux Atlas	cortical thickness	mm	Mean thickness of G-Ins-lg+S-cent-ins in the left hemisphere generated by parcellation of the white surface using Destrieux (a2009s) parcellation	9.23E-02	1.54E-02
1195	27420	aparc-a2009s_lh_thickness_G-insular-short	Destrieux Atlas	cortical thickness	mm	Mean thickness of G-insular-short in the left hemisphere generated by parcellation of the white surface using Destrieux (a2009s) parcellation	8.37E-02	1.44E-02
1196	27421	aparc-a2009s_lh_thickness_G-occipital-middle	Destrieux Atlas	cortical thickness	mm	Mean thickness of G-occipital-middle in the left hemisphere generated by parcellation of the white surface using Destrieux (a2009s) parcellation	1.44E-01	1.75E-02
1197	27422	aparc-a2009s_lh_thickness_G-occipital-sup	Destrieux Atlas	cortical thickness	mm	Mean thickness of G-occipital-sup in the left hemisphere generated by parcellation of the white surface using Destrieux (a2009s) parcellation	1.40E-01	1.75E-02
1198	27423	aparc-a2009s_lh_thickness_G-oc-temp-lat-fusifor	Destrieux Atlas	cortical thickness	mm	Mean thickness of G-oc-temp-lat-fusifor in the left hemisphere generated by parcellation of the white surface using Destrieux (a2009s) parcellation	1.39E-01	2.01E-02
1199	27424	aparc-a2009s_lh_thickness_G-oc-temp-med-Lingual	Destrieux Atlas	cortical thickness	mm	Mean thickness of G-oc-temp-med-Lingual in the left hemisphere generated by parcellation of the white surface using Destrieux (a2009s) parcellation	1.69E-01	1.84E-02
1200	27425	aparc-a2009s_lh_thickness_G-oc-temp-med-Parahip	Destrieux Atlas	cortical thickness	mm	Mean thickness of G-oc-temp-med-Parahip in the left hemisphere generated by parcellation of the white surface using Destrieux (a2009s) parcellation	1.38E-01	1.82E-02
1201	27426	aparc-a2009s_lh_thickness_G-orbital	Destrieux Atlas	cortical thickness	mm	Mean thickness of G-orbital in the left hemisphere generated by parcellation of the white surface using Destrieux (a2009s) parcellation	1.21E-01	1.58E-02
1202	27427	aparc-a2009s_lh_thickness_G-pariet-inf-Angular	Destrieux Atlas	cortical thickness	mm	Mean thickness of G-pariet-inf-Angular in the left hemisphere generated by parcellation of the white surface using Destrieux (a2009s) parcellation	1.63E-01	1.91E-02
1203	27428	aparc-a2009s_lh_thickness_G-pariet-inf-Supramar	Destrieux Atlas	cortical thickness	mm	Mean thickness of G-pariet-inf-Supramar in the left hemisphere generated by parcellation of the white surface using Destrieux (a2009s) parcellation	1.92E-01	2.24E-02

1204	27429	aparc-a2009s_lh_thickness_G-parietal-sup	Destrieux Atlas	cortical thickness	mm	Mean thickness of G-parietal-sup in the left hemisphere generated by parcellation of the white surface using Destrieux (a2009s) parcellation	1.75E-01	1.84E-02
1205	27430	aparc-a2009s_lh_thickness_G-postcentral	Destrieux Atlas	cortical thickness	mm	Mean thickness of G-postcentral in the left hemisphere generated by parcellation of the white surface using Destrieux (a2009s) parcellation	1.79E-01	1.93E-02
1206	27431	aparc-a2009s_lh_thickness_G-precentral	Destrieux Atlas	cortical thickness	mm	Mean thickness of G-precentral in the left hemisphere generated by parcellation of the white surface using Destrieux (a2009s) parcellation	1.83E-01	2.19E-02
1207	27432	aparc-a2009s_lh_thickness_G-precuneus	Destrieux Atlas	cortical thickness	mm	Mean thickness of G-precuneus in the left hemisphere generated by parcellation of the white surface using Destrieux (a2009s) parcellation	1.92E-01	2.00E-02
1208	27433	aparc-a2009s_lh_thickness_G-rectus	Destrieux Atlas	cortical thickness	mm	Mean thickness of G-rectus in the left hemisphere generated by parcellation of the white surface using Destrieux (a2009s) parcellation	9.39E-02	1.73E-02
1209	27434	aparc-a2009s_lh_thickness_G-subcallosal	Destrieux Atlas	cortical thickness	mm	Mean thickness of G-subcallosal in the left hemisphere generated by parcellation of the white surface using Destrieux (a2009s) parcellation	1.06E-01	1.75E-02
1210	27435	aparc-a2009s_lh_thickness_G-temp-sup-G-T-transv	Destrieux Atlas	cortical thickness	mm	Mean thickness of G-temp-sup-G-T-transv in the left hemisphere generated by parcellation of the white surface using Destrieux (a2009s) parcellation	1.67E-01	1.78E-02
1211	27436	aparc-a2009s_lh_thickness_G-temp-sup-Lateral	Destrieux Atlas	cortical thickness	mm	Mean thickness of G-temp-sup-Lateral in the left hemisphere generated by parcellation of the white surface using Destrieux (a2009s) parcellation	2.03E-01	1.99E-02
1212	27437	aparc-a2009s_lh_thickness_G-temp-sup-Plan-polar	Destrieux Atlas	cortical thickness	mm	Mean thickness of G-temp-sup-Plan-polar in the left hemisphere generated by parcellation of the white surface using Destrieux (a2009s) parcellation	8.87E-02	1.58E-02
1213	27438	aparc-a2009s_lh_thickness_G-temp-sup-Plan-tempo	Destrieux Atlas	cortical thickness	mm	Mean thickness of G-temp-sup-Plan-tempo in the left hemisphere generated by parcellation of the white surface using Destrieux (a2009s) parcellation	1.47E-01	2.10E-02

1214	27439	aparc-a2009s_lh_thickness_G-temporal-inf	Destrieux Atlas	cortical thickness	mm	Mean thickness of G-temporal-inf in the left hemisphere generated by parcellation of the white surface using Destrieux (a2009s) parcellation	1.14E-01	1.57E-02
1215	27440	aparc-a2009s_lh_thickness_G-temporal-middle	Destrieux Atlas	cortical thickness	mm	Mean thickness of G-temporal-middle in the left hemisphere generated by parcellation of the white surface using Destrieux (a2009s) parcellation	1.50E-01	1.72E-02
1216	27441	aparc-a2009s_lh_thickness_Lat-Fis-ant-Horizont	Destrieux Atlas	cortical thickness	mm	Mean thickness of Lat-Fis-ant-Horizont in the left hemisphere generated by parcellation of the white surface using Destrieux (a2009s) parcellation	1.00E-01	1.50E-02
1217	27442	aparc-a2009s_lh_thickness_Lat-Fis-ant-Vertical	Destrieux Atlas	cortical thickness	mm	Mean thickness of Lat-Fis-ant-Vertical in the left hemisphere generated by parcellation of the white surface using Destrieux (a2009s) parcellation	7.36E-02	1.43E-02
1218	27443	aparc-a2009s_lh_thickness_Lat-Fis-post	Destrieux Atlas	cortical thickness	mm	Mean thickness of Lat-Fis-post in the left hemisphere generated by parcellation of the white surface using Destrieux (a2009s) parcellation	2.10E-01	2.35E-02
1219	27444	aparc-a2009s_lh_thickness_Pole-occipital	Destrieux Atlas	cortical thickness	mm	Mean thickness of Pole-occipital in the left hemisphere generated by parcellation of the white surface using Destrieux (a2009s) parcellation	1.19E-01	1.61E-02
1220	27445	aparc-a2009s_lh_thickness_Pole-temporal	Destrieux Atlas	cortical thickness	mm	Mean thickness of Pole-temporal in the left hemisphere generated by parcellation of the white surface using Destrieux (a2009s) parcellation	9.72E-02	1.58E-02
1221	27446	aparc-a2009s_lh_thickness_S-calcarine	Destrieux Atlas	cortical thickness	mm	Mean thickness of S-calcarine in the left hemisphere generated by parcellation of the white surface using Destrieux (a2009s) parcellation	1.40E-01	1.69E-02
1222	27447	aparc-a2009s_lh_thickness_S-central	Destrieux Atlas	cortical thickness	mm	Mean thickness of S-central in the left hemisphere generated by parcellation of the white surface using Destrieux (a2009s) parcellation	1.55E-01	1.84E-02
1223	27448	aparc-a2009s_lh_thickness_S-cingul-Marginalis	Destrieux Atlas	cortical thickness	mm	Mean thickness of S-cingul-Marginalis in the left hemisphere generated by parcellation of the white surface using Destrieux (a2009s) parcellation	1.23E-01	1.90E-02

1224	27449	aparc-a2009s_lh_thickness_S-circular-insula-ant	Destrieux Atlas	cortical thickness	mm	Mean thickness of S-circular-insula-ant in the left hemisphere generated by parcellation of the white surface using Destrieux (a2009s) parcellation	6.46E-02	1.45E-02
1225	27450	aparc-a2009s_lh_thickness_S-circular-insula-inf	Destrieux Atlas	cortical thickness	mm	Mean thickness of S-circular-insula-inf in the left hemisphere generated by parcellation of the white surface using Destrieux (a2009s) parcellation	1.37E-01	1.97E-02
1226	27451	aparc-a2009s_lh_thickness_S-circular-insula-sup	Destrieux Atlas	cortical thickness	mm	Mean thickness of S-circular-insula-sup in the left hemisphere generated by parcellation of the white surface using Destrieux (a2009s) parcellation	1.59E-01	2.01E-02
1227	27452	aparc-a2009s_lh_thickness_S-collat-transv-ant	Destrieux Atlas	cortical thickness	mm	Mean thickness of S-collat-transv-ant in the left hemisphere generated by parcellation of the white surface using Destrieux (a2009s) parcellation	7.20E-02	1.61E-02
1228	27453	aparc-a2009s_lh_thickness_S-collat-transv-post	Destrieux Atlas	cortical thickness	mm	Mean thickness of S-collat-transv-post in the left hemisphere generated by parcellation of the white surface using Destrieux (a2009s) parcellation	1.14E-01	1.80E-02
1229	27454	aparc-a2009s_lh_thickness_S-front-inf	Destrieux Atlas	cortical thickness	mm	Mean thickness of S-front-inf in the left hemisphere generated by parcellation of the white surface using Destrieux (a2009s) parcellation	1.74E-01	1.81E-02
1230	27455	aparc-a2009s_lh_thickness_S-front-middle	Destrieux Atlas	cortical thickness	mm	Mean thickness of S-front-middle in the left hemisphere generated by parcellation of the white surface using Destrieux (a2009s) parcellation	1.18E-01	1.58E-02
1231	27456	aparc-a2009s_lh_thickness_S-front-sup	Destrieux Atlas	cortical thickness	mm	Mean thickness of S-front-sup in the left hemisphere generated by parcellation of the white surface using Destrieux (a2009s) parcellation	1.88E-01	2.21E-02
1232	27457	aparc-a2009s_lh_thickness_S-interm-prim-Jense n	Destrieux Atlas	cortical thickness	mm	Mean thickness of S-interm-prim-Jensen in the left hemisphere generated by parcellation of the white surface using Destrieux (a2009s) parcellation	5.43E-02	1.47E-02
1233	27458	aparc-a2009s_lh_thickness_S-intrapariet+P-trans	Destrieux Atlas	cortical thickness	mm	Mean thickness of S-intrapariet+P-trans in the left hemisphere generated by parcellation of the white surface using Destrieux (a2009s) parcellation	1.78E-01	1.84E-02

1234	27459	aparc-a2009s_lh_thickness_S-oc-middle+Lunatus	Destrieux Atlas	cortical thickness	mm	Mean thickness of S-oc-middle+Lunatus in the left hemisphere generated by parcellation of the white surface using Destrieux (a2009s) parcellation	1.05E-01	1.62E-02
1235	27460	aparc-a2009s_lh_thickness_S-oc-sup+transversal	Destrieux Atlas	cortical thickness	mm	Mean thickness of S-oc-sup+transversal in the left hemisphere generated by parcellation of the white surface using Destrieux (a2009s) parcellation	1.62E-01	1.84E-02
1236	27461	aparc-a2009s_lh_thickness_S-occipital-ant	Destrieux Atlas	cortical thickness	mm	Mean thickness of S-occipital-ant in the left hemisphere generated by parcellation of the white surface using Destrieux (a2009s) parcellation	8.26E-02	1.60E-02
1237	27462	aparc-a2009s_lh_thickness_S-oc-temp-lat	Destrieux Atlas	cortical thickness	mm	Mean thickness of S-oc-temp-lat in the left hemisphere generated by parcellation of the white surface using Destrieux (a2009s) parcellation	1.12E-01	1.57E-02
1238	27463	aparc-a2009s_lh_thickness_S-oc-temp-med+Lingual	Destrieux Atlas	cortical thickness	mm	Mean thickness of S-oc-temp-med+Lingual in the left hemisphere generated by parcellation of the white surface using Destrieux (a2009s) parcellation	2.07E-01	1.72E-02
1239	27464	aparc-a2009s_lh_thickness_S-orbital-lateral	Destrieux Atlas	cortical thickness	mm	Mean thickness of S-orbital-lateral in the left hemisphere generated by parcellation of the white surface using Destrieux (a2009s) parcellation	5.34E-02	1.62E-02
1240	27465	aparc-a2009s_lh_thickness_S-orbital-med-olfact	Destrieux Atlas	cortical thickness	mm	Mean thickness of S-orbital-med-olfact in the left hemisphere generated by parcellation of the white surface using Destrieux (a2009s) parcellation	1.10E-01	1.60E-02
1241	27466	aparc-a2009s_lh_thickness_S-orbital-H-Shaped	Destrieux Atlas	cortical thickness	mm	Mean thickness of S-orbital-H-Shaped in the left hemisphere generated by parcellation of the white surface using Destrieux (a2009s) parcellation	1.15E-01	1.66E-02
1242	27467	aparc-a2009s_lh_thickness_S-parieto-occipital	Destrieux Atlas	cortical thickness	mm	Mean thickness of S-parieto-occipital in the left hemisphere generated by parcellation of the white surface using Destrieux (a2009s) parcellation	1.55E-01	1.81E-02
1243	27468	aparc-a2009s_lh_thickness_S-pericallosal	Destrieux Atlas	cortical thickness	mm	Mean thickness of S-pericallosal in the left hemisphere generated by parcellation of the white surface using Destrieux (a2009s) parcellation	1.64E-01	1.77E-02

1244	27469	aparc-a2009s_lh_thickness_S-postcentral	Destrieux Atlas	cortical thickness	mm	Mean thickness of S-postcentral in the left hemisphere generated by parcellation of the white surface using Destrieux (a2009s) parcellation	1.49E-01	1.84E-02
1245	27470	aparc-a2009s_lh_thickness_S-precentral-inf-part	Destrieux Atlas	cortical thickness	mm	Mean thickness of S-precentral-inf-part in the left hemisphere generated by parcellation of the white surface using Destrieux (a2009s) parcellation	1.56E-01	1.77E-02
1246	27471	aparc-a2009s_lh_thickness_S-precentral-sup-part	Destrieux Atlas	cortical thickness	mm	Mean thickness of S-precentral-sup-part in the left hemisphere generated by parcellation of the white surface using Destrieux (a2009s) parcellation	1.54E-01	1.73E-02
1247	27472	aparc-a2009s_lh_thickness_S-suborbital	Destrieux Atlas	cortical thickness	mm	Mean thickness of S-suborbital in the left hemisphere generated by parcellation of the white surface using Destrieux (a2009s) parcellation	8.32E-02	1.57E-02
1248	27473	aparc-a2009s_lh_thickness_S-subparietal	Destrieux Atlas	cortical thickness	mm	Mean thickness of S-subparietal in the left hemisphere generated by parcellation of the white surface using Destrieux (a2009s) parcellation	1.36E-01	1.50E-02
1249	27474	aparc-a2009s_lh_thickness_S-temporal-inf	Destrieux Atlas	cortical thickness	mm	Mean thickness of S-temporal-inf in the left hemisphere generated by parcellation of the white surface using Destrieux (a2009s) parcellation	1.03E-01	1.62E-02
1250	27475	aparc-a2009s_lh_thickness_S-temporal-sup	Destrieux Atlas	cortical thickness	mm	Mean thickness of S-temporal-sup in the left hemisphere generated by parcellation of the white surface using Destrieux (a2009s) parcellation	1.50E-01	1.87E-02
1251	27476	aparc-a2009s_lh_thickness_S-temporal-transverse	Destrieux Atlas	cortical thickness	mm	Mean thickness of S-temporal-transverse in the left hemisphere generated by parcellation of the white surface using Destrieux (a2009s) parcellation	1.48E-01	1.72E-02
1252	27625	aparc-a2009s_rh_thickness_G+S-frontomargin	Destrieux Atlas	cortical thickness	mm	Mean thickness of G+S-frontomargin in the right hemisphere generated by parcellation of the white surface using Destrieux (a2009s) parcellation	9.28E-02	1.49E-02
1253	27626	aparc-a2009s_rh_thickness_G+S-occipital-inf	Destrieux Atlas	cortical thickness	mm	Mean thickness of G+S-occipital-inf in the right hemisphere generated by parcellation of the white surface using Destrieux (a2009s) parcellation	1.44E-01	1.95E-02

1254	27627	aparc-a2009s_rh_thickness_G+S-paracentral	Destrieux Atlas	cortical thickness	mm	Mean thickness of G+S-paracentral in the right hemisphere generated by parcellation of the white surface using Destrieux (a2009s) parcellation	1.68E-01	1.95E-02
1255	27628	aparc-a2009s_rh_thickness_G+S-subcentral	Destrieux Atlas	cortical thickness	mm	Mean thickness of G+S-subcentral in the right hemisphere generated by parcellation of the white surface using Destrieux (a2009s) parcellation	1.98E-01	1.91E-02
1256	27629	aparc-a2009s_rh_thickness_G+S-transv-frontopol	Destrieux Atlas	cortical thickness	mm	Mean thickness of G+S-transv-frontopol in the right hemisphere generated by parcellation of the white surface using Destrieux (a2009s) parcellation	1.11E-01	1.49E-02
1257	27630	aparc-a2009s_rh_thickness_G+S-cingul-Ant	Destrieux Atlas	cortical thickness	mm	Mean thickness of G+S-cingul-Ant in the right hemisphere generated by parcellation of the white surface using Destrieux (a2009s) parcellation	1.30E-01	1.87E-02
1258	27631	aparc-a2009s_rh_thickness_G+S-cingul-Mid-Ant	Destrieux Atlas	cortical thickness	mm	Mean thickness of G+S-cingul-Mid-Ant in the right hemisphere generated by parcellation of the white surface using Destrieux (a2009s) parcellation	9.60E-02	1.75E-02
1259	27632	aparc-a2009s_rh_thickness_G+S-cingul-Mid-Post	Destrieux Atlas	cortical thickness	mm	Mean thickness of G+S-cingul-Mid-Post in the right hemisphere generated by parcellation of the white surface using Destrieux (a2009s) parcellation	1.31E-01	1.83E-02
1260	27633	aparc-a2009s_rh_thickness_G-cingul-Post-dorsal	Destrieux Atlas	cortical thickness	mm	Mean thickness of G-cingul-Post-dorsal in the right hemisphere generated by parcellation of the white surface using Destrieux (a2009s) parcellation	1.29E-01	1.76E-02
1261	27634	aparc-a2009s_rh_thickness_G-cingul-Post-ventral	Destrieux Atlas	cortical thickness	mm	Mean thickness of G-cingul-Post-ventral in the right hemisphere generated by parcellation of the white surface using Destrieux (a2009s) parcellation	1.21E-01	1.73E-02
1262	27635	aparc-a2009s_rh_thickness_G-cuneus	Destrieux Atlas	cortical thickness	mm	Mean thickness of G-cuneus in the right hemisphere generated by parcellation of the white surface using Destrieux (a2009s) parcellation	1.62E-01	1.84E-02
1263	27636	aparc-a2009s_rh_thickness_G-front-inf-Opercular	Destrieux Atlas	cortical thickness	mm	Mean thickness of G-front-inf-Opercular in the right hemisphere generated by parcellation of the white surface using Destrieux (a2009s) parcellation	1.46E-01	1.75E-02

1264	27637	aparc-a2009s_rh_thickness_G-front-inf-Orbital	Destrieux Atlas	cortical thickness	mm	Mean thickness of G-front-inf-Orbital in the right hemisphere generated by parcellation of the white surface using Destrieux (a2009s) parcellation	1.06E-01	1.83E-02
1265	27638	aparc-a2009s_rh_thickness_G-front-inf-Triangul	Destrieux Atlas	cortical thickness	mm	Mean thickness of G-front-inf-Triangul in the right hemisphere generated by parcellation of the white surface using Destrieux (a2009s) parcellation	1.44E-01	1.77E-02
1266	27639	aparc-a2009s_rh_thickness_G-front-middle	Destrieux Atlas	cortical thickness	mm	Mean thickness of G-front-middle in the right hemisphere generated by parcellation of the white surface using Destrieux (a2009s) parcellation	1.77E-01	1.88E-02
1267	27640	aparc-a2009s_rh_thickness_G-front-sup	Destrieux Atlas	cortical thickness	mm	Mean thickness of G-front-sup in the right hemisphere generated by parcellation of the white surface using Destrieux (a2009s) parcellation	2.23E-01	2.06E-02
1268	27641	aparc-a2009s_rh_thickness_G-Ins-lg+S-cent-ins	Destrieux Atlas	cortical thickness	mm	Mean thickness of G-Ins-lg+S-cent-ins in the right hemisphere generated by parcellation of the white surface using Destrieux (a2009s) parcellation	9.86E-02	1.62E-02
1269	27642	aparc-a2009s_rh_thickness_G-insular-short	Destrieux Atlas	cortical thickness	mm	Mean thickness of G-insular-short in the right hemisphere generated by parcellation of the white surface using Destrieux (a2009s) parcellation	1.06E-01	1.51E-02
1270	27643	aparc-a2009s_rh_thickness_G-occipital-middle	Destrieux Atlas	cortical thickness	mm	Mean thickness of G-occipital-middle in the right hemisphere generated by parcellation of the white surface using Destrieux (a2009s) parcellation	1.49E-01	1.87E-02
1271	27644	aparc-a2009s_rh_thickness_G-occipital-sup	Destrieux Atlas	cortical thickness	mm	Mean thickness of G-occipital-sup in the right hemisphere generated by parcellation of the white surface using Destrieux (a2009s) parcellation	1.33E-01	1.62E-02
1272	27645	aparc-a2009s_rh_thickness_G-oc-temp-lat-fusifor	Destrieux Atlas	cortical thickness	mm	Mean thickness of G-oc-temp-lat-fusifor in the right hemisphere generated by parcellation of the white surface using Destrieux (a2009s) parcellation	1.40E-01	2.05E-02
1273	27646	aparc-a2009s_rh_thickness_G-oc-temp-med-Lingual	Destrieux Atlas	cortical thickness	mm	Mean thickness of G-oc-temp-med-Lingual in the right hemisphere generated by parcellation of the white surface using Destrieux (a2009s) parcellation	1.60E-01	1.90E-02

1274	27647	aparc-a2009s_rh_thickness_G-oc-temp-med-Parahip	Destrieux Atlas	cortical thickness	mm	Mean thickness of G-oc-temp-med-Parahip in the right hemisphere generated by parcellation of the white surface using Destrieux (a2009s) parcellation	9.70E-02	1.71E-02
1275	27648	aparc-a2009s_rh_thickness_G-orbital	Destrieux Atlas	cortical thickness	mm	Mean thickness of G-orbital in the right hemisphere generated by parcellation of the white surface using Destrieux (a2009s) parcellation	1.03E-01	1.80E-02
1276	27649	aparc-a2009s_rh_thickness_G-pariet-inf-Angular	Destrieux Atlas	cortical thickness	mm	Mean thickness of G-pariet-inf-Angular in the right hemisphere generated by parcellation of the white surface using Destrieux (a2009s) parcellation	1.75E-01	2.03E-02
1277	27650	aparc-a2009s_rh_thickness_G-pariet-inf-Supramar	Destrieux Atlas	cortical thickness	mm	Mean thickness of G-pariet-inf-Supramar in the right hemisphere generated by parcellation of the white surface using Destrieux (a2009s) parcellation	1.89E-01	2.10E-02
1278	27651	aparc-a2009s_rh_thickness_G-parietal-sup	Destrieux Atlas	cortical thickness	mm	Mean thickness of G-parietal-sup in the right hemisphere generated by parcellation of the white surface using Destrieux (a2009s) parcellation	1.44E-01	1.88E-02
1279	27652	aparc-a2009s_rh_thickness_G-postcentral	Destrieux Atlas	cortical thickness	mm	Mean thickness of G-postcentral in the right hemisphere generated by parcellation of the white surface using Destrieux (a2009s) parcellation	1.74E-01	2.14E-02
1280	27653	aparc-a2009s_rh_thickness_G-precentral	Destrieux Atlas	cortical thickness	mm	Mean thickness of G-precentral in the right hemisphere generated by parcellation of the white surface using Destrieux (a2009s) parcellation	1.74E-01	1.96E-02
1281	27654	aparc-a2009s_rh_thickness_G-precuneus	Destrieux Atlas	cortical thickness	mm	Mean thickness of G-precuneus in the right hemisphere generated by parcellation of the white surface using Destrieux (a2009s) parcellation	1.93E-01	2.05E-02
1282	27655	aparc-a2009s_rh_thickness_G-rectus	Destrieux Atlas	cortical thickness	mm	Mean thickness of G-rectus in the right hemisphere generated by parcellation of the white surface using Destrieux (a2009s) parcellation	8.10E-02	1.56E-02
1283	27656	aparc-a2009s_rh_thickness_G-subcallosal	Destrieux Atlas	cortical thickness	mm	Mean thickness of G-subcallosal in the right hemisphere generated by parcellation of the white surface using Destrieux (a2009s) parcellation	4.47E-02	1.71E-02

1284	27657	aparc-a2009s_rh_thickness_G-temp-sup-G-T-transv	Destrieux Atlas	cortical thickness	mm	Mean thickness of G-temp-sup-G-T-transv in the right hemisphere generated by parcellation of the white surface using Destrieux (a2009s) parcellation	1.62E-01	2.12E-02
1285	27658	aparc-a2009s_rh_thickness_G-temp-sup-Lateral	Destrieux Atlas	cortical thickness	mm	Mean thickness of G-temp-sup-Lateral in the right hemisphere generated by parcellation of the white surface using Destrieux (a2009s) parcellation	1.97E-01	1.92E-02
1286	27659	aparc-a2009s_rh_thickness_G-temp-sup-Plan-polar	Destrieux Atlas	cortical thickness	mm	Mean thickness of G-temp-sup-Plan-polar in the right hemisphere generated by parcellation of the white surface using Destrieux (a2009s) parcellation	9.58E-02	1.58E-02
1287	27660	aparc-a2009s_rh_thickness_G-temp-sup-Plan-tempo	Destrieux Atlas	cortical thickness	mm	Mean thickness of G-temp-sup-Plan-tempo in the right hemisphere generated by parcellation of the white surface using Destrieux (a2009s) parcellation	1.68E-01	2.01E-02
1288	27661	aparc-a2009s_rh_thickness_G-temporal-inf	Destrieux Atlas	cortical thickness	mm	Mean thickness of G-temporal-inf in the right hemisphere generated by parcellation of the white surface using Destrieux (a2009s) parcellation	1.45E-01	1.72E-02
1289	27662	aparc-a2009s_rh_thickness_G-temporal-middle	Destrieux Atlas	cortical thickness	mm	Mean thickness of G-temporal-middle in the right hemisphere generated by parcellation of the white surface using Destrieux (a2009s) parcellation	1.81E-01	1.98E-02
1290	27663	aparc-a2009s_rh_thickness_Lat-Fis-ant-Horizont	Destrieux Atlas	cortical thickness	mm	Mean thickness of Lat-Fis-ant-Horizont in the right hemisphere generated by parcellation of the white surface using Destrieux (a2009s) parcellation	1.07E-01	1.64E-02
1291	27664	aparc-a2009s_rh_thickness_Lat-Fis-ant-Vertical	Destrieux Atlas	cortical thickness	mm	Mean thickness of Lat-Fis-ant-Vertical in the right hemisphere generated by parcellation of the white surface using Destrieux (a2009s) parcellation	6.46E-02	1.33E-02
1292	27665	aparc-a2009s_rh_thickness_Lat-Fis-post	Destrieux Atlas	cortical thickness	mm	Mean thickness of Lat-Fis-post in the right hemisphere generated by parcellation of the white surface using Destrieux (a2009s) parcellation	1.94E-01	2.42E-02
1293	27666	aparc-a2009s_rh_thickness_Pole-occipital	Destrieux Atlas	cortical thickness	mm	Mean thickness of Pole-occipital in the right hemisphere generated by parcellation of the white surface using Destrieux (a2009s) parcellation	1.56E-01	1.77E-02

1294	27667	aparc-a2009s_rh_thickness_Pole-temporal	Destrieux Atlas	cortical thickness	mm	Mean thickness of Pole-temporal in the right hemisphere generated by parcellation of the white surface using Destrieux (a2009s) parcellation	1.11E-01	1.54E-02
1295	27668	aparc-a2009s_rh_thickness_S-calcarine	Destrieux Atlas	cortical thickness	mm	Mean thickness of S-calcarine in the right hemisphere generated by parcellation of the white surface using Destrieux (a2009s) parcellation	1.52E-01	1.74E-02
1296	27669	aparc-a2009s_rh_thickness_S-central	Destrieux Atlas	cortical thickness	mm	Mean thickness of S-central in the right hemisphere generated by parcellation of the white surface using Destrieux (a2009s) parcellation	1.38E-01	1.91E-02
1297	27670	aparc-a2009s_rh_thickness_S-cingul-Marginalis	Destrieux Atlas	cortical thickness	mm	Mean thickness of S-cingul-Marginalis in the right hemisphere generated by parcellation of the white surface using Destrieux (a2009s) parcellation	1.32E-01	1.91E-02
1298	27671	aparc-a2009s_rh_thickness_S-circular-insula-ant	Destrieux Atlas	cortical thickness	mm	Mean thickness of S-circular-insula-ant in the right hemisphere generated by parcellation of the white surface using Destrieux (a2009s) parcellation	9.99E-02	1.55E-02
1299	27672	aparc-a2009s_rh_thickness_S-circular-insula-inf	Destrieux Atlas	cortical thickness	mm	Mean thickness of S-circular-insula-inf in the right hemisphere generated by parcellation of the white surface using Destrieux (a2009s) parcellation	1.91E-01	2.37E-02
1300	27673	aparc-a2009s_rh_thickness_S-circular-insula-sup	Destrieux Atlas	cortical thickness	mm	Mean thickness of S-circular-insula-sup in the right hemisphere generated by parcellation of the white surface using Destrieux (a2009s) parcellation	1.74E-01	2.62E-02
1301	27674	aparc-a2009s_rh_thickness_S-collat-transv-ant	Destrieux Atlas	cortical thickness	mm	Mean thickness of S-collat-transv-ant in the right hemisphere generated by parcellation of the white surface using Destrieux (a2009s) parcellation	5.30E-02	1.53E-02
1302	27675	aparc-a2009s_rh_thickness_S-collat-transv-post	Destrieux Atlas	cortical thickness	mm	Mean thickness of S-collat-transv-post in the right hemisphere generated by parcellation of the white surface using Destrieux (a2009s) parcellation	1.39E-01	1.59E-02
1303	27676	aparc-a2009s_rh_thickness_S-front-inf	Destrieux Atlas	cortical thickness	mm	Mean thickness of S-front-inf in the right hemisphere generated by parcellation of the white surface using Destrieux (a2009s) parcellation	1.49E-01	1.75E-02

1304	27677	aparc-a2009s_rh_thickness_S-front-middle	Destrieux Atlas	cortical thickness	mm	Mean thickness of S-front-middle in the right hemisphere generated by parcellation of the white surface using Destrieux (a2009s) parcellation	1.32E-01	1.95E-02
1305	27678	aparc-a2009s_rh_thickness_S-front-sup	Destrieux Atlas	cortical thickness	mm	Mean thickness of S-front-sup in the right hemisphere generated by parcellation of the white surface using Destrieux (a2009s) parcellation	1.57E-01	2.08E-02
1306	27679	aparc-a2009s_rh_thickness_S-interm-prim-Jensen	Destrieux Atlas	cortical thickness	mm	Mean thickness of S-interm-prim-Jensen in the right hemisphere generated by parcellation of the white surface using Destrieux (a2009s) parcellation	6.17E-02	1.94E-02
1307	27680	aparc-a2009s_rh_thickness_S-intrapariet+P-trans	Destrieux Atlas	cortical thickness	mm	Mean thickness of S-intrapariet+P-trans in the right hemisphere generated by parcellation of the white surface using Destrieux (a2009s) parcellation	1.55E-01	2.02E-02
1308	27681	aparc-a2009s_rh_thickness_S-oc-middle+Lunatus	Destrieux Atlas	cortical thickness	mm	Mean thickness of S-oc-middle+Lunatus in the right hemisphere generated by parcellation of the white surface using Destrieux (a2009s) parcellation	1.12E-01	1.66E-02
1309	27682	aparc-a2009s_rh_thickness_S-oc-sup+transversal	Destrieux Atlas	cortical thickness	mm	Mean thickness of S-oc-sup+transversal in the right hemisphere generated by parcellation of the white surface using Destrieux (a2009s) parcellation	1.62E-01	1.64E-02
1310	27683	aparc-a2009s_rh_thickness_S-occipital-ant	Destrieux Atlas	cortical thickness	mm	Mean thickness of S-occipital-ant in the right hemisphere generated by parcellation of the white surface using Destrieux (a2009s) parcellation	1.18E-01	1.69E-02
1311	27684	aparc-a2009s_rh_thickness_S-oc-temp-lat	Destrieux Atlas	cortical thickness	mm	Mean thickness of S-oc-temp-lat in the right hemisphere generated by parcellation of the white surface using Destrieux (a2009s) parcellation	1.13E-01	1.58E-02
1312	27685	aparc-a2009s_rh_thickness_S-oc-temp-med+Lingual	Destrieux Atlas	cortical thickness	mm	Mean thickness of S-oc-temp-med+Lingual in the right hemisphere generated by parcellation of the white surface using Destrieux (a2009s) parcellation	1.89E-01	1.78E-02
1313	27686	aparc-a2009s_rh_thickness_S-orbital-lateral	Destrieux Atlas	cortical thickness	mm	Mean thickness of S-orbital-lateral in the right hemisphere generated by parcellation of the white surface using Destrieux (a2009s) parcellation	7.83E-02	1.55E-02

1314	27687	aparc-a2009s_rh_thickness_S-orbital-med-olfact	Destrieux Atlas	cortical thickness	mm	Mean thickness of S-orbital-med-olfact in the right hemisphere generated by parcellation of the white surface using Destrieux (a2009s) parcellation	1.32E-01	1.53E-02
1315	27688	aparc-a2009s_rh_thickness_S-orbital-H-Shaped	Destrieux Atlas	cortical thickness	mm	Mean thickness of S-orbital-H-Shaped in the right hemisphere generated by parcellation of the white surface using Destrieux (a2009s) parcellation	9.94E-02	1.65E-02
1316	27689	aparc-a2009s_rh_thickness_S-parieto-occipital	Destrieux Atlas	cortical thickness	mm	Mean thickness of S-parieto-occipital in the right hemisphere generated by parcellation of the white surface using Destrieux (a2009s) parcellation	1.69E-01	1.91E-02
1317	27690	aparc-a2009s_rh_thickness_S-pericallosal	Destrieux Atlas	cortical thickness	mm	Mean thickness of S-pericallosal in the right hemisphere generated by parcellation of the white surface using Destrieux (a2009s) parcellation	2.11E-01	2.06E-02
1318	27691	aparc-a2009s_rh_thickness_S-postcentral	Destrieux Atlas	cortical thickness	mm	Mean thickness of S-postcentral in the right hemisphere generated by parcellation of the white surface using Destrieux (a2009s) parcellation	1.39E-01	1.88E-02
1319	27692	aparc-a2009s_rh_thickness_S-precentral-inf-part	Destrieux Atlas	cortical thickness	mm	Mean thickness of S-precentral-inf-part in the right hemisphere generated by parcellation of the white surface using Destrieux (a2009s) parcellation	1.52E-01	2.03E-02
1320	27693	aparc-a2009s_rh_thickness_S-precentral-sup-part	Destrieux Atlas	cortical thickness	mm	Mean thickness of S-precentral-sup-part in the right hemisphere generated by parcellation of the white surface using Destrieux (a2009s) parcellation	1.49E-01	1.60E-02
1321	27694	aparc-a2009s_rh_thickness_S-suborbital	Destrieux Atlas	cortical thickness	mm	Mean thickness of S-suborbital in the right hemisphere generated by parcellation of the white surface using Destrieux (a2009s) parcellation	4.75E-02	1.65E-02
1322	27695	aparc-a2009s_rh_thickness_S-subparietal	Destrieux Atlas	cortical thickness	mm	Mean thickness of S-subparietal in the right hemisphere generated by parcellation of the white surface using Destrieux (a2009s) parcellation	1.40E-01	1.95E-02
1323	27696	aparc-a2009s_rh_thickness_S-temporal-inf	Destrieux Atlas	cortical thickness	mm	Mean thickness of S-temporal-inf in the right hemisphere generated by parcellation of the white surface using Destrieux (a2009s) parcellation	8.32E-02	1.51E-02

1324	27697	aparc-a2009s_rh_thickness_S-temporal-sup	Destrieux Atlas	cortical thickness	mm	Mean thickness of S-temporal-sup in the right hemisphere generated by parcellation of the white surface using Destrieux (a2009s) parcellation	1.76E-01	1.86E-02
1325	27698	aparc-a2009s_rh_thickness_S-temporal-transverse	Destrieux Atlas	cortical thickness	mm	Mean thickness of S-temporal-transverse in the right hemisphere generated by parcellation of the white surface using Destrieux (a2009s) parcellation	1.53E-01	2.57E-02
